# Genome-Wide Identification, Phylogeny, Duplication, and Expression Analyses of Two-Component System Genes in Chinese Cabbage (*Brassica rapa* ssp. *pekinensis*)

**DOI:** 10.1093/dnares/dsu004

**Published:** 2014-02-27

**Authors:** Zhenning Liu, Mei Zhang, Lijun Kong, Yanxia Lv, Minghua Zou, Gang Lu, Jiashu Cao, Xiaolin Yu

**Affiliations:** 1 Laboratory of Cell and Molecular Biology, Institute of Vegetable Science, Zhejiang University, 866 Yuhangtang Road, Hangzhou 310058, PR China; 2 Laboratory of Horticultural Plant Growth and Quality Regulation, Ministry of Agriculture, Hangzhou 310058, PR China

**Keywords:** two-component system, Chinese cabbage, phylogeny, evolution, expression

## Abstract

In plants, a two component system (TCS) composed of sensor histidine kinases (HKs), histidine phosphotransfer proteins (HPs), and response regulators (RRs) has been employed in cytokinin signal transduction. A TCS exhibits important functions in diverse biological processes, including plant growth, development, and response to environmental stimuli. Conducting an exhaustive search of the Chinese cabbage genome, a total of 20 HK(L) (11 HKs and 9 HKLs), 8 HP (7 authentic and 1 pseudo), and 57 RR (21 Type-A, 17 Type-B, 4 Type-C, and 15 pseudo) proteins were identified. The structures, conserved domains, and phylogenetic relationships of these protein-coding genes were analysed in detail. The duplications, evolutionary patterns, and divergence of the TCS genes were investigated. The transcription levels of TCS genes in various tissues, organs, and developmental stages were further analysed to obtain information of the functions of these genes. Cytokinin-related binding elements were found in the putative promoter regions of Type-A *BrRR* genes. Furthermore, gene expression patterns to adverse environmental stresses (drought and high salinity) and exogenous phytohormones (tZ and ABA) were investigated. Numerous stress-responsive candidate genes were obtained. Our systematic analyses provided insights into the characterization of the TCS genes in Chinese cabbage and basis for further functional studies of such genes.

## 1. Introduction

Cytokinins are *N*^6^-substituted adenine derivatives that have significant functions in various aspects of plant growth and development, including apical dominance, shoot or root branching, leaf expansion, lateral bud growth, photosynthesis, seed germination, floral transition, and leaf senescence.^1^ Cytokinin signal transduction is mediated by a two-component system (TCS), which is similar to the two-component phosphorelay system that allows bacteria to sense and respond to environmental changes.^2^ Although this system was originally discovered in bacteria, two-component signalling elements have also been identified in fungi, slime molds, and plants. However, a canonical histidyl-aspartyl phosphorelay is not found in animals.^3–6^ A simple TCS involves a histidine (His) sensor kinase and a response regulator (RR)^6,7^ His kinase (HK) perceives environmental stimuli via an input domain and autophosphorylates in a conserved His residue in a kinase domain. This phosphoryl group is then transferred to a conserved Asp residue on the receiver (Rec) domain of an RR. An RR undergoes phosphorylation, thereby modulating its ability to mediate downstream signalling. Eukaryotes, such as yeast and plants, have evolved a more complex multi-step TCS with additional phosphorylation steps and His phosphotransfer (HP) proteins.^8^ Multi-component phosphorelay systems employ HK signal transduction in a multi-step His-Asp-His-Asp phosphotransfer process.

Genes involved in the TCS have been extensively studied in *Arabidopsis*. The completion of the *Arabidopsis* genome sequence has revealed 55 genes encoding putative HK (AHK), HP (AHP), RR (ARR), and related proteins. *Arabidopsis* HKs are grouped into three main subfamilies: cytokinin receptor; ethylene receptor; and phytochrome. In addition, three HKs (CKI1, AHK5, and AHK1) are present in *Arabidopsis*; such kinases do not belong in these groups. The overall structures comprise a hybrid sensor HK containing a variable input domain, several N-terminal transmembrane domains, and a transmitter domain with a conserved structure, which includes the His residue that is the site of autophosphorylation, and a fused Rec domain.^9^ However, the transmitters of three ethylene receptors (ETR2, ERS2, and EIN4) and phytochromes lack key residues in highly conserved HK sequence motifs; thus, these transmitters unlikely perform HK activity. For this reason, these molecules are called diverged HKs of two-component elements.^10–13^ Moreover, three cytokinin receptors (AHK2, AHK3, and AHK4) share a cyclase/HK-associated sensory extracellular (CHASE) domain, which is a putative recognition site for cytokinins.^14^ Five ethylene receptors (ETR1, ERS1, ETR2, ERS2, and EIN4) also share an ethylene-binding domain (C2H4). Five phytochromes (PHYA, B, C, D, and E) are characterized by a chromophore-binding domain (PHY) and two PAS (Per/Arndt/Sim) folds. AHPs, which contain a highly conserved xHQxKGSSxS motif, can mediate the transfer of a phosphate group from the Rec domain of an AHK to the Rec domain of an ARR.^15^ AHP6 is a pseudo-His-containing phosphotransfer (HPt) protein without a conserved histidine residue; thus, AHP6 cannot function as a phosphotransfer protein. In fact, AHP6 functions as a negative regulator of cytokinin signalling by inhibiting phosphorelay from phosphorylated AHP1 to ARR1.^16^ The canonical members of ARRs can be divided into two major classes: Type-A ARRs and Type-B ARRs, according to their amino acid sequences and conserved domains. Type-A ARRs are primary cytokinin response proteins comprising a Rec domain along with short C-terminal extensions. Type-B ARRs are composed of an N-terminal Rec domain and a large C-terminal output domain with a Type-B signature GARP (GOLDEN/ARR/Psr1) motif of ∼60 amino acids^17^ that are distantly related to the Myb DNA-binding superfamily.^18,19^ Type-C ARRs are also present, and the members of this protein class were originally considered as Type-A ARRs because these molecules have the same domain structure.^20^ In contrast to the Type-A *ARR* genes, Type-C *ARR* genes cannot be induced by cytokinin,^20–22^ and their functions in the cytokinin signalling pathway remain unknown. In addition, diverge RRs, referred to as pseudo-RRs (PRRs), lack the conserved Asp for phosphorylation.^23^ As a highly characterized pseudo-RR, Clock PRR contains a distinct plant-specific Co, Col and Toc1 (CCT) motif in their C-terminal extensions and functions in the regulation of circadian rhythms.^24–26^

Abiotic stress conditions, such as drought and high salinity, are two of the most common stress factors that adversely affect plant growth and yield. Plants have evolved a complex signalling network at molecular, cellular, and system levels to survive and flourish in varied environments.^27^ Phosphorylation, which is mediated by TCS genes, is a key mechanism of stress signal transduction in plant cells.^28^ Increasing evidence demonstrates that TCS genes are involved in the response pathway to environmental stimuli in *Arabidopsis*, rice, and soybean.^29–37^ To date, TCS genes have been identified at a genome-wide scale in various plant species, including *Arabidopsis*,^13,15^ rice,^26,38–41^ *Lotus japonicus*,^42^ soybean,^28,32^ maize,^43,44^ and *Physcomitrella patens*,^45–47^ among others. However, the knowledge of their potential functions in stress adaptations remains confined to *Arabidopsis* and rice. Studies on TCS genes in Chinese cabbage are yet to be conducted.

Chinese cabbage (*Brassica rapa* ssp. *pekinensis*) is one of the most important *B. rapa* crops and considered as an economically important vegetable worldwide because of its high yield and good quality. The whole-genome sequencing of *B. rapa* (Chiifu-401-42) by the *Brassica rapa* Genome Sequencing Project Consortium^48^ enables us to undertake a genome-wide identification and functional analysis of the gene families related to the morphological diversity and agronomic traits of *Brassica* crops.^49^ Furthermore, the ‘A’ genome of *B. rapa* is an important resource to study the evolution of polyploidy genomes and potential strategies to improve *Brassica*-related crops genetically.^50^ Given the significance of TCS genes in diverse biological and physiological processes, including our main interest area of abiotic stress responses, whole genome-wide analysis was performed to identify the HK, HP, and RR proteins involved in TCS in Chinese cabbage. The gene structures, conserved domains, and phylogenetic relationships of the TCS genes were analysed in detail. A comprehensive analysis of tissue or organ-specific expressions of TCS genes in Chinese cabbage, and their expression profiles to abiotic stresses (drought and high salinity) and exogenous phytohormones (tZ and ABA) were conducted. Moreover, the gene duplications and evolutionary patterns of the TCS genes in Chinese cabbage and *Arabidopsis* were determined. Our results provided a framework of future functional analyses of the TCS genes and lay a good foundation for the utilization of potential genes used for breeding to enhance plant production, quality, and stress-resistance in Chinese cabbage.

## 2. Materials and methods

### 2.1. Identification of TCS genes in Chinese cabbage

*Arabidopsis* TCS protein sequences were used as seed sequences to search the *Brassica* Database (BRAD) Version 1.1 (http://brassicadb.org/brad/)^48,51,52^ and the NCBI database (www.ncbi.nlm.nih.gov). The search was based on a BLASTP search with an expected value of 100. The protein sequences of the identified TCS members were used as queries to reconfirm the multiple databases to ensure that no additional related genes were missing from the database. All of the sequences that satisfied the requirements were analysed by using the Pfam database (http://pfam.janelia.org/),^53^ the SMART database (http://smart.embl-heidelberg.de/),^54^ and the Conserved Domain Database of the NCBI (http://www.ncbi.nlm.nih.gov/Structure/cdd/wrpsb.cgi)^55^ to eliminate the genes that did not contain the known conserved domains and motifs of the TCS members. The genomic information concerning on the chromosome locations of the TCS genes and the amino acid sequences of TCS proteins were obtained from BRAD. HKL members in soybean were identified and supplied as described.^28^ Sequence identities were calculated using the MatGAT software.^56^

### 2.2. Gene structure, motif recognition, multiple-sequence alignment, and phylogenetic analyses

The gene structure schematic of the TCS genes was illustrated using the Gene Structure Display Server (http://gsds.cbi.pku.edu.cn/). To identify the conserved motifs of the TCS protein sequences, we used the online MEME (http://meme.sdsc.edu/meme/meme.html).^57^ The deduced amino acid sequences of the conserved HK domain, Rec domain, Hpt domain, Myb domain, and CCT motif were aligned using the ClustalX program.^58^ For the phylogenetic relationships of *HK*, *HP*, and *RR* genes in *B. rapa* and *Arabidopsis thaliana*, the identified HK, HP, and RR protein sequences were separately aligned with a gap opening penalty of 10 and a gap extension penalty of 0.2 by using ClustalW implemented in the MEGA5.0 software (http://www.megasoftware.net/).^59^ The phylogenetic trees were further constructed using the neighbour-joining (NJ) method, in which Poisson correction, pairwise deletion, and bootstrapping (1000 replicates; random seeds) were considered as parameters.

### 2.3. Analysis of the putative promoter regions of the TCS genes in Chinese cabbage

The upstream sequences (1000 bp) of the transcriptional start site of each TCS gene were chosen as the putative promoter regions to identify the abiotic stress-related and phytohormone-related *cis*-elements. The PlantCARE website (http://bioinformatics.psb.ugent.be/webtools/plantcare/html/search_CARE.html) was used to identify the putative *cis*-regulatory elements along the promoter sequences.^60^ Besides, the recently reported stress-responsive *cis*-motifs that were not covered,^61–66^ such as ICEr1, ICEr2, NACRS, ZFHDR, MYBR, MYCR, CRT, ABRE, EE, G-box, T/G box, and CE3, were also used as queries to search against the promoter sequences. In addition, the upstream sequences (1000 bp) of the Type-A *RR* genes were used to query the PLACE database (http://www.dna.affrc.go.jp/PLACE/)^67^ and search for the three regulatory motifs: the cytokinin-responsive Type-B ARR1-binding elements AGATT^68^ and GATCTT^19,69^ and the cytokinin-enhanced protein-binding element TATTAG.^70^

### 2.4. Chromosomal localization, gene duplications, and evolutionary analysis of the TCS genes in Chinese cabbage and *Arabidopsis*

The *Brassica* Genome Browse (http://brassicadb.org/cgi-bin/gbrowse/cbgdb11/) was used to map the positions of the TCS genes in the physical maps of 10 *B. rapa* chromosomes. The TCS genes of *A. thaliana* were distributed and visualized using a chromosome map tool (http://www.arabidopsis.org/jsp/ ChromosomeMap/tool.jsp). Tandem duplications were defined whether or not the two genes are separated by four or fewer gene loci.^71^ Segmental duplications were identified by synteny analysis using an online tool (http://chibba.agtec.uga.edu/duplication/).^72^ The synteny analysis of the TCS genes was performed using PGDD (http://chibba.agtec.uga.edu/duplication/). The occurrence of duplication events and homologous genes divergence as well as selective pressure on duplicated genes were estimated by calculating synonymous (*Ks*) and non-synonymous substitutions (*Ka*) per site between the duplicated gene pairs by using the Codeml procedure of the PAML program.^73^ The divergence time was calculated at a neutral substitution rate of 1.5 × 10^–8^ substitutions per site per year for the chalcone synthase gene (*Chs*).^74^

### 2.5. Plant growth, treatments, and sampling

*Brassica rapa* subsp. *pekinensis* line Chiifu-401-42 was grown in an experimental farm in Zhejiang University. The roots, floral stems, leaves, little buds (<1.6 mm), medium-sized buds (1.6–2.8 mm), big buds (>2.8 mm), flowers, sepals, petals, stamens, pistils, little siliques, medium-sized siliques, and big siliques of the plant were sampled to analyse the tissue- or organ-specific expressions. Methods and sites of sampling were described previously.^75^ Besides, little siliques, medium-sized siliques, and big siliques were defined as siliques at 3, 14, and 25 days after pollination and fertilization.

All of the seedlings used for treatment were grown at 25 ± 1°C for ∼3 weeks with a 16-h light/8-h dark photoperiod. The nutrient solution was supplied with 200 mM NaCl for salt treatment. The total roots were separately collected at 0, 3, 8, 24, and 48 h after stress induction. Three-week-old seedlings were withheld from watering to simulate drought conditions, and the seedlings were divided into four levels (0, I, II, and III) based on the degree of the drought symptoms as previously described.^75^ The total roots were separately collected at these four levels. For mock control, seedlings were grown at normal conditions with nutrient solution and samples were collected in the meanwhile. For plant hormone treatment, 3-week-old seedlings were sprayed with the following: 100 µM *trans*-zeatin (tZ) for the cytokinin treatment and 100 µM abscisic acid (ABA) for the ABA treatment. Only the second true leaves were sampled to minimize differences. The leaves were sampled at 0, 0.5, 1, 2, and 4 h after spraying, and the control sample was sprayed with double distilled water alone. All the samples were immediately frozen in liquid nitrogen and stored in a refrigerator at −75°C.

### 2.6. RNA extraction and quantitative reverse transcription PCR analysis

RNA extraction, reverse-transcription, and quantitative reverse transcription PCR (qRT-PCR) reactions were performed as previously described^75^ using the primers listed in Supplementary Table S1. The specificity of the reactions was verified by melting curve analysis, and the products were further confirmed by agarose gel electrophoresis. The *BrCyp* gene was used as the reference gene to study different organs or tissues and developmental stages; the *BrUBC30* gene was used in abiotic stress and plant hormone treatment studies as recommended by Xiao *et al.*^76^ The comparative $\Delta \Delta C_{t}$ method was used to calculate the relative expression levels of different genes. qRT-PCR results were clustered using the average linkage method with Pearson correlation distance metric by Multiple Array Viewer.^77^

## 3. Results and discussion

### 3.1. Identification and annotation of the TCS genes in Chinese cabbage

A total of 85 TCS gene members were identified based on BLASTP search results against the BRAD and NCBI databases from the *B. rapa* genome. The DNA, CDS, protein sequences, and promoter regions of all the identified TCS members were provided in Supplementary Dataset 1. A total of 20 *Br*HK(L) proteins were divided into two groups, *Br*HKs and *Br*HKLs, according to the characteristics of their conserved domains (Supplementary Table S2). Eleven *Br*HKs contained a conserved histidine-kinase domain (HK), whereas nine *Br*HKLs comprised a diverged histidine-kinase-like domain (HKL). Seven *Br*HPs with conserved HPt domain and *Br*PHP1 with a pseudo-HPt lacking the His phosphorylation site were retrieved (Supplementary Table S3). A total of 57 RR proteins were further classified into 21 Type-A RRs, 17 Type-B RRs, 4 Type-C RRs, and 15 pseudo-RRs based on their conserved domains and motifs (Supplementary Table S4). *Br*RR22 lacked the Myb domain, but *Br*RR22 was still assigned to Type-B RRs because this gene exhibited high similarities with the proteins in this group. The full-length gene was unlikely obtained; thus, further analysis should be conducted to obtain the full-length clone and to classify this gene in the Type-B RR subfamily. Many TCS proteins in Chinese cabbage shared high sequence identities with their *Arabidopsis* counterparts, indicating the conservative evolution of TCS genes. To show a clear and systematic understanding of the number of TCS genes in plant genomes, we summarized the TCS genes that have already been identified in several plants (Table 1). Considering that *Brassica* genomes have undergone another whole-genome triplication (WGT) after speciation from *A. thaliana*, we presumed that the TCS genes in Chinese cabbage should have approximately three times as many members as that of *Arabidopsis*. However, the TCS genes identified in Chinese cabbage were only 1.55 times as many as those found in *Arabidopsis*. This result suggested that a substantial loss of genes after a hexaploid was formed by WGT. This finding is consistent with that in previous studies, in which a total of 41 174 protein-coding genes have been identified in the *B. rapa* genome; such genes were roughly 1.5 times as many as those found in *A. thaliana* (27 411 genes in TAIR10).^49^ A total of 21 and 18 *HK* genes are found in the genomes of *Glycine max* and *P. patens*; however, this finding has raised a question on the numerous *HK* genes present and the mechanism by which these *HK* genes were obtained during evolution. The origin and evolutionary pattern of the TCS signalling pathway were investigated by surveying the genomes of several sequenced key plant species ranging from unicellular algae, moss, and lycophytes to higher land plants, including *Arabidopsis* and rice, to determine the proteins involved in cytokinin signal transduction.^45^ CHASE domain-containing proteins were not detected in the genomes of any of the algal species. *Physcomitrella patens* was possibly one of the most basal plant species containing all of the components necessary for cytokinin signalling. Moreover, the number of the cytokinin receptor genes did not increase in the same manner as that of the other components. This result is also true for the cytokinin receptor genes of Chinese cabbage in our study. In contrast, Chu *et al.*^43^ identified 11 *Zm*HKs with highly conserved CHASE domains. Eight *Zm*HKs comprised three conserved domains (CHASE, transmitter, and Rec domains). Three *Zm*HKLs contained a CHASE domain, but these proteins do not comprise an integral transmitter domain or Rec domain, suggesting that the CHASE domain is highly conserved in the evolution of *Zm*HKs. This surprising anomaly increased the complexity of the evolution of CHASE domain-containing cytokinin receptor genes in plants. By comparing the numbers of Type-A *RR* and Type-B *RR* genes in various species, it is noted that there was not always more Type-A *RR* genes than Type-B *RR* genes. Until now, we only have some knowledge that Type-B *RR* genes were already found in the unicellular algae while the Type-A *RR* genes, as the youngest subgroup of *RR* genes, only first appeared in the land plant species.^45^ Since RR members were identified in only several species, more rules might be found if more *RR* genes were identified.

**Table 1.** Summary of the TCS gene numbers identified in plants

Species	HK	HPt	Type-A RR	Type-B RR	Type-C RR	Pseudo-RR	Total	References
*Arabidopsis thaliana*	8	6^a^	10	12	2	9	47	^15^
*Oryza sativa*	8	5^b^	13	13	2	8	49	^41^
*Lotus japonicus*	14	7	7	11	1	5^e^	40	^42^
*Glycine max*	21	13	18	15	3	13	83	^28^
*Zea mays*	11	9^c^	16	9	3	11^e^	59	^43,44^
*Physcomitrella patens*	18	3	7	5	2	4^e^	39	^45–47^
*Brassica rapa*	11	8^d^	21	17	4	15	76	This work

^a^Five authentic and one pseudo-HPts.

^b^Two authentic and three pseudo-HPts.

^c^Seven authentic and two pseudo-HPts.

^d^Seven authentic and one pseudo-HPts.

^e^Only clock-associated.

### 3.2. Gene structure, conserved domain, and phylogenetic analysis

**Figure 1.**
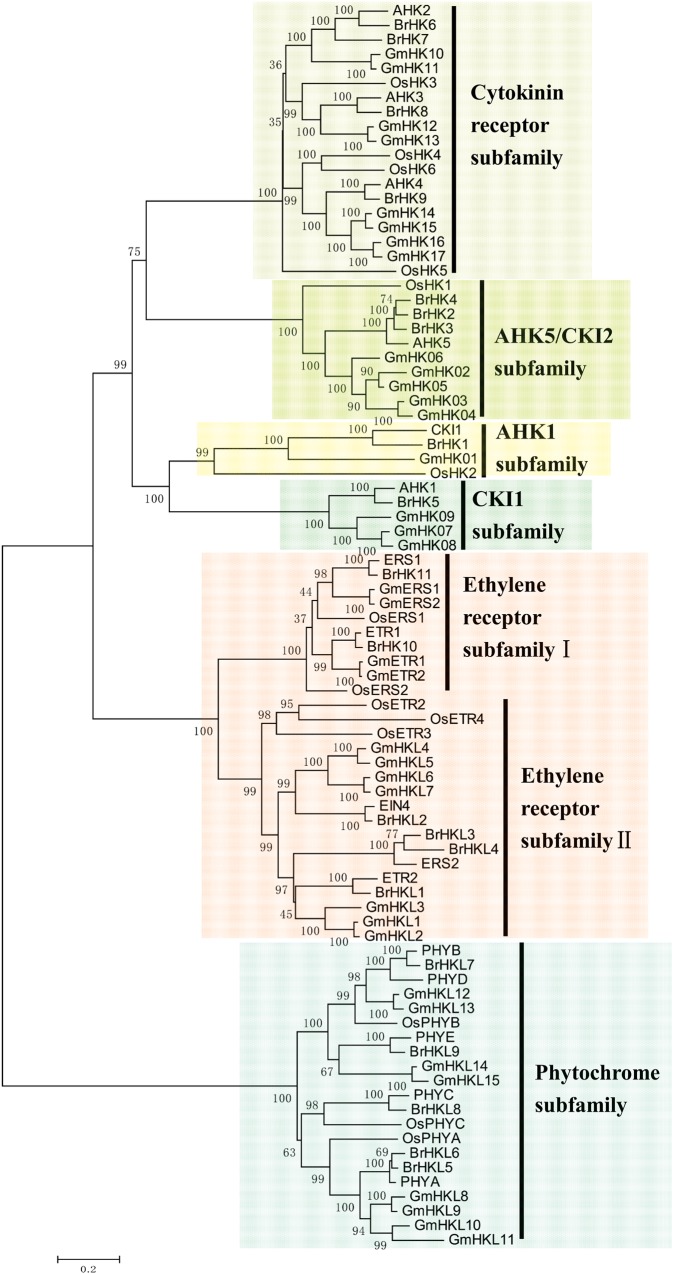
Phylogenetic relationships of HK proteins and related proteins in Chinese cabbage, *Arabidopsis*, rice, and soybean. The bar indicates the relative divergence of the sequences examined. This figure appears in colour in the online version of *DNA Research*.

**Figure 2.**
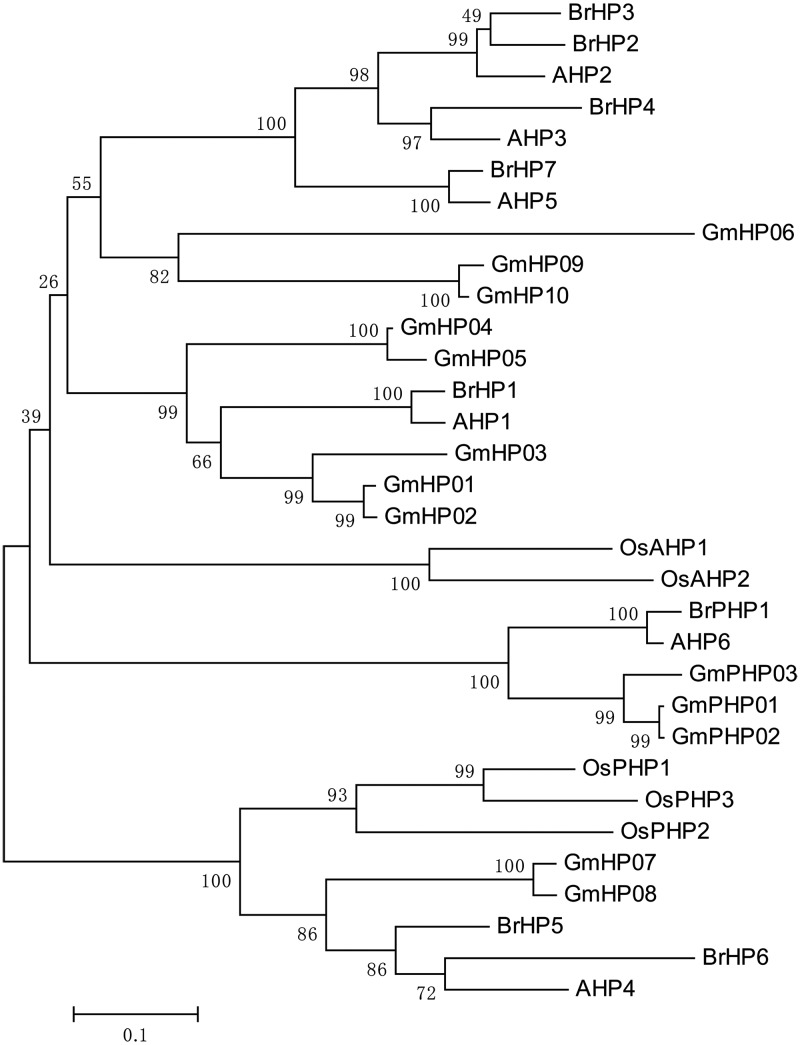
Phylogenetic relationships of HPt proteins and related proteins in Chinese cabbage, *Arabidopsis*, rice, and soybean. The bar indicates the relative divergence of the sequences examined.

**Figure 3.**
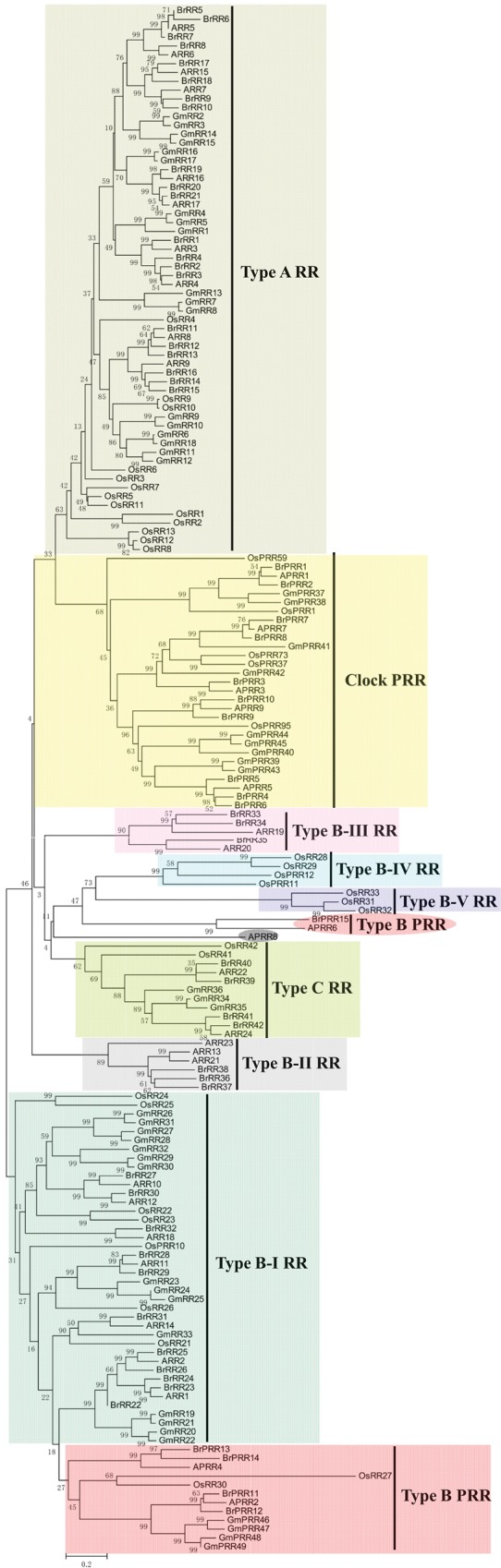
Phylogenetic relationships of RR proteins and related proteins in Chinese cabbage, *Arabidopsis*, rice, and soybean. The bar indicates the relative divergence of the sequences examined. This figure appears in colour in the online version of *DNA Research*.

To analyse the structural characteristics and conserved regions of the TCS genes, we mapped the gene structures containing exons and introns. We also examined their conserved regions and motifs; furthermore, their putative protein sequences were aligned (Supplementary Fig. S1 and Table S4). The HK, HPt, Rec, and Myb domains as well as the CCT motif of the two-component elements were well conserved. However, a group of diverged two-component elements, such as *Br*HKLs, *Br*PHP1, and pseudo-*Br*RRs, lacked the conserved phosphorylation sites. In addition, to identify subgroups and reveal the evolutionary relationships of the TCS genes in Chinese cabbage, *Arabidopsis*, rice, and soybean, the complete amino acid sequences of HK(L), HP, and RR proteins were used to perform multiple alignments and construct phylogenetic trees. Besides, 15 *Gm*HKLs were identified and supplied for a more complete phylogenetic tree (Supplementary Table S5 and Dataset 2). Figure 1 shows that HK(L) members were further divided into six subfamilies: cytokinin receptor subfamily; AHK5/CKI2 subfamily; AHK1 subfamily; CKI1 subfamily; ethylene receptor subfamily; and phytochrome subfamily. Cytokinin receptor subfamily comprised four *Br*HK proteins (*Br*HK6–9) with HK, HATPase, Rec, transmembrane (TM), and CHASE domains. The CHASE domain is specific for cytokinin receptor genes and necessary for cytokinin binding; this result indicated that *BrHK6*–*9* genes may function as cytokinin receptors. The presence of a TM domain in the cytokinin receptor genes of Chinese cabbage indicates the endomembrane location of cytokinin-binding sites.^78,79^ AHK5/CKI2 subfamily comprised three *Br*HK proteins (*Br*HK2–4) with HK as well as HATPase and Rec domains; however, this subfamily lacked a TM domain. Both AHK1 and CKI1 subfamilies contained only one *Br*HK member with HK, HATPase, Rec, and TM domains. Ethylene receptor subfamily could be further divided into ethylene receptor subfamily I and ethylene receptor subfamily II. Ethylene receptor subfamily I was composed of two *Br*HK proteins (*Br*HK10 and *Br*HK11) with TM, C2H4, GAF, and HK domains. *Br*HK11 lacked the Rec domain. Ethylene receptor subfamily II was composed of four *Br*HKL proteins (*Br*HKL1–4) with TM, C2H4, GAF, and HKL domains. Both *Br*HKL3 and *Br*HKL4 lacked the Rec domain. *Br*HKL4 contained an additional MATH (meprin and TRAF homology) domain, which belonged to the TRAF-like superfamily. Based on the gene structure and conserved domains analyses, we proposed that *BrHKL4* was formed by the fusion of two genes (Supplementary Fig. S5A). However, the actual fusion mechanism remains unknown. C_2_H_4_ is an ethylene-binding domain, suggesting its potential function in ethylene receptors. Phytochrome subfamily contained five *Br*HKL proteins (*Br*HKL5–9) with HKL, HATPase, GAF, PHY, and PAS domains. These *Br*HKLs lacked the Rec domain. PHY domain is a chromophore-binding domain. The presence of chromophore-binding domains demonstrated that they were candidate genes involved in phytochromes. The phylogentic tree constructed with all the authentic and pseudo-HPts of Chinese cabbage, *Arabidopsis*, rice, and soybean indicates the presence of subgroups (Fig. 2). *Br*HP1-4 and *Br*HP7 showed a close relationship with AHP1, AHP2, AHP3, and AHP5, which act as positive regulators in CK signalling.^80^ *Br*HP5 and *Br*HP6 were clustered into a same group with AHP4, which showed relatively distinct genetic relationships from the other AHPs and might play negative roles in CK signalling.^80^ Besides, *Br*PHP1 was close to AHP6, a pseudo-HPt that lacked the His phosphorylation site, acting inhibitory roles in CK responses by competing with other AHPs.^16^ According to the classification method proposed by Schaller and co-workers,^13,26^ RR proteins were classified as Type-A, Type-B, Type-C, and pseudo-RRs (Fig. 3). All the Type-A RRs could be well clustered into a group, which comprised 21 Chinese cabbage members (*Br*RR1–21) with one conserved Rec domain (Rec). Type-B RRs seemed to be more diverse with five subgroups (I–V). Seventeen Chinese cabbage Type-B RR members located in Type-B I RRs (*Br*RR22–32), Type-B II RRs (*Br*RR36–38), and Type-B III RRs (*Br*RR33–35). Notably, several *Os*RRs alone occupied two extra subgroups. *Os*RR28, *Os*RR29, *Os*PRR11, and *Os*PRR12 composed a single subgroup, Type-B IV and also *Os*RR31-33 composed another single subgroup, Type-B V. We supposed that this phenomenon might be due to the fact that rice, as a monocotyledon, had distant relationships with dicotyledons. Type-C RRs contained four Chinese cabbage members (*Br*RR39–42) with conserved Rec domain (Rec) alone. Pseudo-RR members were mainly divided into two groups, i.e. Clock PRR and Type-B PRR. These pseudo-RR members had relatively closer phylogenetic relationships with Type-B RR members. Ten Clock *Br*PRR members *(Br*PRR1–10) contained the conserved CCT motif and conserved pseudo-Rec domain, which lacked the conserved amino acid that is essential for phosphorylation. Five Type-B *Br*PRR members were located in two clades, in which one clade contained four Type-B *Br*PRR members (*Br*PRR11–14) with the conserved pseudo-Rec domain and Myb domain; whereas the other clade contained *Br*PRR15 with the pseudo-Rec domain alone. The gene fusion phenomenon was also observed. Besides, the pseudo-Rec and Myb domains, *Br*PRR10 also had a CDF (cation diffusion facilitator) domain. Based on the gene structure and conserved domain analyses, *BrPRR10* was also proposed to be formed by fusion of two genes (Supplementary Fig. S5B). They are not alone, in fact, we previously identified that *BrCKX1-3* was formed by the fusion of the *CKX* and *TPR* gene.^75^

On the whole, all the TCS members from Chinese cabbage, *Arabidopsis*, rice, and soybean were classified and grouped well in our phylogenetic trees, which was consistent with previous results.^26,28^ However, it is evident that TCS members from the same species were interspersed in each subgroup, especially the monocotyledonous rice, suggesting that the closer phylogenetic relationships of the species, the closer phylogenetic relationships of homologous genes. For Chinese cabbage and *Arabidopsis*, each gene of *Arabidopsis* almost corresponded to 1–3 homologous Chinese cabbage genes in each clade of the phylogenetic tree, further supporting that Chinese cabbage genome underwent another WGT after divergence from *Arabidopsis* and this event occurred after Brassicaceae diverged from Gramineae and Leguminosae.

### 3.3. Chromosomal distribution and duplications of TCS genes in Chinese cabbage and *Arabidopsis*

**Figure 4.**
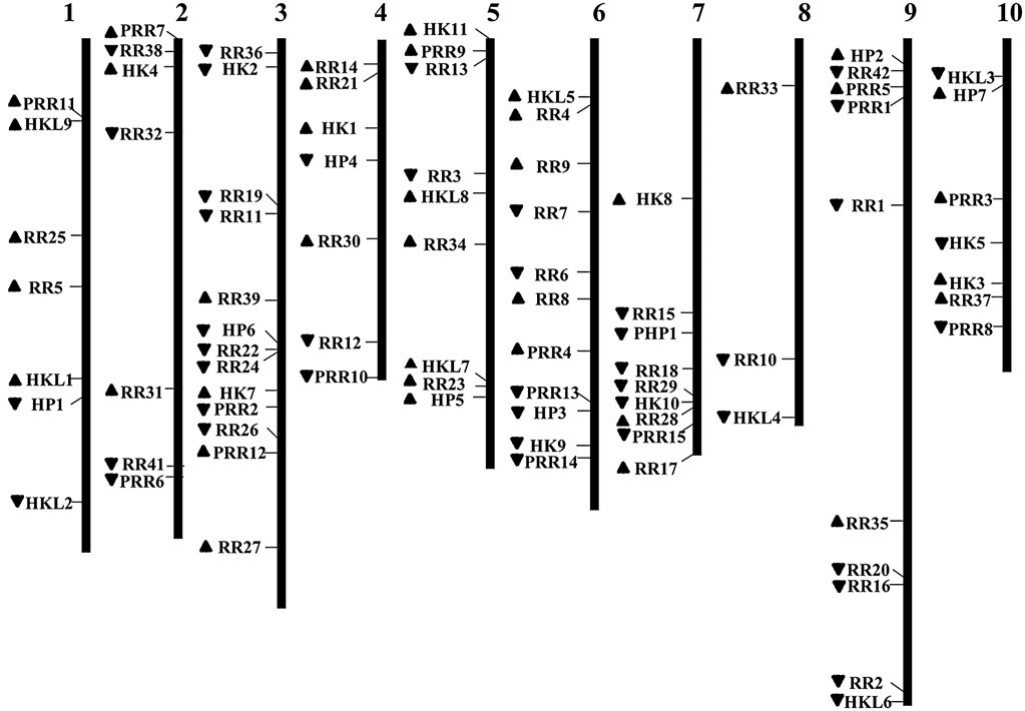
Chromosomal mapping of TCS genes in Chinese cabbage. The arrows next to gene names show the transcription direction.

Chromosomal location of each TCS gene was determined from the genomic sequences of Chinese cabbage; in addition, 83 TCS genes were mapped on the 10 chromosomes with an apparently uneven distribution (Fig. 4). The two unmapped genes, *BrHK6* and *BrRR40*, were located on Scaffold000104 and Scaffold000191, respectively. Gene duplication events are important to gene family evolution because duplicated genes provide the raw materials for the generation of new genes, which in turn facilitate the generation of new functions.^81^ Brassicaceae genomes have undergone three rounds of whole-genome duplication; these genomes are referred to as 1R, 2R, and 3R, which are equivalent to the γ, β, and α duplication events; furthermore, *Brassica* genomes have undergone another WGT (4R) after speciation from *A. thaliana*,^82–84^ which led to significantly increased duplicated gene numbers in *B. rapa*. In plants, gene numbers are mainly expanded by segmental and tandem duplications in gene families.^85^ We examined tandem duplications and segmental duplications of TCS genes in Chinese cabbage. As shown in Table 2, multiple TCS genes were involved in segmental duplications, whereas only one tandem duplicated gene pair was identified; therefore, the expansion of TCS genes in Chinese cabbage was mainly attributed to segmental duplications. Compared with duplicated TCS genes in Chinese cabbage, TCS genes in *A. thaliana* were also involved in segmental duplications, but with smaller percentage. However, all the 10 Type-A *ARR* genes and half of the Type-B *ARR* genes were strikingly segmentally duplicated.

**Table 2.** Comparison of duplicated TCS genes in Chinese cabbage and *Arabidopsis*

Gene family	Number of genes involved in segmental duplications	Percentage (%)	Number of genes involved in tandem duplications	Percentage (%)
*AHK(L)*/*BrHK(L)*	2/8	12.5/40	0/0	0/0
*AHP*/*BrHP*	2/5	33.3/62.5	0/0	0/0
Type-A *ARR*/*BrRR*	10/21	100/100	0/0	0/0
Type-B *ARR*/*BrRR*	6/11	50/64.7	0/2	0/11.8
Type-C *ARR*/*BrRR*	0/4	0/100	0/0	0/0
Pseudo-*ARR*/*BrRR*	0/12	0/80	0/0	0/0

**Figure 5.**
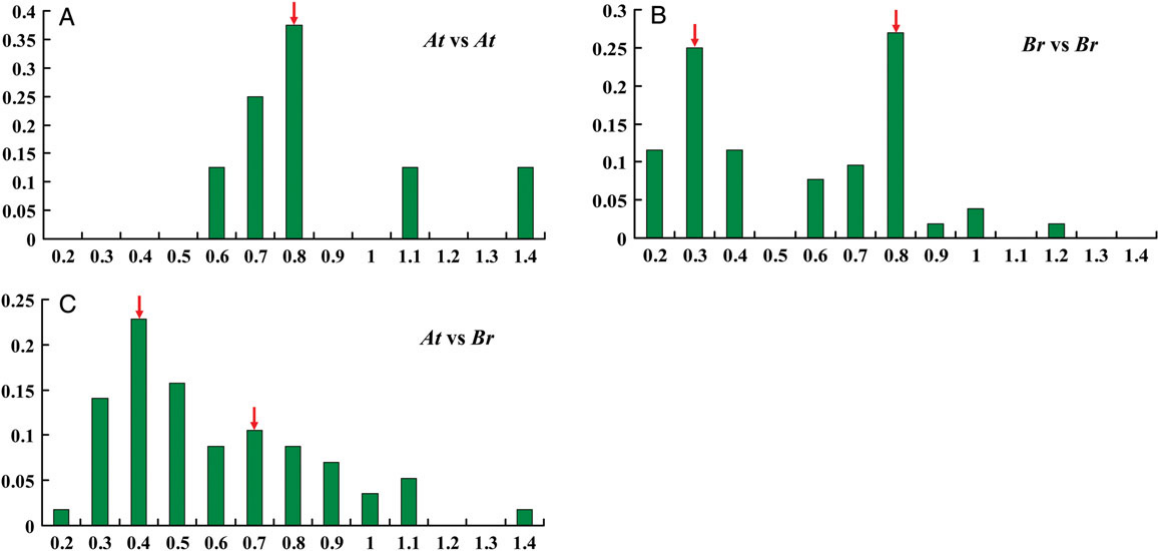
The *Ks* values distribution of the TCS genes in the genome of *A. thaliana (At*) and *B. rapa* (*Br*) viewed through the frequency distribution of *Ks* modes. *Ks* value distributions were obtained from duplicated paralogous genes pairs in the *At* genome (A) and *Br* genome (B) and duplicated orthologous gene pairs between the *Br* and *At* genomes (C). The vertical axis indicates the frequency of paired sequences, whereas the horizontal axis denotes the *Ks* values with a 0.1 interval. This figure appears in colour in the online version of *DNA Research*.

The synonymous substitution rates (*Ks*) and non-synonymous substitution rates (*Ka*) are measured to explore the gene divergence mechanism after duplication. Large-scale duplication events are defined as simultaneous gene duplication. Assuming a molecular clock, the *Ks* of these duplicates are expected to be similar over time. However, substantial rate variations are noted among genes.^86^ To elucidate the evolutionary patterns and divergence of the segmental duplicated TCS genes in Chinese cabbage and *Arabidopsis,* the *Ks* and *Ka* modes for the segmental duplicated paralogs and orthologs were determined (Supplementary Table S6). We used the relative *Ks* measure as the substitute for time to evaluate the divergence time between Chinese cabbage and *Arabidopsis.* The frequency distributions of the relative *Ks* values that were obtained from duplicated orthologous gene pairs, between the Chinese cabbage and *Arabidopsis* genomes and duplicated paralogous gene pairs in the Chinese cabbage and *Arabidopsis* genomes, are shown in Fig. 5. The relative *Ks* distribution of the duplicated paralogous gene pairs for TCS genes in *A. thaliana* peaked from 0.8 to 0.9, which indicated that these duplicated genes occurred at ∼53 to 60 Mya, which corresponds to the 3R time.^87,88^ The relative *Ks* distribution of the duplicated paralogous gene pairs for TCS genes in Chinese cabbage displayed peaks from 0.3 to 0.4 and from 0.8 to 0.9, which suggests an additional duplication event occurred at ∼20 to 26 Mya after 3R, which corresponds to the 4R time.^88^ Our results further confirmed the hypothesis that *Brassica* genomes have undergone another WGT after speciation from *A. thaliana.* The existence of gene duplications in Chinese cabbage and *Arabidopsis* that occurred during 3R also demonstrated their conservation during the long-term evolution. For the duplicated orthologous gene pairs between Chinese cabbage and *Arabidopsis*, the relative *Ks* distribution showed a major peak from 0.4 to 0.5, which suggests that the two genomes were separated at ∼27 to 33 Mya; this finding was in agreement with previous studies that *Brassica* and *Arabidopsis* genomes diverged after the 3R event.^87–91^ In addition, another less obvious peak appeared from 0.7 to 0.8, which was responsible for the 3R event before the speciation between *Brassica* and *Arabidopsis* genomes.

### 3.4. Analysis of the putative promoter regions of TCS genes in Chinese cabbage

*cis*-Regulatory elements, which are located upstream of genes and act as binding sites for TFs, have essential functions in determining the tissue-specific or stress-responsive expression patterns of genes.^92^ To further understand transcriptional regulation and potential functions of these TCS genes, 1000 bp regions upstream of the transcriptional start site were applied to identify *cis*-regulatory elements. A number of abiotic stress-related (e.g. drought, high salinity, extreme temperatures, and wound) and hormone-related (e.g. ABA, auxin, ethylene, GA, MeJA, and SA) *cis*-elements were found in the putative promoters of TCS genes in Chinese cabbage. These *cis*-elements are counted and classified in Supplementary Table S7. The occurrences of these *cis*-elements suggested that these TCS genes might have potential functions in abiotic stress adaptations and various hormone signalling.

GARP is a Type-B signature; and the binding sites of GARP are present in the Type-A *ARR* gene promoters, which suggest that Type-B ARR proteins may bind to these promoters and activate transcription.^19^ Previous research also confirmed that cytokinin-dependent induction of the Type-A *RR* genes is partially dependent on transcriptional regulation by Type-B *RR* genes.^68,93^ We investigated the cytokinin-related binding elements in the Type-A *BrRR* promoter regions. Cytokinin-responsive Type-B ARR1-binding elements AGATT and GATCTT as well as cytokinin-enhanced protein-binding element TATTAG were widely evident (Supplementary Table S8); this finding indicated that Type-A *BrRR* genes might be transcriptionally regulated by Type-B *Br*RR proteins and cytokinin. Two ARR1-binding elements were found in some Type-A *BrRR* gene promoters; thus, we speculated that the TCS is conserved between Chinese cabbage and *Arabidopsis* at the level of system regulation, as well as gene structure.

### 3.5. Expression profiles of TCS genes in various tissues, organs, and developmental stages

**Figure 6.**
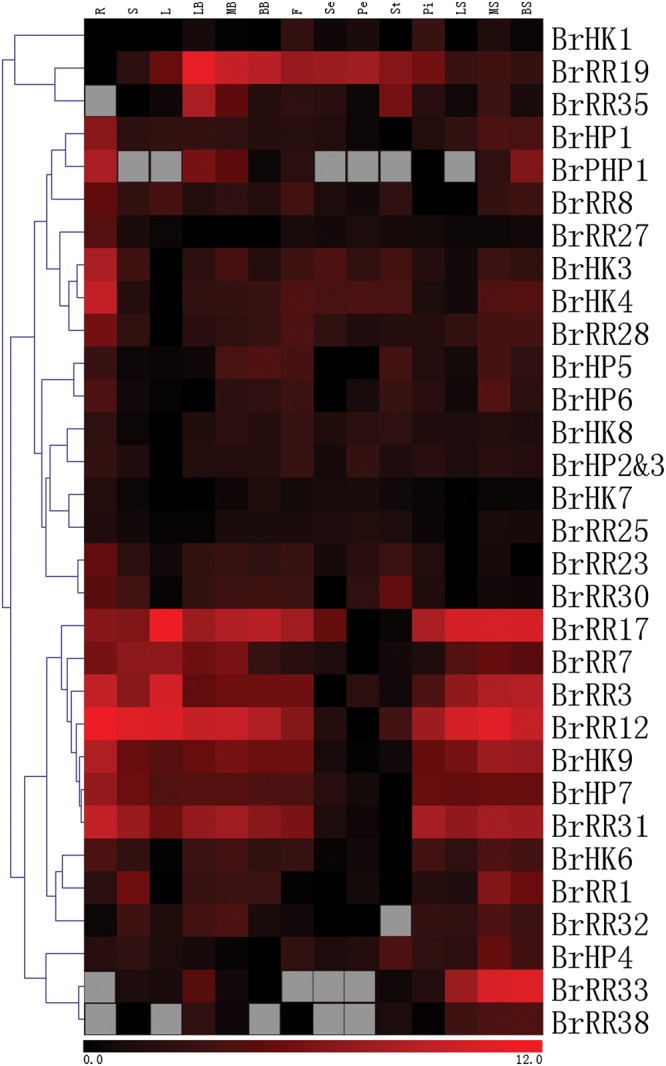
Hierarchical clustering and heat map representation for the tissue- or organ-specific TCS gene expression profiles in Chinese cabbage. R, roots, S, floral stems, L, leaves, LB, little buds, MB, medium-buds, BB, big buds, F, flowers, Se, sepals, Pe, petals, St, stamens, Pi, pistils, LS, little siliques, MS, medium-sized siliques, and BS, big siliques. The expression levels of genes are presented using fold-change values transformed to Log_2_ format. This figure appears in colour in the online version of *DNA Research*.

Tissue- or organ-specific and developmental stage-related expression data are useful in identifying genes that are involved in defining the precise nature of individual tissues in a given developmental stage.^92^ A clear expression profile of genes of interest is an indispensable step to find and utilize agriculturally important genes. To obtain first insight into the functions of TCS genes during the Chinese cabbage vegetative and reproductive development, qRT-PCR was used to analyse the transcription levels of TCS genes in various tissues, organs, and developmental stages. Totally, only 31 of the 61 TCS genes that could be distinguished by qRT-PCR were selected for expression analysis because of the high similarities of the TCS sequences, and the remaining 9 *Br*HKL genes and 15 *Br*PRR genes were not within the scope of our study. Based on Fig. 6 and Supplementary Fig. S6, data analyses indicated high variability in transcript abundance of the TCS genes in Chinese cabbage. A majority of TCS genes showed relatively high expression levels in roots, such as *BrHK3*, *BrHK4*, *BrHP1*, *BrPHP1*, *BrRR3*, *BrRR12*, and *BrRR31*. This finding might be correlated with the fact that cytokinins are mainly synthesized in roots. Notably, *B*r*RR17*, *BrRR7*, *BrRR3*, *BrRR12*, *BrHK9*, *BrHP7*, and *BrRR31* exhibited similar expression patterns and were grouped together. These genes were expressed abundantly in vegetative organs, floral buds, and developing siliques; however, these genes showed low transcripts in sepals, petals, and stamens; these findings indicated that they have spatio-temporal expression characters. *BrRR19* had a relatively higher expression level in floral buds and four whirl flower organs; this finding suggests its function in flower development. Genes with high transcripts in developing siliques may have essential functions in seed quality and yield. We found that *BrRR33* and *BrRR38* were almost expressed exclusively in developing siliques, which demonstrates that they were candidate genes in improving seed quality and yield. To explore the expression patterns of the homologous genes between Chinese cabbage and *Arabidopsis*, gene expression data for *Arabidopsis* TCS genes were retrieved from the Genevestigator database (data not shown) and then we compared these data with our results. Most of the homologous genes showed similar expression patterns, suggesting the functional conservation of homologs. Comparisons of the tissue-specific expression profiles between Chinese cabbage and *Arabidopsis* TCS genes might help in determining the unknown functions of the TCS genes in Chinese cabbage. In *Arabidopsis*, *AtCKI1* is mainly expressed in flowers and participated in female gametophyte development.^94–96^ Similarly, *BrHK1*, which is a *AtCKI1* homologous gene, showed major transcriptions in flowers and pistils in Chinese cabbage. Similar expression profiles may suggest similar function for *Arabidopsis* orthologous TCS genes in Chinese cabbage. Based on the high sequence identity and similar expression patterns, we proposed that *BrHK1* was also related to female gametophyte development in Chinese cabbage. Consistently, with a loss of function mutant line, Ito and Kurata^38^ revealed that *OsHK1*, which is a *AtCKI1* homologous gene in rice, is essential for rice female gametophyte development. This result further strengthened our hypothesis and proved the conservation function of *CKI1*. Furthermore, *AtAHK5*/*CKI2* is mainly expressed in roots^97^ and regulated salt sensitivity and resistance against bacterial and fungal infection^98^; in addition, *AtAHK5*/*CKI2* acted to integrate multiple signals via H_2_O_2_ homeostasis and is independent of ABA signalling in guard cells.^99^ Two homologous genes, *BrHK3* and *BrHK4*, were analysed in Chinese cabbage, and these genes also showed major transcriptions in roots. Thus, *BrHK3* and *BrHK4* were probably involved in stress adaptations in Chinese cabbage.

### 3.6. Expression profiles of TCS genes under drought and salt stresses in Chinese cabbage roots

**Figure 7.**
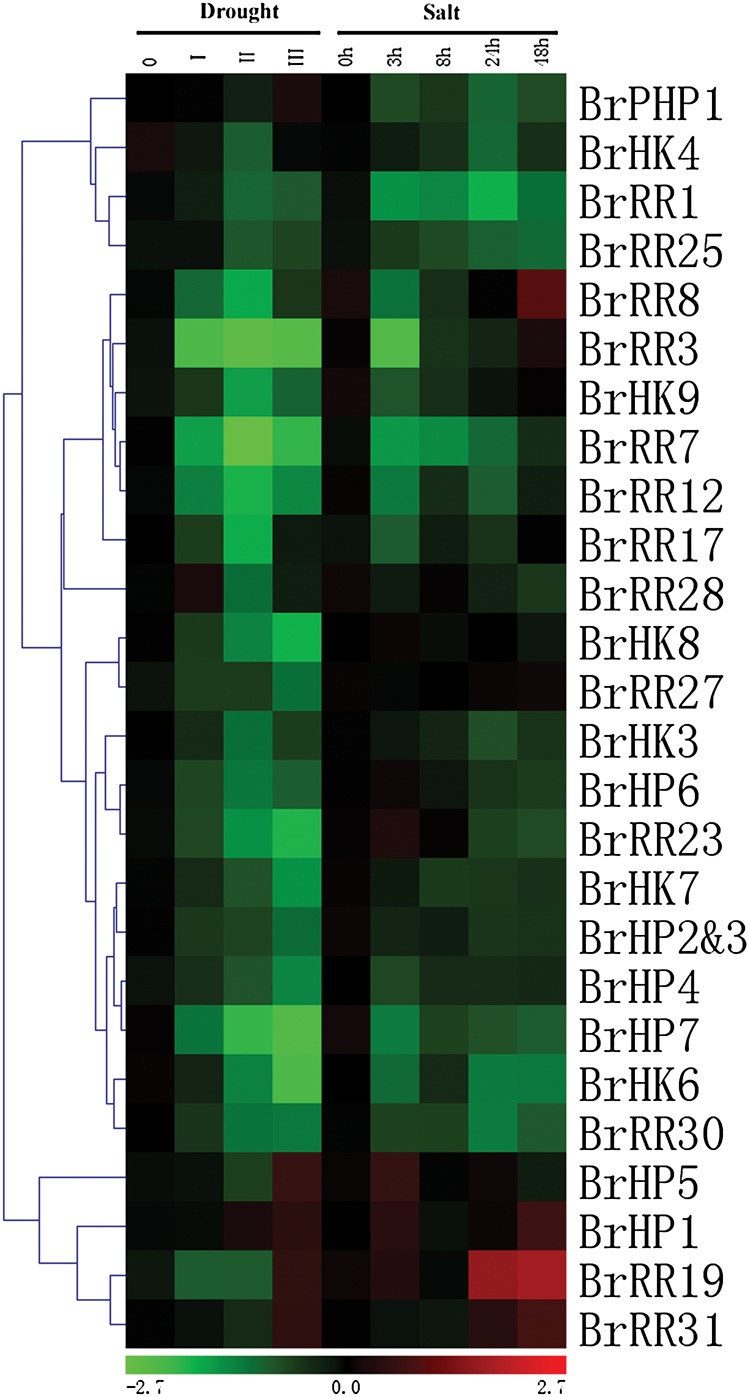
Hierarchical clustering and heat map representation for the response patterns to drought and high salinity conditions of TCS genes in Chinese cabbage roots. The expression levels of genes are presented using fold-change values transformed to Log_2_ format compared with control. This figure appears in colour in the online version of *DNA Research*.

In addition to cytokinin signalling, accumulating evidence proves that TCS is also involved in plant adaptation to various environmental stresses. Relationships between cytokinin signalling and stress responses were summarized in several reviews.^29,100–102^ Roots were generally believed to be the main organs involved in drought and high salinity stresses, thus 26 of the 31 examined TCS genes that showed relatively high expression levels in Chinese cabbage roots were selected; in addition, qRT-PCR was used to analyse their expression profiles under drought and salt stress conditions. As shown in Fig. 7 and Supplementary Figs S7 and S8, most of the TCS genes, as a whole, were suppressed in drought conditions. In different degrees of drought symptoms, expressions of *BrHK6*, *BrHK7, BrHK8*, *BrHP2–3*, *BrHP4*, *BrHP7*, *BrRR1*, *BrRR3*, *BrRR7*, *BrRR23*, *BrRR27*, and *BrRR30* were continually suppressed and decreased to ∼15% of the control level. Transcripts of *BrHK3*, *BrHK9*, *BrHP6*, and *BrRR8* were also primarily suppressed, which subsequently increased again after a fall in level Ⅱ; however, the transcriptions of these genes were still below the basal level in level III, which indicates that they were potential negative regulators in drought stress response. *BrHP5*, *BrHP1*, *BrRR19*, and *BrRR31* were clustered into one group, and these genes showed upregulated expressions in levels II or III. *BrHP1* was consistently induced; this finding suggests the positive roles of *BrHP1* in drought stress. For the salt treatment, most of the TCS genes also showed decreased expression levels; however, the responses of these genes were not always the same with drought treatment. *BrHK8* and *BrRR27* seemed to be unaffected with salt treatment; however, these genes were continually suppressed in drought conditions. The transcript of *BrPHP1*, which is a pseudo-HP gene, was unaffected by drought treatment; *BrPHP1* transcript was suppressed in high salinity conditions. *BrRR8* was mainly induced with 2-fold upregulated expression levels after 48 h salt treatment; however, this gene was suppressed in drought treatment. Likewise, *BrHP5*, *BrHP1*, *BrRR19*, and *BrRR31* were clustered together with increased expressions, suggesting their positive functions under high salinity conditions. *BrRR19* and *BrRR31* transcripts were stable 8 h after salt treatment; however, these genes were continually induced after 8 h and showed 3.4- and 2.0-fold increased expression levels at 48 h. Theoretically, almost all the TCS genes in Chinese cabbage could be induced by drought and salt stresses because of the presence of stress-inducible *cis*-regulatory elements in their promoter regions. However, our results conflicted with our hypothesis with most of the genes being supressed. Although *cis*-element can be used to predict stress-responsive genes via a *cis*-element-based targeted gene-finding approach;^28^ however, the frequency of a *cis*-element sequence in the whole genome is relatively high because of their short length (5–9 bp core) and flexibility. In addition, a number of *cis*-element sequences might be syntactically correct without providing practical regulatory function. Moreover, there are also stress-repressive genes among the TCS genes and we have limited knowledge on the stress-repressive *cis*-element.^103^ In fact, the patterns of responses to abiotic stress stimuli of the TCS genes were complicated and varied in terms of the species, genotypes, and organs or tissues. In *Arabidopsis*, drought significantly induced the expression of a Type-A *ARR* gene subset, *ARR5*, *ARR7*, and *ARR15*,^104^ whereas almost all genes for Type-A *RR* genes were suppressed by drought stress in rice.^35^ Moreover, all of the members of the TCS family had differential transcript abundance (under both non-stress and salinity stress conditions) among salt-tolerant (Pokkali) and salt-sensitive (IR64) rice genotypes.^37^ Furthermore, Argueso *et al.*^101^ collected data from BIO Array Resource and illustrated that TCS gene responsiveness to different abiotic stresses in *Arabidopsis* root and shoot tissues are different. Le *et al.*^32^ examined and compared the TCS gene expression profiles in root and shoot tissues of soybean plants under dehydration stress; in addition, Le *et al.* found that the majority of soybean TCS genes respond to dehydration stress in either root- or shoot-specific manner. This is also the case for the expression patterns of *GmARFs* in drought stress.^105^ So *cis*-element-based predicted genes should be validated experimentally to reveal their authentic expression profiles in specific tissues or organs upon various abiotic stress conditions. One of our interests is that whether genes located in the same clade of the plylogenetic tree would show similar response patterns to abotic stresses. Thus, we carefully examined and compared the expression patterns of TCS genes in *Arabidopsis*, rice, and soybean with our results. It was amazing that even the homologous TCS genes showed varied response patterns, suggesting the stress-responsive functional divergence of the homologous genes. In *Arabidopsis*, *AHK2*, *AHK3*, and *CRE1* transcripts were all rapidly induced by dehydration; *AHK2* expression also appeared to be influenced by NaCl and ABA treatments; and induction of the *AHK3* mRNA was observed during high salinity and cold stresses, whereas expressions of *CKI1* and *CKI2* were not induced by any treatment.^29^ However, in rice, the HK genes (*HK5* and *HK3*) that are homologous to CK receptor genes (*AHK2*, *AHK3*, and *CRE1*) in *Arabidopsis* were induced by drought stress, while *HK6* homologous to *CKI2* was repressed when exposed to drought stress.^35^ Likewise, the soybean HK genes (*GmHK10*–*17*) that are homologous to *Arabidopsis* CK receptor genes (*AHK2*, *AHK3*, and *CRE1*) showed more diverse response patterns, for example, *GmHK11* was repressed while *GmHK12* was induced under dehydration stress.^28^ Furthermore, response patterns unlikely correlated well with their biological functions under abiotic stress conditions. We have mentioned that transcripts of *AHK2*, *AHK3*, and *CRE1* in *Arabidopsis* could be induced by abiotic stresses;^29^ however, loss-of-function analysis of *ahk2*, *ahk3*, and *cre1* indicated that the stress-responsive *AHK2*, *AHK3*, and *CRE1* acted as negative regulators in ABA signalling; in addition, *AHK2* and *AHK3* negatively control osmotic stress responses in *Arabidopsis*.^29^ Other supporting evidence was that *AHK2* and *AHK3* have negative regulatory functions in cold stress signalling, via ABA response inhibition; however, the transcripts of these genes were not altered by cold condition.^36^ The information regarding TCS gene identification would be more valuable if the functional predication is verified. For the TCS genes in Chinese cabbage, expression profiling is an initial step; further elucidating the potential functions of these genes involved in abiotic stress adaptations would be an interesting and meaningful study.

### 3.7. Effects of exogenous tZ and ABA on TCS gene expressions in Chinese cabbage leaves

**Figure 8.**
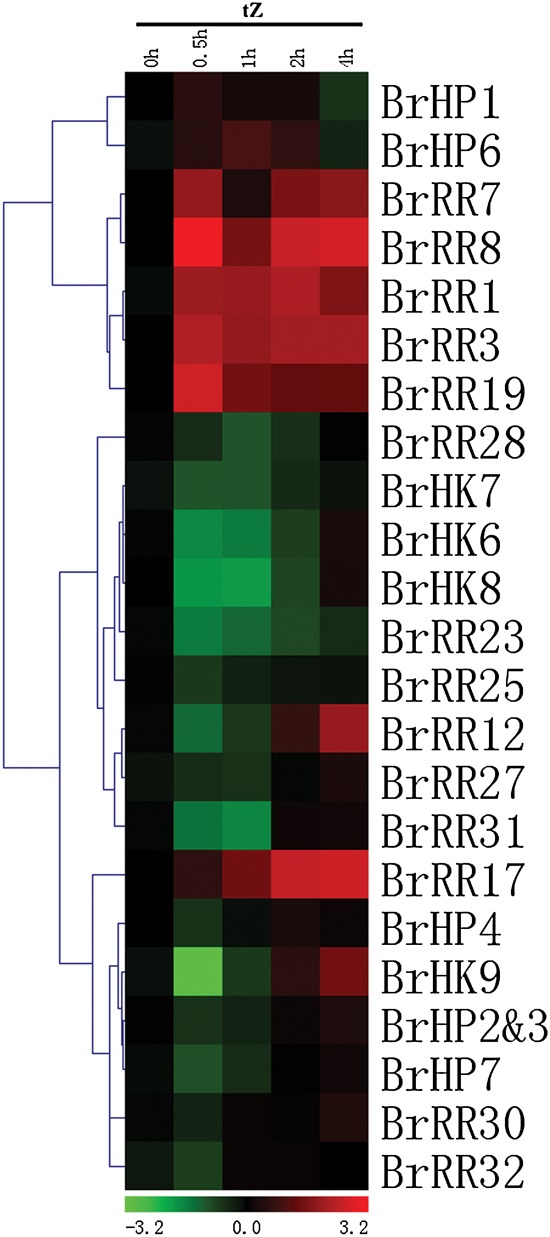
Hierarchical clustering and heat map representation for the response patterns to exogenous tZ of TCS genes in Chinese cabbage leaves. The expression levels of genes are presented using fold-change values transformed to Log_2_ format compared with control. This figure appears in colour in the online version of *DNA Research*.

**Figure 9.**
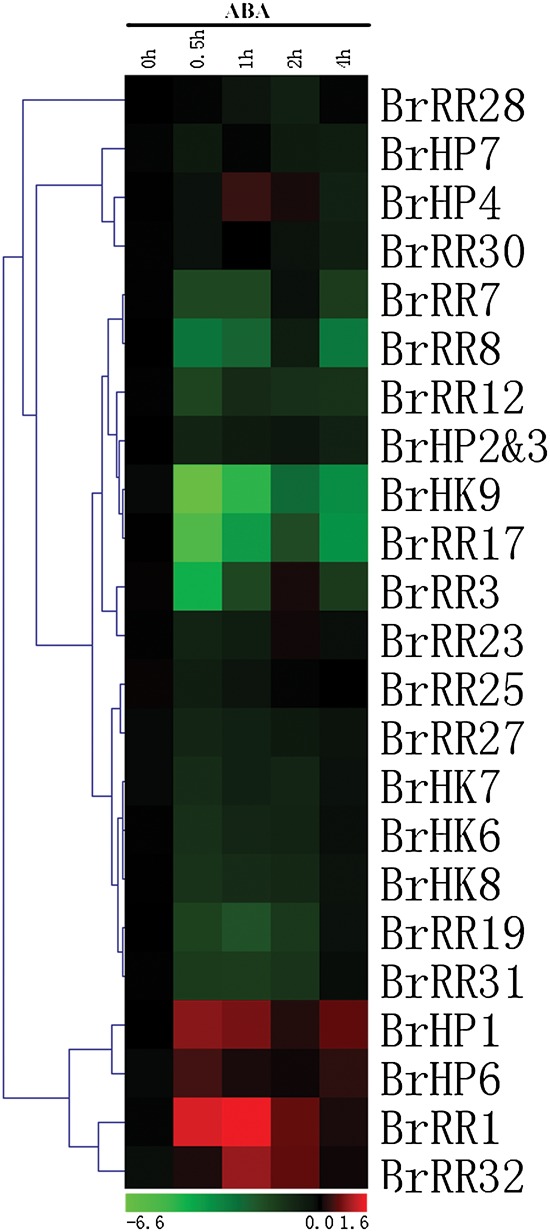
Hierarchical clustering and heat map representation for the response patterns to exogenous ABA of TCS genes in Chinese cabbage leaves. The expression levels of genes are presented using fold-change values transformed to Log_2_ format compared with the control. This figure appears in colour in the online version of *DNA Research*.

The response patterns of TCS genes to exogenous cytokinin levels were carefully examined in *Arabidopsis*. Previous data based on Northern blot, microarray, and qRT-PCR analysis indicated that *AHK*, *AHP*, and Type-B *ARR* genes were not regulated by exogenous tZ and BA except for the upregulated *AHK1* and *AHK4*; Type-A *ARR* genes, which function as the cytokinin primary response genes, are strongly induced upon treatment with cytokinins.^106–109^ To determine the effects of exogenous cytokinin on TCS gene expressions, we investigated the expression profiles of 23 of the 31 examined TCS genes with relatively high expression levels in Chinese cabbage leaves by qRT-PCR analyses (Fig. 8 and Supplementary Fig. S9). Spraying is a simple and easy method for treatment and also leaves response well to exogenous phytohormones. Our results revealed that the selected seven Type-A *RR* genes were transcriptionally upregulated by tZ. The inductions of these genes occurred in 30 min; the transcripts of such genes remained at high levels at 1, 2, and 4 h. Many *BrHK*, *BrHP*, and Type-B *BrRR* genes were primarily suppressed; afterward, their transcripts increased again and remained at the basal levels at 2 and 4 h. Considering that Type-A *ARR* genes negatively regulated *HP* and Type-B *ARR* gene transcriptions in *Arabidopsis*,^93,110^ we supposed that the instantly upregulated Type-A *BrRR* genes suppressed the *BrHP* and Type-B *BrRR* gene transcriptions; therefore, these events led to a negative feedback loop, thereby suppressing the expressions of putative cytokinin receptors (*BrHK6*–*9*) in Chinese cabbage; furthermore, autoregulations of these genes ensured transcript abundance as time progresses. ABA is the key hormone involved in the regulation of both stress- and non-stress-related processes.^111,112^ Furthermore, cytokinins are generally postulated to function as antagonists of ABA in various growth and physiological processes, including environmental stress responses. Studies have also suggested the presence of intensive interactions and crosstalk between cytokinins and ABA as well as their signalling pathways.^100^ Thus, we also examined the effects of exogenous ABA on TCS gene expressions in Chinese cabbage. Figure 9 and Supplementary Fig. S10 showed that many TCS genes were suppressed by ABA treatment; this finding was consistent with the response patterns of drought and salt treatment. *BrHK9*, *BrRR3*, and *BrRR17* were strongly and instantly suppressed, and even dropped to undetectable levels after 30 min. *BrHP4*, *BrHP7*, *BrRR28*, and *BrRR30* transcripts were relatively stable with ABA treatment. *BrHP1*, *BrHP6*, *BrRR1*, and *BrRR32* were clustered together with increased expression levels upon ABA treatment. *BrHP1* was also induced by drought and salt treatment; this result further confirms the positive regulations of *BrHP1* in stress environments. However, similar to the case with drought and salt treatment, promoter regions of Chinese cabbage TCS genes were rich in *cis*-element involved in ABA response, such as MYBR, MYCR, and ABRE etc., many of the TCS genes were contrarily suppressed by ABA treatment, and these could be mainly attributed to the complex interactions of hormone network in addition to the own limitations of *cis*-element-based predicting approach.

### 3.8. Conclusions

In summary, a total of 20 HK(L), 8 HP, and 57 RR proteins were identified and annotated in the Chinese cabbage genome. This study provided the first insight into the TCS members of Chinese cabbage. The analysis results of duplications, evolutionary patterns, and divergence of TCS genes gained useful information on the evolutionary aspects of Chinese cabbage genome. We also focused on the response patterns of the TCS genes to drought and high salinity conditions and screened numerous candidate stress-responsive genes in Chinese cabbage. TCS has an important function in signal transduction involved in plant growth, development, and environmental stimuli. Moreover, Chinese cabbage is one of the most important vegetables that are widely cultivated. Our results contributed relevant information to molecular genetic studies, thereby providing a better understanding of the biological functions of the TCS genes in Chinese cabbage. Our study may also provide guidance for molecular breeders to develop economically important high-yielding and high-quality stress-tolerant crops in agriculture.

## Supplementary data

Supplementary data are available at www.dnaresearch.oxfordjournals.org.

## Funding

This research was partially supported by the Zhejiang Provincial Natural Science Foundation of China (grant no. Y13C150001), the Hi-Tech Research and Development Program of China (grant no. 2012AA100104-4), the Breeding Project of the Science–Technology Foundation of Zhejiang Province (grant no. 2012C12903), and the Key Science and Technology Program of Zhejiang Province (grant no. 2010C12004).

## Supplementary Material

Supplementary Data
