# Supplementary material for: Genome-Wide Identification, Phylogeny, Duplication, and Expression Analyses of Two-Component System Genes in Chinese Cabbage (Brassica rapa ssp. pekinensis)
Source: DNA Res. 2014 Feb 27;21(4):379–96. doi: 10.1093/dnares/dsu004 (PMC4131832; doi:10.1093/dnares/dsu004)
Supplement: Supplementary Data [file supp_21_4_379__index.html]

Genome-Wide Identification, Phylogeny, Duplication, and Expression Analyses of Two-Component System Genes in Chinese Cabbage (Brassica rapa ssp. pekinensis) — Supplementary Data 

# Genome-Wide Identification, Phylogeny, Duplication, and Expression Analyses of Two-Component System Genes in Chinese Cabbage (*Brassica rapa* ssp. *pekinensis*)

## Supplementary Data

Supplementary Data

**Files in this Data Supplement:**

- Supplementary Data - Docx file
- Supplementary Data - Supplementary Data
- Supplementary Data - Supplementary Data
- Supplementary Figure 1 - jpg file
- Supplementary Figure 2 - jpg file
- Supplementary Figure 3 - jpg file
- Supplementary Figure 4 - jpg file
- Supplementary Figure 5 - jpg file
- Supplementary Figure 6 - jpg file
- Supplementary Figure 7 - jpg file
- Supplementary Figure 8 - jpg file
- Supplementary Figure 9 - jpg file
- Supplementary Figure 10 - jpg file
- Supplementary Table 1 - doc file
- Supplementary Table 2 - doc file
- Supplementary Table 3 - doc file
- Supplementary Table 4 - doc file
- Supplementary Table 5 - doc file
