# Supplementary material for: Genome-Wide Identification, Phylogeny, Duplication, and Expression Analyses of Two-Component System Genes in Chinese Cabbage (Brassica rapa ssp. pekinensis)
Source: DNA Res. 2014 Feb 27;21(4):379–96. doi: 10.1093/dnares/dsu004 (PMC4131832; doi:10.1093/dnares/dsu004)
Supplement: Supplementary Data [file supp_dsu004_dsu004supp.docx]

**Supplementary data**

**Supplementary Table S1.** Forward and reverse primers used in the qRT-PCR gene expression studies.

**Supplementary Table S2.** HK(L) proteins in Chinese cabbage.

**Supplementary Table S3.** HP proteins in Chinese cabbage.

**Supplementary Table S4.** RR proteins in Chinese cabbage.

**Supplementary Table S5.** HKL proteins in soybean.

**Supplementary Table S6.** Summary of the duplicated gene pairs and determination of the *Ka* and *Ks* values of the TCS genes in Chinese cabbage and *Arabidopsis*.

**Supplementary Table S7.** Summmary of abiotic-stress related and hormone related *cis*-elements in the putative promoter regions of TCS genes in Chinese cabbage.

**Supplementary Table S8.** Distribution of cytokinin-related binding elements in the putative promoter regions of type-A *BrRRs*.

**Supplementary Figure S1.** Diagram representations of the gene structures and primary domain structures of TCS genes in Chinese cabbage.

**Supplementary Figure S2.** Alignment of deduced amino acid sequences of His-protein kinase transmitter (A) and receiver (B) domains of His-protein kinases and related proteins in Chinese cabbage.

**Supplementary Figure S3.** Alignment of deduced amino acid sequences of His-phosphotransfer proteins in Chinese cabbage.

**Supplementary Figure S4.** Alignment of deduced amino acid sequences of receiver domain (A), Myb domain (B), and CCT motif (C) of response regulators and response regulator-like proteins in Chinese cabbage..

**Supplementary Figure S5.** Gene structure and conserved domain analysis of *BrHKL4* (A) and *BrPRR10* (B).

**Supplementary Figure S6.** Relative expression profiles of TCS genes in various Chinese cabbage tissues and organs..

**Supplementary Figure S7.** Expression of TCS genes in Chinese cabbage roots under drought stress.

**Supplementary Figure S8.** Expression of TCS genes in Chinese cabbage roots under salt stress..

**Supplementary Figure S9.** Expression of TCS genes in Chinese cabbage leaves with tZ treatment..

**Supplementary Figure S10.** Expression of TCS genes in Chinese cabbage leaves with ABA treatment..

**Supplementary Dataset 1.** DNA, CDS, protein sequences and promoter regions of TCS members in Chinese cabbage.

**Supplementary Dataset 2.** Protein sequences of *Gm*HKLs.
