# Supplementary material for: Genome-Wide Identification, Phylogeny, Duplication, and Expression Analyses of Two-Component System Genes in Chinese Cabbage (Brassica rapa ssp. pekinensis)
Source: DNA Res. 2014 Feb 27;21(4):379–96. doi: 10.1093/dnares/dsu004 (PMC4131832; doi:10.1093/dnares/dsu004)
Supplement: Supplementary Data [file supp_dsu004_dsu004supp1.doc]

**DNA and CDS sequences**

>BrHK1(Bra032761)-DNA

ATGGTGAATGTTAGAAAGTTTGTGACTTCTCGTCCAATATTTGTCTTCTTCCTCTTGGTAATATTTTCTTACTTGTTAATTCTTTTTATAATCGTTGGAATATATATATTACTTAATATATAAGGTTTTGTGTCGAATACGTTTAGGCATTTCTGGTGATTGTTTTCGGGTGCATACCGGTCACGATTTGGCTCAGAACAACTAAGAACGTAACAGACGGCATTGTTTTTTGCACTGAAGGCCTCCGGTCAAGTCTAGTTTCTGATATTGAAAACATCGGAAAATTTACCTACCAGAAGACCAGTTCATCTACCACCGGTTTAGCCAACATTATAGATTCTTATCTCACAAACAATGATACTCATTTCAAAAAGATTCAAACACAGGTTTGTTTAAATTAATTTACGTCAATTCATGATTCTAGTGATTATACTAAGAGATATTAACATAACGGCAATGATATTTTTGTCCTTGTTGTTGTTATGTTTCAGGTCGCACCGGTGTTGTTTAAAGCTTATTTAACGATCCCTCAAGTCTCACAAGTTTCGTACATTAGCACGGACGGTCTCTTGTTTTCTTACAAAACAGAACTGAACGCAAGCGTCGCTGTCTTTGCGAACTCTTCAAGCGGTAAAGGAGACTACACTTGGTACACTCAAACCGTCGATCAGATAACCGGTCGTCTTACCGGGAATGCAAAGAAATCTAAGCCTGTGGATGTAACCCATAAAGACTGGTTCCAAGCAGTGCAGAGAAACCACACTACCGCCTTTGTGGGACCTGGTTTAGGAGGAGAAGTTAATGAGGCTATGTTTCAGAGCGTCGTTAGCTTGTACAGCAAGAAAGGAGCTGTTTCATTAGGGTTTCCGGTTAAAACGTTAATCGATTCTTTGAACCGTTTGCATCTAAAGGGTGGAGAGCTTTACTTGTGGACCAAGGAAGGGACCTTGATTGTTCCTGGAAGATCACTAAATGCTACTTTCTTCATCTCCAATGGCTCCATTTGCTTCGGTAGAGAATCGTCTCATTGCATCCCCGGAAACTGTAGTTCTCGTGGTTACCAGGTGGAGATCGGAAGATTGAAATTCCAAGCTTTTTGCTCTGTTCTTGAAGTTTCCGGCGTACCTCTGGTAAATGTTAAATCATCTTCTGATACTCTTTATAGATTCATAACTTTGTTTCTCAGATTCTTCCCTTGTTTTTTTTTTCCAGAGATACACACTAATGTTTCCCAACAAAGAAAGAACACCAAGCATCCGAAGTGCATCGCTATATCTTCTTGTTGCAACAATGTTATTGGGCTTGTGCTGGCCTCTAGGGTTTGTGGCGTGTATGGTGAACGCAGCAGGAAGAGAGATGCATATGCGTGCAACATTGATAAAACAAATGGAAGCAACACAACAGGCCGAGAGGAAAAGCATGAACAAGAGTCAAGCCTTTGCAAGAGCTAGCCACGATATAAGAGGTTCCCTTGCCGGGATTACTGGTCTTATTGATCTATGTCATGACTCTGAAGAAGTTAGACATGGGTCTAACCTAGAGTCTCGTCTTAAGCTAGTGAATGGCTGCACCAAGGATCTGCTTGGTAAGTTTTTTTTTTTTTGATTAACTGGGCTATGCCTGACCAATAGAGACTCTGATTCTTGACCTTTTCTTTTATGTGTTTTGCAATGTTTTGAAAACCGGACCAGATATTGACTTGACACTGAGCTAGGTCAATGATCGGACTTGTCCAACCGGTTCAACCGGGTTTTAAATTTTAAATTAGTTTTAGATTAAGTAACTATATATATAACTATAAAATTATGATTTATAAATTTTTATATAGTTGTTAGAATCTAAAAGTGATATGAAATTTTTTAAAATAATATAAAATATAATGAAAAATCATTTATTTAGATAGATATTTAAAAATATATATTTAAAAAAAACATTGCAGATTTTAAATATTTTTTATTTGAATTTGAGAACGGATCACCGGGTTTAGCGAGTTTCACCAGTTTTTGACCGGATTTGCCGGTTCAACTTCAATCCGGTTTTTAATACAACCCGAAATCGATTAGAAGGACGAGTAATAGGCCGACCAGCCGGTCCGGTTTTCAAAACAGTGGAGTTTTGTCACCTTGAACCCTAACAGTAAGTCTTGTAATGTTGTTTTAAAACAGATTTGCTTAACTCTGTTTTGGACACGAGCAAAATCGAAAGCGGGAAGATGCAGTTAAAGGAAGAAGAGTTCAACCTAGCAAAACTTGTGGAAGACGTCATTGATTTTTTTCATCCCGTGGCGATGAAGAAAGGGGTTGATGTGGTTTTGGATATGCACGATGGCTCGGTTTTCAAATGCTCTAACGTGCGAGGAGATGGTGGAAAACTGAAGCAAATCCTCAACAATCTTGTCAGCAATGCGGTCAAGTTCACGGTCGAGGGACACATTTCGATCCGAGCTTGGGCTCAGAGGACCAGTTCCAATGTGATCCTTGCACCGGAAAATAAAAGAGGTTTATCAAAGTTTTCAAAGAAGAGTAAAGACCAGGCCGGGTCACCGTCGAATTCAGTAAGAAACAATGGGAACATGATGGAGTTTGTGTTTGAAGTGGATGACACAGGGAAAGGGATACCAAAGGAGATGCGTAAGTCGGTGTTTGAGAACTATGTTCAGGTAAGAGAAACAGACCAAGGACAACAAGGAACTGGATTAGGACTTGGGATTGTGCAGTCTCTGGTAAGGTTAATGGGAGGGGAGATAAGAATCATTGACAAGGCCATGGGAGAGAAGGGAACATGTTTTCAGTTCAACGTTTTATTGTCGGCAGCATCAGAGTCTCAAGTGAGTAGACAGGACACGGAAGAAGGAGAACACATGCATGGGCTTATTAAAACTTCATCGGGAGGTAGCATGAGCATACGGAACATGAGCCCTAGGTTACACAATTGGCTCAGCTCAAGTCCAAAGCAAGAAAGGTCTCGAGTGGTTCTTCTGATGAAAGATGGAGAACGTAGAAGGGTTACAGAGAAATACATAAAGAGCCTTGGAATAAAAGTCACAGTGGTGAAAAAATGGGAGCATCTGAATCATGTTCTGGAGAGGCTTGGGGTTTCACGTCAGGGTTCCATGGGAAGAAACGAATCATTAAGCTCTAGCTCAAGGGAGTTGCCTTTGATTGGCATGGATGGGATTGATTCAAGAAGCCAGACTCCTAAAAGAACAAGGCATGGTTTCTCTCCAGCTCTCCTTGTGGTGATTGATGCGGAAACAGGACACTTTCTTGAGCTGTACGACATTGTTGAACAGTTTCGCAGAGGCATGCACCATGGTCTTTCCTGTAAAGTTGTTTGGCTTAATGATAGGGGACATGGAAGTTTGAGAGGGGAAATTAGCTGTTCTAAACCCTTGCATGGATCATGTCTCAACCGAGTGCTGAAGATGTTGCCTGAATTTGGAGCAACCGAGCCAAAAGAAGACAAACAAGGAGCTTTCAAGCCTAGTGAAGATGAGTTGTTGAGTGGAAAGAGGGTTCTGTTGGTGGATGATGATCGTATAACATCTTCAATTGCAACAATAAAGCTGAAAAAGATGGGAGCCTCTGAGGTCAAACAATGCTACAATGGGAAAGAAGCTGTGAGATTAGTGAGTGAATGGCTTACACAAAGAGAGCATGGAGAAGGAGGTTCATCAGAAGTGCTTCTTCCCTTTGACTACATATTCATGGACTGCCAGGTACATTTTATTTGATAAATCCTTCTTCCATAAATTAAGGAAAACGCTACATGGTTCAAAACTCCTACTGCATCATCATCTTTGTGGATACAGATGCCAGAAATGAATGGATATGAAGCAACTAGAGAGATAAGAAAAATGGAGGAAAAATATGGAGGTGGTTTGCATATACCAATTATAGCTGTATCTGGACATGAACTTGGTTCAACGGAAGCAAGAGAAACCATACAAGCTGGAATGGACGCCTTCTTGGAGAAAAACCTGAATCATGACCAACTTGCAAAGGTCATCAGAGAAATCACAAGCAAGGTATGGGCTCTCTCTAAACTAGTATTTTAAGTTGCTTATATCATTAGGCTTTTCTATTCTCGTTTCCATGGCATACAAATATTTTACATTTATCTGTCACAGGTTCAGAACCCATAAACCGGATTTTGATGGAAGAGGGGAGCTATGGATGCAAAACCATCTCCAGACTCCATCATCTGAAATTTTGATTGGTTTCCAACGGTTTCAAAGGGGGGGAGTACGCTTCTATCAGATTCACTGCTAGCTGGATGA

>BrHK1(Bra032761)-CDS

ATGGTGAATGTTAGAAAGTTTGTGACTTCTCGTCCAATATTTGTCTTCTTCCTCTTGGCATTTCTGGTGATTGTTTTCGGGTGCATACCGGTCACGATTTGGCTCAGAACAACTAAGAACGTAACAGACGGCATTGTTTTTTGCACTGAAGGCCTCCGGTCAAGTCTAGTTTCTGATATTGAAAACATCGGAAAATTTACCTACCAGAAGACCAGTTCATCTACCACCGGTTTAGCCAACATTATAGATTCTTATCTCACAAACAATGATACTCATTTCAAAAAGATTCAAACACAGGTCGCACCGGTGTTGTTTAAAGCTTATTTAACGATCCCTCAAGTCTCACAAGTTTCGTACATTAGCACGGACGGTCTCTTGTTTTCTTACAAAACAGAACTGAACGCAAGCGTCGCTGTCTTTGCGAACTCTTCAAGCGGTAAAGGAGACTACACTTGGTACACTCAAACCGTCGATCAGATAACCGGTCGTCTTACCGGGAATGCAAAGAAATCTAAGCCTGTGGATGTAACCCATAAAGACTGGTTCCAAGCAGTGCAGAGAAACCACACTACCGCCTTTGTGGGACCTGGTTTAGGAGGAGAAGTTAATGAGGCTATGTTTCAGAGCGTCGTTAGCTTGTACAGCAAGAAAGGAGCTGTTTCATTAGGGTTTCCGGTTAAAACGTTAATCGATTCTTTGAACCGTTTGCATCTAAAGGGTGGAGAGCTTTACTTGTGGACCAAGGAAGGGACCTTGATTGTTCCTGGAAGATCACTAAATGCTACTTTCTTCATCTCCAATGGCTCCATTTGCTTCGGTAGAGAATCGTCTCATTGCATCCCCGGAAACTGTAGTTCTCGTGGTTACCAGGTGGAGATCGGAAGATTGAAATTCCAAGCTTTTTGCTCTGTTCTTGAAGTTTCCGGCGTACCTCTGAGATACACACTAATGTTTCCCAACAAAGAAAGAACACCAAGCATCCGAAGTGCATCGCTATATCTTCTTGTTGCAACAATGTTATTGGGCTTGTGCTGGCCTCTAGGGTTTGTGGCGTGTATGGTGAACGCAGCAGGAAGAGAGATGCATATGCGTGCAACATTGATAAAACAAATGGAAGCAACACAACAGGCCGAGAGGAAAAGCATGAACAAGAGTCAAGCCTTTGCAAGAGCTAGCCACGATATAAGAGGTTCCCTTGCCGGGATTACTGGTCTTATTGATCTATGTCATGACTCTGAAGAAGTTAGACATGGGTCTAACCTAGAGTCTCGTCTTAAGCTAGTGAATGGCTGCACCAAGGATCTGCTTGATTTGCTTAACTCTGTTTTGGACACGAGCAAAATCGAAAGCGGGAAGATGCAGTTAAAGGAAGAAGAGTTCAACCTAGCAAAACTTGTGGAAGACGTCATTGATTTTTTTCATCCCGTGGCGATGAAGAAAGGGGTTGATGTGGTTTTGGATATGCACGATGGCTCGGTTTTCAAATGCTCTAACGTGCGAGGAGATGGTGGAAAACTGAAGCAAATCCTCAACAATCTTGTCAGCAATGCGGTCAAGTTCACGGTCGAGGGACACATTTCGATCCGAGCTTGGGCTCAGAGGACCAGTTCCAATGTGATCCTTGCACCGGAAAATAAAAGAGGTTTATCAAAGTTTTCAAAGAAGAGTAAAGACCAGGCCGGGTCACCGTCGAATTCAGTAAGAAACAATGGGAACATGATGGAGTTTGTGTTTGAAGTGGATGACACAGGGAAAGGGATACCAAAGGAGATGCGTAAGTCGGTGTTTGAGAACTATGTTCAGGTAAGAGAAACAGACCAAGGACAACAAGGAACTGGATTAGGACTTGGGATTGTGCAGTCTCTGGTAAGGTTAATGGGAGGGGAGATAAGAATCATTGACAAGGCCATGGGAGAGAAGGGAACATGTTTTCAGTTCAACGTTTTATTGTCGGCAGCATCAGAGTCTCAAGTGAGTAGACAGGACACGGAAGAAGGAGAACACATGCATGGGCTTATTAAAACTTCATCGGGAGGTAGCATGAGCATACGGAACATGAGCCCTAGGTTACACAATTGGCTCAGCTCAAGTCCAAAGCAAGAAAGGTCTCGAGTGGTTCTTCTGATGAAAGATGGAGAACGTAGAAGGGTTACAGAGAAATACATAAAGAGCCTTGGAATAAAAGTCACAGTGGTGAAAAAATGGGAGCATCTGAATCATGTTCTGGAGAGGCTTGGGGTTTCACGTCAGGGTTCCATGGGAAGAAACGAATCATTAAGCTCTAGCTCAAGGGAGTTGCCTTTGATTGGCATGGATGGGATTGATTCAAGAAGCCAGACTCCTAAAAGAACAAGGCATGGTTTCTCTCCAGCTCTCCTTGTGGTGATTGATGCGGAAACAGGACACTTTCTTGAGCTGTACGACATTGTTGAACAGTTTCGCAGAGGCATGCACCATGGTCTTTCCTGTAAAGTTGTTTGGCTTAATGATAGGGGACATGGAAGTTTGAGAGGGGAAATTAGCTGTTCTAAACCCTTGCATGGATCATGTCTCAACCGAGTGCTGAAGATGTTGCCTGAATTTGGAGCAACCGAGCCAAAAGAAGACAAACAAGGAGCTTTCAAGCCTAGTGAAGATGAGTTGTTGAGTGGAAAGAGGGTTCTGTTGGTGGATGATGATCGTATAACATCTTCAATTGCAACAATAAAGCTGAAAAAGATGGGAGCCTCTGAGGTCAAACAATGCTACAATGGGAAAGAAGCTGTGAGATTAGTGAGTGAATGGCTTACACAAAGAGAGCATGGAGAAGGAGGTTCATCAGAAGTGCTTCTTCCCTTTGACTACATATTCATGGACTGCCAGATGCCAGAAATGAATGGATATGAAGCAACTAGAGAGATAAGAAAAATGGAGGAAAAATATGGAGGTGGTTTGCATATACCAATTATAGCTGTATCTGGACATGAACTTGGTTCAACGGAAGCAAGAGAAACCATACAAGCTGGAATGGACGCCTTCTTGGAGAAAAACCTGAATCATGACCAACTTGCAAAGGTCATCAGAGAAATCACAAGCAAGGGGGGGAGTACGCTTCTATCAGATTCACTGCTAGCTGGATGA

>BrHK2(Bra006075)-DNA

ATGGTCTGCGAGATGGAGACTGATCAGACCGAAGAGATGGACATCGAAGTCTTGTCATCCATGTGGCCAGAAGATGTCGGAACCCAAGCAGACAACAACCAGTTCAACGTCGAGAAACCCGCGGGAGATTCAGACACCTTAAAAGAAGTCGACATCGCCGAGAAACGCACCATGGCGGATCTAAAACGCTTGCCGGAACTAATGAACACCACTGACCAAGGCACCTCTCAGCTAACCAACCTCGTGAAACAATGGGAGTACATGCAAGACCACGCGGTTAAGCTACTACGAGAAGAGCTCAAAATCCTCACGAAACAAAGAGAAGAAGCCGAAGCCAAGGAGCTTAAGATCATAGAAGAGCATAACTTCGAAAGCCAAGAGCCTGAGAACGTTCCTGTTTTGGATGATACGAGCCATTTGTTCCGAAGGTATAAGCACAAGAAACGTGATGCGTTGATCGGTAGCAAAAGGGTTGAGATCGACGAGGAGTTTGACACGGTTGCGTACTGGAAACAGAAGGCGTTGAGTTTGGAGAAGATGCTTGAAGCGAGTACCGAGAGAGAGAGGAGGTTGATTGAGAAGCTTAACGAGAGTTTGAAGACTATGGAGAGTCACTCTGCACCGGTTGAAGAGTTGACTCAGAATCTCAAGAGAGCTGAAGGGTTCTTGCATTTCATACTTCAGAATGCACCTATTGTTATGGGTCATCAGGATAAAGATCTACGTTACTTGTTCATCTACAACAAGTTTCCTACCTTACGAGAGCAGGTAAGATTAAGCTGTTTCTTGTTCTGTTCAATGTTCTAATCGGCTTAGGCGAATAAATCGTTCAGAGCCAAAATGATTTTTTTTTAAATCTTGATGTGCTTTATATTTGTCTAAAATGTTCTAAATCTGTTAAAAACGGTTTTAATATGTTTAAATCGATATAAATTAGTTTAAGTTTGTCAAATTAACAATAATGTTAGTGTAAATCCACAATTTTTTCCATTTTTTTTTGCATGTCTAAATTTTTATAATTCATCTAAATAATTTTATAATTAACCTTAAAAATTAAAATATCTAATGCAAAAATAAAATTTGTAAAAATAAATCAATAATCTGTTAAAAGCATATATTAATGTGTTTCTTACTAATGTCTCAGGACATATTGGGGAAGACAGACGTAGAGATATTTCATGGAGGTGGAGTCAAAGAGTCTGAATATTTCAAGAGAGAGGTTCTTGAAAAAGGCAAAGCTTCAAAGAGAGAAATCACATTTGAGACAGAGTTGTTCGGATCAAAGACATTTTTGATATACGTTGAGCCTGTTTACAACAAAGCACGTGAGAAAATCGGTATCAACTACATGGGAATGGAAGTAACTGATCAGGTGAGGAAGAGAGAAAAAATGGCTAAACTTAGAGAAGACAATGCAGTGAGAAAAGCAATGGAATCAGAGCTGAACAAGACCATTCACATTACAGAGGAGACAATGAGAGCTAAGCAGATGTTAGCTACAATGTCACATGAGATAAGATCACCGTTGTCAGGAGTAGTGGGAATGGCTGAGATACTTTCAACTACAAAGTTGGATAAAGAGCAAAGACAGTTGTTGACTGTTATGATGTCTTCTGGTGATTTGGTGCTTCAGTTGATTAATGATATTCTTGATCTCTCCAAGGTTGAATCAGGTAAGATAACAATGTTTTTTTTTCAAATTGTGTTTCCCATCTTTTTCAGATTCTTGATGACATGTTTTGTGTGTTGTTTTAAGGTGTGATGAGACTAGAAGCTACCAAGTTCAGACCAAGAGAAGTGGTGAAGCATGTGCTTCAGACAGCTGCTGCATCTCTCAAGAAAGACTTGACATTAGAAGGAAATATTACAGATGAAGTTCCAATTGAGGTTATTACACAAACAACACATTGTCTGAAAAGAGTTAACGTGGCAAAGTCTTTGTGATCTAACTTTTTTCATGTTCAGGTAGTTGGAGATGTTCTAAGGATCAGGCAGATTCTCACCAACTTGATCAGCAATGCTATCAAGTTTACACATGAAGGAAAGGTTGGGATCAAACTCAAAGTAATATCAGAACCATCCTTTGCGAGTGGTATGGAGCTAAACGCAGACGCTGAAGAACAAAACGGTTTGACTGAGACAGAGACTTCGGTTTGGATCCGCTGTGACGTTTATGATACTGGAATTGGAATCCCAGGCAAGTTCAAGAATAAAACTCAAGCTAAGCTATTAGACCTGCTGTTATTACCCGAACATGTCCAACCAAACCGAAAAGTTCGATTTTCAGTTAGTTCAGTTAGAAGATTCGATTCAGTCAGGAAACTAAAAAATATTTGGTTTTCGGTTTGGTTCATTAACCGGTTAGTTCTTTTAAAAAAAAAAAGAAAAAAATACCAAACAGTACTAATTTCAACCAAAATTCCAACCGAACTAACCAAAATCTGAACCGATTAACTGATCCGAACTAACCGAAGAAACCGAACCCGCAGGCCTATAAGCTATAGTTATTAAATATTAACCATGTCCATGTTTTGGTGTTTCTTTGTGCAGAAAAAGCACTTCCTTGTTTGTTCAAGAAGTACATGCAAGCAAGCGCTGATCACGCTCGCAAATACGGTGGAACTGGTCTCGGTCTCGCCATTTGTAAACAGCTGGTAAACTAATCTTCACATTACACAACACAGGTTTTAAAACTATTCATCACAGTAGAGGCAAACCATTCTGTTGTTTGTTTAACAGGTTGAGTTGATGGGAGGTCAACTTACAGTGACAAGCCAGGTCAACGCCGGTTCAACGTTCACGTTCATATTACCATACAAAGTTGCAACATCAGATGATCATTCGGATGATCAAGATTTCTCTGACATGGTGGATCATCACCAACCAGAACCAGACGACACAACCGAAGGATATTTCCAGTTTAAACCGCTTCTTGGTTCTATATATTCTAATGGAGGACCGGTCATAGGCAATAATAACTTCTTACCTCATAAAGTCATGCTCACTAGCCCTCTTAAGCTGATCAACGGCTTTGTCGCCGATCCTTCTAACAACACTGGACAAAGCGAGACGACTCAGGTTGAAAACAATGGTTACATGGATGAAACCTGTTCTGGTCCATGTCCCTCTAAGGAAACAGAATCTTGTAGTAGTTCACAAGCTAGCTCAGAAGGTGGACCCTTGGAAATGGAGTCAGAGCTTACAGTTTCATCTCGCAGGGAAGACGAAACAACAGAGACATCAAAGCAGCCAAAGATTTTGCTTGTGGAAGATAATAAAATCAACATCATGGTTGCAAAGTCTATGATGAAGCAGTTAGGCTATACCTTTGATATTGCTAATAATGGAGTTGAAGCCATAAACGCCATTAAAGACTCCAGCTACGACTTGGTACTAATGGTAAGTCAATTCTCCAAGATCCAAAGACTATTCTTTTCAACTAAACTTAACTTGAAGAGACATAAAACAATATTGGTTCACAGGATGTGTGCATGCCAGTTCTGGATGGTTTAAAAGCCACAAGACTGATACGTTCCTACGAAGAATCTGGGAACTGGGATGCTGCAATAGAAGCTGGAGTAGATATAAAGATATCAGAGAATGAACAAGCTTGTGTGCATTCCACAAACCGGCTGCCTATAATAGCGGTTAGTACTTCTTCATCACATACAGCTCTTAGGTCTGGGAATTTTAGGTATCAGTTCGGCTAAGATATTTCGGGTTAGGGTCTATAAGATCCATTTACTACCTGACCTATTTTTGGTTCGGGTTTGGATTTAGCTCGGCTAGTTTCGGGTTCATATTTGGTTCGTTTCAGATAGAATAACCACTGAACCCGAAAATATCCTCGGTTTCCATTTAGTTTTTGTTTCGGGTATAGAGACATAAGAACATTCAGATATTTGAGGGTTTCGGTCCAGTTCTAGTTTCGGTTATTTCGGTTCGGATGTTAGGTTCCGGTTTTTTTGCCCTGAACTATGGATACATACCTTGACAAAATTTTGAAGAGTAAAATGCGTTGTGGAATTTTGCAGATGACGGCAAATACATTAGCAGAGAGCTCAGAAGAATGTTATGCAAATGGTATGGACTCTTTTATTTCAAAACCTGTAACGTTGCAAAAACTAAAAGAGTGTCTACGACAGTATCTGCACTGA

>BrHK2(Bra006075)-CDS

ATGGTCTGCGAGATGGAGACTGATCAGACCGAAGAGATGGACATCGAAGTCTTGTCATCCATGTGGCCAGAAGATGTCGGAACCCAAGCAGACAACAACCAGTTCAACGTCGAGAAACCCGCGGGAGATTCAGACACCTTAAAAGAAGTCGACATCGCCGAGAAACGCACCATGGCGGATCTAAAACGCTTGCCGGAACTAATGAACACCACTGACCAAGGCACCTCTCAGCTAACCAACCTCGTGAAACAATGGGAGTACATGCAAGACCACGCGGTTAAGCTACTACGAGAAGAGCTCAAAATCCTCACGAAACAAAGAGAAGAAGCCGAAGCCAAGGAGCTTAAGATCATAGAAGAGCATAACTTCGAAAGCCAAGAGCCTGAGAACGTTCCTGTTTTGGATGATACGAGCCATTTGTTCCGAAGGTATAAGCACAAGAAACGTGATGCGTTGATCGGTAGCAAAAGGGTTGAGATCGACGAGGAGTTTGACACGGTTGCGTACTGGAAACAGAAGGCGTTGAGTTTGGAGAAGATGCTTGAAGCGAGTACCGAGAGAGAGAGGAGGTTGATTGAGAAGCTTAACGAGAGTTTGAAGACTATGGAGAGTCACTCTGCACCGGTTGAAGAGTTGACTCAGAATCTCAAGAGAGCTGAAGGGTTCTTGCATTTCATACTTCAGAATGCACCTATTGTTATGGGTCATCAGGATAAAGATCTACGTTACTTGTTCATCTACAACAAGTTTCCTACCTTACGAGAGCAGGACATATTGGGGAAGACAGACGTAGAGATATTTCATGGAGGTGGAGTCAAAGAGTCTGAATATTTCAAGAGAGAGGTTCTTGAAAAAGGCAAAGCTTCAAAGAGAGAAATCACATTTGAGACAGAGTTGTTCGGATCAAAGACATTTTTGATATACGTTGAGCCTGTTTACAACAAAGCACGTGAGAAAATCGGTATCAACTACATGGGAATGGAAGTAACTGATCAGGTGAGGAAGAGAGAAAAAATGGCTAAACTTAGAGAAGACAATGCAGTGAGAAAAGCAATGGAATCAGAGCTGAACAAGACCATTCACATTACAGAGGAGACAATGAGAGCTAAGCAGATGTTAGCTACAATGTCACATGAGATAAGATCACCGTTGTCAGGAGTAGTGGGAATGGCTGAGATACTTTCAACTACAAAGTTGGATAAAGAGCAAAGACAGTTGTTGACTGTTATGATGTCTTCTGGTGATTTGGTGCTTCAGTTGATTAATGATATTCTTGATCTCTCCAAGGTTGAATCAGGTGTGATGAGACTAGAAGCTACCAAGTTCAGACCAAGAGAAGTGGTGAAGCATGTGCTTCAGACAGCTGCTGCATCTCTCAAGAAAGACTTGACATTAGAAGGAAATATTACAGATGAAGTTCCAATTGAGGTAGTTGGAGATGTTCTAAGGATCAGGCAGATTCTCACCAACTTGATCAGCAATGCTATCAAGTTTACACATGAAGGAAAGGTTGGGATCAAACTCAAAGTAATATCAGAACCATCCTTTGCGAGTGGTATGGAGCTAAACGCAGACGCTGAAGAACAAAACGGTTTGACTGAGACAGAGACTTCGGTTTGGATCCGCTGTGACGTTTATGATACTGGAATTGGAATCCCAGGCAAGTTCAAGAATAAAACTCAAGCTAAGCTATTAGACCTGCTGTTATTACCCGAACATGTCCAACCAAACCGAAAAGTTCGATTTTCAGTTAAAAAAGCACTTCCTTGTTTGTTCAAGAAGTACATGCAAGCAAGCGCTGATCACGCTCGCAAATACGGTGGAACTGGTCTCGGTCTCGCCATTTGTAAACAGCTGGTTGAGTTGATGGGAGGTCAACTTACAGTGACAAGCCAGGTCAACGCCGGTTCAACGTTCACGTTCATATTACCATACAAAGTTGCAACATCAGATGATCATTCGGATGATCAAGATTTCTCTGACATGGTGGATCATCACCAACCAGAACCAGACGACACAACCGAAGGATATTTCCAGTTTAAACCGCTTCTTGGTTCTATATATTCTAATGGAGGACCGGTCATAGGCAATAATAACTTCTTACCTCATAAAGTCATGCTCACTAGCCCTCTTAAGCTGATCAACGGCTTTGTCGCCGATCCTTCTAACAACACTGGACAAAGCGAGACGACTCAGGTTGAAAACAATGGTTACATGGATGAAACCTGTTCTGGTCCATGTCCCTCTAAGGAAACAGAATCTTGTAGTAGTTCACAAGCTAGCTCAGAAGGTGGACCCTTGGAAATGGAGTCAGAGCTTACAGTTTCATCTCGCAGGGAAGACGAAACAACAGAGACATCAAAGCAGCCAAAGATTTTGCTTGTGGAAGATAATAAAATCAACATCATGGTTGCAAAGTCTATGATGAAGCAGTTAGGCTATACCTTTGATATTGCTAATAATGGAGTTGAAGCCATAAACGCCATTAAAGACTCCAGCTACGACTTGGTACTAATGGATGTGTGCATGCCAGTTCTGGATGGTTTAAAAGCCACAAGACTGATACGTTCCTACGAAGAATCTGGGAACTGGGATGCTGCAATAGAAGCTGGAGTAGATATAAAGATATCAGAGAATGAACAAGCTTGTGTGCATTCCACAAACCGGCTGCCTATAATAGCGATGACGGCAAATACATTAGCAGAGAGCTCAGAAGAATGTTATGCAAATGGTATGGACTCTTTTATTTCAAAACCTGTAACGTTGCAAAAACTAAAAGAGTGTCTACGACAGTATCTGCACTGA

>BrHK3(Bra009011)-DNA

ATGGTCTGCGAGATGGAGACTGATCAAATCGAAGAGATGGACGTCGAAGTCTTGTCTTCCATGTGGCCCGATGATGTCGGAACCCAAGCAGACAACCAGTTCAACGTAGAGAAACCCGCAGGGGATTCAGACACCTTGAAAGAAGTAGACATCGCCGAGAAACGAACCATGGCTGATCTAAAACGTTTACCCGAACTCCTGAACACCACCGACCAAGGCTCCTCACAACTCACCAACCTCGTGAGACAATGGGAGTACATGCAAGACCACGCGGTTAGGCTTCTAAGAGAAGAGCTCAAGATCCTCACCAGACAGAGAGAAGAAGCTGAGGCCAAGGAGCTTAAGATCATAGAGGAGCATAACTTCGAGACCGAAGAGCCTGAGAACGTCCCCGTTTTGGATGAGACCAGCCATCTTTTCCGCAGGTTTAGGCAGAAGAAACGCGACGAGCTGGTGGACAGCAAGAGGGTTGTGATCGATGAGGAGTTCGACACGGTTGCGTATTGGAAACAGAAGGCGTTGAGTTTGGAGAAGATGCTTGAAGCGAGTACCGAGAGAGAGAGGAGGTTGATTGAGAAGCTGAACGAGAGTTTGAAGACTATGGAGAGTCACTCAGCACCGGTGGAAGAGCTGACTCAGAATCTTAAGAGAGCTGAAGGGTTCTTGCATTTCATACTTCAGAATGCACCTATTGTTATGGGTCATCAGGATAAAGATCTACGTTACTTGTTCATCTACAACAAGTTTCCTTCGTTACGAGAACAGGTAAGAGCAATGTTTAAGAAATTCATTAGACGGTAACTAGATGTTTTATAGCGAACAGTGACTAGAGGGATGATTCGAGGTTTACATGAGGCTTAGGCGTTAGTAAGAGAATCCTGTTTCTTCTTTGTTCTGTTAGATCACTGATCATTCTAATGAACGTGTGTTTCTTACTAATGTGTCAGGACATTTTGGGCAAAACAGACGTGGAGATATTCCACGGAGGTGGAGTTAAAGAGTCTGAAGATTTCAAGAGAGAGGTTCTTGAAAAAGGAAAAGCTTCAAAGAGAGAGATCACATTCGAGACAGAGTTATTCGGATCAAAGACGTTTTTGATATACGTTGAGCCTGTTTACAACAAAGCTCGCGAGAAAATCGGTATAAACTACATGGGAATGGAAGTAACTGATCAGGTAAGGAAAAGAGAAAAAATGGCTAAGCTCAGGGAAGACAACGCGGTGAGAAAAGCGATGGAATCAGAACTGACCAAGACCATTCACATCACAGAGGAGACTATGAGAGCTAAGCAAATGCTGGCGACAATGTCTCATGAGATAAGATCACCGTTGTCAGGAGTGGTGGGAATGGCTGAGATACTTTCTACTACAAAGCTGGATAAAGAGCAAAGACAGTTGTTGAATGTCATGATCTCTTCTGGTGATTTGGTGCTTCAGCTGATTAATGATATTCTTGATCTCTCCAAGGTTGAATCAGGTAAGATAGTATTGTGATCTTGTTGTCTGGTCATGTATTCAAGATTGTTAATAACCATAATAGTAAAGATTTTAATATTTTTTTTCACAAATTTAGAAGTCTACCTACGCATTCTTCAAAATTTTGTGGGTCATATGCCAATGTTTAACTGGCCTATGTCCAGGACCGATCATGTTAATGACATATGTTTCTGTGTGTTGTTTGTAGGTGTGATGAAGTTAGAAGCTACAAAGTTTAGGCCAAGAGAAGTGGTGAAGCATGTGCTTCAGACAGCTGCTGCATCGCTGAAGAAAGATTTGACGTTAGAAGGAAACATTGCAGATGAAGTTCCTATATTGGTTAGTACACAAACAACACATTGTTCCAAGTGGCAAAGTCATTGTAATCTTAACTTTATTCTTTTTTCATGTTCAGGTGGTTGGAGATGTTCTAAGGATCCGGCAGATTCTCACCAACCTGATCAGCAATGCTATCAAATTTACACATCGAGGAAAGGTTGGGATCAAACTCAAAGTGATATCACAACCATCCTTTGCTAGTGATAAAGAACAAAACGAGACTTCGGTTTGGATTTGCTGTGACGTTTATGACACTGGAATTGGAATCCCAGGTACGAAAGCTTTGTTCAAGAACATCAAAACATAGAAACTCCAGCTAAGCTTTGTTCATAATGCAGAGAACGCTCTCCCTTGTTTGTTCAAGAAGTACATGCAAGCAAGCGCTGATCATGCTCGCAAATACGGTGGAACTGGTCTCGGTCTCGCCATTTGTAAACAGCTGGTTGAGCTAATGGGAGGTCAACTCACAGTGACAAGCCAAGTCAACTTAGGTTCAACGTTCACGTTCGTATTACCATACAAAGTTGCAACACCAGATGATCATTCAGATGATCAAGATGAGTTCTCTGACATGGTTGATCATCATCAACCAGAGCCAGACGACACAACCGAAGGATACTTCCAGTTTAAACCCCTTCTAGGATCTATATATTCCAATGGCGGACCGGTCATGGGCAATAACTTCTTACCTCACAAAGCTATGCTCACTAGTCCTATTAAGCTCATCAACGGTTCTGTCGCCGATCCTTCTAACAGCAGTGGACAAAGCCAGACGGTTCAGGTTGAAAATGGTGGTTATATGGATGAATCTGAGTCGGCTCATCAATATGGCAATGGGAATGGTCATCGATGTTCCTCCAAGGAAAGTGAATCTTGTAGCAGTTCACAAGCTAGCTCAGAAATGGAGTCAGAGCTCACAGTTTCATCTCCTAGGGAAGAGGAAAAAACTGAGACGGAGGTCAAAGAGACATCACAGCCAAAGATTTTGCTTGTGGAAGATAACAAAATCAACATCATGGTTGCCAAGTCGATGATGAAGCAACTAGGCTATACCATGGACATTGCCAATAACGGAGTTGAAGCCATAAACGCTGTTAAAGACACTAGCTACGACTTGGTACTCATGGTAAGTCAACTTGTAAGTTCACTTAATTTGAAAAAAAGGCTTATAAAAGAATTTTGGGGTTCACAGGATGTGTGCATGCCAGTTATGGATGGTTTAAAAGCTACAAGACTGATCCGTTCATACGAAGAATCTGGGAACTGGGATGCTGCAATAGAAGCAGGAGTTGATATAAAGACATCAGAGAGTGAGCAAGGCTGTGAGCGTTCCACTGACCGGCTGCCTATAGTCGCTATGACCGCTAATACATTAGCAGAGAGCTCAGAAGAATGTTATGCAAATGGTATGGACTCTTTTATTTCTAAACCTGTAACGTTGCAAAAACTTAAAGAGTGTCTACAACAGTATCTCCAGTGA

>BrHK3(Bra009011)-CDS

ATGGTCTGCGAGATGGAGACTGATCAAATCGAAGAGATGGACGTCGAAGTCTTGTCTTCCATGTGGCCCGATGATGTCGGAACCCAAGCAGACAACCAGTTCAACGTAGAGAAACCCGCAGGGGATTCAGACACCTTGAAAGAAGTAGACATCGCCGAGAAACGAACCATGGCTGATCTAAAACGTTTACCCGAACTCCTGAACACCACCGACCAAGGCTCCTCACAACTCACCAACCTCGTGAGACAATGGGAGTACATGCAAGACCACGCGGTTAGGCTTCTAAGAGAAGAGCTCAAGATCCTCACCAGACAGAGAGAAGAAGCTGAGGCCAAGGAGCTTAAGATCATAGAGGAGCATAACTTCGAGACCGAAGAGCCTGAGAACGTCCCCGTTTTGGATGAGACCAGCCATCTTTTCCGCAGGTTTAGGCAGAAGAAACGCGACGAGCTGGTGGACAGCAAGAGGGTTGTGATCGATGAGGAGTTCGACACGGTTGCGTATTGGAAACAGAAGGCGTTGAGTTTGGAGAAGATGCTTGAAGCGAGTACCGAGAGAGAGAGGAGGTTGATTGAGAAGCTGAACGAGAGTTTGAAGACTATGGAGAGTCACTCAGCACCGGTGGAAGAGCTGACTCAGAATCTTAAGAGAGCTGAAGGGTTCTTGCATTTCATACTTCAGAATGCACCTATTGTTATGGGTCATCAGGATAAAGATCTACGTTACTTGTTCATCTACAACAAGTTTCCTTCGTTACGAGAACAGGACATTTTGGGCAAAACAGACGTGGAGATATTCCACGGAGGTGGAGTTAAAGAGTCTGAAGATTTCAAGAGAGAGGTTCTTGAAAAAGGAAAAGCTTCAAAGAGAGAGATCACATTCGAGACAGAGTTATTCGGATCAAAGACGTTTTTGATATACGTTGAGCCTGTTTACAACAAAGCTCGCGAGAAAATCGGTATAAACTACATGGGAATGGAAGTAACTGATCAGGTAAGGAAAAGAGAAAAAATGGCTAAGCTCAGGGAAGACAACGCGGTGAGAAAAGCGATGGAATCAGAACTGACCAAGACCATTCACATCACAGAGGAGACTATGAGAGCTAAGCAAATGCTGGCGACAATGTCTCATGAGATAAGATCACCGTTGTCAGGAGTGGTGGGAATGGCTGAGATACTTTCTACTACAAAGCTGGATAAAGAGCAAAGACAGTTGTTGAATGTCATGATCTCTTCTGGTGATTTGGTGCTTCAGCTGATTAATGATATTCTTGATCTCTCCAAGGTTGAATCAGGTGTGATGAAGTTAGAAGCTACAAAGTTTAGGCCAAGAGAAGTGGTGAAGCATGTGCTTCAGACAGCTGCTGCATCGCTGAAGAAAGATTTGACGTTAGAAGGAAACATTGCAGATGAAGTTCCTATATTGGTGGTTGGAGATGTTCTAAGGATCCGGCAGATTCTCACCAACCTGATCAGCAATGCTATCAAATTTACACATCGAGGAAAGGTTGGGATCAAACTCAAAGTGATATCACAACCATCCTTTGCTAGTGATAAAGAACAAAACGAGACTTCGGTTTGGATTTGCTGTGACGTTTATGACACTGGAATTGGAATCCCAGAGAACGCTCTCCCTTGTTTGTTCAAGAAGTACATGCAAGCAAGCGCTGATCATGCTCGCAAATACGGTGGAACTGGTCTCGGTCTCGCCATTTGTAAACAGCTGGTTGAGCTAATGGGAGGTCAACTCACAGTGACAAGCCAAGTCAACTTAGGTTCAACGTTCACGTTCGTATTACCATACAAAGTTGCAACACCAGATGATCATTCAGATGATCAAGATGAGTTCTCTGACATGGTTGATCATCATCAACCAGAGCCAGACGACACAACCGAAGGATACTTCCAGTTTAAACCCCTTCTAGGATCTATATATTCCAATGGCGGACCGGTCATGGGCAATAACTTCTTACCTCACAAAGCTATGCTCACTAGTCCTATTAAGCTCATCAACGGTTCTGTCGCCGATCCTTCTAACAGCAGTGGACAAAGCCAGACGGTTCAGGTTGAAAATGGTGGTTATATGGATGAATCTGAGTCGGCTCATCAATATGGCAATGGGAATGGTCATCGATGTTCCTCCAAGGAAAGTGAATCTTGTAGCAGTTCACAAGCTAGCTCAGAAATGGAGTCAGAGCTCACAGTTTCATCTCCTAGGGAAGAGGAAAAAACTGAGACGGAGGTCAAAGAGACATCACAGCCAAAGATTTTGCTTGTGGAAGATAACAAAATCAACATCATGGTTGCCAAGTCGATGATGAAGCAACTAGGCTATACCATGGACATTGCCAATAACGGAGTTGAAGCCATAAACGCTGTTAAAGACACTAGCTACGACTTGGTACTCATGGATGTGTGCATGCCAGTTATGGATGGTTTAAAAGCTACAAGACTGATCCGTTCATACGAAGAATCTGGGAACTGGGATGCTGCAATAGAAGCAGGAGTTGATATAAAGACATCAGAGAGTGAGCAAGGCTGTGAGCGTTCCACTGACCGGCTGCCTATAGTCGCTATGACCGCTAATACATTAGCAGAGAGCTCAGAAGAATGTTATGCAAATGGTATGGACTCTTTTATTTCTAAACCTGTAACGTTGCAAAAACTTAAAGAGTGTCTACAACAGTATCTCCAGTGA

>BrHK4(Bra028573)-DNA

ATGGTCTGCGAGATGGAGACTGATCATCAGACCGAAGAGATGGACGTCGAAGTCTTATCTTCCATGTGGCCTGAAGACGTCGGAGGAACCGAACCAGACAACCAGTTCAACGTCGAGAAACCCGCAGGAGATTCCGACACCTTAAAAGAAGTCGAATTCGCCGAGAAACGCACCATGGCGGATCTAAAACGTTTACCTGACCTCCTGAACACAACAGACCAAGGCTCCTCTCAGCTCACCAACCTCGTGAAGCAATGGGAGTATATGCAGGACCACGCGGTTAGACTATTGCGAGAAGAGCTCAAGATTTTGACTAAGCAAAGAGAAGAAGCCGAGGCCAAGGAGCTTAAGATCATAGAGGAGCACAACTTCGAGACCGAGGAGCCTGAGAATGTTCCGGTTTTGGACGAGAGCAGCGATCTGTTCCGCAGGTTTAAGGAGAAGAAGAGGGATAAGTTGGTCGGTAGGAAGAGGATTGAGATCGATGAGGAGTTTGATACTGTTGCGTACTGGAAACAGAAGGCGTTGAGCTTGGAGAAGATGCTTGAGGCGAGTACCGAGAGAGAGAGGAGGTTGATTGAGAAGCTTAACGAGAGTTTGAAGACTATGGAGAGTCACTCGGCACCGGTTGAAGAGTTGACTCAGAATCTTAAAAGAGCTGAAGGGTTCTTGCATTTTATTCTTCAGAATGCTCCTATTGTTATGGGTCATCAGGTAAGTAAAATGTTTGATTCGCGTTTATTCACAGATTTGTTGATCGTAGATCAAGGTTCTAAAAATCGGTATAAGAAGCAACTCAGTCTTAATCGAAACCATTTAAAAAAAATAACTGATTTATATTATTCAAATCAGTTTAAACTGTTATAAATCGTCTAAAATTAGTTTTAGTCGATATAAATTAGTCTAAATAGGCAATAATGTTAGTTCAAATTCACAATTTTGTCTAATTGTTTTTTTTTTGTATATCTAATTTTGATAATTAAATATGAAAACTAAAATATTTTATATAAGTGTAATTATACATAAAATAAATCAATAATTAATTAATGTTTCTCTGATTCTCTGCTTTTTTTTTTTTTCAGGATAAAGATCTACGCTACTTGTTCATCTACAATAAGTTTCCTACACTACGAGAACATGTAAGAGACAAAGTAAGTTATGTTCTTGTTTGTCTTCTCGTTTACTGATCATGTTAATACTCAGGACATATTGGGGAAAACAGACGTTGAGATATTCCATGGAGGTGGAGTTAAAGAATCCGAAGATTTCAAGAGAGAAGTTCTTGAAAAAGGAAAAGCTTCAAAGAGAGAGATCACATTCGAGACAGACTTGTTTGGTTCAAAGACTTTTTTGATATACGTTGAGCCTGTTTACAACAAAGCTCGCGAGAAAATCGGTATAAACTACATGGGAATGGAAGTAACTGATCAGGTGAGGAAGAGAGAAAAAATGGCTAAACTTAGAGAAGACAACGCGGTGAGAAAGGCAATGGAATCAGAACTGACCAAGACCATTCACATTACTGAGGAGACAATGAGAGCTAAGCAGATGCTGGCGACGATGTCTCATGAGATAAGATCACCATTGTCAGGAGTAGTGGGGATGGCTGAGATACTTTCTACTACAAAGCTGGATAAAGAGCAGAGACAGTTGTTGAATGTCATGATCTCTTCTGGTGATTTGGTGCTTCAGTTTATTAATGATATTCTTGATCTCTCCAAGGTTGAATCAGGTAATGTTTTCAAGTTTTGGTCTTGTTGTGTGGTCATTTTTCAAGATTCTTGATGACATAACCACATTGGACTGGTCTATTGGTAAGCGGACTTTGAGAAGACTTGGGTTCGAAATATCCCAGGATACTAAATTTGTGTGGCCATGCGGATATAAGTTCATGATTTGGACCTCATTTGAAAATTTATCGATAGTGTCTATCAGTAGACTGCACCTACCATTAGAGTACCTACCATTAAAAGATTAGTTGGAGTCCTTCCATAGGCTTGGGATACCCATGTTAATAATAATTAAAAAAATTCTTGATGACGTATGGTGTTTTTGTGTGTTGTTTTTAGGTGTGATGAAGTTAGAAGCTACCAAGTTTAGGCCAAGAGAAGTAGTGAAGCATGTGCTTCAGACAGCTGCTGCATCTCTGAAGAAAGAACTGACATTAGAAGGGAACATTGCAGATGAAGTCCCTATCTTGGTTAGTACACAAAGAGATAATGTGGTAAAGTCGTTGTAATCTAACTTCTTTTTTTTCATGTTTTTAGGTAGTTGGAGATGTTCTAAGGATCCGGCAGATTCTCACCAACTTGATCAGCAATGCTATCAAGTTTACACATCAAGGAAAGGTTGGCATCAAACTCAAAGTGATACCAGAACCATCCTTTGCAAGTGGTTTGGAGTTAAACGCAGACGCTGAAGAACAAAACGGTTTGACTGAGACTGAGACTTCGGTTTGGATTCGCTGCGATGTTTATGACACTGGAATTGGAATCCCGGGTAAGCCAGAAAGCTCCATAACATCAAAATACAAAACTCCAGCTAAGCTAGTTATTGATACGGTCCATGTTTTATTGTGCAGAAAATGCTCTTCCTTGTTTGTTCAAGAAGTACATGCAAGCAAGCGCTGATCATGCCAGAAAATACGGTGGAACTGGTCTCGGTCTCGCCATTTGTAAACAGCTGGTAGAGCTAATGGGAGGTCAACTTACTGTGACGAGCCAAGTCGACTTAGGTTCAACGTTCACGTTCATATTACCCTATAAAGTTGCAACATCGAATGACCATTCGGATGATCAAGATGAGTTCTCTGATATGGTGGATCATCAACCCGAACCAGACGACTCAACCGAAGGATACTTCCAGTTTAAACCGCTTCTAGGATCTATATACTCTAATGGCGGACCGGTCATTGGCAATAACTTCTTACCTCATAAAGTTATGCTCCCTAGTCCTGTTAAGCTCATCAACGGTCAAAGCGAGGCTGTTCAGGTTGAAAACGGTGGTTATATGGATGGACCAAGACATGAAACCCGGTCTGGTCACTGTCCTGAATCACCTCATCAATATGAGAATGGGAATGGTCCATGTCCCTCTAAGGAAAGCGAGTCTTGTAGCAGTTCACAAGCTAGCTCAGAAATGGAGTCAGAGTTTACAGTTTCATCTCCTAGGGAAGAGAAGAAAACTGAGACAGAGGTCAAAAAGACATCAAAGCAGCCAAAGATTTTGCTTGTGGAAGATAATAAAATAAACATCATGGTTGCAAAGTCGATGATGAAACAATTAGGCTATACCTTTGATATTGCTAATAATGGAGTTGAAGCTATAACTGCTATTAACGGTTCTAGCTACGATTTGGTACTCATGGTAAGACTATTATTCTCTAAGATCCAAAAGACTTGTCCTTCTCAAATTCACTTAATCTAAAAAAGGCTTATGAATCAACTTGGGTTCACAGGATGTGTGCATGCCTGTACTGGATGGTTTAAAAGCTACAAGACTGATCCGTTCGTACGAACAATCTGGGAACTGGGATGCTGCAGTAGAAGCTGGAGTAGACATCAAGACACTGGAGGATAAGCAACTCTGTGTGCGTTCCACAAACCGGCTGCCTATAATCGCGGTTAGAACATATTCTTCAAATAACGTTGAACACAGAGCTCTAATGGTTAACTTTAACGAGTAAAATGGGTTGTGGATTTTTTTTTGCAGATGACGGCAAATACATTATCAGAGAGCTCAGAAGAATGTTATGCAAATGGTATGGACTCTTTTATTTCGAAACCTGTAACGTTGCAGAAACTAAAAGAGTGTCTAAAACAGTATCTGCATTGA

>BrHK4(Bra028573)-CDS

ATGGTCTGCGAGATGGAGACTGATCATCAGACCGAAGAGATGGACGTCGAAGTCTTATCTTCCATGTGGCCTGAAGACGTCGGAGGAACCGAACCAGACAACCAGTTCAACGTCGAGAAACCCGCAGGAGATTCCGACACCTTAAAAGAAGTCGAATTCGCCGAGAAACGCACCATGGCGGATCTAAAACGTTTACCTGACCTCCTGAACACAACAGACCAAGGCTCCTCTCAGCTCACCAACCTCGTGAAGCAATGGGAGTATATGCAGGACCACGCGGTTAGACTATTGCGAGAAGAGCTCAAGATTTTGACTAAGCAAAGAGAAGAAGCCGAGGCCAAGGAGCTTAAGATCATAGAGGAGCACAACTTCGAGACCGAGGAGCCTGAGAATGTTCCGGTTTTGGACGAGAGCAGCGATCTGTTCCGCAGGTTTAAGGAGAAGAAGAGGGATAAGTTGGTCGGTAGGAAGAGGATTGAGATCGATGAGGAGTTTGATACTGTTGCGTACTGGAAACAGAAGGCGTTGAGCTTGGAGAAGATGCTTGAGGCGAGTACCGAGAGAGAGAGGAGGTTGATTGAGAAGCTTAACGAGAGTTTGAAGACTATGGAGAGTCACTCGGCACCGGTTGAAGAGTTGACTCAGAATCTTAAAAGAGCTGAAGGGTTCTTGCATTTTATTCTTCAGAATGCTCCTATTGTTATGGGTCATCAGGATAAAGATCTACGCTACTTGTTCATCTACAATAAGTTTCCTACACTACGAGAACATGACATATTGGGGAAAACAGACGTTGAGATATTCCATGGAGGTGGAGTTAAAGAATCCGAAGATTTCAAGAGAGAAGTTCTTGAAAAAGGAAAAGCTTCAAAGAGAGAGATCACATTCGAGACAGACTTGTTTGGTTCAAAGACTTTTTTGATATACGTTGAGCCTGTTTACAACAAAGCTCGCGAGAAAATCGGTATAAACTACATGGGAATGGAAGTAACTGATCAGGTGAGGAAGAGAGAAAAAATGGCTAAACTTAGAGAAGACAACGCGGTGAGAAAGGCAATGGAATCAGAACTGACCAAGACCATTCACATTACTGAGGAGACAATGAGAGCTAAGCAGATGCTGGCGACGATGTCTCATGAGATAAGATCACCATTGTCAGGAGTAGTGGGGATGGCTGAGATACTTTCTACTACAAAGCTGGATAAAGAGCAGAGACAGTTGTTGAATGTCATGATCTCTTCTGGTGATTTGGTGCTTCAGTTTATTAATGATATTCTTGATCTCTCCAAGGTTGAATCAGGTGTGATGAAGTTAGAAGCTACCAAGTTTAGGCCAAGAGAAGTAGTGAAGCATGTGCTTCAGACAGCTGCTGCATCTCTGAAGAAAGAACTGACATTAGAAGGGAACATTGCAGATGAAGTCCCTATCTTGGTAGTTGGAGATGTTCTAAGGATCCGGCAGATTCTCACCAACTTGATCAGCAATGCTATCAAGTTTACACATCAAGGAAAGGTTGGCATCAAACTCAAAGTGATACCAGAACCATCCTTTGCAAGTGGTTTGGAGTTAAACGCAGACGCTGAAGAACAAAACGGTTTGACTGAGACTGAGACTTCGGTTTGGATTCGCTGCGATGTTTATGACACTGGAATTGGAATCCCGGAAAATGCTCTTCCTTGTTTGTTCAAGAAGTACATGCAAGCAAGCGCTGATCATGCCAGAAAATACGGTGGAACTGGTCTCGGTCTCGCCATTTGTAAACAGCTGGTAGAGCTAATGGGAGGTCAACTTACTGTGACGAGCCAAGTCGACTTAGGTTCAACGTTCACGTTCATATTACCCTATAAAGTTGCAACATCGAATGACCATTCGGATGATCAAGATGAGTTCTCTGATATGGTGGATCATCAACCCGAACCAGACGACTCAACCGAAGGATACTTCCAGTTTAAACCGCTTCTAGGATCTATATACTCTAATGGCGGACCGGTCATTGGCAATAACTTCTTACCTCATAAAGTTATGCTCCCTAGTCCTGTTAAGCTCATCAACGGTCAAAGCGAGGCTGTTCAGGTTGAAAACGGTGGTTATATGGATGGACCAAGACATGAAACCCGGTCTGGTCACTGTCCTGAATCACCTCATCAATATGAGAATGGGAATGGTCCATGTCCCTCTAAGGAAAGCGAGTCTTGTAGCAGTTCACAAGCTAGCTCAGAAATGGAGTCAGAGTTTACAGTTTCATCTCCTAGGGAAGAGAAGAAAACTGAGACAGAGGTCAAAAAGACATCAAAGCAGCCAAAGATTTTGCTTGTGGAAGATAATAAAATAAACATCATGGTTGCAAAGTCGATGATGAAACAATTAGGCTATACCTTTGATATTGCTAATAATGGAGTTGAAGCTATAACTGCTATTAACGGTTCTAGCTACGATTTGGTACTCATGGATGTGTGCATGCCTGTACTGGATGGTTTAAAAGCTACAAGACTGATCCGTTCGTACGAACAATCTGGGAACTGGGATGCTGCAGTAGAAGCTGGAGTAGACATCAAGACACTGGAGGATAAGCAACTCTGTGTGCGTTCCACAAACCGGCTGCCTATAATCGCGATGACGGCAAATACATTATCAGAGAGCTCAGAAGAATGTTATGCAAATGGTATGGACTCTTTTATTTCGAAACCTGTAACGTTGCAGAAACTAAAAGAGTGTCTAAAACAGTATCTGCATTGA

>BrHK5(Bra002095)-DNA

ATGCAAAGAGATAGCTTCTCAGTGAGTATTGAGAGTCTTCCTGATTCTCCAATGGGTCCAAGGAAGAAGAAGATCACTAAACTGTTCGATAATATGACTGAATGGGTTACCCCTTGGAGAAGTAACCCAGAGTCGCCAAGAGAGACAAGGATCATACGCGGAGATGTCGAGCAAGAACAGTTTCAATACGCAAGCAGTCACTGCTTGTCTTCTTACTACAGTGTCTTCGTCGTTCGCCTCGCTATAATGGTTAGTTTAGTTTTGTTTATGATCAATCTTTGGATAAGAAAGGTGGAAGCTTTATAAGTTGTTGTGTGTGTGTGTGTGTGTGTGCAGGTGATGCTAGCAATTCTAATAGGGCTTTTAACCATACTAACATGGCACTTCACAAGGATATACACAAAGCAATCGCTTCAAAACTTAGCAACTGGTCTTAGGTACGAGCTTCTTCAGCGTCCAATCTTAAGGATGTGGAGCGTTTTAAACACCACATCCGAGCTAACAACAGCTCAGGTCAAGCTCTCTGAATATGTCATTAAAAAATATGACAAGCCAACCACTCAAGAAGAACTTGTTGAGGTTTCCTTTTCTGTTCTTTATGTTCCTATGTTTTTATTTGAGATGCTTTTTATATTAATTGTTAAAACTTTTTTAATCAGATGTATCAAGCAATGAAAGATGTGACATGGGCTCTGTTTGCTAGTGCCAAAGCTCTAAATGCCATAACCATAAACTACAGAAACGGTTTTGTCCAAGCCTTCCACAGAGATCCAGCAAGCAGCAGCACCTTCTACATCTACTCCGATCTCAAAAACTATTCCATAAGCGAGACGACGGGCCTTGAAGATGTAAAAATGTCAATGGGCCACGGCTGGAACAACCAGACGATACACGGGAACATGACTGCCATTTGGTACCAGCAGCAGCTCGATCCAATCACAGGTGCAAAGCTAGGGAAGCCTCTTCAGATTCCACCTGATGATCTCATCAACATTGCGGGTATCTCACAGGTGCCTGATGGTGAAGCTTCTTGGCATGTGACGGTAAGCAAATACATGGACTCTCCGCTTCTCTCGGCGGCTTTGCCTGTCTTTGATGCTTCGAACAAGAGCATTGTGGCTGTTGTTGGGGTGACGACTTCGCTTTACAGCGTGGGGCAGTTGATGAGAGAGCTTGTGGAAGTACATGGTGGGCATATTTATCTGACATCTCAAGAAGGATACTTGCTTGCTACTTCCACAGATGGTCCTCTGCTAACGAATACATCAAGAGGGCCTAAGCTGATGAAAGCCGTTGATTCGAAGGAGTGGGCTATTAAGACAGGAGCTCATTGGTTAGAGAAGACTTATGGTAGTCACCTCCCTGACGTTGTTCATGCTGACAATGCAAGGCTTGGTGACCAACAGTATTACCTTGATTCATTTTATCTCAATCTCAAGAGACTTCCAATTGTAAGTTTTCTCGCTATGATGAATGGTAACAAACTGCACCGCAGTTAATAGTAACAAAAATTTATATATATACCTATGTATCTATATGTTTTTGTTACTATTAGGACCACACTGTAGTTGTTTCGCATATTGGCATTCAAATCTTCAAGAAGTCTAACTGTTTAACCTTTTTCGGTTTTACGCATTTTGCAGATAGGTGTTGTCATTATTCCGAGGAAGTTCATAATGGGAAAAGTGGATGAAAGAGCCTTCAAGACTTTGGTTATACTGATATCTGCTTCTGTCTGCATCTTCTTTATTGGATGTGTCTGCATTTTAATCCTCACAAATGGAGTTTCAAAGGAGATGAAACTAAGAGCAGAGCTGATAAGGCAGCTTGATGCAAGAAGAAGAGCTGAAGCTTCAAGCAACTATAAAAGCCAGTTTTTGGCAAATATGAGGTTTGTCAGATTTCTTTTGACTGTTTAGCATTCTTAATGCTAAATACTGAGATGAGTTTTTTGTTGGATACTAGCCATGAGTTGAGGACGCCTATGGCTGCTGTGATTGGATTGCTGGATATTCTGATATCAGATGATTGTCTTTCAAATGAGCAATATGCAACAGTCACTCAGATCAGGAAGTGCTCTACTGCACTCCTCCGGCTTCTCAACAACATTCTGGACTTGAGCAAGGTCAGAACTTACTTTTGATATAATTATTACATATGCAGATCTTTAAGTTAGGCTGTGAGTAAACATTATCTGTTTCAGATGTGTTTTTCCTACAAACTGACCTCAGATTTTCTCTTTTTCTGCCATAGGTTGAGTCTGGAAAACTTGTTCTGGAAGAAGCTGAGTTCGATTTGGGAAGAGAACTAGAAGGACTTGTTGACATGTTCTCAGTGCAGTGTATTAACCACAATGTAGAGACTGTTCTAGACCTCTCTGGTACGGCTGGTTATTATATTGTAGACAGCTAAACTACTTAATCTTCATTGAAATGAAACCCGGTTTATGATTTCTCTTTCCACAGATGACATGCCAACATTAGTCCGAGGGGATTCAGCAAGACTTGTTCAGATCTTTGCAAATCTTATAAGCAATTCTATAAAGTTTACAACAAGTAAGTTTTGTGACCATCTCTTTATCTTCTCTTGTTTTTTCTTTCATGACAGAGATGTGCATTGCTAATCTGAAAAAGATGAATACTTTGCAGCGGGCCACATTATTCTCCGTGGATGGTGCGACAGTGTGAGTTCTCTACATGATGAGATGAGCTTAACTGTTGACAGAAAGAAACCATGGGCTCCAGTGAAAACAAAGCTGGTGCATCACAGGAATCATTTGCACAAGTCTTGTAAGAACGAGAACAAAATTGTTCTTTGGTTTGAGGTTGATGACACTGGATGTGGTATGACACTTGAGTTTCTTAACTATAGTACTCTTTATTCCCAATCTCCCAGGTTCTGAAAATCAATTTTCCATATAAATCTTATCTGCAGGAATAGATCCAAGCAAATGGGACTCTGTGTTTGAGAGCTTTGAGCAGGCTGATCCTTCTACCACTCGGACGTAAGTGATCACAATAAAGTGCAGACAGTAAATGTATAAGCAAAACTAGGCCTGCGGATTTGAACTGAACCAGCTGAACCGAACCAAAATTTTTGGTTTTCAGTTCGGTTACGGTTTTGAGTTTGTGATTTTGAAAACGGTTAGGTTTTCGGTTTTAGAAAAATAAAAAAAAGAACTGATTATCTGAAATAACCTAACCAAAAATAATAGAATTTAACCAAAAATAAATCTAAAATATAACCGAAACGCAAAAAGAGAAAGAAAAATTGGTTATCTTCAGATAATTTTTTTAAAACCGAACCGAGCCTTTTTCGGTTTACTTCGGTGGAATTATTGCCGAACTGAAATAACCAAAACCAATAATATCTGACCCGAATAAACCGAAGTATCCGAAACCGCAGGGCTAAGCAAAGCTATGTTCTTATTTGGGTCTTAACCCTCACTGTTCTTGTACAGGCACGGAGGAACTGGACTTGGACTGTGTATCGTGCGAAACTTGGTTAGTCTAGTCTGGAACTTTAGAGTTTGTTGAGTCAAGCAAATCATCTGAAACCTTTGCTTTGTTGTAACTTTACAGGTAAACAAAATGGGTGGAGATATCAAAGTAGTACAGAAAAATGGGAGAGGGACTATAATGAGACTACACTTGATTCTGAGTATTCCAGACAACGCAGAACAAATCTACCAGCCAGAGTTCTCCCAGTACGGTCTTGTGGTACGTAAAGACATGGATTATGACCTATGGATGATAGATTAAAGCTTCTTTGTTTGTGAACTAAGGCATGGGTGTTCGGTTTGGTTTAGGTTCGGGTATTTTGGTTTTAAGTTTTTCGGTTCTAGAAATTTAAGAAAGGTTCAGTTATTTTGGTTTTGGTTCGGATACCAAAGTCGGGAATCGGCTAATATCCAAAAAAATCAGATTCATTCGGTTCTAGTTTTTGAGTAATTTCGGATATTATGGGTAAAAACGTCAGATAATACGGAGTTTTCAGGTAAAATATTGGATAAATCAGATAAAAATATTGGCTAATTCAGAAAATTTTCAAATATTTTGAATAAAAAATATTCAGATAATTTGAGTTTTGGACAATTTGATTTATAAATAGTATTTAAGATATTTAGAAACCAAATACAGTATAATTAATATTTTTAGGTATATAATCTATATTTTAAAATTTATAGTACCAATTTGATTTTCTGTTCCAGTTCGGTTATAGGTTTCCCGATTCCAAAGATATAGAATCTGCTCAGATAATTATGAAATTCAGGTTAAATGTGTCCATGCCTAAATGTGTTATTGGTTTTTAATGGAACAGGTTCTACTTTCAATGTCTGGAAGCACAGCAAGATCAATTCAATCAAAGTGGCTGCGCAAACACGGCATTGCAACAGTCGAAGCATCAGACTGGAACGTGCTGACACAGATCATCAGAGACCTCTTCGAGACGGGAAGCCGCGAAAACAGCTTTGACTCGCAGCACACCATCGCTGAATCACTGAGAGCAGAGCTTTCCAACACACAAGAGATCAGGAACCCTGTGTTCGTCATAGTGGTGGACATCGGAGTTCTCGATCTCACCACAGATATATGGAAGGAACAGCTAAACTACCTCGACAGATTCTCCAGCAAAGCGAAGTTCGCTTGGCTTTTGAAGCACGACACCTCCAACACGGTCAAAACAGAGCTCAGACGAAAAGGGCATGTCATGATGGTTAACAAACCCTTGTACAAGGCCAAGATGATTCAGATTCTTGAAGCTGTGATTAAAAACCGCAAGAGAGGCTTAAGAGGAAACGGTAGTGATGAATCTCACGACTGTTTAGAAATAGATCCAACACAGTTCGACACTTGCAGCTCTGATGACTCTTCAGAGAATACATCTCTGAAACCAACCGCATTGCCCTCTCCAGTGATTAAGAACTACCTTCTTGACATTACTAAAAGCAACGATGAGTCAACAAGTATGACTCAGAAGAAAAAAGAGGAAGAGGAAGAAGATTGGAAAGATCGAAGTAACAGGTTGTATTCAGGAGTTGCTTTGGATGGAAAGAATCAGAAGTCTCTTGAAGGTGTGAGGATCTTGCTAGCGGAAGATACACCGGTTCTTCAGCGAGTAGCCACCATAATGCTTGAGAAAATGGGAGCAACTGTAACTGCAGTTTGGGATGGACAACAAGCTGTTGATGCCCTCAACTACAAGTCTATCAATGCACAAGAACACAACAACTTGTCTGAAGAAGAAGAAACCAATCCTCAATCTGATACACCAAACTCCTCACCTTATGACTTGATCCTCATGGACTGCCAGGTACCACTAACACTTCACACTTTTTTTATATTTCATTCATACTCTCACACACATGCAAATACATCACGTTTTGTTCAACATGGCAGATGCCAAAAATGGATGGATATGAGGCAACAAAGGCGATAAGAAGAGCAGAGATTGGCACCAGTCTCCACGTACCAATCGTGGCGTTGACGGCTCACGCAATGTCTTCTGATGAAGCCAAGTGCCTGGAGGTAGGAATGGATGCTTATCTCACTAAGCCTATTGACCGAAAGCTTATGGTCTCTACCATTTTGTCACTTACAAAACCATCAACAGTCCTAACTTCGTTTTCTGACTGA

>BrHK5(Bra002095)-CDS

ATGCAAAGAGATAGCTTCTCAGTGAGTATTGAGAGTCTTCCTGATTCTCCAATGGGTCCAAGGAAGAAGAAGATCACTAAACTGTTCGATAATATGACTGAATGGGTTACCCCTTGGAGAAGTAACCCAGAGTCGCCAAGAGAGACAAGGATCATACGCGGAGATGTCGAGCAAGAACAGTTTCAATACGCAAGCAGTCACTGCTTGTCTTCTTACTACAGTGTCTTCGTCGTTCGCCTCGCTATAATGGTGATGCTAGCAATTCTAATAGGGCTTTTAACCATACTAACATGGCACTTCACAAGGATATACACAAAGCAATCGCTTCAAAACTTAGCAACTGGTCTTAGGTACGAGCTTCTTCAGCGTCCAATCTTAAGGATGTGGAGCGTTTTAAACACCACATCCGAGCTAACAACAGCTCAGGTCAAGCTCTCTGAATATGTCATTAAAAAATATGACAAGCCAACCACTCAAGAAGAACTTGTTGAGATGTATCAAGCAATGAAAGATGTGACATGGGCTCTGTTTGCTAGTGCCAAAGCTCTAAATGCCATAACCATAAACTACAGAAACGGTTTTGTCCAAGCCTTCCACAGAGATCCAGCAAGCAGCAGCACCTTCTACATCTACTCCGATCTCAAAAACTATTCCATAAGCGAGACGACGGGCCTTGAAGATGTAAAAATGTCAATGGGCCACGGCTGGAACAACCAGACGATACACGGGAACATGACTGCCATTTGGTACCAGCAGCAGCTCGATCCAATCACAGGTGCAAAGCTAGGGAAGCCTCTTCAGATTCCACCTGATGATCTCATCAACATTGCGGGTATCTCACAGGTGCCTGATGGTGAAGCTTCTTGGCATGTGACGGTAAGCAAATACATGGACTCTCCGCTTCTCTCGGCGGCTTTGCCTGTCTTTGATGCTTCGAACAAGAGCATTGTGGCTGTTGTTGGGGTGACGACTTCGCTTTACAGCGTGGGGCAGTTGATGAGAGAGCTTGTGGAAGTACATGGTGGGCATATTTATCTGACATCTCAAGAAGGATACTTGCTTGCTACTTCCACAGATGGTCCTCTGCTAACGAATACATCAAGAGGGCCTAAGCTGATGAAAGCCGTTGATTCGAAGGAGTGGGCTATTAAGACAGGAGCTCATTGGTTAGAGAAGACTTATGGTAGTCACCTCCCTGACGTTGTTCATGCTGACAATGCAAGGCTTGGTGACCAACAGTATTACCTTGATTCATTTTATCTCAATCTCAAGAGACTTCCAATTATAGGTGTTGTCATTATTCCGAGGAAGTTCATAATGGGAAAAGTGGATGAAAGAGCCTTCAAGACTTTGGTTATACTGATATCTGCTTCTGTCTGCATCTTCTTTATTGGATGTGTCTGCATTTTAATCCTCACAAATGGAGTTTCAAAGGAGATGAAACTAAGAGCAGAGCTGATAAGGCAGCTTGATGCAAGAAGAAGAGCTGAAGCTTCAAGCAACTATAAAAGCCAGTTTTTGGCAAATATGAGCCATGAGTTGAGGACGCCTATGGCTGCTGTGATTGGATTGCTGGATATTCTGATATCAGATGATTGTCTTTCAAATGAGCAATATGCAACAGTCACTCAGATCAGGAAGTGCTCTACTGCACTCCTCCGGCTTCTCAACAACATTCTGGACTTGAGCAAGGTTGAGTCTGGAAAACTTGTTCTGGAAGAAGCTGAGTTCGATTTGGGAAGAGAACTAGAAGGACTTGTTGACATGTTCTCAGTGCAGTGTATTAACCACAATGTAGAGACTGTTCTAGACCTCTCTGATGACATGCCAACATTAGTCCGAGGGGATTCAGCAAGACTTGTTCAGATCTTTGCAAATCTTATAAGCAATTCTATAAAGTTTACAACAACGGGCCACATTATTCTCCGTGGATGGTGCGACAGTGTGAGTTCTCTACATGATGAGATGAGCTTAACTGTTGACAGAAAGAAACCATGGGCTCCAGTGAAAACAAAGCTGGTGCATCACAGGAATCATTTGCACAAGTCTTGTAAGAACGAGAACAAAATTGTTCTTTGGTTTGAGGTTGATGACACTGGATGTGGAATAGATCCAAGCAAATGGGACTCTGTGTTTGAGAGCTTTGAGCAGGCTGATCCTTCTACCACTCGGACGCACGGAGGAACTGGACTTGGACTGTGTATCGTGCGAAACTTGGTAAACAAAATGGGTGGAGATATCAAAGTAGTACAGAAAAATGGGAGAGGGACTATAATGAGACTACACTTGATTCTGAGTATTCCAGACAACGCAGAACAAATCTACCAGCCAGAGTTCTCCCAGTACGGTCTTGTGGTTCTACTTTCAATGTCTGGAAGCACAGCAAGATCAATTCAATCAAAGTGGCTGCGCAAACACGGCATTGCAACAGTCGAAGCATCAGACTGGAACGTGCTGACACAGATCATCAGAGACCTCTTCGAGACGGGAAGCCGCGAAAACAGCTTTGACTCGCAGCACACCATCGCTGAATCACTGAGAGCAGAGCTTTCCAACACACAAGAGATCAGGAACCCTGTGTTCGTCATAGTGGTGGACATCGGAGTTCTCGATCTCACCACAGATATATGGAAGGAACAGCTAAACTACCTCGACAGATTCTCCAGCAAAGCGAAGTTCGCTTGGCTTTTGAAGCACGACACCTCCAACACGGTCAAAACAGAGCTCAGACGAAAAGGGCATGTCATGATGGTTAACAAACCCTTGTACAAGGCCAAGATGATTCAGATTCTTGAAGCTGTGATTAAAAACCGCAAGAGAGGCTTAAGAGGAAACGGTAGTGATGAATCTCACGACTGTTTAGAAATAGATCCAACACAGTTCGACACTTGCAGCTCTGATGACTCTTCAGAGAATACATCTCTGAAACCAACCGCATTGCCCTCTCCAGTGATTAAGAACTACCTTCTTGACATTACTAAAAGCAACGATGAGTCAACAAGTATGACTCAGAAGAAAAAAGAGGAAGAGGAAGAAGATTGGAAAGATCGAAGTAACAGGTTGTATTCAGGAGTTGCTTTGGATGGAAAGAATCAGAAGTCTCTTGAAGGTGTGAGGATCTTGCTAGCGGAAGATACACCGGTTCTTCAGCGAGTAGCCACCATAATGCTTGAGAAAATGGGAGCAACTGTAACTGCAGTTTGGGATGGACAACAAGCTGTTGATGCCCTCAACTACAAGTCTATCAATGCACAAGAACACAACAACTTGTCTGAAGAAGAAGAAACCAATCCTCAATCTGATACACCAAACTCCTCACCTTATGACTTGATCCTCATGGACTGCCAGATGCCAAAAATGGATGGATATGAGGCAACAAAGGCGATAAGAAGAGCAGAGATTGGCACCAGTCTCCACGTACCAATCGTGGCGTTGACGGCTCACGCAATGTCTTCTGATGAAGCCAAGTGCCTGGAGGTAGGAATGGATGCTTATCTCACTAAGCCTATTGACCGAAAGCTTATGGTCTCTACCATTTTGTCACTTACAAAACCATCAACAGTCCTAACTTCGTTTTCTGACTGA

>BrHK6(Bra035381)-DNA

ATGTCTATAACTCGTGAACTCACTTCAAAGAAGGCAGAGGAATCCAAGGGTGGTGTGAAGTGGATAAAAAAGCCTCTCTTCTTCATGATAATGTGTTGCTTAACCACCTCTTTGGTGATTGTTCTTTTGATGTCCTCGGGGAAGGAGGAGGAGACCGGTTCTTGTAATGGGGAAGCTAGAGTGTTGTACAGACATCAAAACGTCACTAGGAGTGAGATTCATGACTTGGTCTCTTTGTTCTCTGATTCAGATCAGGTAAATCATCTGAATTGACTTTGGTTTAAAATTGTGCTTCTTGAAAGCTTTATATAAAATAAAATGCATCAGATGTGTCAACTTACCACTCTCTGCATCTGTTTTTTTCAGGTAACATCCTTCGAATGTCGAAAAGAATCCATCCCTGGAATGTGGGCAAACTATGGTATTACATGTTCTCTGAATATGCGTTTGGAGAAAGAGGAGACTCAATGCGGCCATGACAATTCAAAGAGCTTAATTCCCTGGAATCTTATTAATCCATCAGGCTTACAACAGGTTAGATAACATTTGTTTGTAGAAAAATGCCTTCCCAAAACGTTATTTTGGTAATATTATATTTTGGTTTGTGTGTAGAGTTTACTGCACCCCGAAAACCACAGAGAAGGGCTGGATTGGGATTTGCCATCTTATTTAAGAAATACATGGTGGTGCCTTATCTTTGGTCTACTCGTGTGCCATAAGATTTTTGTTTCTCATTCTAAACCACCGAATGAGATAGAAGGAAAAACAAATCTGCAAGATTCTTTAGCTCAAAAGCAGCAGCAGCGAGCTTGTAGAGGGGCTGGGAAATGGAGGAAGAACATCCTTCTCCTCGGTGTCATCGCAGGAGTTTCCATGTCGGTTTGGTGGTTCTGGGACTCCAATCAGAAGATCATACTGCAACGGAGGGAAACTTTAGAAAACATGTGTGATGAGCGAGCCCGTGTGTTACAAGATCAGTTCAATGTTAGCCTGAACCATGTTCACGCCTTATCTATTCTTGTATCCACCTTTCACCATGGTAAAACCCCTTCTGCCATTGATCAGGTGATGTGTTTTTTTTCTTTCTCTTACCGCTATTTACATTTCATGTTTTTGCAATTTGGAAGATGTTAAAACCATAAACTTCTTCTTTGTAGAAAACATTTGGTGAATATACTGAGAGAACAAACTTCGAGAGGCCCCTTACCAGTGGTGTTGCGTATGCTTTGAAAGTCCCACACTCCAAAAGAGAGCAATTTGAGAAGGATCATGGGTGGACAATAAAGAAAATGGAAACTGAGGACCAGACACTTGTCCAAGATTGTGTTCCTGAAAATTTTGATCCAGCACCTATTCAAGACGAGTACGCTCCAGTCATATTTGCTCAAGAAACTGTTTCCCATATTGTATCGGTCGACATGATGTCTGGACAAGTCAGTAACCATGTTCCTAAAATGTTTCCTTTGCCTTTTTCCAAACCAATGTTGGTTAAATAAGAAATTCTCAAAATCTCTCTCTCTATTTATTTTGCAGGAAGACCGTGAAAACATCTTGAGGGCAAGGGCATCAGGAAAGGGTGTATTAACATCTCCGTTTAAGCTTCTGAAGTCAAATCATCTCGGCGTTGTCTTGACCTTTGCTGTCTATGACACGGACCTACCCGCTGATGCTACAGAAGAACAGCGTGTTGAAGCAACTATTGGGTACTCTTTTTTTTTTTTTACTTTTCTCAATTAAAATGGTTGTGCTGATGTACCTTACTTAATAAGATACACATGAAACAGATACCTTGGTGCATCATATGATATGCCATCGCTGGTGGAGAAACTTCTTCACCAACTTGCCAGCAAACAGACAATTGTGGTGGATGTTTATGATACAAGTAACGCCTCGGGTCTAATTAAAATGTACGGAACTGAAATTGGGGATACAAGTGAAGAGTACATAAGTAGCCTTGATTTTGGTGATCCATCCAGAAAACATGAGATGCGCTGCAGGTTTTACTCTAATGACATCTTTCTGTAAAAGCACTCTCTTCTGTCTTTTCTTTAAGTATGAAAATGGCAATATTTTTCTTTTATACAGGTTTAAAGATAAACTTCCCATCCCTTGGACAGCGATAATACCGTCAGGCTTAGTTCTGATTATTACTTTTCTGGTTGGTTATATCTTCCATGATGCCATCAGTCGAATTGCCATAGTTGAAGAGGACTGTCAGAAGATGATGAAACTGAAAGCTCGCGCTGAGGCTGCTGACATTGCCAAATCACAGGTGAATTTTCTCAATCGAATACTCACAAAAGCTTCTTGTCACTGATATGCTTCTTTGTTTATGCAGTTTCTGGCAACTGTTTCTCATGAGATACGGACTCCAATGAATGGTGTTCTAGGTAAATCTTTTTACTGTCTCCTTTGCCATTGCATTTTTTTGTGTGGAATAGCAGCTGAAAGGTTTGATAAGCATATGTTGTAACAGGAATGCTGAAAATGCTTATGGACACTGATCTTGACGCGAAACAAATGGACTATGCACAAACTGCTCATGGGAGTGGGAAGGATCTTATATCACTAATAAACGAGGTTCTTGATCAGGCAAAGATTGAATCAGGAAGGCTTGAGCTTGAAAATGTGCCTTTTGATATGCGCTTAGTTCTGGATAATGTTTCATCTCTACTCTCTGGCAAGGCAAATGAAAAAGGAATTGAGGTACAATATAAACCGCATGACCTTCTGCTTAAATGTTTTCATATATAGCAAACAAATGTTGGTTTATTGTTACAGTTGGCAGTTTATGTTTCTAGTCAAGTTCCTGAAGTTGTAGTAGGTGATCCGAGTCGGTTCAGGCAAATAATTACAAACCTGGTTGGAAACTCAATCAAGGTGATCCAGTTTCCTACTTCTATACAAAAGAAAAAAGTCTTCCTCCACTACGAATAGTACTCATTTGTTCTTTCTGTAACAAACAATATAATAGTTCACACGGGAGAAGGGACACATATTTATCTCGGTGCACCTTGCAGAAGAGGTGAGGGAGAGTGTTAATATCAAAGATGCAGTACTAAGACAGAGGTTAGCTGTGGGAAGCAGTGCGTCCGGTGAGACAGTAAGCGGGTTTCCTGCTGTGAATGCATGGGGAAGCTGGGAGAGTTTCAAGACAATTTACAGCACTGAGAATCATACTTGTGATAGAATCATCAAATTGCTAGTTACAGTGGAGGACACAGGGGTGGGCATACCTTTGGATGCACAAGGAAGAATATTCACTCCTTTTATGCAAGCTGACAGCTCCACATCTCGTACTTATGGTGGGACCGGCATAGGTTTGAGCATAAGCAAGCGTTTGGTTGAACTAATGCAAGGAGAAATGGGGTTTGTGAGCAGGCCTGGGATAGGGAGTACTTTTTCATTTACGGGAGTTTTTGGGAAAGCAGAAACAACAAGTTCGTTTAATACTAAGTTTGATCTAGCTATTCAGGAGTTCAAAGGACTGAGGGCATTAGTTATTGATAGCAGAAACATTCGAGCAGAGGTGACCAGATACCATCTGCAGAGATTGGGAATATGTGCAGACATTGTTTCAAGTCTGAGAACAGCATGCACATGTGTCAAGTACTTCACATACATTTTCTTTACTTCTAGCTTTGCTATGTTAATATGACATGAAATGTTTATTCCATTGCTATATATATATAGTTATGTTTCTGTGTTTTTTTTTGCAGCAAATTAGGAGGTTTGGCGATGGTTCTAATCGATAAAGACGCGTGGAACAAGAAAGACTTTGAACTACTTGATGGGTTGCTTAATTGTAGCAAAGAAACCACCACAAGACCGACAAAGATTTTACTTTTGGCAACCTCTGCAACTCTTGTAGAGCGCAGTGAGATGAAGTCTTCTGGTCTGGTGGACGAGGTGGTAATAAAGCCCCTTCGGATGAGTGTCCTAATATGCTCTTTGCAAGAAACCCTGGTCAATGGTAAGAAGAGGCAACCAAGCAGAAAGCGAACGAATCTTGGACACTTACTGAGAGAAAAGAGGATTCTGGTTGTGGACGATAATCTGGTGAACAGAAGAGTCGCTGAAGGCGCGCTTAAGAAATATGGAGCTATCGTTACATGCGTTGAGAGTGGGAAGGCTGCATTGGCAATGCTCAAGCCGCCTCACAACTTTGATGCTTGCTTCATGGACCTCCAGATGCCTGAGATGGACGGGTAGCTAAGCTAATCACTTTAGGGTCTCATCATCCTCATTCAGCTTTTGATAAATTTTATATCATCTTTTTTTTTTTTGTGTACAGATTTGAAGCAACGAGGAGAGTCCGTGATCTAGAGAAGAAGAAAATAGTTTCTGGTGAAATGTACAGTAGCTGGCACGTTCCAATATTAGCAATGACAGCTGATGTGATTCAGGCTACTAACGAAGAATGCGTCAAATGTGGGATGGATGGTTATGTATCGAAACCGTTTGAAGAAGAAGCTCTCTATGCAGCTGTAGCAAAGTTCTTTGAATCTGATGGTTAA

>BrHK6(Bra035381)-CDS

ATGTCTATAACTCGTGAACTCACTTCAAAGAAGGCAGAGGAATCCAAGGGTGGTGTGAAGTGGATAAAAAAGCCTCTCTTCTTCATGATAATGTGTTGCTTAACCACCTCTTTGGTGATTGTTCTTTTGATGTCCTCGGGGAAGGAGGAGGAGACCGGTTCTTGTAATGGGGAAGCTAGAGTGTTGTACAGACATCAAAACGTCACTAGGAGTGAGATTCATGACTTGGTCTCTTTGTTCTCTGATTCAGATCAGGTAACATCCTTCGAATGTCGAAAAGAATCCATCCCTGGAATGTGGGCAAACTATGGTATTACATGTTCTCTGAATATGCGTTTGGAGAAAGAGGAGACTCAATGCGGCCATGACAATTCAAAGAGCTTAATTCCCTGGAATCTTATTAATCCATCAGGCTTACAACAGAGTTTACTGCACCCCGAAAACCACAGAGAAGGGCTGGATTGGGATTTGCCATCTTATTTAAGAAATACATGGTGGTGCCTTATCTTTGGTCTACTCGTGTGCCATAAGATTTTTGTTTCTCATTCTAAACCACCGAATGAGATAGAAGGAAAAACAAATCTGCAAGATTCTTTAGCTCAAAAGCAGCAGCAGCGAGCTTGTAGAGGGGCTGGGAAATGGAGGAAGAACATCCTTCTCCTCGGTGTCATCGCAGGAGTTTCCATGTCGGTTTGGTGGTTCTGGGACTCCAATCAGAAGATCATACTGCAACGGAGGGAAACTTTAGAAAACATGTGTGATGAGCGAGCCCGTGTGTTACAAGATCAGTTCAATGTTAGCCTGAACCATGTTCACGCCTTATCTATTCTTGTATCCACCTTTCACCATGGTAAAACCCCTTCTGCCATTGATCAGAAAACATTTGGTGAATATACTGAGAGAACAAACTTCGAGAGGCCCCTTACCAGTGGTGTTGCGTATGCTTTGAAAGTCCCACACTCCAAAAGAGAGCAATTTGAGAAGGATCATGGGTGGACAATAAAGAAAATGGAAACTGAGGACCAGACACTTGTCCAAGATTGTGTTCCTGAAAATTTTGATCCAGCACCTATTCAAGACGAGTACGCTCCAGTCATATTTGCTCAAGAAACTGTTTCCCATATTGTATCGGTCGACATGATGTCTGGACAAGAAGACCGTGAAAACATCTTGAGGGCAAGGGCATCAGGAAAGGGTGTATTAACATCTCCGTTTAAGCTTCTGAAGTCAAATCATCTCGGCGTTGTCTTGACCTTTGCTGTCTATGACACGGACCTACCCGCTGATGCTACAGAAGAACAGCGTGTTGAAGCAACTATTGGATACCTTGGTGCATCATATGATATGCCATCGCTGGTGGAGAAACTTCTTCACCAACTTGCCAGCAAACAGACAATTGTGGTGGATGTTTATGATACAAGTAACGCCTCGGGTCTAATTAAAATGTACGGAACTGAAATTGGGGATACAAGTGAAGAGTACATAAGTAGCCTTGATTTTGGTGATCCATCCAGAAAACATGAGATGCGCTGCAGGTTTAAAGATAAACTTCCCATCCCTTGGACAGCGATAATACCGTCAGGCTTAGTTCTGATTATTACTTTTCTGGTTGGTTATATCTTCCATGATGCCATCAGTCGAATTGCCATAGTTGAAGAGGACTGTCAGAAGATGATGAAACTGAAAGCTCGCGCTGAGGCTGCTGACATTGCCAAATCACAGTTTCTGGCAACTGTTTCTCATGAGATACGGACTCCAATGAATGGTGTTCTAGGAATGCTGAAAATGCTTATGGACACTGATCTTGACGCGAAACAAATGGACTATGCACAAACTGCTCATGGGAGTGGGAAGGATCTTATATCACTAATAAACGAGGTTCTTGATCAGGCAAAGATTGAATCAGGAAGGCTTGAGCTTGAAAATGTGCCTTTTGATATGCGCTTAGTTCTGGATAATGTTTCATCTCTACTCTCTGGCAAGGCAAATGAAAAAGGAATTGAGTTGGCAGTTTATGTTTCTAGTCAAGTTCCTGAAGTTGTAGTAGGTGATCCGAGTCGGTTCAGGCAAATAATTACAAACCTGGTTGGAAACTCAATCAAGTTCACACGGGAGAAGGGACACATATTTATCTCGGTGCACCTTGCAGAAGAGGTGAGGGAGAGTGTTAATATCAAAGATGCAGTACTAAGACAGAGGTTAGCTGTGGGAAGCAGTGCGTCCGGTGAGACAGTAAGCGGGTTTCCTGCTGTGAATGCATGGGGAAGCTGGGAGAGTTTCAAGACAATTTACAGCACTGAGAATCATACTTGTGATAGAATCATCAAATTGCTAGTTACAGTGGAGGACACAGGGGTGGGCATACCTTTGGATGCACAAGGAAGAATATTCACTCCTTTTATGCAAGCTGACAGCTCCACATCTCGTACTTATGGTGGGACCGGCATAGGTTTGAGCATAAGCAAGCGTTTGGTTGAACTAATGCAAGGAGAAATGGGGTTTGTGAGCAGGCCTGGGATAGGGAGTACTTTTTCATTTACGGGAGTTTTTGGGAAAGCAGAAACAACAAGTTCGTTTAATACTAAGTTTGATCTAGCTATTCAGGAGTTCAAAGGACTGAGGGCATTAGTTATTGATAGCAGAAACATTCGAGCAGAGGTGACCAGATACCATCTGCAGAGATTGGGAATATGTGCAGACATTGTTTCAAGTCTGAGAACAGCATGCACATGTGTCAACAAATTAGGAGGTTTGGCGATGGTTCTAATCGATAAAGACGCGTGGAACAAGAAAGACTTTGAACTACTTGATGGGTTGCTTAATTGTAGCAAAGAAACCACCACAAGACCGACAAAGATTTTACTTTTGGCAACCTCTGCAACTCTTGTAGAGCGCAGTGAGATGAAGTCTTCTGGTCTGGTGGACGAGGTGGTAATAAAGCCCCTTCGGATGAGTGTCCTAATATGCTCTTTGCAAGAAACCCTGGTCAATGGTAAGAAGAGGCAACCAAGCAGAAAGCGAACGAATCTTGGACACTTACTGAGAGAAAAGAGGATTCTGGTTGTGGACGATAATCTGGTGAACAGAAGAGTCGCTGAAGGCGCGCTTAAGAAATATGGAGCTATCGTTACATGCGTTGAGAGTGGGAAGGCTGCATTGGCAATGCTCAAGCCGCCTCACAACTTTGATGCTTGCTTCATGGACCTCCAGATGCCTGAGATGGACGGATTTGAAGCAACGAGGAGAGTCCGTGATCTAGAGAAGAAGAAAATAGTTTCTGGTGAAATGTACAGTAGCTGGCACGTTCCAATATTAGCAATGACAGCTGATGTGATTCAGGCTACTAACGAAGAATGCGTCAAATGTGGGATGGATGGTTATGTATCGAAACCGTTTGAAGAAGAAGCTCTCTATGCAGCTGTAGCAAAGTTCTTTGAATCTGATGGTTAA

>BrHK7(Bra013186)-DNA

ATGTCTATAGCTTGTGAGCTCTCGAATCCCAATTTAAAGAAGACAAAAGCAGAAAAACGGATACCAACCAAGATTCTGCTTATCCGGGTTTTATGTGGCTTAGTTGTTCTCTGGCTCTGCTTAAGCTTAAGCTTAGGCTTCTTATGTATATGCAAGAAGAAAGAGGCTGCTGCTGCTGCTGATGACTCTTCTTCTTCTGCTAAAGGGATGTTGTTCAGGAATCAGAGCAGAAGTGAGATTGATGCTATGCTTTCTCTCTTCTTTGATTCAAATCAGGTTTCCCACTACTTCTTTCTTCTTTTTATTATTGTTTGACAATTTAATTTACTTAAAGCTTTTGAATAATTGGAGAACAGGTAACATCTTTTGAATGTCGCAAGGAGAATGGTGGTATTACATGTTCCTTGTCAACACGTTCCGAGAAAGGAGACGAGGAGGAGGAGGAGGCTAAGAGACATGTTGTTGCAGAGCTTATGTCATCATCTGAGAATGAAGAAGAAGGAGGTGTGCTGCATCAGGTTGTGTTGTTTTATGTAATGAACAAATGTCATTGGTGGTTGGTCCTTTGTGTACTACTAGTGGGCGGCGGCCGTATGATATTTGCAAGAAAAGAAGTTTCTTCTTTAGTACAAGACAAGCAGCAGCAGCAGCAGCAATGCAAAACAGCTGGGAAGTGGAGGAAGAACATGCTTCTACTCGGCATCATCGCGGGAGTTTCTTTGTCTGTTTTATGGTTTTGGGATACAAACGAGAAGATCTTGTTCCAAAGGAAAGAGACGTTAACCAACATGTGTGAGGAACGAGCTCGGGTGTTGCAGGACCAGTTCAATGTTAGCATGAACCATGTCCACGCCTTGTCCATTCTCGTCTCTACCTTTCACCACGGAAAAACCCCTTCTGCCATTGATCAGGTAAACTTAGAGTCCGAATGGTAACATGTGGAACAACTGCGGTGCGGTTCTAATAGTAACAAAAACGTATAGATACATGTGTAAATCTAGAGATAATAGTAACAAAAACGTATAGATACATGTGTAAATCTAGAGATTTTTTTTAATCTAGAGATTTTTTTTTAACCTAGAGATTTTTTTTAATGTTAATTGCGGTGCGGTTTAACAGTAAAAAAAATGTATATATACATGTGTAGATCTAGAGTTTTTGGTTAATGTTAACTGTGGTGCGGTTCTAATAGTAACAAAAACGTATAGATACATGTGTAAATCTAGAGATTTTTGTTACTGTTAACTGCGATGGTGTTTTAATAGTAACAAAAACGTATAGATACATGTGTAAATCTAGAGATTTTTGTTACTGTTAACTGCGATGATGTTTTAATAGTAAAAAAAACATAAATACATGTGTATATCTAGAGATTTTTGTTAATGTTAACTGGCATGTAGTTCTAATAGTAAAAAAAACGTATAGATACATGCGTAAATCTATAGTTTTTTTTGTTAATGTTAACTGTGGTGTGGTTCTAATAGTAACAAAACGTATAGATATATGTGTATATCTAGAGATTTTTTGTTAATGTTAATGTGCGGTTCCAATAGTAGCAAAAATGTATAGATACACATGTAAATCTAGAGATTTTTGTTAATGCTAATTGTCGTGCCGTTCGGTTAGTTATAAAATTCCATTCTTAAGATTCTTAACTGTTGATATTTTTTTTTTTCAGAAAACGTTTGGTGAGTATACAGAGAGAACTAATTTCGAAAGACCTCTCACGAGCGGTGTTGCTTACGCATTGAAAGTCACACACTCTGAAAGAGAGAAATTCGAGAAGGAACATGGATGGTCAATAAAGAAAATGGACTTTGAAGACCAGACTCTCGTCCAAGGCTTCGATCCAGCTCCTGTTCAAGACGAATACGCACCCGTTATCTTCGCTCAAGAAACCGTCTCTCATATTGTCTCTGTCGACATGATGTCTGGAAAAGTAAATCCCACTTAAGCTTCTTGTTGTCATAGTCTCAGACAGTTGATTAAACCAATCTTTACTCTTTTGCTTTGTAGGAAGACAGAGAAAACATCTTGAGAGCGAGAGCCTTAGGGAAAGGAGTGTTAACATCACCTTTCCAGCTTTTGAAATCAAACCATCTCGGCGTTATATTGACTTTCGCTGTGTACAACACCAACCTACCGCATGACGCTACTGAAGAAGAGAGGATCCAGGCAACAATTGGGTACCTTGGCGCATCGTACGATATGCCTTCACTAGTGGAGAAGCTTCTTCAACAGCTTGCGAGTAAACAGACCATATCTGTAAACGTTTACGACACGACTAACGCGTCGTCTGTCATTAAAATGTATGGCTCGGAGGTTGGTGATATGAGCGAAGAGCACATTAGTAGCCTTGATTTTGGTGATCCCTTTAGGAAGCATGAGATGCACTGCAGGTTTGGTTTCGTTCAGTCTCTCTCTCTACACTCTCTATGATCATTCACTCAGAGATAATGTGGTTTTTTTTTTTTTTAAACAGGTTTACACAAAAACCACCGATTCCTTGGTTAGCTATAATGCCACCAGGCTTCGCATTGGTTATCACGCTTCTTCTTGGTTATATCTTTAATGAAGCCATTAATCGAATTGCTACTGTTGAAGAGGACTATCAGAAAATGATGGAGCTTAAGGCTCGTGCTGAGGCTGCTGATGTGGCAAAGTCACAAGTAACTTTCTTGTTCTTTTTTTGTTTTTTACTTATGCCTAATCAAAGTTCTTATGTTTTGATCAAAATGTTTATTTGATGATTGGCAGTTTCTAGCCACTGTTTCTCATGAGATACGTACCCCAATGGTTGGTGTTCTAGGTAAAACGAGGAAGAAACTTAATTATGATTATTACCGTTTTTTGGATTTTTGTTTTCTCTGGAAGTGAAACATGTAAGGGATTGTTTTTTTGACAGGGATGCTGAAGTTGCTGATGGACACTGATCTTGACGCAAAACAATTGGACTTTGCTGAAACGGCTCATGGCAGTGGGAAAGATCTAATATCGCTGATAAACGAGGTTCTTGATCAGGCGAAGATTGAGTCAGGAAGACTTGAGCTTGAGAACGTGCCTTTTGATCTACGGTTCCTTCTGGATAATGTTTCATCCCTCCTCTCTGGCAAGGCTGCTGAAAAAGGAATCGAGGTATACACAACATCAACACAAAGTTTGGTCTTATTTCATTCAACTCTTTTTCTAATGAATGTTTTCTTTCTTTTGTGTCAAGAACAGTTGGCTGTTTATGTTTCTAGCAATGTTCCAGCTGTTATAGTTGGTGATCCGGGTCGGTTCCGACAGATAATCACAAACCTTGTTGGAAACTCAATCAAGGTATTTACTGCTACCTAGCTCTAGGCATTCGGGTCCAGTTCCGGCTCGGTTCTTCAGTTCTAGAGGTTTAGAATCCGTTCAGTTACTTAGAAAATTTGGTTCCGGTTCGTTCGGTTCTCAATTTGGTTCGGGTAATAAAGTTAGGAACCAGCTAATATCCAAATAATTTCGGATTGTTCTGATTTTTTTCCGGTTAATTCGGACTAAAAAATTGAAAAATTCGGATTTAAAAAAAAAAATTGGGGTTTCTACCCCAGTATAACTTTAAGAACCAACATATAAGTCTCTCCTGATGTTTTGTTCTTTTTCCATAAATAAAGTTCACACAGGAAAAAGGACACATATTCATCTCGGTGCACCTAACCAATGAGGTGAGAGAACCGTTTGAAACAGAAGATGAAATACTAAAACAAAGACTTGGTTCGGATGAGACATCATGTAACACGCTTAGCGGGTATCCAGCTGTGAATGCAAGTGGAAGCTGGAGGAACTTCAAGACATTTCAGGACCATAGTTGTGATAAAACTGAGTTGCTTGTTACTGTGGAGGACACAGGGATTGGCATTCCTGTTGATGCACAGTCTCGGATCTTCACTCCGTTTATGCAAGCCGACAGTTCGACATCACGGACTTACGGTGGGACAGGGATAGGTTTAAGCATAAGCAAGCGTTTGGTTGAGCTCATGCAAGGAGAGATTGGGTTTGTAAGCAAGCTTGGTGTCGGCACTACTTTCTCATTTACCGGAGTTTTTGGAGAACGGGAAAGGGATTCATCAGTCACTGCCTTGGAGCTATTTGATCAATCTATTCAGGAGTTTCAAGGATTGAAAGCATTGGTTATTGATAGCAGAAACATTCGAGCCGAGGTTACTCGTTACCATCTTCAGAGAGTTGGTGTATCTTCTGTAGACATTGCTTCAAGTCTTGCAACTGCATCTTCTTCCTGTGCCAGGTACTTTTGCTTTCCATTTCTTGGTGGAAGTGTCTTAAACTCTCGGTACCGTATGCCTTTTAGGATTAGGAAAAGTACTTTTAATGGGATAAGTTACTATAAATACGGGTCTAAGAGATTGTAAATTATTTGTTTTCACATAATCAATAAATAAGTTTCTTTGCTTTCTTCAAAGTTAATTCGCGTTTGTTGCTTATTAACGACACTTGTGATGAGATTACTTCATGTGTTACTCAAAAATTTTAAAATTGTTTGGCAGTAAATTAGTGAACTTGGATTTGATTCTAATCGACAAAGATGCATGGAACAAGGAAGAGTATGTAGCATTTAACAGTGGTCAAGAGCCCTCTACGAGACACCCAAAGATTATACTTTTGGCTACCTTTACAACTCCAGCAGAGCTCAGTGAGATCAAATCAACTGGTTTGGTAGACGAGGTGGTGATGAAACCGCTTCGAATGAGCGTCTTGATATGCTGCTTGCAAGAAACCGTGGGAAGTGGTGGCAAGAAGAGGCAGACAAATAGAAAACCAAAGAATCTTGGGAACTTGCTTAGAGGAAAACATATTCTGGTGGTAGACGATACGATGGTAAACAGAAGAGTGGCAGATGCAGTGCTAAGGAAATATGGAGCTCTTGTGACTTGCGTTGACAGTGGCAAAGCTGCAGTTGCAATGCTTAAGCCGCCTCATGATTTTGACGCATGCTTCATGGATCTGCAGATGCCTGAAATGGATGGGTAAAGTCTTTTGCTTTTAACGGTTGTGGATATGATGATGAATGCAAAAACTCATGGGTAATCTCTTTTTTGTTTACTGGTGCAGATTTGAAGCAACGAGGAGAGTACGTGGTCTGGAAGAAGAGATGAACAAGAAAGAAGAGTCAAGGAAGTGGCACACACCAATATTAGCTATGACTGCTGATGTGATCCAGGCAACAAATGAAAAATGCATCAAGTGTGGGATGGATGGTTTTGTATCGAAACCGTTTGAAGAAGAAGAGCTTTATTCATCAGTGGCAAGATTCTTCGAAAAATAA

>BrHK7(Bra013186)-CDS

ATGTCTATAGCTTGTGAGCTCTCGAATCCCAATTTAAAGAAGACAAAAGCAGAAAAACGGATACCAACCAAGATTCTGCTTATCCGGGTTTTATGTGGCTTAGTTGTTCTCTGGCTCTGCTTAAGCTTAAGCTTAGGCTTCTTATGTATATGCAAGAAGAAAGAGGCTGCTGCTGCTGCTGATGACTCTTCTTCTTCTGCTAAAGGGATGTTGTTCAGGAATCAGAGCAGAAGTGAGATTGATGCTATGCTTTCTCTCTTCTTTGATTCAAATCAGGTAACATCTTTTGAATGTCGCAAGGAGAATGGTGGTATTACATGTTCCTTGTCAACACGTTCCGAGAAAGGAGACGAGGAGGAGGAGGAGGCTAAGAGACATGTTGTTGCAGAGCTTATGTCATCATCTGAGAATGAAGAAGAAGGAGTGGGCGGCGGCCGTATGATATTTGCAAGAAAAGAAGTTTCTTCTTTAGTACAAGACAAGCAGCAGCAGCAGCAGCAATGCAAAACAGCTGGGAAGTGGAGGAAGAACATGCTTCTACTCGGCATCATCGCGGGAGTTTCTTTGTCTGTTTTATGGTTTTGGGATACAAACGAGAAGATCTTGTTCCAAAGGAAAGAGACGTTAACCAACATGTGTGAGGAACGAGCTCGGGTGTTGCAGGACCAGTTCAATGTTAGCATGAACCATGTCCACGCCTTGTCCATTCTCGTCTCTACCTTTCACCACGGAAAAACCCCTTCTGCCATTGATCAGAAAACGTTTGGTGAGTATACAGAGAGAACTAATTTCGAAAGACCTCTCACGAGCGGTGTTGCTTACGCATTGAAAGTCACACACTCTGAAAGAGAGAAATTCGAGAAGGAACATGGATGGTCAATAAAGAAAATGGACTTTGAAGACCAGACTCTCGTCCAAGGCTTCGATCCAGCTCCTGTTCAAGACGAATACGCACCCGTTATCTTCGCTCAAGAAACCGTCTCTCATATTGTCTCTGTCGACATGATGTCTGGAAAAGAAGACAGAGAAAACATCTTGAGAGCGAGAGCCTTAGGGAAAGGAGTGTTAACATCACCTTTCCAGCTTTTGAAATCAAACCATCTCGGCGTTATATTGACTTTCGCTGTGTACAACACCAACCTACCGCATGACGCTACTGAAGAAGAGAGGATCCAGGCAACAATTGGGTACCTTGGCGCATCGTACGATATGCCTTCACTAGTGGAGAAGCTTCTTCAACAGCTTGCGAGTAAACAGACCATATCTGTAAACGTTTACGACACGACTAACGCGTCGTCTGTCATTAAAATGTATGGCTCGGAGGTTGGTGATATGAGCGAAGAGCACATTAGTAGCCTTGATTTTGGTGATCCCTTTAGGAAGCATGAGATGCACTGCAGGTTTACACAAAAACCACCGATTCCTTGGTTAGCTATAATGCCACCAGGCTTCGCATTGGTTATCACGCTTCTTCTTGGTTATATCTTTAATGAAGCCATTAATCGAATTGCTACTGTTGAAGAGGACTATCAGAAAATGATGGAGCTTAAGGCTCGTGCTGAGGCTGCTGATGTGGCAAAGTCACAATTTCTAGCCACTGTTTCTCATGAGATACGTACCCCAATGGTTGGTGTTCTAGGGATGCTGAAGTTGCTGATGGACACTGATCTTGACGCAAAACAATTGGACTTTGCTGAAACGGCTCATGGCAGTGGGAAAGATCTAATATCGCTGATAAACGAGGTTCTTGATCAGGCGAAGATTGAGTCAGGAAGACTTGAGCTTGAGAACGTGCCTTTTGATCTACGGTTCCTTCTGGATAATGTTTCATCCCTCCTCTCTGGCAAGGCTGCTGAAAAAGGAATCGAGTTGGCTGTTTATGTTTCTAGCAATGTTCCAGCTGTTATAGTTGGTGATCCGGGTCGGTTCCGACAGATAATCACAAACCTTGTTGGAAACTCAATCAAGTTCACACAGGAAAAAGGACACATATTCATCTCGGTGCACCTAACCAATGAGGTGAGAGAACCGTTTGAAACAGAAGATGAAATACTAAAACAAAGACTTGGTTCGGATGAGACATCATGTAACACGCTTAGCGGGTATCCAGCTGTGAATGCAAGTGGAAGCTGGAGGAACTTCAAGACATTTCAGGACCATAGTTGTGATAAAACTGAGTTGCTTGTTACTGTGGAGGACACAGGGATTGGCATTCCTGTTGATGCACAGTCTCGGATCTTCACTCCGTTTATGCAAGCCGACAGTTCGACATCACGGACTTACGGTGGGACAGGGATAGGTTTAAGCATAAGCAAGCGTTTGGTTGAGCTCATGCAAGGAGAGATTGGGTTTGTAAGCAAGCTTGGTGTCGGCACTACTTTCTCATTTACCGGAGTTTTTGGAGAACGGGAAAGGGATTCATCAGTCACTGCCTTGGAGCTATTTGATCAATCTATTCAGGAGTTTCAAGGATTGAAAGCATTGGTTATTGATAGCAGAAACATTCGAGCCGAGGTTACTCGTTACCATCTTCAGAGAGTTGGTGTATCTTCTGTAGACATTGCTTCAAGTCTTGCAACTGCATCTTCTTCCTGTGCCAGTAAATTAGTGAACTTGGATTTGATTCTAATCGACAAAGATGCATGGAACAAGGAAGAGTATGTAGCATTTAACAGTGGTCAAGAGCCCTCTACGAGACACCCAAAGATTATACTTTTGGCTACCTTTACAACTCCAGCAGAGCTCAGTGAGATCAAATCAACTGGTTTGGTAGACGAGGTGGTGATGAAACCGCTTCGAATGAGCGTCTTGATATGCTGCTTGCAAGAAACCGTGGGAAGTGGTGGCAAGAAGAGGCAGACAAATAGAAAACCAAAGAATCTTGGGAACTTGCTTAGAGGAAAACATATTCTGGTGGTAGACGATACGATGGTAAACAGAAGAGTGGCAGATGCAGTGCTAAGGAAATATGGAGCTCTTGTGACTTGCGTTGACAGTGGCAAAGCTGCAGTTGCAATGCTTAAGCCGCCTCATGATTTTGACGCATGCTTCATGGATCTGCAGATGCCTGAAATGGATGGATTTGAAGCAACGAGGAGAGTACGTGGTCTGGAAGAAGAGATGAACAAGAAAGAAGAGTCAAGGAAGTGGCACACACCAATATTAGCTATGACTGCTGATGTGATCCAGGCAACAAATGAAAAATGCATCAAGTGTGGGATGGATGGTTTTGTATCGAAACCGTTTGAAGAAGAAGAGCTTTATTCATCAGTGGCAAGATTCTTCGAAAAATAA

>BrHK8(Bra030037)-DNA

ATGAGTCTGTTCCACGTGCTAGGGTTTTGTCTGAAGATTGGGCAGCTCTTCTGGATGCTATGCTGCTGGTTCCTTTCCTGGTTCCTTGATGCCGACAAGTCTCCTCTTGATCCGGACAAGACTAAGATGAAGAATCATAATAAGATGTGCTTCCTCTGGAACAAGATCTCCACAAGCGGACTCAAGATCCCGCCGAGTTTCTCTCATCATCTCTTTGGCTCCGTTAGATTCGGCAAGACTTTTTGGAGGAAGGTGCTGGTTGCTTGGGTCGTCTCCTGGGTTTTGATTTCTTTCTGGACTTTCTGGTGCCTTACCTCTCAAGCTATGGACAAGAGGAAAGAGACGCTTGCTAGTATGTGCGACGAGAGAGCTCGTATGCTCCAGGATCAGTTCAACGTTAGCATGAATCATGTTCAAGCTATGTCTATCTTGATCTCCACCTTCCACCATTCCAAGTTTCCTTCTGCCATCGATCAGGTCTCCTCTTCTTCTTCTTGTCTCCCAATCATTCATTCACTATATATACTGACGTGAAGAAGCTTTTTTTTTTGTAGAGGACGTTCTCGGAGTACACTGATAGAACTTCCTTTGAGAGGCCTCTCACGAGTGGTGTTGCATATGCGGTGAGAGTGCTCCACTCAGAGAGGCAAGAGTTTGAGAGGCAGCAAGGTTGGACTATCAGGAGGATGGAACAGAACCCTGTTCACAAGGATGACTATGACACTGAAGCTTTGGAACCATCTCCTGTTCAACAGGAGTATGCTCCTGTCATCTTTGCTCAGGACACGGTTTCCCACGTTATTTCTCTCGATATGCTGTCCGGGAAAGTAAGAACACATGGCTTGTTCATATTCAATCTCTGCTTTATAGTTTGGAGCAATGCTCACTCACTGCACTGAGTTTTTTCTTTATTGACAGGAAGATCGTGAAAACGTGTTGCGGGCCAGGAGATCAGGTAAAGGGGTTTTGACAGCTCCTTTCCCACTGATAAAGACGAATAGACTTGGGGTGATCTTGACGTTTGCTGTATACAAGAGAGATCTTCCTTCCAATGCAACCCCAAATGAGAGAATTGAGGCTACTAACGGGTTTGTACCATAGCCGTGTAAATAAATAAATAAACACGGAGTTTGAACATTTCAGTTTTACTTATTTCATCTCTTTTGATGTGTCCCTCAAGGTATCTTGGTGGAGTGTTTGACATTGAAACCTTGGTGGAAAACTTGCTTCAGCAGCTGGCTAGCAAGCAAACGATTCTTGTCAATGTATACGATACCACCAACCACTCTCAGCCCATTAGCATGTATGGTTCTCATGTGTCGGCTGATGGGTTGGAACATGTTAGTCCACTCAACTTCGGCGATCCATTTAGAAAGCATGAAATGCGTTGCAGGTACTTTGGCCAATTCAGCATGTCTTCAGTTTCTTCTTGTTTGCTCCAGGTGCTAACACTCGGTTGTGAGCTTTCTTCCTCTTTGCAGGTTTAAGCAGAAACCACCATGGCCAGTGCAATCGATGGTGACATCTTTTGGTATCCTTGTGATTGCGTTGCTTGTTGCACATATATTCCATGCAACCTTAAGTCGAATACGCAGAGCAGAAGAAGATTGTCATAAAATGGAGCTGCTCAAGAAAAAGGCTGAAGCAGCAGATGTCGCCAAGTCACAGGTACGTACATACATCATCCCCAATTGATAGCTTTCCACTGATAGAGGGAGGGGTTGTAATCTAACTTCTTTTATGCAGTTCCTTGCCACTGTATCACATGAAATCAGAACTCCAATGAATGGTGTTCTTGGTGAGTATCATCAAACTCCTTATCTCACCTCTCTTCCATGAATATTATTACTCAAGAAAAAAGCTGAACTAAATATCTTCCACTTTTGATTTGGGCTGTAGGAATGCTCCATATGCTTATGGACACAGAGTTAGATGTTACACAACAGGATTACGTTAGGACCGCACAGGCAAGTGGGAAAGCTTTAGTGTCGCTAATAAACGAGGTACTGGATCAAGCAAAGATCGAATCTGGAAAGGTTGAGCTTGAGGAGGTGCGGTTTGATTTGAGAGGAATATTAGATGATGTCCTGTCACTCTTCTCTGGCAAGTCCCAAGAAAAAGGGCTAGAGGTAAACACACAAATGATCTTTAATGATTTGCTAAGCAAAGGTTTGTTTGTAACTTGTAAGTGTTGCTGATGAAATGCAGTTGGCAGTATACATATCTGACCGTGTTCCAGAAATGTTAATTGGGGATCCTGGAAGGTTTCGACAAATACTCACAAATCTTATGGGTAATTCCATTAAGGTAAATGTGATATACTTTCCTTATCATTCCATGCATCCCTAGTTGATAGCTGATCAGTTATAAACATCCTGGCAGTTCACTGAGAAAGGTCACATCTTTGTAACTGTTCATCTGGTGGAGGAGCTACTAGACTCTAGTGATGTAGAGACATCATCATCATCATCTACAGAAAACACACTGAGCGGGCTTCCAGTTGCAGACCGGAAGAGAAGCTGGCAAAACTTCAAAGCTTTCAGTTCCAATGGACATAGGAGCTTAGCACTAGCACCATCTGAAATCAACCTAGTCGTCTCAGTTGAGGATACTGGCGTAGGGATCCCTGTAGAAGCGCAGTCACGCATTTTCACACCGTTCATGCAAGTTGGACCATCTATATCCAGGACGCATGGAGGCACAGGGATCGGGCTCAGCATAAGCAAGTGTCTAGTAGGACTGATGAAGGGAGAAATTGGATTCTCGAGTACTCCCAAGGTTGGGTCCACGTTCACGTTTACTGTCGTGTTTGCTAATGGCGTGCATTCAACTGAAAGAAAGAGTGAACTGCATAACAATAATCAGCCCGAGTTTGAGGGAATGAACGCTGTACTTGTGGACCATAGGCCCGCTCGGGCACAAGTCTCGTGGTACCATTTTCAGCGTCTAGGAATCCGAGTTGAGGTAGTTACATCTGTTGATCAGGCTCTACGTTTCATGAAGACTTACGCTACCACTGTGAATATGATACTCATAGAGCAAGAAGTGTGGAATAAAGAAGCTGATGTGTTCGTTAAGGACCCTCTTGTCCATTCTCCTAAACTGTTTTTGTTAGCAAATTCAATAGACACCTCAGTATCAGATACTTTAAGCAACGTTATAGACCCTCCAGTGTTGATAGTGAAGCCGTTAAGGGCGAGTATGCTGGCAGCAACTTTGCAGAGAGGGTTAGGTATTGGGAAGAGGGAAACTCCTCAACGCAAGGGACCTCCTGCTTTGATTCTCAGGAATCTTCTCCTTGGCAGAAAAATACTAATTGTGGATGATAACAACGTGAACCTCAGGGTGGCAGCAGGAGCTTTGAAAAAGTATGGAGCTGATGTGGTCTGCGCTGAGAGCGGGGTAAAGGCAGTCTCATTGCTTAAGCCACCACACGAGTTCGATGCTTGCTTCATGGACATTCAGATGCCAGAAATGGATGGGTAATGTCTTGAATGTTACAGCAAGGTTTGTGATAATGTTTGAAAATATGTAATAAATGCTTGAGATGTCTTTCTGCAGGTTTGAGGCTACGAGGAGAATACGGGGTATGGAAGAGGAGATGAACAATGGGGAGGCGTTGACAGTAGAGGAAGGTAAGAGATCAAGATGGCATCTTCCGGTGTTAGCAATGACTGCTGATGTGATCCAAGCAACGCATGAGAAATGTCTAAAGTGTGGAATGGATGGGTATGTATCAAAACCCTTTGAAGCAGAGCAGTTGTACAGAGAAGTTTCTCGTTTTTTCAATTCGCCTTCAGATACAGAATCATAA

>BrHK8(Bra030037)-CDS

ATGAGTCTGTTCCACGTGCTAGGGTTTTGTCTGAAGATTGGGCAGCTCTTCTGGATGCTATGCTGCTGGTTCCTTTCCTGGTTCCTTGATGCCGACAAGTCTCCTCTTGATCCGGACAAGACTAAGATGAAGAATCATAATAAGATGTGCTTCCTCTGGAACAAGATCTCCACAAGCGGACTCAAGATCCCGCCGAGTTTCTCTCATCATCTCTTTGGCTCCGTTAGATTCGGCAAGACTTTTTGGAGGAAGGTGCTGGTTGCTTGGGTCGTCTCCTGGGTTTTGATTTCTTTCTGGACTTTCTGGTGCCTTACCTCTCAAGCTATGGACAAGAGGAAAGAGACGCTTGCTAGTATGTGCGACGAGAGAGCTCGTATGCTCCAGGATCAGTTCAACGTTAGCATGAATCATGTTCAAGCTATGTCTATCTTGATCTCCACCTTCCACCATTCCAAGTTTCCTTCTGCCATCGATCAGAGGACGTTCTCGGAGTACACTGATAGAACTTCCTTTGAGAGGCCTCTCACGAGTGGTGTTGCATATGCGGTGAGAGTGCTCCACTCAGAGAGGCAAGAGTTTGAGAGGCAGCAAGGTTGGACTATCAGGAGGATGGAACAGAACCCTGTTCACAAGGATGACTATGACACTGAAGCTTTGGAACCATCTCCTGTTCAACAGGAGTATGCTCCTGTCATCTTTGCTCAGGACACGGTTTCCCACGTTATTTCTCTCGATATGCTGTCCGGGAAAGAAGATCGTGAAAACGTGTTGCGGGCCAGGAGATCAGGTAAAGGGGTTTTGACAGCTCCTTTCCCACTGATAAAGACGAATAGACTTGGGGTGATCTTGACGTTTGCTGTATACAAGAGAGATCTTCCTTCCAATGCAACCCCAAATGAGAGAATTGAGGCTACTAACGGGTATCTTGGTGGAGTGTTTGACATTGAAACCTTGGTGGAAAACTTGCTTCAGCAGCTGGCTAGCAAGCAAACGATTCTTGTCAATGTATACGATACCACCAACCACTCTCAGCCCATTAGCATGTATGGTTCTCATGTGTCGGCTGATGGGTTGGAACATGTTAGTCCACTCAACTTCGGCGATCCATTTAGAAAGCATGAAATGCGTTGCAGGTTTAAGCAGAAACCACCATGGCCAGTGCAATCGATGGTGACATCTTTTGGTATCCTTGTGATTGCGTTGCTTGTTGCACATATATTCCATGCAACCTTAAGTCGAATACGCAGAGCAGAAGAAGATTGTCATAAAATGGAGCTGCTCAAGAAAAAGGCTGAAGCAGCAGATGTCGCCAAGTCACAGTTCCTTGCCACTGTATCACATGAAATCAGAACTCCAATGAATGGTGTTCTTGGAATGCTCCATATGCTTATGGACACAGAGTTAGATGTTACACAACAGGATTACGTTAGGACCGCACAGGCAAGTGGGAAAGCTTTAGTGTCGCTAATAAACGAGGTACTGGATCAAGCAAAGATCGAATCTGGAAAGGTTGAGCTTGAGGAGGTGCGGTTTGATTTGAGAGGAATATTAGATGATGTCCTGTCACTCTTCTCTGGCAAGTCCCAAGAAAAAGGGCTAGAGTTGGCAGTATACATATCTGACCGTGTTCCAGAAATGTTAATTGGGGATCCTGGAAGGTTTCGACAAATACTCACAAATCTTATGGGTAATTCCATTAAGTTCACTGAGAAAGGTCACATCTTTGTAACTGTTCATCTGGTGGAGGAGCTACTAGACTCTAGTGATGTAGAGACATCATCATCATCATCTACAGAAAACACACTGAGCGGGCTTCCAGTTGCAGACCGGAAGAGAAGCTGGCAAAACTTCAAAGCTTTCAGTTCCAATGGACATAGGAGCTTAGCACTAGCACCATCTGAAATCAACCTAGTCGTCTCAGTTGAGGATACTGGCGTAGGGATCCCTGTAGAAGCGCAGTCACGCATTTTCACACCGTTCATGCAAGTTGGACCATCTATATCCAGGACGCATGGAGGCACAGGGATCGGGCTCAGCATAAGCAAGTGTCTAGTAGGACTGATGAAGGGAGAAATTGGATTCTCGAGTACTCCCAAGGTTGGGTCCACGTTCACGTTTACTGTCGTGTTTGCTAATGGCGTGCATTCAACTGAAAGAAAGAGTGAACTGCATAACAATAATCAGCCCGAGTTTGAGGGAATGAACGCTGTACTTGTGGACCATAGGCCCGCTCGGGCACAAGTCTCGTGGTACCATTTTCAGCGTCTAGGAATCCGAGTTGAGGTAGTTACATCTGTTGATCAGGCTCTACGTTTCATGAAGACTTACGCTACCACTGTGAATATGATACTCATAGAGCAAGAAGTGTGGAATAAAGAAGCTGATGTGTTCGTTAAGGACCCTCTTGTCCATTCTCCTAAACTGTTTTTGTTAGCAAATTCAATAGACACCTCAGTATCAGATACTTTAAGCAACGTTATAGACCCTCCAGTGTTGATAGTGAAGCCGTTAAGGGCGAGTATGCTGGCAGCAACTTTGCAGAGAGGGTTAGGTATTGGGAAGAGGGAAACTCCTCAACGCAAGGGACCTCCTGCTTTGATTCTCAGGAATCTTCTCCTTGGCAGAAAAATACTAATTGTGGATGATAACAACGTGAACCTCAGGGTGGCAGCAGGAGCTTTGAAAAAGTATGGAGCTGATGTGGTCTGCGCTGAGAGCGGGGTAAAGGCAGTCTCATTGCTTAAGCCACCACACGAGTTCGATGCTTGCTTCATGGACATTCAGATGCCAGAAATGGATGGGTTTGAGGCTACGAGGAGAATACGGGGTATGGAAGAGGAGATGAACAATGGGGAGGCGTTGACAGTAGAGGAAGGTAAGAGATCAAGATGGCATCTTCCGGTGTTAGCAATGACTGCTGATGTGATCCAAGCAACGCATGAGAAATGTCTAAAGTGTGGAATGGATGGGTATGTATCAAAACCCTTTGAAGCAGAGCAGTTGTACAGAGAAGTTTCTCGTTTTTTCAATTCGCCTTCAGATACAGAATCATAA

>BrHK9(Bra024849)-DNA

ATGAACTGGGCACTCAACAACCCAAATCCTGAAGAGAAGGAGCCAACAACAACAACCCAAAGCAGCGATTTTTACCATCTGGGCGCTAAAGATTCGCCACAAAAGCCCAGAAAAATCGATTTTTGGCGTTCTGGGTTGATGGGTTTCGCCAAGATGCAGCATTCAGTGGCGGTGAAGATGAACAACGGTAATAATAACGACCAAGTGGGTAACAAAAAGGGGTCAACTTTCATACAGGAACACAGAGCTTTATTACCAAAGGGTTTGATTCTATGGACAATCATTGTCGGGTTTATAAGCAGAGGGATTTATCAGTGGATGGATGATACTAGCAAGGTCAGAAGAGAAGAGGTTTTGGTTAGTATGTGTGATCAGAGAGCCAGGATGTTGCAGGATCAGTTTAGTGTTAGTGTTAACCATGTTCACGCTTTGGCTATTCTTGTCTCAACGTTTCATTACCACAAGAATCCATCTGCAATTGATCAGGTTAGTGTCTGCCTTATTAAAGGTTAAAGCTTTAGAGAAAAAGATCAAGATTTGTTCATATTTGATTTATAATTGAATAGTTGACTTTTCTTGAAATTTTTTTAATTACTAGTCTAGTTCTGTTTATGAATTAGAGTTTTACATTCATTGTAAAAAATGTTTTGAATCGAATAAATTTGACATTCATTCAAAAAAAAAATAATTGCAGGGGACATTTGCTGACTATACAGCGAGAACAGCATTTGAGAGACCGTTGCTAAGTGGAGTGGCTTACGCTGAAAAGGTTGTGAATGCTGAGAGGGAGATGTTTGAGAGTCAGCACAATTGGGTTATAAAGACAATGGACACAGGAGAGCCTTCACCTGTGAGGGACGAGTATGCTCCCGTCATCTTCTCTCAAGACAGTGTCTCTTACCTCGAGTCACTAGACATGATGTCAGGAGAGGTAATATTTATTACAGATACTTCTTTGTTTTTGGATTATCTTGGTTCTGAAAACTTTTGCTTACAGGAGGATAGAGAGAACATTCTGAGAGCTAGAGAGACAGGGAAAGCTGTCTTAACAAGCCCTTTTAGACTACTGGCTTCTCACCATCTAGGAGTTGTGTTAACCTTCCCTGTGTACAAAGCCTCTCTTCCTAAAAACCCCACCGTCCAAGAGCGTATAGCAGCCACCGCAGGGTACCTCGGCGGCGCGTTTGACGTTGAGTCACTCGTTGAGAATCTACTCGGTCAGCTCGCTGGCAACCAGGCGATAGTAGTGCATGTGTATGACATCACCAACGCGTCGGATCCTCTCGTCATGTACGGGAACCAAGACGAAGAAGGCGACACGTCTCTCTACCACGAGAGCAAGCTTGATTTTGGAGACCCTTTCAGGAAGCATAAGATGATCTGTAGGTACCTCCAGAAGGCGCCTATACCGTTGAACGTACTCACGACCGTGCCGTTGTTCTTTGCTATTGGCTTCTTGGTTGGTTACATACTCTACGGTGCAGCTGTTCATATAGTTAAAGTTGAAGATGATTTCCATGAGATGCAAGAGCTCAAGGTCCGAGCAGAAGCTGCTGACGTGGCTAAATCGCAGTTTCTTGCGACCGTCTCTCACGAGATAAGGACGCCGATGAATGGGATTCTTGGAATGCTTGCTATGCTTCTTGATACGGAGCTTAGCTCTACGCAGAGAGATTACGCTCAGACCGCGCAGGTTTGTGGTAAAGCTTTGATTGCGTTGATAAACGAGGTTCTTGATCGTGCCAAGATCGAAGCTGGGAAGCTGGAGTTGGAGTCTGTGCCTTTTGATATACGTTCGATATTAGATGATGTTCTTTCTCTCTTCTCTGAGGAGTCAAGGAACAAAGGCATTGAGGTAAAAGAATGTTTAAAGAAAAGCTCTTTTTGTAGTAGTTAGCTGCTACTTACTAACTTCTTGAATTTTGTTTGAATTTTTTGGCAGCTTGCGGTTTTCGTTTCAGACAAGGTACCTGAGATAGTCAAAGGAGATTCAGGGAGATTCAGACAGATCATCATAAACCTTGTCGGAAACTCTGTTAAAGTTAGTTCCTTTCCTCCTATGTTCTTGTCTAGGCCTGCTGGTTCCGGTAGTTCAGTTTTCGGATAGTTTGGTTCAACACAAATATTAACGAATTAATCCAAAATAAAGTTCGGTTCGATATTTGGTTAGTTTGGTTTTTGAAAAGTATACTGAAATTTATGATTTCGGTTATATTTTGGTTTAATTTTGTTAAAATTTGAATAAGTTTGGTTAATTTCAGTTAGTTCGGTTTAAAATTTGGTTAGTTTAGTTCAGATTTTTGGTATGGTTTGGTTGTAATTTTCTTAATTTTTTTAAGTAAACCAAAATAACTGATTGCCGAACCTAAAACCAAACTTTTGTAAATAACCTACAAAAACGAACCAAACTCCTAACCAAAATTAATTTTGGTTCGGTCTAATTTAAAATCCCTGCCTTATTCTTGTCCCCCTGTGGGAAGTAGATTGTTGTTTGATCAAGATCTTGTCTTTTTTGACACAGTTCACAGAGAAAGGACATATCTTTGTCAAAGTCCATCTGGCGGAACAATCAAAAGACGGAGCTGAATCCAAACCCGCACTAAACGGAGGAGTAGCCTCTGAAGACATAACCGCCGCTTCCAAACCTTCAAGTTACAACACACTGAGCGGCTACGAAGCTGCTGACGGTCGAAACAGCTGGGACTCATTCAAACACTTACTCTCGTCGGAGGAGCTGTTGACATCATCAGAGTTCGAAGCTTCCAGTAACGACAGGCTTATGGTTTCTATCGAAGACACAGGCATTGGGATCCCTTTAACCGCGCAAGGACGTGTCTTCATGCCGTTTATGCAAGCGGATAGCTCCACTTCGAGAACCTACGGAGGTACTGGGATTGGTTTGAGTATAAGCAAGTGTCTTGTCGAGCTTATGCGCGGTCAGATAAGTTTCGTGAGCAGGCCTCGCGTTGGTAGCACGTTTTGGTTCACTGCTGTGTTTGAGAGGTGTGATAAATGTAGTCTGAAGAAGCCTACGGTTGAGAATCTGCCTTCTAGTTTTAGAGGGATGAGAGCTATTGTTGTTGATGCTAAGCCTGTTCGAGCTGCGGTGACTAGGTATCATATGAAGAGACTTGGGATCAGTGTTGATGTCATGACAAGTCTCAGAACCGCTGTTTCTACTGCGTCTGGAAGAAACGGTTCTCCTCTTCCTTCAGGGTAAGTAAAAGGTTTAAATCTCTTTTGATTTCTGTTGGTTCCATCTGATGATTTAATCTTTTTTTTTTTGCAGAACAACGAAACTGGATATGATCTTGGTGGAGAAAGATTCATGGATATCAACCGAAGATATAGACGCGGAGATACGTCAGATGAACTCAAGAACCAACGGAAACGTGCATCACAAGACACCGAAACTCGCTCTATTCGCAACGAACATCACCAACTCAGAGTTCGACAGAGCTAAATCCGCAGGGTTTGCTGATACGGTGATAATGAAGCCGTTGAGAGCAAGCATGATCGGCGCGTGTTTACAGCAAGTTCTCGAGCTGAGAAAGGCGAGACAGCAGCATCCTGAGGGATCATCACCAGCAACGCTCAAGAGTTTGCTTACAGGGAAGAAGATTCTGGTGGTTGATGATAATATGGTGAACAGGAGAGTAGCTGCAGGAGCTCTGAAGAAGTTTGGAGCAGAGGTGGTGTGTGCAGAGAGTGGTCAATTTGCTTTGGGTTTGCTTCAGATTCCACACAGTTTCGATGCTTGCTTCATGGATATTCAAATGCCACAGATGGACGGGTAAGCCTTTAAACTTGGCCTCCTGTTCCGTAGCTTGATTTTTGGTTAGTTCGGCTCAACATAAATCTTACCGAATTAATCCAAAATAAAGTTTGGTTCGGTATTCGGTTAGTTTGGTTTTGAAAATCGTACATAGGTTTTTGATTTGGGTTTATATTTTGATTAATTTTGTTAAAATTTCGGATAAGTTCGGTTGGTTCGATTTGGGTTTTTGATATTGTTTGGTTATTTTTTTTTAAAGAAAACCAAAGTAACCGATTGCCAAACCAAACCTTTTCAAACCTACAAAATCGAACCAAACTCCTAACTTTTTTGGTTCGGTTTAGTTCGGTTCAAAATCCCAGCCCTACTTAAACTCATTAGGTTTGGTCTTGTACTACTTGGTTTACGTTCTGGTTTTGTTCTCTCAGGTTTGAAGCGACTCGTCAGATAAGGATGATGGAGAAGGAAGCTAAAGAGAAGACGAAGCTGGAATGGCATTTACCGATTCTAGCCATGACAGCTGATGTGATCCACGCGACATACGAGGAGTGTCTGAAAAGTGGAATGGATGGTTATGTCTCTAAACCATTTGAAGAAGAGAATCTCTACAAGTCTGTTGCCAAATCATTCAAAGCTAACCCAATCTCAGATTCATCATGTAGCCAAAGTTGA

>BrHK9(Bra024849)-CDS

ATGAACTGGGCACTCAACAACCCAAATCCTGAAGAGAAGGAGCCAACAACAACAACCCAAAGCAGCGATTTTTACCATCTGGGCGCTAAAGATTCGCCACAAAAGCCCAGAAAAATCGATTTTTGGCGTTCTGGGTTGATGGGTTTCGCCAAGATGCAGCATTCAGTGGCGGTGAAGATGAACAACGGTAATAATAACGACCAAGTGGGTAACAAAAAGGGGTCAACTTTCATACAGGAACACAGAGCTTTATTACCAAAGGGTTTGATTCTATGGACAATCATTGTCGGGTTTATAAGCAGAGGGATTTATCAGTGGATGGATGATACTAGCAAGGTCAGAAGAGAAGAGGTTTTGGTTAGTATGTGTGATCAGAGAGCCAGGATGTTGCAGGATCAGTTTAGTGTTAGTGTTAACCATGTTCACGCTTTGGCTATTCTTGTCTCAACGTTTCATTACCACAAGAATCCATCTGCAATTGATCAGGGGACATTTGCTGACTATACAGCGAGAACAGCATTTGAGAGACCGTTGCTAAGTGGAGTGGCTTACGCTGAAAAGGTTGTGAATGCTGAGAGGGAGATGTTTGAGAGTCAGCACAATTGGGTTATAAAGACAATGGACACAGGAGAGCCTTCACCTGTGAGGGACGAGTATGCTCCCGTCATCTTCTCTCAAGACAGTGTCTCTTACCTCGAGTCACTAGACATGATGTCAGGAGAGGAGGATAGAGAGAACATTCTGAGAGCTAGAGAGACAGGGAAAGCTGTCTTAACAAGCCCTTTTAGACTACTGGCTTCTCACCATCTAGGAGTTGTGTTAACCTTCCCTGTGTACAAAGCCTCTCTTCCTAAAAACCCCACCGTCCAAGAGCGTATAGCAGCCACCGCAGGGTACCTCGGCGGCGCGTTTGACGTTGAGTCACTCGTTGAGAATCTACTCGGTCAGCTCGCTGGCAACCAGGCGATAGTAGTGCATGTGTATGACATCACCAACGCGTCGGATCCTCTCGTCATGTACGGGAACCAAGACGAAGAAGGCGACACGTCTCTCTACCACGAGAGCAAGCTTGATTTTGGAGACCCTTTCAGGAAGCATAAGATGATCTGTAGGTACCTCCAGAAGGCGCCTATACCGTTGAACGTACTCACGACCGTGCCGTTGTTCTTTGCTATTGGCTTCTTGGTTGGTTACATACTCTACGGTGCAGCTGTTCATATAGTTAAAGTTGAAGATGATTTCCATGAGATGCAAGAGCTCAAGGTCCGAGCAGAAGCTGCTGACGTGGCTAAATCGCAGTTTCTTGCGACCGTCTCTCACGAGATAAGGACGCCGATGAATGGGATTCTTGGAATGCTTGCTATGCTTCTTGATACGGAGCTTAGCTCTACGCAGAGAGATTACGCTCAGACCGCGCAGGTTTGTGGTAAAGCTTTGATTGCGTTGATAAACGAGGTTCTTGATCGTGCCAAGATCGAAGCTGGGAAGCTGGAGTTGGAGTCTGTGCCTTTTGATATACGTTCGATATTAGATGATGTTCTTTCTCTCTTCTCTGAGGAGTCAAGGAACAAAGGCATTGAGCTTGCGGTTTTCGTTTCAGACAAGGTACCTGAGATAGTCAAAGGAGATTCAGGGAGATTCAGACAGATCATCATAAACCTTGTCGGAAACTCTGTTAAATTCACAGAGAAAGGACATATCTTTGTCAAAGTCCATCTGGCGGAACAATCAAAAGACGGAGCTGAATCCAAACCCGCACTAAACGGAGGAGTAGCCTCTGAAGACATAACCGCCGCTTCCAAACCTTCAAGTTACAACACACTGAGCGGCTACGAAGCTGCTGACGGTCGAAACAGCTGGGACTCATTCAAACACTTACTCTCGTCGGAGGAGCTGTTGACATCATCAGAGTTCGAAGCTTCCAGTAACGACAGGCTTATGGTTTCTATCGAAGACACAGGCATTGGGATCCCTTTAACCGCGCAAGGACGTGTCTTCATGCCGTTTATGCAAGCGGATAGCTCCACTTCGAGAACCTACGGAGGTACTGGGATTGGTTTGAGTATAAGCAAGTGTCTTGTCGAGCTTATGCGCGGTCAGATAAGTTTCGTGAGCAGGCCTCGCGTTGGTAGCACGTTTTGGTTCACTGCTGTGTTTGAGAGGTGTGATAAATGTAGTCTGAAGAAGCCTACGGTTGAGAATCTGCCTTCTAGTTTTAGAGGGATGAGAGCTATTGTTGTTGATGCTAAGCCTGTTCGAGCTGCGGTGACTAGGTATCATATGAAGAGACTTGGGATCAGTGTTGATGTCATGACAAGTCTCAGAACCGCTGTTTCTACTGCGTCTGGAAGAAACGGTTCTCCTCTTCCTTCAGGAACAACGAAACTGGATATGATCTTGGTGGAGAAAGATTCATGGATATCAACCGAAGATATAGACGCGGAGATACGTCAGATGAACTCAAGAACCAACGGAAACGTGCATCACAAGACACCGAAACTCGCTCTATTCGCAACGAACATCACCAACTCAGAGTTCGACAGAGCTAAATCCGCAGGGTTTGCTGATACGGTGATAATGAAGCCGTTGAGAGCAAGCATGATCGGCGCGTGTTTACAGCAAGTTCTCGAGCTGAGAAAGGCGAGACAGCAGCATCCTGAGGGATCATCACCAGCAACGCTCAAGAGTTTGCTTACAGGGAAGAAGATTCTGGTGGTTGATGATAATATGGTGAACAGGAGAGTAGCTGCAGGAGCTCTGAAGAAGTTTGGAGCAGAGGTGGTGTGTGCAGAGAGTGGTCAATTTGCTTTGGGTTTGCTTCAGATTCCACACAGTTTCGATGCTTGCTTCATGGATATTCAAATGCCACAGATGGACGGGTTTGAAGCGACTCGTCAGATAAGGATGATGGAGAAGGAAGCTAAAGAGAAGACGAAGCTGGAATGGCATTTACCGATTCTAGCCATGACAGCTGATGTGATCCACGCGACATACGAGGAGTGTCTGAAAAGTGGAATGGATGGTTATGTCTCTAAACCATTTGAAGAAGAGAATCTCTACAAGTCTGTTGCCAAATCATTCAAAGCTAACCCAATCTCAGATTCATCATGTAGCCAAAGTTGA

>BrHK10(Bra004160)-DNA

ATGATGGAAGTCTGCAACTGCATCGAGCCTCAATGGCCAGCGGACGAGCTCCTAATGAAGTACCAATACATCTCAGACTTCTTCATCGCCGTCGCCTACTTCTCAATCCCCCTCGAGCTCATCTACTTCGTCAAAAAATCAGCCGTCTTCCCTTACAGGTGGGTCCTCGTCCAGTTCGGCGCCTTCATCGTCCTCTGCGGCGCCACCCACCTCATCAACCTATGGACCTTCACCACCCATTCCCGAACCGTCGCCCTCGTTATGACCACCGCCAAGGTCTTAACCGCCGTCGTCTCCTGCGCCACGGCGTTGATGCTCGTCCACATCATCCCTGACCTCCTCAGCGTCAAGACGCGCGAGCTCTTCTTGAAAAACAAGGCTGCTGAGCTGGACAGGGAGATGGGATTGATCCGGACTCAGGAGGAGACCGGGAGGCACGTGAGGATGCTGACTCATGAGATTAGAAGCACGTTGGATAGGCACACGATCTTGAAAACCACACTAGTTGAACTTGGGAGGACGTTGGCGTTGGAGGAGTGTGCGTTGTGGATGCCCACTAGGACTGGTTTGGAGTTGCAGCTTTCGTATACGCTTCGTCAGCAGCATCCTGTTGAGTACACGGTGCCTATTCAGTTGCCGGTGATTAACCAGGTGTTTGGGACTAGCAGAGCTGTGAAGATATCTCCTAACTCCCCGGTGGCGAGGCTGAGGCCTGTCTCCGGGAAGTATCTTCTCGGGGAGGTGGTGGCTGTGAGGGTTCCGCTTCTCCACCTTTCGAATTTTCAGATTAATGATTGGCCTGAGCTTTCGACGAAGCGATACGCTCTTATGGTGTTGATGCTTCCTTCGGATAGTGCGAGGCAGTGGCATGTTCATGAGCTGGAGCTAGTCGAAGTCGTTGCGGATCAGGTAATGTACACACTTTGGAGCTCAGTTTATGTCTTATGGTGAGCTTGTCTGAACTCTTTTTTTTTATTGTAGGTGGCCGTAGCTCTCTCACATGCTGCGATTCTGGAAGAGTCGATGCGAGCTAGGGACCTTCTGATGGAGCAGAACGTGGCTCTTGATATAGCGAGAAGAGAAGCGGAAACAGCGATCCGTGCTCGGAATGATTTCCTGGCGGTTATGAACCATGAGATGCGGACGCCAATGCATGCGATCATTGCGCTCTCTTCCTTACTTCAAGAGACGGAGTTGACCCCAGAACAAAGGTTGATGGTGGAGACAGTGCTGAAAAGCAGTAGCCTTTTGGCAACTCTGATGAATGATGTCTTGGATCTTTCGAGGCTAGAAGATGGAAGTCTTCAACTTGAGCTCGGGACATTTAATCTTCATACTTTATTTAGAGAGGTAATACCATATTTTACCTCCTGCATAGGCCTGCGAGTACATTAGGATAATTCGATTTTTAAATTTTTTACGAAAATTAACCAAAGTGTTTGGTTTTGATTATATTTTGGTTAAATTCCGGGCTATTGCGGTTCAAAATTTTGGTTAATTCAGATTGAAATTTGGTTAACAATTTTTTTCTTTTTTTTAAAAACCAAACTAACCGATTACTGGACCCAAAACCGGAGTTTTTTATAAACCTGCCAAACTGAACCAAACTAAACGAAAAATTTCCTTTGGTTTGGCTCAGTTCAAACCTGCAGGCCTACTCCTGCATGTCATTGTTTGTATATTTTAGAACAGCTTCATGTTTTAGAAGTCAGAGTATTTGGTTGAAATTTTTGAATCTTGTTGCAGGTCCTTAATCTGATAAAGCCTATAGCCGTTGTTAAGAAATTACCCATCACACTAAACCTCGCACCAGATTTGCCAGAGTTTGTGGTTGGGGATGAGAAACGGCTAATGCAGATAATATTAAACATTGTTGGTAACGCTGTGAAGTTCTCAAAACAAGGTAGCATCTCCGTAACTGCTCTTGTCACAAAGTCAGACAACCGAGCTCCTCCTGACTTCTTTGTGGTGCCAACTGGTAGTCATTTCTACTTGAGAGTAAAGGTTAGTATCTTTGGTCCCTTAAACCACAGATGAGAGTACTCTCATAGATCTTACTATTGACGATTATTCAACCATAGGTAAAAGACTTGGGAGCAGGAATCAATCCGCAAGACATTCCCAAGCTATTCACTAAATTTGCTCAAACACAGTCTTTAGCGACCAGAAGCTCGGGAGGTAGTGGGCTTGGTCTCGCCATCTCCAAGAGGTTGAGCCCTATTTAAAATCCTTTTTTTTCTTCTCTTATGGTACTGATAAACGTTTGCTCATATGTCAAGGTTTGTGAATCTGATGGAGGGTAACATTTGGATTGAGAGCGAAGGTGTTGGAAAAGGATGCACTGCTATCTTTGATGTTAAACTTGGGATTTCAAACGAATCTAAACAGTCTGGCATTCCAAAAGTTCCAGCCAATCCACAGCATGTTAATTTCGCTGGACTGAAGGTTCTTGTCATGGATGAGAATGGGTTAGTACAAGCCTATTAGTTCTATCTTTCTGCTGTTTGTTATCAGCGATTGATGTTTGCTTTGGTTCGCAGGGTAAGTAGAATGGTGACGAAGGGACTTCTTGTACACCTTGGATGCGAAGTGGCCACGGTGAGTTCAAGCGAGGAGTGTCTCAGAGTTGTATCCCATGAGCACAGAGTGGTCTTCATGGACGTGTGCACCCCCGGGGTCGAAAACTACCAGATCGCTCTCCGTATACACGAGAAATTCACAAAACGCCACCAAAGGCCACTGCTCGTGGCGCTCACTGGTAACACCGACAAATCCACAAAGGAGAGATGTATGAGCTTTGGTCTAGACGGCGTGTTGCTCAAACCCGTGTCGCTAGACAACATGAGAAACGTTCTGTCTGATCTTCTAGAACATCGGGTTCTGTACGAGGCCATGTAA

>BrHK10(Bra004160)-CDS

ATGATGGAAGTCTGCAACTGCATCGAGCCTCAATGGCCAGCGGACGAGCTCCTAATGAAGTACCAATACATCTCAGACTTCTTCATCGCCGTCGCCTACTTCTCAATCCCCCTCGAGCTCATCTACTTCGTCAAAAAATCAGCCGTCTTCCCTTACAGGTGGGTCCTCGTCCAGTTCGGCGCCTTCATCGTCCTCTGCGGCGCCACCCACCTCATCAACCTATGGACCTTCACCACCCATTCCCGAACCGTCGCCCTCGTTATGACCACCGCCAAGGTCTTAACCGCCGTCGTCTCCTGCGCCACGGCGTTGATGCTCGTCCACATCATCCCTGACCTCCTCAGCGTCAAGACGCGCGAGCTCTTCTTGAAAAACAAGGCTGCTGAGCTGGACAGGGAGATGGGATTGATCCGGACTCAGGAGGAGACCGGGAGGCACGTGAGGATGCTGACTCATGAGATTAGAAGCACGTTGGATAGGCACACGATCTTGAAAACCACACTAGTTGAACTTGGGAGGACGTTGGCGTTGGAGGAGTGTGCGTTGTGGATGCCCACTAGGACTGGTTTGGAGTTGCAGCTTTCGTATACGCTTCGTCAGCAGCATCCTGTTGAGTACACGGTGCCTATTCAGTTGCCGGTGATTAACCAGGTGTTTGGGACTAGCAGAGCTGTGAAGATATCTCCTAACTCCCCGGTGGCGAGGCTGAGGCCTGTCTCCGGGAAGTATCTTCTCGGGGAGGTGGTGGCTGTGAGGGTTCCGCTTCTCCACCTTTCGAATTTTCAGATTAATGATTGGCCTGAGCTTTCGACGAAGCGATACGCTCTTATGGTGTTGATGCTTCCTTCGGATAGTGCGAGGCAGTGGCATGTTCATGAGCTGGAGCTAGTCGAAGTCGTTGCGGATCAGGTGGCCGTAGCTCTCTCACATGCTGCGATTCTGGAAGAGTCGATGCGAGCTAGGGACCTTCTGATGGAGCAGAACGTGGCTCTTGATATAGCGAGAAGAGAAGCGGAAACAGCGATCCGTGCTCGGAATGATTTCCTGGCGGTTATGAACCATGAGATGCGGACGCCAATGCATGCGATCATTGCGCTCTCTTCCTTACTTCAAGAGACGGAGTTGACCCCAGAACAAAGGTTGATGGTGGAGACAGTGCTGAAAAGCAGTAGCCTTTTGGCAACTCTGATGAATGATGTCTTGGATCTTTCGAGGCTAGAAGATGGAAGTCTTCAACTTGAGCTCGGGACATTTAATCTTCATACTTTATTTAGAGAGGTCCTTAATCTGATAAAGCCTATAGCCGTTGTTAAGAAATTACCCATCACACTAAACCTCGCACCAGATTTGCCAGAGTTTGTGGTTGGGGATGAGAAACGGCTAATGCAGATAATATTAAACATTGTTGGTAACGCTGTGAAGTTCTCAAAACAAGGTAGCATCTCCGTAACTGCTCTTGTCACAAAGTCAGACAACCGAGCTCCTCCTGACTTCTTTGTGGTGCCAACTGGTAGTCATTTCTACTTGAGAGTAAAGGTAAAAGACTTGGGAGCAGGAATCAATCCGCAAGACATTCCCAAGCTATTCACTAAATTTGCTCAAACACAGTCTTTAGCGACCAGAAGCTCGGGAGGTAGTGGGCTTGGTCTCGCCATCTCCAAGAGGTTTGTGAATCTGATGGAGGGTAACATTTGGATTGAGAGCGAAGGTGTTGGAAAAGGATGCACTGCTATCTTTGATGTTAAACTTGGGATTTCAAACGAATCTAAACAGTCTGGCATTCCAAAAGTTCCAGCCAATCCACAGCATGTTAATTTCGCTGGACTGAAGGTTCTTGTCATGGATGAGAATGGGGTAAGTAGAATGGTGACGAAGGGACTTCTTGTACACCTTGGATGCGAAGTGGCCACGGTGAGTTCAAGCGAGGAGTGTCTCAGAGTTGTATCCCATGAGCACAGAGTGGTCTTCATGGACGTGTGCACCCCCGGGGTCGAAAACTACCAGATCGCTCTCCGTATACACGAGAAATTCACAAAACGCCACCAAAGGCCACTGCTCGTGGCGCTCACTGGTAACACCGACAAATCCACAAAGGAGAGATGTATGAGCTTTGGTCTAGACGGCGTGTTGCTCAAACCCGTGTCGCTAGACAACATGAGAAACGTTCTGTCTGATCTTCTAGAACATCGGGTTCTGTACGAGGCCATGTAA

>BrHK11(Bra004449)-DNA

ATGGAGCCGTGTGATTGTTTCGAGACGAATGCGAATCAGGACGATCTGTTAGTGAAATACCAATACATCTCGGACGCGCTAATCGCCCTCGCCTACTTCTCAATCCCACTAGAGCTCATCTACTTCGTCAACAAGTCAGCCTTCTTCCCTTACAAATGGGTCCTGATGCAGTTCGGCGCCTTCATCATCCTCTGCGGCGCCACCCACTTCATCAACCTCTGGATGTTCTTCAACCACTCCAAAGTCGTCGCCATCGTCATGACCCTCGCCAAAGTCTCCTGCGCCGCCGTCTCCTGCGCCACCGCCCTCATGCTCGTTCACATCATCCCTGATCTTCTCAGCGTCAAGAACCGTGAGCTGTTCCTCAAGAAGAAGGCCGACGAGCTTGACAGAGAGATGGGTCTGATTCTCACCCAGGAGGAGACCGGGAGGCACGTGAGGATGCTTACTCATGAGATCAGAAGCACTCTTGATAGACACACTATCCTAAGGACCACGCTTGTGGAGCTTGGTAAGACTCTTTGCCTCGAGGAGTGCGCCTTGTGGATGCCCTCGCAGAGTGGTTTGTATCTTCAGCTTTCTCATACTTTGAGCCATAAGATACAAGTCGGAAGCAGTGTGCCTATTAATCTCCCGATTATTAACCAGCTCTTCAACAGCGCTCAAGCGATGCACATACCTCACACGTGTCCCTTGGCTAAGATTGGGCCTCCTGTTGGGAGATATGCGCCTCCTGAGGTTGTCTCTGTCCGTGTACCTCTTCTGCATCTCTCGAATTTCCAGGGAAGTGACTGGTCTGATCTCTCTGGTAAAGGCTACGCTATCATGGTCCTGATTCTTCCAACTGATGGTGCGAGAAAATGGAGAGACCATGAGCTGGAACTTGTCGAAGTTGTGGCCGATCAGGTCCATATCTTCATAATATGTTTTTGATTTTTGCAATCTGTGTCTCTGACTCTCGCTTTATTGATTTCAGGTGGCTGTGGCTCTTTCACACGCAGCGATTCTGGAGGAATCAATGCACGCTCGTGACCAGCTTATGGAGCAGAACTTTGCGTTAGACAAGGCTCGTCAAGAGGCAGAGATGGCAGTGCATGCTCGGAATGACTTCCTAGCTGTCATGAACCACGAGATGAGGACACCGATGCATGCCATCATCTCTCTCTCTTCTCTCCTTCTTGAGACCGAGCTATCTCCAGAGCAGAGAGTTATGATCGAGACTATACTGAAAAGTAGCAATCTCGTGGCTACACTCATCAGCGACGTTCTGGATCTGTCGAGATTGGAGGATGGGAGCTTGCTCTTGGAAAACGAACCATTCAGTCTCCAAGCCATCTTTGAAGAGGTAACTACTTATTATATAGATTAAGCAGTGAAGTTTATTGTCTTACACAAAAGCTTTGAATCTGTAGGTCATCTCTCTGATAAAGCCAATCGCGTCGGTGAAGAAACTATCGACGAATCTGATCCTATCTGCAGACTTGCCGGCGTATGCTATAGGTGATGAGAAACGTCTGATGCAAACTATGCTCAACATCATGGGGAACGCTGTGAAATTCACCAAGGAAGGCCACGTCTCCATAATAGCTTCTATCATGAAACCTGAGTCCTTGAGAGAATTACCATCTCCAGACTTCTATCCAGTTCCAAGCGACAATCACTTCTACCTATGCGTGCAGGTTAGTCCCAACCAATCCACATATAGTTATTTGCTTCACAGTTCGATCTGTTTCTAAAAAATGTTAATGATATGGGTGCATGTGAAGGCCACAGTGTGTGGAATTCACACGCACGACATTCCTTTTCTCTTCACCAAATTTGTGCAGCCTCGGACTGGAGCTCAGAGGAACCATGCGGGTGCAGGACTCGGTCTAGCTCTCTGCAAACGGTAATGCACTCAAAAGTATATCATGTATTAATATGAAGTGTAAAGCATGTGGTGGTGTAACAAAAGAGCTAAAGAGTGAGAGCATTTGATCTTTGGCTAGGTTCGTTGGGCTAATGGGAGGATGCATATGGATAGAAAGCGAAGGGATAGAGAAAGGCTGCACGGCTTCGTTCATAATAAGGCTGGGTATCTGCAACGGTCCTGGCAGTAGCAGCGGTTCAATGGCGCTACGTCTTGCAGCTAAATCACAAACAAGACCATGGAACTGGTGA

>BrHK11(Bra004449)-CDS

ATGGAGCCGTGTGATTGTTTCGAGACGAATGCGAATCAGGACGATCTGTTAGTGAAATACCAATACATCTCGGACGCGCTAATCGCCCTCGCCTACTTCTCAATCCCACTAGAGCTCATCTACTTCGTCAACAAGTCAGCCTTCTTCCCTTACAAATGGGTCCTGATGCAGTTCGGCGCCTTCATCATCCTCTGCGGCGCCACCCACTTCATCAACCTCTGGATGTTCTTCAACCACTCCAAAGTCGTCGCCATCGTCATGACCCTCGCCAAAGTCTCCTGCGCCGCCGTCTCCTGCGCCACCGCCCTCATGCTCGTTCACATCATCCCTGATCTTCTCAGCGTCAAGAACCGTGAGCTGTTCCTCAAGAAGAAGGCCGACGAGCTTGACAGAGAGATGGGTCTGATTCTCACCCAGGAGGAGACCGGGAGGCACGTGAGGATGCTTACTCATGAGATCAGAAGCACTCTTGATAGACACACTATCCTAAGGACCACGCTTGTGGAGCTTGGTAAGACTCTTTGCCTCGAGGAGTGCGCCTTGTGGATGCCCTCGCAGAGTGGTTTGTATCTTCAGCTTTCTCATACTTTGAGCCATAAGATACAAGTCGGAAGCAGTGTGCCTATTAATCTCCCGATTATTAACCAGCTCTTCAACAGCGCTCAAGCGATGCACATACCTCACACGTGTCCCTTGGCTAAGATTGGGCCTCCTGTTGGGAGATATGCGCCTCCTGAGGTTGTCTCTGTCCGTGTACCTCTTCTGCATCTCTCGAATTTCCAGGGAAGTGACTGGTCTGATCTCTCTGGTAAAGGCTACGCTATCATGGTCCTGATTCTTCCAACTGATGGTGCGAGAAAATGGAGAGACCATGAGCTGGAACTTGTCGAAGTTGTGGCCGATCAGGTGGCTGTGGCTCTTTCACACGCAGCGATTCTGGAGGAATCAATGCACGCTCGTGACCAGCTTATGGAGCAGAACTTTGCGTTAGACAAGGCTCGTCAAGAGGCAGAGATGGCAGTGCATGCTCGGAATGACTTCCTAGCTGTCATGAACCACGAGATGAGGACACCGATGCATGCCATCATCTCTCTCTCTTCTCTCCTTCTTGAGACCGAGCTATCTCCAGAGCAGAGAGTTATGATCGAGACTATACTGAAAAGTAGCAATCTCGTGGCTACACTCATCAGCGACGTTCTGGATCTGTCGAGATTGGAGGATGGGAGCTTGCTCTTGGAAAACGAACCATTCAGTCTCCAAGCCATCTTTGAAGAGGTCATCTCTCTGATAAAGCCAATCGCGTCGGTGAAGAAACTATCGACGAATCTGATCCTATCTGCAGACTTGCCGGCGTATGCTATAGGTGATGAGAAACGTCTGATGCAAACTATGCTCAACATCATGGGGAACGCTGTGAAATTCACCAAGGAAGGCCACGTCTCCATAATAGCTTCTATCATGAAACCTGAGTCCTTGAGAGAATTACCATCTCCAGACTTCTATCCAGTTCCAAGCGACAATCACTTCTACCTATGCGTGCAGGCCACAGTGTGTGGAATTCACACGCACGACATTCCTTTTCTCTTCACCAAATTTGTGCAGCCTCGGACTGGAGCTCAGAGGAACCATGCGGGTGCAGGACTCGGTCTAGCTCTCTGCAAACGGTTCGTTGGGCTAATGGGAGGATGCATATGGATAGAAAGCGAAGGGATAGAGAAAGGCTGCACGGCTTCGTTCATAATAAGGCTGGGTATCTGCAACGGTCCTGGCAGTAGCAGCGGTTCAATGGCGCTACGTCTTGCAGCTAAATCACAAACAAGACCATGGAACTGGTGA

>BrHKL1(Bra023756)-DNA

ATGATGGCGAGAGAAGTAGCTTCTGGGTTATTGATACTATTCTCGATACTGATCTGTGTTTCTCCGGCGGCGGCTGGAAACGGCGGCGGGTGTAACTGCGAAGACGAAGGAGTAAGCTTCTGGAGCACAGAGAACATCCTTGAGACGCAACGAGTAAGCGACTTCTTGATCGCAGTCGCTTACTTCTCAATCCCGATCGAGTTACTCTACTTCGTGAGCTGCTCCAACGTCCCATTCAAATGGGTCCTCTTCGAGTTCATCGCCTTCATTGTCCTCTGCGGTATGACTCATCTCCTCCACGGTTGGACTTACGGTCCCCACCCTTTCAAGCTCATGGTGGCGTTGACCGTTTTCAAGATGTTGACCGCTCTCGTCTCCTGCGCCACTGCCATCACCCTCATCACTCTCATCCCTCTCCTTCTCAAAGTTAAGGTTCGAGAGTTTATGCTCAAGAAGAAGGCTCATGAGCTTGGACGTGAAGTTGGTCTCATTATGATTCAGAAAGAGACCGGCGTTCACGTCCGTATGCTTACTCAAGAGATACGCAAGTCCTTGGACCGTCACACGATTCTTTACACCACTTTGGTTGAGCTTTCGAAGACTTTAGCGTTGCAGAACTGTGCCGTGTGGATGGAGAATGAAGGTAAGAGTGAGATGAATCTGACTCATGAGCTTAGAGGGAGCTCAGGGAGGAGTGGTTATGGCTACTCTGTTTCTATGCATGATGTGGACGTTGTTAGGGTTAGAGAAAGCAATGATGTGAATATACTAAGCGTTGACTCGTTGATAGCTCGAGCTAGCGGCGGAGATGTGAGTGAGATTGGACCGGTGGCTGCCATTAGAATGCCGATGCTTCGCGTGTCGGATTTCAAAGGAGGGACGCCTGAGTTGATTCAGACATGTTACGCCATACTCGTCTGCGTGTTACCTAGCGGACAGCCTCGGGACTGGAGTTATCAGGAGATTGAGATTGTTAAGGTCGTGGCTGATCAAGTAGCCGTTGCGTTGTCTCACGCAGCGGTTCTTGAGGAGTCTCAGATGATGAGGGACAAGCTCGCGGACCAGAACCGGGCCTTGCAGATCGCGAAGAGGGACGCGATGAGGGCGAGCCAGGCGAGGAATGTGTTGCAGAAAGCGATGAGCGAAGGGATGAGACGTCCGATGCATTCTATACTTGGTCTTTTGTCGATGATACAGGACGAGAAGTTGAGTAATGAACAGAAGATGATTGTTGATACGATGGTGAAGACCGGGAATGTTATGTCGAATTTGGTCGGGGACGCGATGGATGTTTCGGACGGTAGATTTGTCACGGAGATGAAGCCGTTTAGTCTGCACCGCACGGTCCGTGAAGCGGCTTGCTTGGCGAGGTGTTTGTGTCTTTACAATGGGTTTCGGTTTACGGTTGATGCGGAGAAGTCTCTGCCTGATAATGTAGTGGGAGATGAAAGAAGAGTGTTTCAGGTGATACTTCATATGGTTGGTAGTTTAGTAAAGCCTAGAAAGTGTCGAGAAGGATCCTTGTCGTCGTCGGTGATCTTTAAGGTTTTTAAAGAAAGAGGAAGCTTGGATAGGAGTGATCAAAGATGGGCTGCATGGAGATCACCTACTTGTTCGGCGGATGGAGATGTGTACATAAGATTCGAAATGAGTGTTGAGAATGATGGTTCAGGCTCTCAATCATTTGCTTCTGTTTCGTCAAGAGATCAAGAAGTTGGCGAAGTGAGATTATCTGGCTATGGGTTAGGACAAGATCTAAGCTTTGGTGTTTGTAAGAAAGTGGTGGAGGTATGTTTCTATAGCTGTCGTTATTAGTGTGGGAAGAAAGAGTATTTATCTCGCATTCTTTTATAACCTATGTGCAGTTAATTAAAGGGAACATCTCGGTGGTCCCTGGATCGGACGGCTCACCGGAAACCATGTCACTGCTCCTCAGGTTTCGACGCCGGCCGTCCATATCAGTCCATGGAATAGGGGAAGCACCGGCTCTAGACCACCACCTTCACCCTCATTCTGATTCTCTATTACGTGGCTTGCAAGTTTTATTAGTAGACACCAACGATTCAAACCGGGCGGTTACGCGTAAACTTTTAGAAAAACTTGGCTGCATTGTAACCGCGGTATCATCTGGATACGATTGCCTCACTGCCATTGCACCTAGCTCTTCCTCATCTTCCCCGACGTTCCAAGTGGTGGTGCTTGATCTACAAATGGCAGAGATGGATGGCTATGAAGTTGCCATGAGGATCAGGAGCCGATCTTGGCCGTTGATTGTCGCAATGACCGTGAGCTTGGACGAAGAAATGTGGGACAAGTGTATGCAGATTGGGATCAATGGAGTTGTGAGAAAGCCGGTCATGTTAAGAGCTATGGAGAGTGAGCTCCGGAGAGTATTGTTGCAAGCTGACCAACTTCTCTAG

>BrHKL1(Bra023756)-CDS

ATGATGGCGAGAGAAGTAGCTTCTGGGTTATTGATACTATTCTCGATACTGATCTGTGTTTCTCCGGCGGCGGCTGGAAACGGCGGCGGGTGTAACTGCGAAGACGAAGGAGTAAGCTTCTGGAGCACAGAGAACATCCTTGAGACGCAACGAGTAAGCGACTTCTTGATCGCAGTCGCTTACTTCTCAATCCCGATCGAGTTACTCTACTTCGTGAGCTGCTCCAACGTCCCATTCAAATGGGTCCTCTTCGAGTTCATCGCCTTCATTGTCCTCTGCGGTATGACTCATCTCCTCCACGGTTGGACTTACGGTCCCCACCCTTTCAAGCTCATGGTGGCGTTGACCGTTTTCAAGATGTTGACCGCTCTCGTCTCCTGCGCCACTGCCATCACCCTCATCACTCTCATCCCTCTCCTTCTCAAAGTTAAGGTTCGAGAGTTTATGCTCAAGAAGAAGGCTCATGAGCTTGGACGTGAAGTTGGTCTCATTATGATTCAGAAAGAGACCGGCGTTCACGTCCGTATGCTTACTCAAGAGATACGCAAGTCCTTGGACCGTCACACGATTCTTTACACCACTTTGGTTGAGCTTTCGAAGACTTTAGCGTTGCAGAACTGTGCCGTGTGGATGGAGAATGAAGGTAAGAGTGAGATGAATCTGACTCATGAGCTTAGAGGGAGCTCAGGGAGGAGTGGTTATGGCTACTCTGTTTCTATGCATGATGTGGACGTTGTTAGGGTTAGAGAAAGCAATGATGTGAATATACTAAGCGTTGACTCGTTGATAGCTCGAGCTAGCGGCGGAGATGTGAGTGAGATTGGACCGGTGGCTGCCATTAGAATGCCGATGCTTCGCGTGTCGGATTTCAAAGGAGGGACGCCTGAGTTGATTCAGACATGTTACGCCATACTCGTCTGCGTGTTACCTAGCGGACAGCCTCGGGACTGGAGTTATCAGGAGATTGAGATTGTTAAGGTCGTGGCTGATCAAGTAGCCGTTGCGTTGTCTCACGCAGCGGTTCTTGAGGAGTCTCAGATGATGAGGGACAAGCTCGCGGACCAGAACCGGGCCTTGCAGATCGCGAAGAGGGACGCGATGAGGGCGAGCCAGGCGAGGAATGTGTTGCAGAAAGCGATGAGCGAAGGGATGAGACGTCCGATGCATTCTATACTTGGTCTTTTGTCGATGATACAGGACGAGAAGTTGAGTAATGAACAGAAGATGATTGTTGATACGATGGTGAAGACCGGGAATGTTATGTCGAATTTGGTCGGGGACGCGATGGATGTTTCGGACGGTAGATTTGTCACGGAGATGAAGCCGTTTAGTCTGCACCGCACGGTCCGTGAAGCGGCTTGCTTGGCGAGGTGTTTGTGTCTTTACAATGGGTTTCGGTTTACGGTTGATGCGGAGAAGTCTCTGCCTGATAATGTAGTGGGAGATGAAAGAAGAGTGTTTCAGGTGATACTTCATATGGTTGGTAGTTTAGTAAAGCCTAGAAAGTGTCGAGAAGGATCCTTGTCGTCGTCGGTGATCTTTAAGGTTTTTAAAGAAAGAGGAAGCTTGGATAGGAGTGATCAAAGATGGGCTGCATGGAGATCACCTACTTGTTCGGCGGATGGAGATGTGTACATAAGATTCGAAATGAGTGTTGAGAATGATGGTTCAGGCTCTCAATCATTTGCTTCTGTTTCGTCAAGAGATCAAGAAGTTGGCGAAGTGAGATTATCTGGCTATGGGTTAGGACAAGATCTAAGCTTTGGTGTTTGTAAGAAAGTGGTGGAGTTAATTAAAGGGAACATCTCGGTGGTCCCTGGATCGGACGGCTCACCGGAAACCATGTCACTGCTCCTCAGGTTTCGACGCCGGCCGTCCATATCAGTCCATGGAATAGGGGAAGCACCGGCTCTAGACCACCACCTTCACCCTCATTCTGATTCTCTATTACGTGGCTTGCAAGTTTTATTAGTAGACACCAACGATTCAAACCGGGCGGTTACGCGTAAACTTTTAGAAAAACTTGGCTGCATTGTAACCGCGGTATCATCTGGATACGATTGCCTCACTGCCATTGCACCTAGCTCTTCCTCATCTTCCCCGACGTTCCAAGTGGTGGTGCTTGATCTACAAATGGCAGAGATGGATGGCTATGAAGTTGCCATGAGGATCAGGAGCCGATCTTGGCCGTTGATTGTCGCAATGACCGTGAGCTTGGACGAAGAAATGTGGGACAAGTGTATGCAGATTGGGATCAATGGAGTTGTGAGAAAGCCGGTCATGTTAAGAGCTATGGAGAGTGAGCTCCGGAGAGTATTGTTGCAAGCTGACCAACTTCTCTAG

>BrHKL2(Bra040134)-DNA

ATGTTAAGATCCTTAGGTCTAGGCTTGCTTCTCTTCGCCCTAATCGCTCTCGTCACCGGAGACAACAACGACTACGGAAGCTGCAACTGCGACGACGAAGGCTACTACTTCTTCACCGTCCACACAATCCTAGAATGCCAGAGAGTGAGCGATCTCCTAATCGCCATCGCCTACTTCTCCATCCCTCTCGAGCTTCTCTACTTCATCAGCTTCTCCAACGTCCCTTTCAAATGGGTCCTCGTACAGTTCATCGCCTTCATCGTCCTCTGCGGTATGACTCACCTCCTCAACGCCTGGACTTACTATGGCCCCCACTCGTTCCAGCTGATGCTATGGCTCACAATCTTTAAGTTCTTGACGGCGTTGGTCTCGTGCGCGACGGCGATTACTCTCTTGACTTTGATCCCTTTGCTACTGAAGTGGAAAGTGAGAGAGCTTTACTTGAAGCAGAACGTGCTTGAGCTTAACGAGGAGGTTGGGCTGATGAAACGGCAGAAGGAGATGAGTGTTCACGTTAGGATGCTTACTAGAGAGATTAGGAAGTCTCTTGATAAGCATATGATACTGAGGACGACGCTTGTTGAGTTGTCAAAGATTCTTGATTTGCAGAACTCCGCTGTCTGGATGCCTAACGAGAACAGAACAGAGATGCATTTGACTCATGAGCTGAGGTCGAATTCTATGAGGAGCTTCAGAGTGGTGCCGATCAACGATCCTGACGTTGTTCAGGTCAGGGAGGCGAAAGTGGTTGCGCTATTGAGGAAAGATTCTCTTCTTGCGGTCGAGAGCAGTGGTTCTGATGAGAGTGGACCCGTGGCGGCTATTCGGATGCCGATGCTCCACGGGTCGAATTTCAAAGGAGGGACTCCTGAGTTTGTTGATACTTCCTACGCTATAATGGTTCTGGTCCTTCCAAACGCGAACTCTCGCGTTTGGACGGACAGAGAGATCGAGATAGCTGAGGTCGTTGCTGACCAAGTGGCTGTGGCTCTCTCGCACGCCTCGGTGCTGGAGGAGTCGCAGCTGATGAGAGAAAAGCTCGGTATTCAGAACCGAGCTTTGCTCCGAGCGAAACAGAACGCGATGATGGCCAGCCAGGCGAGGAACACTTGCCAAAAGGTCATGAGTCACGGCATGAGGAGGCCGATGCACACGATCCTCGGCCTCCTCTCGATGTTCCAGTCTGAGAGCATGAGCCTCGACCAGAAGATCATCGTCGACGCGCTCATGAAAACGAGTACTGTCCTCTCTGCTTTGATCAACGACGTGATAGACATTTCTCCTAAAGACAACGGGAAGTCTCCGTTGGAGGTCAAAAGGTTTCAGCTCCATTCTCTGATAAGAGAAGCAGCCTGCGTGGCGAAATGCTTGAGCGTTTACAAAGGCTACGGGTTCGAGATGGATGTTCAGACGCGTCTGCCTAGCTTAGTGGTGGGAGATGAGAAGAGGACGTTTCAGCTTGTGATGTATATGCTTGGGTATATTTTGGATATGAGTGAAGGAGGGAAGACGGTTACGTTTAGGGTTGTGTCTGAAGGGACTGGGAGTAGTCAGGACAAGAACAAGAGAGAGAGTGGGATGTGGAAGTCGCATTTGTCTGATGATTCGCTCGGTGTGAAGTTTGAAGTTGAGATTAATGAGATTAAGAGTCCTCCGTTGGATGGTTCGGTGATTGCAATGAGGCATGTTACAAACAGAAGGTATCATAGCAATGGGATTAAAGAAGGCTTGAGCTTAGGCATGTGTAGAAAACTTGCACAGGTTAGTGAATCACATATATACATCTGTTTTTATTTTTCTAATACAGTCTCTGTATATGTGATAGGCATGGGCGTTTGGATTTTCGGTACTATGTTCATAAAAGTTCAGTTTTCAATAATTTTCGGATCAGTTTCAGTTTGGATCCTTTCGGATTCAGTTTGGATCAGGTTATAACCGATGTCTGAAATTGTTCAAAAAAATTTAAATGAAATTTAAAAAAATTGACCAAAAAGACACAGAATATATAAATTATTCAAAATCTATATACAAACTAGTTAAACTACCTAATAAGTATTGAAAATAACAAATAGAACAAACTACAAGAATCTTTAAAATGCTTTAAAATATCTAAAAACATTATATGTTTAACTAATTTCGGATATTTTCGGATCCTAATTTGGATTTCGGTTCGGATTATTAAGTTTTGAACCCAAAATTAGCTAGCTCTTAAATTTCGTTCGGATATTTTTTATAGCGGATAGGATTTCGTTTTTTTTCGGTTTGGATTAAGGTTCGGTTTTGGTTTGGGATAAAAATGCCCAGCCTAATATGTAATTTGATATTTGTCTGCAGATGATGCAAGGGAACATATGGATATCACCAAAGTCTCATGGACAAACACAGAGTATGCAACTGGTTCTGAGGTTTCAGACAAGACCTTCTATTAGAAGATCAATCTTAGCAGGAAACGCTCCAGAGCTTCAACATCCAAACTCAAACTCGATTCTTCGTGGTCTAAGAATCACTCTAGCAGATGATGATGATGTGAACAGAACCGTAACCAAGAGGCTTTTAGAGAAACTAGGATGTGAAGTGACAGCTGTGTCTTCTGGTTTCGAGTGTCTGAGTGCCTTGTCTAATGTGGAAATGTCGTATAGGGTTGTGATATTGGATCTGCAGATGCCTGAGATGGATGGTTTCGAAGTAGCGATGAAGATAAGGAAGTTTTGTGGACATCATTGGCCGTTGATTATAGCTTTGACTGCGAGTACTGAGGATCATGTTAGGGAGAGGTGTTTGCAGATGGGGATGAATGGTATGATTCAGAAACCTGTTCTTTTGCATGTCATGGCTTCTGAGCTTAGAAGGGCTTTACAGAGCGCAAGCGAGTGA

>BrHKL2(Bra040134)-CDS

ATGTTAAGATCCTTAGGTCTAGGCTTGCTTCTCTTCGCCCTAATCGCTCTCGTCACCGGAGACAACAACGACTACGGAAGCTGCAACTGCGACGACGAAGGCTACTACTTCTTCACCGTCCACACAATCCTAGAATGCCAGAGAGTGAGCGATCTCCTAATCGCCATCGCCTACTTCTCCATCCCTCTCGAGCTTCTCTACTTCATCAGCTTCTCCAACGTCCCTTTCAAATGGGTCCTCGTACAGTTCATCGCCTTCATCGTCCTCTGCGGTATGACTCACCTCCTCAACGCCTGGACTTACTATGGCCCCCACTCGTTCCAGCTGATGCTATGGCTCACAATCTTTAAGTTCTTGACGGCGTTGGTCTCGTGCGCGACGGCGATTACTCTCTTGACTTTGATCCCTTTGCTACTGAAGTGGAAAGTGAGAGAGCTTTACTTGAAGCAGAACGTGCTTGAGCTTAACGAGGAGGTTGGGCTGATGAAACGGCAGAAGGAGATGAGTGTTCACGTTAGGATGCTTACTAGAGAGATTAGGAAGTCTCTTGATAAGCATATGATACTGAGGACGACGCTTGTTGAGTTGTCAAAGATTCTTGATTTGCAGAACTCCGCTGTCTGGATGCCTAACGAGAACAGAACAGAGATGCATTTGACTCATGAGCTGAGGTCGAATTCTATGAGGAGCTTCAGAGTGGTGCCGATCAACGATCCTGACGTTGTTCAGGTCAGGGAGGCGAAAGTGGTTGCGCTATTGAGGAAAGATTCTCTTCTTGCGGTCGAGAGCAGTGGTTCTGATGAGAGTGGACCCGTGGCGGCTATTCGGATGCCGATGCTCCACGGGTCGAATTTCAAAGGAGGGACTCCTGAGTTTGTTGATACTTCCTACGCTATAATGGTTCTGGTCCTTCCAAACGCGAACTCTCGCGTTTGGACGGACAGAGAGATCGAGATAGCTGAGGTCGTTGCTGACCAAGTGGCTGTGGCTCTCTCGCACGCCTCGGTGCTGGAGGAGTCGCAGCTGATGAGAGAAAAGCTCGGTATTCAGAACCGAGCTTTGCTCCGAGCGAAACAGAACGCGATGATGGCCAGCCAGGCGAGGAACACTTGCCAAAAGGTCATGAGTCACGGCATGAGGAGGCCGATGCACACGATCCTCGGCCTCCTCTCGATGTTCCAGTCTGAGAGCATGAGCCTCGACCAGAAGATCATCGTCGACGCGCTCATGAAAACGAGTACTGTCCTCTCTGCTTTGATCAACGACGTGATAGACATTTCTCCTAAAGACAACGGGAAGTCTCCGTTGGAGGTCAAAAGGTTTCAGCTCCATTCTCTGATAAGAGAAGCAGCCTGCGTGGCGAAATGCTTGAGCGTTTACAAAGGCTACGGGTTCGAGATGGATGTTCAGACGCGTCTGCCTAGCTTAGTGGTGGGAGATGAGAAGAGGACGTTTCAGCTTGTGATGTATATGCTTGGGTATATTTTGGATATGAGTGAAGGAGGGAAGACGGTTACGTTTAGGGTTGTGTCTGAAGGGACTGGGAGTAGTCAGGACAAGAACAAGAGAGAGAGTGGGATGTGGAAGTCGCATTTGTCTGATGATTCGCTCGGTGTGAAGTTTGAAGTTGAGATTAATGAGATTAAGAGTCCTCCGTTGGATGGTTCGGTGATTGCAATGAGGCATGTTACAAACAGAAGGTATCATAGCAATGGGATTAAAGAAGGCTTGAGCTTAGGCATGTGTAGAAAACTTGCACAGATGATGCAAGGGAACATATGGATATCACCAAAGTCTCATGGACAAACACAGAGTATGCAACTGGTTCTGAGGTTTCAGACAAGACCTTCTATTAGAAGATCAATCTTAGCAGGAAACGCTCCAGAGCTTCAACATCCAAACTCAAACTCGATTCTTCGTGGTCTAAGAATCACTCTAGCAGATGATGATGATGTGAACAGAACCGTAACCAAGAGGCTTTTAGAGAAACTAGGATGTGAAGTGACAGCTGTGTCTTCTGGTTTCGAGTGTCTGAGTGCCTTGTCTAATGTGGAAATGTCGTATAGGGTTGTGATATTGGATCTGCAGATGCCTGAGATGGATGGTTTCGAAGTAGCGATGAAGATAAGGAAGTTTTGTGGACATCATTGGCCGTTGATTATAGCTTTGACTGCGAGTACTGAGGATCATGTTAGGGAGAGGTGTTTGCAGATGGGGATGAATGGTATGATTCAGAAACCTGTTCTTTTGCATGTCATGGCTTCTGAGCTTAGAAGGGCTTTACAGAGCGCAAGCGAGTGA

>BrHKL3(Bra015303)-DNA

ATGTTGAAGACATTGTTACTTCACGTGCTTTTGTTCTTCTTCTTCTTAACCGCCTCCGTCGCCGGCAGCTTGTCGATATGCAACTGCGACGACGAGGACAGTTTCTTCACCTTTGAGGCAATCCTCCAATCCCAAAAGGCCGGCGACTTTCTAATCGCAGTCGCCTACTTCTCCATCCCAATCGAGCTCCTCTACTTCGTCAGCCGCACCAACGTGCCTTCCCCTTACAACTGGGTCGTCTGCGAGTTCATAGCCTTCATCGTCCTCTGCGGCATGACCCATCTCCTCGCGGGCTTCACCTACGGGCCTCACTGGGCCTGGGTCACGACAGCTGTCACCGTCTTCAAAATGCTGACTGGGATCGTCTCATTCCTCACGGCCATCTCGCTCGTCACTCTCTTGCCTTTGCTTTTAAAAGCAAAGGTTCGGGAGTTTATGCTGAGTAAGAAGACGAGAGAGCTTGGCCGTGAGGTTGGGATTATAATGAAGCAGACGGAGACGAGTTTGCATGTACGTATGCTCACCAGCAAGATAAGGACGTCGTTGGATAGGCACACGATACTGTACACCACGCTTGTGGAGTTGTCGAAGACTTTGGGGTTAAAGAACTGCGCGGTTTGGATACCTAATGAGATTAAAACGGAGATGAATCTTACTCACGAGTTGAGACCGGGGCACCACGATGGTGGCGGCGGTGGTGGTTATGGTGGTGGGTTTTCGATACCGATCACTGAGTCTGATGTTGTGAGGATTAAGAGGAGTGAGGAAGTGAATATGTTGAACTCTGGCTCTGCGCTTGCCTCGGTTACTAGCCGAGGCAAGCCAGACGGCCAGACGGTTGGGATCAGAGTCCCGATGCTTCGTGTTTGTAACTTTAAAGGCGGGACGCCCGAGGCGATCCACATGTGTTACGCCATCTTGGTTTGTGTGCTTCCCTCGCGGAGCTGGACTTACCAAGAGCTGGAGATCGTTAAAGTTGTGGCTGATCAAGTCGCGGTCGCCATCTCTCATGCTGTGATTCTAGAAGAATCACAGCTCATGAGAGAGAAGCTCGCGGAACAGAACCGCGCGCTTCAGGTGGCGAGGGAGAACGCGCTTAGAGCTAACCAGGCCAAAGCTGCGTTCGAGGAGATGATGGGGGATGCGATGAGGCGTCCTGTTCGGTCCATCCTCGGCTTGTTGCCTGTGATAGCGCGAGACGGGGGATTACAAGAGAACCAAAAGGTTATCGTCGATGCGATGGGGAGAACCAGCGAGCTATTGCTGCACCTCGTGAGCAATGCTGGGGATATAACGCGTCCTGGAGAGACGCATTGTTTCAGTTTGCGTTCTGTTGCGAAGGAAACGGCTTGCTTGGCGAGGTGTTTCTGCGTGGGGAACGGGTTTGGTTTCTCGACGGAGGTTGATAGATCATTGCCTGATTACGTCGTGGGCGATGCTAGAAAGGTGTTTCAAGTGGTCTTGCATATGCTAGGAGGTTTGGTGAACCGGAATATCAAAGGGAATGTGACTTTCTTGGTTTCACCGGAAAGCTCAGAAGTAGATAGCCAAGAAGCGGTATGGCGACAATGCTATTCGAAAGAATACATCAAAGTTAAATTCGGATTCGAGGTAGAGACTAGTAGCGTCCCGAGCTTCAACATCAGTGAAGATATTGTAAAGGTTAGACATGTTTTTTTTATTTTGTTGTTGTCTGCTATAGTATGTAAAAATATGTTAATGGGGATCTTTCTTGGTGTGTGGACACAGTTAATGCAAGGAAACATTAGGGTGGTGGAGGATGGTTCAGGTTTGGTGAAAAGCCTCTCTGTTGCTTTCAGGTTCCAGCTCCGGGGATCTATGCTGTCTCAAGGCGGTGGATATTCTGGAGAAACTTTTAAAACGGCAACTCCTCCATCTACTAGTAACGATCATTGGCGTCAGGAAGAGATCAGATAA

>BrHKL3(Bra015303)-CDS

ATGTTGAAGACATTGTTACTTCACGTGCTTTTGTTCTTCTTCTTCTTAACCGCCTCCGTCGCCGGCAGCTTGTCGATATGCAACTGCGACGACGAGGACAGTTTCTTCACCTTTGAGGCAATCCTCCAATCCCAAAAGGCCGGCGACTTTCTAATCGCAGTCGCCTACTTCTCCATCCCAATCGAGCTCCTCTACTTCGTCAGCCGCACCAACGTGCCTTCCCCTTACAACTGGGTCGTCTGCGAGTTCATAGCCTTCATCGTCCTCTGCGGCATGACCCATCTCCTCGCGGGCTTCACCTACGGGCCTCACTGGGCCTGGGTCACGACAGCTGTCACCGTCTTCAAAATGCTGACTGGGATCGTCTCATTCCTCACGGCCATCTCGCTCGTCACTCTCTTGCCTTTGCTTTTAAAAGCAAAGGTTCGGGAGTTTATGCTGAGTAAGAAGACGAGAGAGCTTGGCCGTGAGGTTGGGATTATAATGAAGCAGACGGAGACGAGTTTGCATGTACGTATGCTCACCAGCAAGATAAGGACGTCGTTGGATAGGCACACGATACTGTACACCACGCTTGTGGAGTTGTCGAAGACTTTGGGGTTAAAGAACTGCGCGGTTTGGATACCTAATGAGATTAAAACGGAGATGAATCTTACTCACGAGTTGAGACCGGGGCACCACGATGGTGGCGGCGGTGGTGGTTATGGTGGTGGGTTTTCGATACCGATCACTGAGTCTGATGTTGTGAGGATTAAGAGGAGTGAGGAAGTGAATATGTTGAACTCTGGCTCTGCGCTTGCCTCGGTTACTAGCCGAGGCAAGCCAGACGGCCAGACGGTTGGGATCAGAGTCCCGATGCTTCGTGTTTGTAACTTTAAAGGCGGGACGCCCGAGGCGATCCACATGTGTTACGCCATCTTGGTTTGTGTGCTTCCCTCGCGGAGCTGGACTTACCAAGAGCTGGAGATCGTTAAAGTTGTGGCTGATCAAGTCGCGGTCGCCATCTCTCATGCTGTGATTCTAGAAGAATCACAGCTCATGAGAGAGAAGCTCGCGGAACAGAACCGCGCGCTTCAGGTGGCGAGGGAGAACGCGCTTAGAGCTAACCAGGCCAAAGCTGCGTTCGAGGAGATGATGGGGGATGCGATGAGGCGTCCTGTTCGGTCCATCCTCGGCTTGTTGCCTGTGATAGCGCGAGACGGGGGATTACAAGAGAACCAAAAGGTTATCGTCGATGCGATGGGGAGAACCAGCGAGCTATTGCTGCACCTCGTGAGCAATGCTGGGGATATAACGCGTCCTGGAGAGACGCATTGTTTCAGTTTGCGTTCTGTTGCGAAGGAAACGGCTTGCTTGGCGAGGTGTTTCTGCGTGGGGAACGGGTTTGGTTTCTCGACGGAGGTTGATAGATCATTGCCTGATTACGTCGTGGGCGATGCTAGAAAGGTGTTTCAAGTGGTCTTGCATATGCTAGGAGGTTTGGTGAACCGGAATATCAAAGGGAATGTGACTTTCTTGGTTTCACCGGAAAGCTCAGAAGTAGATAGCCAAGAAGCGGTATGGCGACAATGCTATTCGAAAGAATACATCAAAGTTAAATTCGGATTCGAGGTAGAGACTAGTAGCGTCCCGAGCTTCAACATCAGTGAAGATATTGTAAAGTTAATGCAAGGAAACATTAGGGTGGTGGAGGATGGTTCAGGTTTGGTGAAAAGCCTCTCTGTTGCTTTCAGGTTCCAGCTCCGGGGATCTATGCTGTCTCAAGGCGGTGGATATTCTGGAGAAACTTTTAAAACGGCAACTCCTCCATCTACTAGTAACGATCATTGGCGTCAGGAAGAGATCAGATAA

>BrHKL4(Bra030564)-DNA

ATGCTAAAGACATTGTTAGTTCACGGCCCTTTGCTCTTCTTCTTCTTCTTCTTGATCAGCTCCGTCGTCGCCGGCGATGAAAACGACGGCGGATTGTCGATATGCAACTGCGACGACGAAGACAGTTACTTCAGCTACGAAGGAATCCTCGAATCTCAAAAAGTCGGCGATTTTCTAATCGCCGTCGCCTACTTCTCCATCCCCATCGAGCTTCTCTACTTCGTGAGCCGCACCAACGTCTCTTCCCCTTACATCTGGGTCGTCTGCGAGTTCATAGCCTTCATCGTCCTCTGCGGCATGTCCCACTTGCTCTCCGGCTTCACCTACGGGCCCCACTACCCTTGGGTCATGACGGCCGCCACCGTCTTCAAAATGCTGACTGCGATCGTCTCTTTCCTCACAGCCATCTCGCTTGTCACTCTCTTACCGTTACTTCTTAAAGCTAAGGTTCGTGAGTTTATGTTGAGTAAGAAGACGAGAGAACTTAACCGTGAGGTTGGGCTTATAATGAAGCAGACGGAGACGAGTTTGCACGTGCGTATGCTCACTACTAAGATAAGGACGTCTTTGGATAGGCACACGATACTCTACACGACGCTTGTGGAGCTGTCGAAGACTCTGGGGCTCAAGAACTGCGCGGTTTGGATCCCTAATGAGATCAAGACGGAGATGAATCTCACGCACGAGTTGAACGGTGAGAATGTGGGTCGTGGGCCTGGTGGTGGGCCTAGTGGTGGGCCTGGTGGGTTTTCGATACCGATCACTGAGTCTGATGTTGTGAGGATTAAGAGAAGTGTGGAGGTGAATATGCTGAGTGCTGGCTCTGCTCTTGCCTCGGTTACTACCCGAGGCAAGTCAGGCCAGACGGTCGGGATCAGAGTCCCGATGCTTCGTGTTTGCAACTTCAAAGGTGGGACCCCTGAGGCTATCCACATGTGCTACGCCATCTTGGTCTGTGTGCTTCCTCTGAGGCGGAGCTGGAGTTACCAGGAGCTGGAGATCGTCAAGGTCGTGGCTGATCAAGTCGCGGTTGCGATCTCTCACGCGGTGATCCTCGAGGAGTCTCAGCTCATGAGGGAGAAGCTCGCGGAGCAGAACCGTGCGCTTCAGGTGGCGAGGGAGAACGCGATGAGAGCTAACCAAGCCAAAGCTGCGTTCGAGGAGATGATGGGGGATGCGATGAGGCGTCCCGTGAGGTCTATCCTCGAGCTGCTTCCTTTGATAACGCAGGACGGGGTGTCGTTACCCGAGACGCAGAAGGTTATCGTTGATGCTATGGGGAGAACCAGCGAGCTGCTGTTACACCTTGTGAACAATGCTGGGGATGTAGCTAGTGGGACCCATTGTTTTAGTTTGCGTTCGGTTGTGAAGGAAACGGCATGCTTGGCGAGGTGTTTGTGTTTGGGGAACGGGTTTGGTTTCACGACGGATGTTGATAGAGCGTTGCCTGATTGCGTCGTGGGCGATGCTAGGAAGGTGTTACAAGTGGTGTTGCATATGCTAGGAGGTGTAATGAACCGGAAGGTCAAAGGGAATGTGACGTTTAAGGTTGTACCGGAACGAGGAAGCTCAGAAGTTGTGAAAGAGAGCCAAGAAGCGGCTTGGCGACAATGCTATTCCAAAGAATACGTTGAAGTTAAGTTTGGATTTGATGTAGCTGCAGAAGGCGAAGAGAGTAGTAGTAGTAGCAGCAGCACTAAGGTTAGACATTTTTGTTTGTTTTTTTTTTTTGATGTTATAAAATATGTTAATAGGCTATTCCTTGTTTTGTGACAGTTCATGCAAGGGAACGTTTTGGTTGTGGAGGATGGTCTAGGTTTGGTGAAAAGCCTCTCTGTTGTGTTCCGGTTCCAGCTCCGGCGATCTATAGTGTCACGAGGCGGTGGATATTCGGGAGAAACTTTTAAAACATCGACTCCTCCGTCTACTAGTAACGGTCATTGGCGTCAGTAAGGGGGACATGTTAGAGTAGAAGAGAGGTGATTTAGGTGAAAGGTTAATAAATCAGAGAGAGAGAGAAGTTAAAAGTAAAAACAGTGTCGTGTTTTGGTTGTATAAACCTGAAAAAAATTAACTAGGTTTTAACCTATTATTATGTAGGCAAAAGGCATGGTTCAGACTTGTTACCTGGTATGGTAGATAACAGAATTAAAAAGTTGAATGTGTCGGAGGATGTTTTTTCCTCTTTATCTTCCTGTTAATTGTCACATCAGTGTCGTTAGATATTTGAAGGAAGAAAACTAATTAGACAAAACAAAAACGGATTAAGATTAACAACTTATTGCAAAGCCTATTAACTCCTTTGTAATATTACTAAAATATATTTTTTTTTGGGTGCACAACTAAAATAATGATATTTTAGATTAGCTAACTTTAAACATGGAATTGGATCGGACCACGTAAGCTCACCGGATTTTTCAAAACTTCATAATCTTGACTCTAACCAAGATTAAGCTTTTTCCTATTAAAAAGCTGTCTGAAACTCATACCATAAATATTTCTTTTGTCATATCTTTATAATCTGAGCAGAAATGTAATAAAAATCAACTAGTGATGAATTATTTCACTGTAAAAAAATATTATTTACTGTCCTCTTAAAACAAAATCCAATTAGACTAACGTAATTTTATTTTTCGGAATATTATGCGATTGGACGAGTGCGGCAACAGGAAAACATTATAATCAAAACAGTTAAGAGTTACTAATCTGGTTTATTTTATTTTAATATTTTTTAAGGTTTTTATTTGCTGGAGCTGTTTCGTGCGACATCTCGCTATTTATCTCTCTAATTCATTTTCTTCTTCCGTCGTCATTTTTTCGTTCTCTCGCTCGCTAGAATCTATTGGGAGCTGCTGCCATTCAACGAATAAACCTCTCTCTCTCTGCTCTTTCCTCCACCATCAACAGGTTCGCTTCGATCTACACGATTAACCCGTTTAAGCCCCTTCAATATTTTGTTCTGATTTCGCGATGATAGATAGTAGTCTATGTTTCCTCTCTAATCCGAGAGCCGTTTTTGCTTTCCCCGATCGTTGATTCCATCTTAGGTTTTAAATTAGGTTTAATTGAAATTGGGCGCGGGTTTCTATTGGAGATTGTAGCTGATTGAGAAATCAATTGGTCTTCCTCTAATTCTTCCAGCTGATGATGCAACCATCTTATTTTTGCGAGATTGAAATTTATTTAGGGTTTATTATCTGATGTTGGCTCACTGAGAAATGAAATTACGGATTTAGACTTTCACCTTTCTTCGCTGTGTTGATTAAATTGGCACAGTTCGAGAGAGTCAGATCGTTAGAGTATGATTCAGTTGTAAGCTTAGGAAAGAGTCAAGAGGCAGATGAATCATTTGCTTTTGTCAGCTAGTCTTATTCATTAGACTGATAACTTCTAAAATTATGTCTTATTAATTTATATGCTCCATTTTTAATTTTTTTTATAGTTTGAAGGAAGCTTTGAAGAAAAATCTGTACTGGGGAATGACAGAGACTGTTAATGAAGATTCTGGAGTGGGGCGATCAGTAGAGGCGAGTTCTAATGGACACCACAGTCTATCAGGAGAATCACTCTCACTCTCTAAGTGGCGGTCTTCTGCTCAGGTTGAGAATGGGACGCCTTCCACATCCCTCTCTTACTGGGACACCGATGATGATGAAGACCACGGTAGGTCTTTTTGCAGCAATATCTTTTTTTTCATGAATTATCATCTGGTTTTACACTCTGTAAGACTTGGTTTTGGGTATCTTCAAGATGAATTTCTTCATACTTTGTACAAACTGAAAGTGGTTTGACAGATGTGACTCAATGATGCAGTCCTTGCAGTTGGCTCTCATTTCTAAATTGTTCTTTTCGAAAAGTTTTTATGGCATCTAAAAACCGGCTATTTCCTCTCGTATATTTATGATATTTCTGTGCTTTATTATACTTTTTTTTTGCTTTTTTTTGGCAGGCCTTAAACCCTCTCAGTTATTTGGGAAACACAAATGGAAGATAGAGAAATTCTCAGAAATCAAGAAAAGGGAGCTCCGTAGCAATTATTTTGAAGCTGGCGGCTACAAATGGTACGTAGCATATTTTAATCTTTCTCATTCTTAAATTTTGTCTCACAATAATAAGTTGGGATCTGGTTTTAAGGCTCTGTTTTGTTACCTTTATCTTCAACTTTTCAGGTACATTTTAATTTATCCACAAGGATGTGATGTTTGCAATCATCTCTCCTTGTTTCTCTGTGTTGCAAACCATGATAAACTTCTTCCAGGTTAGTAGTTATGTCCACTGGTCTGCACGGTCCCCTTTCTCTCTTTGGATACCTTGTCATGTAGGTTCTTATGCTGTCTTTTTGGAACAGGCTGGAGTCATTTCGCTCAGTTTACCATAGCTGTGGTGAATAAAGATCCAAAGAAATCCAAATTTTCAGGTGACTTTTTGAGTCTTGATTTGCCTGCCCCCGTTTAAATATGCAGACAACTGAACTCACATCTTGCAGATACGCTTCACCGGTTTTGGAAGAAGGAGCATGATTGGGGATGGAAAAAGTTTATGGAGTCAACTAAGTTACAGGATGGGTTCATAGATGATTCCGACTCTCTTACAATTGAAGCTCAAGTTCAGGTGATCAGGTAAAACAAAGTTGTCTGTGTGTTGTTAGAGCCTGAGTAAAAGATTACCACGACCAATTTTCCATGCTACCATTCACTTACAGCTCCACAAGTTCATATTTTCACTTATTTATGTATTGTGGATAATATTCCTGTAACTGGTCCTTGCAGGGAAAGGGTGGACCGACCTTTTCGCTGCCTTCACTGCGGTTATAGGAGAGAGCTTGTTAGGGTGTATTTGTCAAACGTAGAGCAAAGTTGCCGACGTTTTGTGGAAGAGAAAAGAAGCAAGCTTGGGAGGTTGATAGAGGACAAGGCAAAATGGACGAGGTATTTCACATTGCAACTTATTCCCGTAGCTCTCTAGATTCCGAAAATGTAATCATAGTGCAGTTGCAATTTTCCTTGCTATGTTTAGAATTTTCTTGTTTTCCACCATGCGTTTGGAGTCCAACATAAATTTGCCTTAGTTTTAGAAATAATTTTTTTTATTTTCGTGGTTGTGGCTCTCCACTGTCTCTGTTTTACAAGTGATTGTAGCCAAGGAACTTGAGATGGTGGGACGGATAATCCCTTAAAGAGGATGTTAGAAAATTTTCTTAGTTCACACACTTGTTGAAGGCCAGGATTTACACCAATCTAGTAGAGTATTGTTGCCTTACGTTATATTTCTTATGTTATCTTTTTCTTTCAAGTTGAGCAATTTTGCCTAAAAAGGAATCGATTATCATTCTTGGTTGCAGCTTCGGTGTTTTCTGGTTAGGGATGGACCAAAACTCTAGGCATCGGATGTCTAGAGAGAAAATGGATGTAATCCTAAAAGGAATTGTAAAACACTTTTTCATAGAGAAGGAAGTTACATCCACTTTGGTGATGGATTCCTTGTATAGTGGGTTGAAGGCTCTTGAAGGCCAATCTAAGAGCAAGAAAGCTAGGCCAAGATCGTTAGATGCCAAGGAATGCCCAGCTCCGATTGTTAGTGTGGACAAAGATATGTTCGTATTAGTTGATGATGTGCTATTACTCTTAGAGAGAGCTGCTCTAGAACCGTTGCCTCCAAAAGAAAATAAAGCCCCCCAAAACCGTACAAAGGTCGGTTTCATGGGTTGTATATATATATTTTTGGTAGAAATTTCGTTATGCGTGATTGTATCTGCATTCCTTTCATATTTATTTTCATATTTGTTTTTGTCGTCTGTCCATTTTTCTCTTCTTGCATTGTGCACTAGAAAGTTACATGTATTGAGATAAATGTTTTTGTTCTTCAATGCAAATCTAGGATGGCAATGATGGAGAAGAGGTCAGCAATGAAGCCGTTGAGCGTGATGAGAGACATCTAACTGAGTTGGGCAGACGAGCTGTGGAGATATTTGTTCTTACCCATATATTCAAGTAACAATCCATAACTATGTTTCTATACGGTGTTCTATTGTGTTCTTGCTTTATGTTCTTATGTATCGTACTTCAATATCTCTGACAACTGCAGCAGCAAAATCGAGGTTGCATATAAAGAAGCCATTGCGCTAAAAAGGCAGGAAGACTTAATTCGTGAGGAAGAGGAAGAGTGGTTAGCAGAAACCGAACAGAGAGCCAAAAGAGGAGCAGCAGAGAGAGAGAAGAAATCTAAGAAGAAACAGGTGTGATATTATCTGCTGTTCCAAAAACTATTTTGCTCCTTCAGCAATGTTCTATACAGCTTGGTGGATGTTGATGAAAGCTTTCTTTGGTTGTTGGACATTTATAGGCAAAGCAGAAACGGAACAAAAATAAAGGGAAGGACAAAAAGAAGGAAGAAAAGGTGACATTAGCAACACATGGAAAGGATCTCGAGGAAAACCACCATGATGAGGAGGAAAATGATTCTGTTACAGAGAAAGCACAACCCTCAGCTGAAAAGACTGATACTCTTGAAGAAGTGTCCGACATATCTGACTCCGTGGATGGTTCGGCTGACATTCTTCACCCTGATTTAGAAGATGGGGACAGTAGTTCTGTTCATTGGGATGCCGATGCTTTGGAAATTCATCCTCCTCCATCAGAAGGGAGCAGCATTTCCATATCTACACCCAATGGAATTGCGGAAAGAAAGACTCAGTCTACTATGGATGATAGTTCCTCGACTTGTTCTAACGATTCTATCCGGTCAGGTGTTACCAACGGCTCTTACAAAGGGAATATGTTGAATTTCCGAAACCAGAAGTCGCCAAACCAGTAAGTTTGTTTTGACCTTCCCTGGCTTCTTCTGTCTGTTTTGGTTTCTAATGATAAGACACCTTTGTGATTTGCAGAGGTAAGAACCAGCAAGTAAAAATAACATCTGATACCCGCAGTTTAGTGACTGAACCAGATGATGATCAACCAAAGAGTCAGAATTCTTCTTCCGAATCTGACTGGGTTGTTGTCTCCCATATCCAGGAATTAGAGAGCTCTCGGAATCGTAGACCCGTGGAGAAGGTTGGTGGTCACTCATACTTTTAGTGTTCAGAACTTATAGAAGTGAGATTCTCACTGATTGAACTAATGCAAATTCCTTTTTCAGCAACGCAACGTGGCTCAAGTCGTTGTGAACTCGGTTCACATGGATCGACCTGAAAAGAAAAGCGCTGCTGTGCTTTCTTCTCCAAGAACTGCTGCTAAGAATCCTTCATCATTAACTCAGACAAAACTGGAGAAAAGGAGCGTTTCAAACGCAGACGCTGTTCCAAACAAGAAAGTAATGTCAGCTACTGGACCACCTTCATCAAGTCAGGTGTCGCCGGCATCTTCAGATAGTCAGTCGCAAGCTGGTGGTCTCAAAGCTGATATGCAAAAGATCTCTGCTCCAAAACAACCTGCAACAACAACAATTGTAACAAGGCCTTTTAGTGCTCCAATAATCCCTGCGATGCGACCCGCCCCTGTTATCGTCTCCTCCTCGGTTCAACCAACAACATCCCTTCCTCGGTCTGTTAGCTCAGCTGGTCGTCTAGGTCCTGACTCTTCACTACGCAACCAACAATCTTACACTCCTCAATCCTATAAACACGCCATAGTTGGTAACTCTCCTGGTTCGTCATCTAGTTTCAACCACCACCCAAGCTCTCATGGAGTTGTCCCAACCACATTGCCGTCAGCATCTTACACACAAACACCGGCTTATCAGTCGTCAAGCTTCCCTTTCGGTCAGGATGGGTCCTTTCGATCAAGGAGTTTTAATTCTGTAAACATGGGAATGAACAACCGTTACACACCGGCCGTGGCTAGCAACACTTCTCTGAACCACATTGATATCGAGACAGCGCGACAACAAGCACAAAGCTTGATGACCGACGAGTTCCCTCACCTCGACATCATCAACGACCTGCTGGAAGACGAAAACTGCAGCAACACGGTGTTTAATGGAAGCATATTCAATTCACAATCCCAGCTGTTCAACAGTCAATACTCTTACCATGGTGGTGGCAGTGCTGATTTAGGCATCTCAGGTGAGTTATTGTCTAGTGGCAGGTCCAGGAGTTTTGGAGATGAAGGATTCCACTACATGGCACGTGGACCATATGCAGAAGGTTTGATACCGACGCAGTGGCAGATGGCAAACATGGACCTGTCTTTACTTGCTATGAGGAATAGTAACGTGGAAGACACAGCATCATACCATCACACATACAACTTTGGATTGGACTCTACGAATCAGAGTTTTTCTTCAGGGATCAATGGCTACACTGAGTTCAGGCCGTCCAATGGTCACTGA

>BrHKL4(Bra030564)-CDS

ATGCTAAAGACATTGTTAGTTCACGGCCCTTTGCTCTTCTTCTTCTTCTTCTTGATCAGCTCCGTCGTCGCCGGCGATGAAAACGACGGCGGATTGTCGATATGCAACTGCGACGACGAAGACAGTTACTTCAGCTACGAAGGAATCCTCGAATCTCAAAAAGTCGGCGATTTTCTAATCGCCGTCGCCTACTTCTCCATCCCCATCGAGCTTCTCTACTTCGTGAGCCGCACCAACGTCTCTTCCCCTTACATCTGGGTCGTCTGCGAGTTCATAGCCTTCATCGTCCTCTGCGGCATGTCCCACTTGCTCTCCGGCTTCACCTACGGGCCCCACTACCCTTGGGTCATGACGGCCGCCACCGTCTTCAAAATGCTGACTGCGATCGTCTCTTTCCTCACAGCCATCTCGCTTGTCACTCTCTTACCGTTACTTCTTAAAGCTAAGGTTCGTGAGTTTATGTTGAGTAAGAAGACGAGAGAACTTAACCGTGAGGTTGGGCTTATAATGAAGCAGACGGAGACGAGTTTGCACGTGCGTATGCTCACTACTAAGATAAGGACGTCTTTGGATAGGCACACGATACTCTACACGACGCTTGTGGAGCTGTCGAAGACTCTGGGGCTCAAGAACTGCGCGGTTTGGATCCCTAATGAGATCAAGACGGAGATGAATCTCACGCACGAGTTGAACGGTGAGAATGTGGGTCGTGGGCCTGGTGGTGGGCCTAGTGGTGGGCCTGGTGGGTTTTCGATACCGATCACTGAGTCTGATGTTGTGAGGATTAAGAGAAGTGTGGAGGTGAATATGCTGAGTGCTGGCTCTGCTCTTGCCTCGGTTACTACCCGAGGCAAGTCAGGCCAGACGGTCGGGATCAGAGTCCCGATGCTTCGTGTTTGCAACTTCAAAGGTGGGACCCCTGAGGCTATCCACATGTGCTACGCCATCTTGGTCTGTGTGCTTCCTCTGAGGCGGAGCTGGAGTTACCAGGAGCTGGAGATCGTCAAGGTCGTGGCTGATCAAGTCGCGGTTGCGATCTCTCACGCGGTGATCCTCGAGGAGTCTCAGCTCATGAGGGAGAAGCTCGCGGAGCAGAACCGTGCGCTTCAGGTGGCGAGGGAGAACGCGATGAGAGCTAACCAAGCCAAAGCTGCGTTCGAGGAGATGATGGGGGATGCGATGAGGCGTCCCGTGAGGTCTATCCTCGAGCTGCTTCCTTTGATAACGCAGGACGGGGTGTCGTTACCCGAGACGCAGAAGGTTATCGTTGATGCTATGGGGAGAACCAGCGAGCTGCTGTTACACCTTGTGAACAATGCTGGGGATGTAGCTAGTGGGACCCATTGTTTTAGTTTGCGTTCGGTTGTGAAGGAAACGGCATGCTTGGCGAGGTGTTTGTGTTTGGGGAACGGGTTTGGTTTCACGACGGATGTTGATAGAGCGTTGCCTGATTGCGTCGTGGGCGATGCTAGGAAGGTGTTACAAGTGGTGTTGCATATGCTAGGAGGTGTAATGAACCGGAAGGTCAAAGGGAATGTGACGTTTAAGGTTGTACCGGAACGAGGAAGCTCAGAAGTTGTGAAAGAGAGCCAAGAAGCGGCTTGGCGACAATGCTATTCCAAAGAATACGTTGAAGTTAAGTTTGGATTTGATGTAGCTGCAGAAGGCGAAGAGAGTAGTAGTAGTAGCAGCAGCACTAAGTTCATGCAAGGGAACGTTTTGGTTGTGGAGGATGGTCTAGGTTTGGTGAAAAGCCTCTCTGTTGTGTTCCGGTTCCAGCTCCGGCGATCTATAGTGTCACGAGGCGGTGGATATTCGGGAGAAACTTTTAAAACATCGACTCCTCCGTCTACTAGTAACGGTCATTGGCGTCATTTGAAGGAAGCTTTGAAGAAAAATCTGTACTGGGGAATGACAGAGACTGTTAATGAAGATTCTGGAGTGGGGCGATCAGTAGAGGCGAGTTCTAATGGACACCACAGTCTATCAGGAGAATCACTCTCACTCTCTAAGTGGCGGTCTTCTGCTCAGGTTGAGAATGGGACGCCTTCCACATCCCTCTCTTACTGGGACACCGATGATGATGAAGACCACGGCCTTAAACCCTCTCAGTTATTTGGGAAACACAAATGGAAGATAGAGAAATTCTCAGAAATCAAGAAAAGGGAGCTCCGTAGCAATTATTTTGAAGCTGGCGGCTACAAATGGTACATTTTAATTTATCCACAAGGATGTGATGTTTGCAATCATCTCTCCTTGTTTCTCTGTGTTGCAAACCATGATAAACTTCTTCCAGGCTGGAGTCATTTCGCTCAGTTTACCATAGCTGTGGTGAATAAAGATCCAAAGAAATCCAAATTTTCAGATACGCTTCACCGGTTTTGGAAGAAGGAGCATGATTGGGGATGGAAAAAGTTTATGGAGTCAACTAAGTTACAGGATGGGTTCATAGATGATTCCGACTCTCTTACAATTGAAGCTCAAGTTCAGGTGATCAGGGAAAGGGTGGACCGACCTTTTCGCTGCCTTCACTGCGGTTATAGGAGAGAGCTTGTTAGGGTGTATTTGTCAAACGTAGAGCAAAGTTGCCGACGTTTTGTGGAAGAGAAAAGAAGCAAGCTTGGGAGGTTGATAGAGGACAAGGCAAAATGGACGAGCTTCGGTGTTTTCTGGTTAGGGATGGACCAAAACTCTAGGCATCGGATGTCTAGAGAGAAAATGGATGTAATCCTAAAAGGAATTGTAAAACACTTTTTCATAGAGAAGGAAGTTACATCCACTTTGGTGATGGATTCCTTGTATAGTGGGTTGAAGGCTCTTGAAGGCCAATCTAAGAGCAAGAAAGCTAGGCCAAGATCGTTAGATGCCAAGGAATGCCCAGCTCCGATTGTTAGTGTGGACAAAGATATGTTCGTATTAGTTGATGATGTGCTATTACTCTTAGAGAGAGCTGCTCTAGAACCGTTGCCTCCAAAAGAAAATAAAGCCCCCCAAAACCGTACAAAGAAAGTTACATGTATTGAGATAAATGTTTTTGTTCTTCAATGCAAATCTAGGATGGCAATGATGGAGAAGAGCAGCAAAATCGAGGTTGCATATAAAGAAGCCATTGCGCTAAAAAGGCAGGAAGACTTAATTCGTGAGGAAGAGGAAGAGTGGTTAGCAGAAACCGAACAGAGAGCCAAAAGAGGAGCAGCAGAGAGAGAGAAGAAATCTAAGAAGAAACAGGCAAAGCAGAAACGGAACAAAAATAAAGGGAAGGACAAAAAGAAGGAAGAAAAGGTGACATTAGCAACACATGGAAAGGATCTCGAGGAAAACCACCATGATGAGGAGGAAAATGATTCTGTTACAGAGAAAGCACAACCCTCAGCTGAAAAGACTGATACTCTTGAAGAAGTGTCCGACATATCTGACTCCGTGGATGGTTCGGCTGACATTCTTCACCCTGATTTAGAAGATGGGGACAGTAGTTCTGTTCATTGGGATGCCGATGCTTTGGAAATTCATCCTCCTCCATCAGAAGGGAGCAGCATTTCCATATCTACACCCAATGGAATTGCGGAAAGAAAGACTCAGTCTACTATGGATGATAGTTCCTCGACTTGTTCTAACGATTCTATCCGGTCAGGTGTTACCAACGGCTCTTACAAAGGGAATATGTTGAATTTCCGAAACCAGAAGTCGCCAAACCAAGGTAAGAACCAGCAAGTAAAAATAACATCTGATACCCGCAGTTTAGTGACTGAACCAGATGATGATCAACCAAAGAGTCAGAATTCTTCTTCCGAATCTGACTGGGTTGTTGTCTCCCATATCCAGGAATTAGAGAGCTCTCGGAATCGTAGACCCGTGGAGAAGCAACGCAACGTGGCTCAAGTCGTTGTGAACTCGGTTCACATGGATCGACCTGAAAAGAAAAGCGCTGCTGTGCTTTCTTCTCCAAGAACTGCTGCTAAGAATCCTTCATCATTAACTCAGACAAAACTGGAGAAAAGGAGCGTTTCAAACGCAGACGCTGTTCCAAACAAGAAAGTAATGTCAGCTACTGGACCACCTTCATCAAGTCAGGTGTCGCCGGCATCTTCAGATAGTCAGTCGCAAGCTGGTGGTCTCAAAGCTGATATGCAAAAGATCTCTGCTCCAAAACAACCTGCAACAACAACAATTGTAACAAGGCCTTTTAGTGCTCCAATAATCCCTGCGATGCGACCCGCCCCTGTTATCGTCTCCTCCTCGGTTCAACCAACAACATCCCTTCCTCGGTCTGTTAGCTCAGCTGGTCGTCTAGGTCCTGACTCTTCACTACGCAACCAACAATCTTACACTCCTCAATCCTATAAACACGCCATAGTTGGTAACTCTCCTGGTTCGTCATCTAGTTTCAACCACCACCCAAGCTCTCATGGAGTTGTCCCAACCACATTGCCGTCAGCATCTTACACACAAACACCGGCTTATCAGTCGTCAAGCTTCCCTTTCGGTCAGGATGGGTCCTTTCGATCAAGGAGTTTTAATTCTGTAAACATGGGAATGAACAACCGTTACACACCGGCCGTGGCTAGCAACACTTCTCTGAACCACATTGATATCGAGACAGCGCGACAACAAGCACAAAGCTTGATGACCGACGAGTTCCCTCACCTCGACATCATCAACGACCTGCTGGAAGACGAAAACTGCAGCAACACGGTGTTTAATGGAAGCATATTCAATTCACAATCCCAGCTGTTCAACAGTCAATACTCTTACCATGGTGGTGGCAGTGCTGATTTAGGCATCTCAGGTGAGTTATTGTCTAGTGGCAGGTCCAGGAGTTTTGGAGATGAAGGATTCCACTACATGGCACGTGGACCATATGCAGAAGGTTTGATACCGACGCAGTGGCAGATGGCAAACATGGACCTGTCTTTACTTGCTATGAGGAATAGTAACGTGGAAGACACAGCATCATACCATCACACATACAACTTTGGATTGGACTCTACGAATCAGAGTTTTTCTTCAGGGATCAATGGCTACACTGAGTTCAGGCCGTCCAATGGTCACTGA

>BrHKL5(Bra020013)-DNA

ATGGCTTGTCGTGAAGCGTTACCCGTTGGACGGGAAAGCTCCGAATTTCTAACGTGCGCTTGTTGCTTCTCGCGAGTGGTAAATTCCTGTCTCAATCTTTGAATTTCATGCTGCTTTATTGATGTTCTGGTTGGAGACTTTGGTAAATCTTATTATGAGTTTAGGGTTTCATCAAATTCGTGTATAGCGGTTACTGTAATGGGTGGAAGTCATTATTATAGCGGCTTGTACCTGGCGTGTCTTCGAAAACGAAAAGGAGCGTGCCTGGCGTTTCTGCTACTGGGCAAGTAATACCAAGTTTCCATTGGTTACAACTGGAGAAAGGGAGCATATCTGCAACGATGATTTTTTGACAAGAAAATGGGAGCCTGATTGGCGTGCCTGCAACTTGACAAGTGGTGCCGACTTTCCATTCCCCAGCCACAACATAAATACTGGGTAATCTATCTTCTTCTTTATTGCTTGCATATATATATATATATATGAATTTTGATCATTTATTATCTTGTGCTGAACTTATGCTATGTTAGTCTTTTCTTGTTATTCATGTTTCCCTTAATGCGTTAGTATTTTTTTTGTTGCATCCGGAATCACTCAGATTGAGTTTCTGATTCTTTTTTACACAGCACTTTAGAGGATCAGCATTTGTCAGCAGAAGATGGTTTAGGCACTTGGGAACCGCCAGAGGATCAGCATTCAAATGTAACAATCATCTGCCAGTACTGCCGAGCGGTAATTGTTATTTATCTCTCTCTTTTTTTCTTTTCCTTTTTGTGTGTGTGTGTTTCAATGGCTCATGAAGAAATCCAGATCCGTCTTAGTTTTCTAGTGATATTGGTTTATATGCAGACCGCAGCTGCCCTTGAGAAGGAATCTGATACCACACAGATGAGTCTCAGACTTTCAATGCTCAAACATTATCAGTGTCCATTCTTGCATGTGAATCAGAACACCAGCTCTCATCCTTATGTGTAAGCTATTCTGATCTGTATTGCTTTTAGTTGCTTTTTTTTTTCAAATGCCATTGTGTTCTGCTATTGTTCAGCTCATACACCACTGGCCGATATGAATTGTGATCTTGATTTTTTTGGGTTGATCTCATGCTATGTTTCCCTCTCACGCTGTCTTTGTCTTTTCTAGTTTTTGATTCTCATCTTCTTTGTTTTACACATTCACAGCACTCAAGATTCAGCCCAAGTCAGTGAGAACAACGAACCCTCTTCTTCTTCCTACCATCAGTGATTTAATGGTATTTATTATATTATTATTTAAGAAGTGATTTTGCTGATAAGTCATATTCTAAATGATTTTAGCTAATTTGATTATTTGTTATGTTATAAATAATTTTTATTAATGATTTTGCTTATTTGTTACTTTTAATGAATAGTGATATATAATTAACAACAAATTTTATATTTGATATTAACAACATAATAATATTGTATCAACTAACCACGTTAAAAAAGTCTAGCCGTTCATGAATGTTGCCAGGTTATATAACCAATTAATATCTTTGCTCACACGAGTTTAAAACTTCTCCATCGTCTAAATTTTTTTTATTTCTTGGTTATATGTAGGTTTTTTATTTTCCAACTTGCTTTTTCCTTCTTTGTTTATTAATATTTTGTGCAGATCCAAAAGTTCAGAATCAAATGATTAGGAAAGAACACAATTTAGAATGATTATTTATGTTTCATAAGCCATAGCACCAAAACAAGTCTTTATTTCTGTTCGTTTGGTACTTGAAGGACTCCTAAATCTACTGTGGTTGTTTGTAGTATTTGATATGAACCTTAATTTGTGATTTTATCATTTTCTATCTATTGCAGCCATAGTCGTATATGTCACTTTAATCTTGATCCTGGAGCATTGACAGAAAGCTTCCTTTGAGTAAAATAGCTTCTGTCTTGGCAAACGACACATGAGAATTTCATGTTTCCTTGCACAGTCTTCCCTCACCAGCTACCTTGTTAAGCTAACAGTGGACGCCATTGACTCCTCTGGAACAAAAGTTGTTCAATCAGTAGCTGAAGAAACTGAAACTCCTATGAGTTGTTATGATGTACCAGAAACACTATTGATGAATGATCCCTCACTGCTGTGTTGAATGTGACACAAGCAGTGGTTCATCTTCATCAAGTGTTCATGATGCATTCATTCCAGAAGAAAACACTTTCTGAGTTTGGTGGCAGGCTGGCAGCAATATATGTTTCTTTTGCGGTTGGAAGCCATGACACAGTAATGGTGTTCTTTATACCAATGCAGTTCAAACTAGAAAAAGATATTGTGGTTGGAAGAAGAGAAATGAGTTTTTTTCAATCCTACAAAGAATCGTTAAGATTCAGATTTGAATAATAATTTCCTTAAAGCAAACTGAAGGTGTTAAAATTAGTTTTATTTTTCTTCTGTTTCATGGCTGCATTTTACTGATTATCTTTTTAGATTCATGTAAGATGACATCAAGTCTGCAAACAATACTAACCTACTTTTTGGCATGGTGCTGACAAAAAAAAATAAAGTCCATTATTTGGTTGGTAATATTACTCAAGAAAATTACCTTGTGGCGAAGGCGTAAAAGTAGTGACCTACTCGCTTTCAAAGGTACAATCACCAAAACATCGAAACATTACTTATCCTTGATAGAAACATATAGTTGAAACTTGTGAGTTTCACTTTGTGAATAACATGCTTCTTTTAGCTCAACTATGAACAATAACAACAACAATACATAAGAAACTGCGAATTACAACGAGGAAGATCATCTAATCAAAACTGATACTCTTTTGGATGAAGACACTTTCATGTCTCTTGCATTTGAATGATGACCCATTGCATTGGTGAAAATCGTACATGGAAAAGTTCAATGGTACAATCGTCACATCAGAATGCAATTAACATTGATGTTATGATGAGGATGAGGTGTTGAGTTTGATGAGGATGCGAAGATTTGATTGGAAGAAGACTTATGAATTTGAGGAGGTACGATCTCTACAGAGATGTGTGATCGCTTCACTGAGTTGGATTTGGAAGATACAATTGGGTCTCTTATTAAATTCATAACCTCCATGGTTATGAGTTTGTCAAAAAAGAAAGTAGGAGATTTTCGATCGGATTCAATTGGTTACTATTAGATTAATTTGTGGACAAAATCTACAGATTTGGAGGGGGGAAATAGAAGTAACGGAGGATTCTATGTTTGACCTTGGAACGTCCAGGATGATCATCATCTCCACTAAAGGGGCCTTTACTCCCTATGTGTCCTTTGTAACACTGAGGCCTCTGGAAAACGTCGGATTGTGTAATAAAACACAAGCTCATGGTCTATACTCATGGAAGTTCCTTCGGGTTAAGAAATTTTTTACTTGTCTCAGTCTATTTTTTATAATTCTATTATATTATTTTATAATCAATTATCTAATGTAACATATAACATATAAATTTATACTATTAAAATAAAATTCATTTTTAATTATATATAAAATTCAGTAAATATATTGTTTAAAATTCATTTTTAATATAACATAGAAATTTAATATTATTAACATTCGTCTTTAATTATATACAAGATTCATTAAGGATAATTTTAAATTAATATATTAATGACTCAACTAATAACAAATAATAAATCAATGATTTAACTAAAACCTAATAAAAACATTACAAGTAAGCAAAATTACCTAAAATCATGGAGAACATGACAAATAAGAAAAATCACTTCATAAATAATAGTATAGATAGATAAACATGAATTAACTTAGCAAAAAAACATTAAAACACACAAAATCCAAATAACAACAAAATAAATAAAAATTAAATAAAAAATAAAGTTTGATGATAATTTTGAAGAATTGTCTTTTAAAAAAACGAATTGAGATAATGACAATATTTCATTGGTTTACTTAATCAATATATACATTCACTTATCTTAATATTTCATAAGTTTAGTTTTAATAAAAAAAACAATAGATAGTGATACTTTTATTATTAAAGTTAATTTATGGTTACTTATATAATGATAAATCTAATTATTCATTAAAAATTAATAAAGTAATAAGAAACCTAAAAAATAAAATTAAAGAGTTATAAAATTCAAAGGCGGATAGTCTAGCACGCAGTGCTCGAAATCAATCGTCTTTCGTCGTTCACATGGATGCAGATCACCCAGTTTGGTTCACAGAGTCAGTATGAGTCTTTTTTTTGATGACAAAAAAAAAAAAAAACATGATTACATATGCAATTCTCTAATTTAATATCAAACCATATTTTTCTTACATACACGAAGATTCTGGTTCCGCACCCTGTCCAGATAATCATAAAGTTAAAAATTTAAGATATAAAACAATTTATAATTAAATATTAGTCAAACAAAAAATATTAACTATATTTTACTTTTAATTTTTTCATGAGTTTCATAGAATTATAACTTTATTTATATTAAAAAAGTTTGAAAAGACTATTATGTAAAGAAAAATGTGATTTTGTTTTAATGAAAACTGTATTTTCCAAAATAATTTATTTTTGTTATAATTATCAAATTTTGAAAGTTAAAATATCATTTGAAAATAACAAATTATGATTTTTTTAATAAACAAATACTATTTTATAGGAAAATATTATCTCTATTTTATAATAAAAATATACAGATTGACTATAACTTATTTTTCTCTATTAACATTTAGAAATGAAAATGATAATAGATAGGTAGTGCCATTATCTTATACATTACATATTAATGTTTTGTTTGTCAACATGGTTAGTCTGTTTAAGTGTTTCTTCAATTATTATGTGAGGTATTTTAAAATATAATTATACATATATCAATATAGTTTATTTACTTTGATTAATCTAATAATTAAAGTTGGGTTTGGTCTAACATTCTTGGGCTTTTTTATTTATTTTGAGATTTTGTAAAAAAAATTGTTTAATTACATCTTTTTATATTAAATATTATATATTTGTAGATAATTGTTTATAAATTTATATAATAAAAACTAGTTAAAGATAACGACATATTTTAAAATATGAAATATTAGTGTTGTTATCCAAAAAGTTAAAAATTAAAAAATTGAAAAATTCATAAAATAGTATTTATTTAATATTTTATTTCAACTTTTTATCTTTAAATTTATAGTACATTTATTAATAAGTATCTACAAAGGAATTATGCCTGTATATGCGTATGTTTTACGAAGATTCTGGTTCTATCTTATTTTATAGCTATATTATTTAAATTAATAAATTATGGACTTAACTAAAAACTATTAATAAATTAATAACTCAGTTTAAATATAATTAAAACATGATAAATAAAAAAAATTATCTAAAATCATGAAAATTATGACAACGATAACTAAGCTAAATAATTTCATAAATAATAATATAGATTTCCCGTAATAGGCATCTAAAACGTGAAAGGTCGATAAAAAGGAATACAAATCTGGAAAGTCCCTTACTTTTAAATTTAATCGACCCAGCGAGGCAATAAACAAACCACAAAAAGCCGATAAAACGTGAAGCCCATAAGGACTTCCCTTGTCGTCTTCCTCAAACTCCACTTTCCACTTCCGGTTATTGCCTTCATTAAACACTGCCCCATCTCATCTCTCTCTCGCTTGTTTTTTTAATAATATCTTTTACTTTTCTTTTTTTAATCTTATCCTCTAGACGATTCTCTTTGATCAGATCATCTTCAATTTGTTCTCCCACTTGCACAGTGATAGTCACACACCCGTCAAAAATAAATATCAGTTTTGATGAAGTAAAAAATAGTTTTCTTTTTAAATCTCTTTTTTTTATTTGGGCTTAGGCCACCACCGTTGTGTGTTTGGAGAGAAAAAAAATATGTTACCGGAGAAGCCGACCTTGTTCTTGGCATGTGTGTGAAGTTTTGTCATTTATCCGAAATTAGCTTAACTTGTCCCGAATATAATATAAACAAATTGGGAGCCCATACTCCGATACATGGGCATACGTAAGGTTAGGTGGACCCATTCATCGTCGTCGTCTGTGCTAGGGAGCACAAATAATAGAGAGAAGAGAGAAGAAGCTGAGGCGGTGATCGAGGTCTTAAAGAAGATTTAGAGAAAGAAAGAATTAAAAAGCCTCTGAAGAGTGTGACTCGACAAGATCTGATCATTGTTTCTTTCAAACTCCTTTTAAAGGTAGCTACTTTCCTTTTCCCCAAGTTCCCTCCTTTTCTGTTGATTTTTTCATATAAATATTGAAGCTTTGCTTTGTTTGTTCTCTCCTCAGTGTGCTCAGCCAGCAACTGGAACTCACACTCCTCTAACTAAAGTAATTAGTTAAAAGCTATTCTCTGTTAGCATCTGTTTTCTATGGTTATAAACTTTGTAGGTCTCGTTTGATCTTCTGGCTTGAGGTGTGTTTGTTTTGTCTCAGTTTTTGTTGGCTGTGACCATTTATAAGAAGAAGCACCCCATCAATCGTTTCCTATTAGCACTCTTTTCCGAAAGGTATAGCTATGAAGTCTCTATGAGTTACCTCTTTAGTCTTTACTTATATTATGTATAGAAGTATTTGAAATGTTGGAAGGCAGATTGGCTCTGATCCTGGTTAGTTTTAGTTGGATTCTCCATGTTGAGTATCAACTACCGTTTGTGATACATGTTTTATTGTCCAGATCTTGTTGATTTTTGGATTGTTCACGGAATCTGTCTCCTTTACCTACTCTGTATACATCATAATCAATGCGGTTCAGTTGCTTGTTGCTTTGTAAATGATGAATCATTGAATAGTGGAACATGGCAGGATGATTCATGCATCTGTTTCTTGGTTAAGCTAGGTAGACTTTCTAGTTTTCTAGCCCATGCCTCTTTTTGATGTGAAAGGAACTTAAGCTATAGCGGTGTGACTTAGTGTTTCAGTTCTCGGTTAGTTCTTGAATGTTTTGCATTTAATTCTTGTTGTTATGTCTTTGGCATCAGGTGAAAACATGTCAGGTTCTAGGCCGAGCCACTCCTCCGAGGGCTCAAGGCGGTCAAGACACAGCGCTAGGATCATTGCGCAGACCACTGTTGACGCAAAGCTCCACGCTGACTTCGAGGAATCAGGCAGCTGCTTTGATTACTCAACCTCGGTCCGCGTCACCGGACCCGTTGTGGAGAACCAGCCACCAAGGTCTGACAAGGTGACCACCACCTATCTCCATCACATACAGAAGGGAAAACTGATTCAGCCCTTCGGCTGTTTGCTCGCCTTGGACGAAAAGACCTTCAAAGTCATTGCCTACAGCGAGAACGCCGCCGAGCTCTTGACGATGGCGAGTCATGCGGTTCCCAGCGTTGGTGAACAGGGTGCTCTAGGCATAGGAACGGACATAAGGAGCCTCTTCACTGCTCCCAGCGCCTCTGCGTTGCAGAAAGCGCTAGGATTCGGAGACGTCTCGCTCTTGAATCCCATTCTCGTGCATTGCAAGACTTCCGCAAAGCCCTTCTACGCGATCGTGCACAGGGTCACAGGGAGCATCATCGTGGACTTCGAACCTGTGAAGCCTTACGAAGTCCCCATGACGGCTGCTGGTGCTTTGCAATCGTACAAGCTCGCTGCAAAGGCCATCACTAGGCTGCAGTCTTTGCCCAGCGGGAGCATGGAGAGGCTCTGCGACACGATGGTTCAGGAGGTTTTCGAGCTCACGGGGTATGACAGAGTGATGGCTTATAAGTTTCACGACGATGATCACGGAGAGGTTGTCTCCGAGGTTACAAAGCCTGGTCTAGAGCCTTACCTTGGCCTCCATTATCCAGCCACAGACATCCCTCAAGCGGCTCGTTTTCTCTTTATGAAGAACAAGGTTCGGATGATAGTTGATTGCAACGCAAAACATGTGACGGTGCTTCAGGACGAAAAGCTTTCCTCTGATCTCACCTTGTGCGGCTCCACGCTCAGAGCGCCGCACAGCTGCCATCTGCAGTACATGGCCAACATGGATTCTATTGCATCTCTTGTTATGGCGGTTGTGGTAAACGAGGAGGACGGAGAAGGTGACGCCGCCGCACCTGATTCCACCGCCCCTCAGAAGAGGAAGAGGCTGTGGGGATTGGTGGTTTGTCACAACACCACTCCGAGGTTTGTTCCGTTTCCTCTCAGGTACGCCTGCGAGTTTCTAGCGCAAGTGTTTGCGATACACGTCAACAAGGAGGTGGAGCTGGAGAATCAGATCGTGGAGAAGAACATCTTGCGTACGCAGACGCTACTCTGCGATATGCTGATGCGTGATGCTCCCCTGGGAATCGTCTCGCAGAGCCCCAACATAATGGACCTTGTGAAATGCGACGGAGCAGCTCTCTTGTACAAAGACAAGGTATGGAAGCTGGGGATAACTCCGAGCGAGTTCCATCTCCAGGAGATAGCTTCGTGGCTGTGCGAGTACCACACTGACTCAACTGGTCTGAGCACTGATAGCTTGCACGACGCTGGGTTTCCAAGAGCTCTAGCTCTTGGAGACTCGGTCTGCGGAATGGCGGCTGTGAGGATCTCGTCAAAAGACATGATCTTCTGGTTCCGTTCTCACACCGCTGGGGAAGTGAGGTGGGGAGGTGCAAAGCATGATCCAGATGATAGGGATGACGCGAGGAGAATGCACCCGAGGTCTTCCTTCAAGGCTTTTCTTGAAGTGGTCAAGACGAGGAGTTTGCCTTGGAAGGACTATGAGATGGATGCCATACACTCCTTGCAGCTTATTCTGAGGAATGCTTTCAAGGATGGAGAAGCTACTGATGTGAATACGAAGATCATCCACTCGAAGCTGAATGATTTGAAAATTGATGGTATACAAGAGCTAGAAGCTGTCACTAGCGAGATGGTTCGTTTGATCGAGACTGCTACTGTGCCGATATTGGCGGTTGATTCTGATGGATTGGTTAATGGTTGGAACACGAAGATTTCTGAGCTGACTGGTCTTCCGGTTGATGAAGCAATCGGGAAGCATCTACTCACACTTGTTGAAGATTCTTCAGTGGAAATCGTTAAGAGGATGTTAGAGAATGCATTAGAAGGTAACTAACTCTCTTCCTAACTTACACTCAGTTTTGCTGAGAATATATTTCAGTTAGATGACACCTTTGGTTGATTGTTTATGCAGGAACTGAGGAGCAGAATGTCCAATTTGAGATCAAGACGCATCTGTCAAGAGCTGACGCTGGACCAATAAGTCTAGTGGTAAACGCATGCGCAAGTAGAGACCTCCACGAAAACGTGGTTGGTGTGTGCTTTGTAGCTCATGATCTTACAGGACAGAAGACTGTGATGGACAAGTTCACTCGCATCGAAGGTGATTACAAGGCCATCATCCAGAATCCAAACCCTCTGATCCCTCCCATATTTGGCACCGACGAGTTCGGGTGGTGCACGGAGTGGAATCCAGCGATGTCAAAGCTAACAGGTCTGAAGCGAGAGGAAGTGATGGACAAGATGCTGCTAGGAGAAGTCTTTGGGACGCAGAAGTCGTGTTGCCGTCTGAAGAATCAAGAAGCGTTTGTGAACCTTGGGATCGTGCTGAACAACGCTGTGACTAGCCAGGAGGCGGAGAAAGTGCCATTTGCTTTTTTTACGAGAGGTGGAAAGTATGTGGAGTGTCTGCTGTGTGTGAGTAAGAAGCTAGACAGGGAAGGTGTGGTGACAGGAGTCTTCTGTTTCCTGCAGCTTGCTAGCCATGAGCTGCAGCAAGCTCTTCATGTTCAGCGTCTAGCTGAGCGGACCGCGTTGAAGAGGTTAAAGGCCTTAGCTTACATTAAAAGACAGATCAGGAATCCGCTGTCCGGTATCATGTTCACAAGGAAAATGATGGAAGTGTCTGAGTTAGGACCAGAGCAGAGAAGGATTCTGCAAACTAGCGCTTTGTGTCAGAAGCAGCTCAGCAAGATCCTGGATGACTCTGATCTCGAAAGCATCATTGAAGGGTATGTATGCAAACAATGAAATGTCTTTGGTTGGTTCTCTCTTTTATTTGTTTTTTTTGCCTTATGTTGCAGGTGTTTGGATCTTGAGATGAAAGAGTTCAGCTTAAACGAAGTGTTGACTGCTTCCACAAGTCAAGTGATGATGAAGAGTAACGGAAAGAGCGTTAGGATAACAAATGATACAGGAGAAGAAGTGATGTCTGATACTTTGTATGGAGACAGTATCAGGCTTCAGCAAGTCTTGGCGGATTTCATGCTGATGTCTGTTAACTTTACACCATCCGGAGGAGAGCTGACTGTTACAGCTTCACTGAGGAAAGATCAGCTTGGAAGATCTGTGCATCTTGCTTATTTGGAGATCAGGTATACTTTTATTAATCAGGCGACATGTTAATGGAAAATTGCATTGATGGTTGTGTTGTTTATGAAAACAGAATAACGCATACAGGAGCTGGGTTACCAGAGTTTTTGCTAAACCAAATGTTTGGAACTGAGGAAGATATGTCGGAGGAAGGACTAAGCTTGATGGTTAGCCGGAAACTTGTGAAGCTGATGAATGGAGATGTTCAGTACTTGAGACAAGCTGGGAAATCGAGTTTTATCATCACTGCAGAACTCGCTGCAGCAAACAAGTAG

>BrHKL5(Bra020013)-CDS

ATGGCTTGTCGTGAAGCGTTACCCGTTGGACGGGAAAGCTCCGAATTTCTAACCACTTTAGAGGATCAGCATTTGTCAGCAGAAGATGGTTTAGGCACTTGGGAACCGCCAGAGGATCAGCATTCAAATGTAACAATCATCTGCCAGTACTGCCGAGCGTTTTTGTTGGCTGTGACCATTTATAAGAAGAAGCACCCCATCAATCGTTTCCTATTAGCACTCTTTTCCGAAAGGCAGATTGGCTCTGATCCTGGTGAAAACATGTCAGGTTCTAGGCCGAGCCACTCCTCCGAGGGCTCAAGGCGGTCAAGACACAGCGCTAGGATCATTGCGCAGACCACTGTTGACGCAAAGCTCCACGCTGACTTCGAGGAATCAGGCAGCTGCTTTGATTACTCAACCTCGGTCCGCGTCACCGGACCCGTTGTGGAGAACCAGCCACCAAGGTCTGACAAGGTGACCACCACCTATCTCCATCACATACAGAAGGGAAAACTGATTCAGCCCTTCGGCTGTTTGCTCGCCTTGGACGAAAAGACCTTCAAAGTCATTGCCTACAGCGAGAACGCCGCCGAGCTCTTGACGATGGCGAGTCATGCGGTTCCCAGCGTTGGTGAACAGGGTGCTCTAGGCATAGGAACGGACATAAGGAGCCTCTTCACTGCTCCCAGCGCCTCTGCGTTGCAGAAAGCGCTAGGATTCGGAGACGTCTCGCTCTTGAATCCCATTCTCGTGCATTGCAAGACTTCCGCAAAGCCCTTCTACGCGATCGTGCACAGGGTCACAGGGAGCATCATCGTGGACTTCGAACCTGTGAAGCCTTACGAAGTCCCCATGACGGCTGCTGGTGCTTTGCAATCGTACAAGCTCGCTGCAAAGGCCATCACTAGGCTGCAGTCTTTGCCCAGCGGGAGCATGGAGAGGCTCTGCGACACGATGGTTCAGGAGGTTTTCGAGCTCACGGGGTATGACAGAGTGATGGCTTATAAGTTTCACGACGATGATCACGGAGAGGTTGTCTCCGAGGTTACAAAGCCTGGTCTAGAGCCTTACCTTGGCCTCCATTATCCAGCCACAGACATCCCTCAAGCGGCTCGTTTTCTCTTTATGAAGAACAAGGTTCGGATGATAGTTGATTGCAACGCAAAACATGTGACGGTGCTTCAGGACGAAAAGCTTTCCTCTGATCTCACCTTGTGCGGCTCCACGCTCAGAGCGCCGCACAGCTGCCATCTGCAGTACATGGCCAACATGGATTCTATTGCATCTCTTGTTATGGCGGTTGTGGTAAACGAGGAGGACGGAGAAGGTGACGCCGCCGCACCTGATTCCACCGCCCCTCAGAAGAGGAAGAGGCTGTGGGGATTGGTGGTTTGTCACAACACCACTCCGAGGTTTGTTCCGTTTCCTCTCAGGTACGCCTGCGAGTTTCTAGCGCAAGTGTTTGCGATACACGTCAACAAGGAGGTGGAGCTGGAGAATCAGATCGTGGAGAAGAACATCTTGCGTACGCAGACGCTACTCTGCGATATGCTGATGCGTGATGCTCCCCTGGGAATCGTCTCGCAGAGCCCCAACATAATGGACCTTGTGAAATGCGACGGAGCAGCTCTCTTGTACAAAGACAAGGTATGGAAGCTGGGGATAACTCCGAGCGAGTTCCATCTCCAGGAGATAGCTTCGTGGCTGTGCGAGTACCACACTGACTCAACTGGTCTGAGCACTGATAGCTTGCACGACGCTGGGTTTCCAAGAGCTCTAGCTCTTGGAGACTCGGTCTGCGGAATGGCGGCTGTGAGGATCTCGTCAAAAGACATGATCTTCTGGTTCCGTTCTCACACCGCTGGGGAAGTGAGGTGGGGAGGTGCAAAGCATGATCCAGATGATAGGGATGACGCGAGGAGAATGCACCCGAGGTCTTCCTTCAAGGCTTTTCTTGAAGTGGTCAAGACGAGGAGTTTGCCTTGGAAGGACTATGAGATGGATGCCATACACTCCTTGCAGCTTATTCTGAGGAATGCTTTCAAGGATGGAGAAGCTACTGATGTGAATACGAAGATCATCCACTCGAAGCTGAATGATTTGAAAATTGATGGTATACAAGAGCTAGAAGCTGTCACTAGCGAGATGGTTCGTTTGATCGAGACTGCTACTGTGCCGATATTGGCGGTTGATTCTGATGGATTGGTTAATGGTTGGAACACGAAGATTTCTGAGCTGACTGGTCTTCCGGTTGATGAAGCAATCGGGAAGCATCTACTCACACTTGTTGAAGATTCTTCAGTGGAAATCGTTAAGAGGATGTTAGAGAATGCATTAGAAGGAACTGAGGAGCAGAATGTCCAATTTGAGATCAAGACGCATCTGTCAAGAGCTGACGCTGGACCAATAAGTCTAGTGGTAAACGCATGCGCAAGTAGAGACCTCCACGAAAACGTGGTTGGTGTGTGCTTTGTAGCTCATGATCTTACAGGACAGAAGACTGTGATGGACAAGTTCACTCGCATCGAAGGTGATTACAAGGCCATCATCCAGAATCCAAACCCTCTGATCCCTCCCATATTTGGCACCGACGAGTTCGGGTGGTGCACGGAGTGGAATCCAGCGATGTCAAAGCTAACAGGTCTGAAGCGAGAGGAAGTGATGGACAAGATGCTGCTAGGAGAAGTCTTTGGGACGCAGAAGTCGTGTTGCCGTCTGAAGAATCAAGAAGCGTTTGTGAACCTTGGGATCGTGCTGAACAACGCTGTGACTAGCCAGGAGGCGGAGAAAGTGCCATTTGCTTTTTTTACGAGAGGTGGAAAGTATGTGGAGTGTCTGCTGTGTGTGAGTAAGAAGCTAGACAGGGAAGGTGTGGTGACAGGAGTCTTCTGTTTCCTGCAGCTTGCTAGCCATGAGCTGCAGCAAGCTCTTCATGTTCAGCGTCTAGCTGAGCGGACCGCGTTGAAGAGGTTAAAGGCCTTAGCTTACATTAAAAGACAGATCAGGAATCCGCTGTCCGGTATCATGTTCACAAGGAAAATGATGGAAGTGTCTGAGTTAGGACCAGAGCAGAGAAGGATTCTGCAAACTAGCGCTTTGTGTCAGAAGCAGCTCAGCAAGATCCTGGATGACTCTGATCTCGAAAGCATCATTGAAGGGTGTTTGGATCTTGAGATGAAAGAGTTCAGCTTAAACGAAGTGTTGACTGCTTCCACAAGTCAAGTGATGATGAAGAGTAACGGAAAGAGCGTTAGGATAACAAATGATACAGGAGAAGAAGTGATGTCTGATACTTTGTATGGAGACAGTATCAGGCTTCAGCAAGTCTTGGCGGATTTCATGCTGATGTCTGTTAACTTTACACCATCCGGAGGAGAGCTGACTGTTACAGCTTCACTGAGGAAAGATCAGCTTGGAAGATCTGTGCATCTTGCTTATTTGGAGATCAGAATAACGCATACAGGAGCTGGGTTACCAGAGTTTTTGCTAAACCAAATGTTTGGAACTGAGGAAGATATGTCGGAGGAAGGACTAAGCTTGATGGTTAGCCGGAAACTTGTGAAGCTGATGAATGGAGATGTTCAGTACTTGAGACAAGCTGGGAAATCGAGTTTTATCATCACTGCAGAACTCGCTGCAGCAAACAAGTAG

>BrHKL6(Bra031672)-DNA

ATGTCAGGCTCTAGGCCAAGTCAGTCCTCTGAGGGCTCGTCAAGGCGATCAAGACACAGCGCTAGGATCATTGCACAGACCACCGTCGATGCAAAGCTCCACGCTGACTTCGAGGAGTCAGGAGGCAGCTCCTTTGATTACTCAACCTCAGTGCGTGTAACTACCCCAGCTGTGGAGAACAACCAGCCGCCGAGGTCCGACAAGGTGACCACGACATATCTCCACCACATACAAAAGGGAAAGCTAATCCAGCCCTTCGGCTGTTTACTCGCCTTGGATGAGAAGACCTTCAAAGTCATCGCGTACAGCGAAAACGCCCCTGAGCTCTTGACAATGGCTAGTCACGCGGTCCCCAGCGTCGGCGAGAGCCCCGTTCTAGGCGTTGGGACGGATATAAGGAGTCTCTTCACTGCTCCTAGTGCCTCTTCGTTGCAGAAGGCACTTGGATTCGGAGACGTCTCTCTTCTGAATCCCATTCTAGTCCACTGCAGGACTTCCGCAAAGCCGTTTTACGCGATTGTGCATAGGGTTACAGGGAGCATTGTAGTCGACTTCGAACCAGTGAAGCCTTATGAAGTTCCCATGACGGCTGCTGGCGCTTTACAATCTTACAAGCTCGCTGCAAAAGCGATCACTAGGCTTCAGTCTTTGCCTAGTGGGAGCATGGAGAGGCTTTGCGATACGATGGTTCAAGAGGTTTTCGAGCTCACGGGCTATGACAGAGTGATGGCTTATAAGTTTCATGAGGATGATCACGGCGAAGTTGTCTCCGAGGTGACGAAGCCTGTTCTTGAGCCTTATCTCGGTCTGCATTATCCGGCCACCGACATCCCTCAAGCGGCTCGGTTTCTCTTTATGAAGAATAAAGTTCGTATGATCGTCGATTGCAACGCAAAACATGTGAAGGTGCTTCAAGACGAGAAGCTTTCCTTTGATCTCACCTTGTGCGGCTCTACCCTTAGAGCGCCGCACACCTGCCATTTGCAGTACATGGCCAACATGGATTCTATTGCATCTCTTGTTATGGCGGTTGTAGTTAACGAGGAAGAAGATGGAGAAGCTACTACGCCTCAAAAGAGGAAGAGACTATGGGGTTTGGTGGTTTGTCACAACACAACTCCTAGGTTTGTCCCCTTTCCTCTCAGGTATGCATGTGAGTTTCTAGCTCAAGTGTTTGCGATACACGTGAACAAAGAGGTGGAGCTGGAGAACCAGATCGTGGAGAAGAACATTCTGCGCACGCAGACTCTCTTGTGCGATATGCTGATGCGCGATGCTCCTCTGGGGATTGTCTCGCAGAGTCCCAATATAATGGACCTTGTGAAATGCGATGGAGCGGCTCTTTTGTATAAAGACAAGGTATGGAATCTTGGAACAACTCCAAGCGAGTTTCATCTACAAGAGATAGCTTTTTGGTTGTGTGAACACCACGCTGATTCAACCGGTCTAAGCACTGATAGCTTGCATGACGCTGGGTTCCCAGGAGCTTTAGCCCTTGGGGATTCAGTATGCGGAATGGCGGCTGTGAGGATATCATCTAGAGACATGATTTTCTGGTTCCGTTCTCATACAGCTGGCGAAGTGAGATGGGGAGGTGCGAAGCATGATCCAGATGATAGAGATGACGCGAGGAGAATGCACCCGAGGTCATCGTTTAAGGCTTTTCTTGAAGTGGTTAAGACGAGGAGTTTACCTTGGAAGGACTATGAGATGGATGCTATACATTCATTGCAGCTTATTCTGAGGAATGCTTTCAAGGATGGAGAAAGTAGTGATGTGAATACTAATATTATTCACTCGAAGCTAAACGATTTAAAGATTGATGGTATACAAGAACTTGAAGCTGTGACTAGTGAGATGGTTCGTTTGATTGAGACTGCTACTGTGCCAATATTGGCGGTTGATTCTGATGGATTGGTTAATGGTTGGAACACGAAAATTGCTGAGCTGACTGGTCTTCCTGTTGATGAAGCGATTGGGAAGGATTTCCTCACACTTGTTGAAGATTCTTCGGTGGAAATCGTTCAGAGGATGTTAGAGAATGCACTAGAAGGTAATTTATCTTCCTAATATACGCTTAGTTTTGCTAAGAATATTTCAATTAGGCCTGGCATTTTGAACCAAAGCAAAATTTTGGTTAGTTCGGTTATAGGTTATAGATTGGTTAGAAGTTCGGTTCGATTCAGTAGATTTTCTTTAAAAAATTAGTTTTCGGTTCGGCTCACTCTGGCAGTCAGTAACTTACTTACTTCGGTTTTCTTTTTTTTATAAAAAAGTATACACAAACCATACTAAAAACCGAACCGAGCTAACCAAATTTCGAGCCGACCTAACCAAAATAATCAACAAAACGAATTTATCCGAGATTTTAACAAAATTAAACCAAAATATGACCGAAATCAAAAACTTCAGTAGGATTTTCAAAAACAGAATTAACAGAATACCGAACCGAACTTTATTCCGGGTTAATTCGTAAGATTAATGTTGAACCAAACTAACCGAAAACCGAACCTGCTGGCCAAATTTCAACTTCTTGACAAAGTGCTCTCAAAGTGCTAAAGAGTATAAACGTGGTTGTTGTATATATATGCAGGAACTGAGGAGCAGAATGTTCAGTTTGAGATCAAGACACATCTGTCAAGAACCGACGCTGGACCAATAAGTTTAGTTGTAAACGCCTGCGCAAGCAAAGACCTCCATGAAAACGTGGTTGGTGTATGTTTCGTAGCCCATGATCTCACCGCACAGAAGACTGTGATGGACAAGTTCACTCGTATCGAAGGCGACTACAAGGCCATCATCCAGAACCCAAACCCTCTGATCCCGCCCATATTTGGAACCGACGAGTCCGGGTGGTGCACGGAGTGGAATCCAGCGATGTCAAATCTAACCGGCTTGAAGCGTGAGGAGGTGGTGGAGAAGATGCTGTTGGGAGAAGTCTTCGGGACGCAGAAGAAGTCGTGCTGTCGTCTCAAGAATCAAGAAGCGTTTGTGAACCTTGGGATTGTGCTTAACAATGCTGTGACCAGTGAGGACGCTGAGAAAGTGCCGTTTGGTTTTTTCACGAGAGGTGGGAAGTATGTGGAGTGTTTGTTGTGTGTGAGTAAGAAGCTTGACAGGGAAGGTGCGGTGACTGGCGTCTTCTGTTTCTTGCAACTTGCTAGTTATGAGCTGCAGCAAGCTCTCCACGTGCAGCGTCTAGCTGAGCGTACTGCGCTGAAGAGACTAAAGGCTTTGGCTTATATAAAAAGAGAGATTCGGAATCCGTTGTCTGGGATCATGTTTACTAGGAAGATGATGGAGGGGACTGAGATAGGACCAGAGCAGAGAATGATCTTGCAAACTAGCGGTTTGTGTGAGAAGCAACTCAGCAAGATTCTTGATGATTCTGATCTTGAAAGCATCATTGAAGGATGCTTGGATCTGGAGATGAAAGAGTTCACCTTAAACGAAGTGTTGACTGCTTCCACAAGTCAAGTAATGATGAAGAGTAACGGAAAGAGCGTTCGTGTAACTAATGAAACAAAGGAAGAAGTAATGTCTGATACTTTGTATGGAGATAGTATTAGGCTTCAACAAGTTCTTGCAGATATCATGCTGATGTCTGTTAACTTCACACCATCTGGAGGTCAGTTAACTGTCACAGCTTCCTTGAGGAAAGATCAGCTTGGGAGATCAGTGCATCTTGCTTATTTAGAGATCAGGTAAATGTTTTAATGAATTACATAATATGATGTTAAAAAGAATGTGTGCATTGAGGAGATTTGTGTTGATGAAAACAGGATAACGCATACCGGAGCTGGTTTACCAGAGTTTTTGCTAAACCAAATGTTTGGGAGTGAGGAAGATGTTTCGGAGGAAGGGTTAAGCTTGATGGTTAGCAGGAAACTGGTGAAGCTGATGAATGGAGATGTTCAGTACTTGAGAGAAGCTGGGAAGTCTAGTTTCATTATCACTGCAGAACTCGCTGCAGCAAGCAAGTAG

>BrHKL6(Bra031672)-CDS

ATGTCAGGCTCTAGGCCAAGTCAGTCCTCTGAGGGCTCGTCAAGGCGATCAAGACACAGCGCTAGGATCATTGCACAGACCACCGTCGATGCAAAGCTCCACGCTGACTTCGAGGAGTCAGGAGGCAGCTCCTTTGATTACTCAACCTCAGTGCGTGTAACTACCCCAGCTGTGGAGAACAACCAGCCGCCGAGGTCCGACAAGGTGACCACGACATATCTCCACCACATACAAAAGGGAAAGCTAATCCAGCCCTTCGGCTGTTTACTCGCCTTGGATGAGAAGACCTTCAAAGTCATCGCGTACAGCGAAAACGCCCCTGAGCTCTTGACAATGGCTAGTCACGCGGTCCCCAGCGTCGGCGAGAGCCCCGTTCTAGGCGTTGGGACGGATATAAGGAGTCTCTTCACTGCTCCTAGTGCCTCTTCGTTGCAGAAGGCACTTGGATTCGGAGACGTCTCTCTTCTGAATCCCATTCTAGTCCACTGCAGGACTTCCGCAAAGCCGTTTTACGCGATTGTGCATAGGGTTACAGGGAGCATTGTAGTCGACTTCGAACCAGTGAAGCCTTATGAAGTTCCCATGACGGCTGCTGGCGCTTTACAATCTTACAAGCTCGCTGCAAAAGCGATCACTAGGCTTCAGTCTTTGCCTAGTGGGAGCATGGAGAGGCTTTGCGATACGATGGTTCAAGAGGTTTTCGAGCTCACGGGCTATGACAGAGTGATGGCTTATAAGTTTCATGAGGATGATCACGGCGAAGTTGTCTCCGAGGTGACGAAGCCTGTTCTTGAGCCTTATCTCGGTCTGCATTATCCGGCCACCGACATCCCTCAAGCGGCTCGGTTTCTCTTTATGAAGAATAAAGTTCGTATGATCGTCGATTGCAACGCAAAACATGTGAAGGTGCTTCAAGACGAGAAGCTTTCCTTTGATCTCACCTTGTGCGGCTCTACCCTTAGAGCGCCGCACACCTGCCATTTGCAGTACATGGCCAACATGGATTCTATTGCATCTCTTGTTATGGCGGTTGTAGTTAACGAGGAAGAAGATGGAGAAGCTACTACGCCTCAAAAGAGGAAGAGACTATGGGGTTTGGTGGTTTGTCACAACACAACTCCTAGGTTTGTCCCCTTTCCTCTCAGGTATGCATGTGAGTTTCTAGCTCAAGTGTTTGCGATACACGTGAACAAAGAGGTGGAGCTGGAGAACCAGATCGTGGAGAAGAACATTCTGCGCACGCAGACTCTCTTGTGCGATATGCTGATGCGCGATGCTCCTCTGGGGATTGTCTCGCAGAGTCCCAATATAATGGACCTTGTGAAATGCGATGGAGCGGCTCTTTTGTATAAAGACAAGGTATGGAATCTTGGAACAACTCCAAGCGAGTTTCATCTACAAGAGATAGCTTTTTGGTTGTGTGAACACCACGCTGATTCAACCGGTCTAAGCACTGATAGCTTGCATGACGCTGGGTTCCCAGGAGCTTTAGCCCTTGGGGATTCAGTATGCGGAATGGCGGCTGTGAGGATATCATCTAGAGACATGATTTTCTGGTTCCGTTCTCATACAGCTGGCGAAGTGAGATGGGGAGGTGCGAAGCATGATCCAGATGATAGAGATGACGCGAGGAGAATGCACCCGAGGTCATCGTTTAAGGCTTTTCTTGAAGTGGTTAAGACGAGGAGTTTACCTTGGAAGGACTATGAGATGGATGCTATACATTCATTGCAGCTTATTCTGAGGAATGCTTTCAAGGATGGAGAAAGTAGTGATGTGAATACTAATATTATTCACTCGAAGCTAAACGATTTAAAGATTGATGGTATACAAGAACTTGAAGCTGTGACTAGTGAGATGGTTCGTTTGATTGAGACTGCTACTGTGCCAATATTGGCGGTTGATTCTGATGGATTGGTTAATGGTTGGAACACGAAAATTGCTGAGCTGACTGGTCTTCCTGTTGATGAAGCGATTGGGAAGGATTTCCTCACACTTGTTGAAGATTCTTCGGTGGAAATCGTTCAGAGGATGTTAGAGAATGCACTAGAAGGAACTGAGGAGCAGAATGTTCAGTTTGAGATCAAGACACATCTGTCAAGAACCGACGCTGGACCAATAAGTTTAGTTGTAAACGCCTGCGCAAGCAAAGACCTCCATGAAAACGTGGTTGGTGTATGTTTCGTAGCCCATGATCTCACCGCACAGAAGACTGTGATGGACAAGTTCACTCGTATCGAAGGCGACTACAAGGCCATCATCCAGAACCCAAACCCTCTGATCCCGCCCATATTTGGAACCGACGAGTCCGGGTGGTGCACGGAGTGGAATCCAGCGATGTCAAATCTAACCGGCTTGAAGCGTGAGGAGGTGGTGGAGAAGATGCTGTTGGGAGAAGTCTTCGGGACGCAGAAGAAGTCGTGCTGTCGTCTCAAGAATCAAGAAGCGTTTGTGAACCTTGGGATTGTGCTTAACAATGCTGTGACCAGTGAGGACGCTGAGAAAGTGCCGTTTGGTTTTTTCACGAGAGGTGGGAAGTATGTGGAGTGTTTGTTGTGTGTGAGTAAGAAGCTTGACAGGGAAGGTGCGGTGACTGGCGTCTTCTGTTTCTTGCAACTTGCTAGTTATGAGCTGCAGCAAGCTCTCCACGTGCAGCGTCTAGCTGAGCGTACTGCGCTGAAGAGACTAAAGGCTTTGGCTTATATAAAAAGAGAGATTCGGAATCCGTTGTCTGGGATCATGTTTACTAGGAAGATGATGGAGGGGACTGAGATAGGACCAGAGCAGAGAATGATCTTGCAAACTAGCGGTTTGTGTGAGAAGCAACTCAGCAAGATTCTTGATGATTCTGATCTTGAAAGCATCATTGAAGGATGCTTGGATCTGGAGATGAAAGAGTTCACCTTAAACGAAGTGTTGACTGCTTCCACAAGTCAAGTAATGATGAAGAGTAACGGAAAGAGCGTTCGTGTAACTAATGAAACAAAGGAAGAAGTAATGTCTGATACTTTGTATGGAGATAGTATTAGGCTTCAACAAGTTCTTGCAGATATCATGCTGATGTCTGTTAACTTCACACCATCTGGAGGTCAGTTAACTGTCACAGCTTCCTTGAGGAAAGATCAGCTTGGGAGATCAGTGCATCTTGCTTATTTAGAGATCAGGATAACGCATACCGGAGCTGGTTTACCAGAGTTTTTGCTAAACCAAATGTTTGGGAGTGAGGAAGATGTTTCGGAGGAAGGGTTAAGCTTGATGGTTAGCAGGAAACTGGTGAAGCTGATGAATGGAGATGTTCAGTACTTGAGAGAAGCTGGGAAGTCTAGTTTCATTATCACTGCAGAACTCGCTGCAGCAAGCAAGTAG

>BrHKL7(Bra022192)-DNA

ATGGTTTCCGGAGTCGGCGGCGGCGGAGGAAGCGGCGGGAGTGGCCGCGGCGGCGGCCGCGGCGGAGAGGAATCCTCGTCAACTCACCGGAGAGAACAAGCTCAATCCTCAGGTACAAAATCTCTCAGGCCACAGAGCCAACCACAAACGGAATCAATCAGCAAAGCTATTCAGCAATACACAGTCGACGCTCGACTCCACGCCGTCTTCGAACAATCCGGCGAGTCAGGAAGATCCTTCGACTACTCGCAATCCCTCAAAACGACGACGTACGGCTCCTCGGTCCCGGAGCAGCAGATCACGGCCTACCTCTCCAGGATCCAGCGCGGCGGCTACATCCAGCCGTTCGGATGCATGATCGCCGTCGACGAGTCCACCTTCGCCATCATCGGCTACAGCGAGAACGCGCGGGAAATGCTCGGCCTCACGCCCCAGTCCGTCCCCAGCCTCGAGAGGCCCGAGATTCTCGCCATGGGGACCGACGTCCGCTCCCTCTTCACCTCCTCCAGCTCGGTCCTCCTCGAACGCGCCTTCGTGGCGCGTGAGATCACGCTCCTGAATCCCGTTTGGATCCACTCCAAGTACACTGGTAAACCGTTTTACGCTATCTTACACAGGATCGACGTTGGAGTCGTGATCGATTTGGAGCCGGCGAGGACTGAGGATCCAGCGCTTTCCATCGCCGGAGCGGTTCAGTCTCAGAAGCTAGCCGTTCGCGCTATCTCGCAGTTACAGTCTCTCCCCGGTGGAGATATTAAGCTGCTTTGTGATACGGTTGTGGAGAGTGTGAGGGACTTAACCGGATACGACCGTGTGATGGTGTACAAGTTTCACGAAGACGAGCATGGAGAGGTTGTTGCGGAGAGTAGGAGAGAGGATTTGGAGCCTTACATTGGTTTGCATTATCCAGCTACTGATATTCCTCAAGCGTCACGGTTCTTGTTTAAGCAGAATCGAGTGCGTATGATTGTTGATTGCCACGCGACGCCGGTTCTCGTGGTGCAAGACGATAGGCTGACTCAGTCTATGTGCTTGGTTGGGTCTACTTTGAGAGCTCCTCACGGTTGTCACTCTCAGTATATGGCTAATATGGGGTCTATCGCGTCTTTAGCGATGGCGGTTATAATAAACGGGAGTGAAGAAGATGGGAGTAGTGTCGCTGGTGGGAGAAGCGCTATGAGGCTTTGGGGTTTAGTCGTTTGCCATCATACTTCTTCTCGATGCATACCGTTTCCGTTGAGGTATGCTTGCGAGTTTTTGATGCAGGCGTTTGGTTTACAGTTGAACATGGAGTTGCAGTTGGCTTTGCAGATGTCGGAGAAACGCGTTTTGAGGACGCAGACGCTGTTGTGCGATATGCTTCTGCGTGACTCGCCTGCTGGGATTGTTACGCAGAGTCCTAGTATCATGGACTTGGTGAAATGTGACGGTGCAGCGTTTCTTTACCACGGGAATTATTACCCGTTGGGTGTTGCTCCGACGGAAGCTCAGATTAAAGACGTTGTGGAGTGGTTGCTTGCGAATCACGCTGATTCAACTGGTTTAAGCACTGATAGTTTAGGGGACGCAGGTTATCCCGGAGCAGCTGCGTTAGGGGATGCTGTGTGCGGTATGGCGGTTGCGTATATCACAAAAAGAGATTTCCTTTTCTGGTTTCGTTCTCATACAGCTAAAGAGATCAAATGGGGAGGCGCTAAGCATCATCCAGAGGACAAAGATGATGGGCAAAGGATGCATCCTCGCTCTTCTTTCAAGGCTTTTCTTGAAGTTGTTAAGAGTCGGAGTCAGCCGTGGGAAACTGCGGAAATGGATGCGATTCATTCGCTCCAGCTGATTCTAAGGGACTCTTTTAAGGAATCTGAGGCAGCTATGAACTCGAAAACTGCAGATGGCGCGGTTCAGCCTTATAGTATGGCAGGAGAGCAGGGGATTGATGAGTTAGGTGCTGTTGCAAGAGAGATGGTTAGACTCATCGAGACAGCAACGGTGCCTATATTCGCTGTGGATGCTGGAGGATGCATCAATGGATGGAACGCTAAGATTGCAGAGTTGACCGGTCTCTCGGTTGAAGAAGCTATGGGGAAGTCTCTGGTTTCTGATCTGATATACAAAGAGAATGAAGAAACTGTTGATAAGCTCATTTCTCGTGCGCTCAGAGGTACATTCAGTTCTCCAACTATGTTGTATTTGCTGTATGGTTATTTGATTATTTTGATGCGTTTTGCAGGGGACGAGGACAAGAATGTGGAGATAAAGCTGAAAACTTTCAGCCCTGAACTACAAGGGAAAGCGGTTTTTGTGGTTGTGAATGCATGTTCGAGCAAGGACTACTCAAACAACATTGTTGGCGTCTGTTTTGTGGGACAGGATGTTACTGGTCAGAAAATCGTAATGGACAAGTTCATCAACATACAAGGAGACTACAAAGCCATTGTCCATAGCCCAAACCCACTGATTCCACCAATCTTTGCGGCGGACGAGAACACGTGCTGCCTGGAGTGGAACACCGCATTGGAAAAGCTCACTGGTTGGTCTCGCAGTGAAGTGATTGGGAAAATGCTTGTTGGGGAAGTGTTTGGGAGCTGTTGCAGGCTAAAGGGTCCTGATGCATTAACCAAGTTCATGATTGTGTTGCATAATGCGATCGGTGGCCAAGAAACAGACAAGTTCCCTTTCCCATTCTTTGACCGCAACGGGAAGTTTGTTCAGGCTCTACTGACTGCAAACAAGCGTGTAAGCTTCGATGGAAAGGTTATTGGGGCTTTCTGTTTCTTGCAGATCCCGAGTCCTGAGCTGCAGCAAGCTTTAGCAGTTCAACGAAGGCAGGACACAGAGTGTTTCACGAAGGCGAAAGAGCTAGCTTACATTTGTCAGGTGGTAAAGAATCCTTTGAGCGGTTTGCGTTTCACAAACTCGTTGTTGGAAGCTACAGACTTGAACGAGGATCAGAAACAGCTTCTTGAAACAAGTGTTTCTTGCGAGAAACAGATTTCCAGGATTGTCAGTGACATGGATCTTGAAAGAATTGAAGACGGGTAAGCTTAGTTGGAATTTTCTATAAAATGGTTTTTGATTACTTGATAGAATGTGACCAGATTCCAAATATTGTTTTGCTCTATTTGATCAGTTCGTTTGAGCTAGTTAGAACGGAGTTTTTACTCGGAAGTGTCATCAACGCGATTGTAAGCCAGGCGATGTTCTTATTAAAGGAGAGAGGTGTTCAGCTGATCCGTGACATTCCTGAAGAGATCAAATCTATACAAGTTTATGGAGACCAGACAAGGATTCAACAGCTCCTAGCTGAGTTTCTGCTGAGTATAATCCGGTATGCACCGTCCCATGAGTGGGTGGAGATCCATATAAGCCATGTTCCGAAGCAAATGGCTGATGGATTCTACGCTATTCGCACAGAATTTAGGTACTTATTTCATTGTTTCAGCTATTGTATCCTCATATCTGTTTTGGTTTTTGTTTGTTTGGATATAGAATGGTTTGGTTTTTGGATCGTTATTTAGGTGGCAGTTATGGCTTGTTTAGACCAGATTGGTAACTTCTACAGGGAACCATATAGGACTAGAATTAGCAGATATTAATGTTTTCAGATTAGCATGTTTGGGGTTCTCTGTTATGTCTCAATTTTCTGCGATTTCAGAATGGCGTGCCCAGGTGAAGGTCTGCCTCCAGAGCTAGTCCGAGACATGTTTCATAGCAGCAGGTGGACGAGCCCTGAAGGGTTAGGACTAAGCGTATGTAGAAAGATTCTAAAGATCATGAACGGTGAGGTTCAGTACATCCGAGAGTCGGAACGTTCCTATTTCCTCATCATACTCGAGCTCCCGGTGCCTATGAAACGCCCTTTGTCAACCGCTAGTGGCAGCGGAGACATGATGTTGATGATGCCATAG

>BrHKL7(Bra022192)-CDS

ATGGTTTCCGGAGTCGGCGGCGGCGGAGGAAGCGGCGGGAGTGGCCGCGGCGGCGGCCGCGGCGGAGAGGAATCCTCGTCAACTCACCGGAGAGAACAAGCTCAATCCTCAGGTACAAAATCTCTCAGGCCACAGAGCCAACCACAAACGGAATCAATCAGCAAAGCTATTCAGCAATACACAGTCGACGCTCGACTCCACGCCGTCTTCGAACAATCCGGCGAGTCAGGAAGATCCTTCGACTACTCGCAATCCCTCAAAACGACGACGTACGGCTCCTCGGTCCCGGAGCAGCAGATCACGGCCTACCTCTCCAGGATCCAGCGCGGCGGCTACATCCAGCCGTTCGGATGCATGATCGCCGTCGACGAGTCCACCTTCGCCATCATCGGCTACAGCGAGAACGCGCGGGAAATGCTCGGCCTCACGCCCCAGTCCGTCCCCAGCCTCGAGAGGCCCGAGATTCTCGCCATGGGGACCGACGTCCGCTCCCTCTTCACCTCCTCCAGCTCGGTCCTCCTCGAACGCGCCTTCGTGGCGCGTGAGATCACGCTCCTGAATCCCGTTTGGATCCACTCCAAGTACACTGGTAAACCGTTTTACGCTATCTTACACAGGATCGACGTTGGAGTCGTGATCGATTTGGAGCCGGCGAGGACTGAGGATCCAGCGCTTTCCATCGCCGGAGCGGTTCAGTCTCAGAAGCTAGCCGTTCGCGCTATCTCGCAGTTACAGTCTCTCCCCGGTGGAGATATTAAGCTGCTTTGTGATACGGTTGTGGAGAGTGTGAGGGACTTAACCGGATACGACCGTGTGATGGTGTACAAGTTTCACGAAGACGAGCATGGAGAGGTTGTTGCGGAGAGTAGGAGAGAGGATTTGGAGCCTTACATTGGTTTGCATTATCCAGCTACTGATATTCCTCAAGCGTCACGGTTCTTGTTTAAGCAGAATCGAGTGCGTATGATTGTTGATTGCCACGCGACGCCGGTTCTCGTGGTGCAAGACGATAGGCTGACTCAGTCTATGTGCTTGGTTGGGTCTACTTTGAGAGCTCCTCACGGTTGTCACTCTCAGTATATGGCTAATATGGGGTCTATCGCGTCTTTAGCGATGGCGGTTATAATAAACGGGAGTGAAGAAGATGGGAGTAGTGTCGCTGGTGGGAGAAGCGCTATGAGGCTTTGGGGTTTAGTCGTTTGCCATCATACTTCTTCTCGATGCATACCGTTTCCGTTGAGGTATGCTTGCGAGTTTTTGATGCAGGCGTTTGGTTTACAGTTGAACATGGAGTTGCAGTTGGCTTTGCAGATGTCGGAGAAACGCGTTTTGAGGACGCAGACGCTGTTGTGCGATATGCTTCTGCGTGACTCGCCTGCTGGGATTGTTACGCAGAGTCCTAGTATCATGGACTTGGTGAAATGTGACGGTGCAGCGTTTCTTTACCACGGGAATTATTACCCGTTGGGTGTTGCTCCGACGGAAGCTCAGATTAAAGACGTTGTGGAGTGGTTGCTTGCGAATCACGCTGATTCAACTGGTTTAAGCACTGATAGTTTAGGGGACGCAGGTTATCCCGGAGCAGCTGCGTTAGGGGATGCTGTGTGCGGTATGGCGGTTGCGTATATCACAAAAAGAGATTTCCTTTTCTGGTTTCGTTCTCATACAGCTAAAGAGATCAAATGGGGAGGCGCTAAGCATCATCCAGAGGACAAAGATGATGGGCAAAGGATGCATCCTCGCTCTTCTTTCAAGGCTTTTCTTGAAGTTGTTAAGAGTCGGAGTCAGCCGTGGGAAACTGCGGAAATGGATGCGATTCATTCGCTCCAGCTGATTCTAAGGGACTCTTTTAAGGAATCTGAGGCAGCTATGAACTCGAAAACTGCAGATGGCGCGGTTCAGCCTTATAGTATGGCAGGAGAGCAGGGGATTGATGAGTTAGGTGCTGTTGCAAGAGAGATGGTTAGACTCATCGAGACAGCAACGGTGCCTATATTCGCTGTGGATGCTGGAGGATGCATCAATGGATGGAACGCTAAGATTGCAGAGTTGACCGGTCTCTCGGTTGAAGAAGCTATGGGGAAGTCTCTGGTTTCTGATCTGATATACAAAGAGAATGAAGAAACTGTTGATAAGCTCATTTCTCGTGCGCTCAGAGGTACATTCAGTTCTCCAACTATGTTGTATTTGCTGTATGGTTATTTGATTATTTTGATGCGTTTTGCAGGGGACGAGGACAAGAATGTGGAGATAAAGCTGAAAACTTTCAGCCCTGAACTACAAGGGAAAGCGGTTTTTGTGGTTGTGAATGCATGTTCGAGCAAGGACTACTCAAACAACATTGTTGGCGTCTGTTTTGTGGGACAGGATGTTACTGGTCAGAAAATCGTAATGGACAAGTTCATCAACATACAAGGAGACTACAAAGCCATTGTCCATAGCCCAAACCCACTGATTCCACCAATCTTTGCGGCGGACGAGAACACGTGCTGCCTGGAGTGGAACACCGCATTGGAAAAGCTCACTGGTTGGTCTCGCAGTGAAGTGATTGGGAAAATGCTTGTTGGGGAAGTGTTTGGGAGCTGTTGCAGGCTAAAGGGTCCTGATGCATTAACCAAGTTCATGATTGTGTTGCATAATGCGATCGGTGGCCAAGAAACAGACAAGTTCCCTTTCCCATTCTTTGACCGCAACGGGAAGTTTGTTCAGGCTCTACTGACTGCAAACAAGCGTGTAAGCTTCGATGGAAAGGTTATTGGGGCTTTCTGTTTCTTGCAGATCCCGAGTCCTGAGCTGCAGCAAGCTTTAGCAGTTCAACGAAGGCAGGACACAGAGTGTTTCACGAAGGCGAAAGAGCTAGCTTACATTTGTCAGGTGGTAAAGAATCCTTTGAGCGGTTTGCGTTTCACAAACTCGTTGTTGGAAGCTACAGACTTGAACGAGGATCAGAAACAGCTTCTTGAAACAAGTGTTTCTTGCGAGAAACAGATTTCCAGGATTGTCAGTGACATGGATCTTGAAAGAATTGAAGACGGTTCGTTTGAGCTAGTTAGAACGGAGTTTTTACTCGGAAGTGTCATCAACGCGATTGTAAGCCAGGCGATGTTCTTATTAAAGGAGAGAGGTGTTCAGCTGATCCGTGACATTCCTGAAGAGATCAAATCTATACAAGTTTATGGAGACCAGACAAGGATTCAACAGCTCCTAGCTGAGTTTCTGCTGAGTATAATCCGGTATGCACCGTCCCATGAGTGGGTGGAGATCCATATAAGCCATGTTCCGAAGCAAATGGCTGATGGATTCTACGCTATTCGCACAGAATTTAGAATGGCGTGCCCAGGTGAAGGTCTGCCTCCAGAGCTAGTCCGAGACATGTTTCATAGCAGCAGGTGGACGAGCCCTGAAGGGTTAGGACTAAGCGTATGTAGAAAGATTCTAAAGATCATGAACGGTGAGGTTCAGTACATCCGAGAGTCGGAACGTTCCTATTTCCTCATCATACTCGAGCTCCCGGTGCCTATGAAACGCCCTTTGTCAACCGCTAGTGGCAGCGGAGACATGATGTTGATGATGCCATAG

>BrHKL8(Bra039485)-DNA

ATGTCATCAGGTAGCAGCAATTCCGGAAGCTGTTCAACTCGATCCAGAAACAACTCTCGACTTTCTTCACAAGTCCTCGCTGATGCAAAGCTCCACGGGAGTTTCGAGGAATCCGAGCGCTTATTCGACTACTCAGCTTCAATTCACGTGAACATGCCCACCTCCTCGTCCTACGACATCCCTTCCTCTTCAGACGTCTCATCTTACTTACACAAGATTCAGAGAGGGATGTTGATTCAGCCCTTTGGTTGCTTAATCGTCGTTGACGACAAGACCCTCAAAGTAATTGCCTTTAGCGAGAACACGCAAGAGATGTTGGGTTTGTCTCCACACACTGTGCCTAGCATGGAGCAACGTGAGGCTCTGAGTATCGGAACTGATGTGCAGTCTCTGTTTCAGTCTCAAGGCTCTTCTGCGTTGCAGAAAGCTGCTGACTTTGGTGAGATTAGTATTCTGAATCCTATCACGCTTCACTGCAGGACTTCGGGTAAGCCTTTCTATGCCATTCTCCATCGGATTGAACAAGGTCTCGTTATAGATTTGGAGCCAGTGGGTCTTGATGAGGTCCCAGTGACTGCTGCTGGTGCGTTGAAGTCATACAAGCTCGCTGCTAAATCGATTTCGAGGTTGCAGGCTTTGCCTAGTGGGAATATGTCGTTGCTCTGTGATGCTTTAGTTAAGGAAGTTAGTGAGTTAACCGGGTATGATAGGGTGATGGTTTATAAGTTTCATGGAGATGGGCATGGGGAAGTGATTGCTGAGTGCTGTAAGGCAGACTTGGAACCTTATCTTGGGTTGCATTACTCGGCTACTGATATACCACAAGCCTCTAGATTTCTCTTTATGAGGAACAAGGTTAGGATGATTTGTGATTGTTCGGCTGTTCCAGTTAAAGTAGTCCAGGACAAGAGTCTCTCACAGCCGATTACTCTCGCTGGATCTACTCTGAGAGCTCCTCATGGCTGTCACGCGCAGTATATGAGTAATATGGGCTCAGTGGCATCTCTTGTTATGTCCGTAACGATCAATGGTAGTGAGAGTGATGAGATGAACAGAGATTTGCAGACGGGTAGAACCTTATGGGGCTTAGTGGTTTGTCATCACGCAAGTCCTAGGGTCGTCCCGTTTCCTCTGAGATATGCCTGTGAATTCTTGACTCAGGTGTTTGGCGTGCACATCAACAAGGAAGCGGAATCAGCTCTTCTGTTGAAAGAGAAGCATATTCTGCAAACTCAGAGTGTGCTATGTGACATGCTTTTTCGCAATGCACCTATAGGTATAGTCACTCAGTCTCCAAATATAATGGATCTTGTTAAATGTGATGGAGCAGCTCTTTATTACAGAGATAAGCTCTGGGCTTTAGGGGTTGCTCCTACAGAGACACAAATTAGAGATATAATCGATTGGGTTCTCAAAAGTCAAGGAGGAGGAAACAGTGGCGTTACCACTGAAAGTCTAATGGAGTCTGGCTATCCGGATGCTTCCGTCCTCGGGGAGTCAATCTGTGGAATGGCTGCCGTACATATAACCCAAAAGGTTTTCCTTTTCTGGTTCCGGTCTGGCACTGCAAAACAGATCAAGTGGGGTGGTGCAAGACATGATCCTGATGACAGAGATGGTAAAAGGATGCATCCTAGATCCTCGTTCAAGGCTTTTATGGAAATAGTCCGGTGGAAAAGTATGCCCTGGGATGACATGGAAATGGATGCAATCAATTCTCTGCAGCTGATAATAAAAGGCTCACTGCAAGAGGAGCATCCAGACACTGTTGTGAATGTGCCGCCGTTTGTGGATAATAGAGTCCAGAAGGTGGATGAAATGTGTGTTATTGTGAACGAAATGGTGCGGTTGATTGACACAGCTGCTGTTCCGATCTTTGCGGCTGATGCCTCTGGTGTTATAAACGGTTGGAATTCAAAAGCGGCTGAGGTGACTGGGTTGGCTGTTGAACAAGCGATAGGCAAACCTGTATCAGATATCGTTGAAGATGATTCTGCAATAACCGTTAAGAACATGTTAGCATTGGCTCTCCAAGGTCAGTATTTGGAAGCAAATGTCGTTTGTGTTGTTTCCGGTTCCCTTTTATATTTAGTTTTCATATTTTCCAGACTATTTTCATTCATAATACTAATACATAATAGGTAGCGAAGAACGTGGCGCCGAGATCAGGATCAGAGCATTTGGTCCTAAAAGGAAAAGCAGTCCTATTGAATTAGTCGTCAACACTTGCTGTAGCAGAGATACAAGGAATAATGTTCTTGGTGTGTGCTTCATTGGACAAGATGTTACAGGCCAGAAGACGCTTATTGAGAAGTATAGCCGCGTGCAAGGAGATTACGCCAGAATCATGTGGAGCCCTTCAACACTGATCCCACCAATTTTTATGACCACTGAGAATGGGTTATGCTCAGAGTGGAACGACGCGATGCAGAAGCTCTCTGGTATAAGGAGAGAAGAAGCTGTGAATAAAATGCTTCTTGGAGAGGTTTTCACCTCAAATGACTCATGCTGTCGCCTTCAAGACCATGACACGTTAACTAAACTCAGAATAGCTTTAAATGCTGTGAGTTCTGGCCAGGATAACATAGAGAAGCTTTTATTTGGCTTCTACCATCGTGATGGTAGATTCATCGAGGCCTTGCTTTCTGCAAACAAAAGAACTGACATGGAAGGAAAAGTTACAGGGGTTTTATGCTTTCTGCAAGTACCTAGTCCAGAACTCCAATACGCTCTACAGGTTCAGCGAATATCAGAGCAGGCAATGGCCTGCGCTGTCAACAAAATGGCATATCTCCGCCAACAAGTGGAGAATCCAGAAAAAGCAATATCCTTCCTTCAAGATTTTCTACATTCATCTGGATTAAATGAAGAACAAAAGCAGCTCTTGAGTACAAGCGTGTCATGCAGGGAGCAGTTAGCCAAAGTCATAAGCGACTCAGACATAGAGGGAATTGAGGATGGGTACTACTATAACCTGGATTCTTGTGTTTTTTTCCTGGTTGGATTGGTTACTATTTTTTGAATTTTCTGTTCTAATCCTGTTGCTTTTTGTTTGTTTGTTTGTTGATGTCTTTAAATCAGGTATGTGCAGCTGGGTTGCAGCGAATTCAGCCTTGAGGAATCCCTGGAATCAGTTGTAAAACAAGTCATGGAGCTGAGCATAGAACGTAAAGTGCAGATCATCTGCGATTATCCTCAAGAAGTTTCGTTAATGAGACTGTATGGAGATTCCTTAAGGCTTCAGCAAATCCTTTCAGAGACACTCTCAAGCAGCATACGGTTCACTCCTGCATTAAAGGGACTGTGCGTATCATTTAAGGTAATGTCACGGATAGAAGCTATAGGGAAAAGAATGAAGAGAGTAGAGCTAGAGTTCAGGATAATACACCCGGCACCGGGACTGCCTGACGATCTAGTAAGAGAGATGTTTCAGCCTTTGAGGAAAGATACATCAAGGGAAGGATTGGGACTACACATAACACAGAAGATGGTGAAACTCATGGAGGGAGGAACGTTGAGATACTTGAGAGAATCGGAAATGTCAGCTTTTGTAATCCTCGCAGAATTTCCATTGCTTTGA

>BrHKL8(Bra039485)-CDS

ATGTCATCAGGTAGCAGCAATTCCGGAAGCTGTTCAACTCGATCCAGAAACAACTCTCGACTTTCTTCACAAGTCCTCGCTGATGCAAAGCTCCACGGGAGTTTCGAGGAATCCGAGCGCTTATTCGACTACTCAGCTTCAATTCACGTGAACATGCCCACCTCCTCGTCCTACGACATCCCTTCCTCTTCAGACGTCTCATCTTACTTACACAAGATTCAGAGAGGGATGTTGATTCAGCCCTTTGGTTGCTTAATCGTCGTTGACGACAAGACCCTCAAAGTAATTGCCTTTAGCGAGAACACGCAAGAGATGTTGGGTTTGTCTCCACACACTGTGCCTAGCATGGAGCAACGTGAGGCTCTGAGTATCGGAACTGATGTGCAGTCTCTGTTTCAGTCTCAAGGCTCTTCTGCGTTGCAGAAAGCTGCTGACTTTGGTGAGATTAGTATTCTGAATCCTATCACGCTTCACTGCAGGACTTCGGGTAAGCCTTTCTATGCCATTCTCCATCGGATTGAACAAGGTCTCGTTATAGATTTGGAGCCAGTGGGTCTTGATGAGGTCCCAGTGACTGCTGCTGGTGCGTTGAAGTCATACAAGCTCGCTGCTAAATCGATTTCGAGGTTGCAGGCTTTGCCTAGTGGGAATATGTCGTTGCTCTGTGATGCTTTAGTTAAGGAAGTTAGTGAGTTAACCGGGTATGATAGGGTGATGGTTTATAAGTTTCATGGAGATGGGCATGGGGAAGTGATTGCTGAGTGCTGTAAGGCAGACTTGGAACCTTATCTTGGGTTGCATTACTCGGCTACTGATATACCACAAGCCTCTAGATTTCTCTTTATGAGGAACAAGGTTAGGATGATTTGTGATTGTTCGGCTGTTCCAGTTAAAGTAGTCCAGGACAAGAGTCTCTCACAGCCGATTACTCTCGCTGGATCTACTCTGAGAGCTCCTCATGGCTGTCACGCGCAGTATATGAGTAATATGGGCTCAGTGGCATCTCTTGTTATGTCCGTAACGATCAATGGTAGTGAGAGTGATGAGATGAACAGAGATTTGCAGACGGGTAGAACCTTATGGGGCTTAGTGGTTTGTCATCACGCAAGTCCTAGGGTCGTCCCGTTTCCTCTGAGATATGCCTGTGAATTCTTGACTCAGGTGTTTGGCGTGCACATCAACAAGGAAGCGGAATCAGCTCTTCTGTTGAAAGAGAAGCATATTCTGCAAACTCAGAGTGTGCTATGTGACATGCTTTTTCGCAATGCACCTATAGGTATAGTCACTCAGTCTCCAAATATAATGGATCTTGTTAAATGTGATGGAGCAGCTCTTTATTACAGAGATAAGCTCTGGGCTTTAGGGGTTGCTCCTACAGAGACACAAATTAGAGATATAATCGATTGGGTTCTCAAAAGTCAAGGAGGAGGAAACAGTGGCGTTACCACTGAAAGTCTAATGGAGTCTGGCTATCCGGATGCTTCCGTCCTCGGGGAGTCAATCTGTGGAATGGCTGCCGTACATATAACCCAAAAGGTTTTCCTTTTCTGGTTCCGGTCTGGCACTGCAAAACAGATCAAGTGGGGTGGTGCAAGACATGATCCTGATGACAGAGATGGTAAAAGGATGCATCCTAGATCCTCGTTCAAGGCTTTTATGGAAATAGTCCGGTGGAAAAGTATGCCCTGGGATGACATGGAAATGGATGCAATCAATTCTCTGCAGCTGATAATAAAAGGCTCACTGCAAGAGGAGCATCCAGACACTGTTGTGAATGTGCCGCCGTTTGTGGATAATAGAGTCCAGAAGGTGGATGAAATGTGTGTTATTGTGAACGAAATGGTGCGGTTGATTGACACAGCTGCTGTTCCGATCTTTGCGGCTGATGCCTCTGGTGTTATAAACGGTTGGAATTCAAAAGCGGCTGAGGTGACTGGGTTGGCTGTTGAACAAGCGATAGGCAAACCTGTATCAGATATCGTTGAAGATGATTCTGCAATAACCGTTAAGAACATGTTAGCATTGGCTCTCCAAGGTAGCGAAGAACGTGGCGCCGAGATCAGGATCAGAGCATTTGGTCCTAAAAGGAAAAGCAGTCCTATTGAATTAGTCGTCAACACTTGCTGTAGCAGAGATACAAGGAATAATGTTCTTGGTGTGTGCTTCATTGGACAAGATGTTACAGGCCAGAAGACGCTTATTGAGAAGTATAGCCGCGTGCAAGGAGATTACGCCAGAATCATGTGGAGCCCTTCAACACTGATCCCACCAATTTTTATGACCACTGAGAATGGGTTATGCTCAGAGTGGAACGACGCGATGCAGAAGCTCTCTGGTATAAGGAGAGAAGAAGCTGTGAATAAAATGCTTCTTGGAGAGGTTTTCACCTCAAATGACTCATGCTGTCGCCTTCAAGACCATGACACGTTAACTAAACTCAGAATAGCTTTAAATGCTGTGAGTTCTGGCCAGGATAACATAGAGAAGCTTTTATTTGGCTTCTACCATCGTGATGGTAGATTCATCGAGGCCTTGCTTTCTGCAAACAAAAGAACTGACATGGAAGGAAAAGTTACAGGGGTTTTATGCTTTCTGCAAGTACCTAGTCCAGAACTCCAATACGCTCTACAGGTTCAGCGAATATCAGAGCAGGCAATGGCCTGCGCTGTCAACAAAATGGCATATCTCCGCCAACAAGTGGAGAATCCAGAAAAAGCAATATCCTTCCTTCAAGATTTTCTACATTCATCTGGATTAAATGAAGAACAAAAGCAGCTCTTGAGTACAAGCGTGTCATGCAGGGAGCAGTTAGCCAAAGTCATAAGCGACTCAGACATAGAGGGAATTGAGGATGGGTATGTGCAGCTGGGTTGCAGCGAATTCAGCCTTGAGGAATCCCTGGAATCAGTTGTAAAACAAGTCATGGAGCTGAGCATAGAACGTAAAGTGCAGATCATCTGCGATTATCCTCAAGAAGTTTCGTTAATGAGACTGTATGGAGATTCCTTAAGGCTTCAGCAAATCCTTTCAGAGACACTCTCAAGCAGCATACGGTTCACTCCTGCATTAAAGGGACTGTGCGTATCATTTAAGGTAATGTCACGGATAGAAGCTATAGGGAAAAGAATGAAGAGAGTAGAGCTAGAGTTCAGGATAATACACCCGGCACCGGGACTGCCTGACGATCTAGTAAGAGAGATGTTTCAGCCTTTGAGGAAAGATACATCAAGGGAAGGATTGGGACTACACATAACACAGAAGATGGTGAAACTCATGGAGGGAGGAACGTTGAGATACTTGAGAGAATCGGAAATGTCAGCTTTTGTAATCCTCGCAGAATTTCCATTGCTTTGA

>BrHKL9(Bra013286)-DNA

ATGGGATTCGAGAGCTCTTCATCAGCCGCAAGCAACATGGAACAACCACAACAAAAGTCCAACACAGCTCAACAATACTCCGTCGACGCCGGCCTCTTCGCCGACTTCGACCACTCTGTCTACTCCGGCAAGTCCTTCAACTACTCCAAGTCCATGATCTCCCCTCCCAACAACGTCCCCGACGAGCACATCACCGCCTACCTCTCCACCATCCAAAGAGGCGGCCTCGTCCAGCCCTTCGGCTGCCTCATCGCCGTCCAAGAGCCTAGCTTCAGAATCCTCGGCCTCAGCGACAACTGCATCGACTTCCTCGGCCTCTCTCTCGCTTCCACCTCTCAACCAAACCACTTCACCGTCAAGGGTTTGATCGGAATCGACGCCAGGTCCCTCTTCACTCCTTCCTCAGCCGCTTCCTTGGTCAAAGCTGCTTCCTTTACTGAAATCTCCTTGATGAACCCTGTTTTAGTTCACTCCAGAACCACCACCACCGCCTCCCATAAGCCCTTTTACGCTATCCTTCACAGGATCGACGCCGGGATTGTGATTGACTTGGAGCCCGCCAAGTCATCCGACCCGGCGTTGACCCTCGCCGGGGCCGTTCAGTCTCAGAAATTGGCAGTCAGAGCCATCTCGAGGCTCCAGTCGCTTCCTGGAGGAGACATTGGTGCCTTGTGTGACACCGTTGTGGAAGATGTTCAGAGGCTTACCGGCTACGACCGTGTGATGGTCTACCAGTTTCACGATGATGATCATGGTGAGGTTGTTTCGGAGATTAGAAGGTCTGATCTCGAGCCTTACTTGGGTTTGCACTACCCTGCGACTGATATTCCACAAGCTGCACGGTTCTTGTTTAAACAGAACCGTGTAAGAATGATCTGTGATTGTAATGCCACTCCGGTTAAAGTTGTGCAGAGCGAAGAGCTCAAGAGATCGCTTTGTTTGGTGAACTCTACATTAAGAGCTCCTCATAGCTGTCATACTCAGTATATGGCGAATATGGGTTCTATTGCATCTCTTGTTCTTGCTATTGTGACTAAAACCAAAAACTCGAGTAAGCTTTGGGGGTTAGTGGTAGGCCACCACTGTTCTCCTAGATACGTGCCGTTTCCGTTACGTTACGCTTGCGAGTTTCTGATGCAAGCGTTTGGACTCCAGCTTCAGATGGAGCTTCAGTTAGCGTCCCAGCTGGCTGAGAAGAAGGCTATGAAGACGCAGACTTTGCTGTGCGATATGCTTCTCCGTGATACTGTCTCCGCTATCGTGACTCAGTCTCCTGGGATTATGGACCTTGTGAAGTGCGATGGAGCTGCTTTGTACTACAAGGGGAGGTGTTGGCTGGTCGGTGTGACTCCCAGCGAGTCGCAGGTTAAGGAACTGGTGGATTGGCTGGTGGAGAATCACGGTGATGAGTCTACAGGTTTGACTACTGATAGTTTGGTGGATGCTGGTTACCCTGGAGCTGTCTCTCTCGGAGATAAGGTCTGCGGCGTGGCTGCGGCGGGGATCTCTTTAAAAGACTACTTGATTTGGTTCAGGTCTAATACCGCTAGTGCAATCAAATGGGGAGGAGCTAAGCATCACCCCAAGGATAAGGATGATGCTGGAAGGATGCATCCGAGGTCGTCTTTTAAAGCGTTTCTTGAAGTTGCAAAGAGCAGGAGCTTACCGTGGGAAGTTTCTGAGATCGACGCGATTCATTCCCTGAGGGTGATAATGAGAGAGTCGTTTACTATCTCAAGGCCTGTTGTGTTGTCGTCTGGTGGTAATAACGGTGTGGTGGGGGGGAGAGATGCGAGTGAGCTTACTTCTTTTGTCTGTGAGATGGTTAGAGTGATTGAAACCGCGACTGCGCCTATCTTTGGTGTTGATTCTAATGGATGTGTCAATGGTTGGAATAATAAAACCGCTGAGATGACGGGGCTGGGAGCTGGTGAAGCTATGGGGAAGTCGCTTGTCGATGAGATTGTTCAGGAGGAGTCACGTGGAGCTCTTGAGAGTGTCTTGTCTAAAGCCTTACAAGGTGAAGAGAAGAAGAACGTAATGCTTAAACTGAGAAAGTTTGGTAACGAAGATTCATCTTCTTCTGATGTGTGTGTTCTCGTTAACTCATGCACGAGTAGAGACTATACGGAGAAAATAGTCGGTGTCTGCTTCGTTGGCCAAGACATGACTAGCGAGAAGGCAATAACAGATAGGTTCATCAGGCTGCAAGGAGACTACAAGACCATTGTCCAAAGCTTAAACCCTTTGATTCCACCTATATTCGCATCTGATGAGAACGCTCGTTGCTCAGAGTGGAACGCAGCAATGGAGAAGCTCACCGGGTGGTCGAAGCACGAGGTGATTGGGAGGATGCTACCTGGTGAAGTCTTTGGAGATTTGTGTAAAGTGAAGTGCCAAGATGCACTCACAAAGTTCTTGATCTCTCTGTACCAAGGCATTGCTGGTGGTGGTAATGTACCAGAGAGTTCGGTGGTTGGGTTCTTTAGTAAAGAAGGGAAGTACATAGAAGCATCATTAACGGCGAACAAGAGTACGAACGGTGAAGGGAAAGTCATAGGGTGTTTCTTCTTCTTGCAGATAATCAATAAAGAATCGAGTTCTAGCTCACCAGAGGTGAAAGAGAGCGCTCAGAGTCTCAACGAGTTGGCTTACATAAGGCAAGAGATCAAGAACCCTCTCAACGGTATCCGGTTTGCGCATAAGCTTCTTGAATCCTCGGAGATTTCAGAGAATCAAAGGCAGTTTCTGGAGACTAGTGATGCTTGTGAGAAGCAGATTGCGACAATAATCGAAGACACTGACTTGAAAAGCATTGAGGAAGGGTAAAGAAACATAACATCTTGAATCTTTTTCTTCATCTACTGTTGTTTTTTTTTTACATTTTAAATCTGAAAAAAATGAAATGGTTTTGTCAGCAAGTTGCAAATGGAGACAGAGGAGTTTCGACTTGAGAGCGTCTTGGACACAATCATTAGCCAAGTGATGATTATGTTGAGAGAGAGGAAGTCACAACTCAGAGTTGAAGTCTCCCAGGAGATCAAAACGCTGCCTCTCTATGGTGACAGAGTAAAGCTTCAGCTCATTCTTGCTGATCTTCTGCGCAACATTGTGAATCATGCCCCGTTTCCAGATAGTTGGGTAGGAATCAAGATCTCACCAGGCCACAAGCTTGCACACGACAATAATCCCTATATCCATCTACAGTTCAGGTATAAACGTCCACTGAAAAATGCAGCCAACCCATTTTCACAAAACAATCATTTTCTCAAACCGTTGATGTTGTTTTGATGGTAGGATGATACATCCTGGAAAGGGACTTCCTTCAGAGATGCTAAGTGATATGTTCGAGACTAGAGAAGGATGGGTCACGCCTGATGGGTTAGGACTAAAGCTTTCAAGGAAACTGCTGGAGCAGATGAATGGTCGTGTGAGTTATGTTAGAGAAGACGAACGTTGTTTCTTTCAAGTGGATCTTCAAGTTAAGACAAGGTTAGGTGTTGAGACTGGTTCAAGCATATAG

>BrHKL9(Bra013286)-CDS

ATGGGATTCGAGAGCTCTTCATCAGCCGCAAGCAACATGGAACAACCACAACAAAAGTCCAACACAGCTCAACAATACTCCGTCGACGCCGGCCTCTTCGCCGACTTCGACCACTCTGTCTACTCCGGCAAGTCCTTCAACTACTCCAAGTCCATGATCTCCCCTCCCAACAACGTCCCCGACGAGCACATCACCGCCTACCTCTCCACCATCCAAAGAGGCGGCCTCGTCCAGCCCTTCGGCTGCCTCATCGCCGTCCAAGAGCCTAGCTTCAGAATCCTCGGCCTCAGCGACAACTGCATCGACTTCCTCGGCCTCTCTCTCGCTTCCACCTCTCAACCAAACCACTTCACCGTCAAGGGTTTGATCGGAATCGACGCCAGGTCCCTCTTCACTCCTTCCTCAGCCGCTTCCTTGGTCAAAGCTGCTTCCTTTACTGAAATCTCCTTGATGAACCCTGTTTTAGTTCACTCCAGAACCACCACCACCGCCTCCCATAAGCCCTTTTACGCTATCCTTCACAGGATCGACGCCGGGATTGTGATTGACTTGGAGCCCGCCAAGTCATCCGACCCGGCGTTGACCCTCGCCGGGGCCGTTCAGTCTCAGAAATTGGCAGTCAGAGCCATCTCGAGGCTCCAGTCGCTTCCTGGAGGAGACATTGGTGCCTTGTGTGACACCGTTGTGGAAGATGTTCAGAGGCTTACCGGCTACGACCGTGTGATGGTCTACCAGTTTCACGATGATGATCATGGTGAGGTTGTTTCGGAGATTAGAAGGTCTGATCTCGAGCCTTACTTGGGTTTGCACTACCCTGCGACTGATATTCCACAAGCTGCACGGTTCTTGTTTAAACAGAACCGTGTAAGAATGATCTGTGATTGTAATGCCACTCCGGTTAAAGTTGTGCAGAGCGAAGAGCTCAAGAGATCGCTTTGTTTGGTGAACTCTACATTAAGAGCTCCTCATAGCTGTCATACTCAGTATATGGCGAATATGGGTTCTATTGCATCTCTTGTTCTTGCTATTGTGACTAAAACCAAAAACTCGAGTAAGCTTTGGGGGTTAGTGGTAGGCCACCACTGTTCTCCTAGATACGTGCCGTTTCCGTTACGTTACGCTTGCGAGTTTCTGATGCAAGCGTTTGGACTCCAGCTTCAGATGGAGCTTCAGTTAGCGTCCCAGCTGGCTGAGAAGAAGGCTATGAAGACGCAGACTTTGCTGTGCGATATGCTTCTCCGTGATACTGTCTCCGCTATCGTGACTCAGTCTCCTGGGATTATGGACCTTGTGAAGTGCGATGGAGCTGCTTTGTACTACAAGGGGAGGTGTTGGCTGGTCGGTGTGACTCCCAGCGAGTCGCAGGTTAAGGAACTGGTGGATTGGCTGGTGGAGAATCACGGTGATGAGTCTACAGGTTTGACTACTGATAGTTTGGTGGATGCTGGTTACCCTGGAGCTGTCTCTCTCGGAGATAAGGTCTGCGGCGTGGCTGCGGCGGGGATCTCTTTAAAAGACTACTTGATTTGGTTCAGGTCTAATACCGCTAGTGCAATCAAATGGGGAGGAGCTAAGCATCACCCCAAGGATAAGGATGATGCTGGAAGGATGCATCCGAGGTCGTCTTTTAAAGCGTTTCTTGAAGTTGCAAAGAGCAGGAGCTTACCGTGGGAAGTTTCTGAGATCGACGCGATTCATTCCCTGAGGGTGATAATGAGAGAGTCGTTTACTATCTCAAGGCCTGTTGTGTTGTCGTCTGGTGGTAATAACGGTGTGGTGGGGGGGAGAGATGCGAGTGAGCTTACTTCTTTTGTCTGTGAGATGGTTAGAGTGATTGAAACCGCGACTGCGCCTATCTTTGGTGTTGATTCTAATGGATGTGTCAATGGTTGGAATAATAAAACCGCTGAGATGACGGGGCTGGGAGCTGGTGAAGCTATGGGGAAGTCGCTTGTCGATGAGATTGTTCAGGAGGAGTCACGTGGAGCTCTTGAGAGTGTCTTGTCTAAAGCCTTACAAGGTGAAGAGAAGAAGAACGTAATGCTTAAACTGAGAAAGTTTGGTAACGAAGATTCATCTTCTTCTGATGTGTGTGTTCTCGTTAACTCATGCACGAGTAGAGACTATACGGAGAAAATAGTCGGTGTCTGCTTCGTTGGCCAAGACATGACTAGCGAGAAGGCAATAACAGATAGGTTCATCAGGCTGCAAGGAGACTACAAGACCATTGTCCAAAGCTTAAACCCTTTGATTCCACCTATATTCGCATCTGATGAGAACGCTCGTTGCTCAGAGTGGAACGCAGCAATGGAGAAGCTCACCGGGTGGTCGAAGCACGAGGTGATTGGGAGGATGCTACCTGGTGAAGTCTTTGGAGATTTGTGTAAAGTGAAGTGCCAAGATGCACTCACAAAGTTCTTGATCTCTCTGTACCAAGGCATTGCTGGTGGTGGTAATGTACCAGAGAGTTCGGTGGTTGGGTTCTTTAGTAAAGAAGGGAAGTACATAGAAGCATCATTAACGGCGAACAAGAGTACGAACGGTGAAGGGAAAGTCATAGGGTGTTTCTTCTTCTTGCAGATAATCAATAAAGAATCGAGTTCTAGCTCACCAGAGGTGAAAGAGAGCGCTCAGAGTCTCAACGAGTTGGCTTACATAAGGCAAGAGATCAAGAACCCTCTCAACGGTATCCGGTTTGCGCATAAGCTTCTTGAATCCTCGGAGATTTCAGAGAATCAAAGGCAGTTTCTGGAGACTAGTGATGCTTGTGAGAAGCAGATTGCGACAATAATCGAAGACACTGACTTGAAAAGCATTGAGGAAGGCAAGTTGCAAATGGAGACAGAGGAGTTTCGACTTGAGAGCGTCTTGGACACAATCATTAGCCAAGTGATGATTATGTTGAGAGAGAGGAAGTCACAACTCAGAGTTGAAGTCTCCCAGGAGATCAAAACGCTGCCTCTCTATGGTGACAGAGTAAAGCTTCAGCTCATTCTTGCTGATCTTCTGCGCAACATTGTGAATCATGCCCCGTTTCCAGATAGTTGGGTAGGAATCAAGATCTCACCAGGCCACAAGCTTGCACACGACAATAATCCCTATATCCATCTACAGTTCAGGATGATACATCCTGGAAAGGGACTTCCTTCAGAGATGCTAAGTGATATGTTCGAGACTAGAGAAGGATGGGTCACGCCTGATGGGTTAGGACTAAAGCTTTCAAGGAAACTGCTGGAGCAGATGAATGGTCGTGTGAGTTATGTTAGAGAAGACGAACGTTGTTTCTTTCAAGTGGATCTTCAAGTTAAGACAAGGTTAGGTGTTGAGACTGGTTCAAGCATATAG

>BrHP1(Bra023876)-DNA

ATGGAGTTGGTTCAGATGCAGAAGAGTTTGCAAGATTACACTAAATCACTCTTCTTGGATGTAAGTTTCTGCTATAGTCATTTTTTTGTTGGAAATAGAATATGAAGGTTGGTTGTTCAAAAAAAAAAAAGAATATGAAGGTTGAAGAAAGTGTTTTAGGAGTTATACAAAAACTGGAGTTTATGGTTAGAATTTAGAATTAATCGCTGTCCAGAAACTAAGGAAAGCTTTTGTAGTTTCTTTGGTAAGCACGAGTGTACGTTTGATTATGTAAATTTATAGTTTCTAATTATGGAAACTTCAGTAAAAAATTATGGAAAGTCTTGTTTTTGTATAAAAGGGGGTTTTGGACAGCCAGTTCTTGCAGCTGCAACAATTACAAGATGAAAGCAATCCAGATTTTGTTTCACAAGTTGTCACTCTCTTTTTCCAAGATTCTGACAGGATCCTCAATGATCTCTCACTGTCCCTGTAAGTAAATTCATTGCACACGCTCTCTCTCTTTCTCCTTAATCTTCCTCTTCTCTTTCTGTCTAGAACTTGTGTTTTCACTCGTCTTATTGCTTTCGCAGAGATCAACAAGTTGTAGACTTCAAAAAAGTTGATCCTCATGTTCATCAACTCAAAGGTAGCAGCTCCAGGTAACAATCAGAAAATATCAACAATATTTGGCTCTTCCATGCAAATGTATTTATGCACTTTGAGATCCTGAAATGTGTACATTAAGATTTTGACCGTGTGATGTTTCCAACAAACAGTATAGGAGCACAGAGAGTTAAGAATGCTTGTGTTGTCTTCCGCAACTTCTGCGAGCAACAAAACGTTGAAGGGTGAGTTTGATCGAATATGTTCCATTATTTTTTGTCTGTTTGATTTTAATATCTGATTATGAAAAAATTTTGATGAAAATCTCAGATGTCATAGATGTTTGCAACAAGTAAAGAAAGAATACTATCTTGTGAAGAACAGGCTAGAGACTCTGTTCAAGGTGAGGACTATGTACTATACCTTTATAATTCATCAAATTTAGCCATATTTGATTCATATTTATCTCATTTTGGTTGTTATTAACGAATCTACGCAGCTCGAGCAACAGATTGTAGCCTCTGGTGGAATGATCCCAGCCATGGAACTCGGATTTTGA

>BrHP1(Bra023876)-CDS

ATGGAGTTGGTTCAGATGCAGAAGAGTTTGCAAGATTACACTAAATCACTCTTCTTGGATGGGGTTTTGGACAGCCAGTTCTTGCAGCTGCAACAATTACAAGATGAAAGCAATCCAGATTTTGTTTCACAAGTTGTCACTCTCTTTTTCCAAGATTCTGACAGGATCCTCAATGATCTCTCACTGTCCCTAGATCAACAAGTTGTAGACTTCAAAAAAGTTGATCCTCATGTTCATCAACTCAAAGGTAGCAGCTCCAGTATAGGAGCACAGAGAGTTAAGAATGCTTGTGTTGTCTTCCGCAACTTCTGCGAGCAACAAAACGTTGAAGGATGTCATAGATGTTTGCAACAAGTAAAGAAAGAATACTATCTTGTGAAGAACAGGCTAGAGACTCTGTTCAAGCTCGAGCAACAGATTGTAGCCTCTGGTGGAATGATCCCAGCCATGGAACTCGGATTTTGA

>BrHP2(Bra036215)-DNA

ATGGACGCTCACGTCGCTCAGCTGCAGATGCAATATCGTAACTACATCCTTTCTCTCTACCAACAGGTTTGATTCATCATTCCCCCCCACACAAAACCCATTTCATTCCACCACATGTCTCTTTGGTCGTGACACATAGATAATCGATGGTAACTAAATCTAGCGATGCTTAAAGTGAATAAAGGTTGAACCTTTTGGTCGGGACACATAGATAATCGAATCTAGAGATGCTTTAAAGTGAATAAAGGTTGAATCTTTGGTTGGAAACTGTGATGGGTTTTGTTAGATCATTGCTTGGTTTTGATTTGTTTGTTGTTTTGGTTGTGAAGGGCTTTCTGGATGATCAGTTTACTGAGTTGAAAAGTCTGCAAGATGATGGAAGTCCTGATTTTGTGGCTGAGGTTCTCTCTCTCTTCTTTGATGATTGTGTCAAGCTTGTCGGTAACATGGCTAGAGCTTTGTAAGTCTCCTGCTCACATACATGTTCATCATACATCTGCTAGACCATCATCATCATCATTATCATCATTGCGTGTGTTTTTTATTTCAGGGACCAGACAGGAACTGTAGATTTTAGTCAGGTTGGTGCTAATGTGCATCAGTTGAAGGGTAGTAGCTCAAGGTATGTTTCTTTAGTTATCTTCTTGAGTTGTGGTTGATTGATGAAGCTCTGTTTTGTTTCTAATGTGCAGTGTTGGTGCCAAGAGGGTTAAAGGCTTGTGTATTAACTTCAAGGAACTTTGTGAGGCTAAGAACTATGAAGGGTGAGTGGTTTTATCTTCTTTTTTTTTGTATGGAGACTCATCGGTTGAGGATTTGTGGATTAATCTCTGATGTTTTCTTACAGGTGTGTGAGATGTCTGCAGCAAGTGGATATAGAGTACAAGACGTTAAAGGCAAAGCTTCAAGATATGTTCAATGTGAGTTGATGATTTTGCTTTTAGTGAATCATTTTGGTGAAGAGTGAATAAAATTTTCATGGCAATTGTGTTGTTGGTTGCAGCTTGAGAAACAAATAGTTCAAGCTGGTGGTATCGTTCCTCAAGTGGATATTAACTAA

>BrHP2(Bra036215)-CDS

ATGGACGCTCACGTCGCTCAGCTGCAGATGCAATATCGTAACTACATCCTTTCTCTCTACCAACAGGGCTTTCTGGATGATCAGTTTACTGAGTTGAAAAGTCTGCAAGATGATGGAAGTCCTGATTTTGTGGCTGAGGTTCTCTCTCTCTTCTTTGATGATTGTGTCAAGCTTGTCGGTAACATGGCTAGAGCTTTGGACCAGACAGGAACTGTAGATTTTAGTCAGGTTGGTGCTAATGTGCATCAGTTGAAGGGTAGTAGCTCAAGTGTTGGTGCCAAGAGGGTTAAAGGCTTGTGTATTAACTTCAAGGAACTTTGTGAGGCTAAGAACTATGAAGGGTGTGTGAGATGTCTGCAGCAAGTGGATATAGAGTACAAGACGTTAAAGGCAAAGCTTCAAGATATGTTCAATCTTGAGAAACAAATAGTTCAAGCTGGTGGTATCGTTCCTCAAGTGGATATTAACTAA

>BrHP3(Bra025394)-DNA

ATGGACGCTCTCGTTGCTCAGCTACAGATGCAGTATCGTAATTACACTGTTTCTCTCTACCAACAGGTTTGTTTCATCACTTACCCACAATCCCATTTCATTCGAGCAGAATTGGATATATATATATATATATATAGATCATTCAATGATACCTGAATCTATCGAAAGCCTGAATCTTTAGTGATTCTGTGTGTTGTTAGGGATTCGAATTCTCAAAGTAGTTTTCATTTCAAGGCTTGTTATTGAGAAAAGTTTGGAACTTTGCTTTTGTTTTGTGTGTTTTTGGGTTGTGTGAAGGGCTTTCTGGATGATCAGTTTACTGAGTTGAAGAAGCTGCAAGATGATGCAAGTCCTGATTTTGTGGCCGAGGTTCTCTCTCTCTTCTTTGAAGATTGTGTCAAGCTTATCGGCAACATGGCCAGAGCTTTGTGAGTTTCTCTTTTTATTTATTTTTTCATCATTTAACTCTGCCTGTCTTCTCTTACACGACTCAATCTATTTCAGGGACCAGACAGGAACTGTGGATTTTAGTCAGGTTGGTGCGAGCGTGCATCAATTGAAGGGTAGTAGCTCCAGGTATGTTTTGTTTTGAGCTTTTAACCAAATTCTTCTTGCGTGTGGTGGATAGATAGATGAAGAGCTCTGTCTTTGTGTATAATATGCAGTGTTGGTGCCAAGAGGGTGAAAGGCTTGTGTGTTACCTTCAAGGAGTACTGCGAGGCTAATAACTACGAAGGGTGAGGCTTTTTAATTATGGTTGAAACTCATTGGTTGAGATTGGTGAAATAATAATAAGTCTCTGATGTTAATTACAGGTGTGTGAGATGTCTGCAGCAGGTGGATATCGAGTACAAGGCGTTACAGACCAAGCTTCAAGACATGTTCAATGTGAGTAATAATTGTGAATAGTGAATCACTTGGTTGATTGAAACTTTCATGACATTGGTGTTTGTTGCTGCAGCTGGAGAAACAAATCATTCAAGCTGGTGGTAAAGTTCCTCAAGTGGATATTAACTAA

>BrHP3(Bra025394)-CDS

ATGGACGCTCTCGTTGCTCAGCTACAGATGCAGTATCGTAATTACACTGTTTCTCTCTACCAACAGGGCTTTCTGGATGATCAGTTTACTGAGTTGAAGAAGCTGCAAGATGATGCAAGTCCTGATTTTGTGGCCGAGGTTCTCTCTCTCTTCTTTGAAGATTGTGTCAAGCTTATCGGCAACATGGCCAGAGCTTTGGACCAGACAGGAACTGTGGATTTTAGTCAGGTTGGTGCGAGCGTGCATCAATTGAAGGGTAGTAGCTCCAGTGTTGGTGCCAAGAGGGTGAAAGGCTTGTGTGTTACCTTCAAGGAGTACTGCGAGGCTAATAACTACGAAGGGTGTGTGAGATGTCTGCAGCAGGTGGATATCGAGTACAAGGCGTTACAGACCAAGCTTCAAGACATGTTCAATCTGGAGAAACAAATCATTCAAGCTGGTGGTAAAGTTCCTCAAGTGGATATTAACTAA

>BrHP4(Bra028236)-DNA

ATGTTCACATATCACTCTCTGGATTTGGGTCCAAAGAATCAAACCGATGTTCTGGAGTGCTGTCGACAGAGTAATGATGCTTTAGATGAAGATAAGTACTTTGTGTTTCTAACTTTCTACAGAGAATCTTCCAGCGTGAGAGATTCTTGATGTCTGTGGGTTTTGGAAAAAGAAAACGCACAGAATGAATGAATGTATCAAATCATGAATGAGATTCCGTTTTTCATGGCCTAAATCAATCTATCTCACTTTCTCAACCTTGTCTTTAATTTTTATTTTGACAACTAATTATTGTATTCTTTATGGACTGAATATTTCTTTATTATTTTTGTCCAATAGAATAATGATCTGTAAGAATCTCTTTTATTGGGATTAGCGTTTCAAAGTTCAAAAACTCAGAACTGCTCATTCTGTTTTCCTATTATCAAACTTCAAATCTCCTCCTTCTTCTCTCTGCTTCATCGTCACAAGTAATCATGGACACACTCGTTTCTCAGTTGCAGAGACAATTTCGTGACTACACCATCTCTCTCTATCACCAGGTTTCTTCTTCGTGTTTTCCTCCCAAACTTACAGATAAAGTTAGTAAATTTTAGCATCTGGGTACGAAAATGAATTCAAAGGTGGATCCTTTTTGTTAGAGATGATCCTCTAAAGTTGTTAGGGTTCTTGATTTGGAAATTGAGGGTTAAAGAGTTAGTTTAAAGTTTCTGTGAAATGGGTTCTTGTTTATTTATCTAAAGGTGACTCCTTTTTGGCGATGATCTTCCTCTACAGTAGTTAGGGTTCTTGGATTTGAATTTGTAACTTGAGGATTTAAGAATTGGTTTTAAGTGTCTGTGAAATGGGTTATTGTCTCTTTTTTCCAAAGGTGTCTCCTTTATATATTTATAAGCTTTTCTTACTGAGAGCTTGAATTTGGAAATTAAGGCTTTGGCTGAGATTAGTTGTTTTGGTATGTGAAGGGTTTTCTGGATGATCAGTTCACTGAGCTGAAAAAGCTGCAAGATGAATGTAGCCCTGATTTTGTGGCAGAGGTTGTTTCTCTCTTCTTTGAAGACTGTGAGAAGCTTATCGGTAACATGGCTAGAGCTCTGTAAGTGTTTTTTCTTTTTGACCTTTGTGTGCACATCTAGTATAATCAAAAGATATGTTTTGGTTTGATATTTGTTTTTGTACATACTTCAGAGATCAGACAGGAAACGTAGATTTCAGTCTGGTAGGTTCTAGTGTTCATCAACTCAAAGGTAGTAGCTCAAGGTAATGTTTACTCAGATCTGTAATCTCACTCCTTTGTCTCTAATCTCCTACTTAGTCTTAGTGAAGAGTTCTTTCTTGTTTCTATGTAGTGTTGGTGCCAAGAGGGTCAAAGGTCTTTGTGTTACCTTCAAGGAGTGTTGTGACTCTCAGAACTTTGAAGGGTAAGCTCATTTTCTGCTATGTTCATTTTTTTTATTGTTGAGACTAATTTGGGGAACAATTCTGCTTCAGGTGTGTGAGATGTTTGCAGCAGGTGGATATTGAGTACAAGTCATTGAAGGCAAAGCTTCAAGATTTGTTCAGTGTAAGTGACAAAAGATTATGATAAACTGTGAAAGAACATAGCTTTTTTTATAAATGATTCTTGTATTGTCTTTTGCAGCTTGAGCAGCAGATTGTCCAAGCTGGTGGTAGAATCCCTCAGGTTGATATATAA

>BrHP4(Bra028236)-CDS

ATGTTCACATATCACTCTCTGGATTTGGGTCCAAAGAATCAAACCGATGTTCTGGAGTGCTGTCGACAGAGTAATGATGCTTTAGATGAAGATAAGTACTTTGTGTTTCTAACTTTCTACAGAGAATCTTCCAGCGGTTTTCTGGATGATCAGTTCACTGAGCTGAAAAAGCTGCAAGATGAATGTAGCCCTGATTTTGTGGCAGAGGTTGTTTCTCTCTTCTTTGAAGACTGTGAGAAGCTTATCGGTAACATGGCTAGAGCTCTAGATCAGACAGGAAACGTAGATTTCAGTCTGGTAGGTTCTAGTGTTCATCAACTCAAAGGTAGTAGCTCAAGTGTTGGTGCCAAGAGGGTCAAAGGTCTTTGTGTTACCTTCAAGGAGTGTTGTGACTCTCAGAACTTTGAAGGGTGTGTGAGATGTTTGCAGCAGGTGGATATTGAGTACAAGTCATTGAAGGCAAAGCTTCAAGATTTGTTCAGTCTTGAGCAGCAGATTGTCCAAGCTGGTGGTAGAATCCCTCAGGTTGATATATAA

>BrHP5(Bra027169)-DNA

ATGGGAAAATGCATGCAGGGATTTCTCGACGAACAATTCATGGAGTTAGAAGAGCTCCAAGATGATGTAAACCCTAATTTTGTTGAAGAAGTTGCCACATTATACTTCAAAGATTCAGCTAGGTTAATCAATAGCATTGACCAAGCGCTGTGAGTACCTTTATCCTTTTCATATTGATGTTTCTAATTGTGCACAAAATTTCAAGTTTTCTAATAAACGAGAATCACAATCCAGGGAAAGAGGATCATTTGATTTCAATCGGCTGGATAACTACATGCATCAGTTTAAGGGCAGCAGCAGCAGGTAAATATTCCCCTTAATTTTGATACACTTATTATAGAAATGAGATATACTCCATCTGTTCCTTAATGATAGACATTTTAGAAAAAAATTTTGTTTCAGAAAAATGTATTTCTTGTGTTTTCTATGAAAAAATTGTAAACTTCGAGAAAATTAATTGAATTACTATTGGTTAAAAGTTATCGAAAATTGAAAATTACAGGAAACGATATATTTATTATGGTAGTTTAATGTATTTTCTTAATATGTGTGAAAATACTGAAAAATCTATCTTTGTGGAACAGATGGAGTAGTGAGTACTTTGAATAAATTTTGAATATAAGTAAAAAGAGTGTAGCACCAGCACCCTGAAAGTTACTTCCATTCTCATTTATGATTAAATGACAAAATTTACTAGACATATTTTTATTTTGAATTATAAGTTTTAGAACTATTAAATAATAGATACTTTTGTTAGAAACGGAGTTAACAGTCACATAACGTGCATTTTTCTCTTTTTGTAGCATTGGTGCGAGTAAGGTGAAAACTGAATGCACTATGTTTAGGGAATACTGCAGAGTTGGAAACGCCGAAGGGTATCATTCCATTAACACCTGCCTACTAGCTTTTTTATACAGAAATATAAACTCACGGGGTCAAAGTGAAAACAAGTAAAACATATAGGGTTAAAACTGCAGTAATTAACCCATTGAACCTTGAACGTTTGTTGTACTTAATCTTCTCACTTATTCCATACTTGCGACGAGTGCAGATGCTTGAGGACTTTCCAGCAAGTGAAAAAAGAGCACGCAACGTTGAGAAAGAAGCTTGAACATTATTTCCAGGCAAGCCAATGA

>BrHP5(Bra027169)-CDS

ATGGGAAAATGCATGCAGGGATTTCTCGACGAACAATTCATGGAGTTAGAAGAGCTCCAAGATGATGTAAACCCTAATTTTGTTGAAGAAGTTGCCACATTATACTTCAAAGATTCAGCTAGGTTAATCAATAGCATTGACCAAGCGCTGGAAAGAGGATCATTTGATTTCAATCGGCTGGATAACTACATGCATCAGTTTAAGGGCAGCAGCAGCAGCATTGGTGCGAGTAAGGTGAAAACTGAATGCACTATGTTTAGGGAATACTGCAGAGTTGGAAACGCCGAAGGATGCTTGAGGACTTTCCAGCAAGTGAAAAAAGAGCACGCAACGTTGAGAAAGAAGCTTGAACATTATTTCCAGGCAAGCCAATGA

>BrHP6(Bra001629)-DNA

ATGCAGAGGCAAGTGGCACTCATTAAGCAGTCCCTCTTTGATCAGGTACAATGTTTATTCCTTTTATTGTGAACATATGAATTTCTTTTTTAGTGTTAGTGTAGTATTTATATTAGTACCAGTTGTGAATTTCTTCAAAGAAAATAATAATTAAAACTCAAATGGAAAAATACATGCAGGGATATCTCGATGAACAATTCATAGAGTTAGAAGAGCTCCAAGATGATGCAAACCCTAATTTTGTTGAAGAAGTTGCAACATTATACTTCAAAGATTCAGCTCGATTAATCAGTAACATTGAGCAAGCTTTGTGAGTAACTTTAATTTTCATATTTATTTTTTCTATTTTTGAACAAGACTTTGAGTTTTCTAATGTACACAAATTACTTTTAACAATGCAGGGAAAGAGGATCATTTGATTTCAATCGGCTAGATAATTACATGCATCAGTTTAAGGGCAGCAGCACAAGGTATAAAATTTCCCCTGAACTTTGATAAATTTACATATAGAAATGCGACTACTTGAGTACTTAGTATGTGTTTAAGTCAAAAATAACACAAGCACATTTAAATTACTTCCATATTCGATCAAGAATATTAATTACTTCCACTCTGATTCATGATTTAATGATATTTACTAGACTTAACATAAATATGTTTATTATAGTTTTATATAATAATAAATAGTAGCATTTGTTAAAACGTAATTAGTAATGACATAATGTGCTTTTTTCTTGTAGCATCGGTGCGAGTAAGGTGAAAACTGAATGCACTATGTTTAGGGAATACTGCAGAGTTGGAAATGCTGAAGGGTACTTTCCCTTTACACCTTCTTGCTTCTGTTTTAACGGAATATAAATTCACCGGGTGAAAATTAACAAGAAAAGTATGGAGTTAAAATTGCAGCAATATCCGTTGAACCTTGAACTTTTCTGCTTTATTTAATTTTCAATGCCCTAAATAGTTTTCTCACTATTCCGTACTTGCGACGAGTGCAGATGTTTGAGGACTTTCAAGCAACTGAAGAAAGAGCACGCAACGTTGAGAAAGAAGCTTGA

>BrHP6(Bra001629)-CDS

ATGCAGAGGCAAGTGGCACTCATTAAGCAGTCCCTCTTTGATCAGGGATATCTCGATGAACAATTCATAGAGTTAGAAGAGCTCCAAGATGATGCAAACCCTAATTTTGTTGAAGAAGTTGCAACATTATACTTCAAAGATTCAGCTCGATTAATCAGTAACATTGAGCAAGCTTTGGAAAGAGGATCATTTGATTTCAATCGGCTAGATAATTACATGCATCAGTTTAAGGGCAGCAGCACAAGCATCGGTGCGAGTAAGGTGAAAACTGAATGCACTATGTTTAGGGAATACTGCAGAGTTGGAAATGCTGAAGGTTTTCTCACTATTCCGTACTTGCGACGAGTGCAGATGTTTGAGGACTTTCAAGCAACTGAAGAAAGAGCACGCAACGTTGAGAAAGAAGCTTGA

>BrHP7(Bra033398)-DNA

ATGAACACCGTCGTTGCTAAGTTGCAGAGACAGTTTCAAGACTACCTCGTTTCTCTTTATCAACAGGTTGGATTTGAAACTGAGTGAGCTCCTCTTTGGTTGATTGTTTTAGTTTGATTTGACTTGGGGTTATAGAAATAGTTTCAGGGTTATATATAAGCTTTTATGGTAGGTACTACTCTATGCATTTTAAATTTGAAGGCTTTTTAAAAAAGAATACTATCTTCTTTTTTTTTTAAAGGGATTTCTGGATAATCAGTTCACTGAGTTGAGAAAGTTGCAAGATGAAGGCACTCCTGATTTTGTAGCTGAGGTTGTCTCTCTTTTCTTTGACGACTGTTCCAAGCTTATTAATAGCATGTCTAGATCACTGTAAGTCTTCCATCAGCTCTGTTATGTGTGTACTTGTTTTTTTTTTTTTGTCAAGAATGTTATGTGTGTACTGTGTCTGGAACTTCGTGTCTTATAATTAGGGAGAGGCCAGAAAATGTGGATTTCAAACAGGTTGATTCAGGGGTGCATCAACTCAAGGGTAGTAGCTCAAGGTATATCCATGATCATGTTTTTTTTATAGAGGAAGCGGTGAACCTTTCTTCATTGTAATTATGAAGTTTTAAGAAATCAAAATACATTGATTTAATGGCTCACTCTCTGCTTTGTACACAGTGTTGGTGCAAGGAGGGTAAAAAATGTGTGTATATCTTTCAAGGAATGTTGTGATGTTCAGAACCGTGAAGGGTGAGTGCTGTCCAAAACAAGTTGCCACCTTTTTCTCCAGTGTATAAGTTTAGATTGTTGATGCGAAATGTTATTGGATTTGTTTGATTTCTGAAATTAATTATTCTAAATTATGATAATGTTATATGGTGTAGGTGTTTAAGGTGTCTGCAGCAGGTGGATTATGAATATAAGATGCTAAAGACCAAACTTCAGGATCTCTTCAATGTGAGTTCTACACTTTGCATAAAACCTACTTGAGTTCTGCTCTCGAACCCTTTTCTTAATGCAATCTTTTTAATGTAATGTCGTTGCAGCTAGAGAAACAGATCGTCCAAGCCGGAGGTGCGATTCCTCAGGTGAATATAAATTAG

>BrHP7(Bra033398)-CDS

ATGAACACCGTCGTTGCTAAGTTGCAGAGACAGTTTCAAGACTACCTCGTTTCTCTTTATCAACAGGGATTTCTGGATAATCAGTTCACTGAGTTGAGAAAGTTGCAAGATGAAGGCACTCCTGATTTTGTAGCTGAGGTTGTCTCTCTTTTCTTTGACGACTGTTCCAAGCTTATTAATAGCATGTCTAGATCACTGGAGAGGCCAGAAAATGTGGATTTCAAACAGGTTGATTCAGGGGTGCATCAACTCAAGGGTAGTAGCTCAAGTGTTGGTGCAAGGAGGGTAAAAAATGTGTGTATATCTTTCAAGGAATGTTGTGATGTTCAGAACCGTGAAGGGTGTTTAAGGTGTCTGCAGCAGGTGGATTATGAATATAAGATGCTAAAGACCAAACTTCAGGATCTCTTCAATCTAGAGAAACAGATCGTCCAAGCCGGAGGTGCGATTCCTCAGGTGAATATAAATTAG

>BrPHP1(Bra003551)-DNA

ATGTTGGGGTTGGGTGTGGACCGGCTTCAAGCCGACATCAACCGCCTTCTAACCTCCCTTTTCCATCAGGTTGAACTTACTATGTAAAACTATCTCTCCATTCACATTTTCTTTATTTATGTGTTGGTTCTATAATTAAAAAGGGATGCTGGATATTACATGTGATGAGTGTAGGGAGTGCTGGACGAGCAGTTCTTGCAGCTGCAGCAGCTTCAAGATGAAACTTCACCAACATTTGTGTACGATGTCATTAATATCTACTTTGACGAATCCGAGAAGCTACTCCGCAGCCTTAGATTATTGCTGTTAGTTCCATGATCTGTCTATCTTTTTTTCCTCTGAAGAAAAATATTGCGTGTCGAAAACTATTAGGTCCAAGAATATGTAAATGAATATACAAATGAATGAACACAGGATGGATAGAGAATTCTCCGACTACAAGAAGATAGGATTGCATCTGAATCAGTTGGTAGGAAGCAGTTCCAGCATTGGTGCTCGTAGGGTTCGTAACGTCTGTGTTGCCTTTCGCTCTGCTTCCGAGCTTAACAACCGCCCAGGGTATACAAATTATTAAACGCTATTTCATTGTACTTATATTACATACAAAAATGTGTATATAATTAATGACTATTTTCAATATCATCATGATACATCAGGTGCTTGAGAGGTCTGGAGATAGTAGAGCATGAGTATCATTACCTCAAGAATATGATGCATGAACTCTTCCAGGTACGAGTTACACACCAAAGTACAGTCTATATATAACTTGGTCGACCAACCGTATATAAATAAACATGAAGCAAACATATATCATGGTAATTATTGATGTTACACTAATTTTGTTGGATTTAATCTGAATATGGTTGCAGCTGGAGCAGCAGAGGCTATTAGCTGCAGGAGTCAGATATCCAATGTAG

>BrPHP1(Bra003551)-CDS

ATGTTGGGGTTGGGTGTGGACCGGCTTCAAGCCGACATCAACCGCCTTCTAACCTCCCTTTTCCATCAGGGAGTGCTGGACGAGCAGTTCTTGCAGCTGCAGCAGCTTCAAGATGAAACTTCACCAACATTTGTGTACGATGTCATTAATATCTACTTTGACGAATCCGAGAAGCTACTCCGCAGCCTTAGATTATTGCTGATGGATAGAGAATTCTCCGACTACAAGAAGATAGGATTGCATCTGAATCAGTTGGTAGGAAGCAGTTCCAGCATTGGTGCTCGTAGGGTTCGTAACGTCTGTGTTGCCTTTCGCTCTGCTTCCGAGCTTAACAACCGCCCAGGGTGCTTGAGAGGTCTGGAGATAGTAGAGCATGAGTATCATTACCTCAAGAATATGATGCATGAACTCTTCCAGCTGGAGCAGCAGAGGCTATTAGCTGCAGGAGTCAGATATCCAATGTAG

>BrRR1(Bra027829)-DNA

ATGCCTCTAGACGGTGGCGTTTCTTGTCGACGCAGGTCAGAGATGATCGGAATAGGAATAGGAGAACTCGAATCTCCGCCGCTGGATTCAGACCAAGTTCACGTTCTAGCCGTTGACGACAGCCTCGTCGATCGTATCGTCATAGAAAGATTGCTTCGTATTACTTCTTGCAAAGGTTTGTTTGTCTTCTTTTTTCTTCATTTCAAAATAAATATATAAAAACTGATCTACTGAGATTCAAATGCAGTCACGGCGGTTGATAGCGGATGGCGTGCTCTGGAGTTCTTAGGGCTGGACGATGATAAATCCTCCGTAGAATTCGATGTAATCTCTCAAAAGAATCAGTTTTAAGTTTATAAGAGCTTTTTTGAGATTTTTACATAATTTGATTTTGATTTTTGAATCTTTCAGAGATTGAAGGTGGATTTGATCATAACTGATTACTGTATGCCTGGAATGACGGGATACGAGCTTCTCAAGAAGATTAAGGTTTTGAATATTTTCCCGCCATGAAAATAAGTTTCTTAAGCTGAACAGTGTGGATTTTTTTTTTGAAACAGGAGTCGACTAGTTTCCGAGAAGTTCCGGTTGTGATAATGTCGTCGGAGAACGTTCTCACTCGAATCGACAGGTTAATCTTTTTTCGGAATATTTTTAAATTTTGAAAAGTTAAATTCAAAAAAATAATAAAAAATGCAGATGCCTTGAAGAAGGTGCGGAGGATTTCTTGTTGAAGCCTGTGAAACTCGCCGACGTGAAACGCCTGAGGACTTACTTGACGAGAGACGTTAAAGTCTCCGACGGAAACAAACCCAAGGTTCCTGAAGATCTCAGCCGTTTCTCTTCTTTGGCTATGGTTACTCCTCCTCCGCCATCCATTACCTCGGTGGAATCCGTGTCTCTGTCGCCGGAGTCTTCGGTTTCGCCGGTGGATTCGCCGATAAGACCGATGGAGATGAGGAGTCCCGGATTAGATTAG

>BrRR1(Bra027829)-CDS

ATGCCTCTAGACGGTGGCGTTTCTTGTCGACGCAGGTCAGAGATGATCGGAATAGGAATAGGAGAACTCGAATCTCCGCCGCTGGATTCAGACCAAGTTCACGTTCTAGCCGTTGACGACAGCCTCGTCGATCGTATCGTCATAGAAAGATTGCTTCGTATTACTTCTTGCAAAGTCACGGCGGTTGATAGCGGATGGCGTGCTCTGGAGTTCTTAGGGCTGGACGATGATAAATCCTCCGTAGAATTCGATAGATTGAAGGTGGATTTGATCATAACTGATTACTGTATGCCTGGAATGACGGGATACGAGCTTCTCAAGAAGATTAAGGAGTCGACTAGTTTCCGAGAAGTTCCGGTTGTGATAATGTCGTCGGAGAACGTTCTCACTCGAATCGACAGATGCCTTGAAGAAGGTGCGGAGGATTTCTTGTTGAAGCCTGTGAAACTCGCCGACGTGAAACGCCTGAGGACTTACTTGACGAGAGACGTTAAAGTCTCCGACGGAAACAAACCCAAGGTTCCTGAAGATCTCAGCCGTTTCTCTTCTTTGGCTATGGTTACTCCTCCTCCGCCATCCATTACCTCGGTGGAATCCGTGTCTCTGTCGCCGGAGTCTTCGGTTTCGCCGGTGGATTCGCCGATAAGACCGATGGAGATGAGGAGTCCCGGATTAGATTAG

>BrRR2(Bra031714)-DNA

ATGGCCAGAGACGGTGGCGTTTCTTGCCTACGGAGTTCAGAGATGATGAGGGTCGGTATCGGAGGAATGGAATCTCCGCCGTTGGATTTAGATGAAGTTCATGTCCTGGCTGTTGATGACAGCCTCGTTGATCGGATTGTCATTGAGAGATTGCTTCGTATTACCTCCTGCAAAGGTCAGCCTTACTATTTTTTTAATTAATTTCATTTAATCTTTTCGCCGGAAAGTTACTAAAAATAGAAATATTTATTCGGCGGTTCTGTTTCAGTTACGGCGGTAGATAGCGGATGGCGTGCTCTGGAGTTTCTAGGACTAGACAACGAGAAAGCATCTGCAGAACTAGATGTAAGTCTTGGAACTTGAAGATATTTTTATATGATTAGTGATCAAAATTTGATTTTAGCAACAAATTTTAGCAAAAATAAAATTTTGATTTTAGTATTTTTTTTGTTACTGATTCTCAGAGATTGAAAGTTGATCTGATCATCACCGATTACTGTATGCCTGGAATGACTGGCTACGAGCTCCTCAAAAAGATCAAGGTCTGAACTTTCCCACTATTTTACAGATTATAATTACAATTTATTTTTGACCAAAAATAATTTACAATTTATTGTTGAATCCTTTCGGACCAATGTGGTTTTTTCCGTTAAACTGAAACAGGAATCGTCCAGTTTCCGACAAGTTCCGGTTGTAATAATGTCGTCAGAGAATGTCTTAACCAGAATCGATAGGTGGGTTTTGTAATAAATTTGCAAACTTTTTTTTGTTGGGTTTTGAGAAACTGTTGATTGATAAAAAAAAAACGCTAAACTGATCTCGGCAGATGTCTTGAGGAAGGGGCGGAGGACTTCTTACTAAAACCGGTGAAACTCGCCGACGTGAAGCGCCTGAGAAGCTATTTAACCGGAGACGTTAAACTTTCCAACGCAAACAAACGGAAGCTTCCGGAAGATTCTGTTCCCGTCAACACCTCGTTTCCTCCTCCGCCGTCGCCGTTTCCTATTTCGCCTGATTCGTCGGACTCTTCTCTGCCGTTGACTATCTCGCCTGAATCGTCGGACTCTTCTCCGCCGTTGTCTCCCCTGGAAATATCTTCCTCGCCACTCTCATCGCCAATAGACGATGAAGATGATGATGTGTTGACATCGTCGCCGGCGCCGGAGGAATCGCCGGCTCGACGGCAGAAGATGAGAAGTCCTCTGGATTAG

>BrRR2(Bra031714)-CDS

ATGGCCAGAGACGGTGGCGTTTCTTGCCTACGGAGTTCAGAGATGATGAGGGTCGGTATCGGAGGAATGGAATCTCCGCCGTTGGATTTAGATGAAGTTCATGTCCTGGCTGTTGATGACAGCCTCGTTGATCGGATTGTCATTGAGAGATTGCTTCGTATTACCTCCTGCAAAGTTACGGCGGTAGATAGCGGATGGCGTGCTCTGGAGTTTCTAGGACTAGACAACGAGAAAGCATCTGCAGAACTAGATAGATTGAAAGTTGATCTGATCATCACCGATTACTGTATGCCTGGAATGACTGGCTACGAGCTCCTCAAAAAGATCAAGGAATCGTCCAGTTTCCGACAAGTTCCGGTTGTAATAATGTCGTCAGAGAATGTCTTAACCAGAATCGATAGATGTCTTGAGGAAGGGGCGGAGGACTTCTTACTAAAACCGGTGAAACTCGCCGACGTGAAGCGCCTGAGAAGCTATTTAACCGGAGACGTTAAACTTTCCAACGCAAACAAACGGAAGCTTCCGGAAGATTCTGTTCCCGTCAACACCTCGTTTCCTCCTCCGCCGTCGCCGTTTCCTATTTCGCCTGATTCGTCGGACTCTTCTCTGCCGTTGACTATCTCGCCTGAATCGTCGGACTCTTCTCCGCCGTTGTCTCCCCTGGAAATATCTTCCTCGCCACTCTCATCGCCAATAGACGATGAAGATGATGATGTGTTGACATCGTCGCCGGCGCCGGAGGAATCGCCGGCTCGACGGCAGAAGATGAGAAGTCCTCTGGATTAG

>BrRR3(Bra018439)-DNA

ATGGCCAAAGATGGTGGCGTTTCTTGTCTACGTAGCTCAGAGATGCTGAACGTCGGTATCGGAGGAATGGAACCTCCACCGTTAGATTTAGATGAAGTTCATGTCTTGGCCGTCGATGACAGCCTCGTCGATCGGATTGTCATCGAGAGATTGCTTCGTATTACCTCCTGCAAAGGTTCGTGACGTCTTCCTACTTATTCATTTATTTTAGATACAAATGATTTTATTCTAGCCGGATATTCTTTAAAGAAGGTTTTTTTTCGGTGGTTCTGTTTCAGTCACGGCGGTTGATAGCGGATGGCGTGCTCTAGAGTTTCTAGGACTAGATAACGATAAGGCATCTGACGAATTTGATGTAAGTTGGTATATTTAATTGTAACGCCATCTCCAACCATGAGCAAAATTAATGTTTAGTTTTTTTTTAGAAGAAAAAAATATTAAGGTGAATTGGAGAGGCTCTTAAGATTATTCTGATTTTTAGTTGTTTATTTACTGATTCTGTAATTTCAGAAATTGAAAGTTGATATGATCATAACTGATTACTGTATGCCTGGAATGACCGGTTACGAGCTTCTCAAGAAAATCAAGGTCTCAACTTTCCCGCCTTTTTTCGATTCAGACAACAATTTATTTTAATAATGTAAATATGAAACAGGAATCGTCCAGTTTCCGACAAGTCCCAGTTGTAATAATGTCGTCGGAGAACGTGTTGACCAGAATCGACAGGTGCCTTGAAGAAGGTGCGGAGGATTTTTTACTGAAACCGGTGAAACTCGCCGACGTGAAGCGACTTAGAAGCTGTTTAACCGGAGACGTTAAACTTTCAAACGGAAACAAACGGAAGCTTCCGGAAGATTCTGTTTCCGTCGACACTTCGCTTCCACCGCCGCCGCTATCCTTGACTTTCTCCACTAATTCGTCGGACTCTTCTCCGCCGTTATCTCCCGTGGAAGTCTTTTCTTCGCCACTCTCATCTCCTGAAGATGATGATGATGTGTTGACTTCGTCGCCTGAGGGTTCGCCGATGTCGGAGGAGTCGCCGATTCGACGGCAGAAGATGGGGAGTCCCGGATTAGATTAG

>BrRR3(Bra018439)-CDS

ATGGCCAAAGATGGTGGCGTTTCTTGTCTACGTAGCTCAGAGATGCTGAACGTCGGTATCGGAGGAATGGAACCTCCACCGTTAGATTTAGATGAAGTTCATGTCTTGGCCGTCGATGACAGCCTCGTCGATCGGATTGTCATCGAGAGATTGCTTCGTATTACCTCCTGCAAAGTCACGGCGGTTGATAGCGGATGGCGTGCTCTAGAGTTTCTAGGACTAGATAACGATAAGGCATCTGACGAATTTGATAAATTGAAAGTTGATATGATCATAACTGATTACTGTATGCCTGGAATGACCGGTTACGAGCTTCTCAAGAAAATCAAGGAATCGTCCAGTTTCCGACAAGTCCCAGTTGTAATAATGTCGTCGGAGAACGTGTTGACCAGAATCGACAGGTGCCTTGAAGAAGGTGCGGAGGATTTTTTACTGAAACCGGTGAAACTCGCCGACGTGAAGCGACTTAGAAGCTGTTTAACCGGAGACGTTAAACTTTCAAACGGAAACAAACGGAAGCTTCCGGAAGATTCTGTTTCCGTCGACACTTCGCTTCCACCGCCGCCGCTATCCTTGACTTTCTCCACTAATTCGTCGGACTCTTCTCCGCCGTTATCTCCCGTGGAAGTCTTTTCTTCGCCACTCTCATCTCCTGAAGATGATGATGATGTGTTGACTTCGTCGCCTGAGGGTTCGCCGATGTCGGAGGAGTCGCCGATTCGACGGCAGAAGATGGGGAGTCCCGGATTAGATTAG

>BrRR4(Bra019932)-DNA

ATGGCCAGCGACGGTGGCGTTTCATGTCTACGGAGGTCGGAGATGATGAGCGTCGGCATCGGAGGAATAGATTCTCCACCGTTGGATGTAGACGAAGTTCACGTCCTGGCCGTCGACGACAGCCTCGTTGATCGGATTGTCATCGAGAGATTGCTTCGTATTACCTCCTGCAAAGGTTCGTCTTCCTAATTTATAAAATAACTAAAAATGTATTTAGACATCGAAAAATCTAAAAAAATAAAACACATCTCGGGCGGTTCTGTTTCAGTCACGGCGGTAGATAGCGGATGGCGTGCTCTGGAGTTTCTAGGGATAGACAACGAGAAAGCCTCTGCAGAACTCGATGTAAAGTCTTCGAATTTAAAGAGATTTTTCTTTTATAATTAGTGATCAAGATTATTGATATTTTTTTTAGTTTTCTTACTGATTCTTAGAGATTGAAAGTTGATCTGATCATCACCGATTACTGCATGCCTGGAATGACTGGTTACGAGCTCCTCAAGAAGATTAAGGTTTGAATTTCACCGCCGTTTTACATAATTATTTATTTTTATTTATTTTTGAATCCTGTCGGAACAATGTGGAATTTTCCGGAAATCTGAAACAGGAATCGTCCAGTTTCCGACAAGTCCCGGTTGTAATAATGTCGTCGGAGAATGTAATAACCAGAATCGACAGGTGAGTTTTCGTTATCAATCCTCAGGCTTTGTTGATTTCGAGTTAGAGTAAACGGTTGACTGATAAAACTGATTCCGGCAGGTGTCTTGAGGAAGGTGCGGAGGACTTCTTACTGAAACCGGTGAAACTCGCCGACGTGAAACGCCTGAGAAACTATTTAACCAGAGACGTTCAAGTTTCCAACGGAAACAAACGGAAGCTTCCAGAAGATTCGCCGCCGTTGACTCTCTCGCATGATTCGTCGGACTCTTCTCCGCCGCCGTCGACTCTCTCGCCTGATTCTTCGGACTCTTCTTCCCCGCCGTTATCTCCAGTGGAGATCTTTTCCTCGCCGCTACTTTCTCCATTAGACGATGAAGATGACGATGTGCTGACAACGTCGCCGGAGTCTACTCCGTCGCCGGTTCGACGGCAGAAGATGAGGAGTCCCGGATTAGATTAG

>BrRR4(Bra019932)-CDS

ATGGCCAGCGACGGTGGCGTTTCATGTCTACGGAGGTCGGAGATGATGAGCGTCGGCATCGGAGGAATAGATTCTCCACCGTTGGATGTAGACGAAGTTCACGTCCTGGCCGTCGACGACAGCCTCGTTGATCGGATTGTCATCGAGAGATTGCTTCGTATTACCTCCTGCAAAGTCACGGCGGTAGATAGCGGATGGCGTGCTCTGGAGTTTCTAGGGATAGACAACGAGAAAGCCTCTGCAGAACTCGATAGATTGAAAGTTGATCTGATCATCACCGATTACTGCATGCCTGGAATGACTGGTTACGAGCTCCTCAAGAAGATTAAGGAATCGTCCAGTTTCCGACAAGTCCCGGTTGTAATAATGTCGTCGGAGAATGTAATAACCAGAATCGACAGGTGTCTTGAGGAAGGTGCGGAGGACTTCTTACTGAAACCGGTGAAACTCGCCGACGTGAAACGCCTGAGAAACTATTTAACCAGAGACGTTCAAGTTTCCAACGGAAACAAACGGAAGCTTCCAGAAGATTCGCCGCCGTTGACTCTCTCGCATGATTCGTCGGACTCTTCTCCGCCGCCGTCGACTCTCTCGCCTGATTCTTCGGACTCTTCTTCCCCGCCGTTATCTCCAGTGGAGATCTTTTCCTCGCCGCTACTTTCTCCATTAGACGATGAAGATGACGATGTGCTGACAACGTCGCCGGAGTCTACTCCGTCGCCGGTTCGACGGCAGAAGATGAGGAGTCCCGGATTAGATTAG

>BrRR5(Bra033773)-DNA

ATGGCGGAGGTTTTGCGTCCGGAGATGTTGGACATATCCAACGACACTTCTTCCTTAGCGTCGCCGGAGCTTCTTCACGTTCTTGCCGTCGACGACAGCATCGTCGATCGGAAGTTCATCGAGCGGTTGCTCAGAGTATCTTCTTGTAAAGGTAAGAAGGAGAAGTTTCTGTTCATCTTGAGGAATCTGTTTGATGTTCGAAATCTGACAGTTTGTTTTTGAAAATTGTCAGTTACTGTTGTCGATAGCGCGACAAGAGCCTTGCAATACCTTGGATTAGATGGAGACAATAGTTCTGTTGGATTTCAGGTAATAAATCTGATTGAACTGATCCTTAATTAGTGTTGAGATTCCATTCAAATTTGGTATTGAAGTTTTTGAATAATATTTCTCCGGACCTGAAGATTAATCTGATAATGACGGATTACTCTATGCCTGGGATGACTGGTTATGAACTATTGAAGAAGATCAAAGTAAGCTTTTCTGTTGATTTGGAAAATTAAAATAAAAAGTTTTTGATCTAACCTGTTTTATGGGTTTTGAAATTCTATAGGAATCATCAGCTTTCAGAGAAATACCGGTTGTGATTATGTCCTCAGAGAACATCTTGCCTCGTATAGATAGGTATGTTCTCTTCTACTGTTATCTTAATCACCATCCTTAAGTCTTTGTTTTAAGTTCAGAATCATCTAATCTAAGAATAGCCATGATTAACCAGTTATAGTAATTTTATTAGATTTTGTTGCATTTATTACTTTGGGATGCTCAAAAGGGGGTTTGTTTAGGGAATCAAGTTTTCTTTTTTTTGAAAGATACCGTTTAGCAATATGCATTGACCTAATTAAATTTATTTTAATAATCATATCAGATTAGTAAGATAATGAATATGATATGGTTTCTCTAAACCATCGTGGGTAACACTGGTGGTGGTTGTAACTTTTGATCAAAGCTAAAGATTTTGGTGTTACTTTAGTGGGTCCTCGAAAGATTTGATAAGAGGATATTAGGAATATGTAATAATTAAAAAAATTGTTTCTTGTGGCCCGAAAATAAGAAAAGAATAATCCGTCATAAGTTAAGCAAGATCTTATCTTGAGATTTGATTCTATTGCCGGTGATGTGTTTGTTAATAACCCCAAATTTTTAAATATAATATATGCTTTTAATTTTGTTTGCATAATCTTTGGTAGGGTCATTTATAAATCCAATCCTCGTGATAGACACCTCACATGCATTTGTTTATTATTAAGGAACCATCTTTACCAAAATTAAAGTCTAATCATGTAGTTGCTTGTTGTTATGAGCTGGCCATTTTCTTGTTCCAAAGCTATGAATTTTATGGAATTAGATCACTAATATTTGCTTATGTATACAGATGTCTTGAAGAAGGTGCTGAAGATTTCTTATTGAAGCCTGTGAAGTTGGCTGATGTGAAGAGATTAAGAGATTCCTTAATGAAAGCTGAGGAAAGAGTATTCAAGAATATTATGCACAAGAGGGAGCTAGAAGCTAATGATATCTACTCACAGCTAAAACGCGCAAAGATCTAA

>BrRR5(Bra033773)-CDS

ATGGCGGAGGTTTTGCGTCCGGAGATGTTGGACATATCCAACGACACTTCTTCCTTAGCGTCGCCGGAGCTTCTTCACGTTCTTGCCGTCGACGACAGCATCGTCGATCGGAAGTTCATCGAGCGGTTGCTCAGAGTATCTTCTTGTAAAGTTACTGTTGTCGATAGCGCGACAAGAGCCTTGCAATACCTTGGATTAGATGGAGACAATAGTTCTGTTGGATTTCAGATTAATCTGATAATGACGGATTACTCTATGCCTGGGATGACTGGTTATGAACTATTGAAGAAGATCAAAGAATCATCAGCTTTCAGAGAAATACCGGTTGTGATTATGTCCTCAGAGAACATCTTGCCTCGTATAGATAGATGTCTTGAAGAAGGTGCTGAAGATTTCTTATTGAAGCCTGTGAAGTTGGCTGATGTGAAGAGATTAAGAGATTCCTTAATGAAAGCTGAGGAAAGAGTATTCAAGAATATTATGCACAAGAGGGAGCTAGAAGCTAATGATATCTACTCACAGCTAAAACGCGCAAAGATCTAA

>BrRR6(Bra019524)-DNA

ATGGCGGAGGTTATGCGTCCGGAAAAGTTGGACATGTCCAACGACACTTCTTCCTTAGGATCACCTGAGCTTCTTCATGTTCTGGCCGCAGACGATAGCATCGTTGATCGAAAATTCATAGAGCGGTTGCTCAGAGTCTCGTCTTGTAAAGGTAAACTTGAAGAAGTTTTGGTTCATCGTTATAGTTTTCGAACTTTCGTAATCTGACATTTTTTTTACATTGTGAAATTTCTCAGTTACTGTTGTCGATAGCGCGACAAGAGCTTTGCAATACCTTGGACTAGATGGAAACCATAGTTTTGTTGGATTTTTGAGGTAATATAATTCTTAATATATAATGATCCTTAGTGTTGAGAATCAATTTTTTTTTTGTCAACTGTTGCGAATCCATTCAATGTTTGGTATTTAAGACTTTTTATTTGAACTAATATTTTCAGGATCTGAAGATTAATCTGATAATGACGGATTACTCTATGCCTGGTATGACTGGATATGAACTATTGAAGAAGATTAAAGTAAGTTTTCCCATCCCTCTTTTTTTTCTGATCGATTTGAAAAATAAAATGAAAGTTTGATCTAATATGTGTTTATGGATATTGAATATTTTTATAGGAATCATCAGCTTTCAGAGAAATACCCGTTGTGATTATGTCGTCAGAGAAATCTTGCCTCGTATTGATAGGTATGTACTGTTTTCCTGATCACGATCACGTAAGATTATGCTTTACAACTTATAGTAATTTTCTTAGATTTTTTATTTGCATTGATTGCTTCAGGAGATTTGGTGATTAAAAGGGTTTTGTTTATGGAAACTTTTAAGTTTTTTTTTTTGACAACAAGTTAATCACACTATTTAACCGATATGTATTTTGACCAGATTAAAATTGTATAGATCTTATCATTTTTGTATGATATAAGATATGATATGATTCTATTTTTCAAAAAAACATATGATATGATTCTATGAAGCATCATGGGTAACTGTGGTGGTGGTTGTATATTTGATGAAGCTAAGATTTGGTGTAACTTTTGGCAGTCCGGTGGCGACAAAATAAGAAAAGAATAATCCATCATAATAAGTTAAGCAAAATCTTCTCATGAGATTTGATTCTATTCCCGGTGATGTGTTATAACCCCCAAAATTATAATATCCCTTATATATTAAAAGAGAAGCATTGTAATAAATGCATTCACATTATAATAAACACGTGGCAGCTTCACAATGATTTGATAATAAATATGCTAACGCGTTCACACTATAATCATAAATGTGTTCACACTATGTACTTTGTGATTTTTTTTAATATAAAACTCACATACATAGTTCCAATAAAACTCTGGATTTTTCGGTTCGAATAAAAATAGATAACGAATCAAAAGCTAAACTATATATATATATTATTTTTATTGTTTACGGATAAAATTGAGCAAAATATTCATAAATTTTGATTCGATTTGTTATCCGTTTTGATTTGAACCAAAAAATCTTGATATTTGAAACTTTACGAAACAAATCAAATACTAAAATACAATATCCAAAAAAGAAGCAAATCACTAATACCAATATTTTTAGGAACATATATCTAATTCGATATGTTATATGCATATATACATATATGAAAAGAATTATATATATTATACTTTATATCAGTTTTACAATATAAATTTATTATATTAGGTACTAGAATTAAAAGGTTATATAATGTTTTATTTTTGTAATAAAATGTTATTATTAAATTATTTTTAAAATTTTATTTTATTTACGAATCAAATCGGATATTCTTTAAAATTCTAAAACATTTCGGATATCGGAGTCACCGAATATCTAGGTGGCTAAAAATCGAATCGACAAAAATGCTTCCAAATATCCAGATACTTGATATGTGTCCACCCCTATTTACGAATGTAATTTTATTTTCTTTTGATGAAAAAATGACTAATGTCAAGGTCTTTTTTATTTTAAATAAATTTTAATTTTATCTTTCATGTATTATTCTGAACAAAAATGTCATTTAATATTAATTAACAATATCTTTATATATTTTTCAACTATTTTTATATACTTTTTACATAAATATACATGTGCACCTTGATGTGAGCATCTTATAACTAAGTATTCACCACAGCTGAAGTATCTAATTTTTTTGAAAGTTGAAATATTTTTTCTTAATGTTTCTTTCACTACCGACCAAATTGTAGTGAAATGATTTGTCTTAATAATTTTCTTTTCTTTTTCTTAAACTATTATCTGTTTAAAAACTATAATATGAAACTATTGGTTCGACATGACGACTATCTAAACTTCATAATATAAAAATAAACATATAATATTAATTTTAGGTTTTTATCCGAAAAAACCTAAAAATCAAATGTTTTAACCGAATAAACTAAAATGAATATTAATTTAAAATAATAGTTATATTTTAGAAGATTAAAAACAAAAAAACGTAAAAACTAACTAATATCTAGATTAAACAGATTTATTGTCTTTTTTATTAAAAATAACGAAACTAATAATCACATCCCGCGCAAGGCGCGGGTTATTACCTAGTATGCTTTTAATTCGTTTGCATAATATTGGGTAAGGTCGTTATAAATCACATTCTAGTGGCAGACACCTCACATGGCTTTTGCTTATTACTAAGGAACCATTTTTACCAAAAAATTAAGTCGAATAATGTAATTGCTTATTAATTATGAGCTGGCCATTTTCTGGATCCAAAGCCAACTTTATGGAATTAGACCACTGATATTTGATTCTCTTTGTGGATTATTTTTTATTCAGATGTCTTGAAGAAGGTGCTGAAGATTTCTTATTGAAGCCTGTGAAACTGGCTGATGTGAAGAGATTAAGAGATTCGTTACTGAAAGCTGACGAAATAGCTTTCAAGAATATTATGCACAAAAGAGAGCTACAAGCTAATGATATCTACTCGCAGCTAAAACGCGCAAAGATCTGA

>BrRR6(Bra019524)-CDS

ATGGCGGAGGTTATGCGTCCGGAAAAGTTGGACATGTCCAACGACACTTCTTCCTTAGGATCACCTGAGCTTCTTCATGTTCTGGCCGCAGACGATAGCATCGTTGATCGAAAATTCATAGAGCGGTTGCTCAGAGTCTCGTCTTGTAAAGTTACTGTTGTCGATAGCGCGACAAGAGCTTTGCAATACCTTGGACTAGATGGAAACCATAGTTTTGTTGGATTTTTGAGATGTCTTGAAGAAGGTGCTGAAGATTTCTTATTGAAGCCTGTGAAACTGGCTGATGTGAAGAGATTAAGAGATTCGTTACTGAAAGCTGACGAAATAGCTTTCAAGAATATTATGCACAAAAGAGAGCTACAAGCTAATGATATCTACTCGCAGCTAAAACGCGCAAAGATCTGA

>BrRR7(Bra018084)-DNA

ATGCGTTCTGAGACGTTAAACATATCCAACAACACTTCTTCCTTAGCATCGCCGGAGCTTCTTCATGTTCTCGCCGTTGACGATAGCATCGTCGATCGGAAGTTCATCGAACGGCTACTCAGAGTCTCTTCGTGTAAAGGTAAACGACATGTTTCGGTTCAACGTTTTGGTTTTGTGGAATCTGTTTGACGTTCAAAATCTGACAGTTTTGATTTTATTATATATTCAGTTACTGTTGTCGATAGCGCGACAAGAGCTTTGCAATACCTTGGATTAGATGGAGACAACAACTCGGTTGGATTTGAGGTAATAGATCTGATGATAGCAGTTTTTTTTTTTTCTTCTTAAAATCCTTTCCGTATTGAAGATTTTCGAAATTTTCAGGATCTCAAGATTAATCTGATAATGACGGATTACTCTATGCCTGGGATGACTGGATATGAACTACTCAAGAAGATCAAAGTATGTTTATCTAATTTGTTTCATTCAATCGATCTCATATCAGCAAATACAATGAAAGTTTTGTACTAATTGGTTTTATGAAATTTGAATTTTTTATAGGAATCATCAGCTTTCAGAGAAATTCCAGTTGTCATTATGTCCTCAGAGAACATCTTGCCTCGTATTGATAGGTATGTTCTGTTTTCCTGTTATCTTAATCACGATTCTTAATCTTTGTTTAAGTTCATAATCATGTAAGATTATGAATTACCAGTAATAGTAATTCTATTAGATTTTGTGCATTTATTACTTCAGGAGATTCGGATATTAAAAGGGTTTTGTTTAGGGTAACTTTTAAGTAATCAAACTACCGTACTTTTTTTTTGTAAATACAGTTAGCCGATATGTATTGACCAAATTACATTGTTTACAATCGTATGAGACTTGCATGATAAAAGTATTATTACTTTTACTATCATGGGCAACTGTGGTGGTGATCGAGCTAAGATTTGGTGTTACTTTCGTGGGTCCTTGACCAAAATTTTCTAGAGGATATTAGGAATATATAATAAGCCATAAATTGTTTCTTGTGTCCACAAAATAAGTGAAGATAATCCATCATAAGTTAAGCAATATCTTCTCTTGAGATTTGATTCTGTTGCCGGGGATATGTGTTAATAACCCCAAAACTGTATAATACTATGCTTTTGATATGTTTTTTTCGTAATCTTTGGTAGGGTCATTTATAAATCTCATCCTCGTGTTAGACACCCCACATGCTTTTTGCTTATTATTAAAGAACCATCATTTACCAAAAAATAAGTCTAGTAATGTGATTGCCTATTATTATTATGAGCTGGCCATTTTGTGGATCCAAAGTTATCATCTTTGTGGAATAAGATCACTGATATTTGATTCTCTATTTGTGGATTCAGATGTCTTGAAGAAGGTGCTGAAGATTTCTTACTGAAGCCTGTGAAATTGGCTGATGTGAAAAGATTAAGAGATTCTTTGCTGAAAGCTGAGGAAAGAGTTTTCAAGAACATTATGCACAAAAGAGAGCTAGAAGCTAATGATATCTTCTCACAGCTAAAACGCGCAAAGATCTGA

>BrRR7(Bra018084)-CDS

ATGCGTTCTGAGACGTTAAACATATCCAACAACACTTCTTCCTTAGCATCGCCGGAGCTTCTTCATGTTCTCGCCGTTGACGATAGCATCGTCGATCGGAAGTTCATCGAACGGCTACTCAGAGTCTCTTCGTGTAAAGTTACTGTTGTCGATAGCGCGACAAGAGCTTTGCAATACCTTGGATTAGATGGAGACAACAACTCGGTTGGATTTGAGGATCTCAAGATTAATCTGATAATGACGGATTACTCTATGCCTGGGATGACTGGATATGAACTACTCAAGAAGATCAAAGAATCATCAGCTTTCAGAGAAATTCCAGTTGTCATTATGTCCTCAGAGAACATCTTGCCTCGTATTGATAGATGTCTTGAAGAAGGTGCTGAAGATTTCTTACTGAAGCCTGTGAAATTGGCTGATGTGAAAAGATTAAGAGATTCTTTGCTGAAAGCTGAGGAAAGAGTTTTCAAGAACATTATGCACAAAAGAGAGCTAGAAGCTAATGATATCTTCTCACAGCTAAAACGCGCAAAGATCTGA

>BrRR8(Bra010132)-DNA

ATGGCTGAGGTTATGCTACCGATGAAAATGGAGATGGCTAACGATCCTTCCAAGTTTACATCACCTGATCTTCTTCATGTTCTCGCCGTCGACGACAGTCACGTTGATCGGAAATTCATAGAACGCTTGCTCAAAGTCTCTTCCTGTAAAGGTAATTAATCATTTTTTGAAAAACAATATCTCGTTATTATTTTCAATCTTGAATTCTTATGGACATGTTGTTTGTGTTTTGGTTCAGTTACTGTTGTTGATAGTGCGACAAGAGCTCTGCAATACCTTGGACTAGACGTAAACGAGAAACCCATCGGTTGTAAGGTAAAGAATCAGTTTCCAAATCCACCCGATATACTTTTTTGTTCTATTTCTTGAGAACGTTTTGATATGAAATTAAGCTCTCACAGCAAATTGTTAACGGTGATTTGTTATGTTTTTTGGTCGATTTTTCAGGATTTGAAAGTTAATTTGATCATGACGGATTACTCTATGCCCGGAATGACTGGATATGAACTCTTGAAGAAGATCAAAGTAAGCAAACCCTCTTTAAGGAATTACTATACAATCTGATGATTTTGTTGAATTCAGTTAGTTAAATCTAAATATTTATGCTAATCTAATCTGTATTTTTTTCACAGGAATCGTCAGCTTTCAGAGATGTGCCTGTGGTGGTTATGTCATCTGAGAACATTTTGCCTCGTATTGATAGGTATATTTTCATGCTGTTCCTTGCATGTAGATCATCTAGTCAAAAAGTTATAGTAAAGTTGAGATGTCTTGAATGATGTTTATGGTACATTTAATTTTAACCCCAATATATTAGATAGATCATCATGTTATTTGATTAGTTTTTAAACCAGCCATCAGTGGTGGTGATTGCATAATTATTCAAATGTGAGATTTGGTGATTTGTCATGGGTCCCAAGATGATTTTTATGTTTCTAAATCTTTTACTTTCGAGATTTGATTCTGTTTCTGGTGATTGTGATATTATACAATAAAAATAGTTGGAAATTGTTTGCCTAATCTTTGGTAGGTATTGTGAAATCACATGCTTTTCTCTGGACCAAATTAAATAAAAAGAATAATATTTTTTCTCTATAGAAATTATAAAATGGCCAACTCAATATATGATTTGGCCATGTTATAGTGTTTGCTACATACACAACAAAACAGACCTGCACAGAAATTTATTGATCCAGAAAATTAATAAACTTTATTGAAATTGTACTAAATTCTATTTTTGTTTATATATGCAGATGTCTTGAAGAAGGGGCTGAAGATTTCTTACTGAAGCCGGTAAAACTCTCGGATGTAAGAAGAATAAGAGATTCTCTGATTAAAGTTGAAGATTTATCTTTCACAAAGAGTATTAACAAGAGAGAGCTAGAAACAGAGAATGTCTACTCCTTGGACTCATCTGTTCCCTTGCAGCTCAAACGCACAAAGATCTGA

>BrRR8(Bra010132)-CDS

ATGGCTGAGGTTATGCTACCGATGAAAATGGAGATGGCTAACGATCCTTCCAAGTTTACATCACCTGATCTTCTTCATGTTCTCGCCGTCGACGACAGTCACGTTGATCGGAAATTCATAGAACGCTTGCTCAAAGTCTCTTCCTGTAAAGTTACTGTTGTTGATAGTGCGACAAGAGCTCTGCAATACCTTGGACTAGACGTAAACGAGAAACCCATCGGTTGTAAGGATTTGAAAGTTAATTTGATCATGACGGATTACTCTATGCCCGGAATGACTGGATATGAACTCTTGAAGAAGATCAAAGAATCGTCAGCTTTCAGAGATGTGCCTGTGGTGGTTATGTCATCTGAGAACATTTTGCCTCGTATTGATAGATGTCTTGAAGAAGGGGCTGAAGATTTCTTACTGAAGCCGGTAAAACTCTCGGATGTAAGAAGAATAAGAGATTCTCTGATTAAAGTTGAAGATTTATCTTTCACAAAGAGTATTAACAAGAGAGAGCTAGAAACAGAGAATGTCTACTCCTTGGACTCATCTGTTCCCTTGCAGCTCAAACGCACAAAGATCTGA

>BrRR9(Bra025708)-DNA

ATGGCAGTTGGTGAGGTCATGAGGATGGAGATCCCAGCCGGTGGAGATATGTCAGTTACTTCACCAGAGTTACATGTTCTTGCCGTCGATGATAGTATTGTGGATCGTAAAGTCATTGAGAGATTGCTGAGAATCTCAGCCTGTAAAGGTATATACCAATATGAGTCTCACTCTCTCTATGTCTTCCCTTCTCTGATGTGTGGTGTCGTTCTCTTGCCTAAATAAAAACCTTATTTGTTATTTCAGTGACTACTGTAGAGAGTGGGGCTAGGGCTTTGCAGTACCTTGGCTTAGATGGAGATAAAGGAGCTTCTGGTCTTAAGGTAACTCTCTTTACAAATCTGTTTGGTTACTGATAGATCCTCTAACTATGATAGACCTTTGCAACTTTTGCTCTTTTCTTCTTGTCAGGAAAATAATAATAACAAGACCTTATTTATATTTTTACAATGAAAAATACAAGACCTTATTTATTAGTAATTGTTTTGGTAGTAATTAAGATCTGATTTTTGTTGAAGTGTTTCATAAACAATTCTGTGTGACATTTCTTATAACAGGATTTGAAGGTGAATTTGATAGTGACGGATTACTCAATGCCAGGACTAACGGGATATGATCTTCTCAAGAAGATTAAGGTAATAATAATAATATGTTTTGATTTTATCAATCTGAGAAAACTCTTTAAATAAATCTTATCTAATGCTCATACAAATACCATTGATATGTGTAGGAATCTTCTGTCTTCAGAGAAATACCAGTTGTGATTATGTCATCTGAGAACATCTTACCTCGTATAGAACAGTAAGTGTTTCTGTAAATATGTAATAATTACAAAGTAGATCATTTTTTTGTCTCACTGTGATCTAACCTATGTGGCTTATTTTGTTTTTCGTTTCAGATGTCTGAGAGAAGGAGCAGAGGATTTTCTGTTAAAACCGGTGAAACTAGCTGATGTAAAGCGAATAAAAGAACTTATAATGAGAAATGAAGCAGAGGATTGCAAAACCTTAAGCCATTCTAACAAGAGAAAATTTGCAGAATACATCGATGATGCATCATCACCATCACCATCATCATCATCAACTCATGATGAATCTGCTGCCAAGGACTTTCCATCTTCAAAACGGATGAAATCAGAAGATGACAAGTTTTCTTCACTCCTTTGA

>BrRR9(Bra025708)-CDS

ATGGCAGTTGGTGAGGTCATGAGGATGGAGATCCCAGCCGGTGGAGATATGTCAGTTACTTCACCAGAGTTACATGTTCTTGCCGTCGATGATAGTATTGTGGATCGTAAAGTCATTGAGAGATTGCTGAGAATCTCAGCCTGTAAAGTGACTACTGTAGAGAGTGGGGCTAGGGCTTTGCAGTACCTTGGCTTAGATGGAGATAAAGGAGCTTCTGGTCTTAAGGATTTGAAGGTGAATTTGATAGTGACGGATTACTCAATGCCAGGACTAACGGGATATGATCTTCTCAAGAAGATTAAGGAATCTTCTGTCTTCAGAGAAATACCAGTTGTGATTATGTCATCTGAGAACATCTTACCTCGTATAGAACAATGTCTGAGAGAAGGAGCAGAGGATTTTCTGTTAAAACCGGTGAAACTAGCTGATGTAAAGCGAATAAAAGAACTTATAATGAGAAATGAAGCAGAGGATTGCAAAACCTTAAGCCATTCTAACAAGAGAAAATTTGCAGAATACATCGATGATGCATCATCACCATCACCATCATCATCATCAACTCATGATGAATCTGCTGCCAAGGACTTTCCATCTTCAAAACGGATGAAATCAGAAGATGACAAGTTTTCTTCACTCCTTTGA

>BrRR10(Bra016526)-DNA

ATGGCTGTTGGTGAGGTCATGAGGATGGAGGTTCCCACCGGTGGAGATCTGACTGTTAGTTCGCCTGACCTACATGTTCTAGCTGTCGACGATAGTATTGTGGATCGTAAGGTCATCGAGAGGTTGCTAAGAATCTCTTCTTGCAAAGGTAAACTATACACGGCACCACAACAATTTTCTGTCTCTCTGTTTTTATCTCTTTGCCTAATAGTTTCTGTTTTTTATTTCAGTGACGACTGTAGAGAGTGGGACTAGGGCTTTGCAGTATCTTGGCTTAGATGGAAACACAGGAGATTCTGATCTTAAGGTACAAATCTGTTTGGTTACTGAGAAATCCTCTTAAGGAAAGAAAGAGAGACCCTATGCAACTTTTGCTCTTGCTTCTTGTCTGAAAAAATAAATAAAATAAAAAGACTATTTAGTAATTAGTTTGTTAGGTCTGATATTTTGTTGTTGTTGAAGTTATTTATTAATAGTTATGTGTGATCTTTCTTATTACAGGATTTGAAGGTGAATTTGATAGTGACTGATTACTCAATGCCAGGACTAACAGGATATGATCTTCTCAAGAAGATTAAGGTAATAATTAATTAATTAGTAATAGATTTTGATTTTTCACGTATGAGAAAAAAAACTTGAATAAATGTTCTCCAATGTTGATACAAGTATATATTGATAATGTGCAGGAATCTTCTACATTCAGAGAAATACCTGTAGTGATCATGTCATCTGAGAACATCTTAACTCGTATAGAACAGTAAGTGATTCTGTAAATGTGTAACAATACAAAGTAGATCATATTTTATCTACCCTGTGATATAAACTAAGTGACTTATCTTCTCTATTTTTTCAGATGTTTGAAGGAAGGAGCAGAGGATTTCCTTTTAAAACCGGTGAAGCTTGCAGATGTAAAGCGAATAAAACAACTTATAATGAGAAATGAAGCAGAAGACCACAGAACCTTGAGCCATTCTAACAAGAGAAAGCTTGGAGAAGATGTTGATACATCACCATCATCAAGTCATGATTATTCCTCTGTCAAGGACTTTCCATCTTCAAAACGAATGAAGTCAGAATCTGACATTTTTTCTCCTTTTATTTGA

>BrRR10(Bra016526)-CDS

ATGGCTGTTGGTGAGGTCATGAGGATGGAGGTTCCCACCGGTGGAGATCTGACTGTTAGTTCGCCTGACCTACATGTTCTAGCTGTCGACGATAGTATTGTGGATCGTAAGGTCATCGAGAGGTTGCTAAGAATCTCTTCTTGCAAAGTGACGACTGTAGAGAGTGGGACTAGGGCTTTGCAGTATCTTGGCTTAGATGGAAACACAGGAGATTCTGATCTTAAGGATTTGAAGGTGAATTTGATAGTGACTGATTACTCAATGCCAGGACTAACAGGATATGATCTTCTCAAGAAGATTAAGGAATCTTCTACATTCAGAGAAATACCTGTAGTGATCATGTCATCTGAGAACATCTTAACTCGTATAGAACAATGTTTGAAGGAAGGAGCAGAGGATTTCCTTTTAAAACCGGTGAAGCTTGCAGATGTAAAGCGAATAAAACAACTTATAATGAGAAATGAAGCAGAAGACCACAGAACCTTGAGCCATTCTAACAAGAGAAAGCTTGGAGAAGATGTTGATACATCACCATCATCAAGTCATGATTATTCCTCTGTCAAGGACTTTCCATCTTCAAAACGAATGAAGTCAGAATCTGACATTTTTTCTCCTTTTATTTGA

>BrRR11(Bra000224)-DNA

ATGGGTGTGGTAACAGAGTCACAGTTCCATGTTTTGGCGGTTGATGATAGTCTCTTTGATCGGAAAATGATAGAGAGATTGCTGCAAAAGTCTTCCTGTCAAGGTAAACAGAACAAAAAGAGGCTAAAATCTTTCATATAAAGAAAAGAGAGAGACAGAAGATTCTTAAAAGCTTTATGTTGTTCGTATGTTTGCAGTAACTACAGTTGATTCAGGCTCTAAAGCTCTCGAGCTTCTTGGTTTGAGAGAAAGCAATGAGAGTGACGACCCAAATGCTACTTCTACATCACCTGTAACTCATCAGTTCAACTGAGTTTATTTCGCGTTTAGGTTTCGTTTTATTGAAGTTTGTTTGTAACTTTGCGCAGGAAGTTGAAATAAATCTTATAATTACAGATTACTGTATGCCTGGCATGACTGGTTATGATTTGCTCAAGAGAGTTAAGGTACTTCTTGTTCTTGTTTTTTTTTTCTTGGACTCAATTGGGTTGCTGTCTTTTCCATCTCTGGAAAATTCGTTTTCCAGAAAAAGAAATAAAGATGTTCTCTTTACTAGTATTAATTTAGAATGTCAATGGTGAAAATATCTTTGGAATATTATTCCCACTCTCTTAAACTTTAGTTATTTATATGATATATTGTATAATACGAGAACAATAATGATTTTCTGAGAGACATCTACTGTATATGCTTTGTGTAGGAATCAGCTGCATTTAGAAGCATTCCCGTTGTAATAATGTCATCTGAGAACGTTCCTGCTAGAATCTCCAGGTAAGCCTCTGTTTTCTTTGACTCTTCTTGATTTGTGAAATGGGGTTAGTATAATTACTTGAACAACAAGTCTTTAAATTTTTAACCTAGAGGAACAACAATGTAGATTTCTTGCAATGTATCTGGTATTTTGGTACTGATATAATGTCAATGGACAACAGATGTCTGGAAGAAGGAGCTGAGGAGTTTTTCTTGAAACCAGTAAAGTTAGCTGATCTCACCAAGTTGAAACCTCATATGATGAAAACCAAGTTGAAGAAAGAAAGTGAAAAACCAGCAGAAGAAGTGAAACCGGAGATAGAAGAAGAAGAATCGCCAGTGATTGAAATCTTGACTCTCCATCAAGAACTTGAATCCGAGCAACAAGAACCAATGTTGAGTAATAATAAGAGGAAAGCAATGGAAGAAGCGATATCTACTGGTCGATCACGTCCTAAATACAACGATATCACAACATCAGTCTGA

>BrRR11(Bra000224)-CDS

ATGGGTGTGGTAACAGAGTCACAGTTCCATGTTTTGGCGGTTGATGATAGTCTCTTTGATCGGAAAATGATAGAGAGATTGCTGCAAAAGTCTTCCTGTCAAGTAACTACAGTTGATTCAGGCTCTAAAGCTCTCGAGCTTCTTGGTTTGAGAGAAAGCAATGAGAGTGACGACCCAAATGCTACTTCTACATCACCTGAAGTTGAAATAAATCTTATAATTACAGATTACTGTATGCCTGGCATGACTGGTTATGATTTGCTCAAGAGAGTTAAGGAATCAGCTGCATTTAGAAGCATTCCCGTTGTAATAATGTCATCTGAGAACGTTCCTGCTAGAATCTCCAGATGTCTGGAAGAAGGAGCTGAGGAGTTTTTCTTGAAACCAGTAAAGTTAGCTGATCTCACCAAGTTGAAACCTCATATGATGAAAACCAAGTTGAAGAAAGAAAGTGAAAAACCAGCAGAAGAAGTGAAACCGGAGATAGAAGAAGAAGAATCGCCAGTGATTGAAATCTTGACTCTCCATCAAGAACTTGAATCCGAGCAACAAGAACCAATGTTGAGTAATAATAAGAGGAAAGCAATGGAAGAAGCGATATCTACTGGTCGATCACGTCCTAAATACAACGATATCACAACATCAGTCTGA

>BrRR12(Bra016943)-DNA

ATGGGTATGGCAACAGAGTCACAGTTCCATGTTTTGGCTGTTGATGATAGTCTAGTTGATCGGAAAATGATAGAGAGATTGCTGCAAAAGTCTTCATGTCAAGGTAAACAGCAGAAAATGTGTAACGACCCGGTTCTCTAAATGTTGGTTTTGTATTGATTTTAATTGAATCAGACCATTGGTAAACCAAAGCATTATATATATCATATTTCTTAACCTAGCAATCAAAAGAAAATGGAAAGGAGAGTGCATCGAGAACCGCCTCCATTAATCTCCATCACGTCTTCTCCCTCTTCATCACGTCTTCAAGTACACACTCCTATTGTCCTTTCCTTCACGCCAGAGAAAGGAGTTCCTTGCGCCGTCATCCTAGATCGATTCAGGAGACTTTAGCCTTTGAACCAAGGCTCTTTGGATAGATCTCATTTCCAGGAATCTGTAGCAGCAAACGGAATCGAGATCGGAGTTGTACAACAAAAGTTATTGACGTTGCTTCCTGGCCAAGAATAAACTTAAGCTGTTGTGGTGCAGAGACAGTTCCGACCAGTCCCAGCGTAAAGGCGAAACGGTCGACCAAAACAGAAGCTTTCAGTATGGAAATTTGTCAGTAAGTAGTTCACTCTTTTATCTATCATCTCCAAGAAGCGGATTGTCATTCCGAGTTATTTTCTGAAAGTTGTGTTTGTTCTCGCGAGAGGTATTGCTGCAGTCTTCGTATGCGACCAGCCTGCAGGTGAGGCCATTTTGGTTGATCAAATGGTGGCCAATCGGAGTAAACCCGGTGGCTACGGACAAAGGACATATAGGACTACATTCTTACCGGAGGGATTGAAAGTTTAACGGTCAGTTCTTGAGTTATTCGTCTGTTTTCTCGAGGGTTCAGGTCTACCCAAATCCTCACATTCGTCTATTTTCAGAAAGCTCCGGAGTTGTTATTTTAGAGCCAGCAAAATTCATTCGGTGTGTTCAGGTTTTCTGAAGAACCTGTGGCAAAGGTGAGGGTCTATTCCCGAACTTCTCATACACGGCTTAGGACCTTGAATAAGTATAATTTATTTTTAATATGTTTCGTCCGCGTGAAATCAAATTTGTCATTGCTTGTTTAGTTTTAGGTTCTTTTCTTAAACCGGATATAAGAACCTAGATGGTTCAACTTTGACTATTTCATTTAAAATTAGAGAATAAAAATAGTTGTTTTATTAAGAGTTATGAGATTGTTTGGAGACAGATACAGAGACGGCTACAGAGACGTGCTGCTGTATGCTGGATTGTATGGACGTTACGGCCAGCTATAATCGACCGGTTGAGCTATAGCCTGGAGGTGTCGATAAATCGTCTATGGGCGTGTGTGACCGAGCACATATTCGATGGTTTTTTGGCCTGTTAGTTGGCAGAGAGTGTGCACCTTGCTAGGACTATTATTTGATCTCTTGGCACTTTAGGTACCGTGTTTGCAACATGTTAGGATTAAATTAAATTTATTCGTAGTGCTTTATCTGAGTCCATGGACATCGCGTTTAGTATCACATACCTCACTGAGTGACTTCCCTGTTACTCACCCTTCTTTCCTACGTTTTCTTTTTAAAGTATAAAATACTTTATATATATATATATATAATAAAAAAAATGTTTGCGAAGTTCTTTAGCATCATTCTGTCCAGTAATTATTAACTCAATTGTCTCTTTATATGACGATGAGTAAAATCATCGTCATTCATTCTTCGACTAACAAGATTCTAGCGAGATGTTCAGTAATAGATGTGTGATGTCTCAGTTCGTCAGTTTTCTCAGGAGTTATCAGTTTCTTTGGTCATGATTCTGAGATTATCCAGGAGTGGATATGTGACGTTTCCGCTTCCACCCTATTTCTTTCAAGCAATCGTTCCACGAGTTTTTGTTACTGGAGATTATGTGGTGTGACGTATGAGCCATCGATTGGCATGTGTTGTTTTTGTGTACGAGTATGTTGACTATTTTGGAGGTATGTTCAGTTTGTAAACTCGTGGGGATCGCTTTTGGATTGGGAGTGTGGCAACTTGTGTTGTATTGATATTGGAGTTACGCTTGGTTTTGTTGTTAGTTTTTAGCATTGTTGATCGTTTGAGAGATGTGTCAGTCAAACAACTGGCATGGGACGTGGGAGGTATTTATATTTATCGCAGTGTACCAGCTATGCTCGGAGGACTGATTTGTTTGGGGACTCGTCAGGGATGGAGCTACGCTTTTACGATGTCATCCTTGGGATGGATTGGATGTCACCACATATTATAGTGTTAGATTTCCTAAGGGCAAGAGTTGATATTGCTGGAGAAAATGAGAGTTTTTATTGCATGCATGCTACATGCTGAGGAGTTATTGGATAGAGAAGCAGAGGGATTTTTGGCGATCATTTCAATGGTTAAGAATGTTGGACAGTATGAGTTGTAGGATCTTTTGGTTATTGCAGATTATGAGGATGTAATAGTGGTCTTTGGAACGGACACCACCAGACACAAGAGACGTTCTTACGATTGATTTGGAAACAAGGACATCACTGGTTTTACGAGTTCTATACCAATTAGCGGTAGCAGAGATGACCGAACTGAAAGAGCATATGGAAAATTCATCAGACATGGATTTTATCATACCGAGTACTTCACCGTGGGGAGCAACATTATTGTTTGTGAAGAAGAAAAATAAGGGTTTCAGAATTTGTCTCGACTACAGTGGTTTGAACAAAGTGACCATTAAGGATAAGTATCGTTTCTTTGTATTGATGATTTGTTGTATTAGCTGCATGGAGGTTCATGGTTCTCGACGTTTGTCTTAGCATCGAGATACCATTAGATGACTACAGCTGAGGAGTATGTACAATAGATATTATGAATTGTAGTGATACCACCGTTAGTATTCTTGAAGATTATGTATGATGTCTTTCGTGAACACTTGGATAAGTGCGTGATTGTATTCATCGATGACACCCTAATTTATTCTCGGAGTAGAGAGGAGCATGCTGAACATTTATGGATTGTGTTAGGTAAGCCTGAGGAGAAGAAACTATTTGCTAGATGAGTGAGTGTAGTTTTAGCAAAGAAAAAAAAATGGATTTTTGAGTCATGTGGTTTCAGAAGCAAGAGTTGCTGTCGATTCATAAAAAAAAAAAAAAAAAAAAAAAAACCTACTACAATTTCATTGTGGCCGACACCAAAAAAAAAAAAGTACAGAGAGTTGAAGAATTATTATCACTGCCTGGTATGAAGAAGGATGTAGCTACATTGTGTCACCATATCAGACTTGTCAGATGGTCAAAATAAAATATCAGATATTATCGAATAATTGCACAATCTACTTCTTCCAGATTGGAAATGAGATATGTTGATTATGGGGTTTCTTATTGGACTATGGAAGTCAAGTTTCATGAAGATTAATGTATGAGCGAACATGAACCGTCTCTCTAAGTTAGACTCAAGAGGGCATCCTCAGGACTTGTGCTTAGCTCGCGGGAGAAAAAGAAATTGAGGAAGATTCAGCCACTGGAATAATTCATGTACAACAACAATTATCACTTGGGCATTCAGATATCACTTTAGGAGGCTCTTTACGGAAGATCATTCACACACATCCTAGACCAAAGTTGGGAAGCACTATGACGTATAGCAACCAATGGTTTAAAGGACATTGAAGCAAATATAAATGCTCAATAACCACCTAAAGGAAGTTGATGACTAAAAAAAAAAAAAAATGCAGTTAATACTGGAGAGACTTGGAAGTTCAGCTGGATAATTTAGTGTACCTAAAAATAAAGACATTTCAGAGAGGATTTAAGACTCGGGAGCTAAAGGAAGCTTAAACCTAAATATATGTGACCGTACCCCTTTGTGGAACAATGGTTTATAGATGGCAGTTATCAGCAAAGTTGTCAGAATGCCATGACGGGTTGGTATTGAGGAAGGTCGTGAGAAAGTCATAGTTTATTTTATAGCAGCCACGACGTGATCTCGGTAAAAACAAAAAAAAAAAAAAGAGAAAAAAAAAATTGTTTACACCTATTCAGTCAGTTGAGATTTTGGATTAACAAGAAAAATCGGTTTAGGAAATGATGACCATGTTCGTCAAGGTTTGTTATGAGAGAAAGAAACAAGATCTAGGAGGAGATCCGGGAGACTGAGACTTATATGATGATTACTTATCTACAACTTTCTCAATATGGTTTTGGACAACCAGCTCCGGCTTTGAATTCGAGGACGAATTCCTTATAAGTAAGGGAGAATTGTAACGACCCGGTTCTCTAAATGTTGGTTTTGTATTGATTTTAATTGAATCAGACCATTCGTAAGCCAAAGCATTATATATATCATATTTCTTAACCTAGCAATCAAAAGAAAATGGAAAGGAGAGTGCATCGAGAACCGCCTCCATTAATCTCCATCACGTCTTCTTCCTTGCCGCGCCAGAGCCTTTATCAACCTTTGTCGTGGGTGAGATCTTAACTGAGAGATGTGGAGGAGGCACGATCTAAACTGGATCATCACGTCTCTGTCACCAATCACATCGCTAGCCACCACTGGAGGTTTGTTCTTCACCGATCACCACTGTATCCATTGTAGCTGATGTCCATGGAGCCATCGTGGCTCTCGTTGGTGTTGTGTAGCGGGCTGAGCTGAGACATGAAGAAAAAACGTACGATCGGAGCTGCCGCGTCATTACACTTACGGTGCCGTTCTACACCGTAATCAATGGTGAGCCTCCGGAGACACAGCCCATGCCGTAACTAGTTCGAGATTCATTGTTGCAAGTCAAGCCGTGAAGGTTGTAGTGCCTTCTCCTTTGTTTCTTGTTTTACAAGGAACATTATAACTTAAAAACCTAAAGGTTTCATGCGTAGGTAGGAGTCATGGAACCTTGCCACCAAATGTAATGTGTACCATTTTTGGGTGTTTCTTGATCCATTTCCAATATTGGCTAAGGTGAGGGCCTTTTCTCGAACTTTTCGTACACGGCTTAGTACCTCGAATAAGTATAATTTATTTTTAATGTGTTCCGTATGCGTGAAATCGAGTCTTTCATTGCTTGTTTAGTTTTTAGGTTCTTTGCTTAAACCGGAACTAGGATTGTTAGATGGTTCAACACTGCTTGTTCATTGATAATTGATAATATATATGTATATAGTATATTGAGGTGATTTGGGTGTTAGCCGACCATGGAGACGTAATTCTGTGTGTCGGCTAGAGACCAGTACATTGGGTGTGGTGTATGCTGCGGCACAGCTTAGTCGACCTGTTGTGCCAAGGCTTCAGAGGTCGATAAATCGTCTACGGGCGTGTGTTACCGAGCACATATTCGGTGGTGTTTTTGGCCTGTTAGTGGGCAGCGAGTGTGCACCTCGCTAGGACCATTGTTTGATCTTTGGGTACTTTAGGTACCGTGTTTGCAACGTGTTAGGATTAAATTATATTTATTCGGAGTGCTCTGTCCGGGTCCATGGACTTCGGGTTTAGTATCCCCATACCTCACTGGGTAACTCACCTGTTACTCACCCCTCATTTGCCCCCATGCAGGTGAGACTGACGAGTATATGATATTTGGATGGATTGGTGCTACTGGGCTTTATTTCGGATTTTTATTTGGGCTTAGGGTGAGTGGAGTCATGTATATGGGTTATTATGCTATGAGATATTGTTGGTGGCACGCTGTTCTCCAGATAGGAGGTGACTGGTGTCACAAAATGGCTATAGGATCTCTCAGCTTTGTTATTGGAGAAAGATAGAGAGAGAGATTCTAAGAAAGTTTTATTTTGTTTGTATTTTGCAGTAACTACAGTTGATTCAGGGTCTAAGGCTCTTGAGTTTCTGGGTTTGAGAGAAAGCAATGAGAGCAACGACCCAAATGCACCCTCTTCATCACCTGTAACACATCAGGTTTATTTATTTATTTACTTATTCACTATTCAATTGAGTTTTTAATCTACACAATTCATAATATTGAAGTTTGTTGGTGGCGTTGTGCAGGAAATTGAAATAAATCTTATAATTACAGATTACTGCATGCCTGGCATGACTGGTTATGATTTGCTCAAGAGAGTTAAGGTATTATCTTCTTGCTTTTTTTTATGGTATTGTATAAAACAGAAAATTTTGATTTCCTGAGAAATTACATTAAGTTAATATGATTGTGTAGGAATCAGCAGCATTTAGAAGCATTCCCGTAGTGATAATGTCATCTGAGAACGTTCCTGCTAGAATCTCCAGGTAAGCATCTGTTTCTTTAAAATTAAAATTTTGAATCTTATTTGTGAGTTGGCAATTTGGCATTAGTGTTACTTAACTTTGGACCAATAGAACCATTAACAATGTATATAATGTCCTGAAAATGTAAACAACAGATGTCTGGAAGAAGGAGCTGAGGAGTTCTTCTTGAAGCCAGTAAAGTTGGCTGATCTTACCAAGTTGAAACCTCATATGATGAAAACCAAACTGAAGAAAGAAAGCGAGAACTCAGCAAAAGAAGATAATGCAGTTTTGAAACATGAGATAAGAAAAGAAGAAGAACCATCCGTGATTGAAGTCTTGCCTCTACATCAAGAAGTTGAACAAGAACCAATGTTGAGTAATAATAAGAGGAAAGCAATGGAAGAAGTGATATCTACTAATCGATCACGTCCTAAATACAATGATATCACAACCTTGGTTTGA

>BrRR12(Bra016943)-CDS

ATGGGTATGGCAACAGAGTCACAGTTCCATGTTTTGGCTGTTGATGATAGTCTAGTTGATCGGAAAATGATAGAGAGATTGCTGCAAAAGTCTTCATGTCAAGTAACTACAGTTGATTCAGGGTCTAAGGCTCTTGAGTTTCTGGGTTTGAGAGAAAGCAATGAGAGCAACGACCCAAATGCACCCTCTTCATCACCTGTAACACATCAGGAAATTGAAATAAATCTTATAATTACAGATTACTGCATGCCTGGCATGACTGGTTATGATTTGCTCAAGAGAGTTAAGGAATCAGCAGCATTTAGAAGCATTCCCGTAGTGATAATGTCATCTGAGAACGTTCCTGCTAGAATCTCCAGATGTCTGGAAGAAGGAGCTGAGGAGTTCTTCTTGAAGCCAGTAAAGTTGGCTGATCTTACCAAGTTGAAACCTCATATGATGAAAACCAAACTGAAGAAAGAAAGCGAGAACTCAGCAAAAGAAGATAATGCAGTTTTGAAACATGAGATAAGAAAAGAAGAAGAACCATCCGTGATTGAAGTCTTGCCTCTACATCAAGAAGTTGAACAAGAACCAATGTTGAGTAATAATAAGAGGAAAGCAATGGAAGAAGTGATATCTACTAATCGATCACGTCCTAAATACAATGATATCACAACCTTGGTTTGA

>BrRR13(Bra004615)-DNA

ATGGGTATGGTAAGAGAGTCACAGTTCCATGTTTTGGCTGTTGATGATAGTCAATTGGATCGGGAAATGATAGAGAGATTGCTGCAAAAGTCTTCATGTCAAGGTAAAACAGAACAAAAACCAAAATCTTTCAGTTATGTTCTTGGAGAAACATAGACAAGATTCTAAAAAACGCTTTTTTTTTTGTCGTATGTTTGCAGTAACTACAGTTGATTCCGGCTCCAAAGCTCTAGAGTTTCTAGGTTTGAGAGAAAGTAACGACCCAAATTCACTCGAAACTCATCAGGTTCTGTTCTTTTTCATCGTTCATTTGGTTTTGTTCAAAATTTACAATCATCTTCTTGTTTTTTTGAATGTTTTGTTGGTTTTTGGGTTTAAGCAGGAAGTTGAAATAAATCTCATAATCACAGACTATTGTATGCCTGGCATGACTGGTTATGATCTGCTCAAGAAAGTTAAAGAATCAGCAGCGTTTAGAAGCATTCCTGTAGTAATAATGTCATCAGAGAACGTTCCTGCTAGAATCTCCAGGTAAAACCTCTGTTTCTTTTGATTTGTGAATAGGCATTAGTATTACTTGCGAGACAAGACTTCAACTTTAGACCTAAAAAAAGAAACATTAGCTGTGTAGTTTTCTTGCAATGGATCTGGTTTAAATGTCCTGAAAATGTGAACTACAGATGTCTGGAAGAAGGAGCTGAAGAGTTTTTCTTGAAACCAGTAAAGTTGGCTGATGTTACCAAGTTGAAACCTCATATGATGAAAACCAAGTTGAAGAAAGAAGGTGAGAAGGTAGCTGAAGAAGAGAATGCAACTTCGAAACCAGAAGAATCATTGGTGGTTGAAATGATCTTGCCTCTGAATCAAGAACTTGAGTTGGAACAACAAGAACCAATGTTGAGTAGTAATAAGAGGAAAGCAATGGAAGAAGTGATATCTGCTGACCGATCACGTCCTAAATACAATGACATTACAACCTCGGTCTGA

>BrRR13(Bra004615)-CDS

ATGGGTATGGTAAGAGAGTCACAGTTCCATGTTTTGGCTGTTGATGATAGTCAATTGGATCGGGAAATGATAGAGAGATTGCTGCAAAAGTCTTCATGTCAAGTAACTACAGTTGATTCCGGCTCCAAAGCTCTAGAGTTTCTAGGTTTGAGAGAAAGTAACGACCCAAATTCACTCGAAACTCATCAGGAAGTTGAAATAAATCTCATAATCACAGACTATTGTATGCCTGGCATGACTGGTTATGATCTGCTCAAGAAAGTTAAAGAATCAGCAGCGTTTAGAAGCATTCCTGTAGTAATAATGTCATCAGAGAACGTTCCTGCTAGAATCTCCAGATGTCTGGAAGAAGGAGCTGAAGAGTTTTTCTTGAAACCAGTAAAGTTGGCTGATGTTACCAAGTTGAAACCTCATATGATGAAAACCAAGTTGAAGAAAGAAGGTGAGAAGGTAGCTGAAGAAGAGAATGCAACTTCGAAACCAGAAGAATCATTGGTGGTTGAAATGATCTTGCCTCTGAATCAAGAACTTGAGTTGGAACAACAAGAACCAATGTTGAGTAGTAATAAGAGGAAAGCAATGGAAGAAGTGATATCTGCTGACCGATCACGTCCTAAATACAATGACATTACAACCTCGGTCTGA

>BrRR14(Bra014649)-DNA

ATGGGCATGGCAGCAGTGGAATCGCAGTTTCATGTTTTAGCCGTTGATGATAGTTCATTCGATAGGAAACTCATAGAGAAACTGCTTCAAAAGTCTTCGTGTCAAGGTGAAAACGTGTTTCAAAGAACATGAATAATAAGTCTTTGTAGCTTGTAAGAACTGAAGAAAGATCTCACCTTTGTTAATGTTTTTGTTGGTGTTTGCAGTAACAACTGTTGATTCAGGCTATAAGGCTTTAGAGTTTCTTGGTATTGAGAGTAACGACCCAAATGCTCTTTCTACATCTCCTCAGGTTCGAAAAGTTTGCTCCTTTGTACTTATTTAAATCACTTCAGTGGTTAACAAGTCTTTGTCTTAATAGAAGTTTGTTCATTCTGCTTGATTTTGGCTTTTGACAGGAGGTTGAAGTGAATCTTATCATTACAGACTATTGTATGCCAGGCATGACTGGTTATGATTTGCTCAAGAAAGTTAAGGTACTTGTTTGTAATATCTTTAACTTGTGATTTGTTTATGTATTGATAACATCTTTTGATTTATTCTGGAATTTAGTAAACTTGTTTTCAAATTTTGTAGGAATCATCAGCTTTTAAGAACATACCGGTAGTAATAATGTCCTCAGAGAATGTTCCTGCAAGGATCAGCAGGTAAAGAACTTTAAAGTCTGATGAGCTTTGTGCTCCTTGCGGATACTAATGAAAGGCTCTTATGTTTATTAATCATTAACAGGTGTTTAGAAGAAGGAGCTGAGGAGTTTTTCTTGAAACCAGTAAGAATGGCTGATCTCAACAAGTTGAAACCTCATATGATGAAAACAAAGTTGAACAACCAGAAGCTGGAAGAGATTGAAAAGCCTTTAAATGTATCAGCAGCAGCCGCCGCAGCAGTTGAACCAGAGATTAAAGATTCAGCAGAAGTTGGAAGCAAGATCTTGACTCTTCAGTCTGAACTAGAACCGAAACAAGTACATCTGCAAGTAGCACAACAAGAGGAGCAAACATTGGGTAACAACAACAAGAGGAAGTCCATGGAAGAAGGGCTCTCAACAGATAGATCACGTCCTAGATTCGAGTGTGTCACAACCGCTGTCTGA

>BrRR14(Bra014649)-CDS

ATGGGCATGGCAGCAGTGGAATCGCAGTTTCATGTTTTAGCCGTTGATGATAGTTCATTCGATAGGAAACTCATAGAGAAACTGCTTCAAAAGTCTTCGTGTCAAGTAACAACTGTTGATTCAGGCTATAAGGCTTTAGAGTTTCTTGGTATTGAGAGTAACGACCCAAATGCTCTTTCTACATCTCCTCAGGAGGTTGAAGTGAATCTTATCATTACAGACTATTGTATGCCAGGCATGACTGGTTATGATTTGCTCAAGAAAGTTAAGGAATCATCAGCTTTTAAGAACATACCGGTAGTAATAATGTCCTCAGAGAATGTTCCTGCAAGGATCAGCAGGTGTTTAGAAGAAGGAGCTGAGGAGTTTTTCTTGAAACCAGTAAGAATGGCTGATCTCAACAAGTTGAAACCTCATATGATGAAAACAAAGTTGAACAACCAGAAGCTGGAAGAGATTGAAAAGCCTTTAAATGTATCAGCAGCAGCCGCCGCAGCAGTTGAACCAGAGATTAAAGATTCAGCAGAAGTTGGAAGCAAGATCTTGACTCTTCAGTCTGAACTAGAACCGAAACAAGTACATCTGCAAGTAGCACAACAAGAGGAGCAAACATTGGGTAACAACAACAAGAGGAAGTCCATGGAAGAAGGGCTCTCAACAGATAGATCACGTCCTAGATTCGAGTGTGTCACAACCGCTGTCTGA

>BrRR15(Bra003265)-DNA

ATGGTTATGGCAGCAGAACCGCAGTTTCATGTTTTAGCTGTTGATGATAGTTTATTCGACCGTAAATTTATAGAGAGATTGCTTCAAAAGGCTTCGTGTCAAGGTAAGAAATGTCTTTGTAGCTTGTAAGAATCCAAGAAAGATCTCAGCTTTTCTATGTTCTTGGTGTTTTGTAGTGACAACTGTTGATTCAGGCTATAAGGCTTTAGAGTTTCTTGGTTTAAGAGAAGGTTTTGAGATTAACGACCCAGATGCAGTTTCTACATCTCCTGTGATTCACCAGGTTCGAAAAGTTTGCTCCTTTCTTAAAAAAGAATCATTTCAATTTTAAGTTTACAAGTTTTTTTTAGCAACAAAAAAAAAAAAAGTTTACAAGTTTTTGTCTAGTGGAAGTTTGTTTTATTCTTTTTTGATTGTGACTTTTTGACAGGAAGTTGAAGTGAATCTTATAATTACAGATTATTGTATGCCAGGCATGACTGGTTATGATTTGCTCAAGAAAGTCAAGGTATACTTGTTTTGTTTGTTTTTGTATAATCAAGAAAGTGAAAATGTCAACTAGGAATATTTTTTCTCTTTTGTGATTTGTTTATTTCATATCATTTATGCTATTTGATGTGTACAGTTGATTGATAACATATTTTGGTTTATTCAGGAATTTTAATATAAACTTCTGTTTTATAATTTTGAAGGAATCATCAGCTTGTAAGAACATACCAGTAGTAATAATGTCCTCTGAGAACGTTCCTGCAAGGATCAGCAGGTAAAGAGCCAATGAATGATTGAAACTTTAAAGCTTGAGTGTGTGTACCTCTTTAGGTAGTGCTTATGTTTAGTAGTGTTAATCATGAAACAGATGTTTAGAAGAAGGAGCTGAGGAGTTTTTCTTGAAACCAGTAAGATTAGCTGATCTCAACAAGTTGAAACCTCTTATGATGAAAACAAAGTTGAAGAACCAGAAGCTGGAAGAGATTGAAGCACCTTCAAAAGACGAAAGTGGAACCGTAGCAGCAGTAGTTGAACCAGAGGTTAAAGATTTAACAGAAATCGGAATCAAAATCTTGCCTCTTCAGTCAGAAGTAGAACCGAAACAAGTACATTTGCAAGTAGTACAACAAGAGGAGCAAACAATGAGTAACAACAAGAGGAAGTCAGTGGAAGAAGGGCTCTCAACAGATAGATCACGTCCTAGATTTGAGGGTATCACAACCGCTGTCTGA

>BrRR15(Bra003265)-CDS

ATGGTTATGGCAGCAGAACCGCAGTTTCATGTTTTAGCTGTTGATGATAGTTTATTCGACCGTAAATTTATAGAGAGATTGCTTCAAAAGGCTTCGTGTCAAGTGACAACTGTTGATTCAGGCTATAAGGCTTTAGAGTTTCTTGGTTTAAGAGAAGGTTTTGAGATTAACGACCCAGATGCAGTTTCTACATCTCCTGTGATTCACCAGGAAGTTGAAGTGAATCTTATAATTACAGATTATTGTATGCCAGGCATGACTGGTTATGATTTGCTCAAGAAAGTCAAGGAATCATCAGCTTGTAAGAACATACCAGTAGTAATAATGTCCTCTGAGAACGTTCCTGCAAGGATCAGCAGGTGTTTAGAAGAAGGAGCTGAGGAGTTTTTCTTGAAACCAGTAAGATTAGCTGATCTCAACAAGTTGAAACCTCTTATGATGAAAACAAAGTTGAAGAACCAGAAGCTGGAAGAGATTGAAGCACCTTCAAAAGACGAAAGTGGAACCGTAGCAGCAGTAGTTGAACCAGAGGTTAAAGATTTAACAGAAATCGGAATCAAAATCTTGCCTCTTCAGTCAGAAGTAGAACCGAAACAAGTACATTTGCAAGTAGTACAACAAGAGGAGCAAACAATGAGTAACAACAAGAGGAAGTCAGTGGAAGAAGGGCTCTCAACAGATAGATCACGTCCTAGATTTGAGGGTATCACAACCGCTGTCTGA

>BrRR16(Bra007295)-DNA

ATGGGTATGGCAGCAGCGGAATGGAAGTTTCATGTTTTAGCCGTTGATGATAGTTTAGTCGATCGGAAACTCATAGAGAGACTGCTTCAAAAGTCTTCTTGTCAAGGTATAAACTAGTGTCAACGACATGAACAAGTCTTTGTAGCTTGTAAGAATCTTAGAAAGATCTAAGCTTTTAATGTTTCTTTTATTGGTGTTTGTAGTAACAACTGTTGATTCAGGCTATAAGGCTTTAGAGTTTCTGGGTTTAAGACAAGGTATTGAGAGTAACGACACAACTGCTCTTTCTCTATCTCCTCAGGTTCGAAAAAGTTTGCTCCTTTCTTTTGAAAAAGTCAGTTTAATGATTGGGATTGATTGGAACTTGATGTCTACAAGTCTTTGTGTAATGGAAGATTGTTTATTCTGTTTGGTTTTGACTTTTGACAGGAAGTGAATCTTATCATTACAGATTATTGTATGCCAGGCATGACTGGTTATGATTTACTCAAGAAACTCAAGGTACTTTTTTTTTTTTTTTTTCTTCCTAGTTTATTCGTTTTAGTCTCTGGATCATGTTTCCTAATATATAATCAAGAAAGTGAGAATGTCAATATGTGAGATAGATATTTGGAATATTTTTCTCTGTTGTCATTTTGTTTATCTTCTCTCCTTTATGCTAGTAGATGTGTTGTAAGTTCATTGATAGCATCATCTGTTTTACAACTTTGCAGGAATCATCTGCTCTTAAGAACATACCGGTAGTAATAATGTCATCGGAGAACGTTCCTGCAAGGATCAGCAGGTAAAGGATAATTGATGAGCTTGTGCCCCTCCTTTAGGTTGTGTTTCATCATCATATGGAAGACTCTTTATGTTTCTTAATCATGAAAACAGATGTTTGGAAGAAGGAGCAGAAGAGTTTTTCTTGAAACCAGTAAGATTGGCTGATCTCAACAAGCTGAAACCTCATATGATGAAAACAAAGTTGAAGAACCAGAAGTTGGAAGAGATTATAACACCTTCAAATGACGAAAATGGAACTGTCGCAGCAGTTGAACCAGAGATTAAAGATTCAGTAGAAATGGAAATCATCAAAATGTTGCCTATACAATCAGAAACAGAACCAAAACGAGTGCTTCTGCAAGTGGTACAACAAGAGGAGCAAATGTTGAGTAACAACAAGAGGAAGTCAATGGAAGAAGGGCTGTCAACAGATAGACCACGTCCTAGGTTAGAGGGCATCGCAACCGCTGTCTGA

>BrRR16(Bra007295)-CDS

ATGGGTATGGCAGCAGCGGAATGGAAGTTTCATGTTTTAGCCGTTGATGATAGTTTAGTCGATCGGAAACTCATAGAGAGACTGCTTCAAAAGTCTTCTTGTCAAGTAACAACTGTTGATTCAGGCTATAAGGCTTTAGAGTTTCTGGGTTTAAGACAAGGTATTGAGAGTAACGACACAACTGCTCTTTCTCTATCTCCTCAGGAAGTGAATCTTATCATTACAGATTATTGTATGCCAGGCATGACTGGTTATGATTTACTCAAGAAACTCAAGGAATCATCTGCTCTTAAGAACATACCGGTAGTAATAATGTCATCGGAGAACGTTCCTGCAAGGATCAGCAGATGTTTGGAAGAAGGAGCAGAAGAGTTTTTCTTGAAACCAGTAAGATTGGCTGATCTCAACAAGCTGAAACCTCATATGATGAAAACAAAGTTGAAGAACCAGAAGTTGGAAGAGATTATAACACCTTCAAATGACGAAAATGGAACTGTCGCAGCAGTTGAACCAGAGATTAAAGATTCAGTAGAAATGGAAATCATCAAAATGTTGCCTATACAATCAGAAACAGAACCAAAACGAGTGCTTCTGCAAGTGGTACAACAAGAGGAGCAAATGTTGAGTAACAACAAGAGGAAGTCAATGGAAGAAGGGCTGTCAACAGATAGACCACGTCCTAGGTTAGAGGGCATCGCAACCGCTGTCTGA

>BrRR17(Bra015885)-DNA

ATGGCACTAGGAGATTTATCTTCTTCTTCTTCTTCTACCTCGGAGTTACATGTTCTCGCTGTGGATGATAGTATTGTTGATCGTAAGGTTATTGAGAGGTTGCTTAGGATCTCTGCTTGTAAAGGTATCTCTTTCTTTTGTTTCTCTTTGTTTGGTCTCTGGTTCGGTTTCTGATTAAAAAAATACGGGTTTTGTTTATCTATATATATACAGTGACGACTGTTGAGAGTGGGACTAGGGCTCTTCAGTATCTTGGTCTAGATGGAGACAAAGGATCTTCTGGTCTTAAGGTAATGTTTTCTTTCTTTCTTTCTTCAAAACCTCTCTCAGTTTTCTGACCAAACAAACTCAAGATTGAAATATTTGGTGTTTTCTTCGCTGGTGGTTTGGCTCCCAAGAAATCCAATAGTTGAAACAATCAGGATCACAAGATTTTAACCTGTCTAAATGAAAGATCAGTTTATTTAAAAATTGACCCTTTGTGTATCATTAAGGGTTTGCTTTTGATGTATGGTTTATATGACTAGTTCTTGCAAATGATTTATTGCAGGATTTGAAGGTGAACTTGATAGTGACAGATTACTCTATGCCGGGACTAACAGGATATGAACTACTCAAGAAGATCAAAGTAAACCATTATTCTTTTTTCCATTTTTTTTTCATTATATGAGAGAAGATATTGAATAAAGCTTTACTCAACATAAACTATGTTGAATATTTTATAACCAAGGAGTCTTCAGCATTCAGAGAAATACCTGTAGTGATAATGTCGTCTGAGAACATACAACCTCGTATAGAACAGTAAGTGGCTGTTAGTTAATTATGATATTACAATAATAAATCTCAAACGTTGTTATTTGACTTTGAAATTTTTTGTGTGCATGCAGATGCATGACAGAAGGAGCAGAGGATTTTCTGCTAAAACCGGTGAAATTAGCAGATGTGAAGCGGCTTAAAGAACTCATAATGAGAGGTGGTGAAGCTGAACAAGAGAAAACCAGTAACCTTATTAGTCCTAAGAGAATCCTTCAAAACAACATCTTCTTCTTCATCATCTTCTTTATTATCACTATCACTATCATCTTCATCGTCTAA

>BrRR17(Bra015885)-CDS

ATGGCACTAGGAGATTTATCTTCTTCTTCTTCTTCTACCTCGGAGTTACATGTTCTCGCTGTGGATGATAGTATTGTTGATCGTAAGGTTATTGAGAGGTTGCTTAGGATCTCTGCTTGTAAAGTGACGACTGTTGAGAGTGGGACTAGGGCTCTTCAGTATCTTGGTCTAGATGGAGACAAAGGATCTTCTGGTCTTAAGGATTTGAAGGTGAACTTGATAGTGACAGATTACTCTATGCCGGGACTAACAGGATATGAACTACTCAAGAAGATCAAAGAGTCTTCAGCATTCAGAGAAATACCTGTAGTGATAATGTCGTCTGAGAACATACAACCTCGTATAGAACAATGCATGACAGAAGGAGCAGAGGATTTTCTGCTAAAACCGGTGAAATTAGCAGATGTGAAGCGGCTTAAAGAACTCATAATGAGAGGTGGTGAAGCTGAACAAGAGAAAACCAGTAACCTTATTAGTCCTAAGAGAATCCTTCAAAACAACATCTTCTTCTTCATCATCTTCTTTATTATCACTATCACTATCATCTTCATCGTCTAA

>BrRR18(Bra003782)-DNA

ATGACAGTGGGAGATTTATCTTCTTCTACAATTTCTTCCCCGGAGTTGCATGTTCTCGCCGTCGATGACAGTTTTGTTGATCGTAAAGTTATAGAGAGGTTGCTTAGGATCTCTGCTTGTAAAGGTAATAAAGAGGCAAAACCGTTTCTTAAAATCTTTTTATTTTCCTCCTCTATGTTTGGTCTCTTGATTGTGGGTTTGTCTACTATATAAAGTGACCACTGTTGAGAGTGGGACTAGGGCTCTTCAGTATCTTGGCCTTGATGGAGACGATGCACCTTCTGCTCTTAAGGTACTGTTTCTTTATAAACCTTCTTGCAGAGAAAAAAGGTTTGAAATTTGGACTTGTTTTCTCTTCTGGTTTGGCTCCCAAGAAACTTAGATTTGAATAATGAGGAATACAATATTTAACCTAAATTTTGTTGTCAGTTTCTTGAAAATAAAGCTTTGAAGATTTGCTTTTGATCAGTTGATATGAATAGTTCTGCTAAATTTTGATAAAAACAGGATTTGAAGGTGAACTTGATAGTGACAGATTACTCTATGCCAGGACTAACAGGATATGAACTACTCAAGAAGATCAAAGTAGGTTTACTTTTTTTTTTTATATAGAATTTTTCATTTATGAGAACTTTTTCTTTTTGAATGAAGCTTAGTTACACAGAAACAAAGTTGTTTAATTTTATTACCAGGAGTCATCTGCATTTAGAGAGATACCAGTAGTCATAATGTCCTCTGAGAATATACAACCTCGTATTGACCAGTAAGTGATTGTTATTAGTATGAAACAATTAATCTCAACTATGTAATCTGATTTTAGGTATTTGTTCTCTTTTTTCTTGTAGATGTATGACAGAAGGAGCAGAGGATTTCCTGCTAAAACCGGTGAAGTTAGCAGACGTGAAGCGGTTAACAGAACTTATAATCAGAAATGTTGAACCTGAAGAAGAAGATAAATCCAAACACTCTTATCCTAACAGAATCCTACAAAACAACACTGATTCATCATCGTCTCATGATGATGTCTCCTCTCTGGATGATGACACTCCATCTTCCAAGCGAATGAAAACTAGAATCCACGGCTCATGA

>BrRR18(Bra003782)-CDS

ATGACAGTGGGAGATTTATCTTCTTCTACAATTTCTTCCCCGGAGTTGCATGTTCTCGCCGTCGATGACAGTTTTGTTGATCGTAAAGTTATAGAGAGGTTGCTTAGGATCTCTGCTTGTAAAGTGACCACTGTTGAGAGTGGGACTAGGGCTCTTCAGTATCTTGGCCTTGATGGAGACGATGCACCTTCTGCTCTTAAGGATTTGAAGGTGAACTTGATAGTGACAGATTACTCTATGCCAGGACTAACAGGATATGAACTACTCAAGAAGATCAAAGAGTCATCTGCATTTAGAGAGATACCAGTAGTCATAATGTCCTCTGAGAATATACAACCTCGTATTGACCAATGTATGACAGAAGGAGCAGAGGATTTCCTGCTAAAACCGGTGAAGTTAGCAGACGTGAAGCGGTTAACAGAACTTATAATCAGAAATGTTGAACCTGAAGAAGAAGATAAATCCAAACACTCTTATCCTAACAGAATCCTACAAAACAACACTGATTCATCATCGTCTCATGATGATGTCTCCTCTCTGGATGATGACACTCCATCTTCCAAGCGAATGAAAACTAGAATCCACGGCTCATGA

>BrRR19(Bra000199)-DNA

ATGAATAGTGGTTCTTGTTCATCTTTAATGGAGGTGGGTTATGATGATCATCACCATCATCATGGTCATGAAGAGCTTCATGTTTTGGCCGTGGATGATAATCTTATTGACCGTAAACTTGTAGAGAAGTTGCTCAAGATCTCCTCTTGCAAAGGTTCCATTCTTTTCTCACCTTCCTATTTTTTCTGATTTTAATTTATTTTGTATTTTGTATATATTTTGGCATTAATTCTTTTATGAAATGTGATATTATTGTAGTTACAACAGCAGAGAATGCCATTAGAGCATTGGAGTATTTGGGTTTGGGAGATCAAGATCAGCATATTGATGCACTGACCAATAATGTAAGCAAGATCTATGGGTTTTTTTTTCCCCTTGACACATCTTGTAGGAGCGAAAATCATGATATTGATTCTGACAAATCTATGAAGAAAACTCAAAAATGTATTTTTTTTCTCCAGGATTTGAAGGTGAATCTAATCATCACAGACTATTGCATGCCAGGGATGACAGGCTTCGAGCTACTCAAGAAAGTGAAGGTAATAACAACTATAACAACTACTTCTTGGAATCAAGAATCACATGTTTTATCGGTTTAATTAGTTCAATTTTAACAAATTATCTCATGTGTTTCTATAGCAGGAGTCATCAAATCTGAAAGAGGTCCCTGTTGTGATAATGTCATCAGAGAACATTCCTACTCGAATCAACAAGTAAGAATAATAGAGATCTAATTAAGACCTTGTCTCTGTTTTCTTCCTATGGAAGTAACTAACTATAAGACCATCTTGTGTTCTGTTTTTGGTCTCTTAAATGCAGATGTTTAGCTAGTGGAGCTCAAATGTTTATGCAGAAGCCACTGAAACTATCGGATGTTGAGAAACTTAAATGTCATCTCATGAATTGCAGAAGCTGA

>BrRR19(Bra000199)-CDS

ATGAATAGTGGTTCTTGTTCATCTTTAATGGAGGTGGGTTATGATGATCATCACCATCATCATGGTCATGAAGAGCTTCATGTTTTGGCCGTGGATGATAATCTTATTGACCGTAAACTTGTAGAGAAGTTGCTCAAGATCTCCTCTTGCAAAGTTACAACAGCAGAGAATGCCATTAGAGCATTGGAGTATTTGGGTTTGGGAGATCAAGATCAGCATATTGATGCACTGACCAATAATGATTTGAAGGTGAATCTAATCATCACAGACTATTGCATGCCAGGGATGACAGGCTTCGAGCTACTCAAGAAAGTGAAGGAGTCATCAAATCTGAAAGAGGTCCCTGTTGTGATAATGTCATCAGAGAACATTCCTACTCGAATCAACAAATGTTTAGCTAGTGGAGCTCAAATGTTTATGCAGAAGCCACTGAAACTATCGGATGTTGAGAAACTTAAATGTCATCTCATGAATTGCAGAAGCTGA

>BrRR20(Bra007242)-DNA

ATGGAGGAAGAGCTCCATGTTTTAGCAGTAGATGACAATCTAATGGACCGTAAACTTGTGGAGAGAATCCTCAAGATCTCTTCTTGCAAAGGCAAATTATATTACTCAAACTTCCAATTTTTTATTTTCCTTTTAAATCCCAAGAACCAAGATTCTTCTATGTTATGGTATTATGGTAATATTACCTCTTAAATTTTGAAAACTGATTTACATTTTTTTTTCTCTTTGCAGTGACAACAGCAGAAAATGGGATCAGAGCATTGGAGTACTTAGGCTTGGGAGATTCACAACAGGCTGATTCATCAAGTACCAACAATGTAAGAATAATCTTCAAGTTATTTAATCCTCAGAGGACAACATTTTGATTGGTGTAGAGAGGATCCAATTGTGTAAATCCAATGACTTTGTGACATTGTGTTGTATTTTTACAGGTTATGAAGGTGAATCTTATCATAACTGATTACTGTATGCCAGGGATGACAGGTTTTGAGCTGCTCAAGATAGTAAAGGTGTTTAACAATTTTTCTTAGTAATGAAAGTCTTCTTGAGAAGATTCAGAGCCTAACATTTTATTTGGTCTTTTGTAATTGACAGCAGGAATCGTCAAATCTAAAGGAAGTACCTGTTGTGATATTGTCATCAGAGAACATTCCTACTCGCATCAACAAGTAACCTTCTTATTCCTTATGATATGACATATTGCTGTTCTGTTTTTTTTTAATGATTCAGTGGTGAATTAAGATTCTGTTATTTTCTTGATGATCTTTGCAGATGTTTAGCCAGCGGAGCTCAGATGTTTATGCAGAAGCCACTGAAACTGTCGGATGTAGAGAAACTCAAGTGTCATCTCTTAAACTGCAGAAGCTGA

>BrRR20(Bra007242)-CDS

ATGGAGGAAGAGCTCCATGTTTTAGCAGTAGATGACAATCTAATGGACCGTAAACTTGTGGAGAGAATCCTCAAGATCTCTTCTTGCAAAGGCAAATTATATTACTCAAACTTCCAATTTTTTATTTTCCTTTTAAATCCCAAGAACCAAGATTCTTCTATGTTATGGTATTATGTGACAACAGCAGAAAATGGGATCAGAGCATTGGAGTACTTAGGCTTGGGAGATTCACAACAGGCTGATTCATCAAGTACCAACAATGTTATGAAGGTGAATCTTATCATAACTGATTACTGTATGCCAGGGATGACAGGTTTTGAGCTGCTCAAGATAGTAAAGCAGGAATCGTCAAATCTAAAGGAAGTACCTGTTGTGATATTGTCATCAGAGAACATTCCTACTCGCATCAACAAATGTTTAGCCAGCGGAGCTCAGATGTTTATGCAGAAGCCACTGAAACTGTCGGATGTAGAGAAACTCAAGTGTCATCTCTTAAACTGCAGAAGCTGA

>BrRR21(Bra014695)-DNA

ATGGACGAAGAGCTTCATGTTTTAGCAGTAGATGACAATCTCATTGACCGTAAACTCGTAGAGAGAATTCTCAAGATCTCTTCCTGCAAAGGCAAGTTATATCATTTTTTGTTAGCTTTTAAAATTCTAGAACCAAGATTACCTCTGAAATGTAATAATTGATTCTTTTTTGCAGTGACAACAGCAGAAAATGGGCTTAGAGCATTGGAGTACTTAGGCTTGGGAGATCCACAACAGACTGAGTCATTAACTACCAACAGTGTAAACTTAACCTTCTAGTTATTTAATACCCAGAGAACATCATTTTCATTGGTTCAGAGAGGACTTATGTGTAAATCCAATGAGTTTTGTTACATTTTTTACAGGTTATGAAGGTGAATCTTATCATCACTGATTATTGTATGCCAGGGATGACAGGTTTTGAGCTTCTGAAGATAGTAAAGGTGTTTAAAAAATTTCTTATTAGGAATCAAGAGTCTTTTTGAGAAGATTCAGAGATTAACATTTTTTCTGCTGGTTTCTATGTTTTTTTGTTTTTGTTATTGACAGCAGGAATCTTCGAATCTTAAGGAAGTACCTGTTGTGATTTTGTCATCAGAGAACATTCCTACTCGCATCAATAAGTAAACTTATTATCTCTCGTGATGAGACATTGCTCTCTTTATTGAGTCGGTGGTGAATTGAGATTCTATTATTTTCTTGGTGATATTTGCAGGTGTTTAGCCAGCGGGGCTCAGATGTTTATGCAGAAGCCATTGAAATTATCGGATGTAGAGAAACTCAAGGGTCATGTCTTAAACTGCAGGAGCTGA

>BrRR21(Bra014695)-CDS

ATGGACGAAGAGCTTCATGTTTTAGCAGTAGATGACAATCTCATTGACCTGACAACAGCAGAAAATGGGCTTAGAGCATTGGAGTACTTAGGCTTGGGAGATCCACAACAGACTGAGTCATTAACTACCAACAGTGTTATGAAGGTGAATCTTATCATCACTGATTATTGTATGCCAGGGATGACAGGTTTTGAGCTTCTGAAGATAGTAAAGCAGGAATCTTCGAATCTTAAGGAAGTACCTGTTGTGATTTTGTCATCAGAGAACATTCCTACTCGCATCAATAAGTGTTTAGCCAGCGGGGCTCAGATGTTTATGCAGAAGCCATTGAAATTATCGGATGTAGAGAAACTCAAGGGTCATGTCTTAAACTGCAGGAGCTGA

>BrRR22(Bra001641)-DNA

ATGTTTTCGTCGGGTCTTCGAGTTCTAGTGGTTGATGATGACCCGACTTGTCTTGCAATCTTAGAGAGGATGCTCAGGGCTTGTAGTTACGAAGGTTCGAATATCAAAGAAGATCTTTTTCGGTTTAGTAATTTTGTGGAGATGCTGTTGACTTTGACTTGAGTTTGGTTTTTGACTGTCGATTCAAAGAGAGATCCTTGTTAGTTAGTTAGTTAGTTAGTTAGGTAGTTAGTTAGTTAGTTAGTACTCAGAGACTCAAAACAGTTCTGTCTTAATTTCATTTGATGAATCTCTCTTTGTTTTCAGTAACGAAATGCAACAGAGCAGAGATGGCATTGTCTCTGCTCCGGAAGAACAAACATGGATTCGACATAGTCATCAGCGACGTTCACATGCCTGACATGGACGGCTTCAAGCTCCTTGAACACGTCGGTCTTGAGATGGACTTGCCTGTTATCAGTACGTGTTGTTATATATCTCTGTTTTAAGTTTGAATCTTTTTGTTCTAACAAAGATCTATTTTTTTTGTGAAATTTGTTTTTAACGCAGTGATGTCTGCGGATAATTCAAAGAGAGTGTGGTTCTAAAGGGAGTAACGCACGGTGCGGTTGATTATCTTATCAAACCTGTGCGCATAGAGGCACTTCAGAACATATGGCAGCACGTGGTTCGGAAGAGGAGGAGCGTACCTGAACATTCTGACGGGGAAGATGCAGCGGATGACAACTCTTCCTCTGTTAATGGAGGGAAGAAGTGGAGGAGCATGTTATGA

>BrRR22(Bra001641)-CDS

ATGTTTTCGTCGGGTCTTCGAGTTCTAGTGGTTGATGATGACCCGACTTGTCTTGCAATCTTAGAGAGGATGCTCAGGGCTTGTAGTTACGAAGTAACGAAATGCAACAGAGCAGAGATGGCATTGTCTCTGCTCCGGAAGAACAAACATGGATTCGACATAGTCATCAGCGACGTTCACATGCCTGACATGGACGGCTTCAAGCTCCTTGAACACGTCGGTCTTGAGATGGACTTGCCTGTTATCAAGAGTGTGGTTCTAAAGGGAGTAACGCACGGTGCGGTTGATTATCTTATCAAACCTGTGCGCATAGAGGCACTTCAGAACATATGGCAGCACGTGGTTCGGAAGAGGAGGAGCGTACCTGAACATTCTGACGGGGAAGATGCAGCGGATGACAACTCTTCCTCTGTTAATGGAGGGAAGAAGTGGAGGAGCATGTTATGA

>BrRR23(Bra022183)-DNA

ATGATGAATCCGAGTCAGGGAAGAGGACTCGGATCGGGTGGTGGGTCGAGCTCCGGTAGAAACAAAGGAGGTGAAGCCGTGGTGGAGATGTTTCCGTCGGGACTTAGAGTACTAGTGGTTGACGATGACCCGACTTGTCTTATGATCTTAGAGAGGATGCTCAGAACTTGTCTCTACGAAGGTTCGAATTCACAAAGACAAGATCTTCTCTTCTAGTTTTTGTTTTGTTTTTTTCTTTTTGGGTTTAGTAATTTTTGATGATTAATCTCACGAGTTTATGTGTCTCTCTGTCCTAATGATTTATTGTGGTCCATTCTCGCAAAAGAAAAAAAAAAGATGTTATTTTGAGGGATAATTATGGTTTTGAGTTTAGGTTGTTGACTAACTCTGCTCTCCCAAATATAGAAACTTTTTAAGATTCACAATATTAATCTTCTTCACTTCCTTTGATTTAGAGATCACATGTTTTTTATTTATTTAACTTGAGGTTTAGAAGGATTCAAATCTGAGAGTCTCTTATGTTGCTGAGCTTTTGACTTGAGTTTGGTTTAGATCATGTTAATTCATGAGATCCTTGTGTCTCTCTTTGATCTAAGTTTAATTAATAGAGATCTCTCTTTTTTTATTTTTTTTCTTGTGTGTGTGTTTGTGACAATTCAGTAACGAAATGCAACAGAGCAGAGATGGCATTGTCTCTGCTCCGGAAGAACAAACACGGATTCGATATTGTCATCAGCGATGTTCACATGCCTGACATGGACGGCTTCAAGCTTCTTGAACATGTTGGTCTAGAGATGGACTTGCCTGTCATCAGTACGTTTCATTAAAAAAATTCCAAAAGCTTGAATCTTTTTATATATGGTTTCTAATTACTGATCTTTGTTTTAATCCTCAGTGATGTCTGCGGATGATTCAAAGAGTGTGGTTCTAAAAGGAGTCACTCACGGTGCGGTTGATTACCTCATCAAACCGGTGCGTATGGAGGCGCTCAAGAACATTTGGCAGCACGTGGTTCGGAAGAGGAGGAGCGAGTGGAGCGTGCCGGAACATTCTGGTAGCATTGAGGAGACCGGACAGCAGCAGCAACAGAGAGGTCCAGCTGTTTCTGAGGATGCGGCAGATGATAACGCTTCCTCTGTTAACAACGAAGGGAACAACTGGAGGAGCAGCAGCAACAACTCGCGGAAACGGAAAGAAGAGGAAGGGGATGAGCAAGGGGACGAAGACGCTTCCAATCTGAAGAAACCGCGTGTTGTCTGGTCTGTTGAGTTGCATCAGCAGTTTGTTGCTGCTGTTAATCAGCTCGGCGTCGAAAGTAAAAGACTCTTAAAGCTTCTTTACATTTTGCATTTGTGAGATATGTTTTGGGGTCTAATTAATTTTCTTTGGGGCAGAGGCGGTTCCTAAGAAGATCTTAGAGCTGATGAATGTTCCTGGTCTAACCCGTGAGAATGTAGCCAGTCACCTTCAGGTATAAACCTTTATATACCTTAAACCGAAACATATGACCGGATCTGAGATTTTGAGAGCTATAAACATTTCTTTTTTTTTTTTGATCAAAAGCTATAAACATTTCTTTAAGAACTTTAATTATTATTACTCCATCTGATATAAAAAGATCCATGTTTTAGAAAAATATGTTTCTAAATATACAACTTTACATTTTAAATGCAGTTTTTATTTTTATTCGACAAAAAATGAAGCTTTTATTAAGTAACAATGGTAACTGTTTATTGAATTTATATTGGTTAAAAGTTACGAAAAATAATTAATCTCAATAAACAATACATTTATGTGAAATTTCGAAAAATAGAGTATTTTGAAACAGAAAAGTATTTTCTTTAAATCATTTATGGGCTTTTACCCATATATATATGTATATAATCTTCAAAAATTTTGGGGTCAAGTCAATGTTTCATTGGGATGTGTGCCCTGCTGGAACGAGCAGTTGTATTGTATCATACAGTATATTCTTAACCGGTTTTGTGTTGGCTTGCAGAAATACAGGATATACCTAAGACGCCTCGGGGGAGTCTCACAGCACCAAGGAAACTTAAACAACTCGTTTATGACGGGTCAGGACGCTAGCTTCGGTCCTCTTTCTTCACTGAATGGGTTTGATCTTCAAGCGTTAGCAGTCACAGGTCAGCTCCCTGCTCAGAGCCTTGCGCAGCTTCAAGCCGCTGGTTTAGGCCGGCCTGCGATGGTCTCCAAGTCAGGGCTACCTGTTTCCTCCGTCGTGGACGAGAGAAGCATCTTCAGCTTCGACAACTCGAAACCAAGATTCGGAGATGGGATTGGTGGTCATCAAACACAGCAGCCGCAGATGAACTTGCTCCACGGTGTCCCCACGGGTATGGAGCCTAGACAGCTCGCGGGGTTGCAACAGCAGCTTCCTGTTGGTGGTAATAGAATGAGCATTCAACAACAGATTGCTGCGGTTCGAGCCGGACATAGCGGAATGCTGATGCCTCAGCAGCAGCCATTCCCGCGAGGGCCACCCTCCATCAGGCAGCCTATGTTACCAAACCGTATAACTGAGAGAAGCGGCTTCTCGGGGAGGAGCAGTGTTCCAGAGAGCAGCCGAGTGTTACCTACAAGCTACACCAACCTCGCAACGCAACAACACTCATCAACTTCGGTAGCCTTCAACAGCTTCCAGCAAGAGCTCCCAGTGAACAGCTTCCCGCTTCCTAGTGCACCAGGCTTATCAGTTCCGACTCAGGTTCGGAAACCACATTCTTCCTCTTCTTCTTACCAAGAAGAGGTTAACAGCTCGGAAGCGGGCTTCGCCACCCCAAGCTACGACATGTTCTCCAACAGACATAACGACTGGGATCTTCGGAGTATCGCCTTCGACGCGCATCAAGACGCAGAATCCGTAGCCTTCTCCAACTCAGAAGCCTTCTCTTTTTCCTCCATGTCAAGAAACAACAACAACAACGCAACGGTTGCAGCCACGGATCTTGGCCGGAACCAGCAGCAAACACTGTCAGGCATGGTACCGCATCATCAGGTTTATGGCAACGGAGGGGGCGGTGGCAGTTCAGTGAGGGTGAAGTCGGAGAGAGATACAGCAGCGATGGCGTTCCACGAGCAGTATAGTAATCAAGAAGATCTTATGAGTGCACTTCTTAAGCAGGTTTGATTTTTTTTTTTGGTTTCTCTCTTATAAACGTTTAGCATTTAGAGTTTTGCGTTTTCAGTATATAACGCTTTCTCACCACTGGTTGCGTGTGTACCTTTCATGATTACGCAAACTACCACTAGCCAAAATAATGAATCTAGAATCTTTTTTTTTCTCTCACGAGAAGTGGATAATAAGTCAACTTTAAAGAGCTTCTTGGGCACTTTTTGTAATAATGTTTGTGTTCAAATTCCGACAGAAGAGCAAATAGAAAGAAACGTTGACTTAATAACAGAACAAACAATCATGTGTTGTTGTTGATTACAGGAAGGAGTGGCACCGGTTGTTGATACCGAGTTCGACTTTGACGCATATTCAATCGATGATATCCCGGTTTGA

>BrRR23(Bra022183)-CDS

ATGATGAATCCGAGTCAGGGAAGAGGACTCGGATCGGGTGGTGGGTCGAGCTCCGGTAGAAACAAAGGAGGTGAAGCCGTGGTGGAGATGTTTCCGTCGGGACTTAGAGTACTAGTGGTTGACGATGACCCGACTTGTCTTATGATCTTAGAGAGGATGCTCAGAACTTGTCTCTACGAAGTAACGAAATGCAACAGAGCAGAGATGGCATTGTCTCTGCTCCGGAAGAACAAACACGGATTCGATATTGTCATCAGCGATGTTCACATGCCTGACATGGACGGCTTCAAGCTTCTTGAACATGTTGGTCTAGAGATGGACTTGCCTGTCATCATGATGTCTGCGGATGATTCAAAGAGTGTGGTTCTAAAAGGAGTCACTCACGGTGCGGTTGATTACCTCATCAAACCGGTGCGTATGGAGGCGCTCAAGAACATTTGGCAGCACGTGGTTCGGAAGAGGAGGAGCGAGTGGAGCGTGCCGGAACATTCTGGTAGCATTGAGGAGACCGGACAGCAGCAGCAACAGAGAGGTCCAGCTGTTTCTGAGGATGCGGCAGATGATAACGCTTCCTCTGTTAACAACGAAGGGAACAACTGGAGGAGCAGCAGCAACAACTCGCGGAAACGGAAAGAAGAGGAAGGGGATGAGCAAGGGGACGAAGACGCTTCCAATCTGAAGAAACCGCGTGTTGTCTGGTCTGTTGAGTTGCATCAGCAGTTTGTTGCTGCTGTTAATCAGCTCGGCGTCGAAAAGGCGGTTCCTAAGAAGATCTTAGAGCTGATGAATGTTCCTGGTCTAACCCGTGAGAATGTAGCCAGTCACCTTCAGAAATACAGGATATACCTAAGACGCCTCGGGGGAGTCTCACAGCACCAAGGAAACTTAAACAACTCGTTTATGACGGGTCAGGACGCTAGCTTCGGTCCTCTTTCTTCACTGAATGGGTTTGATCTTCAAGCGTTAGCAGTCACAGGTCAGCTCCCTGCTCAGAGCCTTGCGCAGCTTCAAGCCGCTGGTTTAGGCCGGCCTGCGATGGTCTCCAAGTCAGGGCTACCTGTTTCCTCCGTCGTGGACGAGAGAAGCATCTTCAGCTTCGACAACTCGAAACCAAGATTCGGAGATGGGATTGGTGGTCATCAAACACAGCAGCCGCAGATGAACTTGCTCCACGGTGTCCCCACGGGTATGGAGCCTAGACAGCTCGCGGGGTTGCAACAGCAGCTTCCTGTTGGTGGTAATAGAATGAGCATTCAACAACAGATTGCTGCGGTTCGAGCCGGACATAGCGGAATGCTGATGCCTCAGCAGCAGCCATTCCCGCGAGGGCCACCCTCCATCAGGCAGCCTATGTTACCAAACCGTATAACTGAGAGAAGCGGCTTCTCGGGGAGGAGCAGTGTTCCAGAGAGCAGCCGAGTGTTACCTACAAGCTACACCAACCTCGCAACGCAACAACACTCATCAACTTCGGTAGCCTTCAACAGCTTCCAGCAAGAGCTCCCAGTGAACAGCTTCCCGCTTCCTAGTGCACCAGGCTTATCAGTTCCGACTCAGGTTCGGAAACCACATTCTTCCTCTTCTTCTTACCAAGAAGAGGTTAACAGCTCGGAAGCGGGCTTCGCCACCCCAAGCTACGACATGTTCTCCAACAGACATAACGACTGGGATCTTCGGAGTATCGCCTTCGACGCGCATCAAGACGCAGAATCCGTAGCCTTCTCCAACTCAGAAGCCTTCTCTTTTTCCTCCATGTCAAGAAACAACAACAACAACGCAACGGTTGCAGCCACGGATCTTGGCCGGAACCAGCAGCAAACACTGTCAGGCATGGTACCGCATCATCAGGTTTATGGCAACGGAGGGGGCGGTGGCAGTTCAGTGAGGGTGAAGTCGGAGAGAGATACAGCAGCGATGGCGTTCCACGAGCAGTATAGTAATCAAGAAGATCTTATGAGTGCACTTCTTAAGCAGGAAGGAGTGGCACCGGTTGTTGATACCGAGTTCGACTTTGACGCATATTCAATCGATGATATCCCGGTTTGA

>BrRR24(Bra001643)-DNA

ATGATGAATCGAGGACTCGGATCGGGCGGTGGGTCGAGCTCCGGCAAGAATCAAGGCGGTGAAGCCGTGGTGGAGATGTTCCCGTCGGGGCTTAGAGTTCTAGTGGTTGACGATGACCCGACTTGTCTTATGATCTTAGAGAGGATGCTCAGAACTTGTCTCTACGAAGGTTCGAAAAATCACAAAGACAAGATCTTTAGTTTGGTTCGGTTTAGTCAAATTTGATTTGTGTCTTCGTATGTAATGATGGGTTGACTAACCTAAAAATAGAAACTTTATTTAAGATTCACAATATTAGATCATGTCTGGAGTCTATCATGTTAAAAGGATGCAAATCTGAGAGTCTCTCTAGCTTTTGACTTGAGTGTTTGGTTAGTTAGTTAGTTAGTTAGGAGACTCAAAAACAGTTCTGTCTTAATTTGATTTGACTTGATGAATGAATCTCTGTGTGTTTTCAGTAACGAAATGCAACAGAGCAGAGATGGCATTGTCTCTGCTCCGGAAGAACAAACATGGATTCGATATAGTCATCAGCGACGTTCACATGCCTGACATGGACGGCTTCGTGCTCCTTGGACACGTCGGTCTTGAGATTGACTTGCCTGTTATCAGTAAGTTTTTTTTAAAAAATTAAATCCCAATATTTTTATACAACTGTTAAGAGTACTAAACAAGGATCTTGTTTAAACTCCAGTGATGTCCGCGGATGATTCAAAGAGTGTGGTTCTAAAGGGGGTGACTCACGGCGCGGTTGACTACCTAATCAAACCTGTACGCATGGAGGCGCTCAAGAACATATGGCAGCACGTGGTGCGCAAGAGGAGGACCGAGTGGAGCGGCGTGCCTACACATTCTGGGAGCGTTGAGGAGACTGGCGAGAGGCGGCAGCAGCAGCAACAGAGAGAAGCTGTTTCCCGTGGGGAGGAAGATGGAGCTGATGACAACAACTCTTCCTCTGTTAACGAAGGCGGCAACAACAACTGGAGGAATAGCAGCAGCAGCAGCTCAAGGAAGCGGAAAGAAGAGGAAGGAGGGGAAGAACAAGGTGACGAAGATGCTTCTAATCTCAAGAAACCGCGTGTTGTCTGGTCTGTTGAGTTGCATCAACAGTTTGTTGCTGCTGTTAATCAGCTCGGCGTCGAAAGTAAGACTAAACACTAAAGCTTTGCGTTTTTGAAAGAGTCTAATTCATCTTCTATGATGCAGAGGCGGTTCCTAAGAAGATCTTGGAGCTGATGAATGTCCCTGGACTAACCCGAGAGAACGTAGCCAGCCACCTCCAGGTATATAAAAACCTTATATACTGCGAGGGCCAGCCCTGAGATTTTGGGGGCTATAGACGATTTAATAAAGATTTCGATTATTTGGGTGCCTAAATTTTTTTTTTTCTTACAAATTTGGGGTCTGAGGCGAATGTTTCATCGGGGTTGGCGCGCCTTGTATACCAGAATGACGATATATGTTCTTACCGGGCGTAAGTGTTGATTTTGCAGAAATACAGAATATATCTAAGACGGCTTGGAGGAGTGTCACAGCACCAAGGCAGTTTAAACAACTCGTTTATGACAAGTCAGGACGCTAGCTTTGGTTCTCTTCCGACGCTGAACGGGTTTGATCTTCAAGCGCTAGCTCAGCTTCCTGCTCAAAGCCTTGCACAGCTACAAGCTGCCGGTTTAGGCCGGCCTGCTGCGATGAACTCTAAGCCGGGATTGCATGTTTCTTCCTCCATTGTGGACGAGAGAAGCGTCTTCAGCTTTGACAACCCGAAAATGAACTTGCTTCACGGTGTCCCCACCGGTATGGAGCCGAGACAGCTTGCGGGGTTACAACAACATCGAATGACTATTCAACAGCAGATTGCTGCGGTTCGAGCAGGACATAGCCTTCAGAACAACGGTATGCGGATGCCGCTAGCGTCTCAGCCGCAGCAGCCATTCTCAAGACCACAACAATCTTCCATCAGACAGCCAATGTTACCGAACCGTAGTGGTTTCTCGGGAAGGAGTAGTATCCCGGAGAGCAGCAGAGTGTTACCTACTACAAGCTACACCAACCTCGCAGCTCAACAACAACACTCGATGGCCTTCAGCAACTTCCAACAAGAGCTCCCTGTGAATAGCTTCCCGCTAGCGAGTGCACCAGGCTTATCTGTTCGGAAACCACACTCTTCTTCTTCTTCTTCTTCTTCTTCTTACAGAGAAGAGTTTAACAGCTCGGAAGCAGGCTTCAGTACGCCTAGCTACGACATGTTCTCAAGCAGACAGAACGATTGGGATCTAAGGAGTATGCTTTCCCCTCATCAAGATTCACAAGCCTACTCTTCTTCCTCCATGTCAAGAAACAACAACACGGCGGTGGCAGCCACTGATCATAGCCGGAACCACCAGCAGACACCGCAGGGTATGGTCTCGCATCATCAGGTTTATGGAAACGGAGGGGGAAGTTCAGTGAAGGTGAAGTCAGAGACAATGGGGTTCCACGAGCAGTATAGTAATCAAGAAGATCTTATGAGCGCACTTCTTAAGCAGGTTTGCTTTTGAACACATTCTTATATATAATAGGACGCATAATCTGATCGCAAACGCACATCACAAGCCGCAACTTGTCGCGTTTGGAAATGAAATATATATGAACATTAATTTTGGTGTTGATACAGGAAGGAATTGGACCGGTTGATACAGAGTTCGACTTCGACGCATATTCGATTGATGATATCCCGGTTTGA

>BrRR24(Bra001643)-CDS

ATGATGAATCGAGGACTCGGATCGGGCGGTGGGTCGAGCTCCGGCAAGAATCAAGGCGGTGAAGCCGTGGTGGAGATGTTCCCGTCGGGGCTTAGAGTTCTAGTGGTTGACGATGACCCGACTTGTCTTATGATCTTAGAGAGGATGCTCAGAACTTGTCTCTACGAAGTAACGAAATGCAACAGAGCAGAGATGGCATTGTCTCTGCTCCGGAAGAACAAACATGGATTCGATATAGTCATCAGCGACGTTCACATGCCTGACATGGACGGCTTCGTGCTCCTTGGACACGTCGGTCTTGAGATTGACTTGCCTGTTATCATGATGTCCGCGGATGATTCAAAGAGTGTGGTTCTAAAGGGGGTGACTCACGGCGCGGTTGACTACCTAATCAAACCTGTACGCATGGAGGCGCTCAAGAACATATGGCAGCACGTGGTGCGCAAGAGGAGGACCGAGTGGAGCGGCGTGCCTACACATTCTGGGAGCGTTGAGGAGACTGGCGAGAGGCGGCAGCAGCAGCAACAGAGAGAAGCTGTTTCCCGTGGGGAGGAAGATGGAGCTGATGACAACAACTCTTCCTCTGTTAACGAAGGCGGCAACAACAACTGGAGGAATAGCAGCAGCAGCAGCTCAAGGAAGCGGAAAGAAGAGGAAGGAGGGGAAGAACAAGGTGACGAAGATGCTTCTAATCTCAAGAAACCGCGTGTTGTCTGGTCTGTTGAGTTGCATCAACAGTTTGTTGCTGCTGTTAATCAGCTCGGCGTCGAAAAGGCGGTTCCTAAGAAGATCTTGGAGCTGATGAATGTCCCTGGACTAACCCGAGAGAACGTAGCCAGCCACCTCCAGAAATACAGAATATATCTAAGACGGCTTGGAGGAGTGTCACAGCACCAAGGCAGTTTAAACAACTCGTTTATGACAAGTCAGGACGCTAGCTTTGGTTCTCTTCCGACGCTGAACGGGTTTGATCTTCAAGCGCTAGCTCAGCTTCCTGCTCAAAGCCTTGCACAGCTACAAGCTGCCGGTTTAGGCCGGCCTGCTGCGATGAACTCTAAGCCGGGATTGCATGTTTCTTCCTCCATTGTGGACGAGAGAAGCGTCTTCAGCTTTGACAACCCGAAAATGAACTTGCTTCACGGTGTCCCCACCGGTATGGAGCCGAGACAGCTTGCGGGGTTACAACAACATCGAATGACTATTCAACAGCAGATTGCTGCGGTTCGAGCAGGACATAGCCTTCAGAACAACGGTATGCGGATGCCGCTAGCGTCTCAGCCGCAGCAGCCATTCTCAAGACCACAACAATCTTCCATCAGACAGCCAATGTTACCGAACCGTAGTGGTTTCTCGGGAAGGAGTAGTATCCCGGAGAGCAGCAGAGTGTTACCTACTACAAGCTACACCAACCTCGCAGCTCAACAACAACACTCGATGGCCTTCAGCAACTTCCAACAAGAGCTCCCTGTGAATAGCTTCCCGCTAGCGAGTGCACCAGGCTTATCTGTTCGGAAACCACACTCTTCTTCTTCTTCTTCTTCTTCTTCTTACAGAGAAGAGTTTAACAGCTCGGAAGCAGGCTTCAGTACGCCTAGCTACGACATGTTCTCAAGCAGACAGAACGATTGGGATCTAAGGAGTATGCTTTCCCCTCATCAAGATTCACAAGCCTACTCTTCTTCCTCCATGTCAAGAAACAACAACACGGCGGTGGCAGCCACTGATCATAGCCGGAACCACCAGCAGACACCGCAGGGTATGGTCTCGCATCATCAGGTTTATGGAAACGGAGGGGGAAGTTCAGTGAAGGTGAAGTCAGAGACAATGGGGTTCCACGAGCAGTATAGTAATCAAGAAGATCTTATGAGCGCACTTCTTAAGCAGGAAGGAATTGGACCGGTTGATACAGAGTTCGACTTCGACGCATATTCGATTGATGATATCCCGGTTTGA

>BrRR25(Bra033527)-DNA

ATGTTAAATCCGGGTCAGGGAAGAGGACCCGATTCGGGAGTCGCTGGTGGGTCGTCGAACTCCGACCCGTTTCCTGCGGGTCTTCGAGTTCTCGTGGTCGACGATGACCCAACTTGTCTGATGATCTTGGAGAGGATGCTCAGGACTTGTCTCTACAGAGGTTCTCTTGGTCTTTCTTTTGATGATTTCTTGGTCCAATTTTCCGATTTTTTTTTTGTTTTGGTAATTTTGTGAAATTATTATGGGATTTTAATACGGTGGTTGACTTAATCCATTAATAGAAAAACGATCTTTGAAATTTGACTAGAAGACTGTTTTAATCACTGTTAGATTCAGAGTGTATATATTACACATAACTCTCTGCTCTGTTCTACATCAATCTGTGATCTGGTTAAAGATTTTGATTTTGCGGTTTATTTTTGTTATTGAGTTTATTTTAAGAAGCCTGGTAATGATTTTGATCAATTCTTGGTTCGGTTTCTGTGTCTTGAATTTAACTCTTGACTCTGTTTTTTTTTATTTCAACAGTAACGAAGTGTAACAGAGCAGAGATCGCATTGTCTCTGCTACGAAAGAACAAGAACGGTTTCGATATTGTCATCAGTGATGTTCATATGCCTGACATGGACGGATTCAAGCTCCTAGAACACGTCGGTCTAGAGATGGATCTACCTGTTATCAGTACGTTTACTTTCTTTAACATTATATAAAACACTCTCTACTTGTTTAGATCTGATGAATCTTTTTTTAAATGGTTTTTAGTGATGTCTGCGGATGATTCAAAGGCCGTTGTGTTGAAAGGAGTGACTCACGGTGCAGTGGACTACCTCATCAAACCAGTACGCATCGAGGCGCTCAAGAACATATGGCAGCATGTGGTGAGGAAGAAGCGTAACGAGTGGAATGTCTCTGAACATTCCGGAAGTGTTGAAGAGACTGGGCAGAGGGAAGATGGTGATAACAACTCTTCTTCAGCTAATAACGAAGGGAGCTGGAGGGGCTCGAGGAAGAGGAAGGAAGAGGAAGTAGACGAGCAAGGGGGGGATGATAAAGAGGACACGTCTAGCTTGAAGAAACCACGTGTGGTTTGGTCTGTTGAGTTGCATCAACAATTTGTCGCTGCTGTGAATCAGCTTGGCGTTGACAGTAAGTGAAAAAAAAGAAGTAAAAACTTGTTTGCTTTGTGAGTTTGTGTTTTGTTGTCTAAATCTTTTGTTTAATGCAGAAGCGGTTCCGAAGAAGATCTTGGAGATGATGAATGTACCAGGACTAACGAGAGAAAACGTAGCTAGTCACCTCCAGGTAAGACATGTAACTGAATAAGTATTACTTTGATGTGTGCTAACATACCATTGTGGTTATTGCAGAAGTATAGGATATATCTAAGACGGCTTGGAGGAGTGTCTCAGCACCAAGGGAACATGAACCATTCGTTTATGACAGGTCAAGATCCGAGTTTCGGTCCTCTTTCTACGTTGAATGGGTTTGATCTTCAAGCTCTAGCTGCTGCTGGTCAGCTCCCAGCTCAGAGCCTCGCACATCTTCAAGCAGCTGGTCTTGCTCGGCCTCCTTCACTCACTAAACCGGGGATGTCCGTAGATCAGAGAAGCATCTTCAGCTTCGAAAACCCAAAAATAAGACATGGGCAGATGATGAACAGTGGTGGTGGTGGTAATAAGCAGATGAATCTGCTTCACGGTGTTCCAATGGGAATGGAACCAAGACAGTTTACAGGTGGTGGTCAAATGCGCGTGCAGCAGCAGCAGCAACAATTGTCTGGTGGTCGTGCTGTTGGACAGAATGTTCAGAGCAGTGGGATGATGATGCCAGTAGGTGGAGGGCCATCAATGCTACAACAGCAACAACAAGTGATGTTGTCGAGTAGTGTTCCAAGGAGAAGCGAGACAAGCAGCAGCAGTAGAGTGTTACCAGCTGCTGCTACTACACAGTCGGTGGTCTTTAATAACTTTTCCTCGGAGCTACCTAGAAACAGCTTCCCGTTGGCAAGTGCACCTGGGATATCAGTTTCTTACCAAGAAGAAGTCAACAGCTCAGACGCAAAAGGTGGTGCTGGGTTTGGTAACCCGAGCTACGACATATTCAATGATTATCCGCAGCAGCACAACAACAACAATGATTGGGATCTGCAGAATATCGGCATGGTCTTTAATTCTCATCAGGACACAACAACAGCATCTGCCGCTTTTTCATCTTCGTCCTCCACTCAGAGACAAAGGGCTGAGCATGTGCAGAACCATCACCAGCAACAGCAGTTGCCAAGCCAGAGTCGTAATCACATGAACGGCGGTGGTTCGGTTAGAGTGAAGTCAGAGAGAGTGGCGGAGACTGTGACTTGTCCTCCAGCAACAACACTGTTTCAAGAGCAGTATAATCAAGAAGATCTCATGAGCGCACTTCTCAAACAGGTTTCATTTTCTATAGACTAGTCATATTAGAGGAGATGATACGAATATTAACTTACATTCTCGTTTTACTCAAAATGCAGGAAGGACTTCCATTGGTAGATAACGAGTTCGACTTTGACGGATACTCCTTCGATAATATTCCTGTCTGA

>BrRR25(Bra033527)-CDS

ATGTTAAATCCGGGTCAGGGAAGAGGACCCGATTCGGGAGTCGCTGGTGGGTCGTCGAACTCCGACCCGTTTCCTGCGGGTCTTCGAGTTCTCGTGGTCGACGATGACCCAACTTGTCTGATGATCTTGGAGAGGATGCTCAGGACTTGTCTCTACAGAGTAACGAAGTGTAACAGAGCAGAGATCGCATTGTCTCTGCTACGAAAGAACAAGAACGGTTTCGATATTGTCATCAGTGATGTTCATATGCCTGACATGGACGGATTCAAGCTCCTAGAACACGTCGGTCTAGAGATGGATCTACCTGTTATCATGATGTCTGCGGATGATTCAAAGGCCGTTGTGTTGAAAGGAGTGACTCACGGTGCAGTGGACTACCTCATCAAACCAGTACGCATCGAGGCGCTCAAGAACATATGGCAGCATGTGGTGAGGAAGAAGCGTAACGAGTGGAATGTCTCTGAACATTCCGGAAGTGTTGAAGAGACTGGGCAGAGGGAAGATGGTGATAACAACTCTTCTTCAGCTAATAACGAAGGGAGCTGGAGGGGCTCGAGGAAGAGGAAGGAAGAGGAAGTAGACGAGCAAGGGGGGGATGATAAAGAGGACACGTCTAGCTTGAAGAAACCACGTGTGGTTTGGTCTGTTGAGTTGCATCAACAATTTGTCGCTGCTGTGAATCAGCTTGGCGTTGACAAAGCGGTTCCGAAGAAGATCTTGGAGATGATGAATGTACCAGGACTAACGAGAGAAAACGTAGCTAGTCACCTCCAGAAGTATAGGATATATCTAAGACGGCTTGGAGGAGTGTCTCAGCACCAAGGGAACATGAACCATTCGTTTATGACAGGTCAAGATCCGAGTTTCGGTCCTCTTTCTACGTTGAATGGGTTTGATCTTCAAGCTCTAGCTGCTGCTGGTCAGCTCCCAGCTCAGAGCCTCGCACATCTTCAAGCAGCTGGTCTTGCTCGGCCTCCTTCACTCACTAAACCGGGGATGTCCGTAGATCAGAGAAGCATCTTCAGCTTCGAAAACCCAAAAATAAGACATGGGCAGATGATGAACAGTGGTGGTGGTGGTAATAAGCAGATGAATCTGCTTCACGGTGTTCCAATGGGAATGGAACCAAGACAGTTTACAGGTGGTGGTCAAATGCGCGTGCAGCAGCAGCAGCAACAATTGTCTGGTGGTCGTGCTGTTGGACAGAATGTTCAGAGCAGTGGGATGATGATGCCAGTAGGTGGAGGGCCATCAATGCTACAACAGCAACAACAAGTGATGTTGTCGAGTAGTGTTCCAAGGAGAAGCGAGACAAGCAGCAGCAGTAGAGTGTTACCAGCTGCTGCTACTACACAGTCGGTGGTCTTTAATAACTTTTCCTCGGAGCTACCTAGAAACAGCTTCCCGTTGGCAAGTGCACCTGGGATATCAGTTTCTTACCAAGAAGAAGTCAACAGCTCAGACGCAAAAGGTGGTGCTGGGTTTGGTAACCCGAGCTACGACATATTCAATGATTATCCGCAGCAGCACAACAACAACAATGATTGGGATCTGCAGAATATCGGCATGGTCTTTAATTCTCATCAGGACACAACAACAGCATCTGCCGCTTTTTCATCTTCGTCCTCCACTCAGAGACAAAGGGCTGAGCATGTGCAGAACCATCACCAGCAACAGCAGTTGCCAAGCCAGAGTCGTAATCACATGAACGGCGGTGGTTCGGTTAGAGTGAAGTCAGAGAGAGTGGCGGAGACTGTGACTTGTCCTCCAGCAACAACACTGTTTCAAGAGCAGTATAATCAAGAAGATCTCATGAGCGCACTTCTCAAACAGGAAGGACTTCCATTGGTAGATAACGAGTTCGACTTTGACGGATACTCCTTCGATAATATTCCTGTCTGA

>BrRR26(Bra012743)-DNA

ATGCTAACTCCGGGTGCAGTAGGTGGGTCGTCGAACTCGGACCCGTTTCCATCGGGTCTACGAGTCCTGGTCGTGGACGATGACCCAACTTGTCTCATGATCTTAGAGAGGATGCTTAAGACTTGTCTATACAGAGGTGAGTTCAAAATTCTGTTTTTTTTTTTTACATTTTAATCTCAAAGTATTCAGAGGTTCATCATGGTCGTTCTTCAACTGAATTCTTGGTCAAATTTGCGAAATTTTATTGGTAACTTTTTTTCTTGTGTGGATTTTAATACGGTGAATTGACTTAAGCCAAATATAGAAGACATATCTTTGAAATTGACTCTTTGAAATTGACTATAAACTTTCACCAAAACACTATTTGAATTGACTAGAAATATTTACCAAAGTAACTGTTAGATTCAGAGTATATATATATATATGCATAAGCTCAGTGCTCTGTTTCCAATCAGTTTTTGATCTGTTTATTTTTCAAGAGATCCTTCTGATTGATTTAGTTTCAGAAACTTAGTTTGGTTCTTGGTATATAATATATTTTATGATATTGTTATTTTTTTTGTCAACTATGATATTGTTATTTTGATTATTATTTTTTTGTGTTTGATATTTAACTAAAGATTTACTCTGTTTCTGTTTTTATTACAGTAACAAAGTGTAACAGAGCAGAGATCGCATTGTCTCTGCTTCGAAAGAACAAGAACGGTTTCGATATTGTCATCAGCGACGTCCACATGCCTGACATGAACGGCTTCAAGCTCCTCGAACACGTCGGTCTAGAGATGGACTTACCTGTCATCAGTAAACGTTTTAACAAGCCTCCCCTAAACTAAAAACATCTCTGCTTCTCTTCAAGATCTGATCTCGTTTTTTAATATGTTTTTAGTGATGTCTGCGGACGATTCAAAGAGCGTTGTGTTGAAAGGAGTGACCCACGGTGCAGTCGACTACCTAATCAAACCCGTACGCATCGAGGCTCTCAAGAATATATGGCAGCATGTGGTGCGGAAGAAGCAGAACGTCTCCGAACATTCTGGAAGTGTTGAAGAGACGGGTGGAGACAGGCAGCAGCAGAGGGATGATGATGATGATGATGGTGGTGATAACAACAACTCGTCTTCAGGTAATAATGAAGGGAACTTGAGGAAGCGGAAGGAAGAGGAGCAAGGGGGGGATGATAAAGAGGACACTTCGAGTTTGAAGAAACCACGTGTGGTTTGGTCTGTTGAGTTGCATCAACAGTTTGTGGCTGCTGTGAATCATCTCGGCGTTGACAGTAAGTAAACAAAAAAAAAAACACACACTTGTTTGGTTATGCATTTGTGTTTTTTTTTCTGGTCTAATCTTTCATTCAATGCAGAAGCTGTTCCTAAGAAGATCTTGGAGATGATGAATGTACAGGGGCTAACGAGGGAAAACGTAGCCAGTCACCTCCAGGTAAAATATATTCCCTCTGGATTTAAAAGATCATGTTTAGGATTTACAATTATTAGTAAAAATTTAGCACATACTTTGATTTATTAGTTTTTGTTACTTGTTTGCTTCCCATTTAAGTTCTACCACTTATACTTTGAAGTTTAATTTATGTTAATTTTAAATTATAATGCATTAATTAAAGTTAGAACATCAATTTTTTATATACAATGAAAAACTAAAACATCAAACATTTGTATACAGAGGGAGTATATGAGTGAATAGTACTATCTATGTTTCAAAATAATGTATGTTTTAGATTTTTCCGCACATATTATTAAAACATTTTAAATTTTAGTATTAAATGTATGATTTTTTTGTAATGATATATTTCTTATAATTACAAACTAAAAAGAATTCAGTAAATGTAATTATTTTTGTTTTTAAATTTACAATCATTCATTATTTTTGATAGAAATGTATGGAAAATATTTTTTTAAAAATGTCTTTGTGAAACAATTTTTTTTTCAAACATGTATTTTATGAAACAGAATGAGTATTACATTTTTATCGCATGCATGCTAACATGTAAGTTGGTGACATTGTGGAAATGCAGAAGTATAGGATATATCTAAAAAGGCTTGGAGGAGTATCTCAGCACCAAGGAAACATAAACCATTCGTTTATGACTGGTCAAGATCCAAGCTACGGTCCGTTGAATGGGTTTGATCTTCAAGGTCTAGCTACTGCGGGTCAGCTCCAAGCTCAGAGCCTCGCACAGCTTCAAGCAGTTGGCCTTGGTCAATCTTCTTCGCCACTCATTAAACCGGGGATAACGTCAGTAGATCAGAGAAGCTTCTTCACCTTCCAAAACTCGAAATCGAGATTCGGAGATGGACATGGGCCGATGATGATGAACGGTGGTAATAAGCAGACAAGTTTGCTTCACGGTGTCCCAACGGGTCACATGCGCTTGCAGCAGCAACAAATGGCTGGTATGCGTGTAGCGGGGCCATCAATGCAGCAGCAGCAACAATCGATGTTGTCAAGAAGAAGTGTCCCGGAGACCAGAAGCAGCAGAGTGTTACCAGGTGCTACACACTCGGCCTTCAACAACAGCTTCCCGTTGGCAAGTGCACCGGGGATGATGTCTGTTTCAGACACAAAAGGCGTGAACGAGTTTTGTAACCCGAGCTACGACATATTGAACAACTTTCCCCAGCAGCAACACCACAACAACAACAACAGCGTGAACGAGTGGGATCTGCGGAATGTGGGGATGGTCTTCAACTCTCATCAGGACAACACAACATCAGCTGCTTTTTCAACTTCAGAAGCTTACTCTTCGTCTTCAACTCATAAAAGAAAGCGGGAGGCAGAGTTAGTGGTTGAGCATGGGCAGAATCAGCAGCAGCCACAAAGCCGAAGTGTGAATCCTATGAACCAGATTTACATGAATGATGGTGGTTCGGTTAGAATGAAGACAGAGACGGTGACTTGTCCTCCTCAGGCAACAACAATGTTTCACGAGCAGTATAGTAATCAAGATGATCTCCTGAGCGCACTTCTAAAGCAGGTTTCATTTTCTATACTATTATGACATGACATGACATTGATATGAATATTGTTGTCTTGTGGTTATAACTTTTAACTCCATTCTCCGTTTTATCAAAATGCAGGAAGGATTGTTAGATACCGAGTTTGATTTTGAAGGATACTCCTTCGATAATATTCTCGTCTGA

>BrRR26(Bra012743)-CDS

ATGCTAACTCCGGGTGCAGTAGGTGGGTCGTCGAACTCGGACCCGTTTCCATCGGGTCTACGAGTCCTGGTCGTGGACGATGACCCAACTTGTCTCATGATCTTAGAGAGGATGCTTAAGACTTGTCTATACAGAGTAACAAAGTGTAACAGAGCAGAGATCGCATTGTCTCTGCTTCGAAAGAACAAGAACGGTTTCGATATTGTCATCAGCGACGTCCACATGCCTGACATGAACGGCTTCAAGCTCCTCGAACACGTCGGTCTAGAGATGGACTTACCTGTCATCATGATGTCTGCGGACGATTCAAAGAGCGTTGTGTTGAAAGGAGTGACCCACGGTGCAGTCGACTACCTAATCAAACCCGTACGCATCGAGGCTCTCAAGAATATATGGCAGCATGTGGTGCGGAAGAAGCAGAACGTCTCCGAACATTCTGGAAGTGTTGAAGAGACGGGTGGAGACAGGCAGCAGCAGAGGGATGATGATGATGATGATGGTGGTGATAACAACAACTCGTCTTCAGGTAATAATGAAGGGAACTTGAGGAAGCGGAAGGAAGAGGAGCAAGGGGGGGATGATAAAGAGGACACTTCGAGTTTGAAGAAACCACGTGTGGTTTGGTCTGTTGAGTTGCATCAACAGTTTGTGGCTGCTGTGAATCATCTCGGCGTTGACAAAGCTGTTCCTAAGAAGATCTTGGAGATGATGAATGTACAGGGGCTAACGAGGGAAAACGTAGCCAGTCACCTCCAGAAGTATAGGATATATCTAAAAAGGCTTGGAGGAGTATCTCAGCACCAAGGAAACATAAACCATTCGTTTATGACTGGTCAAGATCCAAGCTACGGTCCGTTGAATGGGTTTGATCTTCAAGGTCTAGCTACTGCGGGTCAGCTCCAAGCTCAGAGCCTCGCACAGCTTCAAGCAGTTGGCCTTGGTCAATCTTCTTCGCCACTCATTAAACCGGGGATAACGTCAGTAGATCAGAGAAGCTTCTTCACCTTCCAAAACTCGAAATCGAGATTCGGAGATGGACATGGGCCGATGATGATGAACGGTGGTAATAAGCAGACAAGTTTGCTTCACGGTGTCCCAACGGGTCACATGCGCTTGCAGCAGCAACAAATGGCTGGTATGCGTGTAGCGGGGCCATCAATGCAGCAGCAGCAACAATCGATGTTGTCAAGAAGAAGTGTCCCGGAGACCAGAAGCAGCAGAGTGTTACCAGGTGCTACACACTCGGCCTTCAACAACAGCTTCCCGTTGGCAAGTGCACCGGGGATGATGTCTGTTTCAGACACAAAAGGCGTGAACGAGTTTTGTAACCCGAGCTACGACATATTGAACAACTTTCCCCAGCAGCAACACCACAACAACAACAACAGCGTGAACGAGTGGGATCTGCGGAATGTGGGGATGGTCTTCAACTCTCATCAGGACAACACAACATCAGCTGCTTTTTCAACTTCAGAAGCTTACTCTTCGTCTTCAACTCATAAAAGAAAGCGGGAGGCAGAGTTAGTGGTTGAGCATGGGCAGAATCAGCAGCAGCCACAAAGCCGAAGTGTGAATCCTATGAACCAGATTTACATGAATGATGGTGGTTCGGTTAGAATGAAGACAGAGACGGTGACTTGTCCTCCTCAGGCAACAACAATGTTTCACGAGCAGTATAGTAATCAAGATGATCTCCTGAGCGCACTTCTAAAGCAGGAAGGATTGTTAGATACCGAGTTTGATTTTGAAGGATACTCCTTCGATAATATTCTCGTCTGA

>BrRR27(Bra023972)-DNA

ATGACATTGGAACAAGATTTTGAAGCAGTGGACCAGTTTCCAGTGGGGATGAGAGTTCTTGCCGTTGACGATGACCAAACTTGTCTCCGTATTCTCGAAACTCTCCTTCACCGCTGCCAATACCATGGTCCCTTCTCTCTCTCTCTCTCTCTTAATGTTTTCACACATATTTGCTTTTGAGTAATCAAAATTAAGATCTAGATAGATTCATTATTTGAAGTTGTATTAATTCTCTGCATATATGCTACTATAAAAATCAAAGTTTCAGACTTTAAATCTTTTGACTTGGTTTATTTAAAAAAAAACAGAGTTTTTTTGGGGAAATTATTGCAAAGCTAATTACTTTGGGGTAATTAATTATATGTGCAGTTACAACAACGGACAGTGCGCAGACCGCACTGGAGCTGTTGAGGGAGAACAAGAACAAGTTTGATCTCGTTATTAGCGATGTCGACATGCCAGACATGGACGGTTTCAAGCTGCTTGAGCTCGTTGGTCTTGAAATGGACTTACCTGTCATAAGTAAGCACAATCCATTCTCTCTTGTGTTTAGTTTCTGTATTGATCTTTGTGACGTGATGATTTGATTATTATTTTGCAGTGTTATCTGCGCATAGCGATCCGAAGTATGTGATGAAAGGAGTCAAGCACGGTGCCTGCGACTATCTACTTAAACCGGTGCGTATTGAGGAGCTCAAGAACATATGGCAACACGTGGTGAGGAAAAGCAAGTTCAAGAAGATGAAGAGCATTGTGATTAATGATGATCATTCCCAAGGAAACTCTGATCAGAACGGTGTGAAAGCGAATAGAAAACGTAAAGATCAGTTTGAAGAGGTGGAGGAAGAAGATGAAGAAAGAGGGAATGAGAACGATGATCCAACGGCTCAGAAGAAGCCACGTGTTCTCTGGACTCGCGAGCTGCACAATAAGTTCTTAGCAGCTGTTGATCATTTGGGAGTTGAGAGTAAATGATTCTTTGATCTCATCTCTTAGTTAGTTTCTTTTGCGTAGTAATTTTTTTTCTGATTGATAAGTTGAAATGATGCAGAAGCTCAACCGAAAAAGATTCTTGAACTGATGAATGTTGATAAGCTCACAAGAGAGAATGTTGCTAGCCACCTTCAGGTGTGTGTGTGAGGCCATTATTCTTTATAGGATCCCTGAAATTTTAGAGGCTATAGATATTTTTTAAAAAAATTTCATTAAAATTTGTTTTCATGATTTGAAGGCTTATGGCTATATAAAAAATGTTTAAAATTTTTGGAGGCTAAGGCCGATGTTTTGTAAGGTTGTGCTCAGATCCGGCCATGTTGGTTAATAAGGTTTCCACTAACATAACTACAATGTCTTCTGGTTGCAGAAGTTCCGCTCTGCGTTGAAGAAAATAACAAATGAAGCTAATCAACAAGCTAACATGGCGGCTATAGACTCACACTTCATGCAAATGAGTGCTCTCAAAGGGCTTGGCGGTTTCCACAACCAACGGCAGATACCTCTTGGATCAGGTCAGTTCCATGGTGGAGCTGCCACCATGAGGCATTATCCTCTTGGTCGCCTAAACTCCTTTGGAGGAGTGTTCCCACATGTGTCATCGTCGCTTCCTCGTAACCACAATGATGGAGGTTATGTACTTCAGGGAATGCCAATTCCACCATTAGATGATCTTAACAACAAGGCTTTTCCGAGCTTTACTTCACAACAAAGCTCTCTAATGGTTGCTCCCAATAATCAGTTGGTTCTCCAGGGTCACCAGCAGTCATCATATCCATCCTTGAACCCAGGGTTGTCTCCCCATTTCGAGATCAACAAGCGTCTTGATGATTGGTCAAACGCTTTATTGTCAACCAACATTCCACAGAGTGGTGTTCATTCAAAACCAGACGCCTTGGAATGGAACCACTTCTGCAACTCAGATGCTGCACAAGCAGGCTTTATTGATCCATTACAGATGAAGCAGCAGCCTGCGAACAACTTAGGTCCAATGACTGATGCTCAACTATTGAGAAGTAGCAATCCAATTGAAGGTTTATTTGTGGGACAACAGAAGCTAGAGAATGGTTCAATGCCTTCAAATGCTGGTTCCTTGGATGATATTGTCAACTCCATGATGCCAAAGGTATAAACAAACCATTTGCTTTGCCCATTTTTGCACTAGTAGATTTAATGATTATTTACTTCTTTTTTTTTTGGAAAGAATGCTAAATTTATTCAAAATGATTATTTACTTTCAATGTAACAGGAACAGAGCCAAGCTGAGTTATTTGAAGGAGATTTGGGGTTTGGATGGCATAATAGCTCACTCAGAACATGCATATGA

>BrRR27(Bra023972)-CDS

ATGACATTGGAACAAGATTTTGAAGCAGTGGACCAGTTTCCAGTGGGGATGAGAGTTCTTGCCGTTGACGATGACCAAACTTGTCTCCGTATTCTCGAAACTCTCCTTCACCGCTGCCAATACCATGTTACAACAACGGACAGTGCGCAGACCGCACTGGAGCTGTTGAGGGAGAACAAGAACAAGTTTGATCTCGTTATTAGCGATGTCGACATGCCAGACATGGACGGTTTCAAGCTGCTTGAGCTCGTTGGTCTTGAAATGGACTTACCTGTCATAATGTTATCTGCGCATAGCGATCCGAAGTATGTGATGAAAGGAGTCAAGCACGGTGCCTGCGACTATCTACTTAAACCGGTGCGTATTGAGGAGCTCAAGAACATATGGCAACACGTGGTGAGGAAAAGCAAGTTCAAGAAGATGAAGAGCATTGTGATTAATGATGATCATTCCCAAGGAAACTCTGATCAGAACGGTGTGAAAGCGAATAGAAAACGTAAAGATCAGTTTGAAGAGGTGGAGGAAGAAGATGAAGAAAGAGGGAATGAGAACGATGATCCAACGGCTCAGAAGAAGCCACGTGTTCTCTGGACTCGCGAGCTGCACAATAAGTTCTTAGCAGCTGTTGATCATTTGGGAGTTGAGAAAGCTCAACCGAAAAAGATTCTTGAACTGATGAATGTTGATAAGCTCACAAGAGAGAATGTTGCTAGCCACCTTCAGAAGTTCCGCTCTGCGTTGAAGAAAATAACAAATGAAGCTAATCAACAAGCTAACATGGCGGCTATAGACTCACACTTCATGCAAATGAGTGCTCTCAAAGGGCTTGGCGGTTTCCACAACCAACGGCAGATACCTCTTGGATCAGGTCAGTTCCATGGTGGAGCTGCCACCATGAGGCATTATCCTCTTGGTCGCCTAAACTCCTTTGGAGGAGTGTTCCCACATGTGTCATCGTCGCTTCCTCGTAACCACAATGATGGAGGTTATGTACTTCAGGGAATGCCAATTCCACCATTAGATGATCTTAACAACAAGGCTTTTCCGAGCTTTACTTCACAACAAAGCTCTCTAATGGTTGCTCCCAATAATCAGTTGGTTCTCCAGGGTCACCAGCAGTCATCATATCCATCCTTGAACCCAGGGTTGTCTCCCCATTTCGAGATCAACAAGCGTCTTGATGATTGGTCAAACGCTTTATTGTCAACCAACATTCCACAGAGTGGTGTTCATTCAAAACCAGACGCCTTGGAATGGAACCACTTCTGCAACTCAGATGCTGCACAAGCAGGCTTTATTGATCCATTACAGATGAAGCAGCAGCCTGCGAACAACTTAGGTCCAATGACTGATGCTCAACTATTGAGAAGTAGCAATCCAATTGAAGGTTTATTTGTGGGACAACAGAAGCTAGAGAATGGTTCAATGCCTTCAAATGCTGGTTCCTTGGATGATATTGTCAACTCCATGATGCCAAAGGAACAGAGCCAAGCTGAGTTATTTGAAGGAGATTTGGGGTTTGGATGGCATAATAGCTCACTCAGAACATGCATATGA

>BrRR28(Bra004245)-DNA

ATGGATAAAGGCTTCTCTCCCGTCGGTCTAAGAGTTCTTGTGGTTGACGACGACCCAACATGGCTCAAGATTCTTGAGAAGATGCTCAAGAAGTGCTCTTACGAAGGTCTCTATCTCATGTTTTAGACTCACTCCTCAACACCACCTGAGATGTCTCTCCTTAACTTAATTTCCTCTGCTTTTGAATTAACTCTGTTGCAGTGACTACATGTGGGTTAGCTAGAGAAGCTCTGAGACTGCTCCGGGAGCGTAAGGACGGATATGACATCGTGATCAGCGACGTGAACATGCCTGACATGGACGGCTTCAAGCTCCTTGAGCACGTTGGACTCGAGCTGGACCTCCCTGTCATAAGTCAGACTTCTCCACAAGCACAAATCAAGATCTTGTCTGATAAATAGTTACTAACTTTGATGTTTTTTTGTTACAGTGATGTCAGTGGACGGTGAGACGAAAAGAGTGATGAAAGGCGTCCAGCACGGAGCGTGTGATTACCTGTTGAAGCCCATAAGGATGAAGGAGCTGAAGATCATATGGCAGCACGTTCTGAGAAAGAAGCTTCAAGAAGTGAGGGACATCGAAGGATGCTATGAAGGAGGAGCTGATTGGTTCACAAGAAACGATGAGGCACACTTGCTTGGAGGAGGAGGTGAAGATGTTTCTTTTGGGAGAAAGAGGAAAGAGTTTGACTTTGAGAAGAAGCTTCTTCTTCAGGATGAGAGTGACCCATCATCGTCTTCCTCCAAGAAAGCAAGGGTGGTTTGGTCTTTTGAGCTTCATCAGAAGTTTGTCAACGCTGTTAACCAGATCGGTTGCGATCACAGTAAGTACGTTGGGACACTTCAAGTTTCTTTCCCCTTGTCTTTTTCATAGAGACGTGATTTTTAGGGTCTCTTGTGTTTGGCAGAGGCTGGTCCCAAGAAGATATTGGACCTCATGAATGTTCCATGGCTCACTAGGGAAAATGTTGCGAGCCACCTCCAGGTAAAACTTTACCTTCTCGTTAAATCAAAGGGGGAAGTGAAGTGGAGATTAAGCTTTTTGTCACTTTCTGTTTTTTTGTAGAAGTACAGGCTTTACCTGAGCAGATTGGAGAAAGGAAAGGAGCTAAAGTGTTATTCTGGTGGAGTAAAGAATATGGATTCACCTCCAAAAGATTCCGAGTTTAATACAGGCCACCAAAGCCCTGGCAAGAACAGCTATGCCTTCTCTGGAGGCAGTTCTGATCCAAAGCAACTTGCTTCATCTTCTGTGTCTGACCCCAGCAGTGATGTTCATATGCCTCCAAAAGCGAAAAAGACGCGTGTAGAGTTTGATCCTCCCATTTCCTCAAGTTCTGCGTTTGAGTCTCTGCTTCCCTGGAGTGATGTTCCAGACCCACTTGAATCAAAGCCCCAGATTCTGTATGGGAGCAGCTTTCTCCAGCAACAACCATTACCAAGTCAAAGTCCCTATGTTGCAAATTCTGCACCAACTCTGATGGAACAGGAAATGAAGCCTTCGTATGAGACTTCTGTGAATGCGGATGAGTTTCTCATGCCACAAGACAAGAACTCTACCGTGATCCTTCAAGATCTGGACTTGTCCGCTCCCTCTGCCATCTCCAGCATAAATGTAACCAACGATACAGAGTCGATTCTGAGAAGTTTGAGCTGGGAGCTTCCGGAATCACATCATTCTGGTTTTATAGACACTGACTTAGACTTCAGTTGGCTTCAAAACGAGCATTTTCTTGCAAACACCTCCGGGAACTTCCAGTTTCAAGACTACAGTTGTAGCCCATCTCTCCTTTCCGAGCTCCCACCTCACCTTTGGTTTGGAAATGAACCTGACGAGTATACCCTCATGGTAGACCATGGCTTATTCATATCTTGA

>BrRR28(Bra004245)-CDS

ATGGATAAAGGCTTCTCTCCCGTCGGTCTAAGAGTTCTTGTGGTTGACGACGACCCAACATGGCTCAAGATTCTTGAGAAGATGCTCAAGAAGTGCTCTTACGAAGTGACTACATGTGGGTTAGCTAGAGAAGCTCTGAGACTGCTCCGGGAGCGTAAGGACGGATATGACATCGTGATCAGCGACGTGAACATGCCTGACATGGACGGCTTCAAGCTCCTTGAGCACGTTGGACTCGAGCTGGACCTCCCTGTCATAATGATGTCAGTGGACGGTGAGACGAAAAGAGTGATGAAAGGCGTCCAGCACGGAGCGTGTGATTACCTGTTGAAGCCCATAAGGATGAAGGAGCTGAAGATCATATGGCAGCACGTTCTGAGAAAGAAGCTTCAAGAAGTGAGGGACATCGAAGGATGCTATGAAGGAGGAGCTGATTGGTTCACAAGAAACGATGAGGCACACTTGCTTGGAGGAGGAGGTGAAGATGTTTCTTTTGGGAGAAAGAGGAAAGAGTTTGACTTTGAGAAGAAGCTTCTTCTTCAGGATGAGAGTGACCCATCATCGTCTTCCTCCAAGAAAGCAAGGGTGGTTTGGTCTTTTGAGCTTCATCAGAAGTTTGTCAACGCTGTTAACCAGATCGGTTGCGATCACAAGGCTGGTCCCAAGAAGATATTGGACCTCATGAATGTTCCATGGCTCACTAGGGAAAATGTTGCGAGCCACCTCCAGAAGTACAGGCTTTACCTGAGCAGATTGGAGAAAGGAAAGGAGCTAAAGTGTTATTCTGGTGGAGTAAAGAATATGGATTCACCTCCAAAAGATTCCGAGTTTAATACAGGCCACCAAAGCCCTGGCAAGAACAGCTATGCCTTCTCTGGAGGCAGTTCTGATCCAAAGCAACTTGCTTCATCTTCTGTGTCTGACCCCAGCAGTGATGTTCATATGCCTCCAAAAGCGAAAAAGACGCGTGTAGAGTTTGATCCTCCCATTTCCTCAAGTTCTGCGTTTGAGTCTCTGCTTCCCTGGAGTGATGTTCCAGACCCACTTGAATCAAAGCCCCAGATTCTGTATGGGAGCAGCTTTCTCCAGCAACAACCATTACCAAGTCAAAGTCCCTATGTTGCAAATTCTGCACCAACTCTGATGGAACAGGAAATGAAGCCTTCGTATGAGACTTCTGTGAATGCGGATGAGTTTCTCATGCCACAAGACAAGAACTCTACCGTGATCCTTCAAGATCTGGACTTGTCCGCTCCCTCTGCCATCTCCAGCATAAATGTAACCAACGATACAGAGTCGATTCTGAGAAGTTTGAGCTGGGAGCTTCCGGAATCACATCATTCTGGTTTTATAGACACTGACTTAGACTTCAGTTGGCTTCAAAACGAGCATTTTCTTGCAAACACCTCCGGGAACTTCCAGTTTCAAGACTACAGTTGTAGCCCATCTCTCCTTTCCGAGCTCCCACCTCACCTTTGGTTTGGAAATGAACCTGACGAGTATACCCTCATGGTAGACCATGGCTTATTCATATCTTGA

>BrRR29(Bra004076)-DNA

ATGGAGAAAGGCTTCTCTCCCGTCGGTCTCAGGGTTCTTGTGGTTGACGACGACCCAACCTGGCTCAAGATTCTTGAGAAAATGCTCAAGAAGTGCTCCTACGAAGGTTCCCTTCTTCTTTTTCTATGTTTTTACACATTGGGAGTCCACCTGCTTAATTTCCTCTGCTTTTGTTTTAACCCCGTTGCAGTCACTACCTGTGGGTTAGCTAGAGAAGCTCTGAGGTTGCTACGGGAACGTAAGGACGGGTTTGACATCGTGATCAGCGACGTGAACATGCCTGACATGGACGGCTTCAAGCTCCTTGAGCACGTTGGCCTTGAACTGGACCTCCCTGTCATAAGTGAGACTTCTATATATGATCTTGAGTAATAAAATAAAAAAACAGAAAAGGTTATTATTAATTTTGTTGTTTGCTTTTACAGTGATGTCGGTGGACGGTGAAACAAGCAGAGTCATGAAAGGCGTCCAGCATGGAGCGTGTGACTACCTGCTGAAGCCGATAAGAATGAAGGAGCTGAAGATCATATGGCAACATGTTCTTAGAAAGAAGCTTCAAGAAGTGAGGGACATCGAAGGCTGCTGCTATGACGGAGGAGCTGACTGGTTCACCCAAGGGCAGTTTCTTGGAGGTGGTGAAGATGTTTCTTTTGGGAAGAAGAGAAAAGACTTTGACTTTGAGAAGAAGCTTTTTCAGGATGAGAGTGACCAATCTTCTTCTTCCAAGAAAGCTAGAGTGGTTTGGTCTTATGAGCTTCACCAGAAGTTTGTTAACGCTGTTAACCAGATCGGATGTGATCACAGTAAGTACCTCCCTTTAGCATGTCTTCTTCATAGAGCAGTGATTTTTTCTGGTCTCTTTGTGTATGGCAGAAGCTGGTCCCAAGAAGATATTGGACCTCATGAATATCCCATGGCTCACTAGGGAGAATGTTGCTAGCCACCTTCAGGTAAAACTTTACCCTTCTCTTTATCCAAAAGAGGAAGAACACTAAGCTTTTTGTCACTTTTTCTATTTTTATTTTTATTTTTTTTTTGTAGAAATACAGACTGTACTTGAGTAGATTAGAGAAAGGAAAGGAGATAAAGTGTTATTCTGGTGGAGTAAAGAATATGGATTCACCTCCAAAAGATGCTGAGATTAATTCAGGACATCAAAGCCCTGGCAAGAGCAGCAGCTATGCATTCCTTAAAGCAACAGAGACTGATCCAAAGCAACTTGCTTCAGCTTCTGTGTCTGACCCCACCAGTGATATCCACATGCCTCAAAAAGCGAAAAAGACACGTATAGGATTTGATCCTCCCATCTCATCCGGTGTGTTTGGCTCTCTGCTTCCCTGGAATGATGTTCCAGACCCACTTGAATCCAAGCCTCCTATTCTCTATGAGAACAGCTTTCTCCAGCAACAACCATTGCCAAGCCAAAGCTCCTATGTTGCAAACTCTGCACCGTCTCTCATGCAAGAGGAAATGAAGCCTTCTTATGTGAATCCGGATGAGTTTCTCATGCCACAAAACAAGAACTCTACTGTGATCCTTCAAGATATGGACTTGTCCGCTCCCTTCAGCTCCAATGCAACCAGCAATACAGAGTCGATTCCGGGAAGCTTGAACTGGGAACTTCCAGAAGCACATCATTCAGGTTCTTTAGACACTGACTTAGACTTCACTTGGCTTCATGGAGAGCATTTCTTTGCAAACAGCGGACTCCAAAACTTCCAGTTTCAAGACTACAGTAACAGTAGTAGCACATCACTCCTGTCTGAGCTCCCTCCCCATCTTTGGTATGGAAACGACAGGCTGCCCGACCCTGACGAGTATACCCTAATGGTAGACCAAGGTTTATTCATATCTTGA

>BrRR29(Bra004076)-CDS

ATGGAGAAAGGCTTCTCTCCCGTCGGTCTCAGGGTTCTTGTGGTTGACGACGACCCAACCTGGCTCAAGATTCTTGAGAAAATGCTCAAGAAGTGCTCCTACGAAGTCACTACCTGTGGGTTAGCTAGAGAAGCTCTGAGGTTGCTACGGGAACGTAAGGACGGGTTTGACATCGTGATCAGCGACGTGAACATGCCTGACATGGACGGCTTCAAGCTCCTTGAGCACGTTGGCCTTGAACTGGACCTCCCTGTCATAATGATGTCGGTGGACGGTGAAACAAGCAGAGTCATGAAAGGCGTCCAGCATGGAGCGTGTGACTACCTGCTGAAGCCGATAAGAATGAAGGAGCTGAAGATCATATGGCAACATGTTCTTAGAAAGAAGCTTCAAGAAGTGAGGGACATCGAAGGCTGCTGCTATGACGGAGGAGCTGACTGGTTCACCCAAGGGCAGTTTCTTGGAGGTGGTGAAGATGTTTCTTTTGGGAAGAAGAGAAAAGACTTTGACTTTGAGAAGAAGCTTTTTCAGGATGAGAGTGACCAATCTTCTTCTTCCAAGAAAGCTAGAGTGGTTTGGTCTTATGAGCTTCACCAGAAGTTTGTTAACGCTGTTAACCAGATCGGATGTGATCACAAAGCTGGTCCCAAGAAGATATTGGACCTCATGAATATCCCATGGCTCACTAGGGAGAATGTTGCTAGCCACCTTCAGAAATACAGACTGTACTTGAGTAGATTAGAGAAAGGAAAGGAGATAAAGTGTTATTCTGGTGGAGTAAAGAATATGGATTCACCTCCAAAAGATGCTGAGATTAATTCAGGACATCAAAGCCCTGGCAAGAGCAGCAGCTATGCATTCCTTAAAGCAACAGAGACTGATCCAAAGCAACTTGCTTCAGCTTCTGTGTCTGACCCCACCAGTGATATCCACATGCCTCAAAAAGCGAAAAAGACACGTATAGGATTTGATCCTCCCATCTCATCCGGTGTGTTTGGCTCTCTGCTTCCCTGGAATGATGTTCCAGACCCACTTGAATCCAAGCCTCCTATTCTCTATGAGAACAGCTTTCTCCAGCAACAACCATTGCCAAGCCAAAGCTCCTATGTTGCAAACTCTGCACCGTCTCTCATGCAAGAGGAAATGAAGCCTTCTTATGTGAATCCGGATGAGTTTCTCATGCCACAAAACAAGAACTCTACTGTGATCCTTCAAGATATGGACTTGTCCGCTCCCTTCAGCTCCAATGCAACCAGCAATACAGAGTCGATTCCGGGAAGCTTGAACTGGGAACTTCCAGAAGCACATCATTCAGGTTCTTTAGACACTGACTTAGACTTCACTTGGCTTCATGGAGAGCATTTCTTTGCAAACAGCGGACTCCAAAACTTCCAGTTTCAAGACTACAGTAACAGTAGTAGCACATCACTCCTGTCTGAGCTCCCTCCCCATCTTTGGTATGGAAACGACAGGCTGCCCGACCCTGACGAGTATACCCTAATGGTAGACCAAGGTTTATTCATATCTTGA

>BrRR30(Bra032035)-DNA

ATGACTGTTGAACAACAAGATTGTGTAGCCTTGGACCAGTTTCCTGTCGGAATGAGAGTTCTTGCTGTTGACGATGACCAGACTTGTCTTCGTATCCTCGAATCCTTGCTTCATCGTTGCCAATATCATGGTTCCTTTCTCTATCTCTTCTCTCCTTTCTTGCATCGACCATTTCGTGTTTTTGGATCTGTTGATGAGTTTCTCCAAATGGGCTTTTAGATTTAGTTCTATTTATTTTCTTGATCTGAGAGTTTTTTTTAATCACTAATGGAAACTTTTAAGACAAAAGGATTCAAATACCTACCTACCTAACTTCATGTAGAAATCCTAGTTTCTGATTGGATCTTTCCAACATCATAGATCGATTTTGAAGATTGACAATTTTTTCATATACCATAAAAAAATCACATTTTATTTGCTTTGGATCCACACAACTGTAATGATTACATGTGTCACAAGAGCTTATTGTTGAATTCAATTTAAATTAATAAAAACATAAACTTTATATGGCTGCAGTTACAACGACGAACCAGGCCCAAAAGGCTCTAGAGTTATTGAGAGAGAACAAGAACAAGTTTGATCTGGTTATTAGCGATGTTGACATGCCTGACATGGATGGTTTCAAACTGCTTGAGCTTGTTGGTCTTGAAATGGACCTACCTGTCATTAGTAAGTCAACTAATTTTTTTCCATTATTTTTAATCAAATAATGTTTCATATTTTTCTTGATTCTTGGTTTAGTTTTATGATGGTTTTCTTGTTTTTTTTTAACTAAACTCTGCAGTGTTGTCCGCGCATAGCGATCCAAAGTATGTGATGAAAGGAGTTACTCATGGGGCTTGTGACTATCTACTGAAACCGGTTCGTATCGAGGAGCTGAAAAACATATGGCAACATGTGGTGAGAAAGAACCGTGGGAGTAATAACGGTGACAAGAAAGATGGATCGGGTAATGAAGGTGTTGCAAACTCCGATCAGAACAACGGGAGAGCAAATAGAAAACGTAAAGATCAGTACAATGAAGATGAAGACGAGGAAAGAGATGATAACGATGATCCGTCATCTCAAAAGAAGCCTCGTGTTGTTTGGACGGTTGAGCTTCACAAGAAATTTGTAGCAGCTGTTAACCAATTGGGGTTTGAGAGTAAGTTTTGTTTAAATGTTACATGAATTGTTGTTAGCTCAACTTGAAAAGTAGCTACGATAAAGTCTAGTGGTCAGCAGGCTAGATCGAATGAGCCTGCTGGGACGAAACTAATATTACATAATGTTGTTTTGGGATTCAGAGGGATTTAATTTTAATTTTGTTTGTTTCTGATTGCAGAGGCAATGCCTAAAAAGATTCTTGATCTGATGAATGTTGAGAAGCTGACTAGAGAGAATGTGGCTAGTCATCTTCAGGTTTTAAACAATTTTTCATGTTTTCTGTTACACAAATGAAGCTTTTTTTTTTTTTATACATCTTTTAAGATTTTTATTTTTTTCTTTGTGGCTGCAGAAGTTTCGCCTGTACTTGAAGAGGATCAGTGGGAATCAACAAGCTATTATGGCCAACTCTGACTTACATTTCTTGCAAATGAGCAATGGACTTGACGGTTTTCACCACCGACCAATTCCCGTTGGAACCGGTCAGTTCCATGGTGGAGCCGCCGCTGCGGGGATGAGACCCTTCCCTCCAAATGGGATTCTTGGCCGACTCAACACTCCTTCTGGAATGAGTGGTGTCCGTAACCTTTCTTCTTCTCCTTCTTCAGGAATGTTCTTGCAAAACCCGACCGATCTTGGAAAGTTTCACCATGTCTCATCACTTCCTCTTAACCACATTGATGGAGGAAACATACTTCAAGGGTTACCAATGCCTTTAGAGTTCGACCAGCTTCAGACAAACAACAACAAGAGCATCATCGCCGGGAACTCAATGGCTTTTCCTATCTTCCCTACACAACAACAAAGCTCCCTTCCTAATAACAACAATCACTTGGTTCTAGAAGGTCACCCACAAGCACCTCCTTCAGCCTTCCCTGGTCACCAGATCAATAAACGTTTGGAACATTGGTCAAACGCTGTATCATCATCATCGTCCACTCTTCCTCCTCCTGGTCAGAACAGTAATAGCCTCATCAGTCATCAGTTCGATGCCTCCTCATCAAGCTATTCCATCCCATTCTGTGACTCTACAATTCCATTGAATCCAGCGTTGGATCATACAAATCCCCGAGCTTTCTACAGAGCCACGGACATGGATTCAAGTGCAAATGTGCAGCCTGGAGTCTATTATGATTCATTGCAGATGAGAAAATCAGGCAACTACGGTCCAACCACGGATGCTATGCTGAGTAGTAATAACCCCAAGGAAGGGTTCACCGTGGGGCAGCAGAAGTTACAGAGTGGATTCATGGGAGGAGAAGCTGGTTCTTTAGATGATATAGTCAACTCCACTATGAAACAGGTGTGAGATGATTCAGCATCTGATATAAAAGATTAGAATGATCTCTGACATGTTTGCATTTAATGTGAATGTGACTAGGAACAGAGCCAAGGAGACTTGTCGGAAGGTGATTTGGGATATGGAGGCTTTAGCTCACTTAGAACATGCATATGA

>BrRR30(Bra032035)-CDS

ATGACTGTTGAACAACAAGATTGTGTAGCCTTGGACCAGTTTCCTGTCGGAATGAGAGTTCTTGCTGTTGACGATGACCAGACTTGTCTTCGTATCCTCGAATCCTTGCTTCATCGTTGCCAATATCATGTTACAACGACGAACCAGGCCCAAAAGGCTCTAGAGTTATTGAGAGAGAACAAGAACAAGTTTGATCTGGTTATTAGCGATGTTGACATGCCTGACATGGATGGTTTCAAACTGCTTGAGCTTGTTGGTCTTGAAATGGACCTACCTGTCATTATGTTGTCCGCGCATAGCGATCCAAAGTATGTGATGAAAGGAGTTACTCATGGGGCTTGTGACTATCTACTGAAACCGGTTCGTATCGAGGAGCTGAAAAACATATGGCAACATGTGGTGAGAAAGAACCGTGGGAGTAATAACGGTGACAAGAAAGATGGATCGGGTAATGAAGGTGTTGCAAACTCCGATCAGAACAACGGGAGAGCAAATAGAAAACGTAAAGATCAGTACAATGAAGATGAAGACGAGGAAAGAGATGATAACGATGATCCGTCATCTCAAAAGAAGCCTCGTGTTGTTTGGACGGTTGAGCTTCACAAGAAATTTGTAGCAGCTGTTAACCAATTGGGGTTTGAGAAGGCAATGCCTAAAAAGATTCTTGATCTGATGAATGTTGAGAAGCTGACTAGAGAGAATGTGGCTAGTCATCTTCAGAAGTTTCGCCTGTACTTGAAGAGGATCAGTGGGAATCAACAAGCTATTATGGCCAACTCTGACTTACATTTCTTGCAAATGAGCAATGGACTTGACGGTTTTCACCACCGACCAATTCCCGTTGGAACCGGTCAGTTCCATGGTGGAGCCGCCGCTGCGGGGATGAGACCCTTCCCTCCAAATGGGATTCTTGGCCGACTCAACACTCCTTCTGGAATGAGTGGTGTCCGTAACCTTTCTTCTTCTCCTTCTTCAGGAATGTTCTTGCAAAACCCGACCGATCTTGGAAAGTTTCACCATGTCTCATCACTTCCTCTTAACCACATTGATGGAGGAAACATACTTCAAGGGTTACCAATGCCTTTAGAGTTCGACCAGCTTCAGACAAACAACAACAAGAGCATCATCGCCGGGAACTCAATGGCTTTTCCTATCTTCCCTACACAACAACAAAGCTCCCTTCCTAATAACAACAATCACTTGGTTCTAGAAGGTCACCCACAAGCACCTCCTTCAGCCTTCCCTGGTCACCAGATCAATAAACGTTTGGAACATTGGTCAAACGCTGTATCATCATCATCGTCCACTCTTCCTCCTCCTGGTCAGAACAGTAATAGCCTCATCAGTCATCAGTTCGATGCCTCCTCATCAAGCTATTCCATCCCATTCTGTGACTCTACAATTCCATTGAATCCAGCGTTGGATCATACAAATCCCCGAGCTTTCTACAGAGCCACGGACATGGATTCAAGTGCAAATGTGCAGCCTGGAGTCTATTATGATTCATTGCAGATGAGAAAATCAGGCAACTACGGTCCAACCACGGATGCTATGCTGAGTAGTAATAACCCCAAGGAAGGGTTCACCGTGGGGCAGCAGAAGTTACAGAGTGGATTCATGGGAGGAGAAGCTGGTTCTTTAGATGATATAGTCAACTCCACTATGAAACAGGAACAGAGCCAAGGAGACTTGTCGGAAGGTGATTTGGGATATGGAGGCTTTAGCTCACTTAGAACATGCATATGA

>BrRR31(Bra026635)-DNA

ATGACCATCAGCGATCAGTTTCCTTGTGGGTTAAGAGTCCTTGTCGTAGACGACGACGCTTCCTGTCTGATAATCCTCGAGAAAATGCTTCTCCGCCTCATGTACCAAGGTGACTGTCCCATTTCTCTCTTCAAAATCTTCTCTAAAGTCCCTTCCTTTTTGCTCTGTTTTTGCAGTTACCATCTGCTCCCAAGCCGACGTCGCTTTAACCCTCTTGAGAGAAAGAAAAGGCTGTTTCGATCTGGTCTTGAGCGATGTTCATATGCCTGGTATGAACGGCTACAAACTCCTCCAGCAAGTCGGTCTCGAGATGGATCTTCCTGTCATCAGTAAGTAAGAAAGGTTTTGACTTTTTTTCTTTCTGTTCTCTGTTTCTTTACACTTTGCTCTGTTTTTTTTTTTGTCTTCTCTCTTTAGTGATGTCTGTTGATGGAAGAACAGCGACGGTAATGACGGGAATCAACCACGGAGCTTGTGATTATCTTATTAAGCCGATTCGTCTTGAAGAGCTCAAGAACATATGGCAACACGTGGTTCGCAGGAAGTGCACAATCAACAAGAACAGTATCAGCAGCAGCAGCAGCAGCTTGGGGAGTCTTTTCTCTGTTAGTGGAGTCTCAGAGGGGAGTTTGAAACGTAGGAAGAACAAGAGAAGGGTTGATAGTGAAGAAGACGATCTTCTTGATCCTGGAAACAGTTCCAAGAAGTCGCGTGTTGTTTGGTCCATGGAGTTGCATCAACAATTCGTTAAGGCCATAAACCATCTTGGAATCGAGAGTATGAACTTAAACCAATGTTGAGAACTCTGTAGCTTGTCATGTGTGTTTTACTGACCACAACTCTCCTTTTTTGGTTTCAGAAGCTGTACCAAAGCGGATTCTTGAGTTGATGAGTGTGCCTAGCTTAAGCAGAGAAAACGTCGCTAGTCATTTACAGGTTTGGTTGTTTTGTTTGGTTTTCAATCTAGCTTTAAGGTTTCTTGTTTATTTCTGGATGTGTCTTAGAATTAGAAAAAAGTTATTTTATTAAAATAAGGATATTTTCTTAAAAGGAAAACAAAATCGGATTTTGAAACTATTATAAATATAGGTGTAATATATTGTAAATTTATTCATTTACATAGTCAATATACAAAACTCTAAAAAAGTATTTGCCTCAATTTGTTTTGCTCTCTTAAGTTTATTTGCTTTCTTCAAAATAAGATATTATAAATCTATTCATTCACATAATCAATAATATACAAAACCTAAAAAATATTTGCCTCAATTCTTTTTGCTTGCTTTCTTCAAAATTAGTTCGTGTTTGTTCACAACATTTCTTGATTAATTGCAGAAGTACCGATTGTATTTGAAGAGATTAAGTGGTGCAGCTTCTCAGAGTAGGGACGCTGAGTCTATGGAAAGATATGAAAACATTCAAGCTATGGTTTCCTCTGGACAGATACATCCGCAGGCATTAGCTGCCTTGTATGGTCGACCAATAGACAATCATATGTCTGGTGGTTTTGGAGTTTGGATACCTACTGACAATCATCTTGGTGGATCTAATGTATCATCAGCTTCTAACCGGTGTTTTGGGGCTTTGGACAGTCCTTCATCTGTTGCTGCTTCGATGTCTGTTCATGGTTTATCTTCCTCTGGAAATGTAAGACAGCAAGGTAATGGTTTCAGCAACAACACAGACTACAGAATCAGACAAGGGAATGGGTCAGGCATCAGTGAAGAATCTTGGATCTTGGGAAGACCTTTAAGACAGCGAAAGGCTTAA

>BrRR31(Bra026635)-CDS

ATGACCATCAGCGATCAGTTTCCTTGTGGGTTAAGAGTCCTTGTCGTAGACGACGACGCTTCCTGTCTGATAATCCTCGAGAAAATGCTTCTCCGCCTCATGTACCAAGTTACCATCTGCTCCCAAGCCGACGTCGCTTTAACCCTCTTGAGAGAAAGAAAAGGCTGTTTCGATCTGGTCTTGAGCGATGTTCATATGCCTGGTATGAACGGCTACAAACTCCTCCAGCAAGTCGGTCTCGAGATGGATCTTCCTGTCATCATGATGTCTGTTGATGGAAGAACAGCGACGGTAATGACGGGAATCAACCACGGAGCTTGTGATTATCTTATTAAGCCGATTCGTCTTGAAGAGCTCAAGAACATATGGCAACACGTGGTTCGCAGGAAGTGCACAATCAACAAGAACAGTATCAGCAGCAGCAGCAGCAGCTTGGGGAGTCTTTTCTCTGTTAGTGGAGTCTCAGAGGGGAGTTTGAAACGTAGGAAGAACAAGAGAAGGGTTGATAGTGAAGAAGACGATCTTCTTGATCCTGGAAACAGTTCCAAGAAGTCGCGTGTTGTTTGGTCCATGGAGTTGCATCAACAATTCGTTAAGGCCATAAACCATCTTGGAATCGAGAAAGCTGTACCAAAGCGGATTCTTGAGTTGATGAGTGTGCCTAGCTTAAGCAGAGAAAACGTCGCTAGTCATTTACAGAAGTACCGATTGTATTTGAAGAGATTAAGTGGTGCAGCTTCTCAGAGTAGGGACGCTGAGTCTATGGAAAGATATGAAAACATTCAAGCTATGGTTTCCTCTGGACAGATACATCCGCAGGCATTAGCTGCCTTGTATGGTCGACCAATAGACAATCATATGTCTGGTGGTTTTGGAGTTTGGATACCTACTGACAATCATCTTGGTGGATCTAATGTATCATCAGCTTCTAACCGGTGTTTTGGGGCTTTGGACAGTCCTTCATCTGTTGCTGCTTCGATGTCTGTTCATGGTTTATCTTCCTCTGGAAATGTAAGACAGCAAGGTAATGGTTTCAGCAACAACACAGACTACAGAATCAGACAAGGGAATGGGTCAGGCATCAGTGAAGAATCTTGGATCTTGGGAAGACCTTTAAGACAGCGAAAGGCTTAA

>BrRR32(Bra020390)-DNA

ATGGAGTCGGTGAGCAATGAAGAGGGAAGGAGTGATCAGTTTCCGGTGGGGATGAGGGTCCTTGCTGTGGATGACAATCCAACCTGCCTCCGGAAACTTGAAGAGTTACTGCTTCGATGCAAGTATCATGGTACATAACTCTTTGCTTCTGTCTGTTTTGTTTTGTTTTGTTTTGTTTGTGTACTGGATCGCCGCATCATAGTTTGATTGATTTGGTCAGTGACAAAGACGATGGAGTCAAAGAAAGCTTTGGAAATGCTGAGAGAGAAGAGCAACATGTTTGATTTGGTGATAAGCGATGTGGAGATGCCTGATACAGATGGCTTTAAGTTGCTTGAGATTGGTCTTGAAATGGACCTTCCTGTCATCAGTTACGTCTCCCTTTACTCCCTTTTCATGTCATTTTTATTTAGCCTTTTGTCTAAATCTTTTAGTGTTTTGTGTGTTCTCAGTGTTGTCAGCGCATAGTGACTACGACAGCGTTATGAAAGGGATAATCCACGGCGCGTGTGATTATCTTGTCAAACCCGTTAGCCTCAAGGAGCTTCGGAACATATGGCAGCATGTTGTGAAGAAGAACATTGGCTCATACAAAAAAATCATCGCCCCCTCTCGCCACCTACTCCCTACTTCTGAGTACGCCCCAAGAGGCAGCGGGAAACGGAAAGAGAAGGCTGATGACAGCGGTGATGGAGATGATGACAGTGACAGAGACGATGAAGAAGATGGGAGTGAGCAAGATGGAGATGAGTCATCCAGCAGGAAGAAGCGTCGTGTTGTTTGGTCACAGGAGCTTCACCAGAAGTTTGTCCATGCCGTTCAGCAATTGGGCCTTGACAGTGAGTTTGTTTCCCCTTAAACCTCTTATCACGTTTACTTGTGTGTGTGTGAGTATGTGATGTTGATGTCAAGCGTTGTCTCTCCATTTTACAGAAGCTGTTCCCAAAAAGATACTTGATTACATGAACATAGAAGGTCTCACGAGGGAAAACGTAGCCAGCCATTTACAGGTGCTAATTTTTCATCTACTCTTGCATTTTGACCAATATTTTAAAAACCGGATAGGAAACTGAACGGGATAATTATTTGGGTCATGGTTCAGACATAGTTCAATAATTTAGGTTTAATATATTATTAGTATATAAATTTTAAAGTAATGTTCGTAAATATGATACATAACTAAAATAAAAATAAATTTCAAACAGAAAACATTATATATATAATAGTTTTTATGTTCATATGGTTGATTTATGGGTTTTTTTTTAATATAGTTTTAACGATGATGAGACCATACCTAATAGTGTTCCTTTTGTCTCTGGTTCTGTCTGCAGAAGTACAGACTCTACTTGAAAAAGCTAGACGAAGGTCAGCAGCATAACATGTCTCAAGATGCATTTGGGTCAAGAGACTCATCTTACTTTCACATGGCTCAGCTTGAAGGACTAAGAGACTATTCTTCCACAAGACAACTCTCAAGCTCATCCCTCTTAACCCGCTCCAGTCTCACCAAGTTCCATCCCTCCGTGTATTCATCGGTAAACCTTCAAGGATCTAACTCCTCTAGCTTCATCCCACCGGGGCATCATCAGAGCTCAAGCAGCTCAGCTAATCCATTCGGAACATATCACAGTCCTCTCTTGGCAAGATCTCAGAACGTGAATCTCTCTCCGCTAGAGCCACTCCAGTTTCCTAGAAGCAAGTGTTCTCCATACATGGGGGACTTTAAAGGCATAGCGGACCGAGGAATCGGCAGCAGCTTCCTTGATTCTCGGATGTCATTTGGCAGCTCTAGCACCTCTTTGCCTTGTGCTACAAGCAACAATCTGATGCTGCAAGAGAACTTTGGGGTCTCTGATGGAAACCAGTCTTGCCTCAATGGCTTATCTAGTTTCCCTAGTCATCATAGCTGGCAAGGGAATCTGAAAACAACAACTAGATTCCCATCACACTCTTTACCGCTGAATCATGCCTTTGGTCAAGATCAAATGACATGTGGAGGGACCGGTCTAGGAGATTATAACACCTCATTGGTTTCTGCTGATAGCCATGTTGGAGTATTGCAGTGTGAGCCTCCGTTTCTAGGTGACTTCATGCAGAACATGAATACACATAAATGGGAGGAACAGAACTGCACCATGATGAACAACACATTCGGTAATGTTGACTATCCCTTACCAGTGGATAATAATATGGTTTTTAGAGATAACAACGCGACCAGAAGCAAAGGTGTGGATGATTCACTGATGATGAGTCCAATAGAGGACAGTGCAACAACTCTCAACAGTAGAGAGTGTGTGGGAAACGTTACGATGATGGATCCAGAGATGAGGTCGTCGACGAAGCTAGAGAATGATCTTGTTGACAACCAGAACGATGTGTTCGATGATATAATGAATGAGATGTTGAAACAGGTAAAGTAAAAAGTGACTTGTGATTGGTGTGATTGGACTGAAGTTTTTTTTTTGTTAATAGTAGAGAATGAAAGTGATGTTGTGTTTGTTTGTTGCAGGATGAGAATAATGGAATGGTGTCAGTGGCTGCTAGGTTTGGGCTTTGA

>BrRR32(Bra020390)-CDS

ATGGAGTCGGTGAGCAATGAAGAGGGAAGGAGTGATCAGTTTCCGGTGGGGATGAGGGTCCTTGCTGTGGATGACAATCCAACCTGCCTCCGGAAACTTGAAGAGTTACTGCTTCGATGCAAGTATCATGTGACAAAGACGATGGAGTCAAAGAAAGCTTTGGAAATGCTGAGAGAGAAGAGCAACATGTTTGATTTGGTGATAAGCGATGTGGAGATGCCTGATACAGATGGCTTTAAGTTGCTTGAGATTGGTCTTGAAATGGACCTTCCTGTCATCATGTTGTCAGCGCATAGTGACTACGACAGCGTTATGAAAGGGATAATCCACGGCGCGTGTGATTATCTTGTCAAACCCGTTAGCCTCAAGGAGCTTCGGAACATATGGCAGCATGTTGTGAAGAAGAACATTGGCTCATACAAAAAAATCATCGCCCCCTCTCGCCACCTACTCCCTACTTCTGAGTACGCCCCAAGAGGCAGCGGGAAACGGAAAGAGAAGGCTGATGACAGCGGTGATGGAGATGATGACAGTGACAGAGACGATGAAGAAGATGGGAGTGAGCAAGATGGAGATGAGTCATCCAGCAGGAAGAAGCGTCGTGTTGTTTGGTCACAGGAGCTTCACCAGAAGTTTGTCCATGCCGTTCAGCAATTGGGCCTTGACAAAGCTGTTCCCAAAAAGATACTTGATTACATGAACATAGAAGGTCTCACGAGGGAAAACGTAGCCAGCCATTTACAGAAGTACAGACTCTACTTGAAAAAGCTAGACGAAGGTCAGCAGCATAACATGTCTCAAGATGCATTTGGGTCAAGAGACTCATCTTACTTTCACATGGCTCAGCTTGAAGGACTAAGAGACTATTCTTCCACAAGACAACTCTCAAGCTCATCCCTCTTAACCCGCTCCAGTCTCACCAAGTTCCATCCCTCCGTGTATTCATCGGTAAACCTTCAAGGATCTAACTCCTCTAGCTTCATCCCACCGGGGCATCATCAGAGCTCAAGCAGCTCAGCTAATCCATTCGGAACATATCACAGTCCTCTCTTGGCAAGATCTCAGAACGTGAATCTCTCTCCGCTAGAGCCACTCCAGTTTCCTAGAAGCAAGTGTTCTCCATACATGGGGGACTTTAAAGGCATAGCGGACCGAGGAATCGGCAGCAGCTTCCTTGATTCTCGGATGTCATTTGGCAGCTCTAGCACCTCTTTGCCTTGTGCTACAAGCAACAATCTGATGCTGCAAGAGAACTTTGGGGTCTCTGATGGAAACCAGTCTTGCCTCAATGGCTTATCTAGTTTCCCTAGTCATCATAGCTGGCAAGGGAATCTGAAAACAACAACTAGATTCCCATCACACTCTTTACCGCTGAATCATGCCTTTGGTCAAGATCAAATGACATGTGGAGGGACCGGTCTAGGAGATTATAACACCTCATTGGTTTCTGCTGATAGCCATGTTGGAGTATTGCAGTGTGAGCCTCCGTTTCTAGGTGACTTCATGCAGAACATGAATACACATAAATGGGAGGAACAGAACTGCACCATGATGAACAACACATTCGGTAATGTTGACTATCCCTTACCAGTGGATAATAATATGGTTTTTAGAGATAACAACGCGACCAGAAGCAAAGGTGTGGATGATTCACTGATGATGAGTCCAATAGAGGACAGTGCAACAACTCTCAACAGTAGAGAGTGTGTGGGAAACGTTACGATGATGGATCCAGAGATGAGGTCGTCGACGAAGCTAGAGAATGATCTTGTTGACAACCAGAACGATGTGTTCGATGATATAATGAATGAGATGTTGAAACAGGATGAGAATAATGGAATGGTGTCAGTGGCTGCTAGGTTTGGGCTTTGA

>BrRR33(Bra014172)-DNA

ATGTCTATAGCCCATATAACCGAGGATGGAGATAAAGCTCTCTTTCTGCAGCAAGAAACTTCTGAAATCAACTCTCCTCTTAATGAATTTCCGCCGAGTACTAATGTTCTTGTTGTCGACGCCAATCTCAGCACTTTACTTGATATGAAAGAAATCATGGAACGCTGCGCCTATCATGGTAACTCTCTTCTTACATATGTATGTATAAATTTAGCGATTTAATAAAGAGTTGTTAAAGTTAGGGTTTAGGGTTTATACTTATGTGTTTTGTCGTTATCCAGTGACGGCTTATGCGGACGCGGAAGAAGCTATTGCGTTTCTGACAAAGTGTAAACATGAGATTAATATTGTGATTTGGGATTATCATATGCCTGGAATTAATGGACTCCAAGCTCTCGCAATCATTGGTTCAAAGATGGATCTTCCTGTAGTAAGTATGTATATCGCACATTAGAGTTTTATTTTCCGAACTATTATAAACAAAATTCTCTTAATTCGATAGAAATATAATAATTTATTTAATCTTTGTGTAGTTATGTCTGGTGACGATCAAACAGAATCGGTGATGAACGCAATGGTACATGGTGCATGTCACTGTGTTATGAAACCCGTTAGAAAAGAGATCATAGCCACCATATGGCAACACATTGTACGCAAGAGGATGATGTCTAAACCCGGTTTAGTTCCACCTGTTGTGGTTCATGGTGACTATTCCAAGCAAGAGAAAGATGATTCCGTGACCGTAGACCAAGATGATAGTGAGGAGAGCATCGATAAGATAGAAGAGAAAGCAACACAGAAACAGACAATGATATGTATAGAAGAAACTCAACCAATGCAATCACATTTGGTTAAGAGTAACGGTTCAGACCAAGACGACGACGATTCCAGGAGCGTAAGAAATTACAACTATGAACAAAGCATCGACAAGAAAAAAGAGAGAGATTTGAAGAGACCGCGGATTTCGTGGACTGGAGATCTTCAACAGAAATTTCTCGAAGCCATCGATATAGTTGGTGGGCCTAAAAGTAATCTAACTATTCTAAAATCTCTAGTCATTCAATTTCTTTATTATTTCTCTTCTATTTACTAATCTTTTTTTTTTAATGCAAAAGCAGAAGCTAGTCCAAAGGTACTTCTCAAATGCTTGCATGACATGAATATCGAAGGACTCACTAGAAACAATGTGTCCAGTCATCTTCAGGTTCAAAGAAAAACTATTATATATTGTGACAATTAAACTAGGATTCTGCATATATGAATAACGCAAGTTCTACATCAATTGTGTTCTCGTGCAGAAATATCGTCTTAGTCTTGAGGAGACCAAAATTCCTCAACAGTTTCCAGAGACTGGTTGGTCTAGTTTGAGTAGACCTTCACCTTTCTTAGGTATGAACAATGGTTTCATAGCACCAACGTCCCTTAGGAATGGTCCAGCTGTTTACCCGGTCCAGGACAATCAATATCAAAATGGTTACTTGGCAATAAACAACAACCAGTTCGTGACCAATAATATGCATGGTTTTCCCTACTCGGAAAATGATCACCATCTCCAGCAGCAGCATCAACAACGACAATATCAGCTTTCTAATCAGATGATGAATTACATGATGAGAAATGAACCTCAACAAGCTTATAATAGCATTGGTTTAACGGATCTCGAACCAAATATTTATCCAAGTCTGCCGTACTATCCAAACGAGTTTCTATTCGATGGCTACAATTTCAGTAACTGA

>BrRR33(Bra014172)-CDS

ATGTCTATAGCCCATATAACCGAGGATGGAGATAAAGCTCTCTTTCTGCAGCAAGAAACTTCTGAAATCAACTCTCCTCTTAATGAATTTCCGCCGAGTACTAATGTTCTTGTTGTCGACGCCAATCTCAGCACTTTACTTGATATGAAAGAAATCATGGAACGCTGCGCCTATCATGTGACGGCTTATGCGGACGCGGAAGAAGCTATTGCGTTTCTGACAAAGTGTAAACATGAGATTAATATTGTGATTTGGGATTATCATATGCCTGGAATTAATGGACTCCAAGCTCTCGCAATCATTGGTTCAAAGATGGATCTTCCTGTAGTAATTATGTCTGGTGACGATCAAACAGAATCGGTGATGAACGCAATGGTACATGGTGCATGTCACTGTGTTATGAAACCCGTTAGAAAAGAGATCATAGCCACCATATGGCAACACATTGTACGCAAGAGGATGATGTCTAAACCCGGTTTAGTTCCACCTGTTGTGGTTCATGGTGACTATTCCAAGCAAGAGAAAGATGATTCCGTGACCGTAGACCAAGATGATAGTGAGGAGAGCATCGATAAGATAGAAGAGAAAGCAACACAGAAACAGACAATGATATGTATAGAAGAAACTCAACCAATGCAATCACATTTGGTTAAGAGTAACGGTTCAGACCAAGACGACGACGATTCCAGGAGCGTAAGAAATTACAACTATGAACAAAGCATCGACAAGAAAAAAGAGAGAGATTTGAAGAGACCGCGGATTTCGTGGACTGGAGATCTTCAACAGAAATTTCTCGAAGCCATCGATATAGTTGGTGGGCCTAAAAAAGCTAGTCCAAAGGTACTTCTCAAATGCTTGCATGACATGAATATCGAAGGACTCACTAGAAACAATGTGTCCAGTCATCTTCAGAAATATCGTCTTAGTCTTGAGGAGACCAAAATTCCTCAACAGTTTCCAGAGACTGGTTGGTCTAGTTTGAGTAGACCTTCACCTTTCTTAGGTATGAACAATGGTTTCATAGCACCAACGTCCCTTAGGAATGGTCCAGCTGTTTACCCGGTCCAGGACAATCAATATCAAAATGGTTACTTGGCAATAAACAACAACCAGTTCGTGACCAATAATATGCATGGTTTTCCCTACTCGGAAAATGATCACCATCTCCAGCAGCAGCATCAACAACGACAATATCAGCTTTCTAATCAGATGATGAATTACATGATGAGAAATGAACCTCAACAAGCTTATAATAGCATTGGTTTAACGGATCTCGAACCAAATATTTATCCAAGTCTGCCGTACTATCCAAACGAGTTTCTATTCGATGGCTACAATTTCAGTAACTGA

>BrRR34(Bra032275)-DNA

ATGTCGGAGGCAAGTAGAACCGATGATGGCGATAGAACTCTGTTTAAGCAGCGAGAAACTTCTCAAATCAGCTCTTTTCTCAACGAATTTCCGGCGAGTGCTAATGTTCTTGTTGTCGAACCCAATTTTGTAACTTTACGTAAGATGAAAAACCTCATGATCAAATACGGCTATCAAGGTCTCTATTTCTCCGTCTCTCATTTTGATCGATAGATTGTGTTATTATTAGGGTTAGGGTTTATACTGATATCTTTTTTTTGTTATCTAGTGACGGTTTATGCGGATGCAGAAGCAGCTCTCGCGTTTCTGAGAAACTGTGAACATGGGATTAATCTCGTGATCTGGGATTTTCATATGCCTAGAATTAATGGAATCCAAGCTCTCAAAATCATTTGTACAAAGATGGATCTGCCTGTCGTAAGTATGTAAATCGAAGATTTTGTTTTCAAATTATATTAATTAAACCAAATTTCTATTCATTAGATTAAAAAAATAATTATATATTTCGTCTTTGTGTAGTTATGTCTGATGATGACCGAAAAACATCGGTGATGCAAGCAACGGTTCATGGTGCATGCTACTATGTTATGAAACCGATTAGAAAGGAGATCATAGCCACCATATGGCAACACATTGTACGCAAGAGGATGATGTCTAAATCAGGTTTAATTCCACCGGTTCAATTAGATGCGGTTCAGAATCACGATGGTTTCAAGGAAAACAAAGATGACTCCATGCCCGTGGACCAAGGTAATAGTGAACAAAACATCAATATGATAGGAGAAAAGGCAGAAAAGAAACCGCAAATTGGAGAAAATCTACCAATCCAATCAGATTCGGTTCAGAATAACGGTTCAGACCAAGACAACAACGATTCATGGACCAAAAGCCCATACAACAGTGAACAAAACATGGATGGAGAAGAAAGAAAGCAACCGAAGACACGGGTGGTGTGGACTAATGATCTTCAAGAGAAATTTCTCAAAGCCGTCGATATTCTTGGTGGTGCAAGAAGTAATATAACTAGTCTTAATCTGTGTTCTATCAATTTATTTATCTTCTTATTATTACCTTCTTTTATTTGGTTCTAATGATATTTTGAATGCAAATACAGAAGCTAATCCCAAGCCACTTCTCAAAATGCTGGAAGACATGAATATCAAAGGGCTCACTAGAAGACATGTGTGTAGTCATCTTCAGGTAAAAGCCAAAACTTTATCGATTCTACAATTAAATATAGGGTTTTGCGAATATTAGTAACGGAAATTCTACATTAATTATGTTGTTGTACAGAAATATCGTCTTAGTCTTGAGGGAAAAGAAATCACTCAACAGATGCAAGAGTTTGGTTGGTCCAGTGCATGTACAACTTCACCTCTCTTAGGTTTGAACAATGTTCACACTGCAACATCGTCCCTTATAAATGGTGGAGCCTCTTACCCGGTCCAGGAGAATCAGTATCAAAATGGCTACATGGAAGTGAACAACAACCACGCTGCATCATCGACCCTTATGAATGGTCGAGCTACTTACCCGGTCCAGGATAATCAGTATCAAAATGGCTACTTGGGAGTGAACAACAACCAGGTCATGACCAATACTATGCCTTATGATTTTGACCATGATCACTATTTGCAGAAGCAGAAGCCTGAAAAAGCTTCCAGCATCGATATACCGGAGGATCTCGGACTGGCGTATACTATGCCTCGTTTGCCCTATAATCTTGGCCATGGGAACCATCTCCAGCATGAACAACAACATCAGCTTTCTCATCAATGGAATAACGTGATGAGTAACAATGAACCGGAACTACCTTCCAGCAATGGTGTAACGGGCGTTGGGGGGACTTATCCAAGTTTACCGTATGACCCAAACGAGTTCTATAATTATAATCAGTAA

>BrRR34(Bra032275)-CDS

ATGTCGGAGGCAAGTAGAACCGATGATGGCGATAGAACTCTGTTTAAGCAGCGAGAAACTTCTCAAATCAGCTCTTTTCTCAACGAATTTCCGGCGAGTGCTAATGTTCTTGTTGTCGAACCCAATTTTGTAACTTTACGTAAGATGAAAAACCTCATGATCAAATACGGCTATCAAGTGACGGTTTATGCGGATGCAGAAGCAGCTCTCGCGTTTCTGAGAAACTGTGAACATGGGATTAATCTCGTGATCTGGGATTTTCATATGCCTAGAATTAATGGAATCCAAGCTCTCAAAATCATTTGTACAAAGATGGATCTGCCTGTCGTAATTATGTCTGATGATGACCGAAAAACATCGGTGATGCAAGCAACGGTTCATGGTGCATGCTACTATGTTATGAAACCGATTAGAAAGGAGATCATAGCCACCATATGGCAACACATTGTACGCAAGAGGATGATGTCTAAATCAGGTTTAATTCCACCGGTTCAATTAGATGCGGTTCAGAATCACGATGGTTTCAAGGAAAACAAAGATGACTCCATGCCCGTGGACCAAGGTAATAGTGAACAAAACATCAATATGATAGGAGAAAAGGCAGAAAAGAAACCGCAAATTGGAGAAAATCTACCAATCCAATCAGATTCGGTTCAGAATAACGGTTCAGACCAAGACAACAACGATTCATGGACCAAAAGCCCATACAACAGTGAACAAAACATGGATGGAGAAGAAAGAAAGCAACCGAAGACACGGGTGGTGTGGACTAATGATCTTCAAGAGAAATTTCTCAAAGCCGTCGATATTCTTGGTGGTGCAAGAAAAGCTAATCCCAAGCCACTTCTCAAAATGCTGGAAGACATGAATATCAAAGGGCTCACTAGAAGACATGTGTGTAGTCATCTTCAGAAATATCGTCTTAGTCTTGAGGGAAAAGAAATCACTCAACAGATGCAAGAGTTTGGTTGGTCCAGTGCATGTACAACTTCACCTCTCTTAGGTTTGAACAATGTTCACACTGCAACATCGTCCCTTATAAATGGTGGAGCCTCTTACCCGGTCCAGGAGAATCAGTATCAAAATGGCTACATGGAAGTGAACAACAACCACGCTGCATCATCGACCCTTATGAATGGTCGAGCTACTTACCCGGTCCAGGATAATCAGTATCAAAATGGCTACTTGGGAGTGAACAACAACCAGGTCATGACCAATACTATGCCTTATGATTTTGACCATGATCACTATTTGCAGAAGCAGAAGCCTGAAAAAGCTTCCAGCATCGATATACCGGAGGATCTCGGACTGGCGTATACTATGCCTCGTTTGCCCTATAATCTTGGCCATGGGAACCATCTCCAGCATGAACAACAACATCAGCTTTCTCATCAATGGAATAACGTGATGAGTAACAATGAACCGGAACTACCTTCCAGCAATGGTGTAACGGGCGTTGGGGGGACTTATCCAAGTTTACCGTATGACCCAAACGAGTTCTATAATTATAATCAGTAA

>BrRR35(Bra041027)-DNA

ATGTCAGTTTCATCAAACATACTCAAAGAGAATTCTCGCGACTTGTTGCGAGAAGAGGAACCTGGTGATGATGAAGTCGAATTTCCCATTAACGATGAGGACGAAGATTTTTCGATAACGAGTATTAGGATTGTTTTGGTAGACTCAGATCCTGAATCCTTATGCCTCATGAAGAACCTCATGACACAGTACTCTTACCAAGGTATCAAATCACATTTAATTAGGGTTTCTTTTAATGTTTTTAACTTTTTATCAATTAGTTTTAAATTAGGGTTTATGATTAGGTTTTAATTAATTTGTTGTTAGTAAGGGATTTTAAAAACGGAGCTGAAGCTATTGCTTTCTTGATGATGAGCAAGCATGAGATCGATTTAGTGATTTGGGATTTTCACGTGCCCGAGATTAATGGACTGGAAGCTCTCAAAACCATTGGTAAAGAGATGGATTTGCCCGTAGTAAGTACGTAAAATTTCATGATATATTAATCTTTTCTATATAAAACATACATAATATCTTATTCAAAGCTAATGTCAAATTATTTTCAAGTAATGTCTCATGAACACAAGAAGAAAACTGTGATGGAATCGACAAAACGAGGCTCATGTAACTTTCTCCTGAAGCCAGTGAGCAAAGAAATCATTGCAGTTCTATGGCAACATGTTTACCGCAAGAGAGTATCTATATATTCCGTTGAATCAAATCCAGAAGAAAATGTCGGTTTAGATCAAGATGATATTGATCTCTATCAGACCAACTCCAATAGCGGAGAACAAACCAGTTCCTATCAAAAAGAAGGCAAGAACAAGAAACCGCGGATGACTTGGACACCTGAACTTCACCAACTGTTTGAAAAAGCGGTCGAGAAAATGGGCGGTGTTGAACGTAATCATTCACACGAATCTCTTGTGAATATTCAGTTTTTTTTTTCATGTTATTCATTTATTATCTTATTTTTCATTTTGACTATGTTAAATGCAGAGGCCGTTCCAAAGCAGATTCTCAAATGTATGCAAGAAGAAAAGGATGCGGAAGGACTCACTAGAAACAATGTAGCCAGTCATCTTCAGGTAATTATTATCTATATCAATTTTATGTTATTCTAGGCGTTTTGTTTCTTACGAGACAAAACCCCAATATTTAGAGCTAAGAATTTACATACATTGTGATGGCAGAAGTACCGTCTAAATTCAGGGAAAAAGTCATCCATGATCCAGGAAACTCGAGAAGATTCTGAGTGGCGTAATGCTGGACCAAACACCGCTCTCACGGCTTCTAAACCGCTACCAAACTCCATCTTCGGTCTCCATACTAGAGTACCGTATTTTGCGAACGATCAAGACGCAAGAAACGGCCCCATGCAGTATCCTTCAACCAATTACTTCACAATGGACAATGGCCATTTCATGACCAACTCTTTTGCTAATCTACCTTATACTGACTCGTTTCATCAGCAACAACAACAACAATTTCAACATCAGCAGTACTCCAATTCTTCTCTTCAGTTACCTTCCGTGATAACCAAACAAGAGTTTCCATACGTGTCAGCAGCCCTGGAAAATCCCGATCTCATAGCTAATGAAAATTCACTCTACATGGACTTGGGAGACTATTTGCAAGAAGGACTTAGCGATTTCGATAAGACTAATCGTTATTGA

>BrRR35(Bra041027)-CDS

ATGTCAGTTTCATCAAACATACTCAAAGAGAATTCTCGCGACTTGTTGCGAGAAGAGGAACCTGGTGATGATGAAGTCGAATTTCCCATTAACGATGAGGACGAAGATTTTTCGATAACGAGTATTAGGATTGTTTTGGTAGACTCAGATCCTGAATCCTTATGCCTCATGAAGAACCTCATGACACAGTACTCTTACCAAGTAAGGGATTTTAAAAACGGAGCTGAAGCTATTGCTTTCTTGATGATGAGCAAGCATGAGATCGATTTAGTGATTTGGGATTTTCACGTGCCCGAGATTAATGGACTGGAAGCTCTCAAAACCATTGGTAAAGAGATGGATTTGCCCGTAGTAATAATGTCTCATGAACACAAGAAGAAAACTGTGATGGAATCGACAAAACGAGGCTCATGTAACTTTCTCCTGAAGCCAGTGAGCAAAGAAATCATTGCAGTTCTATGGCAACATGTTTACCGCAAGAGAGTATCTATATATTCCGTTGAATCAAATCCAGAAGAAAATGTCGGTTTAGATCAAGATGATATTGATCTCTATCAGACCAACTCCAATAGCGGAGAACAAACCAGTTCCTATCAAAAAGAAGGCAAGAACAAGAAACCGCGGATGACTTGGACACCTGAACTTCACCAACTGTTTGAAAAAGCGGTCGAGAAAATGGGCGGTGTTGAACAGGCCGTTCCAAAGCAGATTCTCAAATGTATGCAAGAAGAAAAGGATGCGGAAGGACTCACTAGAAACAATGTAGCCAGTCATCTTCAGAAGTACCGTCTAAATTCAGGGAAAAAGTCATCCATGATCCAGGAAACTCGAGAAGATTCTGAGTGGCGTAATGCTGGACCAAACACCGCTCTCACGGCTTCTAAACCGCTACCAAACTCCATCTTCGGTCTCCATACTAGAGTACCGTATTTTGCGAACGATCAAGACGCAAGAAACGGCCCCATGCAGTATCCTTCAACCAATTACTTCACAATGGACAATGGCCATTTCATGACCAACTCTTTTGCTAATCTACCTTATACTGACTCGTTTCATCAGCAACAACAACAACAATTTCAACATCAGCAGTACTCCAATTCTTCTCTTCAGTTACCTTCCGTGATAACCAAACAAGAGTTTCCATACGTGTCAGCAGCCCTGGAAAATCCCGATCTCATAGCTAATGAAAATTCACTCTACATGGACTTGGGAGACTATTTGCAAGAAGGACTTAGCGATTTCGATAAGACTAATCGTTATTGA

>BrRR36(Bra005928)-DNA

ATGGCTTTTGCCCAATCTTTCGACAACCAAAGCTCTGATTTAAGAATCAATGTTATGGTCGTGGACGATGATCCTGTTTTCCTAGGAGTTGTGTCACGCATGCTCGAAAAATTCAAATATAGAGGTAATTAAGATCTTATTCTCAAATACAACATGTATAGATAATTTTGTTAAGATTTGAACAGGATAAACCTATTAGCTAACTTCAAATATCTTCCTGATTTCGTTGAGATTTAGAAAAAAAAACTTCAAATATCGTAATTTTTAGCCCTTTGAAAAGTTATATTTTAGTGGAATTCAACAATTGTATTTCTAAATCATCAGGATCTGAACTCCAATTATATGCACACGTCTAAAATTTCAATTGAGTTATTCTTAATTACTTTGATGATTTTTTAAACCAATAGTTTGGTTAATACAAAAGTTTCAACCAATTTAATATGTACGGTTTTAAAAAATATCAATAATTGATTTATAAGATAGTAACTTTTTGTCTGAATGAAAAAATGACATGATCGTGTGTACGTACGATATGTTTTTTGTTCGAAGTTTAAAATTAATTTACGTTCTTCAAATCGCCCTTTCTTATAATGTAATCAACCTGTTAGTCTTTTAAAATGTATATCCATGACAATTTAAATGTTGTTATTTTAAAGAATATGTTTAGTTGATGCTATGAACATTCTAAAATTTTGTGAAATGTTTCATATAAAGTGAAAAATATATATGTATATACATGTATATATATATCTGTGTGTTTATATAATTAGACCTCCATTTTGCTCAGTTTCTTCGTTTTTAAAAGGTTAAGACATGCAAGTAAAAGTATATAGTCTCTCGTACTTGCAATATTTTCACATTTACATTCGAAATTCATTTTTGGTTAAATGGTTTTAGATCCGTCGTTTAAGGAGATCTCGGTTATAGCTGTAAAAGACCCGATCGAAGCATTGTCTACTCTTAAAACCCAAAGACACAATATCGATCTTATAGTCACAGATTATTACATGCCTCACATGAACGGTCTACAACTCAAAAAGCAAATCACTCGAGAATTTGGCAATATACCGGTTATAGGTAAGTTTTTTTTACTACTTACATAAAATTACAATACAATAAGGGTGTAACTGGTAAACAAGTATGAAATTTGTGGAATTTAAAATAAATGAAATGATAGGAATGAAATACAGCAAAAAATGGAATGAAATTCATTCCATTCATTTATGATCAAATTTGTTATGAAATGATTCTCTTTATATTTTCTCTATCTATTTTTGGAATTGATGGAATGAGTATTCCATTCCATTCATGTCAAATGGTAAAAGAATTTACGGAATTAATGGAATATACCATTCCACATCATTCCATTCTAAAAATCTACAATCCAGTTGCAGCCTAATTGTTTCTGCTATAACTAAGCTATAACTATTTTTTCTTTACATTTTCTTAAACATGAAAGTTATGTCATCGGACAGCAACATAGAACATGAGAGTTTGGCTTGTGGAGCGAAGTGTTTTCTTCCAAAACCCATCAGACCAACTGACATACCCCAGATCTACCAGGTTGCTTTAACTTATAAGAGGAATGATAAGTCCATATTATGGACCGAGCACAACTACAGGGACACAGATGTTAGCATCCCTCAGCAAATCCAGTTACATACTGAACAAGCTAATGTCTTGAAGACCAATAATAAGAGGTTCTCGCCTATATCGGATTCAAGACCCGTGAACAGTTCAAATGGAAGTTATGTTAGTACAGACGGTTCAGGAGAAAACCGAAAAAGAAAATCAAACGGTGGTTCTGGTGATGATTCGCGGCCCTTGAAGAAGCCTAAGATAAAGTGGACCGACTGTCTTCATGACCTTTTCCTACAAGCTATCCGATATATCGGTCTTGACAGTAAGTGTTTTAATTATTTGTAGATATATAATACATGATATGGAGTACATCATTTCTTACATTTTCAAGTTTATGTAGATATATACACTGACTAATATGTTTATATTTGTTTACTTAACTTGAATCTTCCAAAGAGGCTGTGCCAAAGAAAATCTTAGAGTACATGAACCTACCGTACTTGACAAGAGAGAACATAGCCAGCCATTTACAGGTTTGATCATATCCCTTTTCAAAAATGTTTTCGGATAATATGTTCTAAACGGGAATTTTCTTTTAAATTAAAAAACAACAATATATGTCAGTCGAATGAAAAGACTTTATGTCTCAATTTGAGATAGTTAGAACTTTTTGTCCGATTCACTTAACTTGTGATTATTTATTTTACCATTTTATGTTAAATAATAAATACTGTAAATTGTGCGTATCATTTCTCTTTTTAATTAACTCAAAATTGTTTGCTACGCAGAAATATCGAATATTCTTAAGGAAAGTTGCTGAACAAGGTTTTTCATGTTCGAGAATGTTGCCGACTAAAGGCATAGACTCGATATTTCTGCAGGCTCACCTCAGAGATCCGTGCTACAACAACTACGCATCATCATCTTCTTCTTTGTACGACACAACTATTAACAATAGATCATTCTATTCCAAACCCATACAAAGTTATGGACAGTCTAGACTCTTGTCCAACACAGCTGAGCCCGTCCGCTTCAACCAGATGCCTTACAACTACATGAACCGGTCATCCACCTATGAGCCGCGAGGTATTGGATCAAACTTAACCATTCCCACCATAAGCAACCTCAGTTTCCCAATCCAACCATCTCAAAACGAAGGAAGAAGAAGCCTTTTCGAACCAACCGTGATGGCGAACAAAACTGTCCAAACATCTCAAGCTATGGGGTTTGGACAACATGGACTCTCGGCTATTAATGGCAATAGTTTCAACAATAACATGGTGAGCAGCTATGGAAGATTGACTCCTACTCAACCAGGAATGATGAGCTATGAAAATTTAACTCCTAGTCAACCAGGAGTGAGAAGCTATGTAAATTCAACTTTTGATCAGCCAGGAATGAACAACCAAAGAAGTTTGACTCCTGACCAACAGGGAATGCGCAGCTACAGAAGTTTAACTTCTAATCAACTAGGAGTGAACATGAATGGAAGTTTTTCTCAAACTCCTAATCAACCAGGAATGAGTAGCTATGGAATTTCAACTTCTAATCAACCAAGAATGAACAACCATGGAAGTTTGACTCTTGACCAACAGGTAATGAGTAGCAACGGAAGTTTAACTTCTAATCAACTGGGAATGAGCAGCCATGGAAGTTTATATCCAAATCAACCAGGTCTGAACAGTTATGGAAGTGTAACTTATAATGCAAGAGTAAACATCCATGAAAGTTTAACACCTAATCAACCAGGAGCTAGCAACTTCTCGTATGGGATGCAAATGTTCTTAAACAACGAGAACACTACATATAAGCCTCAGGCACATGACAATGCAACTACACAGCCAAATCTTGAAATTCCTACACTGGAGAATCTCAGCTTGTGTGATGAGCTTCTGTGCGAGATAAGTAACTTCCAGATCGACCACAACAAGGTATATATCATCATCACTACCCTAATATTTCAAGAATGTACATATACATGCATTATATATATGTTGTGAGACTTTTTTTTTGTCAATGTAACGAGCAGCAACAAGAAGAAGCAGTTTCTACCAACAAATTTGAGCTCCCTGCAAATTTTGAGACTGAACTGAATCAGTTTTTTTCTCTTGAGGAAAATGGTGACGGGAACTTTGTGAACATAAATCAGGTATATACATATTATTTTAGAAATTACAAGATTGAATATGAATCAACGTCCTATCAAAGATTATATATGGTCAAAGAATGTCTTGCTGACTTATATATTTGACTTTGTTCTGAAGGGACGTTCTGATGGAGAAACATCAAACATTGTTGCTGCCCCGGAGACGAATTATCCAGTTTTCAACATGAACCCTAATCATGAGCAGGTACTCTAATATGTATCATTCTAAATATATTTCCATAAATCTATGATGCTATATATATCAAATCATTTTTTTCATTTAAATATACTCTGAAACTTCGAATCTATTTGCTAGGAACAAGGTGTTCCAGGATTTGTCGATTGGTCGTCACTGGATCCGAAGGTACGTAAGGTTAAATTATTTGTCTATAATTTGAAGTTTTTAAGTCATGAAATATTTTTGATTAAACTTTTTCTTATACTATAGGATTTTGCTAATGAGTACGATTTCGTGGACTCGCTGTTAACCAATGACATGAATTAG

>BrRR36(Bra005928)-CDS

ATGGCTTTTGCCCAATCTTTCGACAACCAAAGCTCTGATTTAAGAATCAATGTTATGGTCGTGGACGATGATCCTGTTTTCCTAGGAGTTGTGTCACGCATGCTCGAAAAATTCAAATATAGAGATCCGTCGTTTAAGGAGATCTCGGTTATAGCTGTAAAAGACCCGATCGAAGCATTGTCTACTCTTAAAACCCAAAGACACAATATCGATCTTATAGTCACAGATTATTACATGCCTCACATGAACGGTCTACAACTCAAAAAGCAAATCACTCGAGAATTTGGCAATATACCGGTTATAGTTATGTCATCGGACAGCAACATAGAACATGAGAGTTTGGCTTGTGGAGCGAAGTGTTTTCTTCCAAAACCCATCAGACCAACTGACATACCCCAGATCTACCAGGTTGCTTTAACTTATAAGAGGAATGATAAGTCCATATTATGGACCGAGCACAACTACAGGGACACAGATGTTAGCATCCCTCAGCAAATCCAGTTACATACTGAACAAGCTAATGTCTTGAAGACCAATAATAAGAGGTTCTCGCCTATATCGGATTCAAGACCCGTGAACAGTTCAAATGGAAGTTATGTTAGTACAGACGGTTCAGGAGAAAACCGAAAAAGAAAATCAAACGGTGGTTCTGGTGATGATTCGCGGCCCTTGAAGAAGCCTAAGATAAAGTGGACCGACTGTCTTCATGACCTTTTCCTACAAGCTATCCGATATATCGGTCTTGACAAGGCTGTGCCAAAGAAAATCTTAGAGTACATGAACCTACCGTACTTGACAAGAGAGAACATAGCCAGCCATTTACAGAAATATCGAATATTCTTAAGGAAAGTTGCTGAACAAGGTTTTTCATGTTCGAGAATGTTGCCGACTAAAGGCATAGACTCGATATTTCTGCAGGCTCACCTCAGAGATCCGTGCTACAACAACTACGCATCATCATCTTCTTCTTTGTACGACACAACTATTAACAATAGATCATTCTATTCCAAACCCATACAAAGTTATGGACAGTCTAGACTCTTGTCCAACACAGCTGAGCCCGTCCGCTTCAACCAGATGCCTTACAACTACATGAACCGGTCATCCACCTATGAGCCGCGAGGTATTGGATCAAACTTAACCATTCCCACCATAAGCAACCTCAGTTTCCCAATCCAACCATCTCAAAACGAAGGAAGAAGAAGCCTTTTCGAACCAACCGTGATGGCGAACAAAACTGTCCAAACATCTCAAGCTATGGGGTTTGGACAACATGGACTCTCGGCTATTAATGGCAATAGTTTCAACAATAACATGGTGAGCAGCTATGGAAGATTGACTCCTACTCAACCAGGAATGATGAGCTATGAAAATTTAACTCCTAGTCAACCAGGAGTGAGAAGCTATGTAAATTCAACTTTTGATCAGCCAGGAATGAACAACCAAAGAAGTTTGACTCCTGACCAACAGGGAATGCGCAGCTACAGAAGTTTAACTTCTAATCAACTAGGAGTGAACATGAATGGAAGTTTTTCTCAAACTCCTAATCAACCAGGAATGAGTAGCTATGGAATTTCAACTTCTAATCAACCAAGAATGAACAACCATGGAAGTTTGACTCTTGACCAACAGGTAATGAGTAGCAACGGAAGTTTAACTTCTAATCAACTGGGAATGAGCAGCCATGGAAGTTTATATCCAAATCAACCAGGTCTGAACAGTTATGGAAGTGTAACTTATAATGCAAGAGTAAACATCCATGAAAGTTTAACACCTAATCAACCAGGAGCTAGCAACTTCTCGTATGGGATGCAAATGTTCTTAAACAACGAGAACACTACATATAAGCCTCAGGCACATGACAATGCAACTACACAGCCAAATCTTGAAATTCCTACACTGGAGAATCTCAGCTTGTGTGATGAGCTTCTGTGCGAGATAAGTAACTTCCAGATCGACCACAACAAGCAACAAGAAGAAGCAGTTTCTACCAACAAATTTGAGCTCCCTGCAAATTTTGAGACTGAACTGAATCAGTTTTTTTCTCTTGAGGAAAATGGTGACGGGAACTTTGTGAACATAAATCAGGGACGTTCTGATGGAGAAACATCAAACATTGTTGCTGCCCCGGAGACGAATTATCCAGTTTTCAACATGAACCCTAATCATGAGCAGGAACAAGGTGTTCCAGGATTTGTCGATTGGTCGTCACTGGATCCGAAGGATTTTGCTAATGAGTACGATTTCGTGGACTCGCTGTTAACCAATGACATGAATTAG

>BrRR37(Bra009284)-DNA

ATGGCGTTTGCCCAACCTTTCTACAACCAAAGCTCTCTCTTGAGAATCAATGTTATGGTTGTGGATGATGATCCTGTTTTCCTTGAAATCATGTCACGCACGCTTGAAAAATTAAAATACAGAGGTAAGATCTTATTATCAAATACAGTATGTTATTTATTTTTTTGTATGTTCATTGATATTTGAACAGGATAGACCAATTAGCTGAGAGTGTTCAAAAAAAAAAAAAAAAAAAAAAAAAGACCAATTAGCTGATTACAAACAATAGTAATTTTTGTAGCCCTTTCAGAATATATTTCAGTGAAATTTAAGAATTATCTTTCTAATTTTTTAGGATTTGAATATCAATTACATACATACATCTAAAAATTCGATTGAACTATTCTTATTTTGATGATTTTACAACTAATGGTTTTGGTGATACGAAAACTTCAACGAATATACTATTTAATTTTTAAGAAGAATATATATAGGATAATGTTGATTTAGAAAATAATAACTTATCGTTGGATAAATAATTATATGTTCATAGTGTTAGTGTACGAAATGAACTTTTCTCGAAGGTTCAAATTAATTGACGGTCTTCAAATCGGCTTTCTTATAAAGTTCATCCAGTTAGCCTTTAAAACAAATGCATAGCCATTTAAATGAAAATGTTGTGATTTTAAAGAATATGTTTGGCTGATGCTATGAGTATTCTCAAAAGTTTTAAATATTTCACAGCTCAAGTGTGGAAAAATATTCAGCAAAAAAAAAATAATCGGGGGAAAATATTTAGTAAAAATAGGTTGAATTAGGGGAAACAAACAAATAAATTAAAATATATACATACCTCAATTTTGCCCACTTTCTTCTCTTTTTGAAAAAGGTTATAACAATTTAAAAGTAAACAATATTTCGTACTTGCAATATTTTCACAATTGATTTTTATTGGTTAAATGCTTTAGATTCATCCACTATGGAGATCACGGTTATAGCTGTAAAAGATTCGAGAGAAGCATTGTCTACTCTTAAAATCGAAAGAAACAATATCGATCTCATAGTCACAGATTATTACATGCCTGACATGAACGGTCTACAACTCAAAAAACAGATCACTCAAGAATATGGCAATTTACCGGTCATAGGTAAGTGTTTTTGCAACTTACATAAGATTATAATATATAAATATATATGTATATATATATTTTTTTTTTTGCTATAATTCGAAGCTATAACTTTGTTCTTTACATTTTCTTATATGAAAGTTATGTCATCGGACACCGATAAAGAACATGAGAGTTTAACGTGTGGAGCGATGGGTTTCATTCAAAAACCCATCAAAGCAACTGAATTAACCAAGTTTTACCAACTTGCGTTAAAATGTAAGCGGAATGGTAAGTCCACCTTATGGACCGAGAACAACCACAATGACACAGATGTTAGCATCTCTCAGCAAATCCAGTTTTTTCCTGAGCAAGACAATCTCATGATGACCAAGACGAAGAAGTTCTCACCTAGACCGGATTCAAGATCCATGAACAGTTCAAATGGAACTTGTGTTAGTACTGATGCTTCACGGAAAAATAAAAAAAGAAAAGCAAACGGTGGTTCTGGTGATGGTGTTGAGTCTTTGTCGCAGCCCTCCATGAAGAGTAAGATTACGTGGACGGATGATCTTCATGATCTTTTCTTACAAGCTATCCGACACATCGGTCTTGACAGTAAGTCTTTTGATTTTTTTTTTTAACATTTTTATATATAATACATGTTATACAGTTCATGTCTTGACTAATTTTCTATGTTTGTTTACTAATTAAACTTAAATCTTCGACAGAGGCTGTGCCAAAGAAAATCTTAGAGTTCATGAACGTATCATACTTGACAAGAGAGAACGTAGCCAGTCATTTACAGGTTTGATCACATCCTTTTCAAAAATGTTATTTGGCTTTATGTTTTGAACGATTTTTATTTTTTCAAAACCAATAGTTTATGAAAGTCAAAAACAATAGTTCCATGTTTAATTAATATTAAATACCAAACCATAGATTGTGTATCATTCCTCTTTTAATTAACTAAAAGTTGTTTACTACGCAGAAATATCGACAATTCTTGAGGAAAGTCGCGGAAAGAAGTTCTTTGTGTTCATCAAACATGTTGCCGAGTAATGGCATAGACTCGATATATCCATATCCTCACACTAGAGAGCCGTACTACAACAACTACACTTCTTCTTCTTCTTGGTACGGCACAAGTCTCAGCAATAATAGATCATTCTATTCCAACCCCGGACACGGTCTTGGACAGTCTAGACTCTTATCCAACACATCTGATCCAGTCCGCTCCAATCAGATGCCTCACAGCTACATGAACCGGTCATCCACCTATGATACGCACCGTATTGGATCAAACTTGACGCTGCCCGTCGAGAGCAACCTCAATTACTCATCCCAAAATGTAGGAAGAAGAAGCTTTCTTGAACCAACGGCAAACAAAACTAGCCAAACATCTCAAGCTCTCGGGTTTGAACAGCATGGATTATCAGCTATTAATGGTAGTGGTTTCAACAATAACACGTTGATCAGCTATGGAAGTTTAGCTCCTAATCAACTAGGAACTAACAGCTATAAAGGTTCAATTTCTACTCAACAAGGAATGACCAATGGAAGCCTGGCTCTTAATCAACCAGGAATGAGCGCCTATGGAAGTTCAACTTCTACTCAACCAGGCATGAGCAGCCATATAAGTTTGACTCCTAATCAACCAGGAAGGAACAGCTATGGAAGTTTAACTGCTCCTCAACCAAGAATGAGTACCCATGAAAGCTTATCTCCTAATCAACAAGGAATAAGCAGTCATAAAAATTTAAATTCTAGTCAGCTAGGAATGGACAGCAATGGAAGCTTATCTCATGATCAACCACGTATGAGCAGCTATGAAAATTTAACTTCTAATCAACTAGGATTGAGCAGCCATGGATTTTTAACTCCTAATCAACCAGGACTTAAAAGTTATGGAAGTGCAACTCATAACGTAGGACTAAACAGCTTTGAAGGTTTAACTACTCATCAACCAGGATCTAGCAACTTCTCGTATGGGTTGCAATCATTCTTAAACAATGAGAACACTGCATATGAGCCTCAACCTCTGACACATGCCCAAGCAGCTGCACAGACAAATATTGAAATTCCTCAACAGGAGAATTTCAGCTTGTTTGATGAACTTGCCAATATTAATGAGCTTCTTTGCGACATAAGTAACTTTGAACTGGATCACAACAAGGTATGTGTCTTCACTATCCTAATTTTTCAATAATGTATATATACTGTATACATGACGTATATGTTGTGAGATTTGTTTGTCTGAAATGTGATGCGTGCAGCAACAAGAAGCGGTTTCTACCACCCAATTTGAGCTCCCTGCAAATGTTTCGACTGAAATGAATCAATTTTTCTCTCTTGAAGATGATGACTGGACCTTTGTGAACACAAACCAGGTACTAAATATTATTAGAAAATTTTGGCAGTTACAATATCGGGTTTGAAGCAACTTCCTATCAAAGATTATATATATATTATCAAATCTTTAAGAATCTACAGTGGGAAATTTTATAGCTAGATTTCTCCATTATAATGTCTTAGCGACTTATATAGTTGGCTTTGTTGTGAAGGGATTTTCTAATGGAGAAACATCAAACAATGTTGCTCCGGAGACGAATTCTCAAACTTTCAACATGAGCACTAATCATGATCAGGTACCTCCAGTTTGCATTATTCTAAAATGTTTCCATGTAACTATGATACTAAATATCAACGAGATGATTTATTAAATATAATATGAAACCTGGAATCTATTTGGTAGGAACAAGATGCTCAAGATTTTGTCGACTGGTCGTTCTTGAATCCGGAGGTAAAACAATATAACCTTAGCAAAAGCTGAAAGAGATTATTTTTCAAGTTTAAAATTGTAGTTTATGCGTCATGTGATATATTATTGGTTAACCTTTTTTATATATAGGACTTGGCTAATGAGTACGACTTCATGGACTCTTTGTTCAATGGCATGAACTGA

>BrRR37(Bra009284)-CDS

ATGGCGTTTGCCCAACCTTTCTACAACCAAAGCTCTCTCTTGAGAATCAATGTTATGGTTGTGGATGATGATCCTGTTTTCCTTGAAATCATGTCACGCACGCTTGAAAAATTAAAATACAGAGATTCATCCACTATGGAGATCACGGTTATAGCTGTAAAAGATTCGAGAGAAGCATTGTCTACTCTTAAAATCGAAAGAAACAATATCGATCTCATAGTCACAGATTATTACATGCCTGACATGAACGGTCTACAACTCAAAAAACAGATCACTCAAGAATATGGCAATTTACCGGTCATAGTTATGTCATCGGACACCGATAAAGAACATGAGAGTTTAACGTGTGGAGCGATGGGTTTCATTCAAAAACCCATCAAAGCAACTGAATTAACCAAGTTTTACCAACTTGCGTTAAAATGTAAGCGGAATGGTAAGTCCACCTTATGGACCGAGAACAACCACAATGACACAGATGTTAGCATCTCTCAGCAAATCCAGTTTTTTCCTGAGCAAGACAATCTCATGATGACCAAGACGAAGAAGTTCTCACCTAGACCGGATTCAAGATCCATGAACAGTTCAAATGGAACTTGTGTTAGTACTGATGCTTCACGGAAAAATAAAAAAAGAAAAGCAAACGGTGGTTCTGGTGATGGTGTTGAGTCTTTGTCGCAGCCCTCCATGAAGAGTAAGATTACGTGGACGGATGATCTTCATGATCTTTTCTTACAAGCTATCCGACACATCGGTCTTGACAAGGCTGTGCCAAAGAAAATCTTAGAGTTCATGAACGTATCATACTTGACAAGAGAGAACGTAGCCAGTCATTTACAGAAATATCGACAATTCTTGAGGAAAGTCGCGGAAAGAAGTTCTTTGTGTTCATCAAACATGTTGCCGAGTAATGGCATAGACTCGATATATCCATATCCTCACACTAGAGAGCCGTACTACAACAACTACACTTCTTCTTCTTCTTGGTACGGCACAAGTCTCAGCAATAATAGATCATTCTATTCCAACCCCGGACACGGTCTTGGACAGTCTAGACTCTTATCCAACACATCTGATCCAGTCCGCTCCAATCAGATGCCTCACAGCTACATGAACCGGTCATCCACCTATGATACGCACCGTATTGGATCAAACTTGACGCTGCCCGTCGAGAGCAACCTCAATTACTCATCCCAAAATGTAGGAAGAAGAAGCTTTCTTGAACCAACGGCAAACAAAACTAGCCAAACATCTCAAGCTCTCGGGTTTGAACAGCATGGATTATCAGCTATTAATGGTAGTGGTTTCAACAATAACACGTTGATCAGCTATGGAAGTTTAGCTCCTAATCAACTAGGAACTAACAGCTATAAAGGTTCAATTTCTACTCAACAAGGAATGACCAATGGAAGCCTGGCTCTTAATCAACCAGGAATGAGCGCCTATGGAAGTTCAACTTCTACTCAACCAGGCATGAGCAGCCATATAAGTTTGACTCCTAATCAACCAGGAAGGAACAGCTATGGAAGTTTAACTGCTCCTCAACCAAGAATGAGTACCCATGAAAGCTTATCTCCTAATCAACAAGGAATAAGCAGTCATAAAAATTTAAATTCTAGTCAGCTAGGAATGGACAGCAATGGAAGCTTATCTCATGATCAACCACGTATGAGCAGCTATGAAAATTTAACTTCTAATCAACTAGGATTGAGCAGCCATGGATTTTTAACTCCTAATCAACCAGGACTTAAAAGTTATGGAAGTGCAACTCATAACGTAGGACTAAACAGCTTTGAAGGTTTAACTACTCATCAACCAGGATCTAGCAACTTCTCGTATGGGTTGCAATCATTCTTAAACAATGAGAACACTGCATATGAGCCTCAACCTCTGACACATGCCCAAGCAGCTGCACAGACAAATATTGAAATTCCTCAACAGGAGAATTTCAGCTTGTTTGATGAACTTGCCAATATTAATGAGCTTCTTTGCGACATAAGTAACTTTGAACTGGATCACAACAAGCAACAAGAAGCGGTTTCTACCACCCAATTTGAGCTCCCTGCAAATGTTTCGACTGAAATGAATCAATTTTTCTCTCTTGAAGATGATGACTGGACCTTTGTGAACACAAACCAGGGATTTTCTAATGGAGAAACATCAAACAATGTTGCTCCGGAGACGAATTCTCAAACTTTCAACATGAGCACTAATCATGATCAGGAACAAGATGCTCAAGATTTTGTCGACTGGTCGTTCTTGAATCCGGAGGACTTGGCTAATGAGTACGACTTCATGGACTCTTTGTTCAATGGCATGAACTGA

>BrRR38(Bra028705)-DNA

ATGACTTTTGCCCAATCTCTCAACAACCAAAGCTCTGTCTTGAGAATCAATATTATGGTTGTTGATGATGATCCTGTCTCCCGTGAAATTGTGTCACGCATGCTTGAAAGATCCAAATACAGAGGTAAGGTTATCAAATACAATATGTTACTAAGTTCTTTGTAACTTCATTGATATTTGAAACCAATTAGCCGAACAGAAAGATAGTAAAATTTTAGCCCTTTCAAAAGTTACATTTTGTTGAATTTCAAAATTAAATTTCTTAATCATTAGGATGTGAAATCCAGTTACCTTCACACATCTAAATTTTCAATATAGAGATATTTCTATTTTGATGATTTTTAAACTAATAGTTTTGGTTGATACAAAAATACAACCAATGTACTATGTAAGTTTTCAAAAGTATATTGAACAAAAATGTAAAAAAAAAATTGATATGATATTTTTACCCAAAAAATGATATTTACATCGTGTTTTGCGTCACAACATGATTTTTTTTTGTTGGCTTTCAAAGTTTAAAACTAATGGATGTTCTTCAAGTCTACTTTTCTTATAAATAGTCAATCAGTTAGCCTTTCATAGCCTTTCAAATAAATTAGTGTATAACTAACATCTATTTTGCACTTTGTTCTCATTTTAAAAAGGTTAAAACAATTAAAAGTAATAATCTTTGTACTTTAATATTTTCCCATTACATTTGAATCTTTACTTTGGTCAAATGGCTTTAGATCCATCTATGGAGATCACAGTTATAGCTGTAAGAGACGCAAGGGAAGCATTGTCTACTCTTAAAATCCAAAGAAACAACATCGATCTTATAGTCACAGACTATTACATGCCTGGCATGAATGGTCTACAACTCAAACAACAAATCACTCGACAATTTGGCAACTTTCCGGTCATAGGTCAGTTTGTTTTTTTTTTCTTTTACAACTTACATACAACTGAAAAATTATAATATTTTCTTAAAATAATAATAATATTTTTCTACAAAATTATAAAATTTTCCTAAAAGTTGTTGTATTTCTTTCTAAAAATTTTGGTAAGAAATTGTAATATTTTTTTAAGAAACTATAATATTTTGAAGAAAAATTAAATTGTTTTGCTATAATTCTAAGTTTTAACTATTTTTCTTACATTTTATAAAATATGAAAGTTATGTCATCGGACACCAACAAAGAACAAGAGAGTTTAGCTTGTGGATCGGTGTGTTTCCTTCCAAAACCCATCAAACCGACTGACCTACCAAAGATCTACCAAGTTGCTTTTACTTATAAGAGGAAGGGTAAGTCCATATCAAGGACCGAGCACAACCACATGGATACAAATGTTAGCATCCCTCAGCAAATCCAGTTGCTTCCTGAACAAGCTAATGTCTCAAAGACCAAGAAGAACAAGGAGTTCTCATCTAAATCAGATTCACGATCTGTGAACAGTTTCAATGGAAGTTGTGATAGTACAGATGGTTCACGAAAAAATCGAAAAAGAAAATCAAACGGTGATTTTGGTGATGATGATGAGTCTTTGCCGCAGCCTTCCAAGAAGAGTAAGCTTTCGTGGTCGGACTACCTTCATGACCTTTTCTTACAAGCTATCCACCATATTGGTCTTGACAGTAAGTGTTTTGTTTTTTTTTTTAATTATAACGTATTTATACAATACGCAATATACTGTTTATTTTCTTACATTTTCTTACAAACTTGTTTCTTTGCTGAACTTGAATCTTCCAAAGAGGCTGTGCCAAAGAAAATTTTAGAGTTCATGGACGTATCATACTTGACAAGAGAGAACGTTGCCAGCCATTTACAGGTTTGATCATCGATCTCTTTTTTAATGTTTTTTGATGTTTTAAACGGGATTATTATTTTTATTTTTAACAACAATTTATGTAAGTCAACATTAAACTAACGAAGTTTTAAGGAATACTAGATTTTTACCCGCACAACCGTGCGGGTATATATTTTCACATTGATATATATAGATATTTGTTTTACATAATTACTATATATTTTTTTATGTTACTTACATATTTAAATGTTTGTATAATTATGTCAAATATAATAATTTTATAGTTTTCATGTTGTAAATTAAAATCATCACATATATATGTTGCTTATTATATATTTGTCCTATTGAATTTGTGTTTGATTACGAAACTAAATTTTTTAATGCATGAAACAACATATATGAAAATAATTTTGTATTTAATTTATTATAATCATGATCCATAGTTCAAATCGCTAGATTTTTTTAGTAATTTTTAATGCTTATTAATTTTATATAATAAATTACTGTATATTAAAAAATTTAAGATAAGATAAATTTTTATACATGTATTATATAGTTTACTAATATTAACCCGTTCTACCAACATATTATATTTTTAGCATAAATATTTTATATTTATGAAAATAAAATATGTTAACTTATCAATTTAAAATAATTTTATCATATCTTGTTCAATATAACGTTTTTATTTTAAAATGATAGATATTATTATAAAATGATAAAATAGAATATAATTTTATTCTTTTAGTAACATTTCATTACTAATTACAAAATTAGTTGAAAATATTTATATTAAATTTATGACAATTAAGATCTTATTATAATCTTTTTCAAGAGATTTGTTAGAATTTTAATTTTTTTTTAAAAAAATTAAAAGATATAAAAGATATTATGATTAAAGTAGTTAAAAATATTATGTATATTAGCATTAGTGATATACATTTAATATAAAATTTAAATGATGGTCCAAATAAAAATATCACTCATCAAAAAATCATGATTTTTATTTTATTAGAAAACAAATCAATTTTAATTTCTAATTATATTTTATGATAATTTAAATTAAAACTAACTAATTTTTGAAAGTAAATTTAAAAAGATTCTAAGAAGATTTTAAAAAGATTTTGTTAGAACATTTTAAATATATTCATTTGTATTTCAAATAAAAAAATAAAGATATTAAAAATATAATAATGAACTTATGTAAAATATGATATTTTTTAGAAATGGTCCAAACTAAAAAAATCACACATGAAAAGAAGTCATGACTTCTGTTTTAATATATAAGATAGTAGTATTTTCGTTAAAGTAGTATTTTAAATACTCTGTTAAACAGTGTTAAAGAAATCTTAAAGTTAACTACCAATGGATTTAAATTGGTCTATTTTTCATTGACCTAGTTATAACTTTTTCTTCTATTCGTTTAAGTTGTGTGTTTTTTTTTAATTGCTCTCCATTTTTAAAAACTATAGATCACTAGATTGTGTATCACTCACCTTTAGTTAACTAAAAACTGTTTACCATACAGAAATATCGAAATTTTTTGAGGAAAGTCGCGGAAAATTCAGGCATGTTGCATGGAAGAGGCATGGAGCCGTACCATAGTAACTACACAACCTCTTCTTCTTGGTACGACACAGGTCTTAACAACAAATCTAGCTATTCCAAACCTAGACACGGTCTTGGACAGTCTAGGCTCTTATCCAACACATGTGAACCTGTCCGCTTCAACCAGATGCCTTACAACCACATGAACCGGTTATCTACCTATGAGCCGCACCGTACTGGATCAAACTTGACCATGCCCATCAAAAGCAACCTCAGTTTTTCAACGCAGCCATTGCAAAACGAAGGAAGTAGAAGCTTTCTTGAACCAACCGTGACGGCGAACAAAACTGGCCAAACATCTCAAGTTCTCGGGTTTGGACAACATGGAATGTTGGCTATAAATGGAAATAATTTCAACGACAACACGATGAGCAGCTATGCAAGTTCAACTTCTAATCAACCAAGAATTAACAGCCATGGAAGTTCGACTCCTAATCAACCAGGATTGAGGAGCTATGGATGTTCGATATCTAATCAACCAGGAATGAGTAGCTATGCAAGTGTATCTCCTAATCAACTAGGAATGAGCAGCTATGGAAGCTTAACTCCTTATCAACGAGGAATGAGCAGCCTTGGAAGCTTTACTCCTACTCAACCAGGAATGAGTAGCCATAGAAGCTTGACGCCTACTCAACAAGGAATGAGTAGCTATGGGAGTTTATCTCCTAGTCAACCAGGAGTGAGTACTCAACCAGGAATGAGCAGCAAGGGAAGCTTGACTCCTACTCAACGAGGAATGAGTAGCTATGGGAGTTTAACTTCTTATCAACCAGCATTGAGCACCCATGGAAGTTTATTTCCTAATCAACCAGGAATAAGCAGCCACACAAGCTTACCTCCTACTCAACCAGGAATGAGTAGCTTTGGAAGTTTAACAACTAATCATTCAGGAATGAGTAGCTACGAAAGTTTAACTCCTACTCAACCGGGGCCTAGCAATATCTCATACGGATTGCTCTTAAACAATGAAAACACTGCATATAAGCCTCAACCACATGCCTCTACAACTATACAACTGGATAATCTCAGCATGTATGATGACCTCGGCAACATTAACGAGATTCCTTGCGATTTAAGTAACTTTGACTTCGATCATGACAAGGTATATGTCTTCACATCCCAAAATGTTTCAATTATGTATATATACATGAACTATATGTTGTGAGATTTTCTTGTCTGATATGTGATACGAGCAGCAACAAGAAGAAGCAGTATCTGCCAACAAATTTGAGATCCCTGCAAATCTTGAGACTGAACTGAATCAGACTTCCTCTCTTGAAGAAGATGGTGACTGGACCTTTCTGAACATAAGTCAGGTACATACATATTATTAGGTGACCTTTAGCAATTACAAGATTGTATTTGAATGAACTTCCTATCATAAGATTATACATTATCATATGTTTGAGAATCTAGTGTGTGAAATTTTCTAACTAGATTTGTTACTATTAGAACTTTAGTAGATCTCTGCTACTCTTCTTATAAACAAATATAGGCATTGTATCTAGACATTTGTTCAATATATCTATATATTTAGAAGAAGATAGTGGCTGAATCTTTCTGAACCTAAATCCGGTACATACATATTATTAGGGGATTTAGCAATCACAAGATTGTATTTGAATCAACTTCCTATCAAAGATTATATACATCATCATATGTTTGAGAATATAATATTGTTGGAAATTTTCTAACTAAATTTGTTACTATTAGAACTTTAGTAGAAGATTTCTGCTACTCTTATAAGCAAATATATATATATGCACTGTATCTAGACATTTGTTCAATATATTATATCTATACATTTAGAAGGAGTACTTTCGATATATATAAAGTCTTATTGAATAATATATTTGACTTTGTTGCGAAGGGTCATTTTAATGAAAAAACATCAAACACTTTTGCTGCTCCGGAGACGAATGATCCAACTTTCAACAAAAACCCTAATCATGCTCAGGTACCTCTTATTTGTATTATTTTATAATATTTCCATGATATATCAACGATATTATTATTTTTAACTCTGAAACGTCGCATATATTTGGTAGGAACAAGATGTTCCAGATTTTGATTGGTCGTTGTTGGATTTAGAGGTAAAAGTTTAAACTATTTTTTCTATATAAGTTTATATTGCAGTTTATTGGTCATTGAATATTTTTTGATTAAATTTGTTTCTATATATCTAGAACTTGGCTAATGAGAACGACTTCATGGACTCAATGTTCACCAATGACAACTCAATGTTCACCAATGACATGAATTGA

>BrRR38(Bra028705)-CDS

ATGACTTTTGCCCAATCTCTCAACAACCAAAGCTCTGTCTTGAGAATCAATATTATGGTTGTTGATGATGATCCTGTCTCCCGTGAAATTGTGTCACGCATGCTTGAAAGATCCAAATACAGAGATCCATCTATGGAGATCACAGTTATAGCTGTAAGAGACGCAAGGGAAGCATTGTCTACTCTTAAAATCCAAAGAAACAACATCGATCTTATAGTCACAGACTATTACATGCCTGGCATGAATGGTCTACAACTCAAACAACAAATCACTCGACAATTTGGCAACTTTCCGGTCATAGTTATGTCATCGGACACCAACAAAGAACAAGAGAGTTTAGCTTGTGGATCGGTGTGTTTCCTTCCAAAACCCATCAAACCGACTGACCTACCAAAGATCTACCAAGTTGCTTTTACTTATAAGAGGAAGGGTAAGTCCATATCAAGGACCGAGCACAACCACATGGATACAAATGTTAGCATCCCTCAGCAAATCCAGTTGCTTCCTGAACAAGCTAATGTCTCAAAGACCAAGAAGAACAAGGAGTTCTCATCTAAATCAGATTCACGATCTGTGAACAGTTTCAATGGAAGTTGTGATAGTACAGATGGTTCACGAAAAAATCGAAAAAGAAAATCAAACGGTGATTTTGGTGATGATGATGAGTCTTTGCCGCAGCCTTCCAAGAAGAGTAAGCTTTCGTGGTCGGACTACCTTCATGACCTTTTCTTACAAGCTATCCACCATATTGGTCTTGACAAGGCTGTGCCAAAGAAAATTTTAGAGTTCATGGACGTATCATACTTGACAAGAGAGAACGTTGCCAGCCATTTACAGAAATATCGAAATTTTTTGAGGAAAGTCGCGGAAAATTCAGGCATGTTGCATGGAAGAGGCATGGAGCCGTACCATAGTAACTACACAACCTCTTCTTCTTGGTACGACACAGGTCTTAACAACAAATCTAGCTATTCCAAACCTAGACACGGTCTTGGACAGTCTAGGCTCTTATCCAACACATGTGAACCTGTCCGCTTCAACCAGATGCCTTACAACCACATGAACCGGTTATCTACCTATGAGCCGCACCGTACTGGATCAAACTTGACCATGCCCATCAAAAGCAACCTCAGTTTTTCAACGCAGCCATTGCAAAACGAAGGAAGTAGAAGCTTTCTTGAACCAACCGTGACGGCGAACAAAACTGGCCAAACATCTCAAGTTCTCGGGTTTGGACAACATGGAATGTTGGCTATAAATGGAAATAATTTCAACGACAACACGATGAGCAGCTATGCAAGTTCAACTTCTAATCAACCAAGAATTAACAGCCATGGAAGTTCGACTCCTAATCAACCAGGATTGAGGAGCTATGGATGTTCGATATCTAATCAACCAGGAATGAGTAGCTATGCAAGTGTATCTCCTAATCAACTAGGAATGAGCAGCTATGGAAGCTTAACTCCTTATCAACGAGGAATGAGCAGCCTTGGAAGCTTTACTCCTACTCAACCAGGAATGAGTAGCCATAGAAGCTTGACGCCTACTCAACAAGGAATGAGTAGCTATGGGAGTTTATCTCCTAGTCAACCAGGAGTGAGTACTCAACCAGGAATGAGCAGCAAGGGAAGCTTGACTCCTACTCAACGAGGAATGAGTAGCTATGGGAGTTTAACTTCTTATCAACCAGCATTGAGCACCCATGGAAGTTTATTTCCTAATCAACCAGGAATAAGCAGCCACACAAGCTTACCTCCTACTCAACCAGGAATGAGTAGCTTTGGAAGTTTAACAACTAATCATTCAGGAATGAGTAGCTACGAAAGTTTAACTCCTACTCAACCGGGGCCTAGCAATATCTCATACGGATTGCTCTTAAACAATGAAAACACTGCATATAAGCCTCAACCACATGCCTCTACAACTATACAACTGGATAATCTCAGCATGTATGATGACCTCGGCAACATTAACGAGATTCCTTGCGATTTAAGTAACTTTGACTTCGATCATGACAAGCAACAAGAAGAAGCAGTATCTGCCAACAAATTTGAGATCCCTGCAAATCTTGAGACTGAACTGAATCAGACTTCCTCTCTTGAAGAAGATGGTGACTGGACCTTTCTGAACATAAGTCAGGGTCATTTTAATGAAAAAACATCAAACACTTTTGCTGCTCCGGAGACGAATGATCCAACTTTCAACAAAAACCCTAATCATGCTCAGAACTTGGCTAATGAGAACGACTTCATGGACTCAATGTTCACCAATGACAACTCAATGTTCACCAATGACATGAATTGA

>BrRR39(Bra001099)-DNA

ATGGCAACAACGTCAACATCCACGGGAGATATCAAGAAAACCAAGTCAGTAGAAGTGAAGAAGAAACTTAACGTGCTGATCGTCGATGATGATACAGTAATTCGTAAACTCCACGAGAATATCATCAAATCGATCGGTGGAATTTCACAGACAGCTAAGAACGGTGAGGAGGCAGTGAACATCCACCGCGACGGCAATGCATCTTTCGACCTTATCCTAATGGATAAAGAAATGCCCGAGAGGGATGGACTTTCGGTACAATTAAATAATAATCTTTAATTTAATTTGTGTCGATCATCACTACACTTATCAACCTCCTCTTTAAAATACTCTTTTATGTCCTTTTGTTGGTTTTGTTTAGGCAACTAAGAAGCTAAGAGAAATGAAAGTGACGGCTATGATTATTGGGGTGACGACACTGGCTGACAATGAAGAGGAACGTAAGGCTTTCATGGAAGCTGGACTTAACCATTGCTTGGCAAAACCCTTAAGCAAAGCCAAGATCCTCCCTCTCATCAACAATCTCATGGATGCTTGA

>BrRR39(Bra001099)-CDS

ATGGCAACAACGTCAACATCCACGGGAGATATCAAGAAAACCAAGTCAGTAGAAGTGAAGAAGAAACTTAACGTGCTGATCGTCGATGATGATACAGTAATTCGTAAACTCCACGAGAATATCATCAAATCGATCGGTGGAATTTCACAGACAGCTAAGAACGGTGAGGAGGCAGTGAACATCCACCGCGACGGCAATGCATCTTTCGACCTTATCCTAATGGATAAAGAAATGCCCGAGAGGGATGGACTTTCGGCAACTAAGAAGCTAAGAGAAATGAAAGTGACGGCTATGATTATTGGGGTGACGACACTGGCTGACAATGAAGAGGAACGTAAGGCTTTCATGGAAGCTGGACTTAACCATTGCTTGGCAAAACCCTTAAGCAAAGCCAAGATCCTCCCTCTCATCAACAATCTCATGGATGCTTGA

>BrRR40(Bra040204)-DNA

ATGGCAACAAAATCCATGGGAGATATCGAGAAAATAAAGAAGAAACTAAACGTGTTGATCGTCGATGATGATCCACTAAACCTTATAATTCATGAGAAGATCATCAAAGCGATAGGGGGTATTTCACAGACAGCAAATAACGGCGAGGAGGCAGTAATCATCCACCGTGACGGCGGCTCATCTTTTGACCTTATCCTAATGGACAAAGAAATGCCCGAGAGGGATGGAGTCTCGGTACAATTAATTAATAATCTTAGTCTATGGAAATAAACAGATTATTTAATTAACCTCAGGCTAATATCCTTGTCACTTTCTTATGTTCTTTATTTTGTTTGTTTTATTTAGACAACTAAGAAGCTAAGAGAAATGGAAGTGAAGTCAATGATTGTTGGGGTGACTTCACTGGCTGACAATGAAGAGGAGCGCAGGGCTTTCATGGAAGCTGGACTTAACCATTGCTTGGCAAAACCGTTAACCAAGGACAAGATCATCCCTCTCATTAACCAACTCATGGATGCTTGA

>BrRR40(Bra040204)-CDS

ATGGCAACAAAATCCATGGGAGATATCGAGAAAATAAAGAAGAAACTAAACGTGTTGATCGTCGATGATGATCCACTAAACCTTATAATTCATGAGAAGATCATCAAAGCGATAGGGGGTATTTCACAGACAGCAAATAACGGCGAGGAGGCAGTAATCATCCACCGTGACGGCGGCTCATCTTTTGACCTTATCCTAATGGACAAAGAAATGCCCGAGAGGGATGGAGTCTCGACAACTAAGAAGCTAAGAGAAATGGAAGTGAAGTCAATGATTGTTGGGGTGACTTCACTGGCTGACAATGAAGAGGAGCGCAGGGCTTTCATGGAAGCTGGACTTAACCATTGCTTGGCAAAACCGTTAACCAAGGACAAGATCATCCCTCTCATTAACCAACTCATGGATGCTTGA

>BrRR41(Bra020537)-DNA

ATGGCTGGTGAAGTTCCAAATTTGGAAGCGTCGAAGTTAACAGCTTTGGTCGTCGATGACAACTTTGTAAACCAAAGTGTCCATCATAAACTTTTGGACCGTCTCGGGATTAAAAACGACGTCGTATCTAACGGCCAAGAAGCCGTTGACGTTCATTGTTCCGGCAGAAACTATGATCTCATCCTCATGGACATGGATATGCCCATCATGAATGGTATTCAGGTATATTGATATATCTTTCACTCATCCATAATAAAGAAGCCTTAGGAAACTGTTCTACTTAATGCTGGAATTAGACTCCTTATAAATGCATTTTAGTATTTTTCAATAACTGATGGATTGTTTTGTATTGATTAATTGAATATTTTTGTATTAGGCCACAAGGAGACTAAGAGAGATGGGGATAGAGAGCAAGATAGCAGGAGTAACAACTAGAGCTGAGGAAGAAGAGGTGAAAGAGTTTATGGAAGCTGGACTTAATGACTTTCAAGAGAAACCTCTCACCATTTCTAAACTTGTCTCTATTCTTCATAATCTTGAGCTCTACGTCCAAACCTAG

>BrRR41(Bra020537)-CDS

ATGGCTGGTGAAGTTCCAAATTTGGAAGCGTCGAAGTTAACAGCTTTGGTCGTCGATGACAACTTTGTAAACCAAAGTGTCCATCATAAACTTTTGGACCGTCTCGGGATTAAAAACGACGTCGTATCTAACGGCCAAGAAGCCGTTGACGTTCATTGTTCCGGCAGAAACTATGATCTCATCCTCATGGACATGGATATGCCCATCATGAATGGTATTCAGGCCACAAGGAGACTAAGAGAGATGGGGATAGAGAGCAAGATAGCAGGAGTAACAACTAGAGCTGAGGAAGAAGAGGTGAAAGAGTTTATGGAAGCTGGACTTAATGACTTTCAAGAGAAACCTCTCACCATTTCTAAACTTGTCTCTATTCTTCATAATCTTGAGCTCTACGTCCAAACCTAG

>BrRR42(Bra036579)-DNA

ATGGCTGATGAAGTTCCAAGCTTGGCAGAGTCGAAGTTAACAGCTTTGGTCGTCGATGACAACTTTGTAAATCAAACCATGCATCACAAACTGCTGGACCGTCTAGGTATTAAAAATGACGTCGTTTGCAACGGCCAAGAAGCTGTCGACGTTCATTGTTCCGGGAGAAACTATGATCTTATTCTTATGGACATGGAAATGCCCATCTTGAACGGTATTCAGGTAATATTAATTTATTATATTTTTAGCTCATTAAATTCACTTCGTTTATTCAATTCTTATTTTTTGTTATAAAATTCAGAAAAGAATAAGTTATTACTAATTAATTTTTATTTGAGAATTTTCATATCCATAAACCTTAGTAAAAGAAGTTGTATGAAACTGTTCTAGTTAAATGTTGGAATAAGACTCCTTAATACAGTTTTGTTTATTAATAATGTGCAGTACCCTTTTTATTATTTTCTTAAAAATAGAACCGATGGTTTATATGTATAACTAATGGACTGTGTTTTGTTTGATTAGGCGACAAAGAGACTAAGAGAGATGGGGATAGAGAGCAAGATAGCAGGAGTAACAACTAGAGCTAACGAAGGAGATAAGAAAGAGTTTATGGAAGCTGGTCTTAATGACTTTCAAGAGAAACCTCTCACCATCTCCAAACTTTTGTCGATTCTCCACAGACTTGACTTTCATGTACAAACCTAG

>BrRR42(Bra036579)-CDS

ATGGCTGATGAAGTTCCAAGCTTGGCAGAGTCGAAGTTAACAGCTTTGGTCGTCGATGACAACTTTGTAAATCAAACCATGCATCACAAACTGCTGGACCGTCTAGGTATTAAAAATGACGTCGTTTGCAACGGCCAAGAAGCTGTCGACGTTCATTGTTCCGGGAGAAACTATGATCTTATTCTTATGGACATGGAAATGCCCATCTTGAACGGTATTCAGGCGACAAAGAGACTAAGAGAGATGGGGATAGAGAGCAAGATAGCAGGAGTAACAACTAGAGCTAACGAAGGAGATAAGAAAGAGTTTATGGAAGCTGGTCTTAATGACTTTCAAGAGAAACCTCTCACCATCTCCAAACTTTTGTCGATTCTCCACAGACTTGACTTTCATGTACAAACCTAG

>BrPRR1(Bra035933)-DNA

ATGGATTTGAACGGTGAGTGTAAGGGAGGAGATGGGTTTATTGACAGAAGCAGAGTTAGGATTTTGCTTTGTGACAATGATTCCAAGAGCCTGGGAGATGTTTTCACCCTCCTTTCACAGTGTTCTTATCAAGGTATGCTTTACAACTGATGGTTTTTACTTTGCTATTTGCCATGAAAGTTGGAAGTTCTACTTTAGATTGCAGATCATGGACCTCTATTGATTTGAGTTTTTGTTAGTAGTCAAGGATTAGATTTGGTGTATACTCTAGTTATGAATGGGATGGTTCAAGTATTTTCTTACAATCTGTTCCAGTTAGGTTACTTGTAGAGTCTTTCCCTTGTTCTTGTGCTGTGCAAAGTTTTTAATCAGTTGGTTCCTTGATCACATGTGTTTTAAAAAGAGATTATTTAAGTGACCTCTTTTGTTGATTTTAGTATCATTTTTGTAGTTTTTAAATGCATATGTAGTGTCTATTGCATTCTTTTTTCTTTTTTCTTTTTATTACTTTTTGTACAATGAATATTGTGAATGGATGTTTTTCTTGAAACGCAGTGACTTCAGTGAAATCAGCAAGGCAGGTGATTGATGCACTAAACGCAGAGGGACCTGATATCGATATAATACTTGCGGAAATTGATCTCCCCATGGCCAAGGGTATGAAGATGCTGAGGTACATCACACGTGACAAAGATCTTCGGAGAATCCCTGTCATAAGTAAGCTTTGTACTTTATCTGCTTAAAGCTTGTTTTGACAGCATGATCATTTCCTTAGATTCTAACGAATCTTATTATAATATGCTAAGTGATGTCGAGGCAAGACGAGGTACCTGTTGTGGTGAAGTGCTTGAAGCTAGGTGCAGCTGACTACCTTGTCAAGCCTCTTCGCACCAACGAGCTTCTCAACTTGTGGACATACATGTGGAGAAGAAGACGCATGGTTTGTTTTGCTCCTTACTTCTTAATCTCTGTTACTATACTTTAAGTTAAACTACCTTTGTGTTTTGTTTTTGCAGCTAGGACTTGCTGAGAAGAATATGTTGAGCTATGATTTTGATCTTGTGGGATCTGATCCAAGTGATCCAAACACAAACAGTACCAACCTCTTCTCTGATGACACTGATGACAGAAGTATCAGGTCCACCAACCCGCTGAGAGGACCTTTAAGCCGTCAGGAAAAAGAGGTGAGAAAAGTATTATGAAAGTCTTACAAGTCCTCTAAAAATAGCAAAGAGCTAATTTCTCATGCTTTTGCTGTTAGTGTCCTGCTGCTACTGGTTCTGTTGATGGCACAGCCACTTCAGCTCCTCCTCCATTAAATCATCTTCCTGGGTCTCATCATGAGCCTAATCCAGGTGATTGTTTCGCTTTGATTTAGCACTTTCTAATAGCTTTGTTGCTGTGAATGCTAATATAGTTACCGTTTCTGCAGAGAAATTTTCTTCAGTGCCAAAGAAGAGTAGATTGAAGATTGGAGAGTCCTCAGCTTTCTTCACATATGTCAAGTCTACCGCCCTGGCTACCAACTGTCAAGATCCTCCCCATGTCAATGGAAACGGCTCACTTCATCTTCACCCAGGTGTGGTTGCGGAGAAGCTTCAAGTGGTGGCCAGTGAGGTGATCAACAAGCCCAAGCAAACACACCGAAGCAGAGAGACTGAGAAAAACTTACAGAATGGCGGCGCCACGGAAGAACTTCATGGTAGGAGTTACCAAGAGAGGAATCAGGTTGCTGTGAACAGAAGTAAGGATTCATCTCAAGTTGCCTATCCTTACTATATGCAAGGGGTCATGAACCAAGTTATGATGCAATCAGCAGCCATGATGCCTCAGTATGGTCATCATCAACATCCTCATTACCCACCTAATCATCTCAATGGAATGACAGGAGTTCCTTATTACCACCACCATCCCATGAACACACCCTTACAGCACAATCAAATGTCACAGAATGGTCAGATGTCTATGGTTCATTATCATCCGTCATCTAACGAGGTGAGAGCGAGTAAACTTGACAGAAGAGAGGAAGCTTTGCTTAAATTTAGACGTAAGAGGAACCAAAGGTGTTTTGATAAGAAGATTAGGTATGTGAATAGGAAGAAGCTCGCTGAGAGGAGGCCGCGTGTTAAGGGTCAGTTTGTTAGGAAAATGAACGGGGTGAATGTTGACTTAAATGGGCAGCCTGACTATGATGACGAGGAAGAGGAGGAGGATGAAGAGGAAGAGGAGAATAGGGACTCATCTCCTCAGGATGATGCTCAGGGAACTTGA

>BrPRR1(Bra035933)-CDS

ATGGATTTGAACGGTGAGTGTAAGGGAGGAGATGGGTTTATTGACAGAAGCAGAGTTAGGATTTTGCTTTGTGACAATGATTCCAAGAGCCTGGGAGATGTTTTCACCCTCCTTTCACAGTGTTCTTATCAAGTGACTTCAGTGAAATCAGCAAGGCAGGTGATTGATGCACTAAACGCAGAGGGACCTGATATCGATATAATACTTGCGGAAATTGATCTCCCCATGGCCAAGGGTATGAAGATGCTGAGGTACATCACACGTGACAAAGATCTTCGGAGAATCCCTGTCATAATGATGTCGAGGCAAGACGAGGTACCTGTTGTGGTGAAGTGCTTGAAGCTAGGTGCAGCTGACTACCTTGTCAAGCCTCTTCGCACCAACGAGCTTCTCAACTTGTGGACATACATGTGGAGAAGAAGACGCATGCTAGGACTTGCTGAGAAGAATATGTTGAGCTATGATTTTGATCTTGTGGGATCTGATCCAAGTGATCCAAACACAAACAGTACCAACCTCTTCTCTGATGACACTGATGACAGAAGTATCAGGTCCACCAACCCGCTGAGAGGACCTTTAAGCCGTCAGGAAAAAGAGTGTCCTGCTGCTACTGGTTCTGTTGATGGCACAGCCACTTCAGCTCCTCCTCCATTAAATCATCTTCCTGGGTCTCATCATGAGCCTAATCCAGAGAAATTTTCTTCAGTGCCAAAGAAGAGTAGATTGAAGATTGGAGAGTCCTCAGCTTTCTTCACATATGTCAAGTCTACCGCCCTGGCTACCAACTGTCAAGATCCTCCCCATGTCAATGGAAACGGCTCACTTCATCTTCACCCAGGTGTGGTTGCGGAGAAGCTTCAAGTGGTGGCCAGTGAGGTGATCAACAAGCCCAAGCAAACACACCGAAGCAGAGAGACTGAGAAAAACTTACAGAATGGCGGCGCCACGGAAGAACTTCATGGTAGGAGTTACCAAGAGAGGAATCAGGTTGCTGTGAACAGAAGTAAGGATTCATCTCAAGTTGCCTATCCTTACTATATGCAAGGGGTCATGAACCAAGTTATGATGCAATCAGCAGCCATGATGCCTCAGTATGGTCATCATCAACATCCTCATTACCCACCTAATCATCTCAATGGAATGACAGGAGTTCCTTATTACCACCACCATCCCATGAACACACCCTTACAGCACAATCAAATGTCACAGAATGGTCAGATGTCTATGGTTCATTATCATCCGTCATCTAACGAGGTGAGAGCGAGTAAACTTGACAGAAGAGAGGAAGCTTTGCTTAAATTTAGACGTAAGAGGAACCAAAGGTGTTTTGATAAGAAGATTAGGTATGTGAATAGGAAGAAGCTCGCTGAGAGGAGGCCGCGTGTTAAGGGTCAGTTTGTTAGGAAAATGAACGGGGTGAATGTTGACTTAAATGGGCAGCCTGACTATGATGACGAGGAAGAGGAGGAGGATGAAGAGGAAGAGGAGAATAGGGACTCATCTCCTCAGGATGATGCTCAGGGAACTTGA

>BrPRR2(Bra012964)-DNA

ATGGATTTGAACGGAGAGTGTAAGGGAGGAGGAGGAGATGGGTTTATTGACAGAAGCAGAGTTAGGATTTTGCTTTGTGACAATGATCCCAATAGCTTGGGAGAGGTTTTCACCCTCCTTTCACAGTGTTCTTATCAAGGTATATTTTATATTCAGTTTGATGATGGATTTGAGTTTTTTTTTTACTCTTTGTACAATGAGTATTGTGGATGGCTGTTTTTGTAGTGACGTCAGTCAAATCAGCAAGGCAGGTGATTGATGCACTGAATGCAGAGGGGCCTGACATCGATATAATACTGGCGGAGATTGATCTCCCAATGGCCAAGGGTATGAAGATGCTAAGGTACATCACACGTGACAAAGATCTTCGGAGAATCCCTGTCATCAGTAAGTTCCATTTTCTTTTTAAGTATTCATTTACTTTCTCTACTTAGCAAAGTTTTTTTTTAAATGCATGAGGGTTTGCTGTATGATAACTAATCTTATGATATTTTTCAATTTAAGTGATGTCGAGGCAAGACGAGGTCCCTGTGGTGGTAAAGTGCTTGAAGCTAGGTGCAGCTGACTACCTTGTGAAGCCTCTTCGGACCAACGAGCTTCTCAACTTGTGGACACACATGTGGAGAAGAAGACGCATGGTTTGTTTTTTTTCTATCTTTAGTTCTCTCTATGCGGTTAACTGTCATATTCCTTGTTATATGATTGAGCGTGTGTGTGTGTGTGTTTGTTTTGGCAGCTAGGACTTGCTGAGAAGAACATGTTGAGCTATGAGTTCGATCTCGTGGGATCTGACCCAAGTGATCCAAATACAAACAGCACCAACCTCTTCTCTGATGACACAGATGAGAGAAGTATTAGGTCCACCAATCCGCAGAGAGGAAGTCATCAGGAAAAGGAGGTGAGATAGTATTATGAAAGTCTACAAGTCCTTTCAAAATAGGCAAATTAGCTAATTTTTCTTAGTTTGCGCTCAATGCTTTTGCTGGCAGTGGCCTGTTCCTACTGGTTCCGTTTGTGCTGGCGATGGTGCTGCTGATGGCACAGCCACGTCAACTCCTCCCGTTGCTATCATAGAGCCTCCTTTGAATCATCTTCCTGAACCTCACCATGAGCCTACCAAAAGAAATACTAATCCAGGTGAGATTGCATTGCTTTGATGTAGCACTTTCTAATAACGAGTCTAGCTTCTAATATAGCTACGGTTTTTGTAGCGCAATTTTCTTCAGTACCAAAGAAGAGTAGATTGAAGATTGGAGAGTCCTCCGCTTTCTTCACATATGTCAAGTCTACTGTCAATGGAAATGGCTCAGTTCATCCAGGTATGGCTGAGAAGCTTCAAGCGGTGGCAAGTGAGGTGATCAACAACGCCAAACAAACACGAGGAGGTAGAGAGACTGAGAAAAACAAAGCTCAAGGAGAGAACTTAGTGAACGGCACGCTTGAGCGGTCACGCACGCTTCCAACACCAATGGAACTTCATGGCAGTAGGAGTTACCAAGAAGTTCCAAATTCTATTGAGCGGTCACGCACGCTTCCACCACCAATGGAACTTCACGGTACTAGGAGTTGTTACCAAGAAGGATCTATGGATGATGCCCGGGTTGCTGCTGCTAAGGATTCATCTCAGTTCCCTGCGCAAAATGCCTATCCTTACTATATGCATGGTGTCATGAACCAAGTTATGATGCAATCAGCAGCCATGATGCCTCAGTATGGTCATCATCAACATCCTCACTGCCCGCCTAATCATCTGAATGGCATGACAGGGTTTCCTTATTACCACCACCACCACCAGATGAACACATCGTTACAGAACGGTCATGTGCCTTTACAGAATGGTCAGATGCCTCCTATGGTTCATCATCATCATTCTTGGCCACAGGTGGGAAACCACCCGTCTCCTAACGAGGTGAGAGTGACTAAGCTTGACAGAAGAGAGGAAGCGTTGCTTAAATTTAGACGTAAGAGGAACCAAAGGTGTTTCGATAAGAAGATTAGGTATGTGAATAGGAAGAAGCTTGCTGAGAGGAGGCCACGTGTTAAGGGTCAGTTTGTTAGGAAGATGAACGGCGTAAATGTTGACTTGAACGGGCAGCCTGAACCTGACTCTGCTGACTATGATGACGAGGAAGAGGAGGATGAAGAAGAAGAGGAGAATCGGGACTCGTCTCCTCAGGATGATGCTCTTGGGACTTGA

>BrPRR2(Bra012964)-CDS

ATGGATTTGAACGGAGAGTGTAAGGGAGGAGGAGGAGATGGGTTTATTGACAGAAGCAGAGTTAGGATTTTGCTTTGTGACAATGATCCCAATAGCTTGGGAGAGGTTTTCACCCTCCTTTCACAGTGTTCTTATCAAGTGACGTCAGTCAAATCAGCAAGGCAGGTGATTGATGCACTGAATGCAGAGGGGCCTGACATCGATATAATACTGGCGGAGATTGATCTCCCAATGGCCAAGGGTATGAAGATGCTAAGGTACATCACACGTGACAAAGATCTTCGGAGAATCCCTGTCATCATGATGTCGAGGCAAGACGAGGTCCCTGTGGTGGTAAAGTGCTTGAAGCTAGGTGCAGCTGACTACCTTGTGAAGCCTCTTCGGACCAACGAGCTTCTCAACTTGTGGACACACATGTGGAGAAGAAGACGCATGCTAGGACTTGCTGAGAAGAACATGTTGAGCTATGAGTTCGATCTCGTGGGATCTGACCCAAGTGATCCAAATACAAACAGCACCAACCTCTTCTCTGATGACACAGATGAGAGAAGTATTAGGTCCACCAATCCGCAGAGAGGAAGTCATCAGGAAAAGGAGTGGCCTGTTCCTACTGGTTCCGTTTGTGCTGGCGATGGTGCTGCTGATGGCACAGCCACGTCAACTCCTCCCGTTGCTATCATAGAGCCTCCTTTGAATCATCTTCCTGAACCTCACCATGAGCCTACCAAAAGAAATACTAATCCAGCGCAATTTTCTTCAGTACCAAAGAAGAGTAGATTGAAGATTGGAGAGTCCTCCGCTTTCTTCACATATGTCAAGTCTACTGTCAATGGAAATGGCTCAGTTCATCCAGGTATGGCTGAGAAGCTTCAAGCGGTGGCAAGTGAGGTGATCAACAACGCCAAACAAACACGAGGAGGTAGAGAGACTGAGAAAAACAAAGCTCAAGGAGAGAACTTAGTGAACGGCACGCTTGAGCGGTCACGCACGCTTCCAACACCAATGGAACTTCATGGCAGTAGGAGTTACCAAGAAGTTCCAAATTCTATTGAGCGGTCACGCACGCTTCCACCACCAATGGAACTTCACGGTACTAGGAGTTGTTACCAAGAAGGATCTATGGATGATGCCCGGGTTGCTGCTGCTAAGGATTCATCTCAGTTCCCTGCGCAAAATGCCTATCCTTACTATATGCATGGTGTCATGAACCAAGTTATGATGCAATCAGCAGCCATGATGCCTCAGTATGGTCATCATCAACATCCTCACTGCCCGCCTAATCATCTGAATGGCATGACAGGGTTTCCTTATTACCACCACCACCACCAGATGAACACATCGTTACAGAACGGTCATGTGCCTTTACAGAATGGTCAGATGCCTCCTATGGTTCATCATCATCATTCTTGGCCACAGGTGGGAAACCACCCGTCTCCTAACGAGGTGAGAGTGACTAAGCTTGACAGAAGAGAGGAAGCGTTGCTTAAATTTAGACGTAAGAGGAACCAAAGGTGTTTCGATAAGAAGATTAGGTATGTGAATAGGAAGAAGCTTGCTGAGAGGAGGCCACGTGTTAAGGGTCAGTTTGTTAGGAAGATGAACGGCGTAAATGTTGACTTGAACGGGCAGCCTGAACCTGACTCTGCTGACTATGATGACGAGGAAGAGGAGGATGAAGAAGAAGAGGAGAATCGGGACTCGTCTCCTCAGGATGATGCTCTTGGGACTTGA

>BrPRR3(Bra002512)-DNA

ATGTGTTTCAATAAGAACGATATTACAAACGAAGTGGTAACCGAGAGACAAGCGTTTGGTTCATCCGAAGAAGATGATTCTCGAGTAGAAGATACAGCTGGAAACGCCAACAATTTGTTACAGATTACTCAACAACAACAGCCTTCAGCTCCTGTTGTTAACTGGGAGAGATATCTTCCCGTTAGATCGCTTAAGGTTCTCCTGGTGGAGAATGATGACTCAACTCGCCACATTGTTACTGCTCTTTTGAAGAATTGCAGCTACGAAGGTATTGAATCTTCCTTCTTTATCAGCACTCATGTTGTTGCTTTTGACCAAAATTTGGAAACTTTTGTGTCAGTTACTGCTGTTCCGGATGTTCTCGAAGCATGGAAAGTCCTAGAAGACGAGAACAGTTGCATTGATCTTGTCTTAACGGAGGTTGTTATGCCTGTGAACTCTGGAACCGGTCTGTTGTCCAAGATTATGAGCCATCAGACACTTAAGAACATCCCCGTCATAAGTACTTACTTAAATCCTCATTCTATTGGCTTGATCTAGTTTCAGTATGTGTGTGATGATCATTTTCCACGTTTTGTAGTGATGTCATCTCATGATTCGATGGTTCTGGTGTTCAAGTGTTTGTCGAATGGTGCTGTTGATTTCCTCGTGAAACCTATTAGAAAGAACGAGCTGAAGAATCTTTGGCAGCATGTCTGGAGAAGATGCCACAGCGTAAGACATCTTCTCTGATTACATGTTTCTACAAAAGTTACTCAGTGTTTTTATTTCTTTTTACTTATGCGTTTCCTTGATATAATAGTCTAGCGGCAGCGGGAGTGAGAGTGGGATACACAACAAGAAGTCAGTGAAAACTGAAAGCACTGATGAAGGGTCAGAAGATGATGCCAGCATGAGTGATGAAGATAACGGGAATGATGATAATGGGAGTAATGGATTGAGTAACGGAGAGGGTGGGAGTGATCATGGGAGTGGAACTCAGGTAATCTGTTTCTATGGCGTTAGATTATAACAATATCTGAGGGTATCTCTTTGTGAATTTTTATTGGTTTTGCATTATAATGTTAGAGCTCTTGGACCAAAAGAGCCAGTGATGTTAACGTGGGAACCTATGGCAACGAATGCGAACGTCTTAAGAAACTGAAGGAGGTTGAAGATGAGAATGGACAAATAGGTAACGCACCAACTCATTAGTCAATCAAATGGAACCATTCCAAGATTCTCTCCCTTTTTCAAACTGACGACTTTAGATGATGCTACTGTAGGCATGGGATCGCAGGCAGGACAATGTATGAGTAAGAAAGCTGTTATAGCTTTGGAGAGAAACAATGATGACTTGCTGAATCGCTCCTCTGGTAACTCACAAGTAGAGACCAAAACACCTTCATCTAACCCTGAAGATTTGCAATCACTAGAGCTTACTTTTACAAAACCAAAAGAGGCTGGAGATCAGAGAGTCGGTGGTGATCGAAGTGTTCTGAGGCATTCAAATCACTCTGCATTCTCAAAGTAAGTTCTTGACCTCAACTCCTAAAGGATTGTCGAACCTGTGAGTGATTTGAAGCTCTTTTTTTTTCTTTTCTTGTGTGACACGCAGATACAACAACGGTGCTACTTCTGCTAACAAGGTATATTTTGTCTACGATGCTTAGTGTCGTCTTTGAAATCTATGCCGGTTTCATCGAAGGATTTAAACTTTGCTGAGCAGGCTCCAGAAGAAAACGTAGGAAGCTGTTCTCCTCAAGACAGTTCCGTTGCCAAAATAATTGGTTCCAGTTCAAGCAGTGACAATCCTTCGAATCAGCAGTCTAGTGGAAGCGACCGAGCCGCACAAAGAGAGGCTGCTCTGATGAAGTTTCGCCTTAAACGTAAAGAGCGATGTTTTGAGAAGAAGGTATCTTATCTCTAAACAAGCTTAAGCTCTCATTTCTTGGGAGGCAAGAAAGTTATGGCATGGTTTTGTTTGGTGTGTTTTGCTTTTAGGTAAGGTACCATAGCAGGAAGAAACTAGCTGAGCAACGGCCTCGCATCAAAGGTCAATTCATTCGCAAGTAAGATTCCTCCTCCCCCTCTTCCTCAAAGTCTCTAGATTGTAATGTATTCACAAGAAGTCGTTTGCCTTTAGGATGGATGCTTCTAAATCAGGAAATGAGTGTCAGAGTAGTGACGACAGTTCAAGCAAAATTGGTAAAGAAGACTGA

>BrPRR3(Bra002512)-CDS

ATGTGTTTCAATAAGAACGATATTACAAACGAAGTGGTAACCGAGAGACAAGCGTTTGGTTCATCCGAAGAAGATGATTCTCGAGTAGAAGATACAGCTGGAAACGCCAACAATTTGTTACAGATTACTCAACAACAACAGCCTTCAGCTCCTGTTGTTAACTGGGAGAGATATCTTCCCGTTAGATCGCTTAAGGTTCTCCTGGTGGAGAATGATGACTCAACTCGCCACATTGTTACTGCTCTTTTGAAGAATTGCAGCTACGAAGTTACTGCTGTTCCGGATGTTCTCGAAGCATGGAAAGTCCTAGAAGACGAGAACAGTTGCATTGATCTTGTCTTAACGGAGGTTGTTATGCCTGTGAACTCTGGAACCGGTCTGTTGTCCAAGATTATGAGCCATCAGACACTTAAGAACATCCCCGTCATAATGATGTCATCTCATGATTCGATGGTTCTGGTGTTCAAGTGTTTGTCGAATGGTGCTGTTGATTTCCTCGTGAAACCTATTAGAAAGAACGAGCTGAAGAATCTTTGGCAGCATGTCTGGAGAAGATGCCACAGCTCTAGCGGCAGCGGGAGTGAGAGTGGGATACACAACAAGAAGTCAGTGAAAACTGAAAGCACTGATGAAGGGTCAGAAGATGATGCCAGCATGAGTGATGAAGATAACGGGAATGATGATAATGGGAGTAATGGATTGAGTAACGGAGAGGGTGGGAGTGATCATGGGAGTGGAACTCAGAGCTCTTGGACCAAAAGAGCCAGTGATGTTAACGTGGGAACCTATGGCAACGAATGCGAACGTCTTAAGAAACTGAAGGAGGTTGAAGATGAGAATGGACAAATAGGCATGGGATCGCAGGCAGGACAATGTATGAGTAAGAAAGCTGTTATAGCTTTGGAGAGAAACAATGATGACTTGCTGAATCGCTCCTCTGGTAACTCACAAGTAGAGACCAAAACACCTTCATCTAACCCTGAAGATTTGCAATCACTAGAGCTTACTTTTACAAAACCAAAAGAGGCTGGAGATCAGAGAGTCGGTGGTGATCGAAGTGTTCTGAGGCATTCAAATCACTCTGCATTCTCAAAATACAACAACGGTGCTACTTCTGCTAACAAGGCTCCAGAAGAAAACGTAGGAAGCTGTTCTCCTCAAGACAGTTCCGTTGCCAAAATAATTGGTTCCAGTTCAAGCAGTGACAATCCTTCGAATCAGCAGTCTAGTGGAAGCGACCGAGCCGCACAAAGAGAGGCTGCTCTGATGAAGTTTCGCCTTAAACGTAAAGAGCGATGTTTTGAGAAGAAGGTAAGGTACCATAGCAGGAAGAAACTAGCTGAGCAACGGCCTCGCATCAAAGGTCAATTCATTCGCAAGATGGATGCTTCTAAATCAGGAAATGAGTGTCAGAGTAGTGACGACAGTTCAAGCAAAATTGGTAAAGAAGACTGA

>BrPRR4(Bra009768)-DNA

ATGGGAGAAGTGAGCGACGAAGTTGTTGAGGTGACGGTGGTGGAGAAAGCACCTGAGGCTGGCGGAGGAAAGTTAACGCGGCGGAAGATGCGGCGGAAGGACGCCGCGGAGGGCGGCGACGGTCTGGTGACGTGGGAGAGGTTTCTCCCGAAGATATCTCTCAGGGTTTTGCTCGTTGAAGCAGACGATTCCACCAGACAGATAATCTCTGCTCTCCTCAGGAAATGCAGTTACAGAGGTCAGTTCTAGGACATATCCCTTGTGTTATGTTCTGTCTTTTCGAGTTGTTTGTCTGAACTTAGATTATTCTGAGGGAACTTTACGTTGTCTGAAAAATCTATTGAGTTTGCTGAATCTGTTGTGTCGTTACATAATTCGATTAAAAATAAAGTCTTTTGTCTGAGTACATGTGAAAGACTTTTTCTTTTAATCTCAACTTATAGTTTTCCCTTTTCACTTCTCAGTCGCTGCTGTACCTGATGGGTTGAAAGCTTGGGAGATGCTTAGAGGGAAGCCTGAGAGCGTTGATCTGATATTAACAGAGGTTGATCTTCCTTCGATCTCCGGATACGCTCTGCTAACACTTATAATGGAGCATGACCTCTGCAAGAACATTCCTGTCATAAGTATGTGTTATTTATATAACTTCTGCTTCTTTTTCTTAGTTATTGAAATAATTTTTTTGCCTGATTCTATTGTTTTTTTCTTTTTTTCTCAGTGATGTCAACACAGGACTCGGTTAATACTGTTTATAAGTGCATGTTGAAAGGTGCGGCTGACTATCTAGTCAAGCCGTTGAGGAGGAATGAGCTGAGGAATCTCTGGCAACATGTCTGGAGAAGACAAAGTGTATGTCTCTTTCTTGCTATCTTTTTAAACCTTTTGTGTCCTTGAGCAATCTACAACTTAACGATCCTTATTCAAGTCCTGGGAAATTTCGTTGTCTATTAAAAAGATAATTGAGTTCTCAGATTCTTTTTTTCTTTTCTTGATCATCTTTACAGTCGCTTGCTCCTGGTAGTAACTTCCCAGTAGATGAGAGCCTTGGACAGCAGAAACCTGAGGGCGCGTCTGCAAACAACTCCACTAGTAACCAGGTGAATGGGTTTCAGAGAGAGGAACAACCTGTAATTGGGAATGGTGGTGGTGGTGGTGGTGATGATCAGGTTAGATTCTTATTCTCAGTGATGGATTCTCTTCACCTGTGTGTGTTTGATTGGTGTTCCTAACTTTTTTAGTTTAAAAATCTTGTTCTTGTAGAGCTCATGTTCAAGACCAGAGATGCAAGGTGAGAGCGCCGACGTGGTGGAGGATATCCCAAGAGTCTCATCCAAAGAGGCTATTGACTTCATGGGAGCGTCGTTTAGAAGAAACGGACAAAGCCACAGAGAAGAAAGTGTTGCTAAGCACGATACTTCTCGGATAGAGCTTGATCTCTCCTTGAGAAGACCTGACACTAGTGAGAACCACCAGCCCTCTCTTCATCCCTCTAGTGCCTCGGCTTTCACACGGTTAGTGGTCTGATCTCTAATTCCTTTTATAATCAATCTTTCAATACTTGTTACTGATTGTTTGTGTTGTGGTTCTTGTTGCCAGGTACGTTCACAGGCCATTGCAGACACAATGTTCAGTCTCTCCCTTGGTTACCGATCAGAGAAAGAACGTTGCAGCGAGTGGTGGAGACGATAACAACACTGTGCTAATCAACCAATACAATGTATCTGAACCGCCTCCAAGTGCTCAGAGAAGAAACGAGGCCAGCTTTTACAATAGCTCTGACTCGCCTGGTCCACCTTTTAGCAACCAGATGAATTCTTGGCCAGGACAGGGCTCTTACCCAACGCCAGCTCCTATTATACACTTCCCTGGTCCTAACCACACTTCTACCATGGCTCCCGCTTCAGTGTCTCCAAGCCCTAGCTCGGTTAGCCCGCATGAGTACAGTTCCATGTTTCACCCGTTCAACGGTAAACCCGAGGGCTTGCAAGAGCGGGATGGTTCCATGGATATGGAGGAGAGAAGACACGTCTCCTCTGCAACAGAACATAGTGGAACAGGCAATCACTGCAGTACCAACTACATAGATTATCATCATCAACAGCAGCAGCTTCTAGAGAAGAGGAGCGAAGAAGGATACTCCTCCTCTGTTGGGAAACTTCAGCAATCTCTGCAACGGGAAGCCGCTTTAAACAAATTCAGGATGAAGCGCAAGGAGAGGTGCTTTGAGAAAAAGGTATAAAACTTTGATGCTTAATAAACACATAACGGCTTTGTTAAGAACCAACGGCTTCTAAACCTTTGTTAATGACTTTTGCACACAGGTCCGTTATGAGAGCCGGAAGAAATTAGCAGAGCAACGGCCTCGAATCAAAGGGCAATTCGTTCGTCAAGTCCAGTCCACTGAGACCTCAACACAAGAAGCTCCACAATGA

>BrPRR4(Bra009768)-CDS

ATGGGAGAAGTGAGCGACGAAGTTGTTGAGGTGACGGTGGTGGAGAAAGCACCTGAGGCTGGCGGAGGAAAGTTAACGCGGCGGAAGATGCGGCGGAAGGACGCCGCGGAGGGCGGCGACGGTCTGGTGACGTGGGAGAGGTTTCTCCCGAAGATATCTCTCAGGGTTTTGCTCGTTGAAGCAGACGATTCCACCAGACAGATAATCTCTGCTCTCCTCAGGAAATGCAGTTACAGAGTCGCTGCTGTACCTGATGGGTTGAAAGCTTGGGAGATGCTTAGAGGGAAGCCTGAGAGCGTTGATCTGATATTAACAGAGGTTGATCTTCCTTCGATCTCCGGATACGCTCTGCTAACACTTATAATGGAGCATGACCTCTGCAAGAACATTCCTGTCATAATGATGTCAACACAGGACTCGGTTAATACTGTTTATAAGTGCATGTTGAAAGGTGCGGCTGACTATCTAGTCAAGCCGTTGAGGAGGAATGAGCTGAGGAATCTCTGGCAACATGTCTGGAGAAGACAAAGTTCGCTTGCTCCTGGTAGTAACTTCCCAGTAGATGAGAGCCTTGGACAGCAGAAACCTGAGGGCGCGTCTGCAAACAACTCCACTAGTAACCAGGTGAATGGGTTTCAGAGAGAGGAACAACCTGTAATTGGGAATGGTGGTGGTGGTGGTGGTGATGATCAGAGCTCATGTTCAAGACCAGAGATGCAAGGTGAGAGCGCCGACGTGGTGGAGGATATCCCAAGAGTCTCATCCAAAGAGGCTATTGACTTCATGGGAGCGTCGTTTAGAAGAAACGGACAAAGCCACAGAGAAGAAAGTGTTGCTAAGCACGATACTTCTCGGATAGAGCTTGATCTCTCCTTGAGAAGACCTGACACTAGTGAGAACCACCAGCCCTCTCTTCATCCCTCTAGTGCCTCGGCTTTCACACGGTACGTTCACAGGCCATTGCAGACACAATGTTCAGTCTCTCCCTTGGTTACCGATCAGAGAAAGAACGTTGCAGCGAGTGGTGGAGACGATAACAACACTGTGCTAATCAACCAATACAATGTATCTGAACCGCCTCCAAGTGCTCAGAGAAGAAACGAGGCCAGCTTTTACAATAGCTCTGACTCGCCTGGTCCACCTTTTAGCAACCAGATGAATTCTTGGCCAGGACAGGGCTCTTACCCAACGCCAGCTCCTATTATACACTTCCCTGGTCCTAACCACACTTCTACCATGGCTCCCGCTTCAGTGTCTCCAAGCCCTAGCTCGGTTAGCCCGCATGAGTACAGTTCCATGTTTCACCCGTTCAACGGTAAACCCGAGGGCTTGCAAGAGCGGGATGGTTCCATGGATATGGAGGAGAGAAGACACGTCTCCTCTGCAACAGAACATAGTGGAACAGGCAATCACTGCAGTACCAACTACATAGATTATCATCATCAACAGCAGCAGCTTCTAGAGAAGAGGAGCGAAGAAGGATACTCCTCCTCTGTTGGGAAACTTCAGCAATCTCTGCAACGGGAAGCCGCTTTAAACAAATTCAGGATGAAGCGCAAGGAGAGGTGCTTTGAGAAAAAGGTCCGTTATGAGAGCCGGAAGAAATTAGCAGAGCAACGGCCTCGAATCAAAGGGCAATTCGTTCGTCAAGTCCAGTCCACTGAGACCTCAACACAAGAAGCTCCACAATGA

>BrPRR5(Bra036517)-DNA

ATGCGAGAAAAGAGCGACGAAGTTGTTGAGGTCACGGTGGTGGAGAAAGCAGCTGAGGCTGTCGGAGTAAAGTCAGCGCGGCGGCGGAGAGTGCAGAGGAAGGACGCCGCAGAGGGGGGCGATGGTTTGGTGAAGTGGGAGAGGTTTCTCCCTAAAATCGCGTTGAGAGTTTTGCTAGTAGAAGCAGACGATTCCACCAGACAGATTATTTCTGCTCTCCTCAGGAAATGCAGTTACAGAGGTCAGTTGGCTTCTGTCTGAACCTTGACTGTTTTGTCGGAATCTATGTTGTCTGAAAATTTGTAACTGTAAAACTGATTAGAATGTGAAAGTCTTTTCCTTTTAATCGCAGATTAGTTTATTTCTTATAAAAAAAATGTTTTAGTGGTTTAATGTGGAACTGATTAGCATGTGAAAGTATTTTCCTTTTAATCTCGGATTAGTTTTTTGTTTCTCCAATAAGAATGTTTTTTTTTTTTTGAAAAAAAATATTTTTCCAATAAGAATGTTTCCTTTTAGTGTCAGCTTAATGTATTAATGTGGTTGGTTTTGTGTACTATGTTGTGTCTTCACAGTTGCTGCTGTACCTGATGGCTTGAAAGCTTGGGAGATGCTAAAAGGAAACCCCGAGAGCGTCGATCTGATACTCGCAGAGGTTGATCTTCCTTCAATATCTGGATACGCTCTTCTTACACTTATCATGGAGCATGATGTCTGCAAGAACATTCCTGTCATAAGTATGTGTACATTTATATAACTCATGCCTCCTTTTTTCATCGTTTTGAAATGATTTAATTTTTACATATATTACATAAATTATTTTATATTTTGACTTTAATTATAAAATTAATAAAAGAAATTAAACAGATTTGTGGATTTTAATAATTTTATTACTTAATTTAACAGATTTAAACTAATTTAAATCAGTTTCAACTAATTTTAGCCAATTTATAACTGTTTAAACCGATTGAAGACATTTAAGAGTTTAAATCGATCTCAATCCATTTATAACGGTTTAAACCGATTGAAGATATATAAATTGGATTTTAAGAAAATTGTTTATCGCTAGCGCCTGGACCGGTTTTTAGAACCATGATTTCAGCTCATTATATTTTTCTTGTATTGTTTGTTTCAGTGATGTCGACACATGACTCAGTGAATACTGTGTACAAGTGCATGTTGAAAGGTGCTGCTGACTATCTTGTTAAGCCGTTGAGGAGGAATGAGCTGAGAAATCTCTGGCAACATGTTTGGAGAAGACGACAAAGTGTATGTTCCATTCAAATATCTTTAACCTTTTTTGTGTCTCCCTGTGCAATCTATAATTGAGTTCTTGAATCTTCTTGGCAGACACTTGCTCCTGGTAGCTTTCAACTTGATGAGAGTCTCGGCCATCGGAAACCTGAGGGTGCTCAGGTTAGATTCTCTTCACAAGTGTACTTTGATTCTGTTCCTTAGTGTTTCTTTCTAAAACTTTTGTTCTTGTAGAGCTCATGTACAAGACCAGAGACGGAAGGAGAGAGCGCAGACGTGGAGAAAGACTCATCTAAAGAAGCCATTGACTTCATTGGAGCTTCGTTTACTAGAAACGAACAACACAACAGAGAAGAAAGTGTTAGGATAGAGCTTGATCTCTCCCTGAGAAGATCCTCTCTTCATCCTTCTAGTGGCTCTGCTTTCACAAGGTACGTTCACAAGCCGCTGCAGACACAGTGCTCCGTCTCTCCCTTGGTTCCCGACCAAAGAAAGAACGTTACGGAGAGTGAAGATGGTAACATTGTGGTAACCAATCAATACAAATCATCTGAACCGCCTCCAAGTGCTCCTAGAAGAAACGAGGCCAGCTTTTACAATAGTGCTGACTCACCTGGTCCACCTTCTTGGCCAGGACAAGGTTCTTACCCAACCACAGTTCCCATCAAGAGTATACAGTTCACAAGTCCTAACACAACTGCTGCTTCATTGTCTCCAAGCCCTAGCTCCATTAGCCCGCATGAGTACAGTTCCATGTTTCACCCATACAACGGTAATAAACCCGAGGGTTTGCAAGAGCAGGATGTAGAGGAGAGAAGACACGTCTCTTCTGCAAATGAACACAGCACAATAGGGAACCACTGCACTACCAGCTACATTCAGGATCAGCAGCTTGTGGAGAAGAAGAATGAAGAAGGGTACTCGTCCTCTGTAGGGAAGACTAAGCAATCTCTTAGGGAAGCTGCTTTAAACAAGTTTAGGATGAAGCGCAAGGACAGGTGTTTTGATAAAAAGGTGATGTTCAAACTCATGACAACACACATAAGGGCTTTGTTAAGAGGGATGGTTCTAACGGGTTTTAAAACCTTTTGTAATGACTTCTGCATAGGTTCGTTATGAGAGCAGGAAGAAACTGGCAGAGCAACGGCCGAGAATTAAAGGGCAGTTCGTTCGTCAAGTCCAATCCACTGAGACCTCAACGCAACAAGCTCCACAATGA

>BrPRR5(Bra036517)-CDS

ATGCGAGAAAAGAGCGACGAAGTTGTTGAGGTCACGGTGGTGGAGAAAGCAGCTGAGGCTGTCGGAGTAAAGTCAGCGCGGCGGCGGAGAGTGCAGAGGAAGGACGCCGCAGAGGGGGGCGATGGTTTGGTGAAGTGGGAGAGGTTTCTCCCTAAAATCGCGTTGAGAGTTTTGCTAGTAGAAGCAGACGATTCCACCAGACAGATTATTTCTGCTCTCCTCAGGAAATGCAGTTACAGAGTTGCTGCTGTACCTGATGGCTTGAAAGCTTGGGAGATGCTAAAAGGAAACCCCGAGAGCGTCGATCTGATACTCGCAGAGGTTGATCTTCCTTCAATATCTGGATACGCTCTTCTTACACTTATCATGGAGCATGATGTCTGCAAGAACATTCCTGTCATAATGATGTCGACACATGACTCAGTGAATACTGTGTACAAGTGCATGTTGAAAGGTGCTGCTGACTATCTTGTTAAGCCGTTGAGGAGGAATGAGCTGAGAAATCTCTGGCAACATGTTTGGAGAAGACGACAAAGTACACTTGCTCCTGGTAGCTTTCAACTTGATGAGAGTCTCGGCCATCGGAAACCTGAGGGTGCTCAGAGCTCATGTACAAGACCAGAGACGGAAGGAGAGAGCGCAGACGTGGAGAAAGACTCATCTAAAGAAGCCATTGACTTCATTGGAGCTTCGTTTACTAGAAACGAACAACACAACAGAGAAGAAAGTGTTAGGATAGAGCTTGATCTCTCCCTGAGAAGATCCTCTCTTCATCCTTCTAGTGGCTCTGCTTTCACAAGGTACGTTCACAAGCCGCTGCAGACACAGTGCTCCGTCTCTCCCTTGGTTCCCGACCAAAGAAAGAACGTTACGGAGAGTGAAGATGGTAACATTGTGGTAACCAATCAATACAAATCATCTGAACCGCCTCCAAGTGCTCCTAGAAGAAACGAGGCCAGCTTTTACAATAGTGCTGACTCACCTGGTCCACCTTCTTGGCCAGGACAAGGTTCTTACCCAACCACAGTTCCCATCAAGAGTATACAGTTCACAAGTCCTAACACAACTGCTGCTTCATTGTCTCCAAGCCCTAGCTCCATTAGCCCGCATGAGTACAGTTCCATGTTTCACCCATACAACGGTAATAAACCCGAGGGTTTGCAAGAGCAGGATGTAGAGGAGAGAAGACACGTCTCTTCTGCAAATGAACACAGCACAATAGGGAACCACTGCACTACCAGCTACATTCAGGATCAGCAGCTTGTGGAGAAGAAGAATGAAGAAGGGTACTCGTCCTCTGTAGGGAAGACTAAGCAATCTCTTAGGGAAGCTGCTTTAAACAAGTTTAGGATGAAGCGCAAGGACAGGTGTTTTGATAAAAAGGTTCGTTATGAGAGCAGGAAGAAACTGGCAGAGCAACGGCCGAGAATTAAAGGGCAGTTCGTTCGTCAAGTCCAATCCACTGAGACCTCAACGCAACAAGCTCCACAATGA

>BrPRR6(Bra029407)-DNA

ATGCAAGAAATGAGCGACGAAGTTGTTGAGGTAACGGTGGTTGATAAAGCATCTGAAGCTGACGGAGGAAAGCCAACGCGGCGGAGGATGCGGAGGAAGGACGCCGCAGAGGGCGGCGATGGTTTGATGAAGTGGGAGAGGTTTCTTCCGAAAATCTCGCTAAGAGTTTTGCTCGTGGAAGCAGACGATTCTACCAGACAGATTATCTCTGCTCTCCTCAGGAAATGCAGTTACAGAGGTCAGTTTCTTGTTTCACTGTCGTGTTACTTTGAGCTTGCCTGATGGATTGTTTAAAGGGAATCTCTTTGTCTGAAAATCTGATTAAGTAATTAGAAGCTTCTCCAACATGTGGAAAGACTTATTCTTTTAATCTAAGCTGAGTTTTTTTTTTTCTTTCTATGTGGTCTTCTCAGTTGCTGCTGTAGCTGATGGCTTAAAAGCTTGGGAGATGCTAAAAGGAAATCCCCAAAGCGTTGATCTGATATTGACAGAGGTTGATCTACCTTCAATCTCTGGATACGCTCTTCTAACACTCATCATGGAGCATGATATCTGCAAGAACATCCCTCTCATAAGTATGTTATATTTTTATATATATCTCCTGCTTTTCTTTTTCTCATGTTTTCAGCTTCTTAACTTGTTATTGTTTTCTTTCTTTCAGTGATGTCGACGCAGGACTCGGTGAATACCGTGTATAAGTGTATGTTGAAAGGCGCGTCTGACTATCTTGTTAAGCCCTTGAGGAGGAACGAGCTAAGGAATCTCTGGCAACATGTCTGGAGAAGAAGACAAAATGCTCCTGGTAGCTTTCCACTAGATGAGAGTGTTGGTCATGAGAAGCCTGACGGTGCGTCTGCAAACAATTCGACTAGTAACCAGGAGAATGCATTTGAGAGAGACCAACGTCCTGTAATAGGGAATGGTGGTGATGACCAGGTTAGATATGGTTTCATTGCAGCAGCTAAGTTCCTCATTCTGTTTGTGTTTGTTTCTAAACTTTTTAGTTAATGTCTTTTCTTGTAGAGCTCATGTTCTAGACCAGAGATGCAAGGAGAGAGCGCAGACGTGGAGGACTCAACTAAAGAAGCCATTGACTTCATGGGCGCGTCGTTTAGAAGAAACACACAACGCAACAGAGAAGAAAGTGTTGCTAGATACGAATCTCTGATAGAGCTTGATCTTTCTTTGAGAAGACCTAACACTTGTGAGAACCAATCTTCAGGAGAAAAGCCCTCTCTTCATCCTTCTAGTGCCTCTGCCTTCACACGGTTAGTTCTGGTTCCTTCTTTCTATCAGTGTTTCATCACTTGTCGTTCTTTCAGCTGATTTTGTCTCCTATGTTCTTGTTGCCAGGTACGTTCACAGGCCACAATATTCGGCGTCTCCTTTGGTTTTGGATCAAAGAAAGAATGTTGCAGCAAGTGAAGATGATAACATCAACCAATACAATTCATCTGAACCACCTCCAAATGCTCCGAGAAGAAACGAGGTCAGCTTTAACAATAGAGCTGACTCATCTCAGATGAATTCTTGGCCAGGACAGGGTTCTTACCCAACACCAGTTCCCATCAACAGCATACAGTTCAACACAGCTGCTATGGCTCCTGCTTCACTGTCTCCAAGCCCGAGCTCCGTTAGCCCGCATGAGTACAGCTCCATGTTTCACCCTTTCAACGGTTCCATGGATGCAGAGGAGAGAAGACACATCTCTTCTACAACCGAACATAGTGCAATAGACAATCACTGCAGTGCTAACTCATCGTCTGTGGGGAGAACTCAACAGTCTCTTCAACGGGAAGCTGCCTTAAACAAGTTCCGGATGAAACGCAAGGACAGATGTTTTGACAAAAAGGTATATAAAACTCATGACTACATAGTGGCTATGTTAAGGAGAATGATTCTAACCTTTTGGATGATACTTTGCACCAGGTCCGTTATGAGAGCCGGAAGAAACTGGCTGAGCAACGTCCGCGTATCAAAGGGCAGTTCGTTCGTCAAGTTCAATCCACTGAGACATCAACACAAGAAGCTCCACAATGA

>BrPRR6(Bra029407)-CDS

ATGCAAGAAATGAGCGACGAAGTTGTTGAGGTAACGGTGGTTGATAAAGCATCTGAAGCTGACGGAGGAAAGCCAACGCGGCGGAGGATGCGGAGGAAGGACGCCGCAGAGGGCGGCGATGGTTTGATGAAGTGGGAGAGGTTTCTTCCGAAAATCTCGCTAAGAGTTTTGCTCGTGGAAGCAGACGATTCTACCAGACAGATTATCTCTGCTCTCCTCAGGAAATGCAGTTACAGAGTTGCTGCTGTAGCTGATGGCTTAAAAGCTTGGGAGATGCTAAAAGGAAATCCCCAAAGCGTTGATCTGATATTGACAGAGGTTGATCTACCTTCAATCTCTGGATACGCTCTTCTAACACTCATCATGGAGCATGATATCTGCAAGAACATCCCTCTCATAATGATGTCGACGCAGGACTCGGTGAATACCGTGTATAAGTGTATGTTGAAAGGCGCGTCTGACTATCTTGTTAAGCCCTTGAGGAGGAACGAGCTAAGGAATCTCTGGCAACATGTCTGGAGAAGAAGACAAAATGCTCCTGGTAGCTTTCCACTAGATGAGAGTGTTGGTCATGAGAAGCCTGACGGTGCGTCTGCAAACAATTCGACTAGTAACCAGGAGAATGCATTTGAGAGAGACCAACGTCCTGTAATAGGGAATGGTGGTGATGACCAGAGCTCATGTTCTAGACCAGAGATGCAAGGAGAGAGCGCAGACGTGGAGGACTCAACTAAAGAAGCCATTGACTTCATGGGCGCGTCGTTTAGAAGAAACACACAACGCAACAGAGAAGAAAGTGTTGCTAGATACGAATCTCTGATAGAGCTTGATCTTTCTTTGAGAAGACCTAACACTTGTGAGAACCAATCTTCAGGAGAAAAGCCCTCTCTTCATCCTTCTAGTGCCTCTGCCTTCACACGGTACGTTCACAGGCCACAATATTCGGCGTCTCCTTTGGTTTTGGATCAAAGAAAGAATGTTGCAGCAAGTGAAGATGATAACATCAACCAATACAATTCATCTGAACCACCTCCAAATGCTCCGAGAAGAAACGAGGTCAGCTTTAACAATAGAGCTGACTCATCTCAGATGAATTCTTGGCCAGGACAGGGTTCTTACCCAACACCAGTTCCCATCAACAGCATACAGTTCAACACAGCTGCTATGGCTCCTGCTTCACTGTCTCCAAGCCCGAGCTCCGTTAGCCCGCATGAGTACAGCTCCATGTTTCACCCTTTCAACGGTTCCATGGATGCAGAGGAGAGAAGACACATCTCTTCTACAACCGAACATAGTGCAATAGACAATCACTGCAGTGCTAACTCATCGTCTGTGGGGAGAACTCAACAGTCTCTTCAACGGGAAGCTGCCTTAAACAAGTTCCGGATGAAACGCAAGGACAGATGTTTTGACAAAAAGGTCCGTTATGAGAGCCGGAAGAAACTGGCTGAGCAACGTCCGCGTATCAAAGGGCAGTTCGTTCGTCAAGTTCAATCCACTGAGACATCAACACAAGAAGCTCCACAATGA

>BrPRR7(Bra028861)-DNA

ATGAATGGTGATGAGAACGGTGAGGGTGAGGGGCATAGTGGAGGAGAAGATAAAGCCAATGGAGTCCCTATGGATGTGAGAAATGGGGCTGCTGGCCAAGGCTCTTCGCCTGGACTGCAAGTTCCACTGTCGCAGCAAACGCAGGCCACTGTTTGTTGGGAAAGGTTTCTTCATGTCAGAACCATCAGAGTGCTTCTCGTGGAAAATGATGACTGTACTCGTTATATCGTTACTGCCCTTCTTCGTAATTGTAGCTATGAAGGTCGGTCAGTTCTCAAGACTATGACTTTACTTGTTGCATACCACTAATAGATAATGAGAAAGCAACTTTCATTTATGTCAGTTGTGGAGGTAGCGAATGGTGTACAAGCTTGGTAGGTGTTGGAAGATCTAAACAATCATATTGATATTGTGTTGACCGAGGTGGTCATGCCTTACTTATCTGGTGTCGGTCTCTTATGCAAGATTTTGAATCACAAATCTCGCCGCAACATCCCTGTCATCAGTGAGTGTTTTCTCTCTTCTAGTTGATTCTTCTCAAGCTTCCGGATCTGTTGGTGAAGTAGATGCTTTTAACTACATTCCCCTGTGAGTGTTTTTCTTTTGTTGTTGCAGTGATGTCGTCTCATGACTCAATGGGGCTGATCTTTAAATGCTTATCAAAAGGAGCGGTTGACTTTCTCGTTAAGCCTATAAGAAAAAATGAGCTTAAAATCCTTTGGCAACATGTTTGGAGAAGATGTCAAAGTGTAAGTCCCCGTTTGCTTGTATCTGAAAAAATTGTTGGTATTTATTTTATCTAATTGTGAATTGGTATCTATCTTTTGGTTTTCCTTGTTTCATGTTCACAGTCAAGTGGCAGTGGAAGTGAAAGTGGAACTCATCAAACTCAAAAGTCCGTGAAATCCAAGAATATTATAAAATCTGACAACGATTCAGGACACAGTGGTGAGAATGAGAATGGGAGCATTGGCCTGAACGCTAGTGATGGAAGTAGTGATGGGAGTGGTGCTCAGGTGAGAAAAAATGCGCAAAGATTTCTAGTTTATTCCGAAGTTTTACTTTGCAAACGATCAGGATTTGATTTTGATATATCTTTTCTTCAGAGCTCTTGGACAAAAAAAGCTGTGGAAGTTGATGACAGTCCACGAGCGGTGTCTCTATGGGATAGAGCTGATAGCACATGCGCACAAGTGGTACATTCAAACCCCGAGTTTCCCAATAATCACTTGGTTGCAGCACCTGCTGAGAAGGAGACTCAAGAACAGGATGAAAAAATTGGTAAAGAAAGGAAGCTGTGATCTCTCAGTTTCTTCCGTACTCTTCTTTATATTCAAACTTAAAAACATGATAGTGAACATTTTTTTTCCATAATTTCTGCAGAAGATGTCACAATGGGTAGAGACTTGGAGATTAGTATTCGTAGAAATGATGATCCCAAAGATGAACCCCTAACTAAAACCACTGGCATCGTGAGACAGGAGAATTCATTTGAGAAGAGCTCTAGTAAATGGAAACTGAAAGTTGGAAAAGGACCGTTGGATCTCAACAGTGAAAGTCCTTCAAGTAAACAAATGCATGAAGATGGAGGATCGGGTTTCAAAGCGTTGTCTAGCCACCTTCAAGATAACAGAGAACCGGAGGCGCCTAACCCACACTGCAAAACTTTAGATACAAGTGAAGCTGCCGTCAAAAACTCTGAAGAGCTGATGGACGTTGAACATAGTTCAAAGAGGCATCGTGGAACCAAAGATGATGGGGCAATAGTTAGAGAAGACCGCAATGTGCTGAGGCGTTCAGAGGGTTCAGCTTTCTCAAGGTACTTTTGTTTTTAATATATAACGTTAGAAGGTTGTATTGAAGGCTTATGAGATCGATTGTTGGACTTGTACAGGTATAATCCAGCCGCGAATAACAATAAGCTTCATGATAATAATTGCCAAGATATTACAAAAAAGACTGAAGCGGCATGTGATTGTCACCTGAACATGAACGAGGGTCTCCGCAATAATCATCACTCACGCGTCGGGAGCAATAACTTGGAAATGAGCTCTACGACTGGAGCTCCAAAAGTGAGCTCGGCAGGATCTTCATCAGTAAAGCACTCATCGATTCAGCCTCTTCTACCTTGTGATCATCGTAATCATCATCATCACTCGTCCTATAATCCTGCTCACATCCCTGAGCAGAAGTTACCGCCCCAACGTGGATCCTCAAATGTGTACAATGAGGTGATTGAAGGTAACAACAACAATAAAGTGAATTACAGTGTGAATGGAAGTGGATCAGGAAGTGGTCACGGGAGCAATGATCCATATGGAAGCAGCAATGGGATGAATGCTGGAGGAGGGATGAATACGGGTAGTGCTAATGGTGATGGTGGTGGAAGTGGCGATGGTAGTGGAAGTGGAAGTGGAAGTGGAAGCGGGAATGTGGCAGATGAAAACAAGATGTCTCAAAGAGAAGCTGCTTTGACCAAGTTCCGTCAGAAGAGAAAAGAGAGGTGCTTCAGAAAGAAGGTAATAAGTTAAAAGGCTTTTGATTGTCATTTCAAAACATTTGCATGTGTTAACTTAAACATGTAGTCATATGATTCATAAAAATGTGTTTTTCTTTTTCTCAGGTACGATACCAAAGCCGGAAAAAACTAGCGGAACAACGCCCTCGTGTCCGTGGCCAGTTCGTGCGTAAAACAGCTGATGCAACGAATGATAACGACACAAAAAACGTTGAGGATAGCTGA

>BrPRR7(Bra028861)-CDS

ATGAATGGTGATGAGAACGGTGAGGGTGAGGGGCATAGTGGAGGAGAAGATAAAGCCAATGGAGTCCCTATGGATGTGAGAAATGGGGCTGCTGGCCAAGGCTCTTCGCCTGGACTGCAAGTTCCACTGTCGCAGCAAACGCAGGCCACTGTTTGTTGGGAAAGGTTTCTTCATGTCAGAACCATCAGAGTGCTTCTCGTGGAAAATGATGACTGTACTCGTTATATCGTTACTGCCCTTCTTCGTAATTGTAGCTATGAAGTGATGTCGTCTCATGACTCAATGGGGCTGATCTTTAAATGCTTATCAAAAGGAGCGGTTGACTTTCTCGTTAAGCCTATAAGAAAAAATGAGCTTAAAATCCTTTGGCAACATGTTTGGAGAAGATGTCAAAGTTCAAGTGGCAGTGGAAGTGAAAGTGGAACTCATCAAACTCAAAAGTCCGTGAAATCCAAGAATATTATAAAATCTGACAACGATTCAGGACACAGTGGTGAGAATGAGAATGGGAGCATTGGCCTGAACGCTAGTGATGGAAGTAGTGATGGGAGTGGTGCTCAGAGCTCTTGGACAAAAAAAGCTGTGGAAGTTGATGACAGTCCACGAGCGGTGTCTCTATGGGATAGAGCTGATAGCACATGCGCACAAGTGGTACATTCAAACCCCGAGTTTCCCAATAATCACTTGGTTGCAGCACCTGCTGAGAAGGAGACTCAAGAACAGGATGAAAAAATTGAAGATGTCACAATGGGTAGAGACTTGGAGATTAGTATTCGTAGAAATGATGATCCCAAAGATGAACCCCTAACTAAAACCACTGGCATCGTGAGACAGGAGAATTCATTTGAGAAGAGCTCTAGTAAATGGAAACTGAAAGTTGGAAAAGGACCGTTGGATCTCAACAGTGAAAGTCCTTCAAGTAAACAAATGCATGAAGATGGAGGATCGGGTTTCAAAGCGTTGTCTAGCCACCTTCAAGATAACAGAGAACCGGAGGCGCCTAACCCACACTGCAAAACTTTAGATACAAGTGAAGCTGCCGTCAAAAACTCTGAAGAGCTGATGGACGTTGAACATAGTTCAAAGAGGCATCGTGGAACCAAAGATGATGGGGCAATAGTTAGAGAAGACCGCAATGTGCTGAGGCGTTCAGAGGGTTCAGCTTTCTCAAGGTATAATCCAGCCGCGAATAACAATAAGCTTCATGATAATAATTGCCAAGATATTACAAAAAAGACTGAAGCGGCATGTGATTGTCACCTGAACATGAACGAGGGTCTCCGCAATAATCATCACTCACGCGTCGGGAGCAATAACTTGGAAATGAGCTCTACGACTGGAGCTCCAAAAGTGAGCTCGGCAGGATCTTCATCAGTAAAGCACTCATCGATTCAGCCTCTTCTACCTTGTGATCATCGTAATCATCATCATCACTCGTCCTATAATCCTGCTCACATCCCTGAGCAGAAGTTACCGCCCCAACGTGGATCCTCAAATGTGTACAATGAGGTGATTGAAGGTAACAACAACAATAAAGTGAATTACAGTGTGAATGGAAGTGGATCAGGAAGTGGTCACGGGAGCAATGATCCATATGGAAGCAGCAATGGGATGAATGCTGGAGGAGGGATGAATACGGGTAGTGCTAATGGTGATGGTGGTGGAAGTGGCGATGGTAGTGGAAGTGGAAGTGGAAGTGGAAGCGGGAATGTGGCAGATGAAAACAAGATGTCTCAAAGAGAAGCTGCTTTGACCAAGTTCCGTCAGAAGAGAAAAGAGAGGTGCTTCAGAAAGAAGGTACGATACCAAAGCCGGAAAAAACTAGCGGAACAACGCCCTCGTGTCCGTGGCCAGTTCGTGCGTAAAACAGCTGATGCAACGAATGATAACGACACAAAAAACGTTGAGGATAGCTGA

>BrPRR8(Bra009565)-DNA

ATGAATGTTAATGAGGAGGGTGAGGGTTCGCGTTACCCAGTCACTGATCAGAAGCCCGTTGAGACGAAAGAGAGGCTTAGTGGAGAAGATAAAGCTAATGGAGTTGTTATGGATGTGAGAAACGGGAGTGCAGGAGGGGCTGGGGGTGGACTGCAAATTCCAATTTCGCAGCAAACACCGGCCACTGTTTGTTGGGAAAGGTTTCTTCATGTGAGAACAATAAGAGTGCTGCTGGTGGAGAATGATGACTGTACTCGTTATATAGTTACTGCGCTTCTTCGTAATTGTAGCTATGAAGGTCAGTTTTGAAGCCTATATGTGTATATATTTGCATCCATTAATTGATAATGAGAAGTAACTTCTTTTTTTATTTCATGGCAGTTGTTGAGGTAGCTAATGGTGTACAAGCTTGGAAGGTGTTGGAAGATCTAAACAATCATATTGATATTGTGTTGACGGAGGTAGTCATGCCTTACTTATCTGGTATCGGCCTCTTATGCAAGATCTTAAATCACAAATCCCGTCGGAACATCCCTGTCATTAGTGAGTTCTTTATCTCTCTTGTCGTTGTGCATCAGTCACTTCCCCTGTGATGCTTTTTAACTACATTCCCCTGTGAGATTTGTTGTTGCAGTGATGTCATCTCATGACTCAATGGGGCTGGTCTTCAAGTGCTTATCAAAAGGAGCTGTTGACTTTCTCGTTAAGCCCATAAGAAAAAACGAGCTTAAAATCCTTTGGCAACATGTTTGGAGAAGATGTCAGAGTGTAAGTGCTTTGCTCATATTTGAAACACTGTTGGGAGTTGTTTTATCTGTTTGATGTGATGAAGTCATTTTCAAATTATTGATTTCCTTGTTTTGTTGTTCACAGTCTAGCGGCAGTGGAAGTGAAAGCGGAACTCACCAAACTCAAAAGTCTGTGAAATCAAAGACTATTATGAAATCTGACAACGATTCAGGACGCAGTGGTGAGAATGAGAATGAGAGCAATGGCCTGAATGCTAGTGATGGAAGTAGTGATGGCAGTGGTGCTCAGGTTAGAAAAATGTGCAAATATCAATAATCTAATATATTATCTGATAAGTTGCCTTCCCCGTGATGAATTAAATGATGTTAACCAATTCTGAAGTTTTATTTGTAAAATCTAGATTTGTTTTTGATATATATATATTTTTTTTTTTTTCTTCAGAGCTCTTGGACGAAAAAAGCTGTGGAGGTTGATGATGACAGTCCACGAGCGGTATCTCCATGGGATCGAGTTGATAGCACTTGCGCACAAGTGGTACATTCCAACCCCGAGGTTCCCGGTAATCACTTGATCGCAGCACCTGCTGAGAAGGAGACTCAAGAACAGGATGAAAAATTTGGTAAAGAAAAGAAGTTGTGATCTCTCAGTTTCTTCCATACTCTTCTTTATCACAAACTTTAAAATATGTTATTGAATAAATCTTTTAAATAATTTTTGCAGAAGATATCACAATGGGTAGAGACTTGGAGATTAGTATACATGGAAATTGTGATCTGACACTGGAGCCAAAAGATGAACCCTTAACCAAAAGCACTGGCGTTGGAAAGGGACCTTTGGACCTCAATAGTGAAAGCCGTTCAAGTAAACAAATGCATGAAGACGGAGGCTCGGGTTTCAAAGCTACGTCTGGTCATCAACTTCAAGATAACAGAGAACCCGAGGCACCTACTACCACACACTGCAAAACTGTAGACACCAATGAAGCTGCCATCAAAAACCCCGAAGAGCCAATGCACGTTGAACATAGTTCAAAGAGGCATAGAGGAGCTAAAGATGATGAGACAATAGTTAGAGATGACCGCAATGTGCTGAGGCGTTCAGAGGGTTCAGCTTTCTCAAGGTACTTACTGTAGAATCATAAACAAATCCTAAAAGTTCTGTTTTTATATATAATGTTATATTGTTGGACTTGAACAGGTATAATCCAGCCTTAAACAACAATAAGCTTTCTGGCGGGAACTTGGGAAGCAATGCTCGGCATGATAATAATTGCCAAGAACTTATAAAAAGGACTGAAGCGGCATGTGATTGTCACTCAAACATGAACGAGAGTCTCCCTAGCAATCATCACTCCCGCGTCGGTAGCAATAACGTGGAAATGAGTTCCACAACTGTGAACAACGCTTTCACAAAGCCTGGAGCTCCAAAAGTAAGCCCGGCAGGATCTTCATCAGCAAAGCGTTCATTGTTTCAGCCTCTACCGTGTGATCATCATCACTCCTCGCATAATCTTGTTCACGTCCCTGAGCGGAAGTTACCACCACAATATGGATCGTCTAATGTGTACAATGAGACGATTGAAGGTAACAACAACAACAACAACACAGTCAATTACAGTGTGAATGGAAGTGGATCAGGTAGTGGTCATGGAAGCAATGACCCATATGGAAGCAGTAATGGCATGAATGCTGGAGGAGTGAACATGGGAAGTGAAAATGGTGCTGGCAAAAGCGGAAGTGGTGATGGTAGCGGAAGTGGAAGTGGGAATGTAGCAGATGAGAATAAGATCTCTCAAAGGGAAGCTGCTTTGACAAAGTTCCGTCAGAAGAGAAAGGAGAGGTGCTTCAGAAAGAAGGTAATTTGATTGTTAGATACTTGTGTGTTTATGTATTATAGTCATCATTTAAGATACACAGTATATAACGCAGGAAACTTAAAGATATAAATCCATTGTATTTGAAAATATTGACAATTGTTGCCTTAGTGGGTGATAGATAAAAGTGGTTTGTTATTTTTACTCAGGTGCGATACCAAAGCAGGAAAAAACTAGCGGAACAACGACCTCGTGTCCGTGGCCAGTTCGTCCGAAAAACAGCTGCTGCAACGGATGATAACGACGTAAAAACGCCGAGGGATAGCTAA

>BrPRR8(Bra009565)-CDS

ATGAATGTTAATGAGGAGGGTGAGGGTTCGCGTTACCCAGTCACTGATCAGAAGCCCGTTGAGACGAAAGAGAGGCTTAGTGGAGAAGATAAAGCTAATGGAGTTGTTATGGATGTGAGAAACGGGAGTGCAGGAGGGGCTGGGGGTGGACTGCAAATTCCAATTTCGCAGCAAACACCGGCCACTGTTTGTTGGGAAAGGTTTCTTCATGTGAGAACAATAAGAGTGCTGCTGGTGGAGAATGATGACTGTACTCGTTATATAGTTACTGCGCTTCTTCGTAATTGTAGCTATGAAGTTGTTGAGGTAGCTAATGGTGTACAAGCTTGGAAGGTGTTGGAAGATCTAAACAATCATATTGATATTGTGTTGACGGAGGTAGTCATGCCTTACTTATCTGGTATCGGCCTCTTATGCAAGATCTTAAATCACAAATCCCGTCGGAACATCCCTGTCATTATGATGTCATCTCATGACTCAATGGGGCTGGTCTTCAAGTGCTTATCAAAAGGAGCTGTTGACTTTCTCGTTAAGCCCATAAGAAAAAACGAGCTTAAAATCCTTTGGCAACATGTTTGGAGAAGATGTCAGAGTTCTAGCGGCAGTGGAAGTGAAAGCGGAACTCACCAAACTCAAAAGTCTGTGAAATCAAAGACTATTATGAAATCTGACAACGATTCAGGACGCAGTGGTGAGAATGAGAATGAGAGCAATGGCCTGAATGCTAGTGATGGAAGTAGTGATGGCAGTGGTGCTCAGAGCTCTTGGACGAAAAAAGCTGTGGAGGTTGATGATGACAGTCCACGAGCGGTATCTCCATGGGATCGAGTTGATAGCACTTGCGCACAAGTGGTACATTCCAACCCCGAGGTTCCCGGTAATCACTTGATCGCAGCACCTGCTGAGAAGGAGACTCAAGAACAGGATGAAAAATTTGAAGATATCACAATGGGTAGAGACTTGGAGATTAGTATACATGGAAATTGTGATCTGACACTGGAGCCAAAAGATGAACCCTTAACCAAAAGCACTGGCGTTGGAAAGGGACCTTTGGACCTCAATAGTGAAAGCCGTTCAAGTAAACAAATGCATGAAGACGGAGGCTCGGGTTTCAAAGCTACGTCTGGTCATCAACTTCAAGATAACAGAGAACCCGAGGCACCTACTACCACACACTGCAAAACTGTAGACACCAATGAAGCTGCCATCAAAAACCCCGAAGAGCCAATGCACGTTGAACATAGTTCAAAGAGGCATAGAGGAGCTAAAGATGATGAGACAATAGTTAGAGATGACCGCAATGTGCTGAGGCGTTCAGAGGGTTCAGCTTTCTCAAGGTATAATCCAGCCTTAAACAACAATAAGCTTTCTGGCGGGAACTTGGGAAGCAATGCTCGGCATGATAATAATTGCCAAGAACTTATAAAAAGGACTGAAGCGGCATGTGATTGTCACTCAAACATGAACGAGAGTCTCCCTAGCAATCATCACTCCCGCGTCGGTAGCAATAACGTGGAAATGAGTTCCACAACTGTGAACAACGCTTTCACAAAGCCTGGAGCTCCAAAAGTAAGCCCGGCAGGATCTTCATCAGCAAAGCGTTCATTGTTTCAGCCTCTACCGTGTGATCATCATCACTCCTCGCATAATCTTGTTCACGTCCCTGAGCGGAAGTTACCACCACAATATGGATCGTCTAATGTGTACAATGAGACGATTGAAGGTAACAACAACAACAACAACACAGTCAATTACAGTGTGAATGGAAGTGGATCAGGTAGTGGTCATGGAAGCAATGACCCATATGGAAGCAGTAATGGCATGAATGCTGGAGGAGTGAACATGGGAAGTGAAAATGGTGCTGGCAAAAGCGGAAGTGGTGATGGTAGCGGAAGTGGAAGTGGGAATGTAGCAGATGAGAATAAGATCTCTCAAAGGGAAGCTGCTTTGACAAAGTTCCGTCAGAAGAGAAAGGAGAGGTGCTTCAGAAAGAAGGTGCGATACCAAAGCAGGAAAAAACTAGCGGAACAACGACCTCGTGTCCGTGGCCAGTTCGTCCGAAAAACAGCTGCTGCAACGGATGATAACGACGTAAAAACGCCGAGGGATAGCTAA

>BrPRR9(Bra004507)-DNA

ATGGCGGAGACGATGGTGAGCAGAATAAAGTCGCCGGAGGTTGTACAGTGGGAGAAGTACCTTCCCAAAACGGTGCTTAAGGTTTTACTCGTCGAGTCCGATGACTCAACTCGACAAATCATCACCGCCCTTCTTCAAAAATGCTCTTACAAAGGTCGTTGCTTTACTCTGTTTTTATATTTTAAAAGTTTCCATCTTTGTATCTGATTTTGAATTTTCAAAAACAGTTGTAGCTGTGTCCGATGGTTTAGCTGCGTGGGAGACACTAAAGGAGAAGTCAAATGAGATTGACCTTATACTAACGGAGCTTGATTTGCCAGCTATATCTGGTTTTGCGTTACTTGCTTTGGTGATGGAGCATGAAGCTTGCAAGAACATTCCTGTCATAAGTACGATGGTGATCCCCTCAAGTCCGTTCTTTATTATCTTTTGTAATCTTTAGTCTTTGTTGTTCTGATGTTTTTTTTTGTTTTTTAGTGATGTCGTCGGAAGATTCGATGACGATGGTGTTGAAGTGTATGCTTAAAGGCGCTGCTGATTATCTGATTAAGCCAATGAGGAAGAACGAGTTGAAGAATCTATGGCAACATGTGTGGAGAAGACTTGCTGTAAGTTGTTTTTTTAGTTATGTTTCTAATTTTGTCTGGTTTCTAAAAGTTTGGAAGACTTATTATTGTGTGTTTTGGGTTATGAATAATCAGGTGCGTGATGGTCACAATGGTCACGGTCTTAGCTTACCAGCTTCACAGCAGAACCTTGAAGATAGTGATGAAACTAGTGCAGATCATTCTGATCAAGGAAGTGGTGCTCAGGCTACAACAAGCTGCTACGGTAACAATAAGCTAATGGAGGATGTGACAATGGATTTGATCGGTGGAATTGACAAACGGGCTGAGTGTTTCTACGGAGACAACACTCGTGATGAGTATGTTGGTCCGGAGCTTGGACTTTCTCTGAAAAGATCTTGCTCTGGAAGTTTCGAGAAGCAAGATCAAACCACCAAGCAACAAAAGCTTAGCCTCTCCGATGAATCAGTAGGCATTACATTAACTTGTAGATATGAGAACAGCCAGGCAGCAGAGAAGGGAGAGGTTGGTGTGGAGCCGAGTAGCTCAGGTGAGCCCAAGACACCAAGTGAATCACATGAGAAGCTAAGATTTGATTACGGAAGCGCTACAACGAGCAGCAACCACGAGAACATGGGGTCATCGAGTTTAAGCGGACAAAATGAGTTAAGCTTCCGTAACCAAGTTGGTTCTGAAAGCACCAATGATGTGAAAGCAAAGGAACAGGAAGAAGAAGGTTGTGGTCTGAGTGTAGAGCAACGTCGGAGTCAGAGAGAAGCTGCGTTGATGAAGTTTCGGTTGAAGAGGAAAGATCGATGCTTTGACAAAAAGGTAAGGTATCAGAGCAGGAAGAAGCTAGCAGAGCAGCGTCCACGAGTGAAAGGCCAGTTCGTGCGTGCAGTGATGAACTCAGATGCGTCTAAATAA

>BrPRR9(Bra004507)-CDS

ATGGCGGAGACGATGGTGAGCAGAATAAAGTCGCCGGAGGTTGTACAGTGGGAGAAGTACCTTCCCAAAACGGTGCTTAAGGTTTTACTCGTCGAGTCCGATGACTCAACTCGACAAATCATCACCGCCCTTCTTCAAAAATGCTCTTACAAAGTTGTAGCTGTGTCCGATGGTTTAGCTGCGTGGGAGACACTAAAGGAGAAGTCAAATGAGATTGACCTTATACTAACGGAGCTTGATTTGCCAGCTATATCTGGTTTTGCGTTACTTGCTTTGGTGATGGAGCATGAAGCTTGCAAGAACATTCCTGTCATAATGATGTCGTCGGAAGATTCGATGACGATGGTGTTGAAGTGTATGCTTAAAGGCGCTGCTGATTATCTGATTAAGCCAATGAGGAAGAACGAGTTGAAGAATCTATGGCAACATGTGTGGAGAAGACTTGCTGTGCGTGATGGTCACAATGGTCACGGTCTTAGCTTACCAGCTTCACAGCAGAACCTTGAAGATAGTGATGAAACTAGTGCAGATCATTCTGATCAAGGAAGTGGTGCTCAGGCTACAACAAGCTGCTACGGTAACAATAAGCTAATGGAGGATGTGACAATGGATTTGATCGGTGGAATTGACAAACGGGCTGAGTGTTTCTACGGAGACAACACTCGTGATGAGTATGTTGGTCCGGAGCTTGGACTTTCTCTGAAAAGATCTTGCTCTGGAAGTTTCGAGAAGCAAGATCAAACCACCAAGCAACAAAAGCTTAGCCTCTCCGATGAATCAGTAGGCATTACATTAACTTGTAGATATGAGAACAGCCAGGCAGCAGAGAAGGGAGAGGTTGGTGTGGAGCCGAGTAGCTCAGGTGAGCCCAAGACACCAAGTGAATCACATGAGAAGCTAAGATTTGATTACGGAAGCGCTACAACGAGCAGCAACCACGAGAACATGGGGTCATCGAGTTTAAGCGGACAAAATGAGTTAAGCTTCCGTAACCAAGTTGGTTCTGAAAGCACCAATGATGTGAAAGCAAAGGAACAGGAAGAAGAAGGTTGTGGTCTGAGTGTAGAGCAACGTCGGAGTCAGAGAGAAGCTGCGTTGATGAAGTTTCGGTTGAAGAGGAAAGATCGATGCTTTGACAAAAAGGTAAGGTATCAGAGCAGGAAGAAGCTAGCAGAGCAGCGTCCACGAGTGAAAGGCCAGTTCGTGCGTGCAGTGATGAACTCAGATGCGTCTAAATAA

>BrPRR10(Bra040484)-DNA

ATGGGGGAGGTTGTGGTTTTGAGCAGCGACGAAGGTACTATGGAGACGATGATGAGCAGAGGCAAGTCATCGGAGGTCGTCCGGTGGGAGAAGTATCTTCCGACAACGGTGCTTAGGGTTTTGCTTGTCGAGTCCGATGATTCAACTCGCCAAATCATCACCGCCCTTCTTCAGAAATGCTCTTACAAAGGTCATTTCTTTACTCTGTTTTAATAGGATGTTTTAAAGTTTCCTTCTTTGTCTTGATTTTGAACTCAAAAAGTATGTCCGGTCTTAATCTTAATCTGTTTCAAGTATGTTCGGTCTGTGAATTATTTGTTCTTTGTCTTATTCTGTTTCAAGTGTCGTTCTTTTATGTCTTAATTTTGAAGTTACAAGTATCATGTTCGGTCTGCGTCATTGTCTTAATCTGTTTCAAGTTTCAATCTTTTGTCTTAATTTTGAAGTTACAAGTATGTTCTTGTCTGTGAATTATTTGTTCATTGTCTTAAATCTGTTACAAGTATGTTCGGTCTTTATATGTCTTAATTTTGAAGTTACAAGTATTATGTTCTTGTCTGTGAATTGTTTGTTCATTGTCTTAATCTGTTTCAAGTTTCGATCTTTATGTCTTAATTTTGAAGTTAGAAGTATTATGTTCGGTCCGTGTATTATTTGTTCATTGTCTTAAAATTTTGAAGTTAGAAGTATGTTTCTTGTCTGTGAAACTAAACTCGAGTTTTGTTTTGATTTCGAAACAGTTGTGGCTGTCTCCGATGGTTTAGCTGCGTGGGAGATTCTCAAGGAGAAGACACATAACATCGACCTAATACTAACGGAGCTCGATTTGCCAGCTATATCCGGTTTTGCTCTACTCGCATTGGTGATGGAGCACGAAGCTTGCAAGCACATTCCTGTCATAAGTACGATGTTGGTGATCCCCTAAGTCCGTTCTTTTGTAAAAAAAAAAGCTTTCTCTTTAGTTTTTGTTGTTCTGATGATTTTATTTGTTTTCTTCTAGTGATGTCGTCGCAAGATTCGATGACGATGGTGTTGAAGTGTATGCTTAGAGGTGCTGCTGATTATCTCATCAAGCCCATGAGGAAAAACGAGTTGAAGAATCTATGGCAACATGTCTGGAGAAGACTTACTGTAAGTTCATTTCGTTATGTTTAATTAATTTTAAGCTTGAACGAACGACTTATAATAATTTTTTTGGGTTATGAATCAGTTGCGTGGTGATCTTACTGCTAATGGTCCTAGCTTACCAGCTTCACAGCAGAACGTTGAAGAGAATGATGAAACTTGTGCAGATTCAAGATATCATTCTGATCATGGAAGTGGTTCTCAGGCTATCAGCGACAACGGTGAGGATAAGCTGATGGGGGATGTCAAACCGCTGTTTGAATCTTTTGATGTGACAATGGATTTGATCGGTGGGATAGACAAACGGAGTGAGTGTTACTATGGAGACAACGCACGTGAGCAGCATGTTGGGCCCGAGCTTGGGCTTTCTCTGAAGAGATCTTGCTCTGAAAGAAGTTTAGAGAAGAACCAAGATGAAAGCAAGCATCAGAAGATTAGCCTCTCTGATGCATCAGCCTTCTCAAGGTTTGTGTGCTTTCTTTGAATAAAAACAGAATGCCTTAAATAATAGATATTAATTATTTATAACTTGCAGATACGAGAATGGGAAGGCAGGAGAGAAAGCAGTTGTTGTAGCTGTAGAGGCAAGTAGTTCAGCTGAGCCCAAGACACCAAGCGAATCACATGAAAAGTTGTTCAGATGTGATCACGGAAGCGCTACAACGAGCAGCAACCATGAGAACATTGGTTCATCAAGCGTAAGCGGACACAACCAGTTTCTTCAGTCCGGGACTACGAAACAGAAGCAAGAATCTCTCTTCCCAGTAGAATCCAATCGCCCGAAGGCAAGCAAGGAAGTGGAAGTTGGTTGTCAGAGCACCAACGAGGGGACAGTAACAGCAGGAGGACAAAGTAGGAGCAGCAGCACAAGAGAGAAAGCAAAGGAGGAAGAAGAAGAAGGTGAAGGTGGTGTCACTGCCCAGCAACGCAAGAGTGAGAGAGAAGCTGCGCTGATGAAGTTCCGGATGAAGAAGAAAGATCGATGCTTTGGTAAAAAGGTAAGATATGAGAGCAGGAAGAAGCTAGCAGAGCAGCGTCCAAGAGTGAAAGGCCAGTTTGTGCGTGCTGTAAACTCAGATGCGTCTATTACTAAATAATGCCTCGCAGCCCAACGAGAGAAGTTTCCACACGGTGAAAGGCCCAGTTATAGAGAGACTCGTTGTAATATCAAGTGTGGCTTATTTTGTATTTGAGTGTTATTTTCTCTGCTAATCTTATTAGAATATTGGGGTAACTATTCCACTTACTATTAGATAGATCATCTCTATTATCTGCGAATGATCAAAGCTACGTTAAACCGTAACTCCAGATTTTCTGCAAGTTTTTTTTTTCCATATTGACTTGGCATTTAATGTAAGAGGACTATCAGTATTATCTTCTTCTTTTTTTCACTTAAATGGTAGTACTAGTAAATGATTTGGACATTATTTTCTTTGTGATTCAATTCAATGTATCCAAATATTTTTCCAATTTTGTATTTATTCTACTTTTACAATTTGTTTTCTTCTTCTCAAAACTGTGAACCAATAAAATTATTTCATTTAAAGCCGTGGAAAACCACTGAAAAGGCCCAATCATTTGAGAGTAAACCAGGTAATTAAAGCCCCAATCATTCGGTTGGATTAGTAAAGTAGTGCCAGAAAGACAGGAGAGCAATAATTACGGTGTAATAAGTAGAGTGGCGATGCCACGTGTATATAAAATTGACCGTGGAAAAGACACAACAAGAAAACAAAACCATAGAGAATAGAGATCTGTAAAGGAGGAGTACAAACAAGGATAAGAGAGAGAAAAAAAAAAAAGACCCCGGAAAGAAAAGGAAAAGAGAAGCTGCCCCCATCTCATCTCTCGTGGCCGCCTCATACTTTTCTATTCATAAATAAAAGGCTCCTTCCTTTTTCCTTGACATTCTTTAAAGGGCGAGGAGGAGTAGTGAATCTATCAAACCGGCAACTTCAGCCTCTCGACTCTCTCTCTGGTTAGTTCTTTTCTTTTTTACGTTTTGTACGCTCTGTGTTTGCTACCTTTATTATTATTATTATTATTCGTGTCGAAGTTGAAGAATCAGTCTTTTTTGCCGATGCATCTCTGTAGCTGTATACTATATCTGTTACAGTGTTTCTAGATTCTTCATTGTCACATCTGGAATTGGTCTGATTGTGACTCTTTCATACTTTTAATTAATTATTATAATGATTAGTCTGTTAATATTATTATATCGTATCTATGTGTTTGTGCTAGTAGAACCTCCTCCTGTACAGGACTAGTTGTGCGATCCATGTCTGATTTGTTCTTTTTTAGATTATAAAGTTGCTTTCTTTTTCTTTTTGCTTCAAACAGGTTTACCCAATTTCGGACAACTATATAGCTCCCTTCTTGTTCTTGTTCTTGTCTCATTCTTGCTTACCCCCTTTTATTTTTATTTTTTCAGATGGCGTCTTCAAGCCCCCAACATTGCCACATCATCGAGGTCAATCGAGGTAAATCCGCTGAAGAAAGCACAACAATTCTGGCAAGCAAAGCCTGTGGAGAAGCTCCCTGCGGCTTCTCAGATCTCAACAACGCTTCCGGCGACGCCCAAGAACGCAATGCCTCCATGCGCAAGCTCTGCATCGCCGTGGTGCTATGTCTTCTCTTCATGACCGTTGAAGTCTTCGGTGGCATCAAAGCTAACAGCTTAGCTATACTCACCGACGCAGCTCATCTTCTCTCTGACGTTGCTGCCTTCGCCATCTCCCTGTTCTCCCTGTGGGCTGCTGGCTGGGAAGCGACGCCGAGGCAGACTTATGGTTTTTTCAGGATTGAGATTTTGGGAGCTCTTGTCTCCATCCAGCTCATTTGGCTCCTCACTGGTATTCTTGTCTATGAAGCTATCATCAGACTTCTTAGTGAGACTAGTGAGGTTAATGGTTTCCTTATGTTCCTTGTTGCTGCTTTTGGGTTGCTTGTGAATATCATAATGGCTGTTCTGTTGGGACATGATCATGGTCACGGTCATGGCCATGGTCATGATCATCACAGTCATGGGGTGACGGTTACCACACATCATCACCATCATGGTCATGGAGAGGACAAGCATCATCATCACGCTCATGGGGATGAAGATGTGACTGAGCAGTTGCTGGAGAAATCAGAGAAGAGAAAGAGGAACATCAATGTCCAAGGAGCTTACCTCCATGTCCTTGGAGACTCAATCCAGAGCGTTGGTGTTATGATTGGAGGAGGTATCATCTGGTACAACCCGGAGTGGAAGATAGTTGATCTGATCTGCACGCTTGTCTTTTCCGTGATTGTCTTGGGGACGACCATCAACATGATCAGAAGCATTCTTGAGGTGTTGATGGAGAGCACGCCGAGAGAGATCGACGCTACGAAGCTGGAGAAGGGTTTGCTGGAGATGGAAGAAGTGGTGGCGGTTCATGAGCTTCACATTTGGGCTATCACGGTGGGGAAAGTGCTGCTTGCTTGCCATGTCAATATCACACCGGAGGCAGATGCTGATATGGTGCTTAACAAGGTCATTGATTACATCCGCAGGGAGTATAATATCAGTCATGTCACTATACAAATCGAGCGCTGA

>BrPRR10(Bra040484)-CDS

ATGGGGGAGGTTGTGGTTTTGAGCAGCGACGAAGGTACTATGGAGACGATGATGAGCAGAGGCAAGTCATCGGAGGTCGTCCGGTGGGAGAAGTATCTTCCGACAACGGTGCTTAGGGTTTTGCTTGTCGAGTCCGATGATTCAACTCGCCAAATCATCACCGCCCTTCTTCAGAAATGCTCTTACAAAGTTGTGGCTGTCTCCGATGGTTTAGCTGCGTGGGAGATTCTCAAGGAGAAGACACATAACATCGACCTAATACTAACGGAGCTCGATTTGCCAGCTATATCCGGTTTTGCTCTACTCGCATTGGTGATGGAGCACGAAGCTTGCAAGCACATTCCTGTCATAATGATGTCGTCGCAAGATTCGATGACGATGGTGTTGAAGTGTATGCTTAGAGGTGCTGCTGATTATCTCATCAAGCCCATGAGGAAAAACGAGTTGAAGAATCTATGGCAACATGTCTGGAGAAGACTTACTTTGCGTGGTGATCTTACTGCTAATGGTCCTAGCTTACCAGCTTCACAGCAGAACGTTGAAGAGAATGATGAAACTTGTGCAGATTCAAGATATCATTCTGATCATGGAAGTGGTTCTCAGGCTATCAGCGACAACGGTGAGGATAAGCTGATGGGGGATGTCAAACCGCTGTTTGAATCTTTTGATGTGACAATGGATTTGATCGGTGGGATAGACAAACGGAGTGAGTGTTACTATGGAGACAACGCACGTGAGCAGCATGTTGGGCCCGAGCTTGGGCTTTCTCTGAAGAGATCTTGCTCTGAAAGAAGTTTAGAGAAGAACCAAGATGAAAGCAAGCATCAGAAGATTAGCCTCTCTGATGCATCAGCCTTCTCAAGATACGAGAATGGGAAGGCAGGAGAGAAAGCAGTTGTTGTAGCTGTAGAGGCAAGTAGTTCAGCTGAGCCCAAGACACCAAGCGAATCACATGAAAAGTTGTTCAGATGTGATCACGGAAGCGCTACAACGAGCAGCAACCATGAGAACATTGGTTCATCAAGCGTAAGCGGACACAACCAGTTTCTTCAGTCCGGGACTACGAAACAGAAGCAAGAATCTCTCTTCCCAGTAGAATCCAATCGCCCGAAGGCAAGCAAGGAAGTGGAAGTTGGTTGTCAGAGCACCAACGAGGGGACAGTAACAGCAGGAGGACAAAGTAGGAGCAGCAGCACAAGAGAGAAAGCAAAGGAGGAAGAAGAAGAAGGTGAAGGTGGTGTCACTGCCCAGCAACGCAAGAGTGAGAGAGAAGCTGCGCTGATGAAGTTCCGGATGAAGAAGAAAGATCGATGCTTTGGTAAAAAGATGGCGTCTTCAAGCCCCCAACATTGCCACATCATCGAGGTCAATCGAGGTAAATCCGCTGAAGAAAGCACAACAATTCTGGCAAGCAAAGCCTGTGGAGAAGCTCCCTGCGGCTTCTCAGATCTCAACAACGCTTCCGGCGACGCCCAAGAACGCAATGCCTCCATGCGCAAGCTCTGCATCGCCGTGGTGCTATGTCTTCTCTTCATGACCGTTGAAGTCTTCGGTGGCATCAAAGCTAACAGCTTAGCTATACTCACCGACGCAGCTCATCTTCTCTCTGACGTTGCTGCCTTCGCCATCTCCCTGTTCTCCCTGTGGGCTGCTGGCTGGGAAGCGACGCCGAGGCAGACTTATGGTTTTTTCAGGATTGAGATTTTGGGAGCTCTTGTCTCCATCCAGCTCATTTGGCTCCTCACTGGTATTCTTGTCTATGAAGCTATCATCAGACTTCTTAGTGAGACTAGTGAGGTTAATGGTTTCCTTATGTTCCTTGTTGCTGCTTTTGGGTTGCTTGTGAATATCATAATGGCTGTTCTGTTGGGACATGATCATGGTCACGGTCATGGCCATGGTCATGATCATCACAGTCATGGGGTGACGGTTACCACACATCATCACCATCATGGTCATGGAGAGGACAAGCATCATCATCACGCTCATGGGGATGAAGATGTGACTGAGCAGTTGCTGGAGAAATCAGAGAAGAGAAAGAGGAACATCAATGTCCAAGGAGCTTACCTCCATGTCCTTGGAGACTCAATCCAGAGCGTTGGTGTTATGATTGGAGGAGGTATCATCTGGTACAACCCGGAGTGGAAGATAGTTGATCTGATCTGCACGCTTGTCTTTTCCGTGATTGTCTTGGGGACGACCATCAACATGATCAGAAGCATTCTTGAGGTGTTGATGGAGAGCACGCCGAGAGAGATCGACGCTACGAAGCTGGAGAAGGGTTTGCTGGAGATGGAAGAAGTGGTGGCGGTTCATGAGCTTCACATTTGGGCTATCACGGTGGGGAAAGTGCTGCTTGCTTGCCATGTCAATATCACACCGGAGGCAGATGCTGATATGGTGCTTAACAAGGTCATTGATTACATCCGCAGGGAGTATAATATCAGTCATGTCACTATACAAATCGAGCGCTGA

>BrPRR11(Bra013277)-DNA

ATGGTCTTTACCTCTAACGATATATCAAAATGGGAAAATTTTCCAAAGGGACTTAGAGTCCTTCTCCTCGACTGTGGCGACGGCATATCCGCCGCTGAGACGCGATCAAAGCTTGAGTCCATGGACTATATCGGTGAGCCCACTAATTTTCCTCTGCTTCTCCTCTCTTAAAATCTTTTACTTTTACTTTCGCTTTGTTTCATTAATGTTAAGTGTTACTTTAGCTGTTACATGTGACGAGACCGTTTTTTGTTCTTCACTGCCAATTAATGGAGATTATAGCATTATTATAAAATTAATACTGATATGTATATGGATTATAGTATGAAAAGCAACGTTTTAGTCGGTTGAAAATGGAAGACAGTAGTAAAGGCTGTGCAGTAATGTTTTGGAGTTTTGTTAGTTTACATAATAATTACATTTGTGGAATTTCGAAAGAGCTATTATTACATCTGAAAAACTCGCTGACTTGTCTTTAGCCTTTTCCTGAGTTTGCTATGTTTCTGTGTGTCAAGACTATGCATTCAATTTTTTGTACTTTTGAAACTTTCTTGCAGTCACTACGTTCACTGATGGAACCGAAGCTCTCTCTGCGGTTATCAAAAGCCTGGAGAGCTTCCACATTGCAATAGTGGAGGTGAATACAAGTGACGAGAACGAGAGTTTCAAGTTTCTTGAAGCTGCTAAAGACCACCTTCCTACTATAAGTTTGTGAACCTTACCTTTCAAATTTCTACAAAAAAAAATTAAAATAAATCTTTAAAAAACATGGATTTGTCTTTTTTCTTTACAGTGATTTCAAACGATCATTGCATCACAACTACGATGAAATGCATAGCGGTATGTGACCGCTACTTGTGTTTTTCTTTTTTTCCGTATAATGTATAAAAAATGTTTGGTTATGTGATGAGAATTGACAAGTTTTTTCCAGCTTGGTGCAGTTGAGTTCCTTCAAAAACCGCTCTCACCGGATAAACTAAAGAATATTTGGCAGCATGTCGTTCATAAGGTTCAGCTAGTTAAACTAAACCATTTATATACATTAATGTAGAATAGTACTCACCTCTTTTTTGTTGTTGTTAGGCATTTAACGATGGAGGAACTGACGTTTCTGAATCACTCAAGCCTGTTAAAGAATCTGTTGTCTCGATGCTTCATCTCGATACCGATATGACCATTGATGAGAAAGACTCAGCACCATTAACACCGCAACTAAAACAAGTTTCACGGTTACTAGACGGTGGCGATCGTCAAGAGAACATAAACTCCTCCACCGAGAAAGAGAATATGGAAGATCAAGACATCGGTGAATCCAAATCAGTCGACACTACAAATCATGACGACAATGTGATTGTCAAAGAAGAGAAATTAGATGGTGAAACCGGAGATACAAAAAGCGAGAAGACAGATTCGGTTAAGTTTCAGAAGAAAGAAGATGAGACTACTAAACATAACAATAAATCAACCGGGATTAAGAACTTGTCTGGTAACAAACCTAGTCGAAAGAAGGTAAGTGTAATGTTTCTCTTTAAACACTTCATAGTGGCTAAACGAAGAGCGGCACTTATTGATAAGCTCTCTTTCTTTTCAGGTGGATTGGACACAAGAGCTGCACAAGAAGTTTGTGCAAGCGGTAGACCAACTTGGTGTCGATCAAGCGATACCTTCGCGGATTCTCGAGCTGATGAAAGTAGACGGCTTAACTCGACACAATGTAGCTAGCCATCTTCAGGTAAATTAATTATCTGAATGCTTTGTAGAGATTCTTGTGAAATGGTTACGGAATCTTTATGTGTTCTTGAGCAGAAATTTCGGATGCATAGGAGGAATATTCTTCCAAAGGAGGATCATAACCATAGATATATACAATCTAGAGAGAACCATAGACAAATCCAACGGCAGTATAATGGTTTTCAGCAACAACACCGTCCTGTAATGGCTTATCCCGTTTGGGGTCTTCCCGGTGTTCATCCGCCTGGAGCAGTTCCGCCTCTGTGGCCGCCGCCACTACAGTCTTCCGGTCAACTGCCTCCGTGGCATTGGAGACCACCTTATCCAACGGTAACATTCTAAGTTAACAAGTAATTCAGATTTCATTACAACAAGAAAAACGTCATTAGCTACGATTTACTACACCGCTTAAACGTCTTTTAAGCTCTACGTTTAAATTGTTTTAAGTCGTCAAATTGTAAAACTATTCTTAGTTATTTAGAAAATGTTGTAATCTTATAATTGATTAGCTAAATCGTTTATTGATTGATATTATATACGATCGTGTGACAGGTGAACGTTAATGCATGGGGTTGCCCTATTGTACCGCCGGTAACCGGACCATTCAGTTCTCCTACGGTAACCGGAACATTTAGTACTCCACCGGCGACTCAGCTGGTAAGTGTAATAAATTATATAGAAATAACAAAATAGTTACATTAAACTAATAACATTCGGGTCGGGTTTGGTCAGGATGAGGAAATGGTTGATCAAGTGGTTAAAGAAGCGATCAGCAAACCGTGGCTGCCGTTACCGCTCGGGCTAAAACCACCATCCGCGGAGAGCGTTTTAGCTGAGCTCTCGCGTCAAGGCATCTCAGCCGTCCCTTCTTCTTCTTCCTCAATCAACGGCTCTCGTCGTCTCCGCTGA

>BrPRR11(Bra013277)-CDS

ATGGTCTTTACCTCTAACGATATATCAAAATGGGAAAATTTTCCAAAGGGACTTAGAGTCCTTCTCCTCGACTGTGGCGACGGCATATCCGCCGCTGAGACGCGATCAAAGCTTGAGTCCATGGACTATATCGTCACTACGTTCACTGATGGAACCGAAGCTCTCTCTGCGGTTATCAAAAGCCTGGAGAGCTTCCACATTGCAATAGTGGAGGTGAATACAAGTGACGAGAACGAGAGTTTCAAGTTTCTTGAAGCTGCTAAAGACCACCTTCCTACTATAATGATTTCAAACGATCATTGCATCACAACTACGATGAAATGCATAGCGCTTGGTGCAGTTGAGTTCCTTCAAAAACCGCTCTCACCGGATAAACTAAAGAATATTTGGCAGCATGTCGTTCATAAGGCATTTAACGATGGAGGAACTGACGTTTCTGAATCACTCAAGCCTGTTAAAGAATCTGTTGTCTCGATGCTTCATCTCGATACCGATATGACCATTGATGAGAAAGACTCAGCACCATTAACACCGCAACTAAAACAAGTTTCACGGTTACTAGACGGTGGCGATCGTCAAGAGAACATAAACTCCTCCACCGAGAAAGAGAATATGGAAGATCAAGACATCGGTGAATCCAAATCAGTCGACACTACAAATCATGACGACAATGTGATTGTCAAAGAAGAGAAATTAGATGGTGAAACCGGAGATACAAAAAGCGAGAAGACAGATTCGGTTAAGTTTCAGAAGAAAGAAGATGAGACTACTAAACATAACAATAAATCAACCGGGATTAAGAACTTGTCTGGTAACAAACCTAGTCGAAAGAAGGTGGATTGGACACAAGAGCTGCACAAGAAGTTTGTGCAAGCGGTAGACCAACTTGGTGTCGATCAAGCGATACCTTCGCGGATTCTCGAGCTGATGAAAGTAGACGGCTTAACTCGACACAATGTAGCTAGCCATCTTCAGAAATTTCGGATGCATAGGAGGAATATTCTTCCAAAGGAGGATCATAACCATAGATATATACAATCTAGAGAGAACCATAGACAAATCCAACGGCAGTATAATGGTTTTCAGCAACAACACCGTCCTGTAATGGCTTATCCCGTTTGGGGTCTTCCCGGTGTTCATCCGCCTGGAGCAGTTCCGCCTCTGTGGCCGCCGCCACTACAGTCTTCCGGTCAACTGCCTCCGTGGCATTGGAGACCACCTTATCCAACGGTGAACGTTAATGCATGGGGTTGCCCTATTGTACCGCCGGTAACCGGACCATTCAGTTCTCCTACGGTAACCGGAACATTTAGTACTCCACCGGCGACTCAGCTGGATGAGGAAATGGTTGATCAAGTGGTTAAAGAAGCGATCAGCAAACCGTGGCTGCCGTTACCGCTCGGGCTAAAACCACCATCCGCGGAGAGCGTTTTAGCTGAGCTCTCGCGTCAAGGCATCTCAGCCGTCCCTTCTTCTTCTTCCTCAATCAACGGCTCTCGTCGTCTCCGCTGA

>BrPRR12(Bra012623)-DNA

ATGGTCTTTACCGCCAACGATTTATCAAAATGGGAAAGTTTTCCAAAGGGACTTAGAGTTCTTCTCCTCGACTCCGCCGCCGAGACCCGATCGAAGCTCGAGTCCATGGACTATATTGGTGAGCAAACTACTCTTCCTCAGTTTCTTTCAAACCTTTTTCTGCTTTTGTTTGATTAGAAAAAAGTATATTTTGTTTTAAAAAAAGGCTTTATAAAAAATATTTAATGCCTCTGTATAAATAAATATATAATAAATCATATTTTCATATTTGTCATCTACTTGAGTGAGACCTTACGGTGTATAGGTTTACTAGTTTAATTTTAGAGTTACTTTAGCTCTTACATGTCCCGTGACGATTCCTTTTTTTTTTCAATATCCATTGATTTATAAGAAGATTGTTATGAAATTAATGTATAATGGATTATAGTAAAGAAAAGCAACGTTTTAGTTGGTTGAAAGTGGAAAACAGAAGCAGTAAAGGTTGTGCAGTTATATTTTGGAGTTTTGTTAGTGTTCATAATTACATTTTTGGAATTTTGAAAGAGCTATTACTGCATTTAAACAAACTCATTAACGTTACCCTTACACTGAGAATATGTGGTTTTCTCGGGTTTATTGAACCGGAATCCAAACTATACCAAAGCTCTTGTTTGTGCATTATTCAGTTCTTGATCTTCTTGGAACTATCTTGCAGTCACTACATTCTCTGATGAATCTGAAGCACTCTCTGCGGTTGTCAAAAGCCCGGAGAGCTTCCACATTGCAATCGTCGAGGTGAATACGAGTGCAGAGGATGAGAGTTTCAAGTACCTTGAAGCTGCCAAAGGCCTTCTTCCTACTATAAGTTTGTAAACTGTACCTTTGAAGTTTCTACGAATTTTTGCAATATGCTTTTTTGCTTCTTTTTTTTAAATGTGAATTTTGTCTTTATCTTTACAGTGATTTCGGACGACCATTGCATCGCAACTACAATGAAATGCATAGCGGTAAGTGACAGAAACTTCCATTTTGCTTAGGTGTATAAAAAAAATGTTGGTTATGTGATGAAGTGTTTTCCAGCTTGGTGCAGTTGAGTTCCTTCAAAAACCACTTTCACCGGAGAAACTAAAGAATATTTGGCAGCATGTCGTTCATAAGGTTCGGTTAGTTTATTATATTCTATCTATACATCTTACATAAACCATCTAAGAAAATGTAACATGTTTATTGTTTTCAGGCATTTAATGATGGAGGAGCTAGTGTTTCGGAATCACTTAAGCCCGTTAAAGATTCTGTTGTCTCGATGATTAATATCGATACCAATATGACAATCGATGAGAAAGATCCAGCACCATCAACCCCTCAGTTAAGGCAAGTTTCACGGTTACTAGACCAAGAGAACAGAAATTGCTCGGTGGAAAATGTAAACTCCCCTACCGAGAAAGAGAACACGGAGGATCATGACATCGGTGAATCCAAATCAGTCGACACTACAAATCATGAAGACAATGTGATTGTCAAAGAAGAGAATGGAGATGGGGAAAAGGAAGAAGAACAAGGCCAAACCGAAGAACATAAACAAGAAGAAGGTGAAACCGGAGATTCAGTTAATTTTAATAAGAAAGAAGATGAGACTAATAAACCTATTAATAAATCATCCGGGAACAAGAACTTGTCTAGTAACAAAGCTAGTCGAAAGAAGGTAGTGTCAGGTCTCTTTTTATGGTTCTTAGTTTCTATACGGCTCAGCCTCGTTATTGATATTTTTCCTTCAGGTGGATTGGACACAAGAGCTGCACAAGAAGTTTGTGCAAGCGGTAGAGCAACTTGGAGTCGATCAAGCGATACCTTCAAGGATTCTTGAATTGATGAAAGTAGATGGCTTAACTCGACATAATGTAGCTAGTCACCTTCAGGTATTTAACTCCAAAGTGTCGAATGTTTTTATATATATTTTTATGAAAATGTTACAAAATCTTGGGAATTCTTTGAGCAGAAATTTCGGATGCATAGGAGAAATATTCTTCCAAAGGATGATCATAACCATAGATGGATACAATCTAGAGAGAACCATAGACAAATCCAACGGCAATATAACGGTTTCCAGCAACAGCACCGTCCCGTAATGGCTTACCCCGTTTGGGGTCTTCCCGGTGTTCATCCGCCTGGAGCAGTTCCGCCTTTGTGGCCGCCGGCATTGCCGTCTGCCGGTCAACTACCTCCATGGCATTTTAAACCACCTTATCCAACGGTAACATCTCAAGTTCTCTACTTCTCTTTATATAAAGTCAAAGATTCGAGTAACAAGGAACAAAATCAGATTTATTACAACAAGAAACGTATAAAATTGTCATTGTTTAAGTAGTCAAAAATAATTTCAGTAGTAAAATTGTATAAGTAAAACTTAGTTATTTAGATAAGATTGTTTGACAAAAAAGTAAGTTAGATAAGATTTAGTGATGTAATGTTTAGAAATGATAGCTAAATCAATGATTGATGTTTAGAAATGTTATATTGTCTATTATCGTGTTTCAGTTGAATGGTAATACATGGGGTTACCCGGTTGGACCACCGGTAACCGGAACATTCTTTCCTCCTCCGATAACCGGAACATTCAGTACTCATCCGGCGAATCAGCTGGTAAATTTTCATAAATTCTCTATTTTGCGTTAGAAATAACTACATTAAAGTATTAATTTCCGGGTCGGTTTTGCGTTGAATTTGGGTCGGGTCAGGACGAGGAAATGATTGATCAAGTGGTTAAAGAAGTGATTAGCAAACCGGGGATGCCGCTACCGCTGGGACTGAAACCGCCATCACCCGAGAGCGTTTTAACTGAGCTTTCGCGTCAAGACATCTCGGCCGTTCCTTCTTCCTCTGCCTCTTGTCAAATCAACGGCTCTCCTCGTCTCCGCTGA

>BrPRR12(Bra012623)-CDS

ATGGTCTTTACCGCCAACGATTTATCAAAATGGGAAAGTTTTCCAAAGGGACTTAGAGTTCTTCTCCTCGACTCCGCCGCCGAGACCCGATCGAAGCTCGAGTCCATGGACTATATTGTCACTACATTCTCTGATGAATCTGAAGCACTCTCTGCGGTTGTCAAAAGCCCGGAGAGCTTCCACATTGCAATCGTCGAGGTGAATACGAGTGCAGAGGATGAGAGTTTCAAGTACCTTGAAGCTGCCAAAGGCCTTCTTCCTACTATAATGATTTCGGACGACCATTGCATCGCAACTACAATGAAATGCATAGCGCTTGGTGCAGTTGAGTTCCTTCAAAAACCACTTTCACCGGAGAAACTAAAGAATATTTGGCAGCATGTCGTTCATAAGGCATTTAATGATGGAGGAGCTAGTGTTTCGGAATCACTTAAGCCCGTTAAAGATTCTGTTGTCTCGATGATTAATATCGATACCAATATGACAATCGATGAGAAAGATCCAGCACCATCAACCCCTCAGTTAAGGCAAGTTTCACGGTTACTAGACCAAGAGAACAGAAATTGCTCGGTGGAAAATGTAAACTCCCCTACCGAGAAAGAGAACACGGAGGATCATGACATCGGTGAATCCAAATCAGTCGACACTACAAATCATGAAGACAATGTGATTGTCAAAGAAGAGAATGGAGATGGGGAAAAGGAAGAAGAACAAGGCCAAACCGAAGAACATAAACAAGAAGAAGGTGAAACCGGAGATTCAGTTAATTTTAATAAGAAAGAAGATGAGACTAATAAACCTATTAATAAATCATCCGGGAACAAGAACTTGTCTAGTAACAAAGCTAGTCGAAAGAAGGTGGATTGGACACAAGAGCTGCACAAGAAGTTTGTGCAAGCGGTAGAGCAACTTGGAGTCGATCAAGCGATACCTTCAAGGATTCTTGAATTGATGAAAGTAGATGGCTTAACTCGACATAATGTAGCTAGTCACCTTCAGAAATTTCGGATGCATAGGAGAAATATTCTTCCAAAGGATGATCATAACCATAGATGGATACAATCTAGAGAGAACCATAGACAAATCCAACGGCAATATAACGGTTTCCAGCAACAGCACCGTCCCGTAATGGCTTACCCCGTTTGGGGTCTTCCCGGTGTTCATCCGCCTGGAGCAGTTCCGCCTTTGTGGCCGCCGGCATTGCCGTCTGCCGGTCAACTACCTCCATGGCATTTTAAACCACCTTATCCAACGTTGAATGGTAATACATGGGGTTACCCGGTTGGACCACCGGTAACCGGAACATTCTTTCCTCCTCCGATAACCGGAACATTCAGTACTCATCCGGCGAATCAGCTGGACGAGGAAATGATTGATCAAGTGGTTAAAGAAGTGATTAGCAAACCGGGGATGCCGCTACCGCTGGGACTGAAACCGCCATCACCCGAGAGCGTTTTAACTGAGCTTTCGCGTCAAGACATCTCGGCCGTTCCTTCTTCCTCTGCCTCTTGTCAAATCAACGGCTCTCCTCGTCTCCGCTGA

>BrPRR13(Bra037427)-DNA

ATGCTTCAGAAATGGAAACCTAGTGGTGACGAAAGTGGTGGTCCAGATAGAGATTTACCAAACTCTCGCGATATGTTTTCTGGCAACTTTCCTGAGGGTTTGCGGGTGCTATTGTTTGATGAAGATCGAATATATCTTCAAATATTGGAGAAACATCTCGAAGAGTTTCAATACGAAGGTAGCTATTTTGATTGAGTTGTTTTTGGAGTTTTAAAACAGATCAAGTGATGATAAAATTCATATATAATTTCCAATGTGTTTTTTATTAATTTCAGTATTTCTAAAGTTGACCTAATTGATATTATAATCTTATAGATCTGTGTATTTTTGCAGTGACTACATGCCACGAAGAGGAAAGAGCTACGTATCTACTCTGCAACCATAGAAATATGTTCGACATTGCTATAATCGAAGCACACAATCTAGAGGGGAAAATATTCCGGTTAATCTCGGAAATTCGATCTGAAATAGATATTCCAATCATTAGTAAGCATATAATTTTAAAGTTTGTGTACATTGATTTTTTTGATTAAAAAACATAACAATTAAACACACTTTTTTTCCAGTAACATCCCAAGACGATTCAAGAGAATCTGTGACCAAATGGATGAGGAACGGTGCATGTGACTATCTCATAAAACCGATAAGACCAGAAGATCTACGTTTGATTTATAAATACTTGGTAAAGAAGATGGAACTAAGGGGTATCACGGTTGCCGAAGAGGCAGAAGAGAAAGCAGCAGCTGAGAAGTCATCCTCTGTGGGAGATTCCACCATAAGAAGCCCTAACAGGAGAAAAAGAAACATGTTACAAACAGACGAGGATCCAGATCATAATCGTGATTCCGCCACCAAGAAAAGGCGAGTGGTGTGGGATGATTATCTTAACGGCAAGTTTTTGGATGCAGTGAATTCCCTTGGCAACAATGGTGAGTCACGTACATTAGGGTTCATAGTTACTCGGGTTTGTCATCTTAGATTTATTCTATATATGCATATATATATATATATATACAAAATGTCTTGTTCGCACGTACAAACATAATTTTCTTAGGAATACTTATTAGTTTATATATTCTAATTAAAAAACCAGTATGATAATATACGATAGAAAAATTTCATCAAAATATATGTTTATTATTATGCTAAAATTTTTAAAACGCTCACATGTTAAAATATTTTTTAAATATATCTATTTGAACTATGTGAATTCATGCAGATGTTGTTCCCAAGAAAATTTTGGAGAGGATGAATGTTAGTAGCCTCACCCGAGAAAACGTAGCCAGCCATCTCCAGGTATCGTTTCTTCTTATACTACCACCACGGCGGTGCTTTGTTAGATTTTCAGAAAGCCCAACTTTGTGAATATAGGATTACCTCAAAAAATTGCACCTTGGATATTAAGATATGGATTCAGACTTGAAATATAAAAGATATCCTAGTGGATGCAAAACTTTTATAATAGAGGCTTTTACCTTTTGCATAAGTTTTCGTGAACCTCACCTACAAATCTTTAATTAGATTAAAAATATTGTAAGCAGAAACTATAAATAGCATTGATATATTTCGTACTCTAACCATTTTATAGCATCATATGTGACCGTAGACTTTAATCTGCATATTGTAACGTATATTGTAACGTCCCCGACAGTTTATGGTTACTGGGCCATTACGCCTTCTCATTCGGCTTGTGGACTCCATCCCGTCTAATGGACAGTGCATTAAATTTTTGAAGGCTCAAAATTATTGTTTATTGATCATGCAATTACCACGTAATATTTTTTCGTGTTTTGTGACAATAAACTATAAATAACATCGATATATTATGTACTCTAACCATTTTATAGTATCATATGTCATTGTAGACTTTATCTGCATATTGTAACGTTCCGACTGTCCACAGTTACTGGGCCACCATGCCCTCTCTCTCGGCATGTGGACTCCTCTATCCCGTCTGATGGACGGTGCATTAATTTTTCGGAGGCTCGAAATTATTGTTTATTGATTCTGCAATCACCACATAACATTTTTCTGTGCTTTGGTCTCACTCGCACGATATCGCAAATCACTTTTTGATAGGTCACTCACCCTTTCAGTATTCTAGCTCAAGCACACTTAACTCTGGAGTTCTAAATGGATGTGTGTCGATTAAGGTAAGCGACACTTTGGTAACATAGGTTGTCAAATCAATTCTCTTATGTCTTTCCACATATACCAGAAATTATGATGTTACAATTCATCCTCACTCACAAAGCGCAACACCACTCACAAAGCGCAACACCCCCGTTGTGCATCATGACATGTCTCAAGACGCCTCCCGTCAGAACCGAGATAGCTAAAATGGCTTTGATACCACTCATGTCTCCTCACTTGGCACGTGGACCCCATCCCTTCTGACGGGCGATGCGTTAATTTTTCGGAGTCTCAAAATCATTGTTTATTGACCCTGCAATCACCACATAAAATTTTTTCGTGCTTTGGCTTCACTTGCACGATATCGCAAATCATTTTTTGATATGTCACCAATCTTTTCACTATTCCAGCTCAAGCACGGTTAACTTTAGAGTTCTAAACAGATGTGTACCGGAAAAGGTAAGCACACTTTGGTGACATGGATAGCAAAATCAATTCTTTTAAACTTTTCCACAAATACCAGAAATCAAGATGTTACACATATGTTCATTAAGAATCTGAAGAATACATTTAAATTAAACAAATGAGAACAATTATGTAAAATATAAAAAATATTAACTCTGGAAAAAAAGTCAGCGAATCCACCAGGTTTTTGGTTATTTCCAGCAAATCATAAAGTTTGTCTCGAATCGAATTAAGGAGATTTGTTTGTCATGTAGACTTGACATTCCTCATATCAAACTTTCCATCTCAGTACGAAGAGACCATAAGTTTGTGCGGATCCTAGAAGTACAAAATATTCCACTCCTTTGATCCATAATATTCCACTCCTTTGATCCATATGGCTCATCCGTACGCCACTAACCTAGTCATGATCAATTCATTAAGAATCAACTTAGCAAGATGGGTTCAAGTACTAAACTGAAACAACTAATTGGTCGAAAAGAACTTCTGTTTTACCCTCATCCTCTTTTAGATTTGTGACCCTATAGCTTTCCACTTCAAGAGAAGCTAATTAAACTGTGTACACAAAAAAAATAGATTTTTTCTTGTTGAAGACTTTTTCGGTTTTTGCTTTATAAATGTACCAAATGATCCAATGAATGTTTTAATTTGTGGTTCTATAGTAAATTTTTTCTCCAAAATAGAAAAGCTATATTTTTATAAATATATGTACTTGGAAAAATCCAAAATGGTCATATATTATAGGTCTAGACATTTTACCCGGATCCAAAGACTCGGACCAAAATCAACAAAAAAAGTGGTTTGGATCGAAGGAGATTTTACCCTACTGGGTTTGTTTATAGATAACCACGGGTCTTGGTTCGGATTTATGTCCACCCCGAAACCCAATCTGA

>BrPRR13(Bra037427)-CDS

ATGCTTCAGAAATGGAAACCTAGTGGTGACGAAAGTGGTGGTCCAGATAGAGATTTACCAAACTCTCGCGATATGTTTTCTGGCAACTTTCCTGAGGGTTTGCGGGTGCTATTGTTTGATGAAGATCGAATATATCTTCAAATATTGGAGAAACATCTCGAAGAGTTTCAATACGAAGTGACTACATGCCACGAAGAGGAAAGAGCTACGTATCTACTCTGCAACCATAGAAATATGTTCGACATTGCTATAATCGAAGCACACAATCTAGAGGGGAAAATATTCCGGTTAATCTCGGAAATTCGATCTGAAATAGATATTCCAATCATTATAACATCCCAAGACGATTCAAGAGAATCTGTGACCAAATGGATGAGGAACGGTGCATGTGACTATCTCATAAAACCGATAAGACCAGAAGATCTACGTTTGATTTATAAATACTTGGTAAAGAAGATGGAACTAAGGGGTATCACGGTTGCCGAAGAGGCAGAAGAGAAAGCAGCAGCTGAGAAGTCATCCTCTGTGGGAGATTCCACCATAAGAAGCCCTAACAGGAGAAAAAGAAACATGTTACAAACAGACGAGGATCCAGATCATAATCGTGATTCCGCCACCAAGAAAAGGCGAGTGGTGTGGGATGATTATCTTAACGGCAAGTTTTTGGATGCAGTGAATTCCCTTGGCAACAATGATGTTGTTCCCAAGAAAATTTTGGAGAGGATGAATGTTAGTAGCCTCACCCGAGAAAACGTAGCCAGCCATCTCCAGATAACCACGGGTCTTGGTTCGGATTTATGTCCACCCCGAAACCCAATCTGA

>BrPRR14(Bra020652)-DNA

ATGCTTACCGGCAAGTTTCCTGAAGGTTTGCGGGTGTTGGTCTATGATGAAGATTTCCAAAATCTTATTTCATTGGAGAAACATCTCCAATATTTTCAATACAAAGGTAGCTATCTGTGATATTCTTGTTTTTTAGAAGTTTTCGAAACTGATCAATTGATTCGTATTGTGCTAAATTCAGTGACTATATGCAACGAAGGGGCAGATGCCATGCATATGCTGCGCAACCATATGAACACGTTCGATATTGCTATCATAGAAGCACAAAATTCGGCTGTGGATATATTCAGATTGATTTCTGAAATTGCATCGGAAATTGATCTTCCAATCATTAGTAAGCATATGAATCAATAGTTTTAAAATGTTCATTAAATAAAATGCGACAACAAATTTTATTAAAAAACACTTATTGCAGTAACATCTAAAGACGATTCAGTACAATCCGTGATCAACTGGATGAAGATTGGTGTTTGCGACTATCTTATTAAACCGATAAGACCTGAAGATCTACGTTTTATTTTCAAACACGTGGTAAAGAAGATGCAAGTAGGGAAGAGGGTAGAGTCAGAAGAGAAAGCAACCGCCGAGAAGTCATCCTCTGTGGGAGATTCCACGATAAGAAACCCTAACAAGAGGAAAAGAAGTATGTTCATTGATGGACAAGTGGGGGAAAAGGATCAAGATCATGTTCGTGATTCCACCACCAAGAAAAGGCGAGTAGTGTGGGACAATGAGCTTAAAAAAAAGTTTCTGGATGCAATGGAAGACCTTGGCCCAGGTAGTGAGTCACATACATTAAGTTTAATCATTTTAGACTTATTCTATCCATATATATATATATGAACAGTGTGATTTTATGCAGAAGCTGTTCCCAAGAAAATTTTAGAGAGGATGAATGTTGTTGGCATGACTCGAGAAAATGTAGCCAGCCATCTCCAGGTAACCTTTCTTCTACTACTAACCATGGCCTCACTTAATTAGATTTTTAGAAACCCAAACAAAAATAAAATAAACATGATTTGCCTATAGAATATTTTAGATCAAATAAAATTGCACCTCATATTTGGATTCAGATTTGCCAAACATGAAATTTCAGGATATATCCTCATGCATGTTCACCTTTGATAGTAGGGGATGTACATAAGGTTTCGTGACTCGTGAGCCTTTAACTATAACTAGATCAAAGTCATCGTAAACATGTACGTGTACGACTATAGATAGCATAAATATTTATTGTATACTTTTACCAACTCATAACACTTTTTAGGACCGTAGACAGGTAGACTTTAGGATGTACCATTTTCGTAGGATGTACCATTGTACTTCCAATGAAAAATAACGCATGCTCATGTTCTATAAAATTAAGAAAACATCAGGGGGAGGAACACTTCTGACCAAATTAATAATAATATCCTAACATGACATTCATATCCTAAGGACATGACATGACCGAGTTAATCGAACAAGACAACTTGAAGCTGAATCACGGCATTTCAAAGCTAGCAAATAGCCAATTCTTGTGCCAAATATGGTCAATTCCATTGGGGGGCATTATATGGATTTAGACGAGTTTCTTTCTACATATATCTTTTGTGGTTCTATTTCTTACTTTTGTTTTGTTTCAAAGAACATCTACAAACTTTGATTTGTAAAATAAAACTTTTGTTGAAAGCTTTCCTGTGTAATTCTTTCAACTATTAATGCCTTGTCTATACTACTAATAACGTAGCTGTAAAAGTACATATAAGAACCAGGCTTGAAATGAGTGTTATTTCTGCTGTGAAATGTTTTAGAGGCTTTCTTTTGTTTCCATTTTTCTTTTCAAGTTTTTAAACTTACTTTTATTGTGGTCATTTTTATTTTACAGTATAACCTATTATTGGTTTAATTATTTTAGTTAAATAATGTATAGTGTTTCAAACGTAGAAAATATTAACTTGTTTATTATTTGTGTATGTGTTTTTAGCTAAACCACTATAAGAAAAAGATGAATATTTGTAACATTGTTGTAATTTGGAACTATATATATATAGAATAATTCATCAAAGATACACCACCAAATAATTAAATCAATATGAAATATGGATGTTCTTAAAACTTTCCGTATCAAAGGGAGTTCATATAGCTAGTGAATGTAGTTAGATGATTATTCTATAAATAAACTTAATAAAACAATTGGGAAATGATCTGCATGGTTGACTTTAAATGTATATTATTAATCTTTTTTAAATTGGATGTGTAGAAACACCGGATGCTTCTAAATAGGCAGAAATCTCACAATGAGAAAGATGAGAAGAAACGGTCTTTATTATCGCCACAAGGAGGACTGCATAGTGGAGAAGGAGGCAGCAATATTCAATTCTCAACCCAACATATCAGCAACATTCCTCATCAACCTTTCAGACATCATCCTGATGGAGTCCCTGTTGTCGTTTCCACTAGGAACCTACTGATGACTAACCAACATCACCTTCAAACCTCAGATTTCACATCTATTGAAAACGTAGAAGAGAGTTTGATTTTTACTGAAGAAGACGCTGAGGTTTCAAACTTGGCTTTCTTGTTCACACAAAAATCCGAAGAGATGAGTTTGTCCCACTTACATGAGCCGGTGATGGCAACTACAATGCTTTCCAATGATAATCAGTTGTTTCCCAATCAACAACAGATGATGAACTTTCATGAACCATCCATTTTGCATACACATTCGTTTCCTTTATCTCTCACACCTAGTTCCTTTCTTGATCAGGTACTATGCTTGTGCACTTCTTATGATTTTCTTTTGTTATGTGGATCCATATCTTTAATCTATTTTGTGTGTGTTTCGTACAGAAAGAAACCATAATGATGATGAATGTAGATGAAGGACTACAACAATGGTTACTCAATGAACAAGAACAGCCTAACCTAACTGATGAGAATAGGTTTTCGAGCATTAATCCAAGGTAGGGATCTGCAAGAAGGGATATATGGTTAAGGATCAGGGCCAAGCATGAAACCCGAATTACAAGTAAGAAGATGAAGTCCGATCATATAGTTTAATGTTGTTCTTATTATTTTCTAGGGCTTAG

>BrPRR14(Bra020652)-CDS

ATGCTTACCGGCAAGTTTCCTGAAGGTTTGCGGGTGTTGGTCTATGATGAAGATTTCCAAAATCTTATTTCATTGGAGAAACATCTCCAATATTTTCAATACAAAGTGACTATATGCAACGAAGGGGCAGATGCCATGCATATGCTGCGCAACCATATGAACACGTTCGATATTGCTATCATAGAAGCACAAAATTCGGCTGTGGATATATTCAGATTGATTTCTGAAATTGCATCGGAAATTGATCTTCCAATCATTATAACATCTAAAGACGATTCAGTACAATCCGTGATCAACTGGATGAAGATTGGTGTTTGCGACTATCTTATTAAACCGATAAGACCTGAAGATCTACGTTTTATTTTCAAACACGTGGTAAAGAAGATGCAAGTAGGGAAGAGGGTAGAGTCAGAAGAGAAAGCAACCGCCGAGAAGTCATCCTCTGTGGGAGATTCCACGATAAGAAACCCTAACAAGAGGAAAAGAAGTATGTTCATTGATGGACAAGTGGGGGAAAAGGATCAAGATCATGTTCGTGATTCCACCACCAAGAAAAGGCGAGTAGTGTGGGACAATGAGCTTAAAAAAAAGTTTCTGGATGCAATGGAAGACCTTGGCCCAGAAGCTGTTCCCAAGAAAATTTTAGAGAGGATGAATGTTGTTGGCATGACTCGAGAAAATGTAGCCAGCCATCTCCAGAAACACCGGATGCTTCTAAATAGGCAGAAATCTCACAATGAGAAAGATGAGAAGAAACGGTCTTTATTATCGCCACAAGGAGGACTGCATAGTGGAGAAGGAGGCAGCAATATTCAATTCTCAACCCAACATATCAGCAACATTCCTCATCAACCTTTCAGACATCATCCTGATGGAGTCCCTGTTGTCGTTTCCACTAGGAACCTACTGATGACTAACCAACATCACCTTCAAACCTCAGATTTCACATCTATTGAAAACGTAGAAGAGAGTTTGATTTTTACTGAAGAAGACGCTGAGGTTTCAAACTTGGCTTTCTTGTTCACACAAAAATCCGAAGAGATGAGTTTGTCCCACTTACATGAGCCGGTGATGGCAACTACAATGCTTTCCAATGATAATCAGTTGTTTCCCAATCAACAACAGATGATGAACTTTCATGAACCATCCATTTTGCATACACATTCGTTTCCTTTATCTCTCACACCTAGTTCCTTTCTTGATCAGAAAGAAACCATAATGATGATGAATGTAGATGAAGGACTACAACAATGGTTACTCAATGAACAAGAACAGCCTAACCTAACTGATGAGAATAGGTTTTCGAGCATTAATCCAAGGGCTTAG

>BrPRR15(Bra004306)-DNA

ATGGCCAAAGAAATTGCTTTTGATGTTACCGGAAACAAAATTTCTAATTCCAATGGTGATACTTTCATTCTGCTAATCGATCATGACATTGCATCGATCTCTTCTCTCACTTCAATGCTTCAACAACTTTCTCACAAAGGTAAAGATCTTGTTACTCGTTATAAATTTTCTTTTATTTCGCTGTTTATATAATAGTTATCATTTTTCTATTCACATATTTATTATATCCATTTATAAATGATTTCATCTATTATGTTAACATTATGATTCAAAATACATAGATTTGTGGTTATTAATATTTGAACTAATTTGATAAATCTATTTAAGCTATAATTAATTATTTTATTAAAAATGTATAAAAAAATATAACATTTTATCTCAATTTTAGTTATTCATTTGAATTTTTATGGTATTCCATTATAGAAAATATATGAAACTAAATTTATCTAAATATAATATGTTAGATTAGCATGAAATATAATCAACAATACAAATCTTAACTATTATTTTTATTTCTGAAGATAACTAAAGGTCTAATAAGAAAAAAATTGCTTGTTTTCTTCAACATTTGGTTTTGAACTTTTCAAAAAACTAATATATAAATTGATGTATTAGTGATAAGTGTCAATGTGGCAAGTGAGGCTGTATCGATGCTCAAAAAACAGATGGATATCGTACTTGTCATCGCCAACACTGAAATGCCTCATATAGATTCACATTCCTTCTACACTTCTTTGCTGACCAGAGACATTCCTTTAATATGTACGCTTATTTAATGCATCATCTTACTATTTTGTTCATTTGTTTAATGTATATGGTAACTTGTTTTTTTTTTATCTTGTACATGTTAAAAAGTGATCAGTCCAGAGGGAAAGAAAGCTAAACCATCAAACTCTTTGGAAAAAAGAGCGTGTTATCTCCTAGAGAAGCCTATTTCTGAAAAAGATATCAACAACATGTTGCAGCATGTCTTATCTAATAAGAGCCAAAAGTTAACAAAAATCAGCATACCTAAAAGTGGAGGGGGCAATATGGAGAAACGTATAAATCAGATGAAAGCTTTCAGAGAAATCCTTAGGAGACAGCGTCCATCATCTTTTCTAGGAAAACCATTGTTAAAAAAATCAGCGTACCAAGAAAGAAGAAATATAGCAAATGTTGAAAGGAAAAACAAAACTGTGTATCCTGTTGAGTTTGAAAATAAGAGAAATGAAGGAAATAATATAGATAGTAACACTGGAATACGCAATAATTTCTGGACTTATGAGCATCAGATGAAGTTCTTTTCTGCTGGCGCTAACTTGGGTGAAAAAGGTACGATTTTTATTTCATTTTGACAATTTCTTCTTTGTTTCAATCATATTCTTTTACTCTTCTATTCTGCAGATTCTCATCCAAAATCCTTATTGGGGATTATGAATGATCGAACATCGAAGAATGAATATCCTTTCACATTATCCAACATAGCAAAGAATTTCTTTGCAGGTAATAACTCCGTGGGAATTCTCTTAAAAGGGAGAACTCTTTATCATCACTCATCATTCATCACTCAATGCAAATATGCGTATTTCATAAAATTTCAGATAAGAACCGGATTAAGGAGAGGGATACAATGGCGCTAAAGTTTTATCAAGGAACAAAAATGGACTTAAGCCGTACCTCTTGGTTTGGTAATATACCAAACAGTTCATCCATGGAAGCAGATCGTGTACCTGCGGCCACAAGTAACATCCCACCTTGCAACATTTCTCCCACTGATACTGTTAGCCATACCAATCTGGTTTCAACTAGCTTAAACGATAACAATTTTCTTGACCATTCTGGCCTGCCATCAAGCGTTGGTACATCTAACTCAGGTGCGTTTGATAAGTTATATGCTTCAATATAATTCTAGCACGTTTCAGTTTCTAAACTAGTATAAATTAGATGTTCTTCCTAAATTTAATTTGAACTTACTCTTCCGCAAGACTAATCCAGATCAGTTGGGTTGTGTTTCTTTAGGAGAAATGTGTGCTACTTCTGAAGATATGATTCTATCTACAGTTCAAAATGATGTTATCCATTGCGAACCAAGTCATACTTCTCTGAACAGTGAGGTCACTCTTCAAAGCAATATGACACTTCCTCAAACCAATATAATTGATATAGGTAATACTTCTTTTAGAGAATGCTATGTTATTCCTAAAGATATGATATCATGTGAAACTGATATGACTCTTGAGAGTTTGGTCTCTGGTGGATTAAATTGTGGAACTTATTAACTAGTATGACCATTCTTGGAACCAATATAAACATATGAACCAGATTTCTTCTTAAAAGGTTATGTTATTCCTAAATATATGATTCCTCTAAGACTAATACAAATTTATTTCTTTGTGAAATAAATTATGCTGCTCTAGGTAATGCGACTCCTCTTGAAACCAACATAGAAGATATAAGCCAGTTTCAACAAGATGCATGTTATGATTTGCCGATTGAAGATTTGATTTCTTTTGACACTGACGTTCATGAGATGGACATGCAGCATGTGTTAGGAAATAATGGTTCTTCTGAGGTGAATATTTTGCCCAACACTCATGTCGGCCATGAGGATTGACGACAAGTTTTAAATGATAAGTATATGTTTATTAGTGATAGAGAATAAATGAATAAACCAAGACATTAAATGATATTGGACTTGTTTTATATATTGCAAGTGCATTTTACATAAATAAAAAAACAATATATTGCATGAACATTTTATATTCGTTTTGTATGTCAAAAATACATATCATACTAGTGCATGTGCATTATTTACATATATATAATAACAATGTAGATTTCATAATTTCTTTAGACTCTCAGTAATCTTCAGGTAAATGCGTATTCAAACACTTAGTTATAATTATATATCTCATTAACTTTGGTTTGTTCATATTATATGTTTTCTTGGTTGTAATTTAGAACGGATGAAGGCTATCTACTGTCAAAGGGTTCGAATGCATCAAATTCATTTCAGTATCAAGTTCTTTGTGTTGTGCCACATATATTTAACCAAAAATGCAAATCATAGCCTCCCACAAAACTCTAACTAGCATTGTAAATTCATTTCTGTATCAAGTTTCTTGTCTATATTATTTTGCAGTACCTTGAAGAAGTTGGATGGCCGAAGCAGGCTGTGTTATGCTTGCACACAGCCAAAACGCCTGGTTGTCAAGGTTGATTTTCTCTCCTGCACCACATCATGCAGCTAAGTCTGAATATTAGAGTCTCGAATATGGATATTCGTTAGAATTTTTGGTTAATCCCACACCTAATTTAACAATTTGATTACTGTTGCTCCAAGAGAGTTGCTGACGAGATGAGAGTCAAGCTTGAGGACGAATTTGGTTATACCATTTGATTCATAGGCTATACAACTTCGGTAGGGATTCTTATTTTAAGCTTAAACTAATATCAACAGGAAGCTATGTTCAGGAAGGTTTCTGCAATCATCAAATTGGATACAAAAATTTATATTAAGACAGCAGCGGAACTTCTCATGCAACGTAAAATATTTCGTCTCTGAGAAGATCTCTGTGTATATGTGTTATCAGTTCATGAAGCTAACTGCAAGTCTCGTTTACAGGTGGGATTTTAAAGCTCTTGAAGATGCACTATGTAAACGTATTATAATCACACCCGAGGAAGTTGACAAGTGAAGTCTTGCTAAAGACCGATTGTTCGATTGGTAATGGATCTTTATATATTTGATGCTAACTGAATATCCCTAGTTATGTGTAGATTGATAGACAAGATTATAAAAGCCAATCGGGCAAGATGCTACTGCTATCATATTTGGAACAAAATCAATCCCAAACATAAATGAATACCAAAACAAAGTTAAACTTCAAAAAATATCTACAAGTAAACTACACATATCAAAAGCATATATGAACCTAGTAAGACAAGACGGTCTGTCAATTCTCAATGCCTTTTGAGAAGATTAGTGACTAGGAAAAGAGCAGATAAATCTCTATTACACATTGGGTCTAACTACTGTTTTTTCATTAAAAAAACAACGGGCTTATACATCAATAACAAAACCATCTCTGGTTTATTGATTGAGACGTGTTTCCAAAATCAATCTCTTTTATTTACCTTAGAGTTGCACCGATCATCAATACACAAACTTGTCTCACTCAAATCGAATTGATTCAACAAAACAGACAATCAAAACACACACTTCCTAAAAGAGCCAAATTACCAAATGTTGGAAAGAAACCCTAACCATTTTCCACGGTAAAGGGCTGCGACTTCCTGAACTCACGCTCCGGGAAGGATATGGAGAAGAAGAGCGCGAAAGAGACCTAGACGGACTCGAAACTGAGCGGGAAGAAGATCTGGAACTGGACCGAGATGACGACCTAGAGACAGAAGGAGAACGACAGCCTCGGCTTTGA

>BrPRR15(Bra004306)-CDS

ATGGCCAAAGAAATTGCTTTTGATGTTACCGGAAACAAAATTTCTAATTCCAATGGTGATACTTTCATTCTGCTAATCGATCATGACATTGCATCGATCTCTTCTCTCACTTCAATGCTTCAACAACTTTCTCACAAAGTGATAAGTGTCAATGTGGCAAGTGAGGCTGTATCGATGCTCAAAAAACAGATGGATATCGTACTTGTCATCGCCAACACTGAAATGCCTCATATAGATTCACATTCCTTCTACACTTCTTTGCTGACCAGAGACATTCCTTTAATATTGATCAGTCCAGAGGGAAAGAAAGCTAAACCATCAAACTCTTTGGAAAAAAGAGCGTGTTATCTCCTAGAGAAGCCTATTTCTGAAAAAGATATCAACAACATGTTGCAGCATGTCTTATCTAATAAGAGCCAAAAGTTAACAAAAATCAGCATACCTAAAAGTGGAGGGGGCAATATGGAGAAACGTATAAATCAGATGAAAGCTTTCAGAGAAATCCTTAGGAGACAGCGTCCATCATCTTTTCTAGGAAAACCATTGTTAAAAAAATCAGCGTACCAAGAAAGAAGAAATATAGCAAATGTTGAAAGGAAAAACAAAACTGTGTATCCTGTTGAGTTTGAAAATAAGAGAAATGAAGGAAATAATATAGATAGTAACACTGGAATACGCAATAATTTCTGGACTTATGAGCATCAGATGAAGTTCTTTTCTGCTGGCGCTAACTTGGGTGAAAAAGATTCTCATCCAAAATCCTTATTGGGGATTATGAATGATCGAACATCGAAGAATGAATATCCTTTCACATTATCCAACATAGCAAAGAATTTCTTTGCAGATAAGAACCGGATTAAGGAGAGGGATACAATGGCGCTAAAGTTTTATCAAGGAACAAAAATGGACTTAAGCCGTACCTCTTGGTTTGGTAATATACCAAACAGTTCATCCATGGAAGCAGATCGTGTACCTGCGGCCACAAGTAACATCCCACCTTGCAACATTTCTCCCACTGATACTGTTAGCCATACCAATCTGGTTTCAACTAGCTTAAACGATAACAATTTTCTTGACCATTCTGGCCTGCCATCAAGCGTTGGTACATCTAACTCAGGAGAAATGTGTGCTACTTCTGAAGATATGATTCTATCTACAGTTCAAAATGATGTTATCCATTGCGAACCAAGTCATACTTCTCTGAACAGTGAGGTCACTCTTCAAAGCAATATGACACTTCCTCAAACCAATATAATTGATATAGGTAATGCGACTCCTCTTGAAACCAACATAGAAGATATAAGCCAGTTTCAACAAGATGCATGTTATGATTTGCCGATTGAAGATTTGATTTCTTTTGACACTGACGTTCATGAGATGGACATGCAGCATGTGTTAGGAAATAATGGTTCTTCTGAGAGCCAAATTACCAAATGTTGGAAAGAAACCCTAACCATTTTCCACGGTAAAGGGCTGCGACTTCCTGAACTCACGCTCCGGGAAGGATATGGAGAAGAAGAGCGCGAAAGAGACCTAGACGGACTCGAAACTGAGCGGGAAGAAGATCTGGAACTGGACCGAGATGACGACCTAGAGACAGAAGGAGAACGACAGCCTCGGCTTTGA

**Protein sequences**

>BrHK1(Bra032761)

MVNVRKFVTSRPIFVFFLLAFLVIVFGCIPVTIWLRTTKNVTDGIVFCTEGLRSSLVSDIENIGKFTYQKTSSSTTGLANIIDSYLTNNDTHFKKIQTQVAPVLFKAYLTIPQVSQVSYISTDGLLFSYKTELNASVAVFANSSSGKGDYTWYTQTVDQITGRLTGNAKKSKPVDVTHKDWFQAVQRNHTTAFVGPGLGGEVNEAMFQSVVSLYSKKGAVSLGFPVKTLIDSLNRLHLKGGELYLWTKEGTLIVPGRSLNATFFISNGSICFGRESSHCIPGNCSSRGYQVEIGRLKFQAFCSVLEVSGVPLRYTLMFPNKERTPSIRSASLYLLVATMLLGLCWPLGFVACMVNAAGREMHMRATLIKQMEATQQAERKSMNKSQAFARASHDIRGSLAGITGLIDLCHDSEEVRHGSNLESRLKLVNGCTKDLLDLLNSVLDTSKIESGKMQLKEEEFNLAKLVEDVIDFFHPVAMKKGVDVVLDMHDGSVFKCSNVRGDGGKLKQILNNLVSNAVKFTVEGHISIRAWAQRTSSNVILAPENKRGLSKFSKKSKDQAGSPSNSVRNNGNMMEFVFEVDDTGKGIPKEMRKSVFENYVQVRETDQGQQGTGLGLGIVQSLVRLMGGEIRIIDKAMGEKGTCFQFNVLLSAASESQVSRQDTEEGEHMHGLIKTSSGGSMSIRNMSPRLHNWLSSSPKQERSRVVLLMKDGERRRVTEKYIKSLGIKVTVVKKWEHLNHVLERLGVSRQGSMGRNESLSSSSRELPLIGMDGIDSRSQTPKRTRHGFSPALLVVIDAETGHFLELYDIVEQFRRGMHHGLSCKVVWLNDRGHGSLRGEISCSKPLHGSCLNRVLKMLPEFGATEPKEDKQGAFKPSEDELLSGKRVLLVDDDRITSSIATIKLKKMGASEVKQCYNGKEAVRLVSEWLTQREHGEGGSSEVLLPFDYIFMDCQMPEMNGYEATREIRKMEEKYGGGLHIPIIAVSGHELGSTEARETIQAGMDAFLEKNLNHDQLAKVIREITSKGGSTLLSDSLLAG﹡

>BrHK2(Bra006075)

MVCEMETDQTEEMDIEVLSSMWPEDVGTQADNNQFNVEKPAGDSDTLKEVDIAEKRTMADLKRLPELMNTTDQGTSQLTNLVKQWEYMQDHAVKLLREELKILTKQREEAEAKELKIIEEHNFESQEPENVPVLDDTSHLFRRYKHKKRDALIGSKRVEIDEEFDTVAYWKQKALSLEKMLEASTERERRLIEKLNESLKTMESHSAPVEELTQNLKRAEGFLHFILQNAPIVMGHQDKDLRYLFIYNKFPTLREQDILGKTDVEIFHGGGVKESEYFKREVLEKGKASKREITFETELFGSKTFLIYVEPVYNKAREKIGINYMGMEVTDQVRKREKMAKLREDNAVRKAMESELNKTIHITEETMRAKQMLATMSHEIRSPLSGVVGMAEILSTTKLDKEQRQLLTVMMSSGDLVLQLINDILDLSKVESGVMRLEATKFRPREVVKHVLQTAAASLKKDLTLEGNITDEVPIEVVGDVLRIRQILTNLISNAIKFTHEGKVGIKLKVISEPSFASGMELNADAEEQNGLTETETSVWIRCDVYDTGIGIPGKFKNKTQAKLLDLLLLPEHVQPNRKVRFSVKKALPCLFKKYMQASADHARKYGGTGLGLAICKQLVELMGGQLTVTSQVNAGSTFTFILPYKVATSDDHSDDQDFSDMVDHHQPEPDDTTEGYFQFKPLLGSIYSNGGPVIGNNNFLPHKVMLTSPLKLINGFVADPSNNTGQSETTQVENNGYMDETCSGPCPSKETESCSSSQASSEGGPLEMESELTVSSRREDETTETSKQPKILLVEDNKINIMVAKSMMKQLGYTFDIANNGVEAINAIKDSSYDLVLMDVCMPVLDGLKATRLIRSYEESGNWDAAIEAGVDIKISENEQACVHSTNRLPIIAMTANTLAESSEECYANGMDSFISKPVTLQKLKECLRQYLH﹡

>BrHK3(Bra009011)

MVCEMETDQIEEMDVEVLSSMWPDDVGTQADNQFNVEKPAGDSDTLKEVDIAEKRTMADLKRLPELLNTTDQGSSQLTNLVRQWEYMQDHAVRLLREELKILTRQREEAEAKELKIIEEHNFETEEPENVPVLDETSHLFRRFRQKKRDELVDSKRVVIDEEFDTVAYWKQKALSLEKMLEASTERERRLIEKLNESLKTMESHSAPVEELTQNLKRAEGFLHFILQNAPIVMGHQDKDLRYLFIYNKFPSLREQDILGKTDVEIFHGGGVKESEDFKREVLEKGKASKREITFETELFGSKTFLIYVEPVYNKAREKIGINYMGMEVTDQVRKREKMAKLREDNAVRKAMESELTKTIHITEETMRAKQMLATMSHEIRSPLSGVVGMAEILSTTKLDKEQRQLLNVMISSGDLVLQLINDILDLSKVESGVMKLEATKFRPREVVKHVLQTAAASLKKDLTLEGNIADEVPILVVGDVLRIRQILTNLISNAIKFTHRGKVGIKLKVISQPSFASDKEQNETSVWICCDVYDTGIGIPENALPCLFKKYMQASADHARKYGGTGLGLAICKQLVELMGGQLTVTSQVNLGSTFTFVLPYKVATPDDHSDDQDEFSDMVDHHQPEPDDTTEGYFQFKPLLGSIYSNGGPVMGNNFLPHKAMLTSPIKLINGSVADPSNSSGQSQTVQVENGGYMDESESAHQYGNGNGHRCSSKESESCSSSQASSEMESELTVSSPREEEKTETEVKETSQPKILLVEDNKINIMVAKSMMKQLGYTMDIANNGVEAINAVKDTSYDLVLMDVCMPVMDGLKATRLIRSYEESGNWDAAIEAGVDIKTSESEQGCERSTDRLPIVAMTANTLAESSEECYANGMDSFISKPVTLQKLKECLQQYLQ﹡

>BrHK4(Bra028573)

MVCEMETDHQTEEMDVEVLSSMWPEDVGGTEPDNQFNVEKPAGDSDTLKEVEFAEKRTMADLKRLPDLLNTTDQGSSQLTNLVKQWEYMQDHAVRLLREELKILTKQREEAEAKELKIIEEHNFETEEPENVPVLDESSDLFRRFKEKKRDKLVGRKRIEIDEEFDTVAYWKQKALSLEKMLEASTERERRLIEKLNESLKTMESHSAPVEELTQNLKRAEGFLHFILQNAPIVMGHQDKDLRYLFIYNKFPTLREHDILGKTDVEIFHGGGVKESEDFKREVLEKGKASKREITFETDLFGSKTFLIYVEPVYNKAREKIGINYMGMEVTDQVRKREKMAKLREDNAVRKAMESELTKTIHITEETMRAKQMLATMSHEIRSPLSGVVGMAEILSTTKLDKEQRQLLNVMISSGDLVLQFINDILDLSKVESGVMKLEATKFRPREVVKHVLQTAAASLKKELTLEGNIADEVPILVVGDVLRIRQILTNLISNAIKFTHQGKVGIKLKVIPEPSFASGLELNADAEEQNGLTETETSVWIRCDVYDTGIGIPENALPCLFKKYMQASADHARKYGGTGLGLAICKQLVELMGGQLTVTSQVDLGSTFTFILPYKVATSNDHSDDQDEFSDMVDHQPEPDDSTEGYFQFKPLLGSIYSNGGPVIGNNFLPHKVMLPSPVKLINGQSEAVQVENGGYMDGPRHETRSGHCPESPHQYENGNGPCPSKESESCSSSQASSEMESEFTVSSPREEKKTETEVKKTSKQPKILLVEDNKINIMVAKSMMKQLGYTFDIANNGVEAITAINGSSYDLVLMDVCMPVLDGLKATRLIRSYEQSGNWDAAVEAGVDIKTLEDKQLCVRSTNRLPIIAMTANTLSESSEECYANGMDSFISKPVTLQKLKECLKQYLH﹡

>BrHK5(Bra002095)

MQRDSFSVSIESLPDSPMGPRKKKITKLFDNMTEWVTPWRSNPESPRETRIIRGDVEQEQFQYASSHCLSSYYSVFVVRLAIMVMLAILIGLLTILTWHFTRIYTKQSLQNLATGLRYELLQRPILRMWSVLNTTSELTTAQVKLSEYVIKKYDKPTTQEELVEMYQAMKDVTWALFASAKALNAITINYRNGFVQAFHRDPASSSTFYIYSDLKNYSISETTGLEDVKMSMGHGWNNQTIHGNMTAIWYQQQLDPITGAKLGKPLQIPPDDLINIAGISQVPDGEASWHVTVSKYMDSPLLSAALPVFDASNKSIVAVVGVTTSLYSVGQLMRELVEVHGGHIYLTSQEGYLLATSTDGPLLTNTSRGPKLMKAVDSKEWAIKTGAHWLEKTYGSHLPDVVHADNARLGDQQYYLDSFYLNLKRLPIIGVVIIPRKFIMGKVDERAFKTLVILISASVCIFFIGCVCILILTNGVSKEMKLRAELIRQLDARRRAEASSNYKSQFLANMSHELRTPMAAVIGLLDILISDDCLSNEQYATVTQIRKCSTALLRLLNNILDLSKVESGKLVLEEAEFDLGRELEGLVDMFSVQCINHNVETVLDLSDDMPTLVRGDSARLVQIFANLISNSIKFTTTGHIILRGWCDSVSSLHDEMSLTVDRKKPWAPVKTKLVHHRNHLHKSCKNENKIVLWFEVDDTGCGIDPSKWDSVFESFEQADPSTTRTHGGTGLGLCIVRNLVNKMGGDIKVVQKNGRGTIMRLHLILSIPDNAEQIYQPEFSQYGLVVLLSMSGSTARSIQSKWLRKHGIATVEASDWNVLTQIIRDLFETGSRENSFDSQHTIAESLRAELSNTQEIRNPVFVIVVDIGVLDLTTDIWKEQLNYLDRFSSKAKFAWLLKHDTSNTVKTELRRKGHVMMVNKPLYKAKMIQILEAVIKNRKRGLRGNGSDESHDCLEIDPTQFDTCSSDDSSENTSLKPTALPSPVIKNYLLDITKSNDESTSMTQKKKEEEEEDWKDRSNRLYSGVALDGKNQKSLEGVRILLAEDTPVLQRVATIMLEKMGATVTAVWDGQQAVDALNYKSINAQEHNNLSEEEETNPQSDTPNSSPYDLILMDCQMPKMDGYEATKAIRRAEIGTSLHVPIVALTAHAMSSDEAKCLEVGMDAYLTKPIDRKLMVSTILSLTKPSTVLTSFSD﹡

>BrHK6(Bra035381)

MSITRELTSKKAEESKGGVKWIKKPLFFMIMCCLTTSLVIVLLMSSGKEEETGSCNGEARVLYRHQNVTRSEIHDLVSLFSDSDQVTSFECRKESIPGMWANYGITCSLNMRLEKEETQCGHDNSKSLIPWNLINPSGLQQSLLHPENHREGLDWDLPSYLRNTWWCLIFGLLVCHKIFVSHSKPPNEIEGKTNLQDSLAQKQQQRACRGAGKWRKNILLLGVIAGVSMSVWWFWDSNQKIILQRRETLENMCDERARVLQDQFNVSLNHVHALSILVSTFHHGKTPSAIDQKTFGEYTERTNFERPLTSGVAYALKVPHSKREQFEKDHGWTIKKMETEDQTLVQDCVPENFDPAPIQDEYAPVIFAQETVSHIVSVDMMSGQEDRENILRARASGKGVLTSPFKLLKSNHLGVVLTFAVYDTDLPADATEEQRVEATIGYLGASYDMPSLVEKLLHQLASKQTIVVDVYDTSNASGLIKMYGTEIGDTSEEYISSLDFGDPSRKHEMRCRFKDKLPIPWTAIIPSGLVLIITFLVGYIFHDAISRIAIVEEDCQKMMKLKARAEAADIAKSQFLATVSHEIRTPMNGVLGMLKMLMDTDLDAKQMDYAQTAHGSGKDLISLINEVLDQAKIESGRLELENVPFDMRLVLDNVSSLLSGKANEKGIELAVYVSSQVPEVVVGDPSRFRQIITNLVGNSIKFTREKGHIFISVHLAEEVRESVNIKDAVLRQRLAVGSSASGETVSGFPAVNAWGSWESFKTIYSTENHTCDRIIKLLVTVEDTGVGIPLDAQGRIFTPFMQADSSTSRTYGGTGIGLSISKRLVELMQGEMGFVSRPGIGSTFSFTGVFGKAETTSSFNTKFDLAIQEFKGLRALVIDSRNIRAEVTRYHLQRLGICADIVSSLRTACTCVNKLGGLAMVLIDKDAWNKKDFELLDGLLNCSKETTTRPTKILLLATSATLVERSEMKSSGLVDEVVIKPLRMSVLICSLQETLVNGKKRQPSRKRTNLGHLLREKRILVVDDNLVNRRVAEGALKKYGAIVTCVESGKAALAMLKPPHNFDACFMDLQMPEMDGFEATRRVRDLEKKKIVSGEMYSSWHVPILAMTADVIQATNEECVKCGMDGYVSKPFEEEALYAAVAKFFESDG﹡

>BrHK7(Bra013186)

MSIACELSNPNLKKTKAEKRIPTKILLIRVLCGLVVLWLCLSLSLGFLCICKKKEAAAAADDSSSSAKGMLFRNQSRSEIDAMLSLFFDSNQVTSFECRKENGGITCSLSTRSEKGDEEEEEAKRHVVAELMSSSENEEEGVGGGRMIFARKEVSSLVQDKQQQQQQCKTAGKWRKNMLLLGIIAGVSLSVLWFWDTNEKILFQRKETLTNMCEERARVLQDQFNVSMNHVHALSILVSTFHHGKTPSAIDQKTFGEYTERTNFERPLTSGVAYALKVTHSEREKFEKEHGWSIKKMDFEDQTLVQGFDPAPVQDEYAPVIFAQETVSHIVSVDMMSGKEDRENILRARALGKGVLTSPFQLLKSNHLGVILTFAVYNTNLPHDATEEERIQATIGYLGASYDMPSLVEKLLQQLASKQTISVNVYDTTNASSVIKMYGSEVGDMSEEHISSLDFGDPFRKHEMHCRFTQKPPIPWLAIMPPGFALVITLLLGYIFNEAINRIATVEEDYQKMMELKARAEAADVAKSQFLATVSHEIRTPMVGVLGMLKLLMDTDLDAKQLDFAETAHGSGKDLISLINEVLDQAKIESGRLELENVPFDLRFLLDNVSSLLSGKAAEKGIELAVYVSSNVPAVIVGDPGRFRQIITNLVGNSIKFTQEKGHIFISVHLTNEVREPFETEDEILKQRLGSDETSCNTLSGYPAVNASGSWRNFKTFQDHSCDKTELLVTVEDTGIGIPVDAQSRIFTPFMQADSSTSRTYGGTGIGLSISKRLVELMQGEIGFVSKLGVGTTFSFTGVFGERERDSSVTALELFDQSIQEFQGLKALVIDSRNIRAEVTRYHLQRVGVSSVDIASSLATASSSCASKLVNLDLILIDKDAWNKEEYVAFNSGQEPSTRHPKIILLATFTTPAELSEIKSTGLVDEVVMKPLRMSVLICCLQETVGSGGKKRQTNRKPKNLGNLLRGKHILVVDDTMVNRRVADAVLRKYGALVTCVDSGKAAVAMLKPPHDFDACFMDLQMPEMDGFEATRRVRGLEEEMNKKEESRKWHTPILAMTADVIQATNEKCIKCGMDGFVSKPFEEEELYSSVARFFEK﹡

>BrHK8(Bra030037)

MSLFHVLGFCLKIGQLFWMLCCWFLSWFLDADKSPLDPDKTKMKNHNKMCFLWNKISTSGLKIPPSFSHHLFGSVRFGKTFWRKVLVAWVVSWVLISFWTFWCLTSQAMDKRKETLASMCDERARMLQDQFNVSMNHVQAMSILISTFHHSKFPSAIDQRTFSEYTDRTSFERPLTSGVAYAVRVLHSERQEFERQQGWTIRRMEQNPVHKDDYDTEALEPSPVQQEYAPVIFAQDTVSHVISLDMLSGKEDRENVLRARRSGKGVLTAPFPLIKTNRLGVILTFAVYKRDLPSNATPNERIEATNGYLGGVFDIETLVENLLQQLASKQTILVNVYDTTNHSQPISMYGSHVSADGLEHVSPLNFGDPFRKHEMRCRFKQKPPWPVQSMVTSFGILVIALLVAHIFHATLSRIRRAEEDCHKMELLKKKAEAADVAKSQFLATVSHEIRTPMNGVLGMLHMLMDTELDVTQQDYVRTAQASGKALVSLINEVLDQAKIESGKVELEEVRFDLRGILDDVLSLFSGKSQEKGLELAVYISDRVPEMLIGDPGRFRQILTNLMGNSIKFTEKGHIFVTVHLVEELLDSSDVETSSSSSTENTLSGLPVADRKRSWQNFKAFSSNGHRSLALAPSEINLVVSVEDTGVGIPVEAQSRIFTPFMQVGPSISRTHGGTGIGLSISKCLVGLMKGEIGFSSTPKVGSTFTFTVVFANGVHSTERKSELHNNNQPEFEGMNAVLVDHRPARAQVSWYHFQRLGIRVEVVTSVDQALRFMKTYATTVNMILIEQEVWNKEADVFVKDPLVHSPKLFLLANSIDTSVSDTLSNVIDPPVLIVKPLRASMLAATLQRGLGIGKRETPQRKGPPALILRNLLLGRKILIVDDNNVNLRVAAGALKKYGADVVCAESGVKAVSLLKPPHEFDACFMDIQMPEMDGFEATRRIRGMEEEMNNGEALTVEEGKRSRWHLPVLAMTADVIQATHEKCLKCGMDGYVSKPFEAEQLYREVSRFFNSPSDTES﹡

>BrHK9(Bra024849)

MNWALNNPNPEEKEPTTTTQSSDFYHLGAKDSPQKPRKIDFWRSGLMGFAKMQHSVAVKMNNGNNNDQVGNKKGSTFIQEHRALLPKGLILWTIIVGFISRGIYQWMDDTSKVRREEVLVSMCDQRARMLQDQFSVSVNHVHALAILVSTFHYHKNPSAIDQGTFADYTARTAFERPLLSGVAYAEKVVNAEREMFESQHNWVIKTMDTGEPSPVRDEYAPVIFSQDSVSYLESLDMMSGEEDRENILRARETGKAVLTSPFRLLASHHLGVVLTFPVYKASLPKNPTVQERIAATAGYLGGAFDVESLVENLLGQLAGNQAIVVHVYDITNASDPLVMYGNQDEEGDTSLYHESKLDFGDPFRKHKMICRYLQKAPIPLNVLTTVPLFFAIGFLVGYILYGAAVHIVKVEDDFHEMQELKVRAEAADVAKSQFLATVSHEIRTPMNGILGMLAMLLDTELSSTQRDYAQTAQVCGKALIALINEVLDRAKIEAGKLELESVPFDIRSILDDVLSLFSEESRNKGIELAVFVSDKVPEIVKGDSGRFRQIIINLVGNSVKFTEKGHIFVKVHLAEQSKDGAESKPALNGGVASEDITAASKPSSYNTLSGYEAADGRNSWDSFKHLLSSEELLTSSEFEASSNDRLMVSIEDTGIGIPLTAQGRVFMPFMQADSSTSRTYGGTGIGLSISKCLVELMRGQISFVSRPRVGSTFWFTAVFERCDKCSLKKPTVENLPSSFRGMRAIVVDAKPVRAAVTRYHMKRLGISVDVMTSLRTAVSTASGRNGSPLPSGTTKLDMILVEKDSWISTEDIDAEIRQMNSRTNGNVHHKTPKLALFATNITNSEFDRAKSAGFADTVIMKPLRASMIGACLQQVLELRKARQQHPEGSSPATLKSLLTGKKILVVDDNMVNRRVAAGALKKFGAEVVCAESGQFALGLLQIPHSFDACFMDIQMPQMDGFEATRQIRMMEKEAKEKTKLEWHLPILAMTADVIHATYEECLKSGMDGYVSKPFEEENLYKSVAKSFKANPISDSSCSQS﹡

>BrHK10(Bra004160)

MMEVCNCIEPQWPADELLMKYQYISDFFIAVAYFSIPLELIYFVKKSAVFPYRWVLVQFGAFIVLCGATHLINLWTFTTHSRTVALVMTTAKVLTAVVSCATALMLVHIIPDLLSVKTRELFLKNKAAELDREMGLIRTQEETGRHVRMLTHEIRSTLDRHTILKTTLVELGRTLALEECALWMPTRTGLELQLSYTLRQQHPVEYTVPIQLPVINQVFGTSRAVKISPNSPVARLRPVSGKYLLGEVVAVRVPLLHLSNFQINDWPELSTKRYALMVLMLPSDSARQWHVHELELVEVVADQVAVALSHAAILEESMRARDLLMEQNVALDIARREAETAIRARNDFLAVMNHEMRTPMHAIIALSSLLQETELTPEQRLMVETVLKSSSLLATLMNDVLDLSRLEDGSLQLELGTFNLHTLFREVLNLIKPIAVVKKLPITLNLAPDLPEFVVGDEKRLMQIILNIVGNAVKFSKQGSISVTALVTKSDNRAPPDFFVVPTGSHFYLRVKVKDLGAGINPQDIPKLFTKFAQTQSLATRSSGGSGLGLAISKRFVNLMEGNIWIESEGVGKGCTAIFDVKLGISNESKQSGIPKVPANPQHVNFAGLKVLVMDENGVSRMVTKGLLVHLGCEVATVSSSEECLRVVSHEHRVVFMDVCTPGVENYQIALRIHEKFTKRHQRPLLVALTGNTDKSTKERCMSFGLDGVLLKPVSLDNMRNVLSDLLEHRVLYEAM﹡

>BrHK11(Bra004449)

MEPCDCFETNANQDDLLVKYQYISDALIALAYFSIPLELIYFVNKSAFFPYKWVLMQFGAFIILCGATHFINLWMFFNHSKVVAIVMTLAKVSCAAVSCATALMLVHIIPDLLSVKNRELFLKKKADELDREMGLILTQEETGRHVRMLTHEIRSTLDRHTILRTTLVELGKTLCLEECALWMPSQSGLYLQLSHTLSHKIQVGSSVPINLPIINQLFNSAQAMHIPHTCPLAKIGPPVGRYAPPEVVSVRVPLLHLSNFQGSDWSDLSGKGYAIMVLILPTDGARKWRDHELELVEVVADQVAVALSHAAILEESMHARDQLMEQNFALDKARQEAEMAVHARNDFLAVMNHEMRTPMHAIISLSSLLLETELSPEQRVMIETILKSSNLVATLISDVLDLSRLEDGSLLLENEPFSLQAIFEEVISLIKPIASVKKLSTNLILSADLPAYAIGDEKRLMQTMLNIMGNAVKFTKEGHVSIIASIMKPESLRELPSPDFYPVPSDNHFYLCVQATVCGIHTHDIPFLFTKFVQPRTGAQRNHAGAGLGLALCKRFVGLMGGCIWIESEGIEKGCTASFIIRLGICNGPGSSSGSMALRLAAKSQTRPWNW﹡

>BrHKL1(Bra023756)

MMAREVASGLLILFSILICVSPAAAGNGGGCNCEDEGVSFWSTENILETQRVSDFLIAVAYFSIPIELLYFVSCSNVPFKWVLFEFIAFIVLCGMTHLLHGWTYGPHPFKLMVALTVFKMLTALVSCATAITLITLIPLLLKVKVREFMLKKKAHELGREVGLIMIQKETGVHVRMLTQEIRKSLDRHTILYTTLVELSKTLALQNCAVWMENEGKSEMNLTHELRGSSGRSGYGYSVSMHDVDVVRVRESNDVNILSVDSLIARASGGDVSEIGPVAAIRMPMLRVSDFKGGTPELIQTCYAILVCVLPSGQPRDWSYQEIEIVKVVADQVAVALSHAAVLEESQMMRDKLADQNRALQIAKRDAMRASQARNVLQKAMSEGMRRPMHSILGLLSMIQDEKLSNEQKMIVDTMVKTGNVMSNLVGDAMDVSDGRFVTEMKPFSLHRTVREAACLARCLCLYNGFRFTVDAEKSLPDNVVGDERRVFQVILHMVGSLVKPRKCREGSLSSSVIFKVFKERGSLDRSDQRWAAWRSPTCSADGDVYIRFEMSVENDGSGSQSFASVSSRDQEVGEVRLSGYGLGQDLSFGVCKKVVELIKGNISVVPGSDGSPETMSLLLRFRRRPSISVHGIGEAPALDHHLHPHSDSLLRGLQVLLVDTNDSNRAVTRKLLEKLGCIVTAVSSGYDCLTAIAPSSSSSSPTFQVVVLDLQMAEMDGYEVAMRIRSRSWPLIVAMTVSLDEEMWDKCMQIGINGVVRKPVMLRAMESELRRVLLQADQLL﹡

>BrHKL2(Bra040134)

MLRSLGLGLLLFALIALVTGDNNDYGSCNCDDEGYYFFTVHTILECQRVSDLLIAIAYFSIPLELLYFISFSNVPFKWVLVQFIAFIVLCGMTHLLNAWTYYGPHSFQLMLWLTIFKFLTALVSCATAITLLTLIPLLLKWKVRELYLKQNVLELNEEVGLMKRQKEMSVHVRMLTREIRKSLDKHMILRTTLVELSKILDLQNSAVWMPNENRTEMHLTHELRSNSMRSFRVVPINDPDVVQVREAKVVALLRKDSLLAVESSGSDESGPVAAIRMPMLHGSNFKGGTPEFVDTSYAIMVLVLPNANSRVWTDREIEIAEVVADQVAVALSHASVLEESQLMREKLGIQNRALLRAKQNAMMASQARNTCQKVMSHGMRRPMHTILGLLSMFQSESMSLDQKIIVDALMKTSTVLSALINDVIDISPKDNGKSPLEVKRFQLHSLIREAACVAKCLSVYKGYGFEMDVQTRLPSLVVGDEKRTFQLVMYMLGYILDMSEGGKTVTFRVVSEGTGSSQDKNKRESGMWKSHLSDDSLGVKFEVEINEIKSPPLDGSVIAMRHVTNRRYHSNGIKEGLSLGMCRKLAQMMQGNIWISPKSHGQTQSMQLVLRFQTRPSIRRSILAGNAPELQHPNSNSILRGLRITLADDDDVNRTVTKRLLEKLGCEVTAVSSGFECLSALSNVEMSYRVVILDLQMPEMDGFEVAMKIRKFCGHHWPLIIALTASTEDHVRERCLQMGMNGMIQKPVLLHVMASELRRALQSASE﹡

>BrHKL3(Bra015303)

MLKTLLLHVLLFFFFLTASVAGSLSICNCDDEDSFFTFEAILQSQKAGDFLIAVAYFSIPIELLYFVSRTNVPSPYNWVVCEFIAFIVLCGMTHLLAGFTYGPHWAWVTTAVTVFKMLTGIVSFLTAISLVTLLPLLLKAKVREFMLSKKTRELGREVGIIMKQTETSLHVRMLTSKIRTSLDRHTILYTTLVELSKTLGLKNCAVWIPNEIKTEMNLTHELRPGHHDGGGGGGYGGGFSIPITESDVVRIKRSEEVNMLNSGSALASVTSRGKPDGQTVGIRVPMLRVCNFKGGTPEAIHMCYAILVCVLPSRSWTYQELEIVKVVADQVAVAISHAVILEESQLMREKLAEQNRALQVARENALRANQAKAAFEEMMGDAMRRPVRSILGLLPVIARDGGLQENQKVIVDAMGRTSELLLHLVSNAGDITRPGETHCFSLRSVAKETACLARCFCVGNGFGFSTEVDRSLPDYVVGDARKVFQVVLHMLGGLVNRNIKGNVTFLVSPESSEVDSQEAVWRQCYSKEYIKVKFGFEVETSSVPSFNISEDIVKLMQGNIRVVEDGSGLVKSLSVAFRFQLRGSMLSQGGGYSGETFKTATPPSTSNDHWRQEEIR﹡

>BrHKL4(Bra030564)

MLKTLLVHGPLLFFFFFLISSVVAGDENDGGLSICNCDDEDSYFSYEGILESQKVGDFLIAVAYFSIPIELLYFVSRTNVSSPYIWVVCEFIAFIVLCGMSHLLSGFTYGPHYPWVMTAATVFKMLTAIVSFLTAISLVTLLPLLLKAKVREFMLSKKTRELNREVGLIMKQTETSLHVRMLTTKIRTSLDRHTILYTTLVELSKTLGLKNCAVWIPNEIKTEMNLTHELNGENVGRGPGGGPSGGPGGFSIPITESDVVRIKRSVEVNMLSAGSALASVTTRGKSGQTVGIRVPMLRVCNFKGGTPEAIHMCYAILVCVLPLRRSWSYQELEIVKVVADQVAVAISHAVILEESQLMREKLAEQNRALQVARENAMRANQAKAAFEEMMGDAMRRPVRSILELLPLITQDGVSLPETQKVIVDAMGRTSELLLHLVNNAGDVASGTHCFSLRSVVKETACLARCLCLGNGFGFTTDVDRALPDCVVGDARKVLQVVLHMLGGVMNRKVKGNVTFKVVPERGSSEVVKESQEAAWRQCYSKEYVEVKFGFDVAAEGEESSSSSSSTKFMQGNVLVVEDGLGLVKSLSVVFRFQLRRSIVSRGGGYSGETFKTSTPPSTSNGHWRHLKEALKKNLYWGMTETVNEDSGVGRSVEASSNGHHSLSGESLSLSKWRSSAQVENGTPSTSLSYWDTDDDEDHGLKPSQLFGKHKWKIEKFSEIKKRELRSNYFEAGGYKWYILIYPQGCDVCNHLSLFLCVANHDKLLPGWSHFAQFTIAVVNKDPKKSKFSDTLHRFWKKEHDWGWKKFMESTKLQDGFIDDSDSLTIEAQVQVIRERVDRPFRCLHCGYRRELVRVYLSNVEQSCRRFVEEKRSKLGRLIEDKAKWTSFGVFWLGMDQNSRHRMSREKMDVILKGIVKHFFIEKEVTSTLVMDSLYSGLKALEGQSKSKKARPRSLDAKECPAPIVSVDKDMFVLVDDVLLLLERAALEPLPPKENKAPQNRTKKVTCIEINVFVLQCKSRMAMMEKSSKIEVAYKEAIALKRQEDLIREEEEEWLAETEQRAKRGAAEREKKSKKKQAKQKRNKNKGKDKKKEEKVTLATHGKDLEENHHDEEENDSVTEKAQPSAEKTDTLEEVSDISDSVDGSADILHPDLEDGDSSSVHWDADALEIHPPPSEGSSISISTPNGIAERKTQSTMDDSSSTCSNDSIRSGVTNGSYKGNMLNFRNQKSPNQGKNQQVKITSDTRSLVTEPDDDQPKSQNSSSESDWVVVSHIQELESSRNRRPVEKQRNVAQVVVNSVHMDRPEKKSAAVLSSPRTAAKNPSSLTQTKLEKRSVSNADAVPNKKVMSATGPPSSSQVSPASSDSQSQAGGLKADMQKISAPKQPATTTIVTRPFSAPIIPAMRPAPVIVSSSVQPTTSLPRSVSSAGRLGPDSSLRNQQSYTPQSYKHAIVGNSPGSSSSFNHHPSSHGVVPTTLPSASYTQTPAYQSSSFPFGQDGSFRSRSFNSVNMGMNNRYTPAVASNTSLNHIDIETARQQAQSLMTDEFPHLDIINDLLEDENCSNTVFNGSIFNSQSQLFNSQYSYHGGGSADLGISGELLSSGRSRSFGDEGFHYMARGPYAEGLIPTQWQMANMDLSLLAMRNSNVEDTASYHHTYNFGLDSTNQSFSSGINGYTEFRPSNGH﹡

>BrHKL5(Bra020013)

MACREALPVGRESSEFLTTLEDQHLSAEDGLGTWEPPEDQHSNVTIICQYCRAFLLAVTIYKKKHPINRFLLALFSERQIGSDPGENMSGSRPSHSSEGSRRSRHSARIIAQTTVDAKLHADFEESGSCFDYSTSVRVTGPVVENQPPRSDKVTTTYLHHIQKGKLIQPFGCLLALDEKTFKVIAYSENAAELLTMASHAVPSVGEQGALGIGTDIRSLFTAPSASALQKALGFGDVSLLNPILVHCKTSAKPFYAIVHRVTGSIIVDFEPVKPYEVPMTAAGALQSYKLAAKAITRLQSLPSGSMERLCDTMVQEVFELTGYDRVMAYKFHDDDHGEVVSEVTKPGLEPYLGLHYPATDIPQAARFLFMKNKVRMIVDCNAKHVTVLQDEKLSSDLTLCGSTLRAPHSCHLQYMANMDSIASLVMAVVVNEEDGEGDAAAPDSTAPQKRKRLWGLVVCHNTTPRFVPFPLRYACEFLAQVFAIHVNKEVELENQIVEKNILRTQTLLCDMLMRDAPLGIVSQSPNIMDLVKCDGAALLYKDKVWKLGITPSEFHLQEIASWLCEYHTDSTGLSTDSLHDAGFPRALALGDSVCGMAAVRISSKDMIFWFRSHTAGEVRWGGAKHDPDDRDDARRMHPRSSFKAFLEVVKTRSLPWKDYEMDAIHSLQLILRNAFKDGEATDVNTKIIHSKLNDLKIDGIQELEAVTSEMVRLIETATVPILAVDSDGLVNGWNTKISELTGLPVDEAIGKHLLTLVEDSSVEIVKRMLENALEGTEEQNVQFEIKTHLSRADAGPISLVVNACASRDLHENVVGVCFVAHDLTGQKTVMDKFTRIEGDYKAIIQNPNPLIPPIFGTDEFGWCTEWNPAMSKLTGLKREEVMDKMLLGEVFGTQKSCCRLKNQEAFVNLGIVLNNAVTSQEAEKVPFAFFTRGGKYVECLLCVSKKLDREGVVTGVFCFLQLASHELQQALHVQRLAERTALKRLKALAYIKRQIRNPLSGIMFTRKMMEVSELGPEQRRILQTSALCQKQLSKILDDSDLESIIEGCLDLEMKEFSLNEVLTASTSQVMMKSNGKSVRITNDTGEEVMSDTLYGDSIRLQQVLADFMLMSVNFTPSGGELTVTASLRKDQLGRSVHLAYLEIRITHTGAGLPEFLLNQMFGTEEDMSEEGLSLMVSRKLVKLMNGDVQYLRQAGKSSFIITAELAAANK﹡

>BrHKL6(Bra031672)

MSGSRPSQSSEGSSRRSRHSARIIAQTTVDAKLHADFEESGGSSFDYSTSVRVTTPAVENNQPPRSDKVTTTYLHHIQKGKLIQPFGCLLALDEKTFKVIAYSENAPELLTMASHAVPSVGESPVLGVGTDIRSLFTAPSASSLQKALGFGDVSLLNPILVHCRTSAKPFYAIVHRVTGSIVVDFEPVKPYEVPMTAAGALQSYKLAAKAITRLQSLPSGSMERLCDTMVQEVFELTGYDRVMAYKFHEDDHGEVVSEVTKPVLEPYLGLHYPATDIPQAARFLFMKNKVRMIVDCNAKHVKVLQDEKLSFDLTLCGSTLRAPHTCHLQYMANMDSIASLVMAVVVNEEEDGEATTPQKRKRLWGLVVCHNTTPRFVPFPLRYACEFLAQVFAIHVNKEVELENQIVEKNILRTQTLLCDMLMRDAPLGIVSQSPNIMDLVKCDGAALLYKDKVWNLGTTPSEFHLQEIAFWLCEHHADSTGLSTDSLHDAGFPGALALGDSVCGMAAVRISSRDMIFWFRSHTAGEVRWGGAKHDPDDRDDARRMHPRSSFKAFLEVVKTRSLPWKDYEMDAIHSLQLILRNAFKDGESSDVNTNIIHSKLNDLKIDGIQELEAVTSEMVRLIETATVPILAVDSDGLVNGWNTKIAELTGLPVDEAIGKDFLTLVEDSSVEIVQRMLENALEGTEEQNVQFEIKTHLSRTDAGPISLVVNACASKDLHENVVGVCFVAHDLTAQKTVMDKFTRIEGDYKAIIQNPNPLIPPIFGTDESGWCTEWNPAMSNLTGLKREEVVEKMLLGEVFGTQKKSCCRLKNQEAFVNLGIVLNNAVTSEDAEKVPFGFFTRGGKYVECLLCVSKKLDREGAVTGVFCFLQLASYELQQALHVQRLAERTALKRLKALAYIKREIRNPLSGIMFTRKMMEGTEIGPEQRMILQTSGLCEKQLSKILDDSDLESIIEGCLDLEMKEFTLNEVLTASTSQVMMKSNGKSVRVTNETKEEVMSDTLYGDSIRLQQVLADIMLMSVNFTPSGGQLTVTASLRKDQLGRSVHLAYLEIRITHTGAGLPEFLLNQMFGSEEDVSEEGLSLMVSRKLVKLMNGDVQYLREAGKSSFIITAELAAASK﹡

>BrHKL7(Bra022192)

MVSGVGGGGGSGGSGRGGGRGGEESSSTHRREQAQSSGTKSLRPQSQPQTESISKAIQQYTVDARLHAVFEQSGESGRSFDYSQSLKTTTYGSSVPEQQITAYLSRIQRGGYIQPFGCMIAVDESTFAIIGYSENAREMLGLTPQSVPSLERPEILAMGTDVRSLFTSSSSVLLERAFVAREITLLNPVWIHSKYTGKPFYAILHRIDVGVVIDLEPARTEDPALSIAGAVQSQKLAVRAISQLQSLPGGDIKLLCDTVVESVRDLTGYDRVMVYKFHEDEHGEVVAESRREDLEPYIGLHYPATDIPQASRFLFKQNRVRMIVDCHATPVLVVQDDRLTQSMCLVGSTLRAPHGCHSQYMANMGSIASLAMAVIINGSEEDGSSVAGGRSAMRLWGLVVCHHTSSRCIPFPLRYACEFLMQAFGLQLNMELQLALQMSEKRVLRTQTLLCDMLLRDSPAGIVTQSPSIMDLVKCDGAAFLYHGNYYPLGVAPTEAQIKDVVEWLLANHADSTGLSTDSLGDAGYPGAAALGDAVCGMAVAYITKRDFLFWFRSHTAKEIKWGGAKHHPEDKDDGQRMHPRSSFKAFLEVVKSRSQPWETAEMDAIHSLQLILRDSFKESEAAMNSKTADGAVQPYSMAGEQGIDELGAVAREMVRLIETATVPIFAVDAGGCINGWNAKIAELTGLSVEEAMGKSLVSDLIYKENEETVDKLISRALRGTFSSPTMLYLLYGYLIILMRFAGDEDKNVEIKLKTFSPELQGKAVFVVVNACSSKDYSNNIVGVCFVGQDVTGQKIVMDKFINIQGDYKAIVHSPNPLIPPIFAADENTCCLEWNTALEKLTGWSRSEVIGKMLVGEVFGSCCRLKGPDALTKFMIVLHNAIGGQETDKFPFPFFDRNGKFVQALLTANKRVSFDGKVIGAFCFLQIPSPELQQALAVQRRQDTECFTKAKELAYICQVVKNPLSGLRFTNSLLEATDLNEDQKQLLETSVSCEKQISRIVSDMDLERIEDGSFELVRTEFLLGSVINAIVSQAMFLLKERGVQLIRDIPEEIKSIQVYGDQTRIQQLLAEFLLSIIRYAPSHEWVEIHISHVPKQMADGFYAIRTEFRMACPGEGLPPELVRDMFHSSRWTSPEGLGLSVCRKILKIMNGEVQYIRESERSYFLIILELPVPMKRPLSTASGSGDMMLMMP﹡

>BrHKL8(Bra039485)

MSSGSSNSGSCSTRSRNNSRLSSQVLADAKLHGSFEESERLFDYSASIHVNMPTSSSYDIPSSSDVSSYLHKIQRGMLIQPFGCLIVVDDKTLKVIAFSENTQEMLGLSPHTVPSMEQREALSIGTDVQSLFQSQGSSALQKAADFGEISILNPITLHCRTSGKPFYAILHRIEQGLVIDLEPVGLDEVPVTAAGALKSYKLAAKSISRLQALPSGNMSLLCDALVKEVSELTGYDRVMVYKFHGDGHGEVIAECCKADLEPYLGLHYSATDIPQASRFLFMRNKVRMICDCSAVPVKVVQDKSLSQPITLAGSTLRAPHGCHAQYMSNMGSVASLVMSVTINGSESDEMNRDLQTGRTLWGLVVCHHASPRVVPFPLRYACEFLTQVFGVHINKEAESALLLKEKHILQTQSVLCDMLFRNAPIGIVTQSPNIMDLVKCDGAALYYRDKLWALGVAPTETQIRDIIDWVLKSQGGGNSGVTTESLMESGYPDASVLGESICGMAAVHITQKVFLFWFRSGTAKQIKWGGARHDPDDRDGKRMHPRSSFKAFMEIVRWKSMPWDDMEMDAINSLQLIIKGSLQEEHPDTVVNVPPFVDNRVQKVDEMCVIVNEMVRLIDTAAVPIFAADASGVINGWNSKAAEVTGLAVEQAIGKPVSDIVEDDSAITVKNMLALALQGSEERGAEIRIRAFGPKRKSSPIELVVNTCCSRDTRNNVLGVCFIGQDVTGQKTLIEKYSRVQGDYARIMWSPSTLIPPIFMTTENGLCSEWNDAMQKLSGIRREEAVNKMLLGEVFTSNDSCCRLQDHDTLTKLRIALNAVSSGQDNIEKLLFGFYHRDGRFIEALLSANKRTDMEGKVTGVLCFLQVPSPELQYALQVQRISEQAMACAVNKMAYLRQQVENPEKAISFLQDFLHSSGLNEEQKQLLSTSVSCREQLAKVISDSDIEGIEDGYVQLGCSEFSLEESLESVVKQVMELSIERKVQIICDYPQEVSLMRLYGDSLRLQQILSETLSSSIRFTPALKGLCVSFKVMSRIEAIGKRMKRVELEFRIIHPAPGLPDDLVREMFQPLRKDTSREGLGLHITQKMVKLMEGGTLRYLRESEMSAFVILAEFPLL﹡

>BrHKL9(Bra013286)

MGFESSSSAASNMEQPQQKSNTAQQYSVDAGLFADFDHSVYSGKSFNYSKSMISPPNNVPDEHITAYLSTIQRGGLVQPFGCLIAVQEPSFRILGLSDNCIDFLGLSLASTSQPNHFTVKGLIGIDARSLFTPSSAASLVKAASFTEISLMNPVLVHSRTTTTASHKPFYAILHRIDAGIVIDLEPAKSSDPALTLAGAVQSQKLAVRAISRLQSLPGGDIGALCDTVVEDVQRLTGYDRVMVYQFHDDDHGEVVSEIRRSDLEPYLGLHYPATDIPQAARFLFKQNRVRMICDCNATPVKVVQSEELKRSLCLVNSTLRAPHSCHTQYMANMGSIASLVLAIVTKTKNSSKLWGLVVGHHCSPRYVPFPLRYACEFLMQAFGLQLQMELQLASQLAEKKAMKTQTLLCDMLLRDTVSAIVTQSPGIMDLVKCDGAALYYKGRCWLVGVTPSESQVKELVDWLVENHGDESTGLTTDSLVDAGYPGAVSLGDKVCGVAAAGISLKDYLIWFRSNTASAIKWGGAKHHPKDKDDAGRMHPRSSFKAFLEVAKSRSLPWEVSEIDAIHSLRVIMRESFTISRPVVLSSGGNNGVVGGRDASELTSFVCEMVRVIETATAPIFGVDSNGCVNGWNNKTAEMTGLGAGEAMGKSLVDEIVQEESRGALESVLSKALQGEEKKNVMLKLRKFGNEDSSSSDVCVLVNSCTSRDYTEKIVGVCFVGQDMTSEKAITDRFIRLQGDYKTIVQSLNPLIPPIFASDENARCSEWNAAMEKLTGWSKHEVIGRMLPGEVFGDLCKVKCQDALTKFLISLYQGIAGGGNVPESSVVGFFSKEGKYIEASLTANKSTNGEGKVIGCFFFLQIINKESSSSSPEVKESAQSLNELAYIRQEIKNPLNGIRFAHKLLESSEISENQRQFLETSDACEKQIATIIEDTDLKSIEEGKLQMETEEFRLESVLDTIISQVMIMLRERKSQLRVEVSQEIKTLPLYGDRVKLQLILADLLRNIVNHAPFPDSWVGIKISPGHKLAHDNNPYIHLQFRMIHPGKGLPSEMLSDMFETREGWVTPDGLGLKLSRKLLEQMNGRVSYVREDERCFFQVDLQVKTRLGVETGSSI﹡

>BrHP1(Bra023876)

MELVQMQKSLQDYTKSLFLDGVLDSQFLQLQQLQDESNPDFVSQVVTLFFQDSDRILNDLSLSLDQQVVDFKKVDPHVHQLKGSSSSIGAQRVKNACVVFRNFCEQQNVEGCHRCLQQVKKEYYLVKNRLETLFKLEQQIVASGGMIPAMELGF﹡

>BrHP2(Bra036215)

MDAHVAQLQMQYRNYILSLYQQGFLDDQFTELKSLQDDGSPDFVAEVLSLFFDDCVKLVGNMARALDQTGTVDFSQVGANVHQLKGSSSSVGAKRVKGLCINFKELCEAKNYEGCVRCLQQVDIEYKTLKAKLQDMFNLEKQIVQAGGIVPQVDIN﹡

>BrHP3(Bra025394)

MDALVAQLQMQYRNYTVSLYQQGFLDDQFTELKKLQDDASPDFVAEVLSLFFEDCVKLIGNMARALDQTGTVDFSQVGASVHQLKGSSSSVGAKRVKGLCVTFKEYCEANNYEGCVRCLQQVDIEYKALQTKLQDMFNLEKQIIQAGGKVPQVDIN﹡

>BrHP4(Bra028236)

MFTYHSLDLGPKNQTDVLECCRQSNDALDEDKYFVFLTFYRESSSGFLDDQFTELKKLQDECSPDFVAEVVSLFFEDCEKLIGNMARALDQTGNVDFSLVGSSVHQLKGSSSSVGAKRVKGLCVTFKECCDSQNFEGCVRCLQQVDIEYKSLKAKLQDLFSLEQQIVQAGGRIPQVDI﹡

>BrHP5(Bra027169)

MGKCMQGFLDEQFMELEELQDDVNPNFVEEVATLYFKDSARLINSIDQALERGSFDFNRLDNYMHQFKGSSSSIGASKVKTECTMFREYCRVGNAEGCLRTFQQVKKEHATLRKKLEHYFQASQ﹡

>BrHP6(Bra001629)

MQRQVALIKQSLFDQGYLDEQFIELEELQDDANPNFVEEVATLYFKDSARLISNIEQALERGSFDFNRLDNYMHQFKGSSTSIGASKVKTECTMFREYCRVGNAEGFLTIPYLRRVQMFEDFQATEERARNVEKEA﹡

>BrHP7(Bra033398)

MNTVVAKLQRQFQDYLVSLYQQGFLDNQFTELRKLQDEGTPDFVAEVVSLFFDDCSKLINSMSRSLERPENVDFKQVDSGVHQLKGSSSSVGARRVKNVCISFKECCDVQNREGCLRCLQQVDYEYKMLKTKLQDLFNLEKQIVQAGGAIPQVNIN﹡

>BrPHP1(Bra003551)

MLGLGVDRLQADINRLLTSLFHQGVLDEQFLQLQQLQDETSPTFVYDVINIYFDESEKLLRSLRLLLMDREFSDYKKIGLHLNQLVGSSSSIGARRVRNVCVAFRSASELNNRPGCLRGLEIVEHEYHYLKNMMHELFQLEQQRLLAAGVRYPM﹡

>BrRR1(Bra027829)

MPLDGGVSCRRRSEMIGIGIGELESPPLDSDQVHVLAVDDSLVDRIVIERLLRITSCKVTAVDSGWRALEFLGLDDDKSSVEFDRLKVDLIITDYCMPGMTGYELLKKIKESTSFREVPVVIMSSENVLTRIDRCLEEGAEDFLLKPVKLADVKRLRTYLTRDVKVSDGNKPKVPEDLSRFSSLAMVTPPPPSITSVESVSLSPESSVSPVDSPIRPMEMRSPGLD﹡

>BrRR2(Bra031714)

MARDGGVSCLRSSEMMRVGIGGMESPPLDLDEVHVLAVDDSLVDRIVIERLLRITSCKVTAVDSGWRALEFLGLDNEKASAELDRLKVDLIITDYCMPGMTGYELLKKIKESSSFRQVPVVIMSSENVLTRIDRCLEEGAEDFLLKPVKLADVKRLRSYLTGDVKLSNANKRKLPEDSVPVNTSFPPPPSPFPISPDSSDSSLPLTISPESSDSSPPLSPLEISSSPLSSPIDDEDDDVLTSSPAPEESPARRQKMRSPLD﹡

>BrRR3(Bra018439)

MAKDGGVSCLRSSEMLNVGIGGMEPPPLDLDEVHVLAVDDSLVDRIVIERLLRITSCKVTAVDSGWRALEFLGLDNDKASDEFDKLKVDMIITDYCMPGMTGYELLKKIKESSSFRQVPVVIMSSENVLTRIDRCLEEGAEDFLLKPVKLADVKRLRSCLTGDVKLSNGNKRKLPEDSVSVDTSLPPPPLSLTFSTNSSDSSPPLSPVEVFSSPLSSPEDDDDVLTSSPEGSPMSEESPIRRQKMGSPGLD﹡

>BrRR4(Bra019932)

MASDGGVSCLRRSEMMSVGIGGIDSPPLDVDEVHVLAVDDSLVDRIVIERLLRITSCKVTAVDSGWRALEFLGIDNEKASAELDRLKVDLIITDYCMPGMTGYELLKKIKESSSFRQVPVVIMSSENVITRIDRCLEEGAEDFLLKPVKLADVKRLRNYLTRDVQVSNGNKRKLPEDSPPLTLSHDSSDSSPPPSTLSPDSSDSSSPPLSPVEIFSSPLLSPLDDEDDDVLTTSPESTPSPVRRQKMRSPGLD﹡

>BrRR5(Bra033773)

MAEVLRPEMLDISNDTSSLASPELLHVLAVDDSIVDRKFIERLLRVSSCKVTVVDSATRALQYLGLDGDNSSVGFQINLIMTDYSMPGMTGYELLKKIKESSAFREIPVVIMSSENILPRIDRCLEEGAEDFLLKPVKLADVKRLRDSLMKAEERVFKNIMHKRELEANDIYSQLKRAKI﹡

>BrRR6(Bra019524)

MAEVMRPEKLDMSNDTSSLGSPELLHVLAADDSIVDRKFIERLLRVSSCKVTVVDSATRALQYLGLDGNHSFVGFLRCLEEGAEDFLLKPVKLADVKRLRDSLLKADEIAFKNIMHKRELQANDIYSQLKRAKI﹡

>BrRR7(Bra018084)

MRSETLNISNNTSSLASPELLHVLAVDDSIVDRKFIERLLRVSSCKVTVVDSATRALQYLGLDGDNNSVGFEDLKINLIMTDYSMPGMTGYELLKKIKESSAFREIPVVIMSSENILPRIDRCLEEGAEDFLLKPVKLADVKRLRDSLLKAEERVFKNIMHKRELEANDIFSQLKRAKI﹡

>BrRR8(Bra010132)

MAEVMLPMKMEMANDPSKFTSPDLLHVLAVDDSHVDRKFIERLLKVSSCKVTVVDSATRALQYLGLDVNEKPIGCKDLKVNLIMTDYSMPGMTGYELLKKIKESSAFRDVPVVVMSSENILPRIDRCLEEGAEDFLLKPVKLSDVRRIRDSLIKVEDLSFTKSINKRELETENVYSLDSSVPLQLKRTKI﹡

>BrRR9(Bra025708)

MAVGEVMRMEIPAGGDMSVTSPELHVLAVDDSIVDRKVIERLLRISACKVTTVESGARALQYLGLDGDKGASGLKDLKVNLIVTDYSMPGLTGYDLLKKIKESSVFREIPVVIMSSENILPRIEQCLREGAEDFLLKPVKLADVKRIKELIMRNEAEDCKTLSHSNKRKFAEYIDDASSPSPSSSSTHDESAAKDFPSSKRMKSEDDKFSSLL﹡

>BrRR10(Bra016526)

MAVGEVMRMEVPTGGDLTVSSPDLHVLAVDDSIVDRKVIERLLRISSCKVTTVESGTRALQYLGLDGNTGDSDLKDLKVNLIVTDYSMPGLTGYDLLKKIKESSTFREIPVVIMSSENILTRIEQCLKEGAEDFLLKPVKLADVKRIKQLIMRNEAEDHRTLSHSNKRKLGEDVDTSPSSSHDYSSVKDFPSSKRMKSESDIFSPFI﹡

>BrRR11(Bra000224)

MGVVTESQFHVLAVDDSLFDRKMIERLLQKSSCQVTTVDSGSKALELLGLRESNESDDPNATSTSPEVEINLIITDYCMPGMTGYDLLKRVKESAAFRSIPVVIMSSENVPARISRCLEEGAEEFFLKPVKLADLTKLKPHMMKTKLKKESEKPAEEVKPEIEEEESPVIEILTLHQELESEQQEPMLSNNKRKAMEEAISTGRSRPKYNDITTSV﹡

>BrRR12(Bra016943)

MGMATESQFHVLAVDDSLVDRKMIERLLQKSSCQVTTVDSGSKALEFLGLRESNESNDPNAPSSSPVTHQEIEINLIITDYCMPGMTGYDLLKRVKESAAFRSIPVVIMSSENVPARISRCLEEGAEEFFLKPVKLADLTKLKPHMMKTKLKKESENSAKEDNAVLKHEIRKEEEPSVIEVLPLHQEVEQEPMLSNNKRKAMEEVISTNRSRPKYNDITTLV﹡

>BrRR13(Bra004615)

MGMVRESQFHVLAVDDSQLDREMIERLLQKSSCQVTTVDSGSKALEFLGLRESNDPNSLETHQEVEINLIITDYCMPGMTGYDLLKKVKESAAFRSIPVVIMSSENVPARISRCLEEGAEEFFLKPVKLADVTKLKPHMMKTKLKKEGEKVAEEENATSKPEESLVVEMILPLNQELELEQQEPMLSSNKRKAMEEVISADRSRPKYNDITTSV﹡

>BrRR14(Bra014649)

MGMAAVESQFHVLAVDDSSFDRKLIEKLLQKSSCQVTTVDSGYKALEFLGIESNDPNALSTSPQEVEVNLIITDYCMPGMTGYDLLKKVKESSAFKNIPVVIMSSENVPARISRCLEEGAEEFFLKPVRMADLNKLKPHMMKTKLNNQKLEEIEKPLNVSAAAAAAVEPEIKDSAEVGSKILTLQSELEPKQVHLQVAQQEEQTLGNNNKRKSMEEGLSTDRSRPRFECVTTAV﹡

>BrRR15(Bra003265)

MVMAAEPQFHVLAVDDSLFDRKFIERLLQKASCQVTTVDSGYKALEFLGLREGFEINDPDAVSTSPVIHQEVEVNLIITDYCMPGMTGYDLLKKVKESSACKNIPVVIMSSENVPARISRCLEEGAEEFFLKPVRLADLNKLKPLMMKTKLKNQKLEEIEAPSKDESGTVAAVVEPEVKDLTEIGIKILPLQSEVEPKQVHLQVVQQEEQTMSNNKRKSVEEGLSTDRSRPRFEGITTAV﹡

>BrRR16(Bra007295)

MGMAAAEWKFHVLAVDDSLVDRKLIERLLQKSSCQVTTVDSGYKALEFLGLRQGIESNDTTALSLSPQEVNLIITDYCMPGMTGYDLLKKLKESSALKNIPVVIMSSENVPARISRCLEEGAEEFFLKPVRLADLNKLKPHMMKTKLKNQKLEEIITPSNDENGTVAAVEPEIKDSVEMEIIKMLPIQSETEPKRVLLQVVQQEEQMLSNNKRKSMEEGLSTDRPRPRLEGIATAV﹡

>BrRR17(Bra015885)

MALGDLSSSSSSTSELHVLAVDDSIVDRKVIERLLRISACKVTTVESGTRALQYLGLDGDKGSSGLKDLKVNLIVTDYSMPGLTGYELLKKIKESSAFREIPVVIMSSENIQPRIEQCMTEGAEDFLLKPVKLADVKRLKELIMRGGEAEQEKTSNLISPKRILQNNIFFFIIFFIITITIIFIV﹡

>BrRR18(Bra003782)

MTVGDLSSSTISSPELHVLAVDDSFVDRKVIERLLRISACKVTTVESGTRALQYLGLDGDDAPSALKDLKVNLIVTDYSMPGLTGYELLKKIKESSAFREIPVVIMSSENIQPRIDQCMTEGAEDFLLKPVKLADVKRLTELIIRNVEPEEEDKSKHSYPNRILQNNTDSSSSHDDVSSLDDDTPSSKRMKTRIHGS﹡

>BrRR19(Bra000199)

MNSGSCSSLMEVGYDDHHHHHGHEELHVLAVDDNLIDRKLVEKLLKISSCKVTTAENAIRALEYLGLGDQDQHIDALTNNDLKVNLIITDYCMPGMTGFELLKKVKESSNLKEVPVVIMSSENIPTRINKCLASGAQMFMQKPLKLSDVEKLKCHLMNCRS﹡

>BrRR20(Bra007242)

MEEELHVLAVDDNLMDRKLVERILKISSCKGKLYYSNFQFFIFLLNPKNQDSSMLWYYVTTAENGIRALEYLGLGDSQQADSSSTNNVMKVNLIITDYCMPGMTGFELLKIVKQESSNLKEVPVVILSSENIPTRINKCLASGAQMFMQKPLKLSDVEKLKCHLLNCRS﹡

>BrRR21(Bra014695)

MDEELHVLAVDDNLIDLTTAENGLRALEYLGLGDPQQTESLTTNSVMKVNLIITDYCMPGMTGFELLKIVKQESSNLKEVPVVILSSENIPTRINKCLASGAQMFMQKPLKLSDVEKLKGHVLNCRS﹡

>BrRR22(Bra001641)

MFSSGLRVLVVDDDPTCLAILERMLRACSYEVTKCNRAEMALSLLRKNKHGFDIVISDVHMPDMDGFKLLEHVGLEMDLPVIKSVVLKGVTHGAVDYLIKPVRIEALQNIWQHVVRKRRSVPEHSDGEDAADDNSSSVNGGKKWRSML﹡

>BrRR23(Bra022183)

MMNPSQGRGLGSGGGSSSGRNKGGEAVVEMFPSGLRVLVVDDDPTCLMILERMLRTCLYEVTKCNRAEMALSLLRKNKHGFDIVISDVHMPDMDGFKLLEHVGLEMDLPVIMMSADDSKSVVLKGVTHGAVDYLIKPVRMEALKNIWQHVVRKRRSEWSVPEHSGSIEETGQQQQQRGPAVSEDAADDNASSVNNEGNNWRSSSNNSRKRKEEEGDEQGDEDASNLKKPRVVWSVELHQQFVAAVNQLGVEKAVPKKILELMNVPGLTRENVASHLQKYRIYLRRLGGVSQHQGNLNNSFMTGQDASFGPLSSLNGFDLQALAVTGQLPAQSLAQLQAAGLGRPAMVSKSGLPVSSVVDERSIFSFDNSKPRFGDGIGGHQTQQPQMNLLHGVPTGMEPRQLAGLQQQLPVGGNRMSIQQQIAAVRAGHSGMLMPQQQPFPRGPPSIRQPMLPNRITERSGFSGRSSVPESSRVLPTSYTNLATQQHSSTSVAFNSFQQELPVNSFPLPSAPGLSVPTQVRKPHSSSSSYQEEVNSSEAGFATPSYDMFSNRHNDWDLRSIAFDAHQDAESVAFSNSEAFSFSSMSRNNNNNATVAATDLGRNQQQTLSGMVPHHQVYGNGGGGGSSVRVKSERDTAAMAFHEQYSNQEDLMSALLKQEGVAPVVDTEFDFDAYSIDDIPV﹡

>BrRR24(Bra001643)

MMNRGLGSGGGSSSGKNQGGEAVVEMFPSGLRVLVVDDDPTCLMILERMLRTCLYEVTKCNRAEMALSLLRKNKHGFDIVISDVHMPDMDGFVLLGHVGLEIDLPVIMMSADDSKSVVLKGVTHGAVDYLIKPVRMEALKNIWQHVVRKRRTEWSGVPTHSGSVEETGERRQQQQQREAVSRGEEDGADDNNSSSVNEGGNNNWRNSSSSSSRKRKEEEGGEEQGDEDASNLKKPRVVWSVELHQQFVAAVNQLGVEKAVPKKILELMNVPGLTRENVASHLQKYRIYLRRLGGVSQHQGSLNNSFMTSQDASFGSLPTLNGFDLQALAQLPAQSLAQLQAAGLGRPAAMNSKPGLHVSSSIVDERSVFSFDNPKMNLLHGVPTGMEPRQLAGLQQHRMTIQQQIAAVRAGHSLQNNGMRMPLASQPQQPFSRPQQSSIRQPMLPNRSGFSGRSSIPESSRVLPTTSYTNLAAQQQHSMAFSNFQQELPVNSFPLASAPGLSVRKPHSSSSSSSSSYREEFNSSEAGFSTPSYDMFSSRQNDWDLRSMLSPHQDSQAYSSSSMSRNNNTAVAATDHSRNHQQTPQGMVSHHQVYGNGGGSSVKVKSETMGFHEQYSNQEDLMSALLKQEGIGPVDTEFDFDAYSIDDIPV﹡

>BrRR25(Bra033527)

MLNPGQGRGPDSGVAGGSSNSDPFPAGLRVLVVDDDPTCLMILERMLRTCLYRVTKCNRAEIALSLLRKNKNGFDIVISDVHMPDMDGFKLLEHVGLEMDLPVIMMSADDSKAVVLKGVTHGAVDYLIKPVRIEALKNIWQHVVRKKRNEWNVSEHSGSVEETGQREDGDNNSSSANNEGSWRGSRKRKEEEVDEQGGDDKEDTSSLKKPRVVWSVELHQQFVAAVNQLGVDKAVPKKILEMMNVPGLTRENVASHLQKYRIYLRRLGGVSQHQGNMNHSFMTGQDPSFGPLSTLNGFDLQALAAAGQLPAQSLAHLQAAGLARPPSLTKPGMSVDQRSIFSFENPKIRHGQMMNSGGGGNKQMNLLHGVPMGMEPRQFTGGGQMRVQQQQQQLSGGRAVGQNVQSSGMMMPVGGGPSMLQQQQQVMLSSSVPRRSETSSSSRVLPAAATTQSVVFNNFSSELPRNSFPLASAPGISVSYQEEVNSSDAKGGAGFGNPSYDIFNDYPQQHNNNNDWDLQNIGMVFNSHQDTTTASAAFSSSSSTQRQRAEHVQNHHQQQQLPSQSRNHMNGGGSVRVKSERVAETVTCPPATTLFQEQYNQEDLMSALLKQEGLPLVDNEFDFDGYSFDNIPV﹡

>BrRR26(Bra012743)

MLTPGAVGGSSNSDPFPSGLRVLVVDDDPTCLMILERMLKTCLYRVTKCNRAEIALSLLRKNKNGFDIVISDVHMPDMNGFKLLEHVGLEMDLPVIMMSADDSKSVVLKGVTHGAVDYLIKPVRIEALKNIWQHVVRKKQNVSEHSGSVEETGGDRQQQRDDDDDDGGDNNNSSSGNNEGNLRKRKEEEQGGDDKEDTSSLKKPRVVWSVELHQQFVAAVNHLGVDKAVPKKILEMMNVQGLTRENVASHLQKYRIYLKRLGGVSQHQGNINHSFMTGQDPSYGPLNGFDLQGLATAGQLQAQSLAQLQAVGLGQSSSPLIKPGITSVDQRSFFTFQNSKSRFGDGHGPMMMNGGNKQTSLLHGVPTGHMRLQQQQMAGMRVAGPSMQQQQQSMLSRRSVPETRSSRVLPGATHSAFNNSFPLASAPGMMSVSDTKGVNEFCNPSYDILNNFPQQQHHNNNNSVNEWDLRNVGMVFNSHQDNTTSAAFSTSEAYSSSSTHKRKREAELVVEHGQNQQQPQSRSVNPMNQIYMNDGGSVRMKTETVTCPPQATTMFHEQYSNQDDLLSALLKQEGLLDTEFDFEGYSFDNILV﹡

>BrRR27(Bra023972)

MTLEQDFEAVDQFPVGMRVLAVDDDQTCLRILETLLHRCQYHVTTTDSAQTALELLRENKNKFDLVISDVDMPDMDGFKLLELVGLEMDLPVIMLSAHSDPKYVMKGVKHGACDYLLKPVRIEELKNIWQHVVRKSKFKKMKSIVINDDHSQGNSDQNGVKANRKRKDQFEEVEEEDEERGNENDDPTAQKKPRVLWTRELHNKFLAAVDHLGVEKAQPKKILELMNVDKLTRENVASHLQKFRSALKKITNEANQQANMAAIDSHFMQMSALKGLGGFHNQRQIPLGSGQFHGGAATMRHYPLGRLNSFGGVFPHVSSSLPRNHNDGGYVLQGMPIPPLDDLNNKAFPSFTSQQSSLMVAPNNQLVLQGHQQSSYPSLNPGLSPHFEINKRLDDWSNALLSTNIPQSGVHSKPDALEWNHFCNSDAAQAGFIDPLQMKQQPANNLGPMTDAQLLRSSNPIEGLFVGQQKLENGSMPSNAGSLDDIVNSMMPKEQSQAELFEGDLGFGWHNSSLRTCI﹡

>BrRR28(Bra004245)

MDKGFSPVGLRVLVVDDDPTWLKILEKMLKKCSYEVTTCGLAREALRLLRERKDGYDIVISDVNMPDMDGFKLLEHVGLELDLPVIMMSVDGETKRVMKGVQHGACDYLLKPIRMKELKIIWQHVLRKKLQEVRDIEGCYEGGADWFTRNDEAHLLGGGGEDVSFGRKRKEFDFEKKLLLQDESDPSSSSSKKARVVWSFELHQKFVNAVNQIGCDHKAGPKKILDLMNVPWLTRENVASHLQKYRLYLSRLEKGKELKCYSGGVKNMDSPPKDSEFNTGHQSPGKNSYAFSGGSSDPKQLASSSVSDPSSDVHMPPKAKKTRVEFDPPISSSSAFESLLPWSDVPDPLESKPQILYGSSFLQQQPLPSQSPYVANSAPTLMEQEMKPSYETSVNADEFLMPQDKNSTVILQDLDLSAPSAISSINVTNDTESILRSLSWELPESHHSGFIDTDLDFSWLQNEHFLANTSGNFQFQDYSCSPSLLSELPPHLWFGNEPDEYTLMVDHGLFIS﹡

>BrRR29(Bra004076)

MEKGFSPVGLRVLVVDDDPTWLKILEKMLKKCSYEVTTCGLAREALRLLRERKDGFDIVISDVNMPDMDGFKLLEHVGLELDLPVIMMSVDGETSRVMKGVQHGACDYLLKPIRMKELKIIWQHVLRKKLQEVRDIEGCCYDGGADWFTQGQFLGGGEDVSFGKKRKDFDFEKKLFQDESDQSSSSKKARVVWSYELHQKFVNAVNQIGCDHKAGPKKILDLMNIPWLTRENVASHLQKYRLYLSRLEKGKEIKCYSGGVKNMDSPPKDAEINSGHQSPGKSSSYAFLKATETDPKQLASASVSDPTSDIHMPQKAKKTRIGFDPPISSGVFGSLLPWNDVPDPLESKPPILYENSFLQQQPLPSQSSYVANSAPSLMQEEMKPSYVNPDEFLMPQNKNSTVILQDMDLSAPFSSNATSNTESIPGSLNWELPEAHHSGSLDTDLDFTWLHGEHFFANSGLQNFQFQDYSNSSSTSLLSELPPHLWYGNDRLPDPDEYTLMVDQGLFIS﹡

>BrRR30(Bra032035)

MTVEQQDCVALDQFPVGMRVLAVDDDQTCLRILESLLHRCQYHVTTTNQAQKALELLRENKNKFDLVISDVDMPDMDGFKLLELVGLEMDLPVIMLSAHSDPKYVMKGVTHGACDYLLKPVRIEELKNIWQHVVRKNRGSNNGDKKDGSGNEGVANSDQNNGRANRKRKDQYNEDEDEERDDNDDPSSQKKPRVVWTVELHKKFVAAVNQLGFEKAMPKKILDLMNVEKLTRENVASHLQKFRLYLKRISGNQQAIMANSDLHFLQMSNGLDGFHHRPIPVGTGQFHGGAAAAGMRPFPPNGILGRLNTPSGMSGVRNLSSSPSSGMFLQNPTDLGKFHHVSSLPLNHIDGGNILQGLPMPLEFDQLQTNNNKSIIAGNSMAFPIFPTQQQSSLPNNNNHLVLEGHPQAPPSAFPGHQINKRLEHWSNAVSSSSSTLPPPGQNSNSLISHQFDASSSSYSIPFCDSTIPLNPALDHTNPRAFYRATDMDSSANVQPGVYYDSLQMRKSGNYGPTTDAMLSSNNPKEGFTVGQQKLQSGFMGGEAGSLDDIVNSTMKQEQSQGDLSEGDLGYGGFSSLRTCI﹡

>BrRR31(Bra026635)

MTISDQFPCGLRVLVVDDDASCLIILEKMLLRLMYQVTICSQADVALTLLRERKGCFDLVLSDVHMPGMNGYKLLQQVGLEMDLPVIMMSVDGRTATVMTGINHGACDYLIKPIRLEELKNIWQHVVRRKCTINKNSISSSSSSLGSLFSVSGVSEGSLKRRKNKRRVDSEEDDLLDPGNSSKKSRVVWSMELHQQFVKAINHLGIEKAVPKRILELMSVPSLSRENVASHLQKYRLYLKRLSGAASQSRDAESMERYENIQAMVSSGQIHPQALAALYGRPIDNHMSGGFGVWIPTDNHLGGSNVSSASNRCFGALDSPSSVAASMSVHGLSSSGNVRQQGNGFSNNTDYRIRQGNGSGISEESWILGRPLRQRKA﹡

>BrRR32(Bra020390)

MESVSNEEGRSDQFPVGMRVLAVDDNPTCLRKLEELLLRCKYHVTKTMESKKALEMLREKSNMFDLVISDVEMPDTDGFKLLEIGLEMDLPVIMLSAHSDYDSVMKGIIHGACDYLVKPVSLKELRNIWQHVVKKNIGSYKKIIAPSRHLLPTSEYAPRGSGKRKEKADDSGDGDDDSDRDDEEDGSEQDGDESSSRKKRRVVWSQELHQKFVHAVQQLGLDKAVPKKILDYMNIEGLTRENVASHLQKYRLYLKKLDEGQQHNMSQDAFGSRDSSYFHMAQLEGLRDYSSTRQLSSSSLLTRSSLTKFHPSVYSSVNLQGSNSSSFIPPGHHQSSSSSANPFGTYHSPLLARSQNVNLSPLEPLQFPRSKCSPYMGDFKGIADRGIGSSFLDSRMSFGSSSTSLPCATSNNLMLQENFGVSDGNQSCLNGLSSFPSHHSWQGNLKTTTRFPSHSLPLNHAFGQDQMTCGGTGLGDYNTSLVSADSHVGVLQCEPPFLGDFMQNMNTHKWEEQNCTMMNNTFGNVDYPLPVDNNMVFRDNNATRSKGVDDSLMMSPIEDSATTLNSRECVGNVTMMDPEMRSSTKLENDLVDNQNDVFDDIMNEMLKQDENNGMVSVAARFGL﹡

>BrRR33(Bra014172)

MSIAHITEDGDKALFLQQETSEINSPLNEFPPSTNVLVVDANLSTLLDMKEIMERCAYHVTAYADAEEAIAFLTKCKHEINIVIWDYHMPGINGLQALAIIGSKMDLPVVIMSGDDQTESVMNAMVHGACHCVMKPVRKEIIATIWQHIVRKRMMSKPGLVPPVVVHGDYSKQEKDDSVTVDQDDSEESIDKIEEKATQKQTMICIEETQPMQSHLVKSNGSDQDDDDSRSVRNYNYEQSIDKKKERDLKRPRISWTGDLQQKFLEAIDIVGGPKKASPKVLLKCLHDMNIEGLTRNNVSSHLQKYRLSLEETKIPQQFPETGWSSLSRPSPFLGMNNGFIAPTSLRNGPAVYPVQDNQYQNGYLAINNNQFVTNNMHGFPYSENDHHLQQQHQQRQYQLSNQMMNYMMRNEPQQAYNSIGLTDLEPNIYPSLPYYPNEFLFDGYNFSN﹡

>BrRR34(Bra032275)

MSEASRTDDGDRTLFKQRETSQISSFLNEFPASANVLVVEPNFVTLRKMKNLMIKYGYQVTVYADAEAALAFLRNCEHGINLVIWDFHMPRINGIQALKIICTKMDLPVVIMSDDDRKTSVMQATVHGACYYVMKPIRKEIIATIWQHIVRKRMMSKSGLIPPVQLDAVQNHDGFKENKDDSMPVDQGNSEQNINMIGEKAEKKPQIGENLPIQSDSVQNNGSDQDNNDSWTKSPYNSEQNMDGEERKQPKTRVVWTNDLQEKFLKAVDILGGARKANPKPLLKMLEDMNIKGLTRRHVCSHLQKYRLSLEGKEITQQMQEFGWSSACTTSPLLGLNNVHTATSSLINGGASYPVQENQYQNGYMEVNNNHAASSTLMNGRATYPVQDNQYQNGYLGVNNNQVMTNTMPYDFDHDHYLQKQKPEKASSIDIPEDLGLAYTMPRLPYNLGHGNHLQHEQQHQLSHQWNNVMSNNEPELPSSNGVTGVGGTYPSLPYDPNEFYNYNQ﹡

>BrRR35(Bra041027)

MSVSSNILKENSRDLLREEEPGDDEVEFPINDEDEDFSITSIRIVLVDSDPESLCLMKNLMTQYSYQVRDFKNGAEAIAFLMMSKHEIDLVIWDFHVPEINGLEALKTIGKEMDLPVVIMSHEHKKKTVMESTKRGSCNFLLKPVSKEIIAVLWQHVYRKRVSIYSVESNPEENVGLDQDDIDLYQTNSNSGEQTSSYQKEGKNKKPRMTWTPELHQLFEKAVEKMGGVEQAVPKQILKCMQEEKDAEGLTRNNVASHLQKYRLNSGKKSSMIQETREDSEWRNAGPNTALTASKPLPNSIFGLHTRVPYFANDQDARNGPMQYPSTNYFTMDNGHFMTNSFANLPYTDSFHQQQQQQFQHQQYSNSSLQLPSVITKQEFPYVSAALENPDLIANENSLYMDLGDYLQEGLSDFDKTNRY﹡

>BrRR36(Bra005928)

MAFAQSFDNQSSDLRINVMVVDDDPVFLGVVSRMLEKFKYRDPSFKEISVIAVKDPIEALSTLKTQRHNIDLIVTDYYMPHMNGLQLKKQITREFGNIPVIVMSSDSNIEHESLACGAKCFLPKPIRPTDIPQIYQVALTYKRNDKSILWTEHNYRDTDVSIPQQIQLHTEQANVLKTNNKRFSPISDSRPVNSSNGSYVSTDGSGENRKRKSNGGSGDDSRPLKKPKIKWTDCLHDLFLQAIRYIGLDKAVPKKILEYMNLPYLTRENIASHLQKYRIFLRKVAEQGFSCSRMLPTKGIDSIFLQAHLRDPCYNNYASSSSSLYDTTINNRSFYSKPIQSYGQSRLLSNTAEPVRFNQMPYNYMNRSSTYEPRGIGSNLTIPTISNLSFPIQPSQNEGRRSLFEPTVMANKTVQTSQAMGFGQHGLSAINGNSFNNNMVSSYGRLTPTQPGMMSYENLTPSQPGVRSYVNSTFDQPGMNNQRSLTPDQQGMRSYRSLTSNQLGVNMNGSFSQTPNQPGMSSYGISTSNQPRMNNHGSLTLDQQVMSSNGSLTSNQLGMSSHGSLYPNQPGLNSYGSVTYNARVNIHESLTPNQPGASNFSYGMQMFLNNENTTYKPQAHDNATTQPNLEIPTLENLSLCDELLCEISNFQIDHNKQQEEAVSTNKFELPANFETELNQFFSLEENGDGNFVNINQGRSDGETSNIVAAPETNYPVFNMNPNHEQEQGVPGFVDWSSLDPKDFANEYDFVDSLLTNDMN﹡

>BrRR37(Bra009284)

MAFAQPFYNQSSLLRINVMVVDDDPVFLEIMSRTLEKLKYRDSSTMEITVIAVKDSREALSTLKIERNNIDLIVTDYYMPDMNGLQLKKQITQEYGNLPVIVMSSDTDKEHESLTCGAMGFIQKPIKATELTKFYQLALKCKRNGKSTLWTENNHNDTDVSISQQIQFFPEQDNLMMTKTKKFSPRPDSRSMNSSNGTCVSTDASRKNKKRKANGGSGDGVESLSQPSMKSKITWTDDLHDLFLQAIRHIGLDKAVPKKILEFMNVSYLTRENVASHLQKYRQFLRKVAERSSLCSSNMLPSNGIDSIYPYPHTREPYYNNYTSSSSWYGTSLSNNRSFYSNPGHGLGQSRLLSNTSDPVRSNQMPHSYMNRSSTYDTHRIGSNLTLPVESNLNYSSQNVGRRSFLEPTANKTSQTSQALGFEQHGLSAINGSGFNNNTLISYGSLAPNQLGTNSYKGSISTQQGMTNGSLALNQPGMSAYGSSTSTQPGMSSHISLTPNQPGRNSYGSLTAPQPRMSTHESLSPNQQGISSHKNLNSSQLGMDSNGSLSHDQPRMSSYENLTSNQLGLSSHGFLTPNQPGLKSYGSATHNVGLNSFEGLTTHQPGSSNFSYGLQSFLNNENTAYEPQPLTHAQAAAQTNIEIPQQENFSLFDELANINELLCDISNFELDHNKQQEAVSTTQFELPANVSTEMNQFFSLEDDDWTFVNTNQGFSNGETSNNVAPETNSQTFNMSTNHDQEQDAQDFVDWSFLNPEDLANEYDFMDSLFNGMN﹡

>BrRR38(Bra028705)

MTFAQSLNNQSSVLRINIMVVDDDPVSREIVSRMLERSKYRDPSMEITVIAVRDAREALSTLKIQRNNIDLIVTDYYMPGMNGLQLKQQITRQFGNFPVIVMSSDTNKEQESLACGSVCFLPKPIKPTDLPKIYQVAFTYKRKGKSISRTEHNHMDTNVSIPQQIQLLPEQANVSKTKKNKEFSSKSDSRSVNSFNGSCDSTDGSRKNRKRKSNGDFGDDDESLPQPSKKSKLSWSDYLHDLFLQAIHHIGLDKAVPKKILEFMDVSYLTRENVASHLQKYRNFLRKVAENSGMLHGRGMEPYHSNYTTSSSWYDTGLNNKSSYSKPRHGLGQSRLLSNTCEPVRFNQMPYNHMNRLSTYEPHRTGSNLTMPIKSNLSFSTQPLQNEGSRSFLEPTVTANKTGQTSQVLGFGQHGMLAINGNNFNDNTMSSYASSTSNQPRINSHGSSTPNQPGLRSYGCSISNQPGMSSYASVSPNQLGMSSYGSLTPYQRGMSSLGSFTPTQPGMSSHRSLTPTQQGMSSYGSLSPSQPGVSTQPGMSSKGSLTPTQRGMSSYGSLTSYQPALSTHGSLFPNQPGISSHTSLPPTQPGMSSFGSLTTNHSGMSSYESLTPTQPGPSNISYGLLLNNENTAYKPQPHASTTIQLDNLSMYDDLGNINEIPCDLSNFDFDHDKQQEEAVSANKFEIPANLETELNQTSSLEEDGDWTFLNISQGHFNEKTSNTFAAPETNDPTFNKNPNHAQNLANENDFMDSMFTNDNSMFTNDMN﹡

>BrRR39(Bra001099)

MATTSTSTGDIKKTKSVEVKKKLNVLIVDDDTVIRKLHENIIKSIGGISQTAKNGEEAVNIHRDGNASFDLILMDKEMPERDGLSATKKLREMKVTAMIIGVTTLADNEEERKAFMEAGLNHCLAKPLSKAKILPLINNLMDA﹡

>BrRR40(Bra040204)

MATKSMGDIEKIKKKLNVLIVDDDPLNLIIHEKIIKAIGGISQTANNGEEAVIIHRDGGSSFDLILMDKEMPERDGVSTTKKLREMEVKSMIVGVTSLADNEEERRAFMEAGLNHCLAKPLTKDKIIPLINQLMDA﹡

>BrRR41(Bra020537)

MAGEVPNLEASKLTALVVDDNFVNQSVHHKLLDRLGIKNDVVSNGQEAVDVHCSGRNYDLILMDMDMPIMNGIQATRRLREMGIESKIAGVTTRAEEEEVKEFMEAGLNDFQEKPLTISKLVSILHNLELYVQT﹡

>BrRR42(Bra036579)

MADEVPSLAESKLTALVVDDNFVNQTMHHKLLDRLGIKNDVVCNGQEAVDVHCSGRNYDLILMDMEMPILNGIQATKRLREMGIESKIAGVTTRANEGDKKEFMEAGLNDFQEKPLTISKLLSILHRLDFHVQT﹡

>BrPRR1(Bra035933)

MDLNGECKGGDGFIDRSRVRILLCDNDSKSLGDVFTLLSQCSYQVTSVKSARQVIDALNAEGPDIDIILAEIDLPMAKGMKMLRYITRDKDLRRIPVIMMSRQDEVPVVVKCLKLGAADYLVKPLRTNELLNLWTYMWRRRRMLGLAEKNMLSYDFDLVGSDPSDPNTNSTNLFSDDTDDRSIRSTNPLRGPLSRQEKECPAATGSVDGTATSAPPPLNHLPGSHHEPNPEKFSSVPKKSRLKIGESSAFFTYVKSTALATNCQDPPHVNGNGSLHLHPGVVAEKLQVVASEVINKPKQTHRSRETEKNLQNGGATEELHGRSYQERNQVAVNRSKDSSQVAYPYYMQGVMNQVMMQSAAMMPQYGHHQHPHYPPNHLNGMTGVPYYHHHPMNTPLQHNQMSQNGQMSMVHYHPSSNEVRASKLDRREEALLKFRRKRNQRCFDKKIRYVNRKKLAERRPRVKGQFVRKMNGVNVDLNGQPDYDDEEEEEDEEEEENRDSSPQDDAQGT﹡

>BrPRR2(Bra012964)

MDLNGECKGGGGDGFIDRSRVRILLCDNDPNSLGEVFTLLSQCSYQVTSVKSARQVIDALNAEGPDIDIILAEIDLPMAKGMKMLRYITRDKDLRRIPVIMMSRQDEVPVVVKCLKLGAADYLVKPLRTNELLNLWTHMWRRRRMLGLAEKNMLSYEFDLVGSDPSDPNTNSTNLFSDDTDERSIRSTNPQRGSHQEKEWPVPTGSVCAGDGAADGTATSTPPVAIIEPPLNHLPEPHHEPTKRNTNPAQFSSVPKKSRLKIGESSAFFTYVKSTVNGNGSVHPGMAEKLQAVASEVINNAKQTRGGRETEKNKAQGENLVNGTLERSRTLPTPMELHGSRSYQEVPNSIERSRTLPPPMELHGTRSCYQEGSMDDARVAAAKDSSQFPAQNAYPYYMHGVMNQVMMQSAAMMPQYGHHQHPHCPPNHLNGMTGFPYYHHHHQMNTSLQNGHVPLQNGQMPPMVHHHHSWPQVGNHPSPNEVRVTKLDRREEALLKFRRKRNQRCFDKKIRYVNRKKLAERRPRVKGQFVRKMNGVNVDLNGQPEPDSADYDDEEEEDEEEEENRDSSPQDDALGT﹡

>BrPRR3(Bra002512)

MCFNKNDITNEVVTERQAFGSSEEDDSRVEDTAGNANNLLQITQQQQPSAPVVNWERYLPVRSLKVLLVENDDSTRHIVTALLKNCSYEVTAVPDVLEAWKVLEDENSCIDLVLTEVVMPVNSGTGLLSKIMSHQTLKNIPVIMMSSHDSMVLVFKCLSNGAVDFLVKPIRKNELKNLWQHVWRRCHSSSGSGSESGIHNKKSVKTESTDEGSEDDASMSDEDNGNDDNGSNGLSNGEGGSDHGSGTQSSWTKRASDVNVGTYGNECERLKKLKEVEDENGQIGMGSQAGQCMSKKAVIALERNNDDLLNRSSGNSQVETKTPSSNPEDLQSLELTFTKPKEAGDQRVGGDRSVLRHSNHSAFSKYNNGATSANKAPEENVGSCSPQDSSVAKIIGSSSSSDNPSNQQSSGSDRAAQREAALMKFRLKRKERCFEKKVRYHSRKKLAEQRPRIKGQFIRKMDASKSGNECQSSDDSSSKIGKED﹡

>BrPRR4(Bra009768)

MGEVSDEVVEVTVVEKAPEAGGGKLTRRKMRRKDAAEGGDGLVTWERFLPKISLRVLLVEADDSTRQIISALLRKCSYRVAAVPDGLKAWEMLRGKPESVDLILTEVDLPSISGYALLTLIMEHDLCKNIPVIMMSTQDSVNTVYKCMLKGAADYLVKPLRRNELRNLWQHVWRRQSSLAPGSNFPVDESLGQQKPEGASANNSTSNQVNGFQREEQPVIGNGGGGGGDDQSSCSRPEMQGESADVVEDIPRVSSKEAIDFMGASFRRNGQSHREESVAKHDTSRIELDLSLRRPDTSENHQPSLHPSSASAFTRYVHRPLQTQCSVSPLVTDQRKNVAASGGDDNNTVLINQYNVSEPPPSAQRRNEASFYNSSDSPGPPFSNQMNSWPGQGSYPTPAPIIHFPGPNHTSTMAPASVSPSPSSVSPHEYSSMFHPFNGKPEGLQERDGSMDMEERRHVSSATEHSGTGNHCSTNYIDYHHQQQQLLEKRSEEGYSSSVGKLQQSLQREAALNKFRMKRKERCFEKKVRYESRKKLAEQRPRIKGQFVRQVQSTETSTQEAPQ﹡

>BrPRR5(Bra036517)

MREKSDEVVEVTVVEKAAEAVGVKSARRRRVQRKDAAEGGDGLVKWERFLPKIALRVLLVEADDSTRQIISALLRKCSYRVAAVPDGLKAWEMLKGNPESVDLILAEVDLPSISGYALLTLIMEHDVCKNIPVIMMSTHDSVNTVYKCMLKGAADYLVKPLRRNELRNLWQHVWRRRQSTLAPGSFQLDESLGHRKPEGAQSSCTRPETEGESADVEKDSSKEAIDFIGASFTRNEQHNREESVRIELDLSLRRSSLHPSSGSAFTRYVHKPLQTQCSVSPLVPDQRKNVTESEDGNIVVTNQYKSSEPPPSAPRRNEASFYNSADSPGPPSWPGQGSYPTTVPIKSIQFTSPNTTAASLSPSPSSISPHEYSSMFHPYNGNKPEGLQEQDVEERRHVSSANEHSTIGNHCTTSYIQDQQLVEKKNEEGYSSSVGKTKQSLREAALNKFRMKRKDRCFDKKVRYESRKKLAEQRPRIKGQFVRQVQSTETSTQQAPQ﹡

>BrPRR6(Bra029407)

MQEMSDEVVEVTVVDKASEADGGKPTRRRMRRKDAAEGGDGLMKWERFLPKISLRVLLVEADDSTRQIISALLRKCSYRVAAVADGLKAWEMLKGNPQSVDLILTEVDLPSISGYALLTLIMEHDICKNIPLIMMSTQDSVNTVYKCMLKGASDYLVKPLRRNELRNLWQHVWRRRQNAPGSFPLDESVGHEKPDGASANNSTSNQENAFERDQRPVIGNGGDDQSSCSRPEMQGESADVEDSTKEAIDFMGASFRRNTQRNREESVARYESLIELDLSLRRPNTCENQSSGEKPSLHPSSASAFTRYVHRPQYSASPLVLDQRKNVAASEDDNINQYNSSEPPPNAPRRNEVSFNNRADSSQMNSWPGQGSYPTPVPINSIQFNTAAMAPASLSPSPSSVSPHEYSSMFHPFNGSMDAEERRHISSTTEHSAIDNHCSANSSSVGRTQQSLQREAALNKFRMKRKDRCFDKKVRYESRKKLAEQRPRIKGQFVRQVQSTETSTQEAPQ﹡

>BrPRR7(Bra028861)

MNGDENGEGEGHSGGEDKANGVPMDVRNGAAGQGSSPGLQVPLSQQTQATVCWERFLHVRTIRVLLVENDDCTRYIVTALLRNCSYEVMSSHDSMGLIFKCLSKGAVDFLVKPIRKNELKILWQHVWRRCQSSSGSGSESGTHQTQKSVKSKNIIKSDNDSGHSGENENGSIGLNASDGSSDGSGAQSSWTKKAVEVDDSPRAVSLWDRADSTCAQVVHSNPEFPNNHLVAAPAEKETQEQDEKIEDVTMGRDLEISIRRNDDPKDEPLTKTTGIVRQENSFEKSSSKWKLKVGKGPLDLNSESPSSKQMHEDGGSGFKALSSHLQDNREPEAPNPHCKTLDTSEAAVKNSEELMDVEHSSKRHRGTKDDGAIVREDRNVLRRSEGSAFSRYNPAANNNKLHDNNCQDITKKTEAACDCHLNMNEGLRNNHHSRVGSNNLEMSSTTGAPKVSSAGSSSVKHSSIQPLLPCDHRNHHHHSSYNPAHIPEQKLPPQRGSSNVYNEVIEGNNNNKVNYSVNGSGSGSGHGSNDPYGSSNGMNAGGGMNTGSANGDGGGSGDGSGSGSGSGSGNVADENKMSQREAALTKFRQKRKERCFRKKVRYQSRKKLAEQRPRVRGQFVRKTADATNDNDTKNVEDS﹡

>BrPRR8(Bra009565)

MNVNEEGEGSRYPVTDQKPVETKERLSGEDKANGVVMDVRNGSAGGAGGGLQIPISQQTPATVCWERFLHVRTIRVLLVENDDCTRYIVTALLRNCSYEVVEVANGVQAWKVLEDLNNHIDIVLTEVVMPYLSGIGLLCKILNHKSRRNIPVIMMSSHDSMGLVFKCLSKGAVDFLVKPIRKNELKILWQHVWRRCQSSSGSGSESGTHQTQKSVKSKTIMKSDNDSGRSGENENESNGLNASDGSSDGSGAQSSWTKKAVEVDDDSPRAVSPWDRVDSTCAQVVHSNPEVPGNHLIAAPAEKETQEQDEKFEDITMGRDLEISIHGNCDLTLEPKDEPLTKSTGVGKGPLDLNSESRSSKQMHEDGGSGFKATSGHQLQDNREPEAPTTTHCKTVDTNEAAIKNPEEPMHVEHSSKRHRGAKDDETIVRDDRNVLRRSEGSAFSRYNPALNNNKLSGGNLGSNARHDNNCQELIKRTEAACDCHSNMNESLPSNHHSRVGSNNVEMSSTTVNNAFTKPGAPKVSPAGSSSAKRSLFQPLPCDHHHSSHNLVHVPERKLPPQYGSSNVYNETIEGNNNNNNTVNYSVNGSGSGSGHGSNDPYGSSNGMNAGGVNMGSENGAGKSGSGDGSGSGSGNVADENKISQREAALTKFRQKRKERCFRKKVRYQSRKKLAEQRPRVRGQFVRKTAAATDDNDVKTPRDS﹡

>BrPRR9(Bra004507)

MAETMVSRIKSPEVVQWEKYLPKTVLKVLLVESDDSTRQIITALLQKCSYKVVAVSDGLAAWETLKEKSNEIDLILTELDLPAISGFALLALVMEHEACKNIPVIMMSSEDSMTMVLKCMLKGAADYLIKPMRKNELKNLWQHVWRRLAVRDGHNGHGLSLPASQQNLEDSDETSADHSDQGSGAQATTSCYGNNKLMEDVTMDLIGGIDKRAECFYGDNTRDEYVGPELGLSLKRSCSGSFEKQDQTTKQQKLSLSDESVGITLTCRYENSQAAEKGEVGVEPSSSGEPKTPSESHEKLRFDYGSATTSSNHENMGSSSLSGQNELSFRNQVGSESTNDVKAKEQEEEGCGLSVEQRRSQREAALMKFRLKRKDRCFDKKVRYQSRKKLAEQRPRVKGQFVRAVMNSDASK﹡

>BrPRR10(Bra040484)

MGEVVVLSSDEGTMETMMSRGKSSEVVRWEKYLPTTVLRVLLVESDDSTRQIITALLQKCSYKVVAVSDGLAAWEILKEKTHNIDLILTELDLPAISGFALLALVMEHEACKHIPVIMMSSQDSMTMVLKCMLRGAADYLIKPMRKNELKNLWQHVWRRLTLRGDLTANGPSLPASQQNVEENDETCADSRYHSDHGSGSQAISDNGEDKLMGDVKPLFESFDVTMDLIGGIDKRSECYYGDNAREQHVGPELGLSLKRSCSERSLEKNQDESKHQKISLSDASAFSRYENGKAGEKAVVVAVEASSSAEPKTPSESHEKLFRCDHGSATTSSNHENIGSSSVSGHNQFLQSGTTKQKQESLFPVESNRPKASKEVEVGCQSTNEGTVTAGGQSRSSSTREKAKEEEEEGEGGVTAQQRKSEREAALMKFRMKKKDRCFGKKMASSSPQHCHIIEVNRGKSAEESTTILASKACGEAPCGFSDLNNASGDAQERNASMRKLCIAVVLCLLFMTVEVFGGIKANSLAILTDAAHLLSDVAAFAISLFSLWAAGWEATPRQTYGFFRIEILGALVSIQLIWLLTGILVYEAIIRLLSETSEVNGFLMFLVAAFGLLVNIIMAVLLGHDHGHGHGHGHDHHSHGVTVTTHHHHHGHGEDKHHHHAHGDEDVTEQLLEKSEKRKRNINVQGAYLHVLGDSIQSVGVMIGGGIIWYNPEWKIVDLICTLVFSVIVLGTTINMIRSILEVLMESTPREIDATKLEKGLLEMEEVVAVHELHIWAITVGKVLLACHVNITPEADADMVLNKVIDYIRREYNISHVTIQIER﹡

>BrPRR11(Bra013277)

MVFTSNDISKWENFPKGLRVLLLDCGDGISAAETRSKLESMDYIVTTFTDGTEALSAVIKSLESFHIAIVEVNTSDENESFKFLEAAKDHLPTIMISNDHCITTTMKCIALGAVEFLQKPLSPDKLKNIWQHVVHKAFNDGGTDVSESLKPVKESVVSMLHLDTDMTIDEKDSAPLTPQLKQVSRLLDGGDRQENINSSTEKENMEDQDIGESKSVDTTNHDDNVIVKEEKLDGETGDTKSEKTDSVKFQKKEDETTKHNNKSTGIKNLSGNKPSRKKVDWTQELHKKFVQAVDQLGVDQAIPSRILELMKVDGLTRHNVASHLQKFRMHRRNILPKEDHNHRYIQSRENHRQIQRQYNGFQQQHRPVMAYPVWGLPGVHPPGAVPPLWPPPLQSSGQLPPWHWRPPYPTVNVNAWGCPIVPPVTGPFSSPTVTGTFSTPPATQLDEEMVDQVVKEAISKPWLPLPLGLKPPSAESVLAELSRQGISAVPSSSSSINGSRRLR﹡

>BrPRR12(Bra012623)

MVFTANDLSKWESFPKGLRVLLLDSAAETRSKLESMDYIVTTFSDESEALSAVVKSPESFHIAIVEVNTSAEDESFKYLEAAKGLLPTIMISDDHCIATTMKCIALGAVEFLQKPLSPEKLKNIWQHVVHKAFNDGGASVSESLKPVKDSVVSMINIDTNMTIDEKDPAPSTPQLRQVSRLLDQENRNCSVENVNSPTEKENTEDHDIGESKSVDTTNHEDNVIVKEENGDGEKEEEQGQTEEHKQEEGETGDSVNFNKKEDETNKPINKSSGNKNLSSNKASRKKVDWTQELHKKFVQAVEQLGVDQAIPSRILELMKVDGLTRHNVASHLQKFRMHRRNILPKDDHNHRWIQSRENHRQIQRQYNGFQQQHRPVMAYPVWGLPGVHPPGAVPPLWPPALPSAGQLPPWHFKPPYPTLNGNTWGYPVGPPVTGTFFPPPITGTFSTHPANQLDEEMIDQVVKEVISKPGMPLPLGLKPPSPESVLTELSRQDISAVPSSSASCQINGSPRLR﹡

>BrPRR13(Bra037427)

MLQKWKPSGDESGGPDRDLPNSRDMFSGNFPEGLRVLLFDEDRIYLQILEKHLEEFQYEVTTCHEEERATYLLCNHRNMFDIAIIEAHNLEGKIFRLISEIRSEIDIPIIITSQDDSRESVTKWMRNGACDYLIKPIRPEDLRLIYKYLVKKMELRGITVAEEAEEKAAAEKSSSVGDSTIRSPNRRKRNMLQTDEDPDHNRDSATKKRRVVWDDYLNGKFLDAVNSLGNNDVVPKKILERMNVSSLTRENVASHLQITTGLGSDLCPPRNPI﹡

>BrPRR14(Bra020652)

MLTGKFPEGLRVLVYDEDFQNLISLEKHLQYFQYKVTICNEGADAMHMLRNHMNTFDIAIIEAQNSAVDIFRLISEIASEIDLPIIITSKDDSVQSVINWMKIGVCDYLIKPIRPEDLRFIFKHVVKKMQVGKRVESEEKATAEKSSSVGDSTIRNPNKRKRSMFIDGQVGEKDQDHVRDSTTKKRRVVWDNELKKKFLDAMEDLGPEAVPKKILERMNVVGMTRENVASHLQKHRMLLNRQKSHNEKDEKKRSLLSPQGGLHSGEGGSNIQFSTQHISNIPHQPFRHHPDGVPVVVSTRNLLMTNQHHLQTSDFTSIENVEESLIFTEEDAEVSNLAFLFTQKSEEMSLSHLHEPVMATTMLSNDNQLFPNQQQMMNFHEPSILHTHSFPLSLTPSSFLDQKETIMMMNVDEGLQQWLLNEQEQPNLTDENRFSSINPRA﹡

>BrPRR15(Bra004306)

MAKEIAFDVTGNKISNSNGDTFILLIDHDIASISSLTSMLQQLSHKVISVNVASEAVSMLKKQMDIVLVIANTEMPHIDSHSFYTSLLTRDIPLILISPEGKKAKPSNSLEKRACYLLEKPISEKDINNMLQHVLSNKSQKLTKISIPKSGGGNMEKRINQMKAFREILRRQRPSSFLGKPLLKKSAYQERRNIANVERKNKTVYPVEFENKRNEGNNIDSNTGIRNNFWTYEHQMKFFSAGANLGEKDSHPKSLLGIMNDRTSKNEYPFTLSNIAKNFFADKNRIKERDTMALKFYQGTKMDLSRTSWFGNIPNSSSMEADRVPAATSNIPPCNISPTDTVSHTNLVSTSLNDNNFLDHSGLPSSVGTSNSGEMCATSEDMILSTVQNDVIHCEPSHTSLNSEVTLQSNMTLPQTNIIDIGNATPLETNIEDISQFQQDACYDLPIEDLISFDTDVHEMDMQHVLGNNGSSESQITKCWKETLTIFHGKGLRLPELTLREGYGEEERERDLDGLETEREEDLELDRDDDLETEGERQPRL﹡

**Promoter regions**

>BrHK1(Bra032761)

TTTTTGTGCATATAAACTCTCAGAAGGATTATCATCTAGTGGACATTAAGAAATGATCTTATGTATTAATAGTATAGATGTAACATTTAATTATTATCCAAAATATATTATTTAACATGTTGATTTTTATGTCCCCACATATTAATCCCATTTAATACTAGGCCTTAAATAGATTTGATTTTATTCGTGATATGCGATTAATTTTTAGATTCCTTAATTGTACAATGCAAATCACATTTAGATGCATATGACAAAACAAGTCGATTTGTGCTGAACTAAACTCTGCGGGAGCTTTCTTTGGTCTAATCTAAAAGCAGGTCTTATGTTTTGAGATATATTGCGAGAGTGTGGTTTGTTCCAAGTTACCCATATTTTCATTACTCACAGCTATCTCTGTCTCTTCCTATTGCTCTCAGTTTAGCAGATTCAGCTCCAGAAACACAACAAAGAACCCTAGTTGTCACATTATCTTATCAAAAGGAAAACTATTATTACGTCTCAGACTTGTACTCCTCTTTCACATTCTTGAGTTATTTTTTTTTTTGGTGTACGAAATGGAAGAGATGAAACAAGTTAGATAGGATTCAAATACTCTCATCTAGATGTTTTTAGCTTCGCTCTTCTGTTTTAGTGACGAGAATTGTAAATTGAAACCTAGAGATTTAGGACTATAACCAGTTTTAAAATTTGCGTTTTGGTGTGTTGTGCTGATTGTTCGTCTAAACTCTGGAACGAAATTTTTCAGTGAAGGAGAACAATCATATATAAGTTGTTGCAGCTTGGAGGATATAGTCAGAAGCTGTCGTGCATGTGTGTTCGAGTTTGTGATTTAGAGTCAAGAAGCTAAAGATATATAAACGGTTGTTGCATTCTCAGGTAAACAACTTATGTATCAAAGGATTTTCTGCATCTAGAATGAAAAGTTTCTAGAACTAACTTGCCCATAGCTTATGTAACTTAACACTATTTAACTATGAAATGTTAATTTACAGAAGAAGTG

>BrHK2(Bra006075)

GATTGCTTCTATCTTTGTGTTTTTATTTGCCTTTTGTTGCAGCAAAGTTGTTAGCCAGTATGGCTTTGTTATCATCTTTGTAAACAATTCCTCCTCTAATGGAGACTAGTTATCTATAAAATCTTGTTTGACCAAAAAAAAAAAAAAAGAAAGAAAAACAGAGTAACTAGAAAGAAGAACAGAGCCGTGGAAATGTGAGACAGATCGAAACGTGTTGATTTCTCTGTCATTCGAGTACTTGGATTCGGTCTCAGAAAGAAAACAAACGTGCACACACACAGAGAGAGAGAAAAAAGAGAGAGAGAGAGTCGCCATTAGAATACTCGAGTGAAAAAAAAAGGAGACAGAACAGAACAGAGGAATATAATATACATTGACCTCTCTGTCTTAAGGGGAAAGCACATGGCTCTCTCTCCTCTTCTCCGTAAAATCGCGTATTTTTTAATTTTATCCTATCTTTCATAATTCACATGCACCCTCTCTTCTCTTCATTGGTTAATTAATCTTCCCAATGCTCATCATTTGAAGTTTATTTACTCTACCCAGGAGACTCTCTTCAGATCTATTTACTGTTCATTCTTCTTCCTTCTTTCTCTGTTAATCTCCCAGTGATCGGAGCCGGTGAAGGTATGCTTTTTTTTTCTTTTCCCGGCTTCTCATTCAATTTGTGTTTCTTTTTCCGATCATAGCTCAAAACTCAAAAGGTTTCATCTTTATTAAAACCCAGTTCCTTAGTTTTTGATTATTCTACTTTTTTCTAACTTCTTTACGTTTATTACATGTTTGATAGGTGACTTTATGATCCAATATTTCAATTCGAATATTAGTACATTTTTCATAAATATCAAGATTCTGGTCGGAGATGGATTGTACTGACTCTAATGTCTGAAACGTTGATACAGGTTCTTGATCAGACCAAGATTCTGTCTAAATAAAAAAGTGGTGTTGAGTTAAGAGTGTTTTTGTTCTTTTTTTGATAAAAAGAAAAAACTCTGATCAA

>BrHK3(Bra009011)

AAAAAACAGAAGGACATTTAAGCGAGAAGTAGAATTCAGTGTCATTCTCAACTGGGATTGGGTCTCAGAAAGTAAACGAACGTGCAGACTGCAGAGAGAAAAGAGAAGAAAGAGTCTGGGCCATTAGAATAGTCCTCCCTGCCAATATAATATACATTGACCTCTCTGTACTCTTGAGGGTAGCACATGCTCCTCCTCTTTCTCCTTCCATTTTTTTTTTTCGTTTCTCAACTCTTCTCCGTAAAATAGCATTTGTTTTTGTTTTTAACTCCTATCTTTCACAATTCACATGCCATTTCTCTTTCTTCATTGGTTAATCTGTTCCCAATGCTCATTTGAAGTTTATTTACTCTCTTCTCCCAAAGACGACAGAGGACGACTCTCTTCAGATCTACTCACTGTTCATTACCCTTTCATCATCTGTTGATTTTCCGGTGACCGGAACCATATAAGGTAAGCTTCTGATCCAATCCCTTCCAATTGTTTCTTTCCCCCCTGCTTCTCATTTGTATCAAATTGTTTTTTTCTTTCTTCTTCTTTTTTTTTCTATCTTTCTGCTTTCCTCATTGACTCCCTCCAGTTCATGATTTCGCTGATATATTTATTTATGATCCAAATCTTCTCCAGACTGTTTTTTTATCATTTCTCAAAAAGTTTCATCCGGTTAACTTTGATTTTTAGATTTTTGTTTCGGTTTGTCAAGTTCGACTTGGTCTAGAATTGTTTCCATCTCCATGTAACCGTAATTTGAAATTTCCCGCGAATGTTTAATCCCATAACTGGTCCACACTACAACCTTTTTTTTTAAGTCTTTTTCAAAATTCGATTTAGTTACTTATTATTTACAGATCATGATTCTGAACTGAAGATGATCAATTGTCCTGACTTCTAATATCTGTGTCTGAACTGTGACTATCTTTATACACGTTTCTTGGTCAGATCAAGATTCTGGTTGATTTAGAGTGTTTTGTTTCTTGTATAAAGGAGGAACTCTGATTAA
[truncated: 83,778 more chars]
