# Supplementary material for: Genome-Wide Identification, Phylogeny, Duplication, and Expression Analyses of Two-Component System Genes in Chinese Cabbage (Brassica rapa ssp. pekinensis)
Source: DNA Res. 2014 Feb 27;21(4):379–96. doi: 10.1093/dnares/dsu004 (PMC4131832; doi:10.1093/dnares/dsu004)
Supplement: Supplementary Data [file supp_dsu004_dsu004supp2.doc]

>GmHKL1(Glyma10g33240)

MLKAVASGLVITSILICVSANDNGFPRCNCDDEASFWTIESILECQRVGDFLIAVAYFSIPIELLYFISCSNVPFKWVLIQFIAFIVLCGLTHLLNGWTYGPHTFQLMVALTVSKILTALVSCATAITLITLIPLLLKVKVREFMLKKKTWDLGREVDHIMRQKEAAMHVRMLTQEIRKSLDRHTILYTTLVELSKTLGLQNCAVWMPNVDKTEMNLTHELNGRNFNLTIPISDPDVVRIKGSDDVNILSSDSALAVGSRGVSGEAGPVAAIRMPMLRVCNFKGGTPELRQACYAILVLILPTGDNQEPRSWSNQELEIIKVVADQVAVALSHAAILEESQLMREKLEEQNRALQQAKRNALMASQARNAFQKVMSDGMRRPMHSILGLLSMIQDDNLKNEQKLIVDAMLRTSNVLSNLINDAMDNSTKDEGRFSLEIRSFGLHSMLKEAACLSKCMCVYKGFGFMVEVEKSLPDNVMGDERRVFQKTGSQGRSDKGWTTWRPSSSSGDVNIRFEIGINSSDSEVGSSISSGFGGRKYSSDRVGGRLSFSICKRVVQLMQGNIWLVPCNHGFPQSMTLLLRFQLRPSITIAISDPGEGSEHTDSNSMLRSLQVLLVDNDDVNRAVTQRLLQKLGCVVTSVASGFECLTVIGPAGSSIQVILLDLHMPDIDGFEVATRIRKFRSGNRPMIVALTASAEEDLWDRCMQVGINGVIRKPVLLHGIASELRRILMQGNIVL﹡

>GmHKL2(Glyma20g34420)

MLKAVASGLVITSILICVSANDNGFPRCNCDDEASLWTIESILECQRVGDFLIAVAYFSIPIELLYFISCSNVPFKWVLIQFIAFIVLCGLTHLLNGWTYGPHTFQLMVALTVSKILTALVSCATAITLITLIPLLLKVKVREFMLKKKTWDLGREVDHIMRQKEAAMHVRMLTQEIRKSLDRHTILYTTLVELSKTLGLQNCAVWMPNVDKTEMNLTHELNGRNFNLTIRITDPDVVRIKGSDGVNILSSDSALAVGSRGVSGEAGPVAAIRMPMLRVCNFKGGTPELRQACYAILVLILPSGDNQEPRSWSNQELEIIKVVADQVAVALSHAAILEESQLMREKLEEQNRALQQAKRNALMASQARNAFQKVMSDGMRRPMHSILGLLSMIQDDKLKSEQKLIVDAMLRTSNVLSNLINDAMDNSTKDEGRFPLEIRSFGLHSMLKEAACLSKCMCVYKGFGFMVEVEKCLPDNVMGDERRVFQVILHMVGNLLEHNHGGGILVYRVFAETGSQGRSDKGWTTWRPSSSSGDVNIRFEIGINSSDSEVGSSISSGFGGRKYSSDRVGGRLSFSICKRVVQLMQGNIWLVPCNHGFPQSMTLLLRFQLRPSISIAISDPGEGSERTDSNSMLRNLQVLLVENDDVNRAVTQRLLQKLGCVVTPVASGFECLTVIGPAGCSIQVILLDLHMPDLDGFEVATRIRKFRSGNQPMIVALTASAEEDLWERCMQVGINGVIRKPVLLHGIASELRRILMQGNNVL﹡

>GmHKL3(Glyma20g21780)

MLKAVSFELLLICLLLLRTCVCATTDNGFPRCNCDDESSLWTIETILECQRIGDFLIAVAYFSIPIELLYFVSCSNFPFKWVLFQFIAFIVLCGMTHLLNGWTYGPHTFQLMVALTVFKILTALVSCATTITLLTLIPMLLKVKVRELMLKKKTWDLGREVGIIMKQKEAAMHVRMLTQEIRKSLDRHKILYTTLVELSKTLGLQNCAVWMPNVEKTEMNLTHELNGRNVNCSIPITNPDVVRIKGSDEVNIIDSDSILATASSGVYGGAGPVAAIRMPMLQVCNFKGGTPELRQTCYAILVLTLPSAEPRSWGAQELEIIKVVADQVAVALSHASILEESQLMREKLEEQNRALQMEKMNTMMASQARASFQKVTSNGMRRPMHSILGLLSMMQDDNLKSEQKLIVNSMLRTSTVLSNLINDAMDYSTRDDGRFPLEMKPFGLHAMVKEAACLAKCMCVYRVIGFVVDVDKFEIGINNGDPELESSVPSGQLAGTDRTSDKVEERLSFSICKRIIQLMQGNIWLVPNAQGFPQVMALFLRFQLWRSIAVSNSEPGENSETSNSNSFFRGLQVLLADNDDVNRAVTQKLLQKLGCVVTSVSSGFECLNVIGPAGSSFQVILLDLHMPELDGFEVATRIPKFRSRNWPVIVALTASTDDLWERCMQIGMNGVIRKPVLLHGIASELRRIILQGNSVM﹡

>GmHKL4(Glyma03g41220)

MERGLLLLLFLLLVMVLSVCGNDVEYSQCNCDEEGLWSIHNVLVCQKVSDFFIAIAYFSIPLELLYFVSCSNVPFKLVFLQFIAFIVLCGLTHLLNAYTYYGPHSFQLFLSLTVAKFLTALVSCATAISFPTLIPLLLKIKVRELFLRQNVLELGQEVGMMKKQKEASWHVRMLTCEIRKSLDKHTILYITLVELSKALDLHNCAVWMPDEDRREMHLTHELKPNSTRIFHNSIPISDPDVLDIKKSQGVWILRPDSALGAASSGGGGSGDSGAVAAIRMPILHVSNFKGGTPEFVETSYGVLVLVLPNSDSRAWTSHEMEIVKVVADQVAVALSHASVLEESQLMSQKLAEQNRALQQAQKNAMMARKARSSFEKVMSHGMRRPMHSILGLLSMFQEDNIRPEQKIVIDSILKVSNALSRLINDVMEIAANDNGSFQLEMKPFHLHSMMREASCTAKCLCIYKGFGLEVDVDKSLPDLVIGDEARTFQVILHMIGYLLNIYDKGNLIFQVYLKSDSGDRDDRSFGLWRSSMQNEYVHIKFNFQINGISSQSDESVSTRNYTGRRHYNNEPKEGLSFSMCKTLVQMMQGNIWISTNSLGLAQGMTLLLKFQIGSSHGRFTLAPTDFSNSQFRGLKVVLADDDDVNRTVTKKLLEKLGCQVTAVSSGFECLGAISGSGNSFKIILLDLHMPEMDGFEVARRIRKFQSHNWPLIIAFTASAEEHIKERCLQVGMNGLIRKPILLREIADELGTVLQRAGEKL﹡

>GmHKL5(Glyma19g43840)

MERGLLLLLFLLLVMVLSVYANDVEYSQCNCDEEGLWSIHSVLVCQKVSDFFIAIAYFSIPLELLYFVSCSNVPFKLVFLQFIAFIVLCGLTHLLNAYTYYGPHSFQLFLSLTVAKFLTALVSCATAISFPTLIPLLLKIKVRELFLRQNVLELGQEVGMMKKQKEASWHVRMLTCEIRKSLDKHTILYTTLVELSKALDLHNCAVWMPDEDRREMHLTHELKPSSARSFHNSIAISDPDVLDIKKSQGVWILRPDSALGAASSGGGSGDSGAVAAIRLPILHVSNFKGGTPELVETSYGVLVLVLPNSNSRAWTSHEMEIVEVVADQVAVALSHASVLEESQLMSQKLAEQNRALQQAQKNAMMARKARSSFEKVMSHGMRRPMHSILGLLSMFQEDNIRPEQKIVIDSILKVSNALSRLINDVMEIAENDNGSFQLEMKPFHLHSMMREASCTAKCLCIYKGFGLEVDVDKSLPDLVIGDEARTFQVILHMIGYLLNIYDKGTLTFQVYLESDSGDKDDRSFGIWRSSIQNEYVHIKFNFQINGISFHSDESVSTRNYTGRNHCNNELKEGLSFSMCKTLVQMMQGNIWISTNSLGLAQGMTLLLKFQIGSSHGRFILAPKEFSNSQFRGLKVVLADDDDVNRTVTKKLLEKLGCQVTAVSSGFECLGAISASGNSFKIIMLDLHMPEMDGFEVARRIRKFQSHNFFLCSLLLLQVQKNTSRRDVYRWE﹡

>GmHKL6(Glyma10g31040)

MEITPPLLHHLWFLLLLLLLCYLVLCASATDVEFDNCNCDDGEGIWSIHSILVGQKVSDFFIAVAYFSIPIELLYFVSRSNVPFKLLFLQFIAFIVLCGMTHLLNAYSYHGPPSFQLLLSLTVAKFLTALVSCATALTLPPLIPLLLKVKVRELFLRQNVMELGQEVGMMKKQKEASWHVRMLTREIRKSLDKHNILYTTLVELSKALDLHNCAVWMPNEDRREMHLTHELKTNSAKNFQNSIPVNDPDVLEIRKTKGVKILGPESALGAASSGGSVELGAVAAIRMPLLHVSNFKGGTPELVETCYAILVLVLPSSSTRVWTYHEMEIVEVVADQVAVALSHASVLEESQLMRQKLEERNRALQQAKKNAMMASQARKSFQKVMSHGMRRPMHSVLGMLSLFQEDNLRSEQKIIGDTMLKVGHVLSSLINDVMEISENEKGGFRLEMKPFLLHSMVREAASIAKCLCVYEGFGFEIDVQKSLPETVMGDEARTFQVILHMIGYLLNMNDKGTLNFRVFLESDGGDRDDKNIGIWRSSNQNEYVHIKFDFQITESSQSDEAISTIHYTGRRQYYNNEPKGGLSFSMCKKLVQMMQGNIWISPNSLGLVHGMTLLLKFQIGPSLEKSIFAPKDYSSSQFRGLKVVLAEDDGVNRTVTKKLLEKLGCQVIAVSSGFECLSAVSGAGNSFRIILLDLHMPEMDGFELAKRIRKFHSRSWPLIIALITSAEEHVREKCLLAGMNGLIQKPIVLHQIADELRTVLQRAGEKL﹡

>GmHKL7(Glyma20g36440)

MEITPPPLLLLFLLLLFFLCYLVLCASATDVDFDNCNCDDGEGIWSIHSILVGQKVSDFFIAVAYFSIPIELLYFVSRSNVPFKLLFLQFIAFIVLCGMTHLLNAYSYHGPPSFQLLLSLTVAKFLTALVSCATALTLPPLIPLLLKIKVRELFLRQNVMELGQEVGMMKKQKEASWHVRMLTREIRKSLDKHNILYTTLVELSKALDLHNCAVWMPNEDRREMHLTHELKTNSAKNFQNSIPVNDPDVLEIRKTKGVKILRPDSAIGAASSGGSAELGAVAAIRMPLLHVSNFKGGTPQLVETCYAILVLVLPSSSTRVWTYHEMEIVEVVADQVAVALSHASVLEESQQMRQKLEERNRALQQAKKNAMMASQARKSFQEVMSHGMRRPMHSILGMLSLFQEDNLRSEQKIIGDTMLKVGHVLSSLINDVMEISENEKGGFRLEMKPFLLHSMMREAASIAKCLCVYEGFGFEIDVQKSLPETVMGDEARTFQVILHMIGYLLNMNDKGTLNFRVFLESDGGDRDDKNIGIWRSSSQNEYVHIKFDFQITESSQSDKAISTIHYSSRRQYYNNEPKEGLSFSMCKKLVQMMQGNIWISPNSLGLVQGMTLLLKFQIGPSLGKSIFAPKDYSSSQFRGLKVLLAEDDGVNRTVTKKLLEKLGCQVIAVSSGFECLSAISGAGNSFRIILLDLHMPEMEGFEVAKRIRKFHSRSWPLIIALIASAEEHVREKCLLAGMNGLIQKPIVLHQIANELRTVLQRAGEKL﹡

>GmHKL8(Glyma10g28170)

MSTSRPSQSSSNSRRSRHSARMAQATVDAKIHATFEESGSSFDYSSSVRVSGTADGVNQPRSDKVTTAYLNHMQRGKMIQPFGCLLAIDEKTCKVIAYSENAPEMLTMVSHAVPSVGDHPALGIGTDIKTLFTAPSVSGLQKALGCADVSLLNPILVHCKTSGKPFYAIVHRVTGSLIVDFEPVKPYEVPMTAAGALQSYKLAAKAITRLQSLPSGNMERLCDTMVQEVFELTGYDRVMAYKFHEDDHGEVIAEITKPGLEPYLGLHYPATDIPQASRFLFRKNKVRMIVDCHAKHVRVLQDEKLQFDLILCGSTLRAPHSCHAQYMANMDSIASLVLAVVVNDNEEDGDTDAVQPQKRERLWGLVVCHNTTPRFVPFPLRYACEFLAQVFAVHVHKEIELEYQIIEKNILRTQALLCMLMRDAPLGIVSESPNIMDLVKCDGAALIYRNKVWRLGVTPSEPQIREIALWLSEYHMDSTGFSTDSLFDAGFPSALSLGDVVCGMASVRVTAKDMVFWFRSHTAAEIRWGGAKHEAGEKDDSRRMHPRSSFKAFLEVVKARSLPWKEYEMDAIHSLQIILRNAFKEDTESLDLNAKAINTRLSDLKIEGINDLKIERMQELEAVTSEIVRLIDTATVPILAVDVDGLVNGWNIKIAELTGLPIGEATGKHLLTLVEDSSTDRVKKMLNLALLGEEEKNVQFEIKTHGSKMDSGPISLVVNACASRDLRDNVVGVCFVAHDITAQKNVMDKFIRIEGDYKAIVQNRNPLIPPIFGTDEFGWCCEWNPAMMKLTGWKREEVMDKMLLGEIFGTQMAACRLKNQEAFVNLGVVLNKAMTGSETEKVPFGFFARNGKYVECLLSVSKKLDVEGLVTGVFCFLQLASPELQQALHIQRLSEQTASKRLNALSYMKRQIRNPLCGIVFSRKMLEGTDLGTEQKQLLRTSAQCQQQLSKILDDSDLDTIIDGYLDLEMAEFTLHEVLVTSLSQVMTKSNGKSIRIVNDVAGHIMMETLYGDSLRLQQVLADFLLISINFTPNGGQVVVAGSLTKEQLGKSVHLVKLELSITHGGSGVPEVLLNQMFGNNGLESEEGISLLISRKLLKLMNGDVRYLREAGKSAFILSAELAAAHNLKA﹡

>GmHKL9(Glyma20g22160)

MSTSRPSQSSSNSGRSRRSARAMALATVDAKLHATFEESGSSFDYSSSVRISGTADGVNQPRHDKVTTAYLHHMQKGKMIQPFGCLLALDEKTCKVIAYSENAPEMLTMVSHAVPSVGDHPALGIGTDIKTLFTAPSASALQKALGFAEVLLLNPVLIHCKTSGKPFYAIIHRVTGSMIIDFEPVKPYEVPMTAAGALQSYKLAAKAITRLQSLPSGSMERLCDTMVQEVFELTGYDRVMAYKFHEDDHGEVIAEITKPGLEPYLGLHYPATDIPQASRFLFMKNKVRMIVDCHAKHVRVLQDEKLPFDLTLCGSTLRAPHSCHAQYMANMDSIASLVMAVVVNDNEEDGDTDAIQPQKRKRLWGLVVCHNTTPRFVPFPLRYACEFLAQVFAIHVNKEIELEYQIIEKNILRTQTLLCDLVMRDAPLGIVSESPNIMDLVKCDGAALIYKNKVWRLGVTPSESQIREIAFWLSEYHMDSTGFSTDSLSDAGFPSALSLGDVVCGMAAVRVTAKDVVFWFRSHTAAEIRWGGAKHEAGEKDDGRRMHPRSSFKVFLDVVKARSLPWKEYEIDAMHSLQLILRNAFKDTESMDLNTKAINTRLSDLKIEGMQELEAVTSEIVRLIETATVPILAVDVDGLVNGWNIKIAELTGLPVGEAMGKHLLTLVEDSSTDRVKKMLNLALLGEEEKNVQFEIKTHGSKMDSGPISLVVNACASRDLRDNVVGVCFVAHDITAQKNVMDKFTRIEGDYKAIVQNRNPLIPPIFGTDEFGWCCEWNPAMTKLTGWKREEVMDKMLLGELFGTHMAACRLKNQEAFVNLGVVLNKAMTGLETEKVPFGFFARNGKYVECLLSVSKKLDVEGLVTGVFCFLQLASPELQQALHIQRLSEQTALKRLNALSYMKRQIRNPLCGIIFSRKMLEGTALGTEQKQLLRTSAQCQQQLSKILDDSDLDSIIDGYLDLEMAEFTLHEVLVTSLSQVMTKSNGKSIRIVNDVAEQIVMETLYGDSLRLQQVLADFLLISINFTPNGGQVVVAGTLTKEQLGKSVHLVKLELSITHGGSGVPEALLNQMFGNNGLESEEGISLLISRKLLKLMNGDVRYLREAGKSAFILSAELAAAHNLKG﹡

>GmHKL10(Glyma19g41210)

MSSSRPSQSSSNNSGRSRTSRLSARRMAQTTLDAKLHATFEESGSSFDYSSSVRMSPAGTVSGDHQPRSDRATSSYLHQTQKIKLIQPFGCLLALDEKTCKVIAYSENAPEMLTMVSHAVPSVGDHPALGIGTDIRTIFTAPSSAAIQKALRFGDVSLHNPILVHCKTSGKPFYAIIHRVTGSVIIDFEPVKPHEVPMTASGALQSYKLAAKAITRLESLTTGNMETLCNTMVREVFELTGYDRVMAYKFHEDDHGEVIAEVKRPGLEPYLGLHYPATDIPQATRFLFMKNKVRMIVDCCAKHVNVLQDKKIPFDLTLCGSTLRAAHSCHLQYMENMNSSASLVMAVVVNDNDEDGDSSDAVQPQKSKRLWGLVVCHHTTPRFVPFPLRYACQFLAQVFAVHVSKELEIEYQIIEKNILQTQTLLCDMLVQGEPLGIVSQSPNIMDLVKCDGAALLYKNKVWRLGVTPSESQIKEIALWLFECHEDSTGFCTDSLSDAGFPGAAALGDIACGMAAARIASKDILFWFRSHTASEIRWGGAKHEPGERDDGRRVHPRSSFKAFLEVVKTRSLPWKTYETDAIHSLQLILRDAFKETQSMEISTYAIDTRLGDLKIEGMQELDAVTSEVVRLIETATVPILAVDVNGMINGWNTKIAELTGLPVDEAIGKHLLTLVEDFSVDRVKKMLDMALQGEEERNVQFEIQTHHMKIDSGPISLVVNACASRDLQDNVVGVCFLAQDITAQKTMMDKFTRIEGDYKAIVQNPNPLIPPIFGTDEFGWCCEWNSAMAKLTGWKREEVMDKMLLGEVFGTQIACCRLRNHEAVVNFSIVLNTAMAGLETEKVPFGFFARDGKHVECILSMTKKLDAEGVVTGVFCFLQLASAELQQALHIQRISEQTSLKRLKDLTYLKRQIQNPLYGIMFSRKLLEGTELGAEQKQFLQTGIRCQRQISKILDDSDLDSIIDGYMDLEMVEFTLHEVLVASLSQVMTKSNAKGIRVVNDVEEKITTETLYGDSIRLQQVLADFLLISINFTPTGGQVVVAATLTQQQLGKLVHLANLEFSITHDSFGVPETLLNQMFGRDGHESEEGISMLISRKLLKLMNGDVRYLREAGKSSFILSVELAAAHKSNT﹡

>GmHKL11(Glyma03g38620)

MIILVYVLGMMFLSFPGQLRSSRPSARRISQTSLDAKPHATFEESGSSFDYSNSVKMSPAGTGGGTVSGEHEPKSDRAATTAYLHQMQKGKLIQPFGCLLVLDEKTYKVIAYSENAPEMLTMASHAVPSVDDHPALDIGTYIRTIFTAPSIASIHKVLGFGDLSLHNTILVHCKTFGNPFYAIIHLVTGSTIIDFESVQPPEVPMTASGSLQSYYKLAAKATTRLQSLATVNMETLCNTMVQEVFELTGYDRVMAYKFHDDDHGEVIAEVKRPGLEPYLGLHYPATDIPHATRFSLWRTRCVIQDKKIPFDLALYGSTLRAAHSCHLQFMVNMNSSASLVLAVVINDNDEDGNSSDDAAVQQPHKSSTSLWGLVVCHHTTPKFVPQGRICHPRVGKELEIEYQIVEKNILRTQTHLFDVLTRDEPLAIVSQSPNMMDLVKCDGATLLYKNKVWRLGVTPSESQIREIALWLSQCHRDSTGFFTDSLSDAGFPGAAALGDIACGMTSARITSKDIVFWFWSHTAAEIRCDGAKHEPGERDDVVKNRSLLWKVYETDAIHSLHLILRDAFKETESMKIATYAPNSRLGCLNIEETQGLEAVTNEMVRLIETATVPVLAVDVNGMVNGWNTKIAELTGLPSDEAMGKHFLTLVEDFSVDRVKKMLHMALQGEEEEERNVQFEINTYDFKIDSGPASLVVNACASRDLQDNIVGVCFVAQGITAQKTMMEKFPRIEGDYKAIVQNPNPSIPPLFSTDEFGWCCEWNSAMAKLTGWKREEVMDKMLLGEIFGTQIAGCRLRNHEAVVNFSIVLNTAMAGLETEKVPIGFFTRDGKHVESSPELQQALHIQLLSEQTAMKRLKDLNYLKRQIRNPLYGIMFSRKLLEGTELGAEQKQFLQMSTQCQHQLSKILDDSDLDSIIDGCHLCGILSIYQQEFLLNHTIVNITHDGFGVPETLLNQMFGRDGHESEEGISMLISRKLMKGDVRYIREAGKIIFHLIC﹡

>GmHKL12(Glyma09g03990)

MASASGAANSSVPPPQIHTSRTKLSHHSSNNNNNIDSMSKAIAQYTEDARLHAVFEQSGESGRSFNYSESIRIASESVPEQQITAYLVKIQRGGFIQPFGSMIAVDEPSFRILGYSDNARDMLGITPQSVPSLDDKNDAAFALGTDVRALFTHSSALLLEKAFSAREISLMNPIWIHSRTSGKPFYGILHRIDVGIVIDLEPARTEDPALSIAGAVQSQKLAVRAISQLQSLPGGDVKLLCDTVVESVRELTGYDRVMVYKFHEDEHGEVVSESKRPDLEPYIGLHYPATDIPQASRFLFKQNRVRMIVDCHASAVRVVQDEALVQPLCLVGSTLRAPHGCHAQYMANMGSIASLVMAVIINGNDEEGVGGRSSMRLWGLVVCHHTSARCIPFPLRYACEFLMQAFGLQLNMELQLAAQSLEKRVLRTQTLLCDMLLRDSPTGIVTQSPSIMDLVKCDGAALYFQGNYYPLGVTPTEAQIRDIIEWLLAFHGDSTGLSTDSLGDAGYPGAASLGDAVCGMAVAYITEKDFLFWFRSHTAKEIKWGGAKHHPEDKDDGQRMHPRSSFKAFLEVVKSRSLPWENAEMDAIHSLQLILRDSFKDAEHRNSKAVVDPHVSEQELQGVDELSSVAREMVRLIETATAPIFAVDVDGHVNGWNAKVSELTGLPVEEAMGKSLVHDLVFKESEETMNKLLSRALKGEEDKNVEIKMRTFGPEHQNKAVFLVVNACSSKDFTNNVVGVCFVGQDVTGQKIVMDKFINIQGDYKAIVHSPNPLIPPIFASDDNTCCLEWNTAMEKLTGWGRVDVIGKMLVGEVFGSCCQLKGSDSITKFMIVLHNALGGQDTDKFPFSFLDRHGKYVQTFLTANKRVNMEGQIIGAFCFLQIMSPELQQALKAQRQQEKNSFGRMKELAYICQGVKNPLSGIRFTNSLLEATSLTNEQKQFLETSVACEKQMLKIIRDVDLESIEDGSLELEKGEFLLGNVINAVVSQVMLLLRERNLQLIRDIPEEIKTLAVYGDQLRIQQVLSDFLLNIVRYAPSPDGWVEIHVRPRIKQISDGLTLLHAEFRMVCPGEGLPPELIQDMFNNSRWGTQEGLGLSMSRKILKLMNGEVQYIREAERCYFYVLLELPVTRRSSKKC﹡

>GmHKL13(Glyma15g14980)

MASASGAENSSVPPSPLPPPPPPQIHTSRTKLSHHHHNNNNNNNNNIDSTSKAIAQYTEDARLHAVFEQSGESGRSFDYSQSIRVTSESVPEQQITAYLLKIQRGGFIQPFGSMIAVDEPSFRILAYSDNARDMLGITPQSVPSLDDKNDAAFALGTDIRTLFTHSSAVLLEKAFSAREISLMNPIWIHSRTSGKPFYGILHRIDVGIVIDLEPARTEDPALSIAGAVQSQKLAVRAISQLQSLPGGDVKLLCDTVVESVRELTGYDRVMVYRFHEDEHGEVVAETKRPDLEPYIGLHYPATDIPQASRFLFKQNRVRMIVDCHASAVRVVQDEALVQPLCLVGSTLRAPHGCHAQYMANMGSTASLVMAVIINGNDEEGVGGRTSMRLWGLVVCHHTSARCIPFPLRYACEFLMQAFGLQLNMELQLAAQSLEKRVLRTQTLLCDMLLRDSPTGIVTQSPSIMDLVKCDGAALYYQGNYYPLGVTPTEAQIRDIIEWLLAFHRDSTGLSTDSLADAGYPGAASLGDAVCGMAVAYITEKDFLFWFRSHTAKEIKWGGAKHHPEDKDDGQRMHPRSSFKAFLEVVKSRSLPWENAEMDAIHSLQLILRDSFKDAEHSNSKAVLDPRMSELELQGVDELSSVAREMVRLIETATAPIFAVDVDGRINGWNAKVSELTGLPVEEAMGKSLVRDLVFKESEETVDKLLSRALKGEEDKNVEIKMRTFGPEHQNKAVFVVVNACSSKDYTNNVVGVCFVGQDVTGQKIVMDKFINIQGDYKAIVHNPNPLIPPIFASDDNTCCLEWNTAMEKLTGWSRADVIGKMLVGEVFGSCCQLKGSDSITKFMIVLHNALGGHDTDRFPFSFLDRYGKHVQAFLTANKRVNMDGQIIGAFCFLQIVSPELQQALKAQRQQEKNSFARMKELAYICQGVKNPLSGIRFTNSLLEATCLSNEQKQFLETSAACEKQMLKIIHDVDIESIEDGSLELEKGEFLLGNVINAVVSQVMLLLRERNLQLIRDIPEEIKTLAVYGDQLRIQQVLSDFLLNIVRYAPSPDGWVEIHVHPRIKQISDGLTLLHAEFRMVCPGEGLPPELIQNMFNNSGWGTQEGLGLSMSRKILKLMNGEVQYIREAQRCYFYVLLELPVTRRSSKKC﹡

>GmHKL14(Glyma09g11600)

MSFGSRGKLKDTSLSTSAESNMNSKRDKILAQYSADAEILAEFEQSGVSGKSFDYSRMVLDPPRLVSEQKMTAYLSKIQRGGLIQPFGCMLAIEESTFRIIGFSDNCFQLLGLERQIDSKQFMGLIGVDATTLFTPPSGASLAKAAASREISLLNPIWVYARTTQKPFYAILHRIDVGVVIDLEPARMSDPALSLAGAVQSQKLAVRAISRLQSLPGEDIGLLCDTVVEEVQKLTGYDRVMVYKFHEDDHGEVVSEIRRSDLEPYLGLHYPATDIPQASRFLFKQNRVRMICDCHAKPVKVIQSEELRQPLCLVNSTLRLPHGCHTQYMANMGSIASLVMAIVVNGKHATRLWGLLVCHHTSPRYVSFPVRYACEFLMQAFGLQLYMEIQLASQMAEKRILKTQTLLCDMLLRDAPLGIVNQSPSIMDLVKCDGAALYYEGNCWLLGTTPTEAQVKDIAEWLLSNHGDSTGLTTDSLADAGYPGAASLGDAVCGMATARINSKHFLFWFRSHTAKEVKWGGAKHHPEDKDDGGKMNPRSSFKAFLEVVKSKSLPWEVPEINAIHSLQLIIRDSFQDTENTGPKTLTYVQKSDTATGGMDELSSVALEMVRLIETATVPIFGVDLGGVINGWNTKIAELTGLQASEAMGKSLVNEIIHADSCDTFKSTLSRALQGQEDKNVELKIKHFGLDQQQEVAYLVVNACTSRDHTDAIVGVCFVGQDITCEKVVQDKFIQLEGDYKAIIQSLSPLIPPIFSSDENACCSEWNAAMERLTGWKRDEVIGKLLPGEIFGSFCRLKGQDTLTNFMILLYRGISGQDSEKIPFGFFDRNGEFIETYITANKRIDTGGNMLGCFCFLQIVMPDLNQPSEEHKPRGRESISESKELAYILQEMKKPLNGIRFTRKLLENTAVSENQKQFLDTSDACERQILAIIEDTNLGSINEGTLQLNMEEFVLGNILDAIVSQVMMLIREKNLQLFHEIPDEIKMLSLYGDQIRLQVVLSDFLLNVVSHTASPNGWVEIKISPGLTLQDGNEFIHLKFSMAHSGQGIPSNVLHDMFEGGNQWTTQEGLGLYMSRKILSRISGHVQYVREQNKCYFLIDLEIRKRKERKRNLHAETSMLS﹡

>GmHKL15(Glyma15g23400)

MICDCHAKPVKVIQSEELRQPLCLVNSTLRLPHGCHTQYMANMGSIASLVMAIIVNGKDATRLWGLLVCHHTSPRSVSFLVRYACEFLMQTFGLQLYMEIQLASQMAEKRILKTQTLLCDMLLRDAPFGIVNQSPSIMDLVKCDGAALYYEGNCWLLGTTPTEAQVKDIAEWLLSNHGDSTGLTTDSLADAGYPGAASLGDAVCGMATARINSKHFLFWFRSHTAKEVKWGGAKHHPEDKDDGGKMNPRSSFKAFLEVVKSKSLPWEVLEINAIHSLQLIIRDSFQDTENTGPKTLSYVQKSDTAAGGMDELSSVALQMVRLIETATVPIFGVDLGGVINGWNTKIAELTGLQASEAMGKSLVNEIIHADSGDTFKNTLSRALQGQEDKNVELKIKHFGLDQQQEVACLMVNACISRDYTDAIVGVCFVGEDITYEKVVQDKFIKLEGDYKAIIQSLSPLIPPIFSSDENVCCSEWNAAMERLTGWKRDEVIGKLLPGEIFGSFCRLKGQDTLTNFMILLYRGISRQDSEKLPFGFFHRNGEFIETYITANKKIDAGGNMLGCFCFLQIVMPDLNQPSEEHNPRGRESISESEEAYILQEMKKPLNGIRFTRKLLENTTVSENQKQFLDTSDACERQIMAIIEDTHLGSINEDTLQLNVEEFVLGNILDAIVSQVMMLIREKNLQLFHEIPDEIKMLSLYGDQIRLQVVLSDFLLNVVSHTASPNGWVEIKVSPTLKIIQDGDEFIHLQFRIAHSGQGIPSNVIHEMVEGGNQWTTQEGLGLYMSRKILRRMSGHVRYQRGQDMCYFLIDLEIRTRKERQRNLHAKTSMLS﹡
