# Supplementary figures and images for: Genome-Wide Identification, Phylogeny, Duplication, and Expression Analyses of Two-Component System Genes in Chinese Cabbage (Brassica rapa ssp. pekinensis)
Source: DNA Res. 2014 Feb 27;21(4):379–96. doi: 10.1093/dnares/dsu004 (PMC4131832; doi:10.1093/dnares/dsu004)

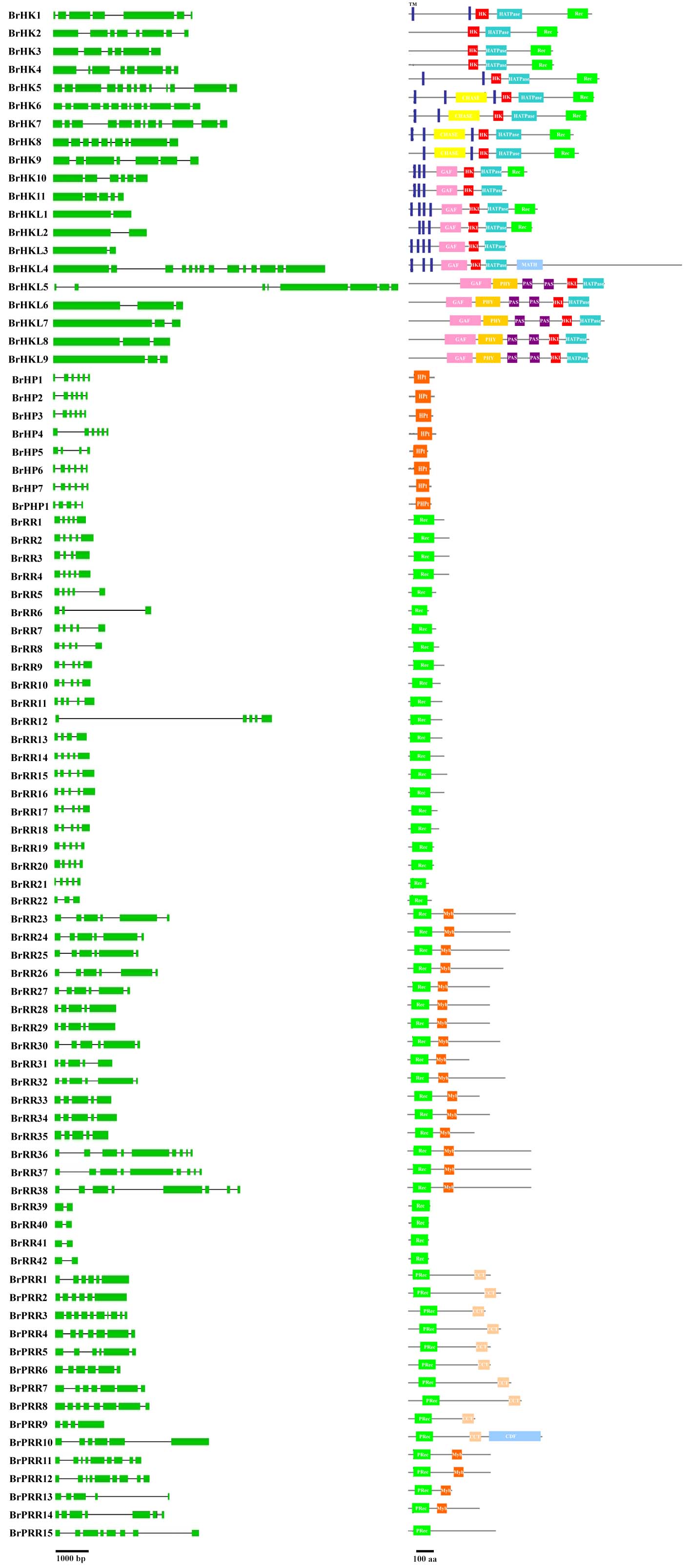

Supplement: Supplementary Data [file supp_dsu004_dsu004supp_fig1.jpg]

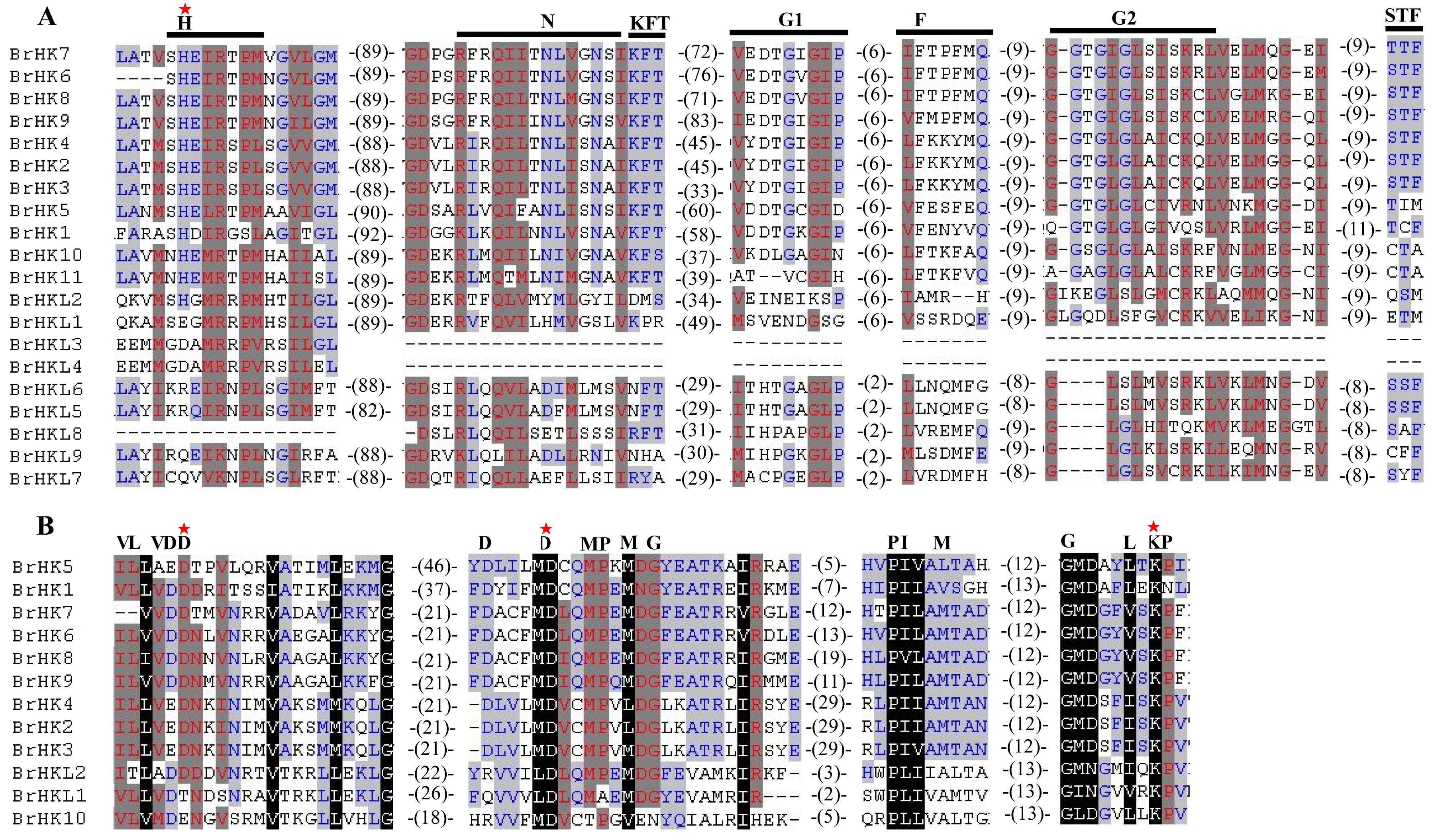

Supplement: Supplementary Data [file supp_dsu004_dsu004supp_fig2.jpg]

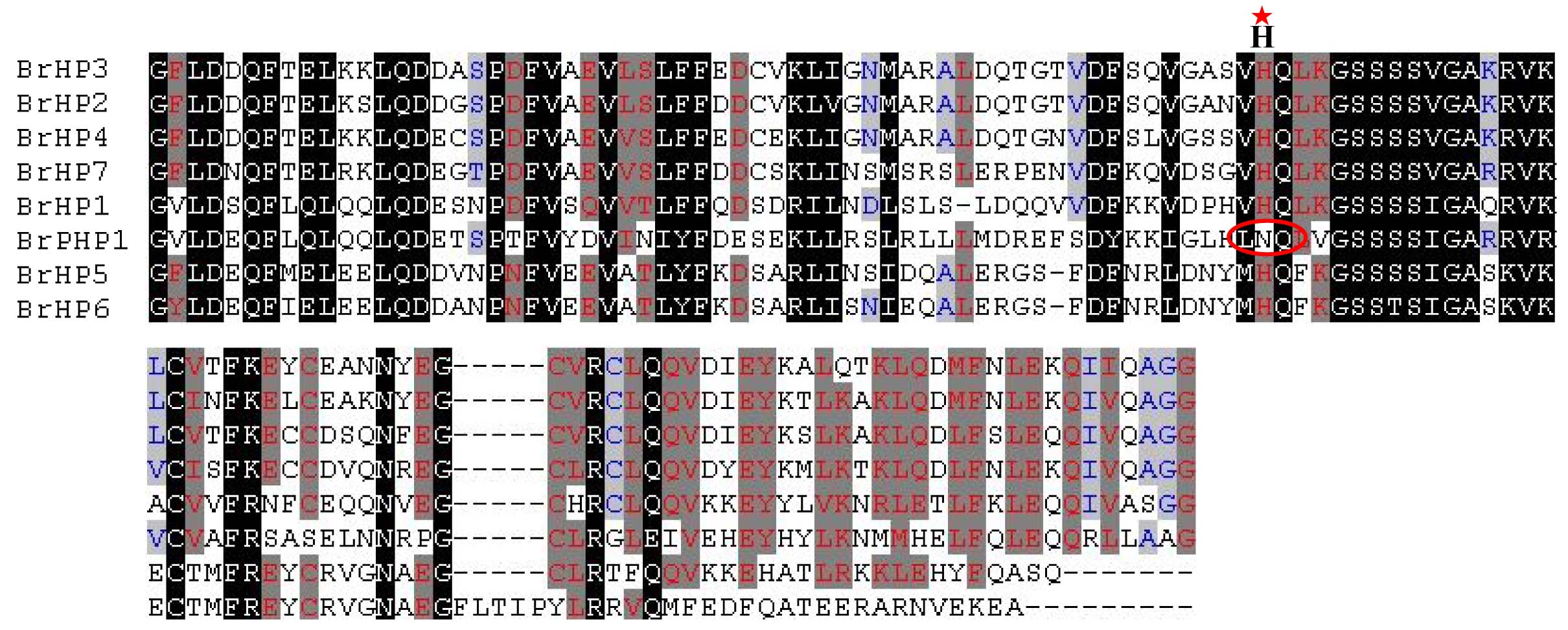

Supplement: Supplementary Data [file supp_dsu004_dsu004supp_fig3.jpg]

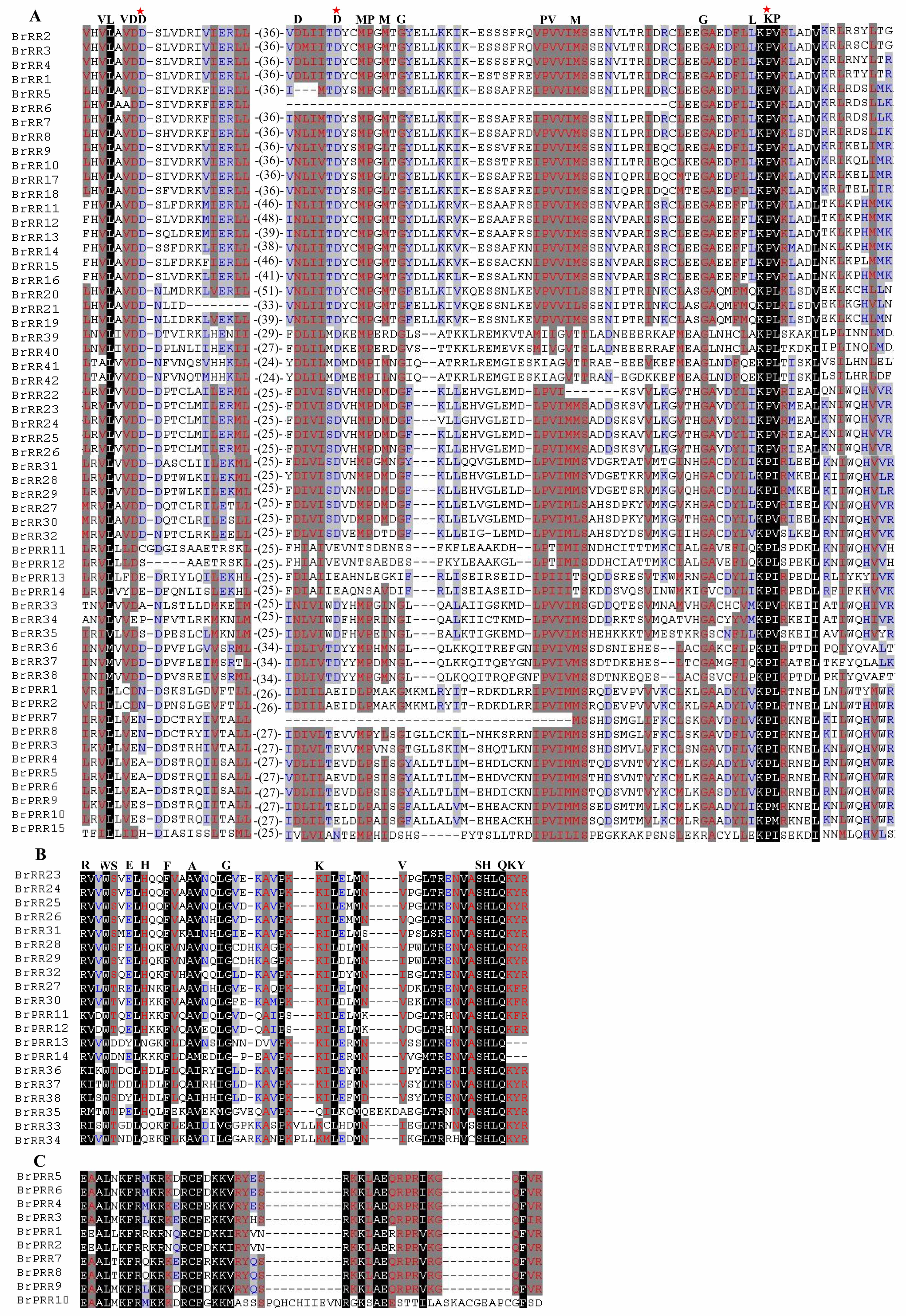

Supplement: Supplementary Data [file supp_dsu004_dsu004supp_fig4.jpg]

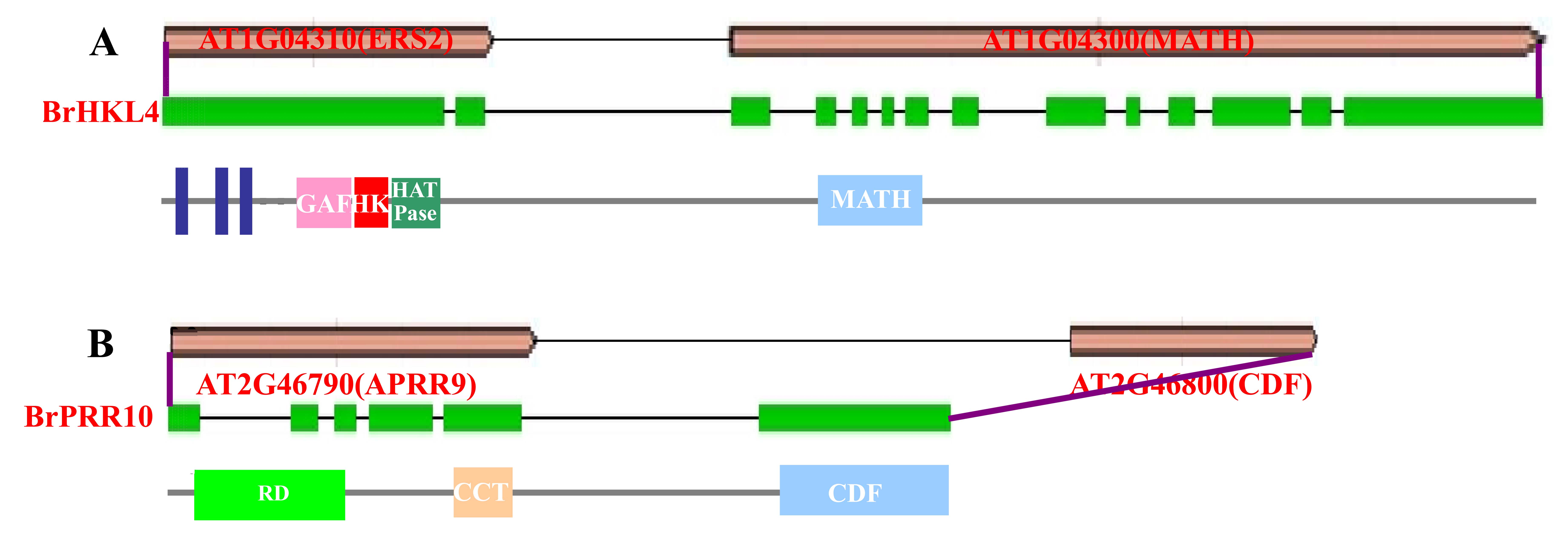

Supplement: Supplementary Data [file supp_dsu004_dsu004supp_fig5.jpg]

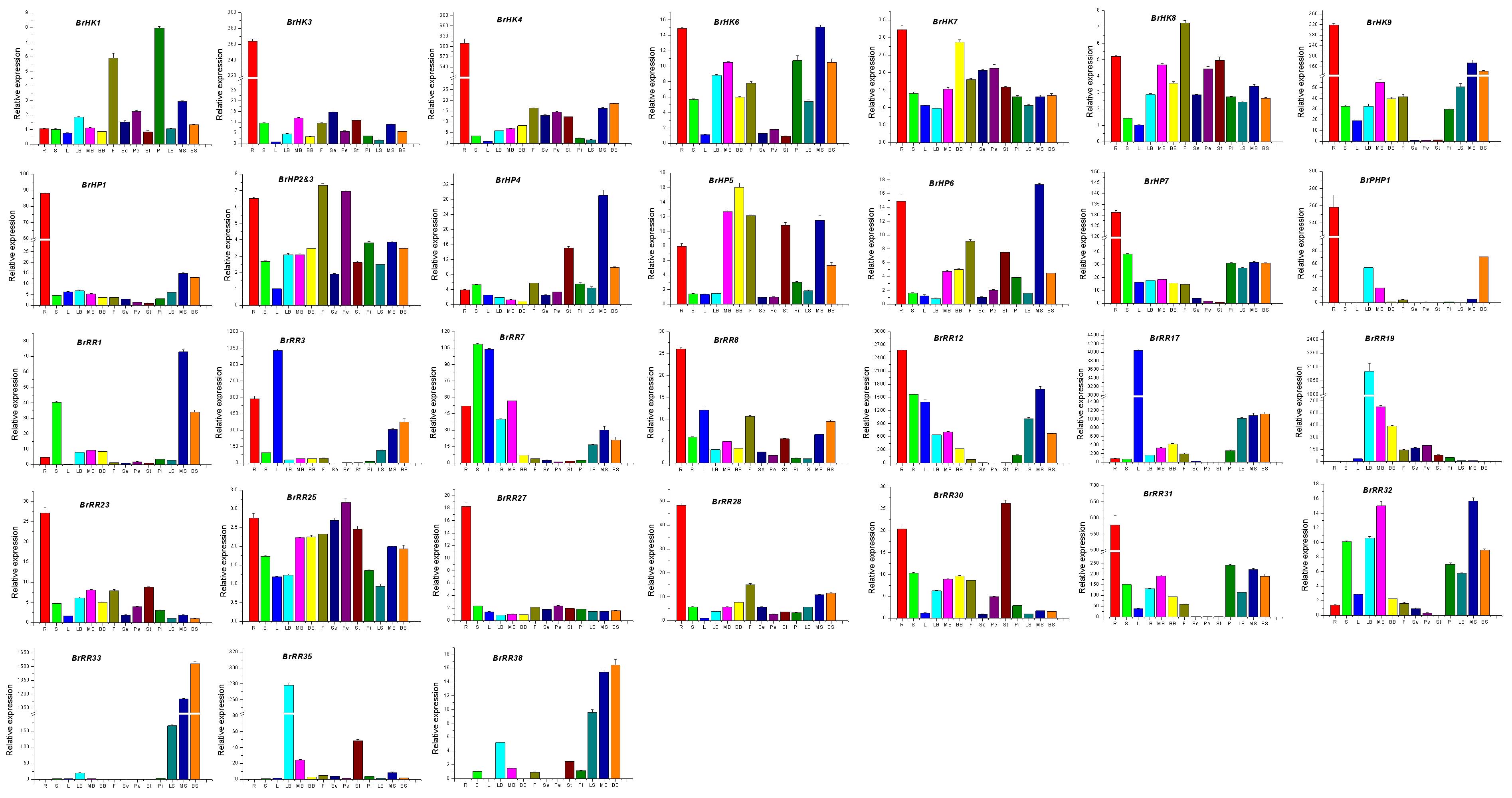

Supplement: Supplementary Data [file supp_dsu004_dsu004supp_fig6.jpg]

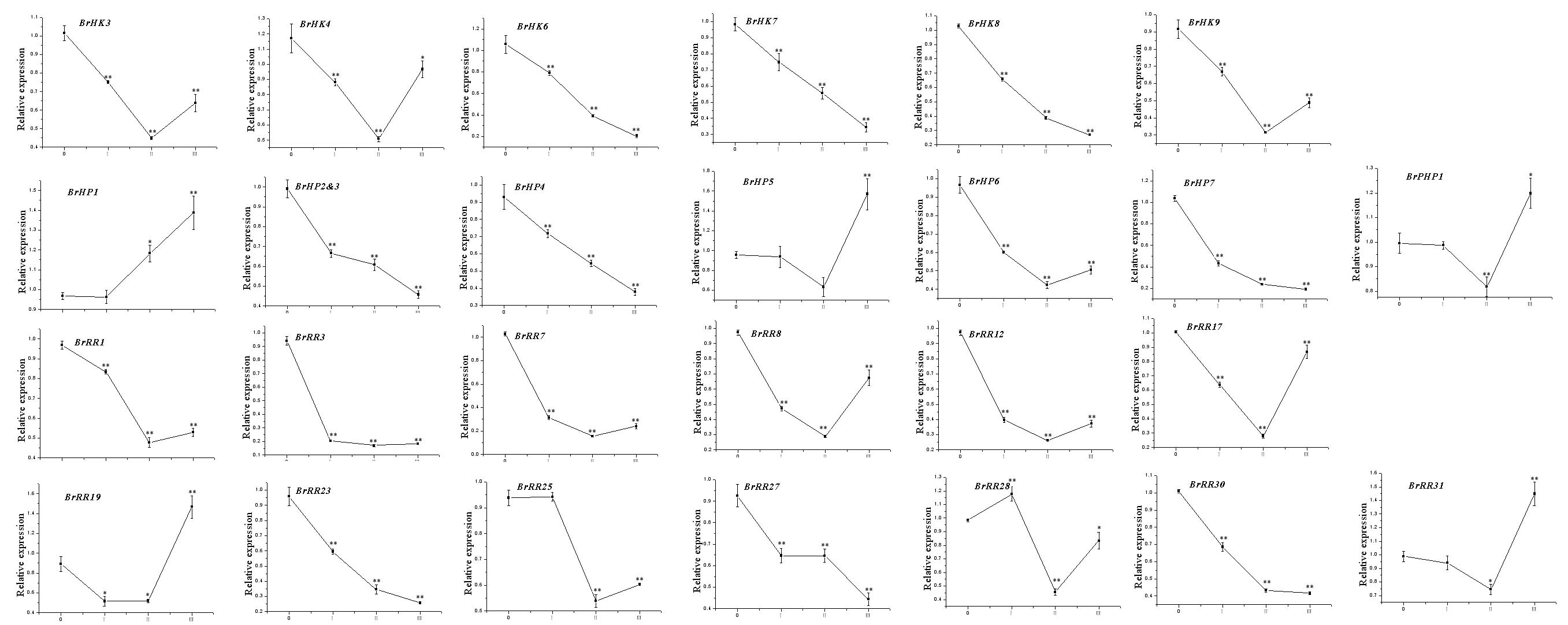

Supplement: Supplementary Data [file supp_dsu004_dsu004supp_fig7.jpg]

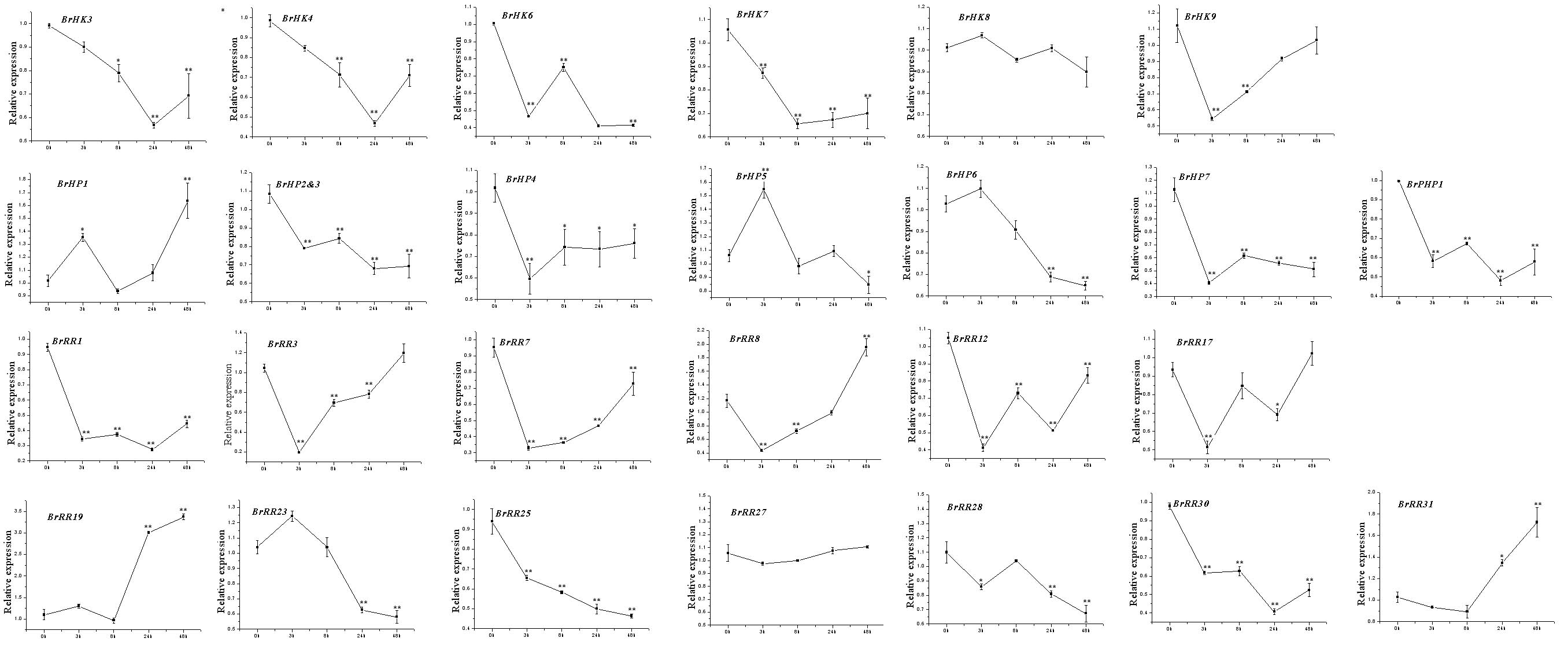

Supplement: Supplementary Data [file supp_dsu004_dsu004supp_fig8.jpg]

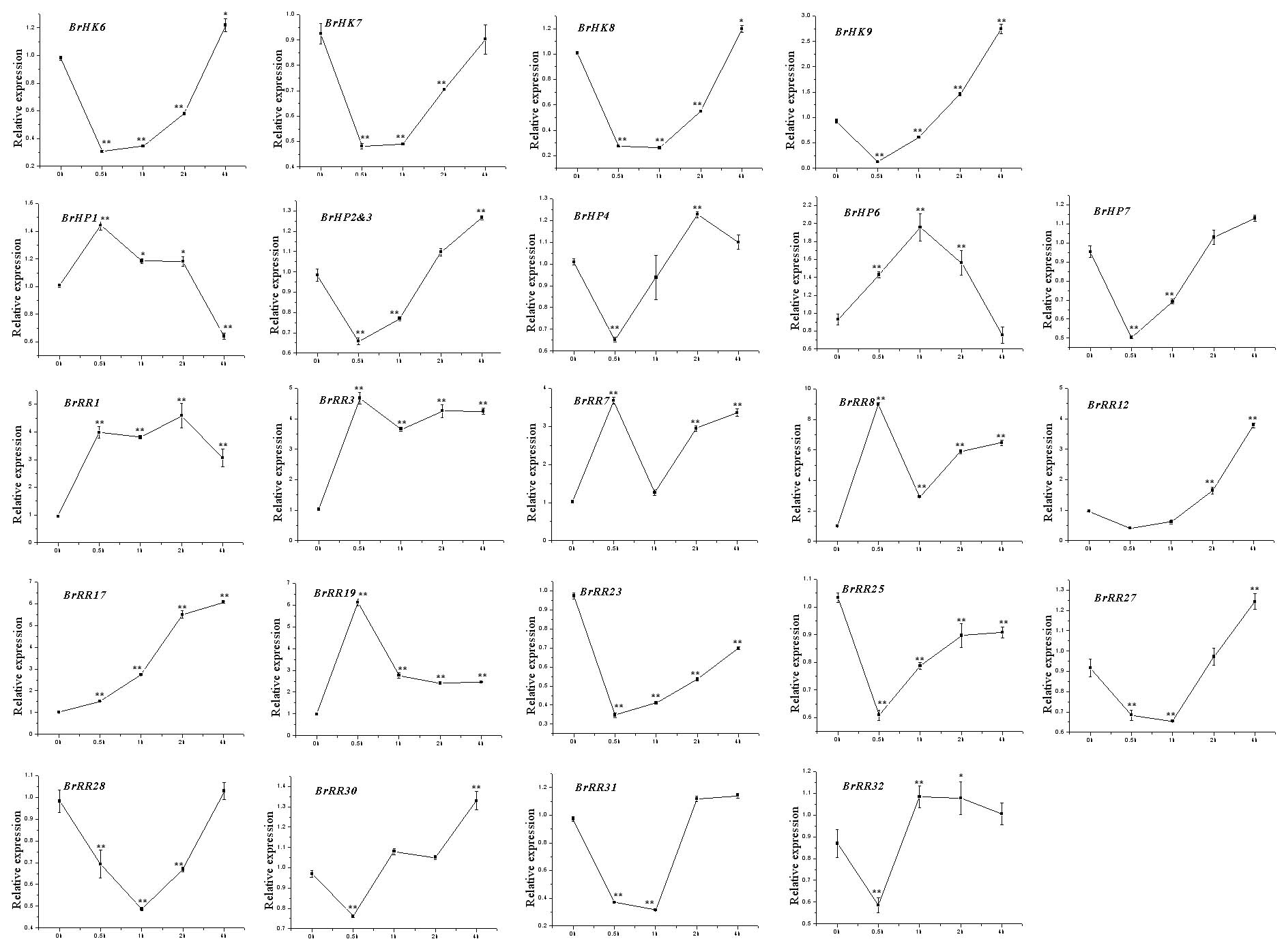

Supplement: Supplementary Data [file supp_dsu004_dsu004supp_fig9.jpg]

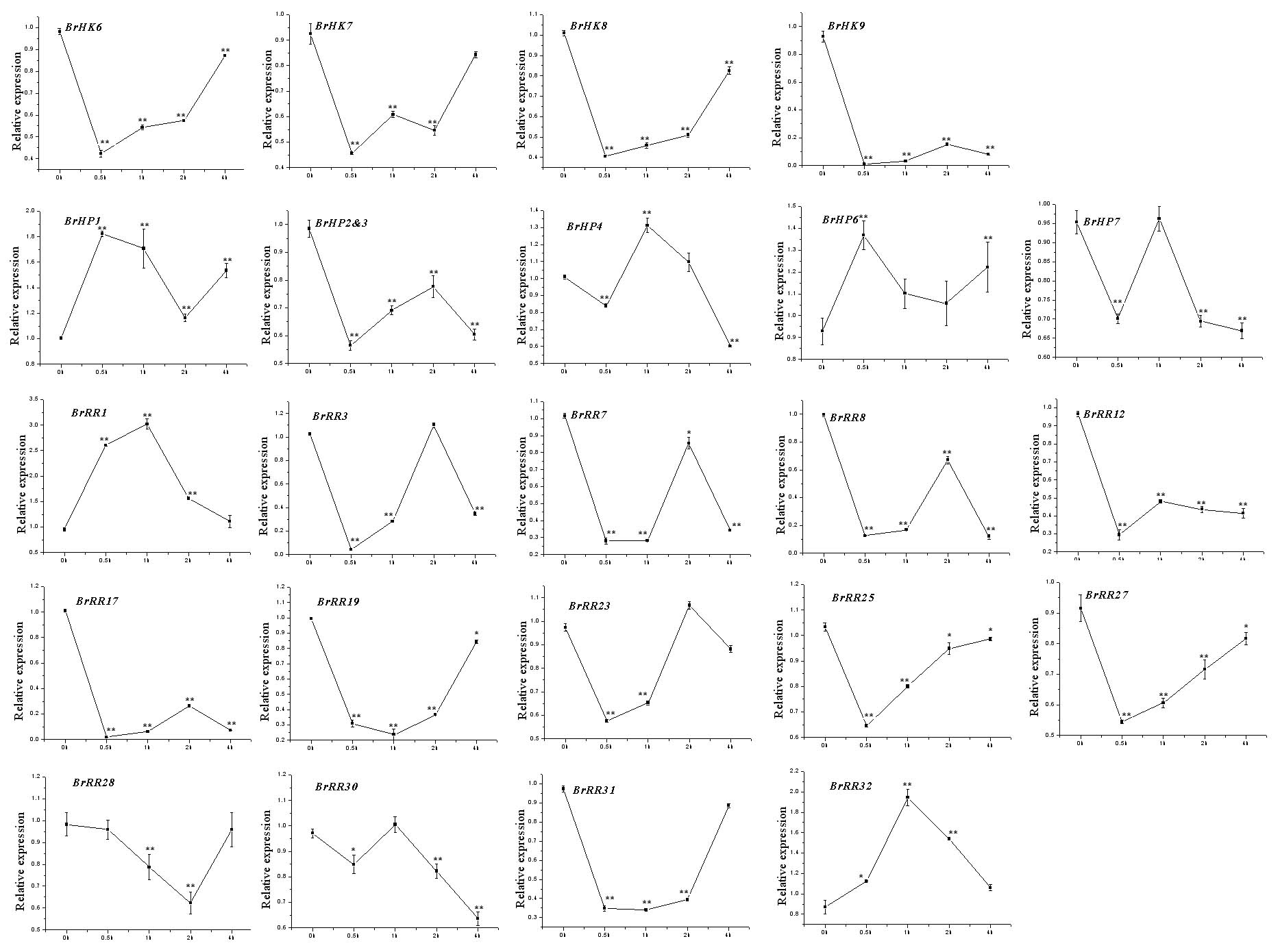

Supplement: Supplementary Data [file supp_dsu004_dsu004supp_fig10.jpg]
