# Supplementary material for: Genome-Wide Identification, Phylogeny, Duplication, and Expression Analyses of Two-Component System Genes in Chinese Cabbage (Brassica rapa ssp. pekinensis)
Source: DNA Res. 2014 Feb 27;21(4):379–96. doi: 10.1093/dnares/dsu004 (PMC4131832; doi:10.1093/dnares/dsu004)
Supplement: Supplementary Data [file supp_dsu004_dsu004supp_table1.doc]

Supplementary Table S1. Forward and reverse primers used in the qRT-PCR gene expression studies

| Primer name | Forward sequence | Reverse sequence |
| --- | --- | --- |
| *BrCyp* | AGGAGGAGATTTCACCGC | TCTCTAACGACATCCATCCC |
| *BrUBC30* | TGAAAGAGCAGTGGAGCC | GGTCTGTCTTGTAGGTGTGAGC |
| *BrHK1* | GAACACCAAGCATCCGAAGT | TCACCATACACGCCACAAA |
| *BrHK2* | TATTAGACCTGCTGTTATTACCC | AAATGGCGAGACCGAGAC |
| *BrHK3* | TTCTGTCGCCGATCCTTC | CATTGCCATATTGATGAGCC |
| *BrHK4* | CGAGGCTGTTCAGGTTGA | GGACCATTCCCATTCTCATA |
| *BrHK6* | ATGTGGGCAAACTATGGTATT | ACTCTGTTGTAAGCCTGATGG |
| *BrHK7* | GGATTGGCATTCCTGTTGA | CCCTGTCCCACCGTAAGT |
| *BrHK8* | AAGCAGCAGATGTCGCCAAGT | TGCCTGTGCGGTCCTAACG |
| *BrHK9* | CCGTTGAACGTACTCACGAC | CAAGAAACTGCGATTTAGCC |
| *BrHP5* | TTGGTGCGAGTAAGGTGAA | TGGCTTGCCTGGAAATAA |
| *BrHP6* | GGCAGCAGCACAAGCATC | GCAAGTACGGAATAGTGAGAAAA |
| *BrHP7* | AAGGGTGTTTAAGGTGTCTGC | CGGCTTGGACGATCTGTT |
| *BrPHP1* | CTCCGCAGCCTTAGATTATTG | CGAACCCTACGAGCACCA |
| *BrHP1* | TTGTCTTCCGCAACTTCTG | GCTGGGATCATTCCACCA |
| *BrHP2，3* | TGCCAAGAGGGTGAAAGG | CACCAGCTTGAATGATTTGTTT |
| *BrHP4* | GGGTCCAAAGAATCAAACC | CCACAAAATCAGGGCTACA |
| *BrRR1* | ATCGACAGATGCCTTGAAGAA | GGAGGAGGAGTAACCATAGCC |
| *BrRR3* | GGACTCTTCTCCGCCGTTAT | TCAGGCGACGAAGTCAACA |
| *BrRR7* | GCGTTCTGAGACGTTAAA | TCTCCATCTAATCCAAGGTA |
| *BrRR8* | CTGAGGTTATGCTACCGATGA | TTGAGCAAGCGTTCTATGA |
| *BrRR12* | GAAAGAAAGCGAGAACTCAG | GTAGAGGCAAGACTTCAATCA |
| *BrRR17* | ATGGCACTAGGAGATTTAT | CAACAGTCGTCACTTTACA |
| *BrRR19* | ATCTTTAATGGAGGTGGGT | CTCTGCTGTTGTAACTTTGC |
| *BrRR23* | AGCGTTAGCAGTCACAGGTCA | CACCAATCCCATCTCCGAAT |
| *BrRR25* | AATGGGAATGGAACCAAGAC | CCCTCCACCTACTGGCATC |
| *BrRR27* | CATCGTCGCTTCCTCGTAA | TGCTGGTGACCCTGGAGA |
| *BrRR28* | CAAGAAACGATGAGGCACA | AGGAAGACGATGATGGGT |
| *BrRR30* | TACTTGAAGAGGATCAGTGGGAATC | ACGGGAATTGGTCGGTGG |
| *BrRR31* | TGGGTTAAGAGTCCTTGTCG | GGCTTGGGAGCAGATGGTA |
| *BrRR32* | CATAGCGGACCGAGGAAT | GTAGCACAAGGCAAAGAGGT |
| *BrRR33* | GCAGCATCAACAACGACA | AGTACGGCAGACTTGGATAA |
| *BrRR35* | CTGAGTGGCGTAATGCTGG | GCCGTTTCTTGCGTCTTG |
| *BrRR38* | CTCAACCGGGGCCTAGCA | TTACTTAAATCGCAAGGAATCTCGT |
