# Supplementary material for: Genome-Wide Identification, Phylogeny, Duplication, and Expression Analyses of Two-Component System Genes in Chinese Cabbage (Brassica rapa ssp. pekinensis)
Source: DNA Res. 2014 Feb 27;21(4):379–96. doi: 10.1093/dnares/dsu004 (PMC4131832; doi:10.1093/dnares/dsu004)
Supplement: Supplementary Data [file supp_dsu004_dsu004supp_table2.doc]

Supplementary Table S2. HK(L) proteins in Chinese cabbage

| Gene name^a^ | Locus^b^ | Features^c^ | Family^d^ | Chr^e^ | Length^f^  (aa) | Identity^g^  (%) |
| --- | --- | --- | --- | --- | --- | --- |
| *BrHK1* | *Bra032761* | HK, Rec | *CKI1* like | A04 | 1039 | 64.5 |
| *BrHK2* | *Bra006075* | HK, Rec | *CKI2/AHK5* like | A03 | 934 | 82.5 |
| *BrHK3* | *Bra009011* | HK, Rec | *CKI2/AHK5* like | A10 | 898 | 86.4 |
| *BrHK4* | *Bra028573* | HK, Rec | *CKI2/AHK5* like | A02 | 911 | 85.9 |
| *BrHK5* | *Bra002095* | HK, Rec | *AHK1* like | A10 | 1194 | 86.6 |
| *BrHK6* | *Bra035381* | CHASE, HK, Rec | *AHK2* like | Scaffold000104 | 1149 | 81.0 |
| *BrHK7* | *Bra013186* | CHASE, HK, Rec | *AHK2* like | A03 | 1097 | 68.0 |
| *BrHK8* | *Bra030037* | CHASE, HK, Rec | *AHK3* like | A07 | 1017 | 84.3 |
| *BrHK9* | *Bra024849* | CHASE, HK, Rec | *AHK4* like | A06 | 1040 | 86.2 |
| *BrHK10* | *Bra004160* | C2H4, HK, Rec | *ETR1* like | A07 | 736 | 95.7 |
| *BrHK11* | *Bra004449* | C2H4, HK | *ERS1* like | A05 | 611 | 94.0 |
| *BrHKL1* | *Bra023756* | C2H4, HKL, Rec | *ETR2* like | A01 | 780 | 85.6 |
| *BrHKL2* | *Bra040134* | C2H4, HKL, Rec | *EIN4* like | A01 | 766 | 94.1 |
| *BrHKL3* | *Bra015303* | C2H4, HKL | *ERS2* like | A10 | 616 | 77.7 |
| *BrHKL4* | *Bra030564* | C2H4, HKL | *ERS2* like | A08 | 1683 | 29.9 |
| *BrHKL5* | *Bra020013* | PHY, HKL | *PHYA* like | A06 | 1210 | 80.2 |
| *BrHKL6* | *Bra031672* | PHY, HKL | *PHYA* like | A09 | 1121 | 83.9 |
| *BrHKL7* | *Bra022192* | PHY, HKL | *PHYB* like | A05 | 1192 | 91.0 |
| *BrHKL8* | *Bra039485* | PHY, HKL | *PHYC* like | A05 | 1117 | 88.2 |
| *BrHKL9* | *Bra013286* | PHY, HKL | *PHYE* like | A01 | 1114 | 88.0 |

^a^Gene names given in this work.

^b^Locus represented by the *B. rapa* genome database.

^c^Features indicate conserved histidine–kinase domain (HK), diverged histidine–kinase-like domain (HKL), receiver domain (Rec), CHASE domain for cytokinin binding (CHASE), ethylene-binding domain (C2H4), and chromophore-binding domain (PHY).

^d^Family indicates classification based on the highest amino acid sequence identity with the *Arabidopsis* counterpart.

^e^Chr represents chromosome localization of the corresponding genes.

^f^Length indicates the numbers of amino acids of the proteins.

^g^Identity to the closest *Arabidopsis* orthologue.
