# Supplementary material for: Genome-Wide Identification, Phylogeny, Duplication, and Expression Analyses of Two-Component System Genes in Chinese Cabbage (Brassica rapa ssp. pekinensis)
Source: DNA Res. 2014 Feb 27;21(4):379–96. doi: 10.1093/dnares/dsu004 (PMC4131832; doi:10.1093/dnares/dsu004)
Supplement: Supplementary Data [file supp_dsu004_dsu004supp_table3.doc]

Supplementary Table S3. HP proteins in Chinese cabbage

| Gene name^a^ | Locus^b^ | Features^c^ | Family^d^ | Chr^e^ | Length^f^  (aa) | Identity^g^  (%) |
| --- | --- | --- | --- | --- | --- | --- |
| *BrHP1* | *Bra023876* | HPt | *AHP1* like | A01 | 154 | 94.8 |
| *BrHP2* | *Bra036215* | HPt | *AHP2* like | A09 | 156 | 86.5 |
| *BrHP3* | *Bra025394* | HPt | *AHP2* like | A06 | 156 | 90.4 |
| *BrHP4* | *Bra028236* | HPt | *AHP3* like | A04 | 178 | 74.2 |
| *BrHP5* | *Bra027169* | HPt | *AHP4* like | A05 | 124 | 72.4 |
| *BrHP6* | *Bra001629* | HPt | *AHP4* like | A03 | 136 | 71.0 |
| *BrHP7* | *Bra033398* | HPt | *AHP5* like | A10 | 156 | 93.6 |
| *BrPHP1* | *Bra003551* | Pseudo-HPt | *AHP6* like | A07 | 154 | 96.1 |

^a^Gene names given in this work.

^b^Locus represented by the *B. rapa* genome database.

^c^Features indicate whether the proteins possess a conserved His-containing phosphotransfer domain (HPt) or a pseudo-HPt lacking the His phosphorylation site.

^d^Family indicates classification based on the highest amino acid sequence identity with the *Arabidopsis* counterpart.

^e^Chr represents chromosome localization of the corresponding genes.

^f^Length indicates the numbers of amino acids of the proteins.

^g^Identity to the closest *Arabidopsis* orthologue.
