# Supplementary material for: Genome-Wide Identification, Phylogeny, Duplication, and Expression Analyses of Two-Component System Genes in Chinese Cabbage (Brassica rapa ssp. pekinensis)
Source: DNA Res. 2014 Feb 27;21(4):379–96. doi: 10.1093/dnares/dsu004 (PMC4131832; doi:10.1093/dnares/dsu004)
Supplement: Supplementary Data [file supp_dsu004_dsu004supp_table4.doc]

Supplementary Table S4. RR proteins in Chinese cabbage

| Gene name^a^ | Locus^b^ | Features^c^ | Family^d^ | Chr^e^ | Length^f^  (aa) | Identity^g^  (%) |
| --- | --- | --- | --- | --- | --- | --- |
| Type-A RR | | | | | | |
| *BrRR1* | *Bra027829* | Rec | *ARR3* like | A09 | 226 | 78.4 |
| *BrRR2* | *Bra031714* | Rec | *ARR4* like | A09 | 261 | 86.7 |
| *BrRR3* | *Bra018439* | Rec | *ARR4* like | A05 | 251 | 83.0 |
| *BrRR4* | *Bra019932* | Rec | *ARR4* like | A06 | 253 | 82.0 |
| *BrRR5* | *Bra033773* | Rec | *ARR5* like | A01 | 180 | 95.1 |
| *BrRR6* | *Bra019524* | Rec | *ARR5* like | A06 | 134 | 65.2 |
| *BrRR7* | *Bra018084* | Rec | *ARR5* like | A06 | 179 | 91.8 |
| *BrRR8* | *Bra010132* | Rec | *ARR6* like | A06 | 190 | 82.6 |
| *BrRR9* | *Bra025708* | Rec | *ARR7* like | A06 | 213 | 80.3 |
| *BrRR10* | *Bra016526* | Rec | *ARR7* like | A08 | 207 | 82.6 |
| *BrRR11* | *Bra000224* | Rec | *ARR8* like | A03 | 216 | 85.0 |
| *BrRR12* | *Bra016943* | Rec | *ARR8* like | A04 | 222 | 83.3 |
| *BrRR13* | *Bra004615* | Rec | *ARR8* like | A05 | 214 | 83.2 |
| *BrRR14* | *Bra014649* | Rec | *ARR9* like | A04 | 234 | 74.8 |
| *BrRR15* | *Bra003265* | Rec | *ARR9* like | A07 | 240 | 78.8 |
| *BrRR16* | *Bra007295* | Rec | *ARR9* like | A09 | 236 | 76.5 |
| *BrRR17* | *Bra015885* | Rec | *ARR15* like | A07 | 185 | 74.4 |
| *BrRR18* | *Bra003782* | Rec | *ARR15* like | A07 | 197 | 77.4 |
| *BrRR19* | *Bra000199* | Rec | *ARR16* like | A03 | 161 | 88.5 |
| *BrRR20* | *Bra007242* | Rec | *ARR17* like | A09 | 169 | 71.2 |
| *BrRR21* | *Bra014695* | Rec | *ARR17* like | A04 | 127 | 75.0 |
| Type-B RR | | | | | | |
| *BrRR22* | *Bra001641* | Rec | *ARR1* like | A03 | 148 | 25.2 |
| *BrRR23* | *Bra022183* | Rec, Myb | *ARR1* like | A05 | 681 | 82.3 |
| *BrRR24* | *Bra001643* | Rec, Myb | *ARR1* like | A03 | 650 | 75.2 |
| *BrRR25* | *Bra033527* | Rec, Myb | *ARR2* like | A01 | 633 | 73.2 |
| *BrRR26* | *Bra012743* | Rec, Myb | *ARR2* like | A03 | 592 | 65.9 |
| *BrRR27* | *Bra023972* | Rec, Myb | *ARR10* like | A03 | 518 | 70.5 |
| *BrRR28* | *Bra004245* | Rec, Myb | *ARR11* like | A07 | 512 | 81.8 |
| *BrRR29* | *Bra004076* | Rec, Myb | *ARR11* like | A07 | 509 | 81.7 |
| *BrRR30* | *Bra032035* | Rec, Myb | *ARR12* like | A04 | 581 | 80.5 |
| *BrRR31* | *Bra026635* | Rec, Myb | *ARR14* like | A02 | 377 | 71.6 |
| *BrRR32* | *Bra020390* | Rec, Myb | *ARR18* like | A02 | 623 | 68.2 |
| *BrRR33* | *Bra014172* | Rec, Myb | *ARR19* like | A08 | 449 | 51.7 |
| *BrRR34* | *Bra032275* | Rec, Myb | *ARR19* like | A05 | 505 | 34.5 |
| *BrRR35* | *Bra041027* | Rec, Myb | *ARR20* like | A09 | 420 | 42.7 |
| *BrRR36* | *Bra005928* | Rec, Myb | *ARR21* like | A03 | 759 | 54.5 |
| *BrRR37* | *Bra009284* | Rec, Myb | *ARR21* like | A10 | 773 | 52.3 |
| *BrRR38* | *Bra028705* | Rec, Myb | *ARR21* like | A02 | 767 | 50.4 |
| Type-C RR | | | | | | |
| *BrRR39* | *Bra001099* | Rec | *ARR22* like | A03 | 143 | 79.2 |
| *BrRR40* | *Bra040204* | Rec | *ARR22* like | Scaffold000191 | 136 | 78.9 |
| *BrRR41* | *Bra020537* | Rec | *ARR24* like | A02 | 134 | 81.3 |
| *BrRR42* | *Bra036579* | Rec | *ARR24* like | A09 | 134 | 84.2 |
| Pseudo RR | | | | | | |
| *BrPRR1* | *Bra035933* | Pseudo-Rec, CCT | *APRR1* like | A09 | 509 | 71.6 |
| *BrPRR2* | *Bra012964* | Pseudo-Rec, CCT | *APRR1* like | A03 | 576 | 73.5 |
| *BrPRR3* | *Bra002512* | Pseudo-Rec, CCT | *APRR3* like | A10 | 484 | 69.9 |
| *BrPRR4* | *Bra009768* | Pseudo-Rec, CCT | *APRR5* like | A06 | 563 | 63.1 |
| *BrPRR5* | *Bra036517* | Pseudo-Rec, CCT | *APRR5* like | A09 | 497 | 60.0 |
| *BrPRR6* | *Bra029407* | Pseudo-Rec, CCT | *APRR5* like | A02 | 509 | 60.0 |
| *BrPRR7* | *Bra028861* | Pseudo-Rec, CCT | *APRR7* like | A02 | 638 | 73.6 |
| *BrPRR8* | *Bra009565* | Pseudo-Rec, CCT | *APRR7* like | A10 | 704 | 81.5 |
| *BrPRR9* | *Bra004507* | Pseudo-Rec, CCT | *APRR9* like | A05 | 412 | 65.8 |
| *BrPRR10* | *Bra040484* | Pseudo-Rec, CCT | *APRR9* like | A04 | 824 | 41.7 |
| *BrPRR11* | *Bra013277* | Pseudo-Rec, Myb | *APRR2* like | A01 | 503 | 78.6 |
| *BrPRR12* | *Bra012623* | Pseudo-Rec, Myb | *APRR2* like | A03 | 513 | 75.5 |
| *BrPRR13* | *Bra037427* | Pseudo-Rec, Myb | *APRR4* like | A06 | 273 | 54.6 |
| *BrPRR14* | *Bra020652* | Pseudo-Rec, Myb | *APRR4* like | A02 | 441 | 32.3 |
| *BrPRR15* | *Bra004306* | Pseudo-Rec | *APRR6* like | A07 | 541 | 30.2 |

^a^Gene names given in this work.

^b^Locus represented by the *B. rapa* genome database.

^c^Features indicate receiver domain (Rec), pseudo-receiver domain (Pseudo-Rec) lacking the conserved D, Myb-*like* domain (Myb), plant-specific CCT motif found in clock proteins.

^d^Family indicates classification based on the highest amino acid sequence identity with the *Arabidopsis* counterpart.

^e^Chr represents chromosome localization of the corresponding genes.

^f^Length indicates the numbers of amino acids of the proteins.

^g^Identity to the closest *Arabidopsis* orthologue.
