# Supplementary material for: Genome-Wide Identification, Phylogeny, Duplication, and Expression Analyses of Two-Component System Genes in Chinese Cabbage (Brassica rapa ssp. pekinensis)
Source: DNA Res. 2014 Feb 27;21(4):379–96. doi: 10.1093/dnares/dsu004 (PMC4131832; doi:10.1093/dnares/dsu004)
Supplement: Supplementary Data [file supp_dsu004_dsu004supp_table5.doc]

Supplementary Table S5. HKL proteins in soybean

| Gene name^a^ | Locus^b^ | Features^c^ | Family^d^ | Chr^e^ | Length^f^  (aa) | Identity^g^  (%) |
| --- | --- | --- | --- | --- | --- | --- |
| *GmHKL1* | *Glyma10g33240* | C2H4, HKL, Rec | *ETR2* | A10 | 737 | 65 |
| *GmHKL2* | *Glyma20g34420* | C2H4, HKL, Rec | *ETR2* | A20 | 762 | 58 |
| *GmHKL3* | *Glyma20g21780* | C2H4, HKL, Rec | *ETR2* | A20 | 696 | 64 |
| *GmHKL4* | *Glyma03g41220* | C2H4, HKL, Rec | *EIN4* | A03 | 760 | 59 |
| *GmHKL5* | *Glyma19g43840* | C2H4, HKL, Rec | *EIN4* | A19 | 731 | 59 |
| *GmHKL6* | *Glyma10g31040* | C2H4, HKL, Rec | *EIN4* | A10 | 767 | 58 |
| *GmHKL7* | *Glyma20g36440* | C2H4, HKL, Rec | *EIN4* | A20 | 766 | 58 |
| *GmHKL8* | *Glyma10g28170* | PHY, HKL | *PHYA* like | A10 | 1130 | 74 |
| *GmHKL9* | *Glyma20g22160* | PHY, HKL | *PHYA* like | A20 | 1123 | 76 |
| *GmHKL10* | *Glyma19g41210* | PHY, HKL | *PHYA* like | A19 | 1130 | 71 |
| *GmHKL11* | *Glyma03g38620* | PHY, HKL | *PHYA* like | A03 | 996 | 64 |
| *GmHKL12* | *Glyma09g03990* | PHY, HKL | *PHYB like* | A09 | 1137 | 77 |
| *GmHKL13* | *Glyma15g14980* | PHY, HKL | *PHYB like* | A15 | 1149 | 76 |
| *GmHKL14* | *Glyma09g11600* | PHY, HKL | *PHYE like* | A09 | 1120 | 62 |
| *GmHKL15* | *Glyma15g23400* | PHY, HKL | *PHYE like* | A15 | 831 | 57 |

^a^Gene names given in this work.

^b^Locus represented by the *B. rapa* genome database.

^c^Features indicate conserved histidine–kinase domain (HK), diverged histidine–kinase-like domain (HKL), receiver domain (Rec), ethylene-binding domain (C2H4), and chromophore-binding domain (PHY).

^d^Family indicates classification based on the highest amino acid sequence identity with the *Arabidopsis* counterpart.

^e^Chr represents chromosome localization of the corresponding genes.

^f^Length indicates the numbers of amino acids of the proteins.

^g^Identity to the closest *Arabidopsis* orthologue.
